# TMEM106B Puncta Is Increased in Multiple Sclerosis Plaques, and Reduced Protein in Mice Results in Delayed Lipid Clearance Following CNS Injury

**DOI:** 10.3390/cells12131734

**Published:** 2023-06-27

**Authors:** Bridget Shafit-Zagardo, Simone Sidoli, James E. Goldman, Juwen C. DuBois, John R. Corboy, Stephen M. Strittmatter, Hillary Guzik, Ukuemi Edema, Anita G. Arackal, Yair M. Botbol, Emilio Merheb, Rashed M. Nagra, Sarah Graff

**Affiliations:** 1 Department of Pathology, Albert Einstein College of Medicine, New York, NY 10461, USA; 2 Department of Biochemistry, Albert Einstein College of Medicine, New York, NY 10461, USA; 3 Department of Pathology and Cell Biology, Columbia University College of Physicians and Surgeons, New York, NY 10032, USA; 4 Rocky Mountain MS Brain Bank, Department of Neurology, University of Colorado School of Medicine, Aurora, CO 80045, USA; 5 Departments of Neurology and Neuroscience, Yale School of Medicine, Boyer Center for Molecular Medicine, New Haven, CT 06510, USA; 6 Analytic Imaging Facility, Albert Einstein College of Medicine, New York, NY 10461, USA; 7 Department of Anatomic and Clinical Pathology, Montefiore Medical Center, Bronx, NY 10467, USA; 8 UCLA Brain Bank, VA Healthcare System, Los Angeles, CA 90073, USA

**Keywords:** multiple sclerosis (MS), TMEM106B, myelin oligodendrocyte glycoprotein (MOG)-induced EAE, demyelination, lipids

## Abstract

During inflammatory, demyelinating diseases such as multiple sclerosis (MS), inflammation and axonal damage are prevalent early in the course. Axonal damage includes swelling, defects in transport, and failure to clear damaged intracellular proteins, all of which affect recovery and compromise neuronal integrity. The clearance of damaged cell components is important to maintain normal turnover and restore homeostasis. In this study, we used mass spectrometry to identify insoluble proteins within high-speed/mercaptoethanol/sarcosyl-insoluble pellets from purified white matter plaques isolated from the brains of individuals with relapsing–remitting MS (RRMS). We determined that the transmembrane protein 106B (TMEM106B), normally lysosome-associated, is insoluble in RRMS plaques relative to normal-appearing white matter from individuals with Alzheimer’s disease and non-neurologic controls. Relative to wild-type mice, hypomorphic mice with a reduction in TMEM106B have increased axonal damage and lipid droplet accumulation in the spinal cord following myelin-oligodendrocyte-glycoprotein-induced experimental autoimmune encephalomyelitis. Additionally, the corpora callosa from cuprizone-challenged hypomorphic mice fail to clear lipid droplets efficiently during remyelination, suggesting that when TMEM106B is compromised, protein and lipid clearance by the lysosome is delayed. As TMEM106B contains putative lipid- and LC3-binding sites, further exploration of these sites is warranted.

## 1. Introduction

Multiple sclerosis (MS) is a debilitating neurological disease with most individuals diagnosed between the ages of 20 and 50 [1,2,3,4,5]. In the United States, there are an estimated one million individuals living with MS [1], (www.nationalmssociety.org accessed on 20 May 2023). During inflammatory attacks, immune cells cross the protected blood–brain barrier and invade the nervous system, resulting in demyelination and axonal damage in the brain and spinal cord [6,7,8,9]. When prolonged by inefficient clearance of cellular damage and myelin debris, the repair processes are delayed [10,11,12]. Over time, the progressive nature of MS results in a chronic disease course with permanent motor damage, demyelination, axonal damage, neuronal loss, OilRedO+ lipid inclusions, and oligodendrocyte cell death [3,10,13,14]. Thus, a major obstacle for individuals with MS is the maintenance of axonal function, and the ability to recover following a relapse.

**Figure 1:**
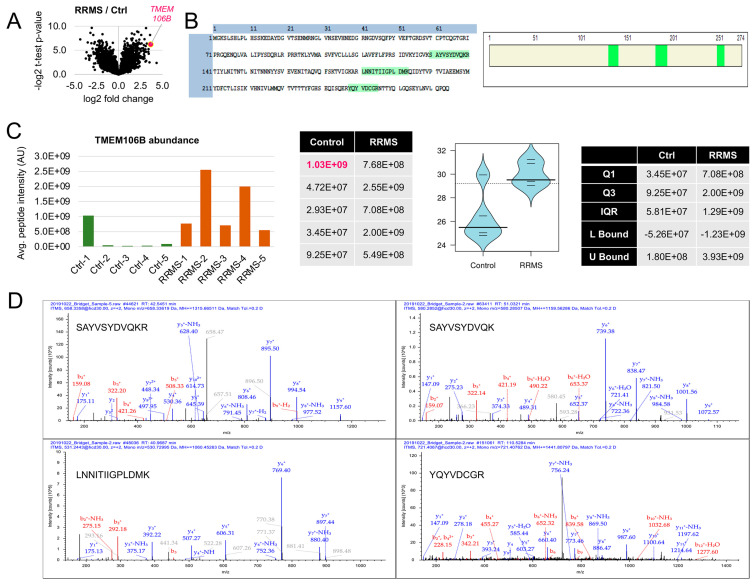
TMEM106B is elevated in protein pellets isolated from white matter plaques of individuals with RRMS. Identification and quantification of TMEM106B via mass-spectrometry-based proteomics. (**A**) Volcano plot displaying log2 fold change and −log2 *t*-test *p*-values for the comparison of RRMS vs. control samples. TMEM106B is highlighted in red. The distribution of the data points shows upregulated and downregulated proteins, including several non-significant ones (significance threshold set at 4.32 = −log2(0.05)). The mean log2 of TMEM106B peptides identified in the sarcosyl-insoluble protein pellets from plaques of individuals with RRMS relative to non-neurologic controls following nanoLC-ms/ms analysis of peptides on an Orbitrap Fusion Lumos mass spectrometer (Thermo Fisher Scientific, Fair Lawn, NJ, USA). The mean age of the non-neurologic control group was 63.2 ± 3.0, and the mean age of RRMS was 64.8 ± 1.3 years. The mean age of each group and the post-mortem intervals were not significantly different. Mann–Whitney U test, 24, *p* = 0.5476. (**B**) Coverage of the protein TMEM106B based on confidently identified peptides. The first two peptides are in the same region of the protein, but they differ by one missed cleavage of the tryptic digestion. (**C**) Quantification of the protein TMEM106B using the average signal intensity of the four identified peptides. From left to right: protein abundance for each of the replicates as a bar plot, raw values as a table, bean plot representation of the log2 transformed values, and outlier analysis of the data distribution. The upper bound of the control (Ctrl) distribution is smaller than the first replicate value, and therefore we categorized it as an outlier. Without the outlier, the Mann–Whitney test is 0.0071; including the outlier the *p*-value, it is 0.035. The box plot shows the distribution of the data. Quartile (Q) values represent the quarters with the interquartile range (IQR), identifying 50% of the data. (**D**) Annotated MS/MS spectra for each of the identified peptides. Fragment ion signals highlighted in red represent the b-series fragments (N-terminus), while those in blue are the y-series fragments (C-terminus).

In our current study, we identified increased levels of transmembrane protein 106B (TMEM106B) protein in sarcosyl-insoluble protein precipitates in white matter plaques from individuals with relapsing–remitting multiple sclerosis (RRMS) relative to the white matter regions of normal-appearing, subcortical white matter from non-neurologic control brains (Figure 1). Prior to this study, there have been no reports of insoluble TMEM106B in MS plaques. TMEM106B is a 274-amino-acid glycoprotein expressed in neurons and glia [15,16,17,18,19,20]. TMEM106B regulates lysosomal trafficking and lysosomal acidification [21]. The glycosylated carboxy-terminus faces the lysosomal lumen, and the 96-amino-acid amino-terminus is within the cytosol [21]. The first 42 amino acids of TMEM106B are a region of high disorder permitting interaction with other proteins [21]. TMEM106B co-localizes with LAMP1 on lysosomes and binds progranulin and the microtubule-associated protein MAP6 [22]. Over-expression and deletion of TMEM106B in vitro result in varying pathologic consequences. In vivo, TMEM106B has opposing effects in mouse models of lysosomal diseases where it was found that TMEM106B differentially modulates the progression of the lysosomal storage diseases Gaucher disease and neuronal ceroid lipofuscinosis [23]. Mice with deletion of TMEM106B and point mutations demonstrate functional defects in lysosomes, axonal damage, neurodegeneration, and hypomyelination [24,25,26].

The increased insoluble TMEM106B led us to examine the consequence of reducing TMEM106B in the inflammatory model of experimental autoimmune encephalomyelitis (EAE) [27], and the cuprizone model [28], to determine if decreased TMEM106B in a hypomorphic mouse impacts the clearance of lipids. We used TMEM106B (trap/trap; TMEM106B^t/t^) knockin mice, established by a gene trap insertion of *lacz* between exons 3 and 4 generating a hypomorphic knockin strain with reduced levels of total TMEM106B protein [23]. EAE is the most commonly studied model for MS [27,29,30,31]. Our study shows that when TMEM106B^t/t^ mice are sensitized with myelin oligodendrocyte glycoprotein_35-55_ (MOG_35-55_) peptide, lumbar spinal cords show increased lipid droplet accumulation, axonal damage, and myelin loss relative to wild-type (WT) mice during chronic disease.

## 2. Materials and Methods

### 2.1. Isolation of Insoluble Proteins from Human White Matter

All sections were dorsal–lateral frontal subcortical white matter. Subcortical white matter lesions and normal appearing white matter were dissected and β-mercaptoethanol-, sarkosyl-insoluble proteins were purified using the isolation procedure from [32], followed by sonication, tryptic enzymatic digestion, and nanoLC-ms/ms analysis of the peptides using an Orbitrap Fusion Lumos mass spectrometer (Thermo Fisher Scientific). Subcortical white matter lesions from five individuals with relapsing–remitting multiple sclerosis (RRMS) and normal-appearing subcortical white matter (NAWM) from non-neurologically impaired individuals were compared. RRMS and non-neurological controls (NNCs) were obtained from the Rocky Mountain MS Center Tissue Bank. Supplemental Table S1 lists the cases studied.

### 2.2. Protein Extraction and Sample Preparation

To analyze the proteome, the insoluble proteins in the pellet were processed for protein digestion using S-Trap filters (Protifi), according to the manufacturer’s instructions. Briefly, the pellet was mixed with 5% SDS, followed by incubation with 5 mM DTT for 1 h, and then 20 mM iodoacetamide for 30 min in the dark to reduce and alkylate proteins. Phosphoric acid was added to the samples at a final concentration of 1.2%. The samples were diluted in six volumes of binding buffer (90% methanol and 10 mM ammonium bicarbonate, pH 8.0). After gently mixing, the protein solution was loaded onto an S-Trap filter and spun at 500× *g* for 30 s. The samples were washed twice with a binding buffer. Finally, 1 µg of sequencing grade trypsin (Promega), diluted in 50 mM ammonium bicarbonate, was added into the S-trap filter and the samples were digested overnight at 37 °C. Peptides were eluted in three steps: (i) 40 µL of 50 mM ammonium bicarbonate, (ii) 40 µL of 0.1% trifluoroacetic acid (TFA), and (iii) 40 µL of 60% acetonitrile and 0.1% TFA. The peptide solution was pooled, spun at 1000× *g* for 30 s, and dried in a vacuum centrifuge.

### 2.3. Sample Desalting

Prior to mass spectrometry analysis, the samples were desalted using a 96-well filter plate (Orochem) packed with 1 mg of Oasis HLB C-18 resin (Waters). Briefly, the samples were resuspended in 100 µL of 0.1% TFA and loaded onto the HLB resin, which had been previously equilibrated using 100 µL of the same buffer. After washing with 100 µL of 0.1% TFA, the samples were eluted with a buffer containing 70 µL of 60% acetonitrile and 0.1% TFA, and then dried in a vacuum centrifuge.

### 2.4. LC-MS/MS Acquisition

The samples were resuspended in 10 µL of 0.1% TFA and loaded onto a Dionex RSLC Ultimate 300 (Thermo Scientific), coupled online with an Orbitrap Fusion Lumos (Thermo Scientific). Chromatographic separation was performed with a two-column system, consisting of a C-18 trap cartridge (300 µm ID, 5 mm length) and a picofrit analytical column (75 µm ID, 25 cm length) packed in-house with reversed-phase Repro-Sil Pur C18-AQ 3 µm resin. To analyze the proteome, peptides were separated using a 120 min gradient of 4–30% buffer B (buffer A: 0.1% formic acid; buffer B: 80% acetonitrile + 0.1% formic acid) at a flow rate of 300 nL/min. The mass spectrometer was set to acquire spectra in a data-dependent acquisition (DDA) mode. Briefly, the full MS scan was set to 300–1200 *m*/*z* in the orbitrap with a resolution of 120,000 (at 200 *m*/*z*) and an AGC target of 5 × 10^−5^. MS/MS was performed in the ion trap using the top speed mode (2 s), an AGC target of 1 × 10^4^, and an HCD collision energy of 30.

### 2.5. Proteomics Data Analysis

Proteome raw files were searched using Proteome Discoverer software (v2.4, Thermo Scientific), using the SEQUEST search engine, and the SwissProt human database (updated February 2020). The search for total proteome included variable modification of N-terminal acetylation and fixed modification of carbamidomethyl cysteine. Trypsin was specified as the digestive enzyme with two missed cleavages allowed. Mass tolerance was set to 10 ppm for precursor ions and 0.02 Da for product ions. The peptide and protein false discovery rate was set to 1%. Prior to statistical analysis, proteins were log2 transformed, normalized by the average value of each sample, and missing values were imputed using a normal distribution 2 standard deviations lower than the mean. Statistical regulation was assessed using a heteroscedastic *t*-test (if *p*-value < 0.05). The mass spectrometry raw data were uploaded to the repository Chorus (chorusproject.org) under project number 1814.

### 2.6. Stains and Antibodies

#### 2.6.1. Myelin Stain

The Brain Stain Imaging kit (B34650; Molecular Probes, Eugene, OR, USA) was used to stain the myelin (FluoroMyelin Green), nuclei, and neurons (Nissl) in the frozen brain sections.

#### 2.6.2. Antibodies

Antibody SMI32 (Millipore; 1:10,000) recognizes non-phosphorylated neurofilament protein in damaged and swollen axons. SMI99 (Millipore; 1:1000) recognizes myelin basic protein (MBP). Iba1 rabbit polyclonal (WAKO, for IHC; 019-19741; 1:400), and Iba1 monoclonal antibody (Millipore, 1:300) recognize microglia/macrophages. An affinity-purified TMEM106B antibody was purchased from Proteintech (rabbit polyclonal 290995-1-AP; 1:100). APC/CC1 (OP80 Millipore; 1:20) recognizes mature oligodendrocytes. Carbonic anhydrase II (CAII; ab124687; Abcam; 1:250) recognizes oligodendrocytes. LC3(A/B) (D3U4C) XP Rabbit monoclonal antibody was purchased from Cell Signaling (12,741). β-actin antibody (ab6276; Abcam) was used at 1:150,000 for Western blot analysis. CD11b (A1581, 1:100) and Arginase1 (ARG1, A4923, 1:100) antibodies were purchased from ABclonal Technologies. A rabbit polyclonal to iNOS was purchased from Abcam (ab15323, 1:100). The microglia-specific antibody 4D4 was obtained from Dr. Oleg Butovsky (Harvard Medical School, Brigham and Women’s Hospital; 1:1000). All Alexa-fluorescent secondary antibodies were purchased from Fisher Scientific. 

### 2.7. Immunostaining and Analysis of Human Brain Sections

As noted above, all sections were dorsal–lateral frontal subcortical white matter. Fresh frozen MS and non-neurological control tissue (Rocky Mountain Brain Bank) was used for mass spectrometry and some samples were prepared for immunostaining by formalin fixation and paraffin embedding. Additional formalin-fixed tissue was obtained from the UCLA Brain Bank and was paraffin-embedded or embedded in O.C.T. compound (4583; Tissue-Tek; Sakura; Torrence, CA); 5–7-micron sections were prepared. Alzheimer’s brain tissue was obtained from the New York Brain Bank at Columbia University’s Irving Medical Center, and additional material was obtained from the Montefiore Neuropathology Division of the Department of Pathology. Following the staining of human brain sections, slides were scanned on a P250 slide scanner, and full sections were evaluated and photographed in Caseviewer/Slideviewer. At least 30 random fields of white matter were examined for all cases and representative fields were photographed at ×63 white matter (0.0337 mm^2^) in Caseviewer. Figure legends detail the staining and antibodies. Supplemental Table S1 lists the cases studied.

### 2.8. Mice

TMEM106B^t/t^ mice on a C57Black/6J background were obtained from Dr. Stephen Strittmatter at Yale University [33] and were used to examine two mouse models of CNS damage, the cuprizone model of demyelination/remyelination and myelin oligodendrocyte glycoprotein (MOG)-induced experimental autoimmune encephalomyelitis (EAE). The strain TMEM106B (trap/trap; TMEM106B^t/t^) was established by a gene trap insertion of *lacz* between exons 3 and 4 generating a hypomorphic knockin strain herein referred to as TMEM106B^t/t^. Low amounts of the full-length protein are detected by Western blot analysis [23]. Primers P019 and P020 detect WT. P017 and P020 detect TMEM106B^t/t^. The genotypes were further confirmed by Transnetyx using duplexed PCR assays for both WT and knockout (KO) alleles.

P017: 5′ GGGATCTCATGCTGGAGTTCTTCG 3′

P019: 5′ TTCTCTCCATGTGCTGCATTATGAGC 3′

P020: 5′ ACGTGCTTCTCTCATCTAGAGTTTTCC 3′

The TMEM106B^t/t^ mouse model differs from models with a complete deletion of TMEM106B [24,25,26]. Unlike our strain, a complete knockout of TMEM106B showed motor deficits at 5.5 months of age, while other groups reported oligodendrocyte deficits using the cuprizone model [25,26].

Ethical Statement Regarding Animal Housing and Husbandry, Care, and Monitoring. All the animals were bred, maintained, and treated in the Barrier Facility under the guidance of the Albert Einstein College of Medicine (AECOM) veterinary staff and Dr. Shafit-Zagardo’s approved protocol number 00001158. All procedures are in complete compliance with the AECOM Institutional Review Board and with the NIH Guide for the Care of Laboratory Animals. The mice were continually monitored for any distress including weight loss, changes in excretion, or lethargy. Wherever possible, such as for scoring mice, the testers were blinded to the genotypes of the groups. Experiments were repeated at least twice to ensure consistent findings. Mice undergoing EAE were monitored daily and provided with standard chow pellets and DietGel Recovery (clearh20com) at the cage bottom if they showed hindlimb weakness or paralysis. Mice were sacrificed if they scored 5 (moribund). Each animal acquired a significant value protected by good animal care and by ensuring the animal’s comfort. The approved euthanasia protocol employed was outlined by the Panel on Euthanasia of the American Veterinary Medical Association and IACUC offices. To sacrifice moribund animals, mice received isoflurane in a sealed chamber and were perfused with 4% paraformaldehyde. Both males and females were included in the studies unless otherwise specified.

### 2.9. MOG-Induced EAE

C57Bl6J and TMEM106B^t/t^ mice were immunized with myelin oligodendrocyte glycoprotein (MOG)_35-55_ peptide emulsified in complete Freund’s adjuvant and injected with pertussis toxin on day 0 and day 2 [34]. The mice were monitored daily for clinical signs of EAE and were scored as follows: 0 = no clinical signs of EAE, 1 = flaccid tail, 2 = flaccid tail and hind limb weakness, 3 = hind limb paralysis, 4 = hind limb paralysis and forelimb weakness, and 5 = moribund. Mice that did not present with clinical symptoms were excluded from the analysis.

### 2.10. Cuprizone Treatment

The mice were fed 0.2% (*w*/*w*) cuprizone (Sigma; St. Louis, MO, USA) in powdered chow ad libitum for 5 weeks, followed by regular chow pellets. Mice were sacrificed at 5 weeks to confirm demyelination, and during the recovery phase, 2 or 3 weeks after the removal of cuprizone from the diet. For immunohistochemical analysis, the brains were removed following cardiac perfusion with 4% paraformaldehyde, and paraffin-fixed and frozen sections were prepared. Coronal sections of the brain (10 μm) were cut on a cryostat and the midline of the corpus callosum was evaluated at the region of the fornix corresponding to sections 260–280 of Sidman’s mouse atlas [35].

### 2.11. Oil Red O Staining

The frozen sections were incubated in deionized water for 1 min followed by a 2-min incubation in 100% propylene glycol (Polyscientific; Bayshore, New York, NY, USA) and immediately transferred to Oil Red O stain (Polyscientific) for 48 h at room temperature. The sections were incubated for 1 min in 85% propylene glycol, rinsed in double-distilled deionized water for 1 min, and mounted with glycerin jelly mounting medium (Polyscientific). Mounted cross-sections were scanned on a 3D P250 High-Capacity Slide Scanner. The slides were imaged with a 4-megapixel color CMOS camera with a ×20 objective and then stitched together. The images were analyzed using Case Viewer software (v3.3). Coronal brain sections were graded on a scale of 0–4 where a score of 0 detected no OilRedO+ puncta in the corpus callosum. A minimum of two sections were examined to obtain the score and usually four brain sections were evaluated/mouse. A score of 1 signifies very few small OilRedO+ puncta in a limited region of the corpus callosum, usually on only one side of the midline of the corpus callosum. A score of 2 signifies small OilRedO+ puncta in the left and right regions from the midline of the corpus callosum. A score of 3 signifies abundant OilRedO+ puncta in the left and right of the midline of the corpus callosum. A score of 4 signifies abundant large OilRedO+ puncta throughout the corpus callosum extending into the lateral regions. The OilRedO+ puncta score was determined using GraphPad Prism Software (v9.5.1) and the Mann–Whitney U two-tailed test. Data from three independent experiments were pooled and presented as the median value with an interquartile range. During EAE, coronal spinal cord sections were scored by counting the total number of OilRedO+ puncta throughout the spinal cord parenchyma at ×40. A minimum of two slides with 2–3 spinal cord sections were counted and analysis was performed using GraphPad Prism Software and the Mann–Whitney U two-tailed test.

### 2.12. Histologic Grading

Histological sections were graded on a scale of 0–4 as previously described [36]. For Iba1^+^ staining, cross-sectional spinal cords or brains were scored on a 0–4 inflammatory scale where a score of 0 is the equivalent pathology observed in a naïve mouse, 1 = mild inflammation, 2 = moderate, 3 = severe inflammation, and 4 = very severe inflammation involving 50% or more of the tissue. For relative changes in myelination and to assess decreased MBP staining, the scores were assigned as follows: 0 = MBP immunoreactivity observed in naïve mice, 1 = mild demyelination consisting of a few small white matter areas within the cord parenchyma, usually adjacent to the meninges; 2 = moderate demyelination consisting of at least two regions of the cord, often with multiple ventral white matter involvement on both sides; 3 = severe demyelination consisting of large demyelinated lesions observed with paralysis and ventral and lateral involvement; and 4 = very severe involving >50% of white matter observed with front and hind limb involvement. For relative axonal damage, we either counted all SMI32^+^ axons in the white matter or quantified the SMI32^+^ axonal swellings (>3 µm) in the left and the right ventral region of the white matter of mouse spinal cords using multiple ×20 fields. The scores were assigned as follows: 0 = 0 SMI32^+^ axonal swellings, as observed in naïve mice, 1 = ≤10 SMI32^+^ swellings, 2 = 10–20 SMI32^+^ swellings, 3 = 20–50 SMI32^+^, and 4 = ≥50 SMI32^+^ swellings. The slides were blinded and at least three sections of the lumbar spinal cord for each animal were assessed by two individuals; the number of animals used for each experiment is indicated in the figure legends. The Mann–Whitney *U* test was used to evaluate statistical significance.

### 2.13. Statistical Analysis

All values are presented as mean or median ± standard error of the mean (SEM). Statistical analyses were performed in GraphPad Prism Software. Significance was determined using a two-tailed unpaired Student’s *t*-test for parametric analyses and Mann–Whitney for nonparametric analyses. Multiple group analysis was performed using one-way ANOVA.

### 2.14. Data Availability

The authors confirm that the data supporting the findings of this study are available within the article and its Supplemental Material. The mass spectrometry raw data were uploaded to the repository Chorus (www.chorusproject.org accessed on 20 May 2023) under project number 1814.

## 3. Results

### 3.1. TMEM106B Is Elevated in Insoluble Protein Pellets Isolated from the White Matter Plaques of Individuals with RRMS

Mass spectrometry was used to determine the composition of β-mercaptoethanol/sarkosyl-insoluble proteins within the subcortical white matter of MS plaques relative to those in normal-appearing white matter. We hypothesized that these insoluble proteins are possibly sequestered or unavailable for normal function. While the mass spectrometry studies characterizing other proteins are ongoing, we examined several of the proteins in more detail, including TMEM106B, which we determined to be significantly enriched in the pellets from RRMS plaques (red dot, Figure 1A; *p* = 0.0071, Mann–Whitney U test). The log2 TMEM106B peptides represented in insoluble pellets from non-neurological controls (*n* = 5) and RRMS (*n* = 5) samples were examined from one experiment in which all 10 samples were processed and run at the same time (Supplemental Table S1). Both male and female samples showed no difference in scores within the group. The ranks of the top 50 insoluble proteins are listed in Supplemental Table S2. The unique peptides identified for TMEM106B are shown in Figure 1B,D. A bean plot displays the differences between the non-neurological white matter controls (*n* = 4; one outlier) and those from RRMS plaques (*n* = 5; Figure 1C). The data are presented as median ± interquartile range (1st quartile, 3rd quartile). The median value for the non-neurologic controls was log2 25.79 (25.04, 26.46), and the log2 for RRMS was 30.26 (29.40, 30.90). TMEM106B presence was confirmed and quantified using four peptides confidently identified by mass spectrometry (false discovery rate < 0.01).

### 3.2. White Matter Plaques of Individuals with RRMS Show Increased TMEM106B+ Puncta Relative to the White Matter of Controls

**Figure 2:**
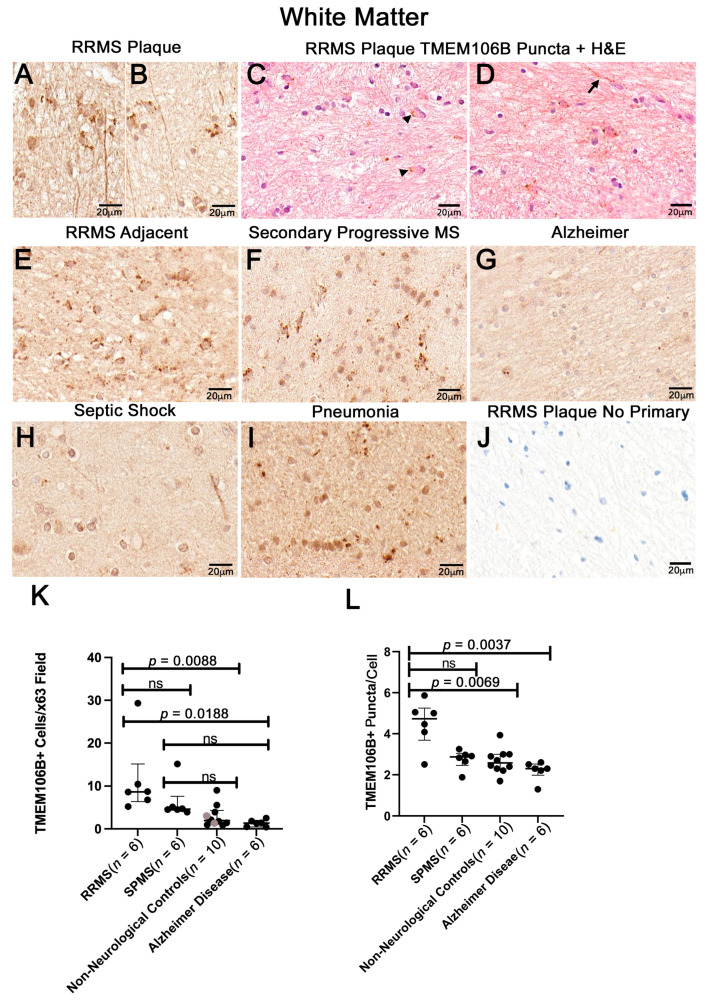
Increased TMEM106B in RRMS plaques. (**A**–**D**) Subcortical white matter sections from RRMS plaques and adjacent white matter (**E**) and SPMS (**F**) brains have abundant TMEM106B+ puncta relative to Alzheimer’s (**G**), and non-neurological white matter controls (**H**,**I**). (**J**) No primary control of an RRMS plaque. (**K**) Statistical analysis of TMEM106B+ cells/(×63) 0.0337 mm^2^/field, 30 fields was performed using the Kruskal–Wallis test followed by post hoc pairwise comparisons. The Kruskal–Wallis test statistic = 16.74; *p* = 0.0008. Multiple comparisons adjusted *p*-values for RRMS vs. AD = 0.0188; RRMS vs. NNC = 0.0088. Gray dots in the non-neurological control sample represent septic shock and pneumonia samples. H&E counterstain with TMEM106B immunostaining showed that TMEM106B immunoreactivity is detected within the cell body cytoplasm and processes ((**D**), arrow). Visualization was performed by diaminobenzidine (DAB). ns = not significant. (**L**) Statistical analysis of TMEM106B+ puncta/×63 fields, was performed using the Kruskal–Wallis test followed by post hoc pairwise Dunn’s multiple comparisons test. The Kruskal–Wallis test statistics; *p* = 0.0063. Multiple comparisons adjusted *p*-values for RRMS vs. AD = 0.0037; RRMS vs. NNC = 0.069; RRMS vs. SPMS, SPMS vs. NNCs *p* > 0.05.

TMEM106B immunoreactivity was examined using paraffin-embedded brain sections containing MS plaques, and adjacent white matter from individuals with RRMS. Consistent with the presence of the TMEM106B protein in sarcosyl-insoluble pellets, we observed numerous TMEM106B+ puncta in RRMS plaques (Figure 2A–D) and in the white matter adjacent to the RRMS plaque (Figure 2E). H&E counterstain with TMEM106B immunostaining showed that TMEM106B immunoreactivity is detected within the cell body cytoplasm and processes (Figure 2C,D, arrowhead, and arrow). Plaques from an individual with secondary progressive multiple sclerosis (SPMS) also showed TMEM106B+ puncta (Figure 2F and Supplemental Table S1). When compared to white matter from individuals with Alzheimer’s disease (AD) (Figure 2G; *n* = 6), and non-neurologic controls (Figure 2H,I; *n* = 10), the number of cells with TMEM106B+ puncta were increased/×63 fields in RRMS plaques (8.65 ± 3.64; median ± SEM; *n* = 6), compared to AD white matter cases showing fewer than 3 cells/×63 white matter field (1.35 ± 0.326; median ± SEM; *n* = 6), in 30 random fields/individual examined (Figure 2K). Examination of subcortical white matter from individuals who had no neurologic involvement at the time of death showed lower numbers of TMEM106B+ cells/×63 microscopic fields (1.94 ± 0.814; median ± SEM; *n* = 10). The TMEM106B+ puncta/cell from individuals who had no neurologic involvement was similar to the number of TMEM106B+ puncta/cell observed for AD white matter (Figure 2L). The number of puncta/cell was increased in the MS plaques with ~4–8 puncta/cell (5.0 ± 0.68) compared to AD white matter cells containing ~2–3 puncta/cell.

While the mass spectrometry data show that TMEM106B is enriched in insoluble pellets in RRMS, and immunostaining shows increased numbers of TMEM106B+ cells/field, post-mortem MS material does not allow for the type of analysis that mouse models permit. Therefore, we examined TMEM106B^t/t^ mice in two models of nervous system injury, MOG-induced EAE and the cuprizone model. Our prediction was that a reduction in TMEM106B levels would result in more axonal damage in TMEM106B^t/t^ mice during chronic EAE, with OilRedO+ deposition indicating that myelin debris is not efficiently cleared.

### 3.3. Naïve WT and TMEM106B^t/t^ Mice Exhibit Similar Myelination and CNS Architecture at the Corpus Callosum and Spinal Cord

Prior to performing studies, we analyzed the brains and spinal cords of naïve C57B6J and TMEM106B^t/t^ mice for any deficits in 8–10-week young adult mice and in 5.5-month-old adults. We determined that TMEM106B^t/t^ mice have normal cellular architecture and normal-appearing gray matter with equivalent motor neurons in the spinal cord. A myelin stain (Brain Stain Imaging kit (B34650)) of the corpus callosum and the spinal cord is shown in Supplemental Figure S1. Our data are consistent with the data of Zhou et al. who found that myelin protein levels were not significantly reduced in naïve TMEM106B^−/−^ or TMEM106B^+/−^ corpus callosum or sciatic nerve relative to wild-type mice [25]. Immunoblot analysis of an isolated naïve corpus callosum incubated with an MBP antibody (SMI99) showed no significant difference in abundance (Supplemental Figure S1G,H). While we did not study the fine structure of myelin fibers in the nervous system, naïve TMEM106B^−/−^ mice did not show differences in myelin thickness [25]. On outward inspection, behavioral analyses of the TMEM106B^t/t^ mice suggested no gait abnormalities or weakness on a hanging test.

### 3.4. TMEM106B^t/t^ Mice Have More OilRedO+ Puncta, Axonal Damage, and Demyelination during Chronic EAE

**Figure 3:**
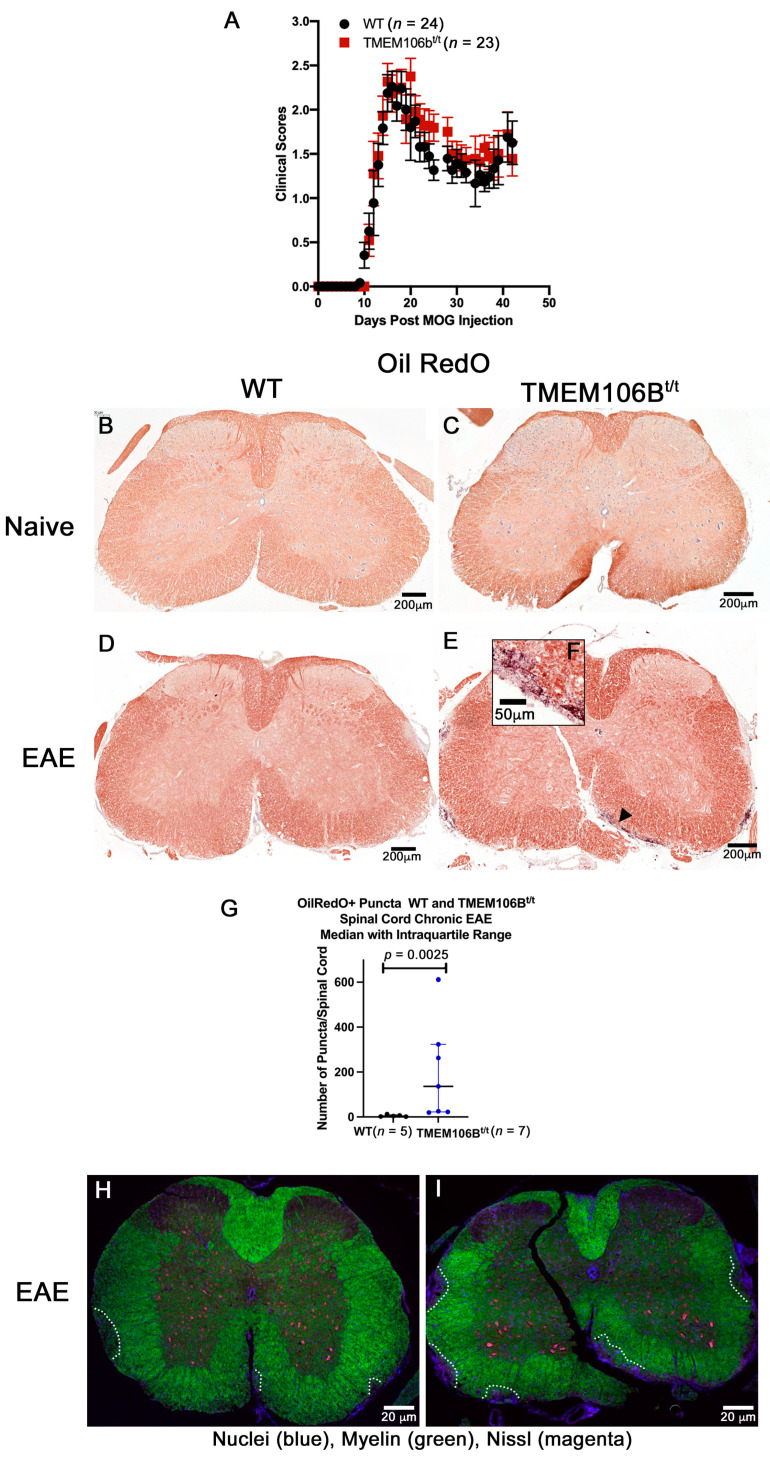
TMEM106B^t/t^ spinal cords show increased OilRedO+ lipid deposition and greater loss of myelin than control mice with similar clinical scores during chronic EAE. (**A**) EAE Curve: No differences in the clinical course between the two groups of mice were observed in male and female mice. OilRedO staining of naïve female WT (**B**) and TMEM106B^t/t^ (**C**) spinal cords shows no OilRedO puncta (*n* = 3/group). OilRedO staining of WT (**D**) and TMEM106B^t/t^ (**E**,**F**) spinal cords at ~42 days post-MOG injection. No enlarged region of the WT spinal cord is shown at day 42 since there was no clustered OilRedO+ puncta staining in the parenchyma. (**G**) Total OilRedO+ puncta per spinal cord of WT (*n* = 5) and TMEM106B^t/t^ (*n* = 7) mice. Mann–Whitney U test. Images from chronic EAE are shown in (**D**–**F**). A myelin stain (Brain Stain Imaging kit B34650; Molecular Probes) shows reduced myelin in a TMEM106B^t/t^ spinal cord ((**I**), green) relative to control (**H**), outlined in white. Nuclei are stained blue (DAPI), neurons are stained magenta (Nissl), and lipids (green).

**Figure 4:**
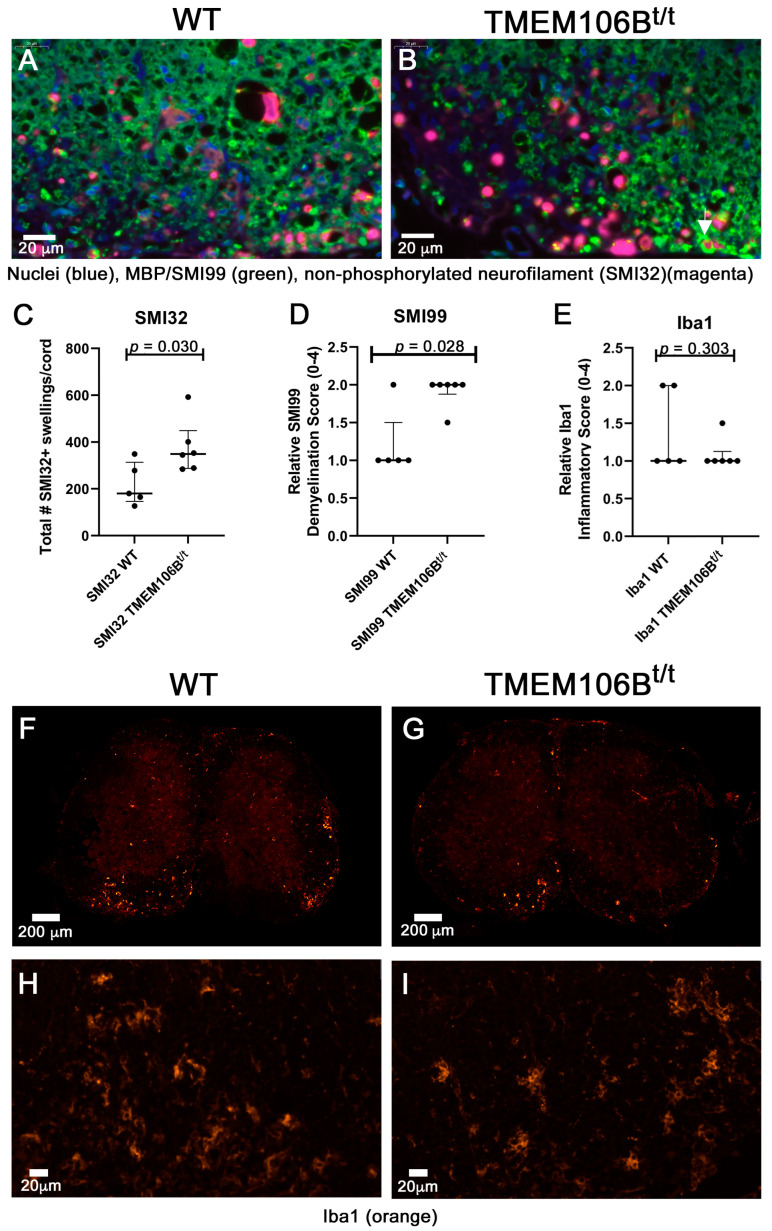
TMEM106B^t/t^ spinal cords show more axonal damage and demyelination relative to control mice during chronic EAE. (**A**) Female WT (*n* = 5) and (**B**) TMEM106B^t/t^ (*n* = 6) spinal cord sections were stained with DAPI (blue), MBP (green), and SMI32 (magenta). Shown is the ventral region of the lumbar spinal cord during chronic EAE. (**C**) SMI32^+^ swellings/cord were counted and quantified. Mann–Whitney U test, 3; *p* = 0.0303. (**D**) Relative SMI99 (MBP) was scored on a 0–4 scale (see Section 2.12). Mann–Whitney U test, 3.5; *p* = 0.0281. (**E**) Iba1inflammatory score showed no differences between the two groups during chronic EAE. Statistical analysis was performed using GraphPadPrism Software. Mann–Whitney U test. Iba1 Mann–Whitney U test, 10.5; *p* = 0.303. Representative Iba1 immunofluorescent staining (orange) of WT (**F**,**H**) and TMEM106B^t/t^ spinal cords (**G**,**I**).

**Figure 5:**
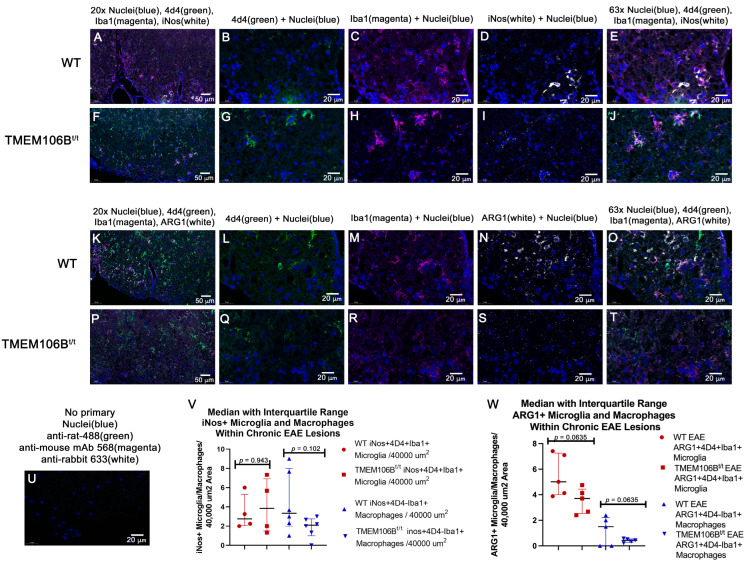
No difference in the number of pro-inflammatory (iNOS+)- and anti-inflammatory (ARG1+) microglia and macrophages in the ventral chronic spinal cords of WT and TMEM106B^t/t^ mice. Monoclonal antibody 4D4 was used to identify resident microglia. Iba1 identified microglia and macrophages. WT (**A**–**E**) and TMEM106B^t/t^ (**F**–**J**) spinal cords were immunostained with DAPI (blue), 4D4 (green), Iba1 (Magenta), and iNOS (white). The region of the ventral spinal cord examined is shown at ×20 in (**A**,**F**). WT (**B**–**E**) and TMEM106B^t/t^ (**G**–**J**) are shown and were quantified at ×63 (**V**). No primary control is shown (**U**). WT (**K**–**O**) and TMEM106B^t/t^ (**P**–**T**) spinal cords were immunostained with DAPI (blue), 4D4 (green), Iba1 (magenta), and ARG1 (white). The region of the ventral spinal cord examined is shown at ×20 in (**K**,**P**). WT (**L**–**O**) and TMEM106B^t/t^ (**Q**–**T**) are shown and were quantified at ×63 (**W**).

MOG-induced EAE is a pertinent model to validate and characterize proteins in the context of inflammatory disease. To examine the consequences of reduced TMEM106B during EAE, TMEM106B^t/t^ and WT mice were sensitized with MOG_35-55_ peptide and monitored over the course of EAE. There was no difference in the day of onset of clinical scores or indices (CI) between the WT (12.6 ± 0.75 days) and the TMEM106B^t/t^ mice (11.0 ± 1.2 days) when individual groups of male and female mice (*n* = ~10/group) were challenged. No significant differences in the clinical indices of WT and TMEM106B^t/t^ were observed. The same clinical course was observed for both groups of mice during acute and chronic disease (Figure 3A). Mice were sacrificed at approximately day 42 post-MOG injection; the mean clinical score per group was 1.17 ± 0.17 (WT) and 1.63 ± 0.47 (TMEM106B^t/t^). When lumbar spinal cord sections were examined histologically, there was no significant difference in the overall number of lesions assessed by H&E, the extent of gliosis as assessed for GFAP+ astrocytes, or Iba1+ activated microglia (Figure 4 and Figure 5).

The coronal sections of spinal cords were examined for OilRedO+ deposition of neutral lipid droplets, changes in myelin (Brain Stain Imaging kit B34650; Molecular Probes), myelin basic protein (MBP, SMI99) immunoreactivity, and axonal swelling by assessing SMI32+ in axons. To assess the extent of efficient clearance of neutral lipid debris in the two groups of mice, we stained frozen spinal cord sections with OilRedO. Naïve WT and TMEM106B^t/t^ mice have no OilRedO+ puncta deposition in the spinal cord at 5.5 months of age (Figure 3B,C); all MOG-sensitized mice were less than 4.5 months of age at the time of sacrifice. While there was no OilRedO+ puncta deposition in all the WT spinal cords examined during chronic EAE (Figure 3D), there were OilRedO+ lesions remaining in TMEM106B^t/t^ spinal cords both in the white and gray matter (Figure 3E,F). The ventral region of the spinal cord with OilRedO+ puncta staining in Figure 3E (arrowhead) is magnified in Figure 3F. An additional TMEM106B^t/t^ mouse spinal cord with OilRedO+ puncta in a lateral lesion following chronic EAE is shown in Supplemental Figure S2. Total OilRedO+ puncta were counted in multiple spinal cord sections. The total number of puncta/spinal cord was higher in TMEM106B^t/t^ (*n* = 7) than in WT (*n* = 5) mice (Figure 3G). The mice were matched for similar clinical courses throughout EAE and the final chronic clinical score. Myelin (green), Nissl (magenta), and DAPI (blue) stains showed the extent of demyelinated regions in the frozen cross-sections of WT and TMEM106B^t/t^ spinal cords with the same clinical score CI = 2; myelin loss is outlined in white (Figure 3G,H).

To quantify changes in myelin and axonal damage, we stained paraffin-embedded spinal cord sections with an antibody to myelin basic protein (MBP; SMI99) and assessed axonal damage using an antibody to non-phosphorylated neurofilament protein (SMI32). There was a significant difference in MBP staining (green) in the spinal cords of TMEM106B^t/t^ mice when myelin loss was scored on a 1–4 scale (Figure 4). The demyelinated ventral region of TMEM106B^t/t^ lumbar spinal cord shows an increased number of SMI32^+^ swellings (magenta) in the lumbar spinal cord (Figure 4A,B). The SMI32^+^ swellings are observed on both naked axons devoid of myelin, and axons still ensheathed by myelin (white arrow), indicative of ongoing axonal injury. The total number of SMI32^+^ axonal swellings within the entire cross-section of the lumbar spinal cord was significantly higher in TMEM106B^t/t^ mice relative to WT (Figure 4C; *p* = 0.030). The relative degree of demyelination as assessed by SMI99^+^ staining in the TMEM106B^t/t^ mice relative to WT mice was also significant (*p* = 0.028; Figure 4D). No significant differences in Iba1+ glia were observed between WT and TMEM106B^t/t^ mice using a relative Iba1 inflammatory score (0–4; Figure 4E–G; Mann–Whitney U test, 10.50; *p* = 0.303), or in the mean fluorescent intensity/area measured for each lumbar spinal cord region; Kruskal–Wallis statistic, 8.467; *p* = 0.1323. When viewed at higher magnification, the morphology of the Iba1+ glia is predominantly reactive and ameboid (Figure 4H,I).

We delineated resident microglia from infiltrating myeloid cells using the antibody 4D4 which stains only resident microglia [37,38,39,40], with Iba1 as the microglia/macrophage marker. Moreover, we examined iNOS and ARG1 expression in chronic EAE lesions (Figure 5A–J,K–T) and observed no differences (V,W). In WT spinal cords, the majority of ARG1+-expressing cells were Iba1+ with occasional Iba1+4D4+ARG1+ microglia. There did not appear to be a difference in the numbers of ARG1+ macrophages between WT and TMEM106B^t/t^ spinal cords; rather we observed more ARG1+ cells at the meninges in TMEM106B^t/t^ spinal cords.

### 3.5. WT and TMEM106B^t/t^ Mice Have Equivalent OilRedO+ Staining during Late Acute EAE

**Figure 6:**
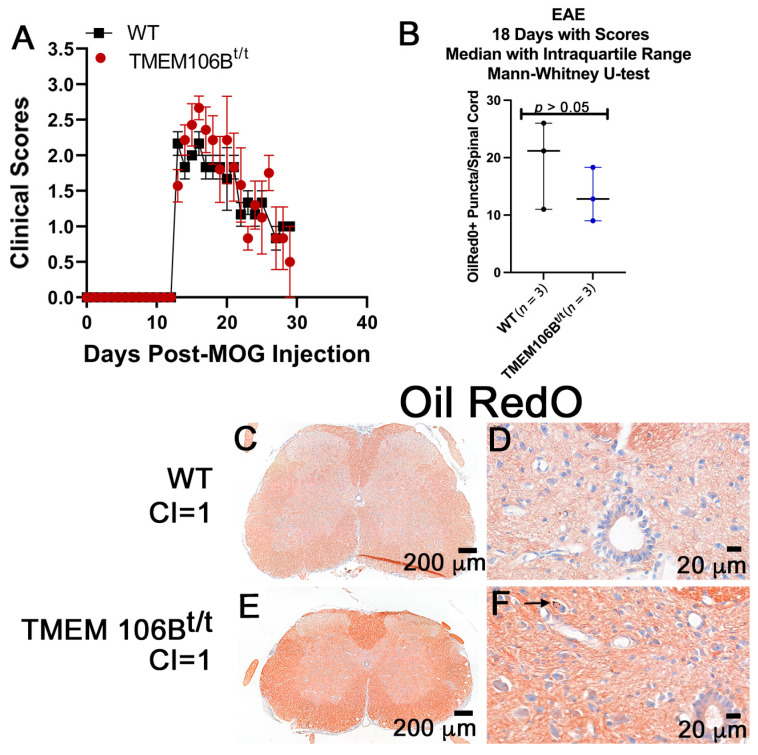
TMEM106B^t/t^ mice sensitized with MOG_35-55_ peptide have OilRedO+ lipid deposits at the central canal and in the spinal cord after consecutive clinical scores of CI > 1 for 18 days. (**A**) No significant difference in the clinical scores of female WT (black) and TMEM106B^t/t^ (red) mice. (**B**) Total OilRedO+ puncta/spinal cord of mice with CI = 1, *n* = 3. Mann–Whitney U test. OilRedO+ staining of frozen spinal cord sections of WT mice CI = 1 (*n* = 3; (**C**,**D**)), and TMEM106B^t/t^ mice CI = 1 (*n* = 3; (**E**,**F**)).

We sensitized a small group of WT and TMEM106B^t/t^ mice with MOG peptide and examined the lipid droplet accumulation in the spinal cords of mice. Mice with scores >1 for 18 consecutive days were sacrificed on the 18th day (Figure 6A, late acute EAE). Again, there was no significant difference in the clinical scores of the two groups of mice. Frozen spinal cord sections from WT mice all with a final CI = 1 (*n* = 3) and TMEM106B^t/t^ (*n* = 5; CI = 1) mice were incubated with OilRedO. Mice with CI = 1 were paired based on the number of days with the same final clinical score. When compared to the OilRedO+ spinal cords during chronic EAE, there was minimal OilRedO+ staining in the three WT spinal cords (Figure 6B–D). TMEM106B^t/t^ spinal cords with CI = 1 (*n* = 3) and a similar course to the WT (*n* = 3) showed OilRedO+ puncta in neurons and within the parenchyma (Figure 6F, arrow) but the total OilRedO+ puncta/spinal cord was not significantly different from that of WT spinal cords.

### 3.6. TMEM106B^t/t^ Mice Have OilRedO+ Deposits in the Corpus Callosum at 2 and 3 Weeks Post-Cuprizone Withdrawal

**Figure 7:**
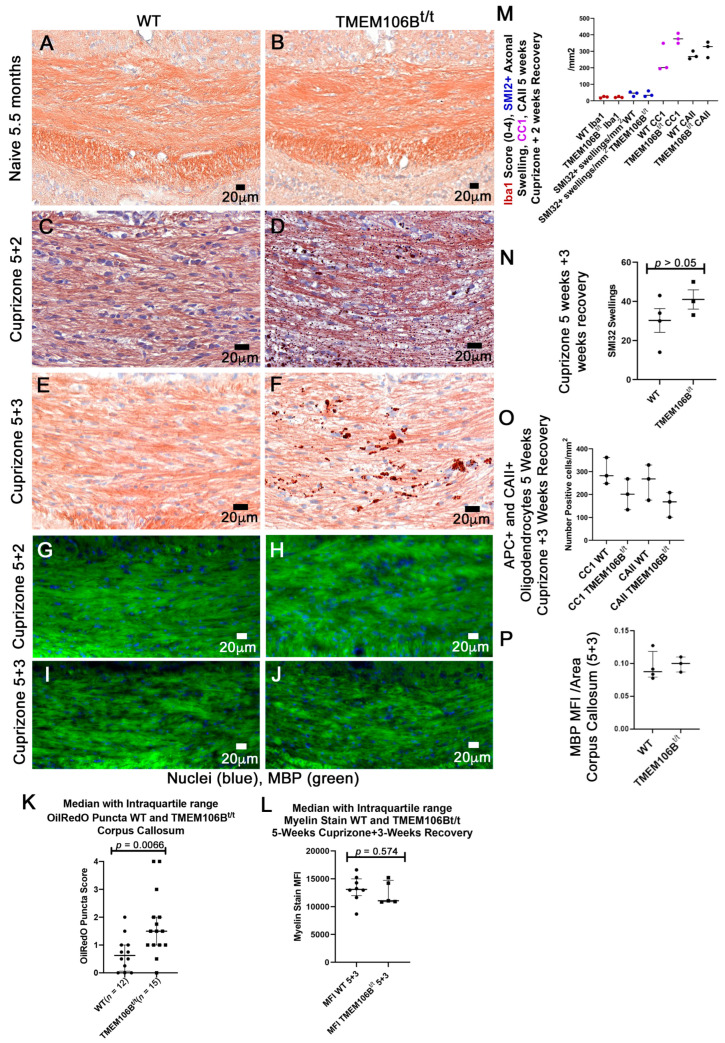
TMEM106B^t/t^ mice have significant OilRedO+ deposition in the corpus callosum at 2 and 3 weeks post-cuprizone withdrawal. (**A**–**F**) OilRedO stain. (**A**,**B**) illustrates the lack of OilRedO+ staining in the corpora callosa of control, naïve WT, and TMEM106B^t/t^ mice at 5.5 months of age. (**C**–**J**) Ten-week old mice were given a five-week cuprizone diet, followed by regular chow for two weeks (5 + 2; C,D,G,H) and three weeks (5 + 3; **E**,**F**,**I**,**J**) prior to sacrifice. OilRedO+ puncta scores from WT (*n* = 12) and TMEM106B^t/t^ (*n* = 15) corpora callosa at 5 weeks cuprizone + 3 weeks recovery (**K**). Median with interquartile range, *p* = 0.0066, two-tailed Mann–Whitney U test. (**G**–**J**) A myelin stain (Brain Stain Imaging kit B34650; Molecular Probes) shows no significant change in remyelination at 2 weeks (**G**,**H**) and 3 weeks (**I**,**J**) post-cuprizone withdrawal. MFI myelin stain for WT (*n* = 8) and TMEM106B^t/t^ (*n* = 5) at 5 weeks cuprizone + 3 weeks recovery (**L**). Median with interquartile range, Mann–Whitney U test. (**M**) Mann–Whitney U test determined no difference in Iba1 (4.5, >0.9999), SMI32 (4.5, *p* => 0.9999), APC/CC1 (0.5000, *p* = 0.2000), and CAII (2, *p* = 0.4000) immunopositive cells/mm^2^ at 2 weeks post-cuprizone withdrawal (*n* = 3/group). Mann–Whitney U test showed no difference in SMI32 (**N**), APC/CC1, and CAII immunopositive cells/mm^2^ (**O**) or MBP (**P**) mean fluorescent intensity (4, *p* = 0.6286) at 3 weeks post-cuprizone withdrawal. WT (*n* = 4), TMEM106B^t/t^ (*n* = 3).

WT and TMEM106B^t/t^ 10-week-old male and female mice were fed 0.2% cuprizone in powdered chow for 5 weeks, and groups of mice were sacrificed 2 and 3 weeks post-cuprizone withdrawal. Based on our analyses during EAE, we predicted that the corpora callosa of TMEM106B^t/t^ mice would retain OilRedO+ deposition throughout recovery. TMEM106B^t/t^ mice had significantly more and larger OilRedO+ inclusions at the midline and lateral regions of the corpus callosum at 2 weeks post-cuprizone withdrawal when there was minimal deposition in the WT corpus callosum (Figure 7C,D). When analyzed at the midline of WT and TMEM106B^t/t^ corpora callosa (*n* = 3/group), less than 10% of the area of WT corpora callosa contained OilRedO+ puncta. All images were divided into four quadrants and counts obtained at ×40 in CaseViewer. Small puncta with a diameter of <3 µm were observed in one to two quadrants of the WT corpora callosa; the lateral regions of the corpus callosum showed few OilRedO+ puncta of <2 µm. OilRedO+ puncta were detected throughout all four quadrants of the midline of the callosa of TMEM106B^t/t^ mice, and the sizes of the puncta were larger. OilRedO+ puncta ranging from 3.8 to 8.9 µm were detected in all quadrants. After 3 weeks of recovery, there were still OilRedO+ inclusions at the midline of TMEM106B^t/t^ callosa and the lateral regions. The WT mice had no visible OilRedO+ puncta at the midline and only one of the three had small puncta in the lateral regions (Figure 7E,F). The callosa of control 5.5-month naïve WT and TMEM106B^t/t^ mice appeared normal with no OilRedO+ deposits (Figure 7A,B). In two additional experiments, we examined OilRedO+ puncta in the callosa of mice at 3 weeks of recovery. In these experiments, the OilRedO+ puncta were more in the range of 2 µm. To analyze the data across all samples from the three experiments and 3-week recovery, we used an OilRedO puncta score of 0–4 (see Section 2) and analyzed the data from WT (*n* = 12) and TMEM106B^t/t^ (*n* = 15) mice, which showed more OilRedO+ puncta in TMEM106B^t/t^ than WT (*p* = 0.0066, two-tailed Mann–Whitney U test) (Figure 7K).

Immunostaining for myelin basic protein showed no significant difference in WT and TMEM106B^t/t^ corpora callosa at 2 and 3 weeks of recovery (Figure 7G–J,N). The myelin stain (Brain Stain Imaging kit) showed no significant difference in myelin in WT and TMEM106B^t/t^ corpora callosa at 3 weeks of recovery (Figure 7L). Examination of oligodendrocyte protein markers showed no significant difference in oligodendrocyte number as assessed by APC/CC1 immunofluorescent staining (Figure 7M,O). Carbonic anhydrase (CAII), another oligodendrocyte marker for oligodendrocytes that participates in the myelination of small caliber axons [41] showed no difference in oligodendrocyte number between the two groups (Figure 7M,O). SMI32^+^ staining of axons at both 2 and 3 weeks post-cuprizone withdrawal showed no significant differences in the number of axonal swellings (Figure 7M,N). The Iba1+ score was no different at 2 weeks of recovery (Figure 7M), and there was no difference in the extent of gliosis. It is likely that the presence of OilRedO+ droplets observed in the TMEM106B^t/t^ CNS following cuprizone treatment and during chronic EAE reflect a delay in the delivery of neutral lipids to the lysosome for degradation via lipophagy, a selective form of autophagy that targets lipid droplets.

## 4. Discussion

We identified the enrichment of TMEM106B in β-mercaptoethanol/sarkosyl-insoluble pellets from white matter plaques isolated from individuals with RRMS by mass spectrometry, and observed increased TMEM106B immunoreactive puncta in human white matter from individuals with RRMS relative to Alzheimer’s disease and non-neurologic controls. TMEM106B protein levels are known to affect lysosomal function [14,30]. Low TMEM106B levels result in the clustering of small lysosomes near the nucleus; and in neurons, altered retrograde lysosomal mobility and shortened dendritic branching have been reported [22]. High levels of TMEM106B result in larger and fewer lysosomes that fail to produce normal intra-lysosomal acidity, resulting in lysosomal stress [42]. The presence of TMEM106B in sarcosyl-insoluble pellets from the plaques of individuals with MS suggests that sequestration of TMEM106B within insoluble pellets may result in lysosomal dysfunction/stress perhaps with delayed and inefficient clearance of debris within the plaque. OilRedO+ neutral lipid droplets stain the myelin debris within and adjacent to MS plaques [5,7,43,44]. We observed OilRedO+ deposits in frozen sections of MS plaques from individuals in which we performed nanoLC-ms/ms, although we did not observe TMEM106B+/OilRedO+ cells within all frozen MS brain sections examined.

Since post-mortem material is static and does not allow for the type of analysis mouse models permit, we examined hypomorphic TMEM106B^t/t^ mice that express low levels of TMEM106B, and levels of myelin proteins equivalent to wild-type mice [23]. The TMEM106B^t/t^ mice allowed us to examine the impact of impaired TMEM106B in two models of nervous system injury, MOG-induced EAE and the cuprizone model [27,28]. We acknowledge that insoluble proteins that we identified in RRMS lesions are not found in EAE or the cuprizone model, yet these versatile mouse models allow for further characterization of the impact of reduced TMEM106B within a compromised nervous system. While our prediction was that the TMEM106^t/t^ mice would have higher clinical scores than WT mice during acute and chronic EAE, we observed the same clinical course in both groups. However, during chronic EAE, we observed increased OilRedO+ lipid deposits in TMEM106B^t/t^ spinal cords, suggesting that there is lipid droplet accumulation and likely a failure to efficiently clear debris following prolonged injury. Future studies will evaluate changes in lipid profiles in the spinal cords of WT and TMEM106B^t/t^ mice during chronic EAE by lipidomics.

We observed more axonal damage, reflected in an increase in SMI32^+^ axonal swellings and less intact myelin during chronic EAE. It is well established that myelination supports axonal integrity and demyelination disrupts nerve signals and contributes to axon degeneration. Although remyelination supports axonal integrity and neuronal function after inflammatory demyelination [45], remyelination does not occur in EAE [46]. Inflammatory EAE results in demyelination and axonal damage reflected in increased axonal swellings and spheroids that can disrupt intracellular trafficking and the synthesis and degradation of proteins that may be exacerbated by a reduction or sequestration of TMEM106B. Although we observed no difference in inflammation at the chronic time point, TMEM106B functions in clearance may impact the clearance of lipid droplets and damaged proteins. An interaction between TMEM106B and the microtubule-associated protein MAP6 may impact transport and intracellular trafficking as MAP6 binds to the cytoplasmic N-terminus of TMEM106B [22].

We delineated resident microglia from infiltrating myeloid cells using antibody 4D4 that stains only resident microglia [37,38,39,40] with other monocyte/macrophage and microglia markers, such as Iba1 and CD11b. CD11b is expressed on steady-state microglia, monocytes, macrophages, neutrophils, natural killer cells, and granulocytes. Similar to staining with Iba1, the majority of 4D4+/CD11b+ cells were ramified microglia. ARG1 is an anti-inflammatory marker for microglia/macrophages [47] expressed in infiltrating cells and resident microglia [48,49]. ARG1+ microglia are considered protective [47,50]. ARG1 catalyzes the conversion of arginine to ornithine and urea and competes with inducible nitric oxide synthase (iNOS) for the substrate of arginine. ARG1 downregulates the production of nitric oxide and is beneficial for tissue repair following pathologic conditions [47,51,52,53]. In addition to limiting inflammation in microglia and macrophages, ARG1 promotes efferocytosis and enhances phagocytosis in microglia [48,50]. In WT spinal cords during chronic EAE, the majority of ARG1+-expressing cells were Iba1+ with occasional Iba1+4D4+ARG1+ microglia. There did not appear to be a difference in ARG1+ macrophage between WT and TMEM106B^t/t^ spinal cords; rather, we observed more ARG1+ cells at the meninges in TMEM106B^t/t^ spinal cords. The data show that equivalent ARG1+ anti-inflammatory glia are present in WT and TMEM106B^t/t^ parenchyma, and during chronic EAE, there is no difference in the numbers of iNOS+ inflammatory macrophages and microglia.

As a type II transmembrane glycoprotein that localizes to lysosomes [18,54,55], there is limited data on TMEM106B’s normal function in vivo. Our study used the TMEM106B hypomorphic mouse model, while others have used complete TMEM106B knockout mice to explore the impact of reduced TMEM106B function in the CNS. Beneficial effects of reduced TMEM106B levels have been reported, especially in frontotemporal dementia-related phenotypes in progranulin-deficient mice where the loss of TMEM106B ameliorated abnormal lysosomal phenotypes [33]. However, TMEM106B has opposing effects in different mouse models of lysosomal diseases [23]. In facial motor neurons, proximal axonal swelling with enlarged LAMP1+ vacuoles and an accumulation of autophagolysosomes revealed that TMEM106B functions in axonal transport of LAMP1+ organelles and axonal sorting at the initial segment [15]. Our TMEM106B^t/t^ mouse model retains low levels of TMEM106B protein detected by Western blot analysis [21,23]. This reduced protein level is insufficient to maintain homeostasis within the CNS and efficiently clear lipid-laden debris in challenged mice.

Of the total TMEM106B knockout mouse models generated, loss of TMEM106B led to myelination deficits, such as delayed cerebellar myelination in young mouse brains and a loss of Purkinje cells in aged mice [16,17,21,24,25,33,56,57]. Similar to our cuprizone study, the total loss of TMEM106B did not show a significant difference in the number of oligodendrocytes within the corpus callosum during recovery but did show changes in remyelination [25]. The study did not report persistent lipid debris with increased OilRedO+ deposition during 2 and 3 weeks of recovery from cuprizone-induced demyelination. Combined studies suggest that during recovery from cuprizone-induced toxicity, mature oligodendrocytes in the corpora callosa of TMEM106B^t/t^ mice can remyelinate, but a complete loss of TMEM106B function affects remyelination. Future studies will determine whether prolonged cuprizone treatment (12 weeks) or a second 5-week cuprizone treatment course after the initial treatment and recovery results in deficits in remyelination in TMEM106B^t/t^-treated relative to control mice. In addition, the cuprizone model in combination with EAE can be performed where increased brain inflammation was reported using this model [58].

Our combined data demonstrate that in mice, reduction in TMEM106B in the spinal cord during EAE has a more profound effect than in the corpus callosum. The presence of OilRedO+ neutral lipids within the CNS suggests that TMEM106B has a functional role in the clearance of lipids. Lipophagy functions to regulate intracellular lipid stores, cellular levels of free lipids such as fatty acids, and energy homeostasis [59]. Structural similarities between human TMEM106B and two yeast proteins Vac7 and Tag1 predict that all three proteins have LEA-2 domains and are lipid transfer proteins [60]. The C-termini of TMEM106B and LEA-2 are highly homologous. Vac7, a regulator of PI(3,5)P2 stress responses and production, has one LEA-2 domain. Tag1, which signals to terminate autophagy, has three LEA-2 domains, and TMEM106B has one LEA-2 domain. The authors speculate that these proteins may sense a signal derived from autophagic material build-up, possibly lipids, and provide feedback to regulate autophagy. TMEM106B has the potential to regulate lysosomal function, and when TMEM106B levels are reduced there is retention of OilRedO+ lipids following CNS injury that can contribute to CNS impairment, as observed during EAE, and recovery from cuprizone-induced demyelination. The notion that TMEM106B may sense a signal derived from lipids and communicate signals to regulate autophagy is intriguing and warrants further examination.

**Figure 8:**
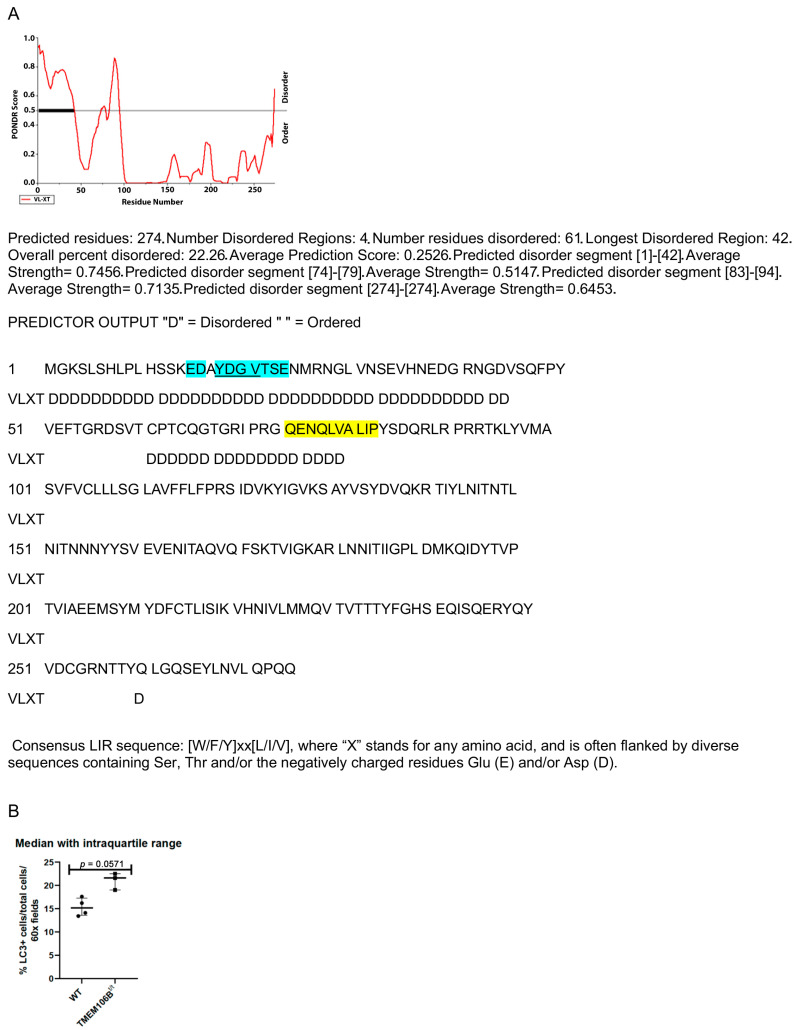
(**A**) Regions of disorder and a possible LIR motif [W/F/Y]-X1-X2-[L/I/V] for potential lysosomal localization within the TMEM106B protein (pndr.com). (**B**) Percentage LC3+ cells/total cells per ×60 fields in WT (*n* = 4) and TMEM106B^t/t^ (*n* = 3) corpora callosa 3 weeks after cuprizone withdrawal.

Selective autophagy is important for sequestering protein cargo into autophagosomes and targeting autophagy receptors to LC3 (microtubule-associated proteins 1A/1B light chain 3B) for delivery to the lysosome. Several proteins containing LC3 interacting region (LIR) motifs have been identified [61]. We examined the proteins in our RRMS insoluble pellet and identified several unique LIR peptides within the aggregate including TMEM106B. TMEM106B contains a possible LC3 LIR motif [Y/F/W]X1-X2[L/I/V] at amino acid 18–21, in a naturally disordered region EDAYDGVTSE (PONDR.org, Figure 8A). The LIR is often flanked by diverse sequences containing Ser, Thr, and/or the negatively charged residues Glu (E) and/or Asp (D). We performed a preliminary study with a small number of WT and TMEM106B^t/t^ brains and measured the number of cells at the midline of the corpus callosum with LC3+ puncta as a ratio of the total number of cells in the corpus callosum per ×60 field at 3 weeks post-cuprizone withdrawal. Although we observed a 1.4-fold increase in the percentage of cells containing LC3+ puncta in TMEM106B^t/t^ corpora callosa (*n* = 3) relative to WT mice (*n* = 4), significance was not obtained (*p* = 0.0571, Mann–Whitney U test) (Figure 8B). We found that very low LC3-II levels were detected in WT and TMEM106B^t/t^ isolated corpora callosa at 3 weeks of recovery by Western blot analysis. Additional studies are required to determine whether there is delayed lipophagy in the TMEM106B^t/t^ CNS following demyelination and inflammatory insults. The accumulation of lipids without efficient clearance by lipophagy could result in a more severe disease course in multiple sclerosis since it was found that stimulating autophagy with trehalose reduced the lipid load within cultures of myelin-laden macrophages [62]. In future studies, trehalose can be tested in vivo to determine if it can reduce OilRedO+ debris in WT and TMEM106B^t/t^ mice during EAE, where the blood–brain barrier is disrupted. Therefore, it is important to pursue further studies, especially since lipid droplets can co-localize with LC3 [63].

Several proteins have been suggested to bind within TMEM106B’s disordered regions [64] and future studies will test whether the in vitro mutagenesis of the motifs limits the efficient clearance of the insoluble proteins and lipids. Alterations in TMEM106B function have been linked to neurodegenerative disease [65,66,67,68,69,70,71], and defects in dendritic trafficking of lysosomes and axons [15,22,72,73,74]. The presence of insoluble TMEM106B suggests that a proportion of TMEM106B in MS plaques is unavailable to traffic to the lysosome to carry out its function in clearance. Since dysfunction of TMEM106B is known to impair the transport and function of lysosomes [75], loss of functional TMEM106B could impact recovery from demyelinating lesions in RRMS.

## Data Availability

The mass spectrometry raw data were uploaded to the repository Chorus (www.chorusproject.org) under project number 1814.

## Supplementary Materials

The following supporting information can be downloaded at: https://www.mdpi.com/article/10.3390/cells12131734/s1, Figure S1: Naïve WT and TMEM106B^t/t^ mice have similar myelin deposition in brain and spinal cord.; Figure S2: Lateral lesion in spinal cord parenchyma shows increased OilRedO+ lipid deposition during chronic EAE relative to WT spinal cords.; Figure S3: Staining of WT and TMEM106B^t/t^ corpus callosum at 5-weeks cuprizone and 2-weeks recovery (A–H) and 3-weeks recovery (I–P).; Table S1: Available samples for the ongoing study. Table S2: Proteomics analysis of RRMS plaques and controls via liquid chromatography coupled online to mass spectrometry.

## Abbreviations

APC, adenomatous polyposis coli; CAII, carbonic anhydrase II; CI, clinical index; EAE, experimental autoimmune encephalomyelitis; GFAP, glial fibrillary acid protein; Iba1, Ionized calcium-binding adaptor molecule-1; RRMS, relapsing–remitting multiple sclerosis; SPMS, secondary progressive multiple sclerosis; MOG, myelin-oligodendrocyte glycoprotein; MBP, myelin basic protein; MS, multiple sclerosis; TMEM106B, transmembrane protein106B; WT, wild-type; CD11b, integrin alpha M and macrophage-1 antigen.
