# Supplementary material for: TMEM106B Puncta Is Increased in Multiple Sclerosis Plaques, and Reduced Protein in Mice Results in Delayed Lipid Clearance Following CNS Injury
Source: Cells. 2023 Jun 27;12(13):1734. doi: 10.3390/cells12131734 (PMC10340176; doi:10.3390/cells12131734)
Supplement: Supplementary file 1 [file cells-12-01734-s001.zip › Supplemental TABLE S1.pdf]

Supplemental TABLE 1: Available samples for the ongoing study. **For nanoLC-ms/ms**:

<u>RRMS</u>

Case #	PMI*	Age	Gender
1	5h	65	Diagnosis
2	5h	65	Female RRMS
3	24h	61	Female RRMS
4	2h	69	Male RRMS
5	7h	64	Female RRMS

*Post-mortem interval

<u>Non-neurological White Matter Controls</u>

Case #	PMI	Age Gender	Diagnosis
1	5h	74	Male Cardiac
2	5h	57	Female Breast Cancer
3	19h	59	Male Cardiac
4	9h	63	Male Infarct/Cardiac
5	6.5h	65	Male Cardiac

<u>For IF and IHC Staining:</u>

<u>MS Cases Paraffin-</u>

Case	PMI	Age	Gender Diagnosis
11	15h	49	Female Secondary Progressive, Plaque and
12	<10h	61	NAWM Female RRMS, Plaque and NAWM
13	10h	61	Mal RRMS, Plaque and NAWM
14	UNK	68	e MS type unknown, Plaque and
15	14h	73	Mal NAWM RRMS, Plaque and
16	21h	65	e Female NAWM RRMS, Plaque and
17	14h	64	Mal NAWM Male RRMS, Plaque
18	12h	69	e and NAWM Male RRMS,
			Plaque and NAWM

19	12h	57	Male	RRMS, Plaque and NAWM
20	18h	68	Male	RRMS, Plaque and NAWM

Other Neurologic Controls and Non-Neurologic Controls

Alzheimer's Disease Paraffin-embedded

Case #	PMI	Age	Gender	Diagnosis
21	UNK*	86	Female	AD
22	UNK	66	Female	AD
23	UNK	62	Male	AD
24	12 h	70	Male	AD
25	UNK	63	Male	AD
26	UNK	80	Female	AD
27	UNK	61	Male	AD

*Unknown

Non-Neurological Controls (NNCs) Paraffin-embedded

Case #	PMI	Age	Gender	Diagnosis
28	2 d	72	Male	Pneumonia
29	22h	65	Female	Septic shock
30	UNK	78	Male	No diagnostic abnormality recognized
31	UNK	67	Male	No diagnostic abnormality recognized
32	UNK	62	Male	No diagnostic abnormality recognized
33	UNK	81	Male	Primary Age-related tauopathy
34	UNK	26	Male	Severe and chronic lung injury, respiratory distress syndrome, liver cirrhosis
35	UNK	59	Male	Advanced K-ras-mutated non-small cell adenocarcinoma stage IV
36	UNK	48	Female	Metastatic carcinoma probable urachal origin and hypertension
37	UNK	68	Female	Toxic epidermal necrolysis 60% body
