# Supplementary material for: TMEM106B Puncta Is Increased in Multiple Sclerosis Plaques, and Reduced Protein in Mice Results in Delayed Lipid Clearance Following CNS Injury
Source: Cells. 2023 Jun 27;12(13):1734. doi: 10.3390/cells12131734 (PMC10340176; doi:10.3390/cells12131734)
Supplement: Supplementary file 1 [file cells-12-01734-s001.zip › Supplementary Table S2.pdf]

| Accession | Description | Gene Symbol | Volcano | RRMS/Ctrl | p-value | Z-score | Control-1 | Control-2 | Control-3 | Control-4 | Control-5 | RRMS-1 | RRMS-2 | RRMS-3 | RRMS-4 | RRMS-5 |
|---|---|---|---|---|---|---|---|---|---|---|---|---|---|---|---|---|
| P24310 | Cytochrome c oxidase subunit 7A1, mitochondrial | COX7A1 | | 5.25 | 2.39 | | -0.40 | -0.46 | -0.53 | -0.46 | -0.46 | 1.93 | 1.86 | -0.54 | -0.48 | -0.47 |
| Q9NUM4 | Transmembrane protein 106B | TMEM106B | | 3.67 | 6.20 | | 0.71 | -1.09 | -1.36 | -1.27 | -0.69 | 0.54 | 1.24 | 0.49 | 1.09 | 0.34 |
| P00491 | Purine nucleoside phosphorylase | PNP | | 3.42 | 6.30 | | -0.14 | -0.20 | -1.18 | -0.99 | -1.12 | 0.26 | 1.37 | 1.77 | -0.21 | 0.45 |
| P46926 | Glucosamine-6-phosphate isomerase 1 | GNPDA1 | | 3.17 | 4.61 | | -0.76 | -0.64 | -0.76 | -0.41 | -0.84 | 1.85 | 1.29 | 1.08 | -0.37 | -0.44 |
| P09455 | Retinol-binding protein 1 | RBP1 | | 3.17 | 3.57 | | 1.05 | -0.74 | -1.59 | -0.62 | -0.83 | 1.12 | 1.23 | 0.06 | -0.52 | 0.84 |
| P42330 | Aldo-keto reductase family 1 member C3 | AKR1C3 | | 3.17 | 3.50 | | -0.91 | -1.01 | -0.77 | 1.00 | -0.99 | 0.93 | -0.99 | 0.51 | 1.10 | 1.12 |
| Q9BS40 | Latexin | LXN | | 3.14 | 3.75 | | -0.96 | -0.11 | -1.77 | 0.05 | 0.00 | -0.48 | 0.47 | 2.05 | 0.28 | 0.47 |
| Q9UL68 | Myelin transcription factor 1-like protein | MYT1L | | 3.12 | 3.17 | | 0.57 | -0.78 | -0.86 | -0.78 | -0.75 | 0.32 | 0.43 | 0.30 | -0.74 | 2.29 |
| Q5VW36 | Focadhesin | FOCAD | | 3.11 | 1.98 | | 0.95 | -0.86 | -0.29 | -0.84 | -0.86 | 0.81 | 2.11 | -0.12 | -0.05 | -0.84 |
| Q16881 | Thioredoxin reductase 1, cytoplasmic | TXNRD1 | | 3.03 | 4.78 | | -1.60 | -0.12 | -0.06 | 0.30 | -1.92 | 0.97 | 0.91 | 0.38 | 0.39 | 0.74 |
| P15259 | Phosphoglycerate mutase 2 | PGAM2 | | 3.00 | 3.77 | | -0.83 | -0.91 | 0.10 | -0.38 | -0.92 | 1.21 | -0.84 | 1.70 | -0.30 | 1.17 |
| Q5JY77 | G-protein coupled receptor-associated sorting protein 1 | GPRASP1 | | 2.95 | 2.29 | | 1.57 | -1.24 | -0.85 | -0.82 | -0.77 | 0.89 | 1.30 | -0.47 | -0.15 | 0.55 |
| Q8NCW5 | NAD(P)H-hydrate epimerase | NAXE | | 2.94 | 3.54 | | 0.55 | -0.97 | -0.24 | -1.07 | -1.00 | 1.30 | 1.08 | 1.39 | -0.82 | -0.23 |
| Q92823 | Neuronal cell adhesion molecule | NRCAM | | 2.91 | 3.54 | | 0.41 | -0.65 | -2.03 | -0.14 | -0.30 | 1.50 | 1.02 | 0.82 | -0.34 | -0.30 |
| A0A0C4DH68 | Immunoglobulin kappa variable 2-24 | IGKV2-24 | | 2.91 | 4.17 | | 0.70 | -0.92 | -1.01 | -0.80 | -0.92 | 0.53 | 1.55 | 1.09 | -0.90 | 0.70 |
| P15153 | Ras-related C3 botulinum toxin substrate 2 | RAC2 | | 2.87 | 4.60 | | -0.60 | -0.61 | -0.87 | -0.67 | -0.68 | 1.60 | 1.35 | -0.92 | 0.24 | 1.15 |
| P14136 | Glial fibrillary acidic protein | GFAP | | 2.85 | 4.24 | | -0.10 | -1.23 | -0.59 | -0.49 | -0.73 | 1.81 | -0.06 | 0.07 | -0.39 | 1.71 |
| O60664 | Perilipin-3 | PLIN3 | | 2.84 | 1.75 | | -0.72 | -0.67 | 1.27 | -0.79 | -0.84 | -0.78 | -0.79 | 0.71 | 1.33 | 1.27 |
| Q96AX1 | Vacuolar protein sorting-associated protein 33A | VPS33A | | 2.84 | 2.27 | | 0.16 | -0.02 | -1.51 | 0.70 | -1.41 | -0.63 | -0.38 | 1.62 | 0.73 | 0.73 |
| P01019 | Angiotensinogen | AGT | | 2.83 | 3.29 | | 1.23 | -1.35 | -0.68 | -0.49 | -1.34 | 1.10 | 1.07 | 0.84 | -0.24 | -0.14 |
| P01700 | Immunoglobulin lambda variable 1-47 | IGLV1-47 | | 2.76 | 4.08 | | 0.02 | -0.03 | -1.72 | 0.41 | -1.68 | 0.48 | -0.02 | 1.50 | 0.42 | 0.62 |
| P36959 | GMP reductase 1 | GMPR | | 2.74 | 4.81 | | -1.13 | -1.25 | 1.03 | -0.62 | -1.37 | 1.06 | 0.20 | 0.74 | 0.33 | 1.01 |
| P54803 | Galactocerebrosidase | GALC | | 2.73 | 6.91 | | -0.58 | -1.29 | -1.55 | 0.12 | -0.38 | 1.32 | 1.42 | -0.19 | 0.53 | 0.61 |
| Q9BUT1 | 3-hydroxybutyrate dehydrogenase type 2 | BDH2 | | 2.72 | 4.69 | | -0.54 | -0.87 | 0.27 | 0.09 | -2.17 | 0.62 | 0.42 | 1.50 | 0.09 | 0.60 |
| Q7Z7A4 | PX domain-containing protein kinase-like protein | PXK | | 2.72 | 2.23 | | -0.66 | -2.24 | 0.55 | 0.54 | -0.27 | 0.14 | -0.71 | 0.93 | 0.88 | 0.84 |
| P07741 | Adenine phosphoribosyltransferase | APRT | | 2.71 | 3.53 | | -0.43 | 0.70 | -1.46 | 0.29 | -1.94 | 0.82 | 1.11 | -0.02 | 0.38 | 0.54 |
| P01591 | Immunoglobulin J chain | JCHAIN | | 2.70 | 5.30 | | -0.82 | -1.51 | 0.40 | -0.49 | -0.89 | 0.46 | 0.92 | 1.77 | -0.49 | 0.65 |
| Q63HR2 | Tensin-2 | TNS2 | | 2.70 | 1.79 | | 1.45 | -0.76 | -0.84 | -0.79 | -0.83 | 1.50 | 1.25 | -0.48 | -0.52 | 0.02 |
| P11387 | DNA topoisomerase 1 | TOP1 | | 2.68 | 5.15 | | 0.65 | -1.14 | -0.87 | -0.75 | -1.15 | 1.20 | 0.23 | 1.45 | -0.51 | 0.89 |
| Q13938 | Calcyphosin | CAPS | | 2.68 | 6.30 | | -0.49 | -1.99 | -0.55 | -0.37 | -0.16 | 1.60 | 0.33 | 0.31 | 0.05 | 1.25 |
| O95154 | Aflatoxin B1 aldehyde reductase member 3 | AKR7A3 | | 2.67 | 10.39 | | -0.13 | -1.22 | -1.10 | -0.50 | -1.22 | 1.37 | 0.87 | 1.20 | 0.66 | 0.08 |
| P02787 | Serotransferrin | TF | | 2.64 | 2.81 | | -0.04 | -2.30 | -0.01 | 0.43 | -0.46 | -0.36 | 0.10 | 1.60 | 0.66 | 0.38 |
| O14980 | Exportin-1 | XPO1 | | 2.62 | 5.77 | | 0.10 | -0.45 | -1.52 | 0.05 | -1.66 | 0.91 | 1.19 | 1.20 | 0.09 | 0.09 |
| Q9NWV4 | CXXC motif containing zinc binding protein | CZIB | | 2.61 | 6.52 | | -1.26 | -1.45 | -0.10 | -1.36 | 0.33 | 1.25 | 0.73 | 0.40 | 0.74 | 0.72 |
| O15067 | Phosphoribosylformylglycinamidine synthase | PFAS | | 2.57 | 2.48 | | 1.21 | -1.07 | -0.49 | -1.19 | -0.64 | 1.59 | 1.05 | 0.38 | -0.12 | -0.71 |
| Q9Y2W1 | Thyroid hormone receptor-associated protein 3 | THRAP3 | | 2.53 | 2.28 | | 0.20 | -1.82 | -0.82 | -0.10 | 0.46 | 1.98 | 0.77 | -0.07 | -0.14 | -0.47 |
| Q96C23 | Aldose 1-epimerase | GALM | | 2.52 | 6.94 | | -1.07 | -0.30 | -1.32 | 0.12 | -1.12 | 1.72 | -0.01 | 0.74 | 0.14 | 1.10 |
| Q96TA1 | Protein Niban 2 | NIBAN2 | | 2.45 | 4.65 | | 0.19 | -1.63 | 0.12 | -0.23 | -1.58 | 1.45 | 0.91 | -0.15 | 0.03 | 0.90 |
| Q92673 | Sortilin-related receptor | SORL1 | | 2.44 | 5.77 | | -0.19 | -0.73 | -0.92 | -0.23 | -1.45 | 0.31 | 2.24 | 0.24 | 0.35 | 0.37 |
| P01742 | Immunoglobulin heavy variable 1-69 | IGHV1-69 | | 2.42 | 3.50 | | 0.18 | -0.70 | -0.87 | -0.70 | -0.75 | 1.68 | -0.59 | 1.21 | -0.71 | 1.25 |
| P00739 | Haptoglobin-related protein | HPR | | 2.42 | 2.65 | | 0.34 | 0.58 | -0.38 | -0.66 | -2.19 | -0.17 | 0.66 | 1.31 | -0.40 | 0.91 |
| Q13616 | Cullin-1 | CUL1 | | 2.40 | 3.89 | | 0.00 | -0.37 | -2.15 | 0.33 | -0.67 | 0.98 | 1.60 | 0.41 | -0.01 | -0.12 |
| P29375 | Lysine-specific demethylase 5A | KDM5A | | 2.40 | 6.05 | | -1.04 | -1.24 | -0.07 | -0.70 | -0.58 | 1.16 | 1.84 | 0.86 | 0.02 | -0.26 |
| P50995 | Annexin A11 | ANXA11 | | 2.39 | 4.01 | | -0.58 | -1.26 | 0.64 | -0.98 | -0.71 | 1.57 | -0.17 | 0.61 | -0.54 | 1.42 |
| B2RTY4 | Unconventional myosin-IXa | MYO9A | | 2.39 | 3.58 | | 0.62 | -0.56 | -2.34 | -0.32 | -0.21 | 0.89 | 1.00 | 0.71 | -0.32 | 0.54 |
| O94874 | E3 UFM1-protein ligase 1 | UFL1 | | 2.39 | 4.54 | | -0.16 | -0.92 | -0.90 | -0.37 | -0.92 | 1.50 | 1.75 | -0.51 | 0.82 | -0.29 |
| Q86VP6 | Cullin-associated NEDD8-dissociated protein 1 | CAND1 | | 2.39 | 3.17 | | 0.60 | -1.23 | -0.80 | -0.71 | -0.43 | 1.54 | 0.02 | 0.02 | -0.72 | 1.70 |
| P10301 | Ras-related protein R-Ras | RRAS | | 2.38 | 7.49 | | -0.74 | -0.71 | -0.74 | -0.24 | -1.47 | 1.54 | 0.67 | 0.39 | -0.19 | 1.48 |
| Q5JVF3 | PCI domain-containing protein 2 | PCID2 | | 2.31 | 3.91 | | -0.45 | -0.55 | -0.78 | -0.62 | -0.71 | -0.53 | -0.63 | 1.21 | 1.28 | 1.79 |
| Q86TU7 | Actin-histidine N-methyltransferase | SETD3 | | 2.30 | 2.11 | | -0.14 | 0.02 | -1.07 | 0.06 | -0.91 | 0.33 | 0.25 | 0.00 | -0.97 | 2.43 |
| Q8N6H7 | ADP-ribosylation factor GTPase-activating protein 2 | ARFGAP2 | | 2.29 | 2.86 | | 1.11 | -0.82 | -1.22 | -0.76 | -0.70 | 0.69 | 0.88 | -1.16 | 1.00 | 0.98 |
| P0CG30 | Glutathione S-transferase theta-2B | GSTT2B | | 2.28 | 3.12 | | 0.30 | -1.49 | 0.28 | 0.34 | -2.07 | 0.54 | 0.29 | 1.24 | 0.01 | 0.56 |
| Q9C040 | Tripartite motif-containing protein 2 | TRIM2 | | 2.26 | 2.71 | | -0.51 | -0.90 | -0.03 | -0.39 | -0.62 | 1.88 | 1.71 | -0.91 | -0.30 | 0.09 |
| O75223 | Gamma-glutamylcyclotransferase | GGCT | | 2.24 | 2.64 | | -0.40 | -0.15 | -0.98 | -0.87 | 0.00 | 1.22 | -0.70 | 2.16 | -0.61 | 0.32 |
| O43592 | Exportin-T | XPOT | | 2.24 | 2.67 | | -0.63 | 0.27 | -1.09 | 1.11 | -2.08 | 0.58 | 0.06 | 0.18 | 1.05 | 0.54 |
| P15531 | Nucleoside diphosphate kinase A | NME1 | | 2.24 | 3.61 | | -1.50 | 0.46 | -1.74 | 0.19 | -0.17 | -0.07 | 0.61 | 1.63 | -0.05 | 0.64 |
| Q9Y617 | Phosphoserine aminotransferase | PSAT1 | | 2.24 | 6.50 | | -0.34 | -0.95 | -0.51 | 0.06 | -1.88 | 1.46 | -0.18 | 1.15 | 0.50 | 0.68 |
| P08758 | Annexin A5 | ANXA5 | | 2.23 | 5.32 | | -1.04 | -0.51 | -0.43 | -0.24 | -1.24 | 1.88 | -0.05 | 0.09 | 0.02 | 1.52 |
| O43665 | Regulator of G-protein signaling 10 | RGS10 | | 2.23 | 4.05 | | 0.41 | -1.29 | -1.67 | 0.65 | -1.16 | 0.28 | 0.11 | 1.00 | 1.07 | 0.60 |
| P48454 | Serine/threonine-protein phosphatase 2B catalytic subunit gamma | PPP3CC | | 2.22 | 2.53 | | -0.01 | -0.42 | -1.55 | -0.33 | 0.04 | -0.36 | 0.34 | 0.22 | -0.36 | 2.43 |
| P51531 | Probable global transcription activator SNF2L2 | SMARCA2 | | 2.22 | 5.82 | | -0.18 | -1.26 | -0.77 | -0.54 | -0.87 | 1.46 | -0.29 | 0.99 | -0.13 | 1.59 |
| Q9P2P6 | StAR-related lipid transfer protein 9 | STARD9 | | 2.20 | 2.18 | | 1.05 | -0.86 | -1.04 | -0.90 | -0.26 | 1.52 | 0.96 | 0.94 | -0.95 | -0.45 |
| Q9NQT8 | Kinesin-like protein KIF13B | KIF13B | | 2.20 | 3.53 | | -0.58 | -0.05 | -1.68 | -0.21 | -0.22 | 0.26 | -0.46 | 2.29 | 0.19 | 0.47 |
| O15540 | Fatty acid-binding protein, brain | FABP7 | | 2.17 | 5.00 | | -0.57 | -0.36 | -0.57 | 0.37 | -2.17 | 1.37 | -0.14 | 0.86 | 0.48 | 0.72 |
| Q8WXI4 | Acyl-coenzyme A thioesterase 11 | ACOT11 | | 2.15 | 2.29 | | 0.79 | -0.71 | -0.76 | -0.71 | -0.73 | 1.80 | 1.06 | -0.84 | -0.72 | 0.81 |
| Q9Y3D0 | Cytosolic iron-sulfur assembly component 2B | CIAO2B | | 2.15 | 3.99 | | -0.82 | -0.99 | -1.02 | 0.85 | -0.90 | -0.89 | 1.36 | 0.46 | 1.02 | 0.93 |
| Q9UNF0 | Protein kinase C and casein kinase substrate in neurons protein 2 | PACSIN2 | | 2.14 | 2.39 | | 1.16 | -1.38 | -0.60 | -1.00 | -0.32 | 1.01 | 1.40 | 0.66 | -0.93 | 0.01 |
| P01834 | Immunoglobulin kappa constant | IGKC | | 2.14 | 5.34 | | -0.07 | -1.90 | -0.17 | -0.59 | -0.59 | 1.39 | -0.10 | 1.40 | -0.22 | 0.85 |
| Q96GR2 | Long-chain-fatty-acid--CoA ligase ACSBG1 | ACSBG1 | | 2.13 | 3.59 | | 0.40 | -1.23 | -1.40 | 0.60 | -1.10 | 1.24 | 1.10 | -0.65 | 0.68 | 0.37 |
| Q96GA7 | Serine dehydratase-like | SDSL | | 2.13 | 3.33 | | 0.84 | -1.37 | -1.12 | 0.31 | -1.30 | 1.62 | 0.14 | -0.24 | 0.40 | 0.72 |
| P28161 | Glutathione S-transferase Mu 2 | GSTM2 | | 2.13 | 6.89 | | -0.53 | -0.84 | -1.34 | -0.20 | -0.90 | 1.87 | -0.15 | 0.79 | 0.21 | 1.10 |
| Q5VYK3 | Proteasome adapter and scaffold protein ECM29 | ECPAS | | 2.10 | 3.87 | | 0.58 | 0.10 | -1.66 | -0.39 | -1.49 | 1.38 | 1.00 | 0.68 | -0.37 | 0.17 |
| Q8WTS6 | Histone-lysine N-methyltransferase SETD7 | SETD7 | | 2.10 | 5.46 | | -1.90 | -0.81 | -0.41 | -0.57 | 0.34 | 1.27 | 0.82 | 0.22 | -0.26 | 1.31 |
| O75533 | Splicing factor 3B subunit 1 | SF3B1 | | 2.09 | 3.14 | | 0.79 | -0.62 | -1.01 | -0.90 | -0.80 | 1.67 | 0.49 | 0.23 | -1.02 | 1.18 |
| Q5EBL8 | PDZ domain-containing protein 11 | PDZD11 | | 2.08 | 2.56 | | -0.82 | -1.06 | -1.06 | 0.29 | 0.39 | 0.58 | -0.96 | 2.11 | 0.15 | 0.39 |
| P51648 | Aldehyde dehydrogenase family 3 member A2 | ALDH3A2 | | 2.06 | 3.43 | | -0.09 | -0.01 | -0.33 | 0.15 | -2.43 | 1.35 | 0.21 | -0.18 | 0.37 | 0.96 |
| P00390 | Glutathione reductase, mitochondrial | GSR | | 2.06 | 2.37 | | -0.46 | -0.86 | -1.04 | -0.36 | 0.54 | 0.43 | -0.90 | 2.24 | -0.27 | 0.67 |
| Q96K76 | Ubiquitin carboxyl-terminal hydrolase 47 | USP47 | | 2.06 | 2.27 | | 0.42 | -0.12 | -2.10 | 0.36 | -0.64 | 1.96 | 0.06 | -0.07 | 0.19 | -0.06 |
| O14939 | Phospholipase D2 | PLD2 | | 2.06 | 4.57 | | -1.24 | -1.42 | 0.57 | 0.26 | -1.32 | 1.30 | 1.15 | -0.06 | 0.36 | 0.41 |
| P58107 | Epiplakin | EPPK1 | | 2.04 | 2.32 | | -0.52 | 0.66 | -0.20 | 0.05 | -2.09 | 0.98 | 0.92 | -0.94 | 0.06 | 1.09 |

| | | | | | | | | | | | | | | |
|---|---|---|---|---|---|---|---|---|---|---|---|---|---|---|
| P35052 | Glypican-1 | GPC1 | 2.04 | 3.61 | -0.45 | -0.57 | -0.77 | -0.57 | -0.63 | 1.85 | -0.58 | 1.63 | -0.56 | 0.65 |
| Q63HK | Teashirt homolog 3 | TSHZ3 | 2.04 | 1.13 | 1.17 | -0.60 | -0.64 | -0.60 | -0.61 | 1.58 | 1.57 | -0.76 | -0.56 | -0.56 |
| 5 P09211 | Glutathione S-transferase P | GSTP1 | 2.03 | 5.21 | -0.77 | -1.05 | -0.39 | -0.51 | -0.83 | 1.43 | -0.36 | 0.92 | -0.21 | 1.77 |
| Q93034 | Cullin-5 | CUL5 | 2.02 | 1.59 | 1.63 | -1.34 | -0.12 | -0.48 | -1.35 | 1.35 | 0.68 | 0.15 | -0.17 | -0.37 |
| P01859 | Immunoglobulin heavy constant gamma 2 | IGHG2 | 2.01 | 3.70 | -0.10 | -2.41 | -0.29 | 0.01 | -0.03 | 0.85 | -0.28 | 1.34 | 0.25 | 0.66 |
| P09972 | Fructose-bisphosphate aldolase C | ALDOC | 2.00 | 4.00 | -0.54 | -1.33 | 0.14 | -0.70 | -0.57 | 1.23 | -0.53 | 1.38 | -0.55 | 1.46 |
| Q13884 | Beta-1-syntrophin | SNTB1 | 2.00 | 1.63 | 1.23 | -0.58 | -1.16 | -0.39 | -0.75 | 1.48 | 1.44 | -0.87 | -0.30 | -0.10 |
| Q13685 | Angio-associated migratory cell protein | AAMP | 2.00 | 3.54 | 1.00 | -0.63 | -1.91 | -0.30 | -0.92 | 0.32 | 1.31 | 1.06 | -0.14 | 0.22 |
| Q92820 | Gamma-glutamyl hydrolase | GGH | 2.00 | 2.41 | -0.26 | -1.63 | -1.66 | 0.30 | 1.04 | -0.22 | 0.52 | 1.22 | -0.04 | 0.73 |
| Q9H4E | Differentially expressed in FDCP 6 homolog | DEF6 | 2.00 | 2.01 | -0.52 | 0.55 | -1.00 | -0.08 | -0.90 | 1.89 | 1.30 | -1.15 | -0.13 | 0.04 |
| 7 | Core histone macro-H2A.1 | H2AFY | 1.99 | 2.64 | 0.27 | -1.58 | 0.32 | -0.28 | -1.01 | 0.13 | 2.27 | -0.08 | -0.19 | 0.17 |
| O75367 | Guanine nucleotide-binding protein G(I)/G(S)/G(O) subunit gammaGNG12 | | 1.99 | 2.64 | -0.83 | 0.50 | -1.06 | -0.08 | -0.85 | 1.08 | 0.07 | -1.08 | 0.31 | 1.94 |
| Q9UBI6 | Immunoglobulin heavy constant gamma 1 | IGHG1 | 1.99 | 3.73 | -0.55 | -1.56 | 0.53 | -0.69 | -0.52 | 1.58 | 0.06 | 1.08 | -0.85 | 0.92 |
| P01857 | Immunoglobulin heavy variable 1-2 | IGHV1-2 | 1.97 | 2.78 | 0.55 | -0.69 | -0.85 | -0.65 | -0.78 | 1.49 | -0.78 | 1.15 | -0.77 | 1.32 |
| P23083 | Transcription activator BRG1 | SMARCA | 1.96 | 3.90 | 0.10 | -0.77 | -2.45 | 0.19 | -0.09 | 0.87 | 0.06 | 0.96 | 0.66 | 0.49 |
| P51532 | Core-binding factor subunit beta | 4 CBFB | 1.96 | 3.24 | -0.40 | -0.53 | -0.79 | -0.50 | -0.57 | 1.61 | -0.58 | -0.90 | 1.51 | 1.15 |
| Q13951 | Metastasis-associated protein MTA2 | MTA2 | 1.96 | 3.06 | -0.60 | -1.55 | -0.32 | -0.08 | 0.01 | 1.60 | 1.81 | -0.25 | -0.17 | -0.45 |
| Q94TK66 | Protein kinase C and casein kinase substrate in neurons protein 3PACSIN3 | | 1.95 | 3.99 | -0.80 | -1.35 | -0.16 | 0.71 | -1.28 | 0.38 | -0.73 | 1.07 | 0.72 | 1.43 |
| P01860 | Immunoglobulin heavy constant gamma 3 | IGHG3 | 1.95 | 5.19 | -0.14 | -1.90 | -0.01 | -0.33 | -0.88 | 1.64 | -0.16 | 0.86 | -0.08 | 1.01 |
| O14907 | Tax1-binding protein 3 | TAX1BP | 1.94 | 3.48 | -0.42 | -0.20 | -0.30 | 0.50 | -2.32 | 1.14 | 0.60 | -0.44 | 0.42 | 1.01 |
| P10643 | Complement component C7 | 3 C7 | 1.94 | 3.19 | 1.07 | -1.53 | 0.01 | -0.67 | -1.49 | 1.05 | 1.12 | 0.32 | -0.37 | 0.49 |
| O14579 | Coatomer subunit epsilon | COPE | 1.93 | 4.05 | 0.37 | -0.39 | -0.33 | -0.35 | -2.25 | 0.89 | 0.92 | -0.04 | -0.15 | 1.32 |
| Q96CN | EVI5-like protein | EVI5L | 1.93 | 2.84 | 1.11 | -1.13 | -0.68 | -0.53 | -1.14 | 1.35 | 1.09 | 0.94 | -0.33 | -0.67 |
| 4 | Calcium-binding protein 39 | CAB39 | 1.92 | 2.30 | -0.86 | -1.11 | -1.15 | 0.15 | 0.89 | -0.73 | -0.30 | 1.66 | 0.26 | 1.20 |
| Q9X5D764DH72 | Immunoglobulin kappa variable 1-6 | IGKV1-6 | 1.92 | 1.19 | 1.44 | -0.69 | -0.75 | -0.67 | -0.64 | 1.63 | 1.16 | -0.77 | -0.66 | -0.04 |
| Q9Y3C8 | Ubiquitin-fold modifier-conjugating enzyme 1 Annexin | UFC1 | 1.92 | 4.04 | 0.26 | 0.28 | -1.96 | -0.71 | -0.78 | 1.70 | 0.21 | -0.15 | 0.19 | 0.96 |
| P04083 | A1 | ANXA1 | 1.92 | 4.24 | -1.20 | -0.57 | -0.75 | 0.52 | -1.00 | 1.79 | -0.81 | 0.49 | 0.51 | 1.03 |
| Q9NYL | Mitogen-activated protein kinase kinase kinase 20 | MAP3K2 | 1.92 | 2.45 | 0.76 | -0.88 | -1.10 | -0.02 | -0.95 | 1.46 | 0.91 | -1.25 | 0.06 | 1.01 |
| 2 P08133 | Annexin A6 | 0 | 1.91 | 5.72 | -0.95 | -1.52 | -0.89 | -0.23 | 0.17 | 0.27 | 0.72 | 2.06 | 0.18 | 0.20 |
| P80748 | Immunoglobulin lambda variable 3-21 | ANXA6 | 1.91 | 2.92 | -0.41 | -0.51 | -0.70 | -0.53 | -0.47 | 1.73 | -0.46 | 2.03 | -0.48 | -0.19 |
| P12109 | Collagen alpha-1(VI) chain | IGLV3-21 | 1.89 | 2.96 | -0.72 | -0.74 | 0.69 | -0.78 | -0.93 | 0.25 | 2.40 | 0.04 | -0.39 | 0.18 |
| P23297 | Protein S100-A1 | S100A6A1 | 1.88 | 3.67 | 0.08 | 0.54 | -1.76 | 0.00 | -1.73 | 0.80 | 1.08 | 0.45 | -0.21 | 0.76 |
| Q7Z406 | Myosin-14 | MYH14 | 1.88 | 2.47 | 1.29 | -1.44 | 0.14 | -0.63 | -1.63 | 1.10 | 0.93 | -0.14 | 0.02 | 0.35 |
| Q9H898 | Zinc finger matrin-type protein 4 | ZMAT4 | 1.88 | 1.16 | 1.44 | -0.64 | -0.73 | -0.65 | -0.71 | 1.63 | 1.16 | -0.78 | -0.67 | -0.05 |
| O94826 | Mitochondrial import receptor subunit TOM70 | TOMM70 | 1.87 | 3.13 | 0.02 | -0.82 | 0.78 | -0.49 | -2.04 | 0.77 | 1.34 | 0.44 | -0.60 | 0.60 |
| Q9Y295 | Developmentally-regulated GTP-binding protein 1 | DRG1 | 1.87 | 3.62 | 0.01 | 0.36 | -1.65 | -0.50 | -0.96 | 1.34 | 1.33 | -0.83 | 0.05 | 0.84 |
| Q9UNK | Syntaxin-8 | STX8 | 1.85 | 2.74 | -0.76 | -0.86 | 0.83 | -0.76 | -0.81 | 0.35 | 1.56 | -1.06 | 0.07 | 1.45 |
| 0 P19022 | Cadherin-2 | CDH | 1.84 | 2.24 | -0.34 | -0.50 | -0.57 | -0.37 | -0.42 | -0.48 | 2.48 | 1.10 | -0.48 | -0.42 |
| P21266 | Glutathione S-transferase Mu 3 | 2GSTM3 | 1.83 | 6.50 | -1.00 | -1.07 | -1.01 | -0.09 | -0.58 | 1.48 | 0.08 | 0.23 | 0.16 | 1.80 |
| O43765 | Small glutamine-rich tetratricopeptide repeat-containing protein alSGTA | | 1.83 | 2.67 | 0.01 | -0.20 | -0.26 | -0.53 | -1.39 | 1.88 | 1.42 | -1.05 | -0.05 | 0.16 |
| Q9BWD | Acetyl-CoA acetyltransferase, cytosolic | ACAT2 | 1.83 | 2.94 | 0.37 | -0.87 | -1.25 | -0.42 | -0.33 | 1.04 | -0.85 | 2.11 | 0.15 | 0.04 |
| 1 Q13636 | Ras-related protein Rab-31 | RAB31 | 1.82 | 4.35 | -0.78 | -1.44 | -1.66 | 0.59 | 0.20 | -0.06 | 1.07 | 0.32 | 0.45 | 1.31 |
| P06213 | Insulin receptor | INSR | 1.82 | 3.62 | 0.08 | 0.02 | -1.72 | 0.47 | -1.64 | 0.51 | 0.41 | -0.31 | 0.75 | 1.42 |
| O00567 | Nucleolar protein 56 | NOP56 | 1.81 | 2.77 | -0.16 | -0.18 | -2.69 | 0.21 | 0.31 | 0.84 | 0.20 | 0.26 | 0.49 | 0.72 |
| P0DOX7 | Immunoglobulin kappa light chain | Immunoglobulin | 1.81 | 4.44 | -0.53 | -1.80 | -0.17 | -0.50 | -0.06 | 1.09 | -0.32 | 1.81 | -0.26 | 0.75 |
| P54284 | Voltage-dependent L-type calcium channel subunit beta-3 | CACNB3 | 1.81 | 1.65 | -0.18 | 0.74 | 0.48 | -1.37 | -1.35 | 0.72 | 0.32 | 0.88 | 1.12 | -1.36 |
| Q9UBB9 | Tuftelin-interacting protein 11 | TFIP11 | 1.81 | 2.76 | 0.88 | -1.39 | -1.37 | 0.68 | -1.21 | 1.08 | 0.73 | -0.44 | 0.36 | 0.69 |
| P35625 | Metalloproteinase inhibitor 3 | TIMP3 | 1.80 | 2.82 | 0.07 | -0.45 | -0.57 | -0.03 | -1.45 | -0.68 | -0.42 | 1.34 | 0.23 | 1.97 |
| Q99567 | Nuclear pore complex protein Nup88 | NUP88 | 1.79 | 2.24 | 0.44 | -0.76 | -0.83 | -0.12 | -0.84 | 1.75 | 1.59 | -1.02 | -0.39 | 0.18 |
| P08670 | Vimentin | VIM | 1.79 | 2.10 | -0.21 | -0.06 | -0.33 | -0.77 | -0.70 | 1.93 | -0.01 | -0.78 | -0.75 | 1.69 |
| Q8TEB1 | DDB1- and CUL4-associated factor 11 | DCAF11 | 1.79 | 3.26 | -0.74 | 0.63 | -1.51 | 0.44 | -1.43 | 0.92 | 1.44 | 0.41 | 0.41 | -0.57 |
| P12955 | Xaa-Pro dipeptidase | PEPD | 1.78 | 3.03 | 0.31 | -0.87 | 0.20 | -0.56 | -1.57 | -0.12 | 0.56 | 2.24 | -0.16 | -0.03 |
| P63172 | Dynein light chain Tctex-type 1 | DYNLT1 | 1.78 | 2.11 | 0.28 | 0.66 | -1.20 | 0.43 | -2.20 | 0.98 | 0.37 | -0.49 | 0.55 | 0.62 |
| Q7Z3V4 | Ubiquitin-protein ligase E3B | UBE3B | 1.78 | 2.28 | -0.94 | -0.10 | -1.00 | 0.09 | -0.20 | 0.23 | -1.34 | 1.22 | 0.12 | 1.92 |
| P52566 | Rho GDP-dissociation inhibitor 2 | ARHGDIB | 1.77 | 4.83 | -1.83 | -0.12 | -0.57 | 0.39 | -1.05 | 1.05 | -0.61 | 0.68 | 0.94 | 1.11 |
| P13797 | Plastin-3 | PLS3 | 1.76 | 2.40 | -0.76 | -0.92 | -1.12 | -0.52 | 1.17 | 0.21 | 1.63 | 1.09 | 0.11 | -0.89 |
| Q86YJ6 | Threonine synthase-like 2 | THNSL2 | 1.75 | 3.25 | -0.72 | -0.75 | -0.99 | 0.75 | -0.89 | 0.56 | -0.73 | 0.16 | 0.51 | 2.11 |
| P31153 | S-adenosylmethionine synthase isoform type-2 | MAT2A | 1.75 | 3.64 | 0.34 | -1.17 | -0.38 | -0.78 | -0.83 | 0.47 | 1.97 | 1.25 | -0.35 | -0.52 |
| Q17R31 | Putative deoxyribonuclease TATDN3 | TATDN3 | 1.73 | 2.26 | 0.51 | -0.75 | -0.27 | -0.81 | -0.80 | 0.77 | 0.55 | 2.21 | -0.54 | -0.86 |
| Q01105 | Protein SET | SET | 1.73 | 2.10 | -0.31 | -0.03 | -0.67 | -0.55 | -0.52 | -0.53 | -0.48 | 2.68 | 0.02 | 0.40 |
| Q86WG | Myotubularin-related protein 13 | SBF2 | 1.73 | 4.23 | 0.77 | -1.24 | -1.26 | 0.18 | -1.54 | 1.07 | 1.22 | 0.14 | 0.29 | 0.38 |
| 5 P49756 | RNA-binding protein 25 | RBM25 | 1.73 | 3.45 | 0.62 | -1.26 | -1.38 | -0.71 | 0.07 | 1.57 | 1.14 | 0.72 | -0.33 | -0.44 |
| P02647 | Apolipoprotein A-I | APOA1 | 1.73 | 2.13 | 0.79 | 0.05 | -1.03 | 0.00 | -1.81 | 0.09 | 0.59 | 1.58 | -0.89 | 0.62 |
| P01876 | Immunoglobulin heavy constant alpha 1 | IGHA1 | 1.72 | 4.34 | -0.42 | -1.60 | -0.11 | -0.66 | -0.29 | 0.74 | 0.13 | 2.18 | -0.45 | 0.48 |
| Q6NUM | All-trans-retinol 13,14-reductase | RETSAT | 1.72 | 2.38 | 0.66 | -0.68 | -0.79 | -0.69 | -0.69 | 1.06 | 1.42 | -1.02 | -0.66 | 1.38 |
| 9 P04150 | Glucocorticoid receptor | NR3C1 | 1.72 | 1.80 | 0.76 | -0.77 | -1.49 | -1.32 | 1.02 | 0.03 | 0.13 | 1.49 | 0.61 | -0.47 |
| O75165 | DnaJ homolog subfamily C member 13 | DNAJC13 | 1.72 | 1.43 | 0.95 | -0.13 | -1.20 | -0.51 | -0.63 | 1.55 | 1.42 | -1.22 | -0.26 | 0.02 |
| Q8NBX | Saccharopine dehydrogenase-like oxidoreductase | SCCPDH | 1.72 | 1.53 | -0.24 | -0.16 | -0.15 | 0.02 | -1.11 | 2.00 | 1.26 | -1.30 | 0.25 | -0.57 |
| 0 | SPRY domain-containing protein 7 | SPRYD7 | 1.72 | 5.01 | -0.90 | -1.05 | -1.30 | -0.90 | 0.90 | 0.13 | 0.10 | 0.32 | 1.26 | 1.43 |
| Q5W111 | Apoptosis inhibitor 5 | API5 | 1.71 | 1.93 | 1.22 | -0.11 | -0.51 | -2.00 | -0.48 | 1.29 | 1.06 | 0.12 | -0.41 | -0.18 |
| Q9BZZ5 | AP-3 complex subunit mu-2 | AP3M2 | 1.71 | 3.00 | -0.10 | 0.03 | -0.83 | -0.90 | -0.78 | 1.88 | -0.06 | -0.46 | -0.48 | 1.70 |
| P53677 | Nuclear receptor corepressor 1 | NCOR1 | 1.71 | 1.77 | -1.18 | -0.20 | 0.69 | -0.32 | -0.77 | 1.75 | 1.56 | -0.29 | -0.47 | -0.77 |
| O75376 | PTB domain-containing engulfment adapter protein 1 GTPase | GULP1 | 1.71 | 1.80 | 1.08 | -0.46 | -1.46 | -1.13 | 0.19 | 0.99 | 1.31 | -0.30 | -0.93 | 0.71 |
| Q9UBP9 | IMAP family member 4 | GIMAP4 | 1.71 | 5.31 | -0.76 | -0.67 | -1.86 | 0.20 | -0.22 | 1.96 | 0.43 | 0.48 | 0.41 | 0.03 |
| Q9NUV | eIF-2-alpha kinase activator GCN1 | GCN1 | 1.71 | 1.02 | 1.43 | -1.35 | -0.76 | -0.22 | -0.27 | 1.34 | 1.33 | -0.75 | -0.26 | -0.49 |
| 9 | Glucose-6-phosphate isomerase | GPI | 1.70 | 6.42 | -0.52 | -0.08 | -0.65 | -0.29 | -2.11 | 1.13 | 1.35 | 0.54 | 0.02 | 0.60 |
| Q92616 | Immunoglobulin lambda constant 2 | IGLC2 | 1.69 | 5.11 | -0.74 | -1.71 | 0.11 | -0.06 | -0.85 | 1.52 | -0.50 | 0.92 | 0.14 | 1.17 |
| P06744 | Delta(3,5)-Delta(2,4)-dienoyl-CoA isomerase, mitochondrial Serum | ECH1 | 1.69 | 2.70 | -0.83 | -1.65 | 0.74 | -0.54 | -0.03 | -0.43 | 0.22 | 2.05 | -0.08 | 0.55 |
| P0DOY2 | amyloid P-component | APCS | 1.69 | 2.08 | -0.78 | -0.78 | 0.93 | -0.81 | -0.53 | 0.10 | -0.80 | 1.67 | -0.51 | 1.51 |
| Q13011 | Dual specificity protein phosphatase 23 | DUSP23 | 1.68 | 2.70 | 0.33 | -1.62 | -1.79 | 0.27 | 0.45 | 1.30 | 0.10 | -0.15 | 0.07 | 1.03 |
| P02743 | Acetolactate synthase-like protein | ILVBL | 1.68 | 5.20 | -0.35 | -1.14 | -1.62 | 0.33 | -0.49 | 0.61 | 1.02 | 0.96 | -0.67 | 1.35 |
| Q9BVJ7 | Glutathione S-transferase Mu 5 | GSTM5 | 1.67 | 2.97 | 0.29 | 0.13 | -1.59 | -1.35 | 0.08 | 1.09 | -0.42 | -0.55 | 0.85 | 1.47 |
| A1L0T0 | Lactadherin | MFGE8 | 1.67 | 3.17 | 0.31 | -0.85 | -0.97 | -0.86 | -0.27 | -0.70 | 0.20 | 1.37 | -0.23 | 1.99 |
| P46439 | | | | | | | | | | | | | | |
| Q08431 | | | | | | | | | | | | | | |

| | | | | | | | | | | | | | | |
|---|---|---|---|---|---|---|---|---|---|---|---|---|---|---|
| O00299 | Chloride intracellular channel protein 1 | CLIC1 | 1.67 | 4.69 | -0.85 | -0.04 | -0.55 | -0.14 | -1.59 | 1.79 | -0.06 | -0.15 | 0.10 | 1.48 |
| Q8TC07 | TBC1 domain family member 15 | TBC1D15 | 1.67 | 1.19 | -1.03 | 0.62 | -1.54 | 0.35 | 0.29 | 1.16 | 0.64 | -1.61 | 0.51 | 0.62 |
| P35270 | Sepiapterin reductase | SPR | 1.67 | 2.75 | -0.51 | -0.75 | 0.49 | -0.82 | -0.82 | -0.76 | -0.72 | 1.84 | 1.12 | 0.92 |
| P11172 | Uridine 5'-monophosphate synthase | UMPS | 1.66 | 1.80 | 0.18 | -0.43 | -0.87 | -0.02 | -0.70 | 0.80 | 2.10 | -1.54 | 0.16 | 0.33 |
| Q14005 | Pro-interleukin-16 | IL16 | 1.65 | 2.73 | -1.53 | 0.39 | -2.10 | 0.57 | 0.21 | 0.23 | 0.19 | 0.36 | 1.02 | 0.66 |
| P80297 | Metallothionein-1X | MT1X | 1.65 | 2.00 | 0.24 | 0.39 | -0.06 | -1.18 | -1.30 | 0.76 | 0.19 | 1.78 | -1.30 | 0.49 |
| Q12840 | Kinesin heavy chain isoform 5A | KIF5A | 1.65 | 3.98 | -0.77 | -0.13 | -0.94 | -0.47 | -0.77 | 0.38 | 2.23 | 1.13 | -0.52 | -0.14 |
| B9A064 | Immunoglobulin lambda-like polypeptide 5 | IGLL5 | 1.64 | 4.05 | -0.38 | -1.34 | 0.32 | -0.68 | -0.87 | 1.59 | -0.42 | 1.20 | -0.55 | 1.14 |
| Q8WVM7 | Cohesin subunit SA-1 | STAG1 | 1.64 | 0.97 | 1.63 | -0.69 | -0.79 | -0.53 | -0.75 | 2.02 | -0.11 | -0.24 | -0.49 | -0.06 |
| Q04760 | Lactoylglutathione lyase | GLO1 | 1.64 | 4.32 | 0.18 | -1.15 | -0.52 | -0.84 | -0.78 | 0.85 | 0.61 | 2.12 | -0.65 | 0.18 |
| Q14118 | Dystroglycan | DAG1 | 1.64 | 2.54 | -0.94 | 0.40 | 0.55 | -1.10 | -1.14 | 1.60 | -1.15 | 0.59 | 0.26 | 0.91 |
| Q5VW32 | BRO1 domain-containing protein BROX | BROX | 1.63 | 5.01 | -0.44 | -2.05 | -0.64 | 0.56 | -0.75 | 1.39 | 0.20 | 0.21 | 1.02 | 0.49 |
| P51178 | 1-phosphatidylinositol 4,5-bisphosphate phosphodiesterase delta-1 | PLCD1 | 1.63 | 3.91 | -0.03 | -1.27 | -1.08 | 0.45 | -0.94 | 1.39 | -0.87 | 0.17 | 1.28 | 0.90 |
| Q6IC98 | GRAM domain-containing protein 4 | GRAMD4 | 1.62 | 2.24 | -0.40 | -0.40 | -0.61 | -0.36 | -0.42 | -0.40 | -0.40 | -0.75 | 2.07 | 1.68 |
| O94913 | Pre-mRNA cleavage complex 2 protein Pcf11 | PCF11 | 1.61 | 3.95 | 0.60 | -1.66 | -1.79 | 0.30 | -0.50 | 0.57 | 0.45 | 0.16 | 0.79 | 1.09 |
| P37802 | Transgelin-2 | TAGLN2 | 1.60 | 4.27 | -0.74 | -0.32 | -1.47 | -0.96 | 0.50 | 0.34 | -0.42 | 1.29 | 0.04 | 1.75 |
| Q96B70 | Leukocyte receptor cluster member 9 | LENG9 | 1.60 | 2.09 | -0.32 | -0.37 | -0.57 | -0.37 | -0.48 | 2.25 | 1.46 | -0.72 | -0.46 | -0.42 |
| P04003 | C4b-binding protein alpha chain | C4BPA | 1.59 | 4.47 | -0.70 | -1.23 | 0.84 | -0.32 | -1.72 | 0.93 | 0.19 | 0.48 | 0.09 | 1.44 |
| P00915 | Carbonic anhydrase 1 | CA1 | 1.59 | 2.92 | -0.86 | -0.85 | 0.51 | -0.05 | -1.20 | -0.71 | 0.88 | 2.11 | 0.26 | -0.09 |
| O95837 | Guanine nucleotide-binding protein subunit alpha-14 | GNA14 | 1.59 | 2.71 | 0.54 | -0.10 | -1.77 | 0.60 | -1.67 | 1.21 | 0.87 | -0.21 | 0.12 | 0.40 |
| Q9NUQ2 | 1-acyl-sn-glycerol-3-phosphate acyltransferase epsilon | AGPAT5 | 1.58 | 1.35 | 1.25 | -0.94 | -1.05 | 0.33 | -1.04 | 1.22 | 1.04 | -1.22 | 0.45 | -0.03 |
| Q9ULL5 | Proline-rich protein 12 | PRR12 | 1.58 | 2.04 | 1.08 | -0.16 | -0.90 | 0.01 | -1.97 | 1.16 | 1.29 | -0.23 | -0.10 | -0.18 |
| Q16222 | UDP-N-acetylhexosamine pyrophosphorylase | UAP1 | 1.58 | 3.14 | -0.78 | -0.83 | -1.02 | 1.09 | -0.99 | -0.86 | 0.27 | 1.59 | 0.71 | 0.81 |
| Q9NQZ5 | StAR-related lipid transfer protein 7, mitochondrial | STARD7 | 1.57 | 2.30 | -0.52 | 0.23 | 0.01 | 0.49 | -2.32 | 0.59 | 0.48 | -0.80 | 1.18 | 0.67 |
| Q6PD62 | RNA polymerase-associated protein CTR9 homolog | CTR9 | 1.57 | 1.89 | 1.12 | -0.96 | -1.11 | -0.55 | -0.33 | 1.46 | 1.55 | -0.04 | -0.49 | -0.65 |
| Q8N4T8 | Carbonyl reductase family member 4 | CBR4 | 1.56 | 2.24 | -1.64 | 0.82 | 0.37 | 0.24 | -1.93 | 0.05 | 0.38 | 1.13 | 0.03 | 0.54 |
| Q96KN2 | Beta-Ala-His dipeptidase | CNDP1 | 1.56 | 2.22 | 0.42 | -1.39 | -1.60 | 0.15 | 0.38 | -0.17 | -0.57 | 1.53 | 1.20 | 0.05 |
| Q9H977 | WD repeat-containing protein 54 | WDR54 | 1.55 | 2.81 | -0.37 | 0.40 | -1.10 | 0.74 | -2.09 | 0.99 | -0.44 | 0.45 | 0.43 | 1.00 |
| Q15323 | Keratin, type I cuticular Ha1 | KRT31 | 1.55 | 1.35 | 1.13 | 0.54 | -0.89 | -1.06 | -1.17 | 0.81 | 1.35 | 0.75 | -0.61 | -0.86 |
| Q9UKV8 | Protein argonaute-2 | AGO2 | 1.55 | 2.04 | -0.76 | 0.73 | -0.96 | -0.08 | -0.89 | 1.66 | 0.70 | 1.25 | -0.85 | -0.80 |
| Q04721 | Neurogenic locus notch homolog protein 2 | NOTCH2 | 1.55 | 1.26 | -1.24 | -0.33 | -1.41 | 0.61 | 1.00 | -1.36 | 0.24 | 0.72 | 0.88 | 0.88 |
| Q9NZL3 | Zinc finger protein 224 | ZNF224 | 1.55 | 2.91 | 0.93 | -1.09 | -1.28 | -0.96 | 0.00 | -0.95 | 0.21 | 1.09 | 0.78 | 1.28 |
| P60174 | Triosephosphate isomerase | TPI1 | 1.55 | 3.34 | -0.68 | 0.02 | -0.17 | -0.43 | -1.43 | 0.07 | -0.08 | 2.48 | -0.07 | 0.29 |
| Q86XZ4 | Spermatogenesis-associated serine-rich protein 2 | SPATS2 | 1.55 | 1.71 | 0.81 | 0.85 | -1.23 | -1.06 | -1.08 | 0.97 | 0.90 | 0.84 | -1.15 | 0.15 |
| Q9Y4E8 | Ubiquitin carboxyl-terminal hydrolase 15 | USP15 | 1.55 | 1.57 | -0.92 | -0.33 | 1.24 | -0.43 | -1.17 | -1.09 | 0.07 | 1.51 | 1.22 | -0.09 |
| Q8IWJ2 | GRIP and coiled-coil domain-containing protein 2 | GCC2 | 1.55 | 1.76 | 0.92 | -0.68 | -0.83 | -0.52 | -0.66 | 0.77 | 1.87 | -0.99 | -0.72 | 0.84 |
| Q16637 | Survival motor neuron protein | SMN1 | 1.54 | 3.44 | 0.24 | -0.55 | -1.70 | 0.78 | -1.48 | 0.00 | 0.55 | 0.21 | 0.41 | 1.56 |
| Q9BWW4 | Single-stranded DNA-binding protein 3 | SSBP3 | 1.54 | 2.43 | -0.68 | -0.73 | -0.87 | 0.89 | -0.79 | -0.68 | -0.71 | 0.95 | 0.83 | 1.81 |
| P29376 | Leukocyte tyrosine kinase receptor | LTK | 1.54 | 1.72 | 1.73 | -0.87 | -0.99 | -0.78 | -0.82 | 0.80 | 1.20 | 0.40 | 0.18 | -0.85 |
| P68036 | Ubiquitin-conjugating enzyme E2 L3 | UBE2L3 | 1.54 | 6.49 | -0.01 | -1.18 | -1.60 | -0.09 | -0.70 | 0.08 | 1.59 | 1.35 | 0.21 | 0.38 |
| O15488 | Glycogenin-2 | GYG2 | 1.54 | 2.00 | -0.13 | -1.65 | -1.14 | 0.66 | 0.36 | 0.93 | -1.33 | 0.76 | 0.85 | 0.69 |
| Q7Z6J6 | FERM domain-containing protein 5 | FRMD5 | 1.53 | 2.42 | 0.67 | -0.22 | -2.13 | 0.21 | -0.70 | 1.40 | 0.94 | -0.69 | 0.32 | 0.19 |
| Q16527 | Cysteine and glycine-rich protein 2 | CSRP2 | 1.53 | 1.68 | 0.30 | 0.40 | -2.41 | 0.42 | -0.43 | 0.82 | 0.11 | -0.72 | 0.38 | 1.13 |
| Q9NUJ3 | T-complex protein 11-like protein 1 | TCP11L1 | 1.52 | 2.98 | -0.53 | 0.16 | -0.80 | -0.65 | -0.76 | 1.21 | 1.60 | -0.96 | -0.59 | 1.33 |
| P43897 | Elongation factor Ts, mitochondrial | TSFM | 1.52 | 2.02 | -0.34 | -0.73 | 1.26 | -1.00 | -1.11 | 1.13 | 1.64 | -0.75 | -0.07 | -0.03 |
| Q8IWA5 | Choline transporter-like protein 2 | SLC44A2 | 1.52 | 2.01 | -1.26 | -1.35 | 0.58 | 0.53 | -0.42 | 1.17 | -1.31 | 0.99 | 0.82 | 0.25 |
| Q14139 | Ubiquitin conjugation factor E4 A | UBE4A | 1.51 | 3.30 | -0.54 | 0.39 | -0.61 | 0.27 | -2.13 | 1.48 | -0.20 | -0.19 | 0.50 | 1.04 |
| Q6EEV6 | Small ubiquitin-related modifier 4 | SUMO4 | 1.51 | 1.36 | -0.85 | -0.95 | -1.07 | 0.77 | 0.65 | -0.89 | -0.87 | 1.46 | 0.83 | 0.91 |
| Q96EK7 | Constitutive coactivator of peroxisome proliferator-activated rece | FAM120B | 1.51 | 3.62 | 0.93 | -0.52 | -0.17 | -1.48 | -1.53 | 1.12 | -0.12 | 0.16 | 1.40 | 0.22 |
| Q99747 | Gamma-soluble NSF attachment protein | NAPG | 1.50 | 7.12 | -1.41 | -0.86 | -1.26 | -0.47 | 0.28 | 1.05 | 0.90 | 1.27 | -0.35 | 0.85 |
| P43405 | Tyrosine-protein kinase SYK | SYK | 1.50 | 1.17 | 1.08 | -0.39 | -2.01 | -0.11 | 0.14 | 1.21 | 1.20 | -0.79 | -0.04 | -0.27 |
| O15484 | Calpain-5 | CAPN5 | 1.50 | 1.23 | 1.61 | -0.66 | -0.73 | -0.76 | -0.81 | 1.32 | 1.33 | -0.62 | -0.49 | -0.19 |
| Q7Z3K3 | Pogo transposable element with ZNF domain | POGZ | 1.50 | 1.96 | 1.13 | -1.60 | -1.78 | -0.17 | 0.47 | 0.93 | 0.85 | -0.03 | -0.16 | 0.36 |
| Q9NRF8 | CTP synthase 2 | CTPS2 | 1.50 | 4.52 | 0.10 | -0.64 | 0.09 | -0.56 | -2.07 | 0.47 | 1.23 | 1.24 | -0.53 | 0.66 |
| P11171 | Protein 4.1 | EPB41 | 1.49 | 3.06 | 1.25 | -1.23 | -0.72 | -0.74 | -1.06 | 0.85 | 0.35 | 0.56 | -0.71 | 1.44 |
| Q8N126 | Cell adhesion molecule 3 | CADM3 | 1.49 | 2.53 | -0.17 | 0.94 | -2.37 | -0.15 | -0.55 | 0.27 | 1.39 | 0.35 | 0.11 | 0.17 |
| Q96FS4 | Signal-induced proliferation-associated protein 1 | SIPA1 | 1.49 | 2.11 | -0.67 | 0.75 | -0.94 | -0.21 | -0.93 | 1.00 | 1.94 | -1.19 | 0.15 | 0.11 |
| Q9H9B4 | Sideroflexin-1 | SFXN1 | 1.49 | 2.09 | -0.61 | 0.42 | -0.48 | -0.56 | -0.80 | -0.64 | 1.41 | 2.11 | -0.20 | -0.63 |
| Q32P44 | Echinoderm microtubule-associated protein-like 3 | EML3 | 1.49 | 1.91 | -0.39 | 0.60 | -0.14 | 0.42 | -2.37 | 0.76 | -0.84 | 0.79 | 0.33 | 0.83 |
| C9J069 | Apical junction component 1 homolog | AJM1 | 1.48 | 1.64 | 0.40 | -0.04 | 0.46 | -1.01 | -1.49 | 1.38 | 1.21 | -0.10 | 0.50 | -1.33 |
| Q9GZT4 | Serine racemase | SRR | 1.48 | 1.42 | 0.93 | -0.38 | -2.20 | 0.30 | -0.15 | 1.25 | 0.72 | -0.91 | 0.16 | 0.29 |
| Q9Y2D4 | Exocyst complex component 6B | EXOC6B | 1.48 | 0.77 | 1.06 | 0.10 | -0.73 | -0.69 | -0.68 | 1.23 | 1.83 | -0.84 | -0.66 | -0.62 |
| Q15274 | Nicotinate-nucleotide pyrophosphorylase [carboxylating] | QPRT | 1.48 | 1.50 | -0.24 | -0.75 | -0.10 | 0.84 | -1.33 | 0.81 | -1.28 | 1.75 | 0.65 | -0.36 |
| P21817 | Ryanodine receptor 1 | RYR1 | 1.48 | 2.47 | -0.38 | -0.44 | -0.68 | -0.44 | -0.41 | -0.51 | -0.45 | 2.25 | 1.47 | -0.41 |
| P06703 | Protein S100-A6 | S100A6 | 1.47 | 1.90 | 0.05 | 0.05 | 0.65 | -0.08 | -2.57 | 1.13 | -0.15 | 0.04 | 0.04 | 0.83 |
| A2RTX5 | Threonine--tRNA ligase 2, cytoplasmic | TARSL2 | 1.47 | 2.65 | 0.89 | -0.97 | -0.14 | 0.17 | -2.37 | 0.45 | 0.78 | 0.85 | 0.09 | 0.25 |
| Q15057 | Arf-GAP with coiled-coil, ANK repeat and PH domain-containing p | ACAP2 | 1.47 | 2.14 | -0.16 | 0.53 | -2.60 | 0.57 | -0.44 | 0.18 | 0.02 | 0.33 | 0.93 | 0.63 |
| O00244 | Copper transport protein ATOX1 | ATOX1 | 1.46 | 1.94 | -0.84 | 0.41 | -0.77 | -0.88 | 0.17 | 0.35 | -1.02 | 2.32 | -0.16 | 0.43 |
| P42126 | Enoyl-CoA delta isomerase 1, mitochondrial | ECI1 | 1.46 | 2.52 | -0.57 | -0.77 | 0.38 | -0.67 | -0.67 | -0.47 | -0.53 | 2.47 | 0.24 | 0.60 |
| P01619 | Immunoglobulin kappa variable 3-20 | IGKV3-20 | 1.46 | 1.46 | 0.95 | -1.65 | 0.51 | -1.65 | 0.28 | 0.96 | -0.26 | 1.01 | -0.46 | 0.31 |
| P50416 | Carnitine O-palmitoyltransferase 1, liver isoform | CPT1A | 1.46 | 2.60 | -2.39 | 0.01 | 0.48 | -0.60 | 0.19 | 1.09 | -0.06 | 0.00 | 0.07 | 1.21 |
| Q8IX04 | Ubiquitin-conjugating enzyme E2 variant 3 | UEVLD | 1.46 | 5.69 | -0.33 | -0.63 | -0.66 | 0.24 | -2.13 | 1.21 | 0.30 | 0.44 | 0.28 | 1.28 |
| Q03113 | Guanine nucleotide-binding protein subunit alpha-12 | GNA12 | 1.46 | 2.05 | -0.36 | -0.28 | 0.45 | -0.37 | -1.41 | 1.33 | 1.22 | -1.58 | 0.19 | 0.81 |
| Q9UFN0 | Protein NipSnap homolog 3A | NIPSNAP3A | 1.46 | 1.42 | -0.96 | -1.17 | 1.22 | -0.71 | 0.13 | 1.05 | -0.66 | 1.70 | -0.16 | -0.43 |
| P04843 | Dolichyl-diphosphooligosaccharide--protein glycosyltransferase sul | RPN1 | 1.45 | 2.14 | -0.25 | -0.41 | -0.64 | -0.32 | -0.50 | 1.89 | -0.41 | -0.79 | -0.42 | 1.86 |
| P09668 | Pro-cathepsin H | CTSH | 1.44 | 2.33 | -0.24 | -0.38 | -0.59 | -0.50 | -0.55 | -0.39 | -0.48 | 2.50 | -0.41 | 1.05 |
| P31937 | 3-hydroxyisobutyrate dehydrogenase, mitochondrial | HIBADH | 1.44 | 1.77 | -0.48 | 0.89 | -0.87 | -0.57 | -0.74 | -0.59 | 0.17 | 2.29 | -0.64 | 0.55 |
| Q08345 | Epithelial discoidin domain-containing receptor 1 | DDR1 | 1.44 | 1.59 | 1.01 | -1.17 | -1.35 | 0.45 | -0.57 | 1.52 | -1.13 | 0.42 | 0.14 | 0.67 |
| Q99707 | Methionine synthase | MTR | 1.44 | 4.16 | -0.85 | 0.41 | -2.24 | -0.12 | -0.23 | 0.30 | 0.59 | 1.51 | 0.55 | 0.09 |
| Q9Y5P4 | Ceramide transfer protein | CERT | 1.44 | 3.44 | 0.48 | -0.82 | -2.41 | 0.39 | -0.52 | 0.40 | 0.75 | 0.78 | 0.29 | 0.66 |
| P55001 | Microfibrillar-associated protein 2 | MFAP2 | 1.44 | 1.75 | 0.46 | -1.08 | 1.08 | -1.05 | -1.15 | 0.69 | 1.14 | 0.07 | -1.14 | 0.99 |
| P63098 | Calcineurin subunit B type 1 | PPP3R1 | 1.43 | 1.52 | 0.91 | -1.10 | -1.27 | -1.04 | 0.92 | 1.22 | -0.14 | 1.22 | -0.64 | -0.09 |
| P61626 | Lysozyme C | LYZ | 1.43 | 1.81 | -0.65 | -0.83 | -0.96 | -0.65 | 1.30 | -0.77 | -0.73 | 1.34 | 0.82 | 1.12 |

| | | | | | | | | | | | | | | |
|---|---|---|---|---|---|---|---|---|---|---|---|---|---|---|
| Q13867 | Bleomycin hydrolase | BLMH | 1.42 | 2.04 | -0.52 | -1.24 | -0.61 | 0.09 | 0.30 | -0.16 | -0.98 | 2.33 | 0.36 | 0.43 |
| P56537 | Eukaryotic translation initiation factor 6 | EIF6 | 1.42 | 2.05 | 0.40 | -0.45 | -1.13 | -0.91 | 0.12 | 1.09 | -0.83 | 1.04 | -0.95 | 1.63 |
| P02788 | Lactotransferrin | LTF | 1.41 | 4.61 | -0.87 | 0.10 | -1.10 | -0.52 | -0.84 | 1.01 | 1.93 | 0.92 | 0.04 | -0.67 |
| Q9UIA9 | Exportin-7 | XPO7 | 1.41 | 5.40 | -0.36 | -0.66 | -0.87 | -0.03 | -1.49 | 0.08 | 1.31 | 1.85 | -0.31 | 0.48 |
| Q15417 | Calponin-3 | CNN3 | 1.41 | 5.92 | 0.14 | -1.41 | -0.80 | -0.34 | -1.08 | 1.30 | 0.26 | -0.47 | 0.87 | 1.53 |
| P78559 | Microtubule-associated protein 1A | MAP1A | 1.41 | 2.66 | -0.26 | -0.61 | -0.65 | -0.29 | -0.65 | -1.19 | 0.97 | 1.72 | -0.49 | 1.45 |
| Q8WWH5 | Probable tRNA pseudouridine synthase 1 | TRUB1 | 1.41 | 1.76 | -1.46 | -1.59 | -0.26 | 0.30 | 1.24 | 0.05 | -0.63 | 0.90 | 0.26 | 1.18 |
| Q14203 | Dynactin subunit 1 | DCTN1 | 1.41 | 2.10 | -1.15 | -0.15 | -0.35 | -0.44 | 0.03 | -0.65 | -0.16 | 0.83 | -0.43 | 2.46 |
| Q16666 | Gamma-interferon-inducible protein 16 | IFI16 | 1.41 | 1.35 | 0.91 | -2.71 | -0.09 | 0.33 | 0.05 | 0.49 | 0.61 | 0.15 | 0.26 | 0.01 |
| Q15459 | Splicing factor 3A subunit 1 | SF3A1 | 1.41 | 2.81 | -0.18 | -0.69 | -2.15 | -0.30 | 0.93 | 1.53 | 0.60 | -0.11 | -0.03 | 0.42 |
| A6NDG6 | Glycerol-3-phosphate phosphatase | PGP | 1.40 | 2.53 | 1.14 | -1.47 | -0.08 | -0.27 | -1.57 | 0.34 | 0.08 | 1.63 | -0.22 | 0.42 |
| Q8NBR6 | Ubiquitin carboxyl-terminal hydrolase MINDY-2 | MINDY2 | 1.40 | 4.75 | -0.07 | -0.27 | -2.12 | 0.23 | -0.98 | 1.17 | 0.28 | 0.08 | 0.31 | 1.37 |
| Q8TD19 | Serine/threonine-protein kinase Nek9 | NEK9 | 1.40 | 2.93 | -0.63 | 0.79 | -1.87 | 0.10 | -0.83 | 1.30 | 1.03 | -0.73 | 0.13 | 0.70 |
| Q9NPB8 | Glycerophosphocholine phosphodiesterase GPCPD1 | GPCPD1 | 1.40 | 2.46 | 0.90 | -1.71 | 0.11 | -0.30 | -1.17 | 0.88 | 1.57 | -0.25 | -0.48 | 0.47 |
| P20472 | Parvalbumin alpha | PVALB | 1.40 | 1.73 | -0.32 | 0.03 | -0.59 | -0.47 | -0.46 | -0.50 | -0.41 | 2.68 | -0.48 | 0.53 |
| Q9NR19 | Acetyl-coenzyme A synthetase, cytoplasmic | ACSS2 | 1.39 | 1.95 | -0.59 | 0.24 | -2.35 | 0.47 | 0.32 | 0.39 | -0.51 | 1.46 | 0.46 | 0.10 |
| Q9UNS2 | COP9 signalosome complex subunit 3 | COPS3 | 1.39 | 1.94 | 1.27 | -0.71 | -0.86 | -0.76 | -0.82 | -0.72 | 1.26 | 1.30 | -0.73 | 0.77 |
| P35219 | Carbonic anhydrase-related protein | CA8 | 1.38 | 2.19 | -0.65 | -0.71 | -1.00 | 1.05 | -0.71 | -0.75 | -0.77 | 1.00 | 1.14 | 1.41 |
| P06702 | Protein S100-A9 | S100A9 | 1.38 | 1.91 | 0.95 | -0.37 | -2.02 | -0.89 | 0.46 | 0.31 | 0.43 | 1.42 | -0.66 | 0.37 |
| P00505 | Aspartate aminotransferase, mitochondrial | GOT2 | 1.38 | 1.63 | -1.08 | 0.41 | 0.22 | -0.86 | -0.37 | -0.44 | -0.89 | 2.13 | -0.20 | 1.09 |
| Q9Y216 | Myotubularin-related protein 7 | MTMR7 | 1.38 | 1.95 | -1.32 | 0.36 | -0.06 | 1.12 | -2.04 | -0.23 | 0.59 | -0.03 | 0.86 | 0.76 |
| Q99613 | Eukaryotic translation initiation factor 3 subunit C | EIF3C | 1.38 | 2.85 | 0.66 | -0.65 | -1.46 | -0.44 | -0.52 | 1.02 | 1.82 | -0.74 | -0.38 | 0.69 |
| O43660 | Pleiotropic regulator 1 | PLRG1 | 1.38 | 1.58 | -0.46 | -1.52 | -0.63 | 0.31 | 0.67 | -0.37 | 2.12 | -0.28 | 0.72 | -0.57 |
| P00492 | Hypoxanthine-guanine phosphoribosyltransferase | HPRT1 | 1.37 | 2.11 | -0.66 | -0.48 | -0.20 | -0.25 | -0.53 | -0.83 | 0.87 | 2.53 | -0.14 | -0.33 |
| P40763 | Signal transducer and activator of transcription 3 | STAT3 | 1.37 | 1.34 | 0.69 | -0.22 | -2.58 | 0.22 | 0.40 | 0.97 | 0.60 | -0.47 | 0.11 | 0.27 |
| P09234 | U1 small nuclear ribonucleoprotein C | SNRPC | 1.37 | 1.11 | -0.94 | -1.03 | 0.31 | 0.51 | -0.11 | -0.99 | -1.02 | 0.24 | 1.47 | 1.57 |
| P49354 | Protein farnesyltransferase/geranylgeranyltransferase type-1 subu | FNTA | 1.37 | 6.64 | -0.42 | -0.91 | -1.03 | -0.74 | -0.84 | 2.11 | 0.01 | 0.61 | 0.32 | 0.88 |
| Q5QJ74 | Tubulin-specific chaperone cofactor E-like protein | TBCEL | 1.37 | 2.43 | 1.01 | -2.13 | -0.90 | 0.01 | -0.20 | 1.43 | -0.25 | 0.26 | 0.18 | 0.60 |
| P0DOX2 | Immunoglobulin alpha-2 heavy chain | Immunoglobulin | 1.37 | 2.75 | -1.92 | 0.41 | -0.48 | 0.15 | -0.49 | 2.03 | -0.38 | 0.58 | -0.13 | 0.25 |
| Q5VWQ8 | Disabled homolog 2-interacting protein | DAB2IP | 1.37 | 2.45 | -0.02 | 0.20 | -0.34 | -0.04 | -1.98 | 1.72 | 1.11 | -0.79 | -0.14 | 0.27 |
| P47897 | Glutamine--tRNA ligase | QARS | 1.36 | 3.80 | 0.40 | -0.83 | 0.00 | -0.19 | -2.27 | 1.32 | 0.38 | 0.64 | -0.23 | 0.77 |
| Q14515 | SPARC-like protein 1 | SPARCL1 | 1.36 | 1.13 | 1.35 | -0.55 | -1.10 | -0.78 | -0.17 | 1.37 | 1.12 | 0.53 | -1.08 | -0.68 |
| P28370 | Probable global transcription activator SNF2L1 | SMARCA1 | 1.36 | 3.35 | 0.15 | -1.52 | -1.18 | -0.24 | 0.16 | 0.58 | 2.16 | 0.28 | -0.33 | -0.06 |
| P49137 | MAP kinase-activated protein kinase 2 | MAPKAPK2 | 1.36 | 1.44 | 1.09 | -1.03 | -1.16 | 0.63 | -1.05 | 1.32 | 0.49 | -1.20 | 0.25 | 0.65 |
| P08754 | Guanine nucleotide-binding protein G(i) subunit alpha | GNAI3 | 1.35 | 3.02 | -0.17 | 0.24 | -0.70 | 0.37 | -2.23 | 1.15 | -0.22 | -0.35 | 0.84 | 1.07 |
| Q96QK1 | Vacuolar protein sorting-associated protein 35 | VPS35 | 1.35 | 3.30 | -1.04 | 0.55 | 0.06 | -0.36 | -1.81 | 1.13 | 1.44 | -0.67 | 0.04 | 0.66 |
| P04040 | Catalase | CAT | 1.35 | 3.64 | 0.30 | -1.52 | 0.27 | -0.79 | -1.00 | 0.90 | 0.03 | 1.99 | -0.32 | 0.15 |
| O60271 | C-Jun-amino-terminal kinase-interacting protein 4 | SPAG9 | 1.34 | 0.67 | 1.39 | -0.15 | -0.74 | -0.63 | -0.69 | 1.22 | 1.66 | -0.78 | -0.62 | -0.65 |
| P12111 | Collagen alpha-3(VI) chain | COL6A3 | 1.34 | 3.31 | -0.33 | -0.44 | 0.61 | -1.21 | -1.24 | 0.72 | 1.95 | -0.28 | -0.56 | 0.78 |
| P26038 | Moesin | MSN | 1.34 | 4.95 | 0.07 | -1.40 | 0.02 | -0.23 | -1.66 | 1.93 | 0.15 | 0.39 | 0.20 | 0.53 |
| Q68DK2 | Zinc finger FYVE domain-containing protein 26 | ZFYVE26 | 1.34 | 1.96 | -0.56 | -0.70 | -0.77 | -0.59 | 0.69 | 1.75 | -0.74 | -1.08 | 0.92 | 1.07 |
| P01042 | Kininogen-1 | KNG1 | 1.33 | 1.88 | 0.95 | -1.72 | 0.89 | -0.52 | -1.48 | 0.70 | 0.79 | 0.23 | -0.51 | 0.67 |
| Q9P286 | Serine/threonine-protein kinase PAK 5 | PAK5 | 1.33 | 3.02 | 0.23 | -0.63 | -2.48 | 0.25 | 0.07 | 0.66 | 0.89 | 0.94 | 0.36 | -0.29 |
| P25325 | 3-mercaptopyruvate sulfurtransferase | MPST | 1.33 | 3.94 | 0.31 | -1.03 | -0.04 | -0.20 | -1.89 | 0.93 | 0.28 | 1.51 | 0.76 | -0.63 |
| Q9NQ48 | Leucine zipper transcription factor-like protein 1 | LZTFL1 | 1.33 | 2.84 | -0.70 | -0.53 | -0.48 | 0.17 | -0.95 | 0.34 | 2.58 | -0.32 | 0.22 | -0.33 |
| Q15049 | Membrane protein MLC1 | MLC1 | 1.33 | 4.23 | -0.34 | -1.51 | -0.38 | -0.17 | -0.67 | 0.86 | -0.96 | 1.75 | 0.36 | 1.05 |
| O75131 | Copine-3 | CPNE3 | 1.33 | 1.86 | 0.17 | -1.25 | -0.56 | 0.21 | -0.42 | 1.78 | -1.57 | 0.09 | 0.65 | 0.90 |
| P11217 | Glycogen phosphorylase, muscle form | PYGM | 1.32 | 3.90 | -0.21 | -1.20 | -0.21 | -0.24 | -1.09 | 1.94 | 0.39 | -0.42 | -0.37 | 1.41 |
| P48163 | NADP-dependent malic enzyme | ME1 | 1.32 | 4.38 | -0.43 | -1.66 | -0.02 | -0.89 | -0.04 | 1.10 | 0.77 | 1.69 | -0.74 | 0.23 |
| P54920 | Alpha-soluble NSF attachment protein | NAPA | 1.32 | 4.60 | -2.33 | -0.74 | -0.52 | 0.10 | 0.20 | 1.01 | 0.89 | 0.14 | 0.46 | 0.81 |
| Q4G0X4 | BTB/POZ domain-containing protein KCTD21 | KCTD21 | 1.32 | 2.25 | -0.39 | -0.63 | -0.90 | 0.52 | -0.72 | 1.87 | -0.70 | -0.99 | 0.88 | 1.05 |
| O94760 | N(G),N(G)-dimethylarginine dimethylaminohydrolase 1 | DDAH1 | 1.32 | 3.71 | -0.18 | -1.09 | -1.64 | -0.65 | 0.78 | 0.51 | 0.95 | 1.67 | -0.36 | 0.01 |
| P09525 | Annexin A4 | ANXA4 | 1.32 | 2.12 | -0.07 | -0.07 | 0.04 | -0.57 | -1.36 | 0.51 | -1.28 | 1.58 | -0.30 | 1.52 |
| Q13491 | Neuronal membrane glycoprotein M6-b | GPM6B | 1.31 | 3.05 | -0.43 | -1.04 | -0.98 | 0.80 | -0.85 | -0.16 | -0.45 | 1.83 | 1.36 | -0.08 |
| Q9HD42 | Charged multivesicular body protein 1a | CHMP1A | 1.31 | 2.59 | 1.38 | -0.18 | -1.31 | -1.81 | -0.44 | 0.50 | 0.60 | 1.09 | 0.22 | -0.05 |
| O95825 | Quinone oxidoreductase-like protein 1 | CRYZL1 | 1.31 | 0.85 | 1.49 | -1.09 | -0.10 | -0.20 | -1.12 | 1.15 | 1.45 | -0.51 | -0.47 | -0.61 |
| P35240 | Merlin | NF2 | 1.31 | 2.73 | -0.33 | 0.58 | -1.60 | 0.48 | -1.46 | 1.25 | 0.62 | -0.88 | 0.62 | 0.72 |
| P47895 | Aldehyde dehydrogenase family 1 member A3 | ALDH1A3 | 1.30 | 2.85 | -0.35 | -0.40 | -0.81 | -0.42 | -0.60 | 0.91 | 0.15 | 2.49 | -0.58 | -0.40 |
| Q16816 | Phosphorylase b kinase gamma catalytic chain, skeletal muscle/h | PHKG1 | 1.30 | 2.54 | 0.22 | -0.07 | -2.68 | 0.43 | -0.25 | 0.97 | 0.39 | 0.13 | 0.52 | 0.36 |
| Q9H223 | EH domain-containing protein 4 | EHD4 | 1.30 | 3.59 | -0.15 | 0.89 | -1.28 | -0.46 | -1.78 | 1.24 | -0.03 | -0.02 | 0.35 | 1.24 |
| Q96NY7 | Chloride intracellular channel protein 6 | CLIC6 | 1.30 | 1.03 | 0.49 | 0.46 | -0.64 | -0.94 | -0.57 | 0.04 | 0.61 | -0.69 | -1.01 | 2.25 |
| Q6IAN0 | Dehydrogenase/reductase SDR family member 7B | DHRS7B | 1.30 | 2.17 | -0.33 | -0.46 | -0.61 | -0.35 | -0.41 | -0.34 | -0.35 | -0.85 | 1.51 | 2.19 |
| Q9NZU5 | LIM and cysteine-rich domains protein 1 | LMCD1 | 1.30 | 2.31 | -1.06 | 0.36 | -1.51 | 0.46 | -0.34 | 1.02 | -1.39 | 0.64 | 0.70 | 1.13 |
| P12271 | Retinaldehyde-binding protein 1 | RLBP1 | 1.29 | 3.08 | -0.49 | 0.19 | -2.51 | 0.60 | -0.44 | 0.84 | 0.56 | 0.16 | 0.22 | 0.88 |
| Q14152 | Eukaryotic translation initiation factor 3 subunit A | EIF3A | 1.29 | 3.57 | -0.78 | -0.87 | -0.50 | -0.54 | -0.24 | 2.10 | 0.74 | -0.62 | -0.49 | 1.20 |
| Q8NDH3 | Probable aminopeptidase NPEPL1 | NPEPL1 | 1.29 | 2.30 | 0.68 | -2.45 | -0.29 | -0.09 | 0.01 | 0.54 | 1.13 | 0.84 | 0.04 | -0.41 |
| P48539 | Calmodulin regulator protein PCP4 | PCP4 | 1.29 | 2.66 | -0.31 | -0.43 | -0.63 | -0.54 | -0.57 | -0.44 | -0.41 | 2.14 | -0.43 | 1.61 |
| Q8NFZ4 | Neuroligin-2 | NLGN2 | 1.29 | 0.99 | 1.66 | -0.39 | -1.18 | -0.10 | -1.14 | 1.29 | 1.06 | -0.16 | -0.36 | -0.68 |
| P27986 | Phosphatidylinositol 3-kinase regulatory subunit alpha | PIK3R1 | 1.29 | 3.34 | -0.14 | -1.13 | -1.17 | -0.60 | 0.38 | 1.13 | 1.83 | 0.02 | 0.59 | -0.92 |
| Q01433 | AMP deaminase 2 | AMPD2 | 1.29 | 2.03 | 0.78 | 0.32 | -1.03 | -0.55 | -1.45 | 0.57 | 0.82 | 1.63 | -1.08 | -0.02 |
| Q9NSY1 | BMP-2-inducible protein kinase | BMP2K | 1.28 | 2.18 | 0.29 | 0.69 | -0.93 | 0.06 | -2.15 | 1.08 | 0.10 | -0.59 | 0.30 | 1.16 |
| Q9NUY8 | TBC1 domain family member 23 | TBC1D23 | 1.28 | 1.49 | -0.60 | 0.10 | -1.25 | -0.32 | 0.49 | 1.60 | 0.93 | -1.63 | 0.79 | -0.11 |
| O76071 | Probable cytosolic iron-sulfur protein assembly protein CIAO1 | CIAO1 | 1.28 | 1.89 | 0.62 | -0.02 | -2.42 | 0.42 | -0.46 | 0.47 | 0.49 | -0.70 | 0.61 | 1.01 |
| Q8N145 | Leucine-rich repeat LGI family member 3 | LGI3 | 1.28 | 1.09 | -1.83 | -0.32 | 0.98 | 0.63 | -0.70 | -1.29 | 0.08 | 0.97 | 0.74 | 0.74 |
| P13716 | Delta-aminolevulinic acid dehydratase | ALAD | 1.28 | 3.61 | -0.73 | -1.17 | -0.09 | -0.47 | -0.43 | 1.02 | -0.95 | 2.11 | 0.51 | 0.21 |
| Q9Y6G9 | Cytoplasmic dynein 1 light intermediate chain 1 | DYNC1LI1 | 1.28 | 3.21 | -0.93 | -0.10 | -0.15 | -0.98 | -0.53 | 0.48 | 1.87 | -0.23 | -0.92 | 1.50 |
| Q9Y365 | START domain-containing protein 10 | STARD10 | 1.28 | 1.11 | 0.65 | -0.57 | -1.16 | 0.09 | -0.28 | 1.71 | 1.31 | -1.46 | -0.06 | -0.23 |
| Q5SGD2 | Protein phosphatase 1L | PPM1L | 1.27 | 2.26 | 0.58 | -0.76 | -2.61 | 0.31 | 0.26 | 0.56 | 0.16 | 0.61 | 0.52 | 0.35 |
| Q96D46 | 60S ribosomal export protein NMD3 | NMD3 | 1.27 | 2.54 | 0.46 | -0.34 | -2.66 | 0.07 | 0.11 | 0.84 | 0.18 | 0.05 | 0.63 | 0.65 |
| Q92945 | Far upstream element-binding protein 2 | KHSRP | 1.27 | 2.79 | -0.17 | 0.72 | -2.06 | -0.15 | -0.71 | 0.51 | -0.09 | 1.65 | -0.42 | 0.72 |
| P0DP03 | Immunoglobulin heavy variable 3-30-5 | IGHV3-30-5 | 1.27 | 1.17 | -0.99 | -1.05 | 0.57 | 0.45 | -0.28 | -1.03 | -1.04 | 1.57 | 0.76 | 1.05 |
| Q9Y696 | Chloride intracellular channel protein 4 | CLIC4 | 1.27 | 3.14 | -1.19 | -0.79 | 0.14 | 0.73 | -1.41 | 1.61 | -0.67 | -0.07 | 0.80 | 0.87 |
| O00471 | Exocyst complex component 5 | EXOC5 | 1.27 | 1.59 | -1.14 | 0.30 | -1.36 | 0.71 | -0.14 | 0.35 | 1.03 | -1.53 | 0.63 | 1.14 |

| | | | | | | | | | | | | | | |
|---|---|---|---|---|---|---|---|---|---|---|---|---|---|---|
| P06396 | Gelsolin | GSN | 1.26 | 3.14 | -0.75 | 0.40 | -0.29 | -0.04 | -1.84 | 2.00 | 0.03 | -0.36 | 0.04 | 0.82 |
| A0JNW5 | UHRF1-binding protein 1-like | UHRF1BP1L | 1.26 | 1.67 | 0.60 | 0.05 | -2.18 | 0.67 | -0.85 | -0.34 | 0.78 | -0.38 | 0.34 | 1.31 |
| Q96DB2 | Histone deacetylase 11 | HDAC11 | 1.26 | 0.78 | 0.72 | -0.38 | -0.51 | -0.40 | -0.39 | -0.37 | 2.65 | -0.57 | -0.40 | -0.35 |
| Q9H4A3 | Serine/threonine-protein kinase WNK1 | WNK1 | 1.26 | 1.36 | 1.54 | -1.12 | -0.91 | -0.36 | -0.60 | 1.37 | 1.26 | -0.23 | -0.25 | -0.69 |
| O75925 | E3 SUMO-protein ligase PIAS1 | PIAS1 | 1.25 | 1.93 | 0.67 | -1.79 | -0.30 | -1.05 | 0.62 | 1.43 | -0.99 | 0.67 | 0.36 | 0.38 |
| Q5JSZ5 | Protein PRRC2B | PRRC2B | 1.25 | 1.22 | 1.20 | -1.02 | -0.79 | -0.63 | -0.10 | 1.19 | 0.11 | -0.91 | -0.71 | 1.66 |
| Q7KZF4 | Staphylococcal nuclease domain-containing protein 1 | SND1 | 1.25 | 2.58 | -1.27 | -0.17 | -0.09 | -0.57 | -0.24 | 0.39 | -0.50 | 2.51 | -0.39 | 0.33 |
| P42566 | Epidermal growth factor receptor substrate 15 | EPS15 | 1.25 | 2.05 | 0.12 | -0.20 | -2.67 | 0.24 | 0.49 | 0.76 | 0.02 | -0.06 | 0.86 | 0.44 |
| Q9BST9 | Rhotekin | RTKN | 1.24 | 1.59 | -1.10 | 0.52 | -1.44 | 0.22 | 0.17 | 0.35 | 1.03 | -1.53 | 0.51 | 1.27 |
| O00267 | Transcription elongation factor SPT5 | SUPT5H | 1.24 | 1.19 | 0.87 | -1.79 | 0.27 | -1.28 | 0.61 | 1.14 | 0.33 | 0.60 | -1.05 | 0.31 |
| Q16513 | Serine/threonine-protein kinase N2 | PKN2 | 1.24 | 1.91 | 0.70 | -0.73 | -0.84 | -0.25 | -0.76 | 0.68 | -0.62 | -1.10 | 1.05 | 1.86 |
| Q9Y678 | Coatomer subunit gamma-1 | COPG1 | 1.24 | 2.56 | -0.75 | -0.15 | -0.71 | 0.00 | -0.76 | 0.81 | 2.10 | -1.22 | -0.20 | 0.86 |
| O95248 | Myotubularin-related protein 5 | SBF1 | 1.24 | 1.71 | 0.87 | -0.50 | -0.80 | -0.65 | -0.66 | 1.06 | 2.13 | -0.78 | -0.42 | -0.25 |
| Q9ULL4 | Plexin-B3 | PLXNB3 | 1.24 | 3.43 | -0.33 | -0.63 | -0.87 | -0.46 | -0.59 | 1.50 | -0.56 | 1.83 | -0.74 | 0.85 |
| P28331 | NADH-ubiquinone oxidoreductase 75 kDa subunit, mitochondrial | NDUFS1 | 1.24 | 1.03 | 0.89 | -0.70 | 0.10 | -0.92 | -0.56 | 1.70 | 1.28 | -1.13 | -0.85 | 0.19 |
| Q96DR7 | Rho guanine nucleotide exchange factor 26 | ARHGEF26 | 1.23 | 2.70 | -0.16 | -1.07 | -1.36 | 0.60 | -0.32 | 1.57 | -1.08 | 1.15 | 0.73 | -0.05 |
| Q9Y2E4 | Disco-interacting protein 2 homolog C | DIP2C | 1.23 | 2.83 | 0.45 | 0.27 | -2.41 | -0.13 | -0.62 | 1.38 | 0.71 | 0.24 | 0.18 | -0.06 |
| Q08945 | FACT complex subunit SSRP1 | SSRP1 | 1.23 | 1.33 | 1.53 | -1.25 | -0.11 | -0.85 | -0.76 | 1.52 | -0.48 | 0.70 | -0.76 | 0.45 |
| P61326 | Protein mago nashi homolog | MAGOH | 1.23 | 2.97 | 0.51 | -1.85 | -1.56 | 0.46 | -0.07 | 0.17 | -0.38 | 1.01 | 0.88 | 0.84 |
| Q16656 | Nuclear respiratory factor 1 | NRF1 | 1.23 | 1.61 | -0.84 | -0.97 | -1.14 | 0.16 | 1.16 | -1.10 | -0.29 | 0.54 | 1.29 | 1.20 |
| P51153 | Ras-related protein Rab-13 | RAB13 | 1.23 | 2.26 | -0.61 | 0.85 | -0.97 | -0.77 | -0.59 | 0.92 | 0.05 | -1.37 | 0.92 | 1.56 |
| Q9UL18 | Protein argonaute-1 | AGO1 | 1.22 | 1.00 | -1.30 | -0.18 | 0.31 | -0.05 | 0.06 | -1.75 | 0.38 | 1.95 | 0.27 | 0.33 |
| Q96HH9 | GRAM domain-containing protein 2B | GRAMD2B | 1.22 | 1.14 | -0.61 | -1.53 | -0.75 | 0.92 | 0.70 | 0.84 | -0.72 | -0.93 | 0.80 | 1.28 |
| Q96D96 | Voltage-gated hydrogen channel 1 | HVCN1 | 1.22 | 1.72 | 0.63 | -0.68 | -0.36 | -0.69 | -0.68 | 0.98 | -0.65 | -1.01 | 0.31 | 2.14 |
| Q16650 | T-box brain protein 1 | TBR1 | 1.22 | 1.43 | 1.18 | -0.43 | -0.98 | -0.59 | -0.68 | 1.11 | 1.46 | 0.74 | -0.67 | -1.13 |
| O14558 | Heat shock protein beta-6 | HSPB6 | 1.22 | 1.86 | -0.17 | 0.47 | -1.33 | -0.13 | -0.68 | 1.97 | -1.05 | -0.45 | 0.26 | 1.11 |
| Q06187 | Tyrosine-protein kinase BTK | BTK | 1.22 | 2.19 | -1.41 | 0.16 | -0.51 | -0.76 | 0.48 | 2.16 | 0.80 | -0.05 | -0.11 | -0.76 |
| Q08AF3 | Schlafen family member 5 | SLFN5 | 1.21 | 1.55 | 1.28 | -0.02 | -1.03 | -0.89 | -0.93 | 0.92 | 1.45 | -0.43 | -1.02 | 0.68 |
| P34931 | Heat shock 70 kDa protein 1-like | HSPA1L | 1.21 | 1.34 | -0.20 | -0.25 | -0.46 | -0.24 | -0.35 | -0.20 | -0.25 | -0.56 | -0.31 | 2.83 |
| Q9P0S3 | ORM1-like protein 1 | ORMDL1 | 1.21 | 1.24 | -0.61 | 1.49 | -0.86 | -0.62 | -0.76 | -0.71 | 0.62 | -0.96 | 1.12 | 1.29 |
| Q5T9C2 | Protein FAM102A | FAM102A | 1.21 | 1.75 | 0.38 | 0.20 | -1.24 | 0.02 | -1.11 | 1.67 | 1.15 | -1.37 | 0.04 | 0.26 |
| Q96FZ7 | Charged multivesicular body protein 6 | CHMP6 | 1.20 | 1.41 | 1.39 | -0.76 | -0.99 | -0.36 | -0.77 | 0.66 | 1.77 | -1.00 | -0.28 | 0.33 |
| P67936 | Tropomyosin alpha-4 chain | TPM4 | 1.20 | 2.64 | -1.66 | 0.57 | -0.61 | -0.03 | -0.55 | 0.11 | 1.79 | 1.27 | -0.31 | -0.58 |
| O43776 | Asparagine--tRNA ligase, cytoplasmic | NARS | 1.20 | 4.74 | 0.19 | -2.03 | -0.80 | 0.39 | -1.03 | 1.13 | 1.11 | 0.08 | 0.55 | 0.41 |
| O43175 | D-3-phosphoglycerate dehydrogenase | PHGDH | 1.20 | 4.44 | -0.78 | -0.74 | -0.45 | 0.20 | -1.35 | 2.07 | -0.41 | -0.14 | 0.67 | 0.93 |
| O43929 | Origin recognition complex subunit 4 | ORC4 | 1.20 | 1.77 | 0.79 | -1.04 | 0.75 | -1.12 | -1.13 | 0.38 | -0.09 | 1.01 | 1.44 | -0.98 |
| Q9H0S4 | Probable ATP-dependent RNA helicase DDX47 | DDX47 | 1.20 | 2.64 | 1.00 | -0.70 | -1.07 | -0.71 | -0.81 | 0.25 | 2.01 | 0.54 | -0.86 | 0.35 |
| Q14353 | Guanidinoacetate N-methyltransferase | GAMT | 1.19 | 2.75 | 0.69 | -1.45 | -0.66 | 0.53 | -1.45 | 1.30 | 0.14 | -0.72 | 0.65 | 0.98 |
| O95864 | Acyl-CoA 6-desaturase | FADS2 | 1.19 | 1.84 | 0.71 | -1.46 | -1.63 | 1.02 | -0.48 | 0.82 | 0.42 | -0.43 | 1.18 | -0.16 |
| Q96DF8 | Splicing factor ESS-2 homolog | ESS2 | 1.19 | 2.65 | -0.23 | -0.50 | -0.72 | -0.55 | -0.46 | -0.53 | -0.42 | 1.93 | 1.83 | -0.36 |
| Q86TI2 | Dipeptidyl peptidase 9 | DPP9 | 1.19 | 1.40 | 1.15 | -0.81 | -0.65 | -0.25 | -0.94 | 1.69 | 1.35 | -0.22 | -0.54 | -0.79 |
| Q9UK22 | F-box only protein 2 | FBXO2 | 1.19 | 3.05 | -0.73 | -0.53 | -0.40 | 0.36 | -1.26 | 1.65 | 0.58 | -1.24 | 0.28 | 1.28 |
| Q12846 | Syntaxin-4 | STX4 | 1.19 | 1.92 | 0.29 | 0.37 | -0.48 | -0.04 | -1.99 | 1.27 | 0.69 | -1.32 | 0.59 | 0.63 |
| Q96CN7 | Isochorismatase domain-containing protein 1 | ISOC1 | 1.18 | 1.24 | -0.57 | -0.68 | 0.53 | 0.04 | -0.71 | -0.73 | -0.69 | 2.50 | 0.39 | -0.08 |
| Q15428 | Splicing factor 3A subunit 2 | SF3A2 | 1.18 | 2.03 | 1.43 | -0.90 | -1.55 | -0.20 | -0.72 | 1.02 | 1.31 | -0.62 | 0.05 | 0.18 |
| Q86U86 | Protein polybromo-1 | PBRM1 | 1.18 | 2.21 | 0.06 | -1.13 | -1.20 | -0.99 | 1.23 | 0.50 | 0.53 | 1.25 | 0.79 | -1.03 |
| Q7Z6B0 | Coiled-coil domain-containing protein 91 | CCDC91 | 1.18 | 2.33 | 1.03 | -0.95 | -0.61 | -1.14 | -0.45 | 1.40 | 1.19 | -0.88 | -0.48 | 0.89 |
| P62304 | Small nuclear ribonucleoprotein E | SNRPE | 1.18 | 1.18 | -1.33 | 0.53 | 0.55 | 0.41 | -1.46 | -1.43 | 0.33 | 0.75 | 0.47 | 1.19 |
| Q16537 | Serine/threonine-protein phosphatase 2A 56 kDa regulatory subuni | PPP2R5E | 1.18 | 1.93 | -1.00 | -0.72 | -0.56 | -1.09 | 1.51 | -1.06 | 0.43 | 1.27 | 0.58 | 0.65 |
| O15160 | DNA-directed RNA polymerases I and III subunit RPAC1 | POLR1C | 1.18 | 1.78 | 1.20 | -1.45 | -1.78 | 0.56 | -0.33 | -0.38 | 0.60 | 1.07 | 0.45 | 0.07 |
| Q99536 | Synaptic vesicle membrane protein VAT-1 homolog | VAT1 | 1.18 | 2.38 | 1.03 | -1.51 | 0.14 | -0.27 | -1.55 | 1.19 | 1.30 | -0.20 | -0.01 | -0.13 |
| Q13107 | Ubiquitin carboxyl-terminal hydrolase 4 | USP4 | 1.17 | 1.61 | 0.24 | -0.90 | -1.13 | 0.34 | -0.22 | 0.59 | 2.22 | -1.19 | 0.00 | 0.05 |
| Q9H469 | F-box/LRR-repeat protein 15 | FBXL15 | 1.17 | 0.96 | 1.22 | -1.11 | -1.22 | 0.08 | -0.09 | 1.37 | 1.11 | -1.26 | 0.12 | -0.23 |
| Q15173 | Serine/threonine-protein phosphatase 2A 56 kDa regulatory subuni | PPP2R5B | 1.17 | 3.00 | 1.08 | -1.10 | -1.78 | -0.01 | -0.70 | 1.68 | 0.22 | 0.20 | 0.14 | 0.27 |
| Q8NEU8 | DCC-interacting protein 13-beta | APPL2 | 1.17 | 3.02 | 0.06 | -0.56 | -1.71 | 0.34 | -0.61 | 1.53 | -0.46 | -0.60 | 0.58 | 1.42 |
| P53990 | IST1 homolog | IST1 | 1.17 | 4.16 | -1.79 | -0.94 | -0.64 | -0.39 | 0.81 | 0.49 | -0.38 | 1.52 | 0.46 | 0.89 |
| Q12904 | Aminoacyl tRNA synthase complex-interacting multifunctional pr | AIMP1 | 1.17 | 2.86 | -0.19 | -1.05 | 0.03 | -0.75 | -0.55 | 0.37 | 1.60 | -0.97 | -0.30 | 1.80 |
| Q03013 | Glutathione S-transferase Mu 4 | GSTM4 | 1.17 | 1.13 | 0.73 | -1.38 | -0.89 | 0.57 | -0.30 | 0.93 | 1.04 | -1.69 | 0.31 | 0.67 |
| Q9HBL8 | NmrA-like family domain-containing protein 1 | NMRAL1 | 1.16 | 0.93 | 1.26 | -0.60 | -1.47 | 0.07 | -0.35 | 1.04 | 1.09 | -1.51 | 0.46 | 0.00 |
| Q6ZUJ8 | Phosphoinositide 3-kinase adapter protein 1 | PIK3AP1 | 1.16 | 1.86 | -0.27 | 0.12 | 0.70 | -1.11 | -1.27 | 0.48 | 0.08 | 0.68 | -1.25 | 1.83 |
| Q96I15 | Selenocysteine lyase | SCLY | 1.16 | 2.23 | -0.32 | -0.49 | -0.64 | -0.30 | -0.44 | -0.42 | -0.34 | 2.62 | 0.78 | -0.44 |
| P58546 | Myotrophin | MTPN | 1.16 | 5.45 | -0.66 | 0.42 | -0.34 | -1.25 | -1.51 | 1.10 | 1.07 | 1.12 | -0.61 | 0.66 |
| P51003 | Poly(A) polymerase alpha | PAPOLA | 1.16 | 2.39 | -1.47 | -1.62 | 0.75 | 0.60 | -0.42 | -0.05 | -0.18 | 1.65 | 0.58 | 0.16 |
| O75052 | Carboxyl-terminal PDZ ligand of neuronal nitric oxide synthase pr | NOS1AP | 1.16 | 1.43 | -0.90 | 0.40 | 1.12 | -1.04 | -1.08 | 0.89 | 1.30 | -1.35 | 0.27 | 0.39 |
| Q9NTJ4 | Alpha-mannosidase 2C1 | MAN2C1 | 1.15 | 1.04 | -0.85 | 0.19 | -1.16 | 0.12 | 0.50 | -1.01 | -1.00 | 1.96 | 0.70 | 0.54 |
| O14782 | Kinesin-like protein KIF3C | KIF3C | 1.15 | 1.88 | -0.65 | -0.08 | -2.33 | 0.62 | 0.57 | -0.33 | 0.03 | 0.26 | 0.47 | 1.43 |
| Q8WUM4 | Programmed cell death 6-interacting protein | PDCD6IP | 1.15 | 7.22 | -1.15 | -0.11 | -0.17 | -0.74 | -1.57 | 1.16 | 1.45 | 0.79 | -0.33 | 0.67 |
| Q5TC84 | Opioid growth factor receptor-like protein 1 | OGFRL1 | 1.15 | 2.46 | -1.14 | 0.72 | -1.39 | -0.21 | -0.17 | 1.79 | 0.59 | -1.10 | 0.53 | 0.38 |
| P49189 | 4-trimethylaminobutyraldehyde dehydrogenase | ALDH9A1 | 1.15 | 2.93 | -0.48 | -1.14 | 0.09 | -0.22 | -0.79 | 1.16 | -0.65 | 2.24 | -0.11 | -0.11 |
| P04733 | Metallothionein-1F | MT1F | 1.15 | 1.81 | -0.19 | 0.05 | -0.70 | -0.45 | -0.58 | 0.69 | -0.67 | 2.57 | -0.60 | -0.11 |
| Q9NZJ9 | Diphosphoinositol polyphosphate phosphohydrolase 2 | NUDT4 | 1.15 | 4.21 | -1.53 | 0.64 | -1.33 | -1.45 | 0.41 | 0.80 | 0.68 | 0.50 | 0.59 | 0.69 |
| O15040 | Tectonin beta-propeller repeat-containing protein 2 | TECPR2 | 1.15 | 1.57 | 1.22 | -0.43 | -1.61 | -0.05 | -0.74 | 1.63 | 1.07 | -0.37 | -0.39 | -0.33 |
| Q9NUQ7 | Ufm1-specific protease 2 | UFSP2 | 1.15 | 2.21 | -0.81 | 0.28 | -2.02 | 0.76 | -0.25 | 0.74 | -0.69 | -0.01 | 0.50 | 1.51 |
| P07108 | Acyl-CoA-binding protein | DBI | 1.15 | 1.86 | -1.77 | 0.59 | -0.39 | -0.65 | 0.40 | 0.49 | 0.00 | 2.01 | -0.71 | 0.03 |
| Q86UP2 | Kinectin | KTN1 | 1.15 | 1.91 | 0.98 | -1.41 | -0.79 | -0.21 | -0.42 | 0.76 | 1.40 | -0.54 | -0.97 | 1.19 |
| P50213 | Isocitrate dehydrogenase [NAD] subunit alpha, mitochondrial | IDH3A | 1.14 | 2.73 | -0.51 | -2.22 | 1.16 | -0.18 | -0.69 | 0.58 | 1.04 | 0.71 | 0.02 | 0.09 |
| Q9BUV0 | Arginine/serine-rich protein 1 | RSRP1 | 1.14 | 1.44 | 0.88 | -0.04 | -2.06 | -0.73 | 0.42 | 1.11 | 1.21 | -0.49 | -0.52 | 0.21 |
| P50135 | Histamine N-methyltransferase | HNMT | 1.14 | 2.45 | 0.79 | -1.03 | -0.17 | -0.58 | -1.20 | -0.05 | 0.76 | 2.15 | -0.54 | -0.14 |
| O95336 | 6-phosphogluconolactonase | PGLS | 1.14 | 4.92 | -1.26 | -0.23 | -0.30 | -0.62 | -0.94 | -0.62 | 1.79 | 1.44 | 0.18 | 0.56 |
| Q9H074 | Polyadenylate-binding protein-interacting protein 1 | PAIP1 | 1.14 | 1.25 | 1.41 | -0.78 | -1.02 | -0.12 | -0.86 | 0.89 | -0.80 | 1.28 | -0.87 | 0.87 |
| Q13188 | Serine/threonine-protein kinase 3 | STK3 | 1.14 | 2.23 | -0.26 | -0.52 | -0.55 | -0.47 | -0.40 | -0.36 | -0.37 | -0.78 | 2.18 | 1.53 |
| Q13547 | Histone deacetylase 1 | HDAC1 | 1.14 | 2.11 | -0.29 | -0.34 | -0.61 | -0.42 | -0.46 | -0.38 | -0.37 | -0.86 | 1.61 | 2.11 |
| P28065 | Proteasome subunit beta type-9 | PSMB9 | 1.14 | 3.08 | 0.72 | -2.28 | -0.04 | -0.42 | -0.53 | 1.13 | 0.74 | 0.98 | -0.09 | -0.21 |

| | | | | | | | | | | | | | | |
|---|---|---|---|---|---|---|---|---|---|---|---|---|---|---|
| O14949 | Cytochrome b-c1 complex subunit 8 | UQCRQ | 1.14 | 3.45 | -0.29 | -0.62 | -0.81 | -0.48 | -0.69 | -0.77 | 1.64 | 0.96 | -0.56 | 1.63 |
| O75828 | Carbonyl reductase [NADPH] 3 | CBR3 | 1.14 | 2.04 | -0.20 | -0.35 | -1.29 | 0.44 | -0.58 | 1.07 | -1.59 | 0.38 | 0.51 | 1.60 |
| O00291 | Huntingtin-interacting protein 1 | HIP1 | 1.14 | 3.44 | -1.26 | -0.55 | -0.81 | 0.50 | -0.59 | 1.28 | 0.27 | -1.00 | 0.44 | 1.72 |
| Q9C0E8 | Endoplasmic reticulum junction formation protein lunapark | LNPK | 1.14 | 4.70 | -1.79 | -0.49 | -0.34 | 0.42 | -0.92 | 1.29 | -0.15 | -0.24 | 1.41 | 0.83 |
| Q16539 | Mitogen-activated protein kinase 14 | MAPK14 | 1.13 | 1.69 | 0.81 | -0.68 | -1.25 | 0.51 | -1.09 | 0.41 | 1.35 | -1.36 | 0.33 | 0.97 |
| P22033 | Methylmalonyl-CoA mutase, mitochondrial | MMUT | 1.13 | 2.08 | -1.47 | -1.86 | 1.02 | 0.00 | 0.31 | -0.46 | 0.35 | 0.70 | 1.17 | 0.24 |
| P06737 | Glycogen phosphorylase, liver form | PYGL | 1.13 | 2.12 | -0.54 | -0.70 | -0.97 | 1.06 | -0.84 | 0.43 | 1.82 | -1.19 | 0.67 | 0.26 |
| Q8NAP3 | Zinc finger and BTB domain-containing protein 38 | ZBTB38 | 1.13 | 0.91 | 1.37 | -0.54 | -0.72 | -0.58 | -0.60 | 1.66 | 1.29 | -0.75 | -0.56 | -0.56 |
| O14745 | Na(+)/H(+) exchange regulatory cofactor NHE-RF1 | SLC9A3R1 | 1.13 | 1.78 | -0.15 | -0.55 | -0.78 | -1.65 | 1.35 | 0.20 | 0.10 | 1.84 | -0.29 | -0.08 |
| Q9H6T3 | RNA polymerase II-associated protein 3 | RPAP3 | 1.13 | 1.68 | -0.74 | -1.05 | -1.13 | 0.95 | 0.28 | -0.82 | -0.80 | 0.86 | 1.06 | 1.39 |
| P51687 | Sulfite oxidase, mitochondrial | SUOX | 1.13 | 1.78 | -0.30 | 0.13 | -0.66 | -0.54 | -0.48 | -0.55 | -0.43 | 2.54 | -0.55 | 0.84 |
| Q15326 | Zinc finger MYND domain-containing protein 11 | ZMYND11 | 1.13 | 1.60 | 0.90 | -1.70 | -1.34 | -0.16 | 0.65 | 0.61 | 1.60 | -0.08 | -0.22 | -0.26 |
| Q7LBR1 | Charged multivesicular body protein 1b | CHMP1B | 1.13 | 2.60 | -0.01 | -2.64 | 0.37 | -0.01 | -0.08 | -0.06 | 0.31 | 0.86 | 0.27 | 0.99 |
| Q9ULB1 | Neurexin-1 | NRXN1 | 1.12 | 1.44 | -0.21 | -0.48 | -0.54 | 0.12 | -0.48 | -0.44 | 2.75 | 0.19 | -0.52 | -0.39 |
| P27635 | 60S ribosomal protein L10 | RPL10 | 1.12 | 1.28 | 0.16 | -0.22 | 0.80 | -1.19 | -0.95 | 0.54 | 1.07 | -1.55 | -0.13 | 1.46 |
| Q5T5Y3 | Calmodulin-regulated spectrin-associated protein 1 | CAMSAP1 | 1.12 | 1.24 | 0.59 | 0.16 | -2.74 | 0.58 | 0.00 | 0.15 | 0.66 | -0.14 | 0.35 | 0.40 |
| P14384 | Carboxypeptidase M | CPM | 1.12 | 1.74 | 0.35 | -0.89 | -0.98 | 0.64 | -0.88 | -0.78 | -0.89 | 1.72 | 1.11 | 0.59 |
| Q12965 | Unconventional myosin-Ie | MYO1E | 1.12 | 1.70 | 0.52 | -0.24 | 0.15 | 0.35 | -2.52 | 0.82 | 0.49 | -0.74 | 0.45 | 0.71 |
| P11802 | Cyclin-dependent kinase 4 | CDK4 | 1.12 | 2.23 | 0.03 | -0.58 | -0.83 | -0.19 | -0.59 | 2.33 | 0.91 | -0.78 | -0.74 | 0.44 |
| Q8WVC6 | Dephospho-CoA kinase domain-containing protein | DCAKD | 1.12 | 3.04 | 0.73 | -1.12 | -1.83 | 0.36 | -0.65 | 0.86 | -0.29 | 1.54 | 0.34 | 0.05 |
| Q9HCC0 | Methylcrotonoyl-CoA carboxylase beta chain, mitochondrial | MCCC2 | 1.11 | 2.79 | -1.61 | 0.44 | -0.58 | -1.08 | 0.48 | 1.56 | -0.14 | 1.23 | -0.63 | 0.34 |
| Q15814 | Tubulin-specific chaperone C | TBCC | 1.11 | 1.73 | 0.29 | -0.04 | -2.50 | 0.40 | 0.09 | 1.50 | 0.41 | -0.27 | 0.00 | 0.11 |
| Q6UX71 | Plexin domain-containing protein 2 | PLXDC2 | 1.11 | 2.43 | -0.31 | -0.47 | -0.64 | -0.28 | -0.63 | -0.49 | -0.55 | 1.60 | -0.38 | 2.13 |
| P16152 | Carbonyl reductase [NADPH] 1 | CBR1 | 1.11 | 3.68 | -0.59 | -0.40 | 0.06 | 0.18 | -2.01 | 1.76 | -0.29 | -0.08 | 0.31 | 1.07 |
| P05155 | Plasma protease C1 inhibitor | SERPING1 | 1.11 | 1.63 | -0.59 | -0.85 | -0.80 | 0.57 | -0.02 | -0.74 | -0.71 | 2.30 | 0.06 | 0.78 |
| P02763 | Alpha-1-acid glycoprotein 1 | ORM1 | 1.11 | 0.96 | 1.55 | -0.23 | -1.34 | -1.27 | 0.18 | 0.74 | 0.40 | 1.18 | -0.99 | -0.20 |
| Q13409 | Cytoplasmic dynein 1 intermediate chain 2 | DYNC1I2 | 1.11 | 2.84 | -0.63 | -0.45 | -0.35 | 0.05 | -1.13 | 2.53 | -0.09 | -0.58 | 0.17 | 0.47 |
| Q9NR12 | PDZ and LIM domain protein 7 | PDLIM7 | 1.11 | 1.30 | 0.90 | 0.85 | -1.13 | -0.96 | -1.06 | 0.73 | 0.26 | -1.25 | 0.28 | 1.38 |
| Q6UWP2 | Dehydrogenase/reductase SDR family member 11 | DHRS11 | 1.11 | 1.83 | -0.35 | -0.52 | 0.03 | -0.46 | -0.59 | 1.27 | -0.60 | 2.34 | -0.61 | -0.50 |
| Q92888 | Rho guanine nucleotide exchange factor 1 | ARHGEF1 | 1.11 | 0.96 | 1.59 | -0.59 | -0.78 | -0.63 | -0.70 | 1.49 | 1.10 | -0.87 | -0.67 | 0.08 |
| P19652 | Alpha-1-acid glycoprotein 2 | ORM2 | 1.10 | 1.35 | -0.17 | -0.30 | -0.43 | -0.22 | -0.39 | -0.38 | -0.34 | 2.84 | -0.30 | -0.31 |
| P21589 | 5'-nucleotidase | NT5E | 1.10 | 2.08 | -1.18 | -0.34 | -0.09 | 1.06 | -1.40 | -1.28 | 0.56 | 0.80 | 1.04 | 0.84 |
| Q6NTE8 | MRN complex-interacting protein | MRNIP | 1.10 | 2.72 | 0.52 | -0.20 | -0.78 | -0.27 | -1.61 | 1.25 | 1.48 | -0.98 | -0.23 | 0.81 |
| Q8IYJ1 | Copine-9 | CPNE9 | 1.10 | 1.35 | -0.11 | -0.27 | -0.49 | -0.25 | -0.40 | -0.22 | 2.82 | -0.55 | -0.27 | -0.26 |
| Q9UK61 | Protein TASOR | TASOR | 1.10 | 0.94 | 1.23 | -1.03 | -1.16 | -0.81 | 0.68 | 0.94 | 1.31 | -1.15 | 0.05 | -0.06 |
| Q99623 | Prohibitin-2 | PHB2 | 1.10 | 1.19 | 1.37 | 0.62 | -0.79 | -1.13 | -1.39 | 0.39 | 1.16 | 0.70 | -0.96 | 0.03 |
| Q9UKV3 | Apoptotic chromatin condensation inducer in the nucleus | ACIN1 | 1.10 | 2.42 | 1.28 | -0.97 | -1.27 | -0.74 | -0.46 | 1.81 | 0.65 | -0.44 | -0.17 | 0.31 |
| O14981 | TATA-binding protein-associated factor 172 | BTAF1 | 1.10 | 1.29 | 1.38 | -1.43 | -1.13 | -0.27 | 0.05 | 0.82 | 1.09 | 0.85 | -0.37 | -0.99 |
| Q9NTI5 | Sister chromatid cohesion protein PDS5 homolog B | PDS5B | 1.10 | 1.71 | 1.18 | -0.03 | -1.07 | 0.12 | -1.93 | 1.40 | -0.50 | -0.09 | 0.66 | 0.26 |
| Q15257 | Serine/threonine-protein phosphatase 2A activator | PTPA | 1.10 | 3.57 | -0.33 | 0.05 | -2.39 | 0.34 | -0.47 | 1.24 | 0.39 | -0.21 | 0.39 | 0.98 |
| Q9Y6M4 | Casein kinase I isoform gamma-3 | CSNK1G3 | 1.10 | 2.44 | -1.18 | -0.10 | 0.47 | 0.61 | -1.99 | 0.35 | 1.27 | 0.34 | -0.65 | 0.88 |
| Q8N122 | Regulatory-associated protein of mTOR | RPTOR | 1.10 | 0.65 | 0.98 | 0.01 | -1.50 | -0.21 | -0.10 | 1.16 | 1.43 | -1.53 | -0.11 | -0.14 |
| Q9UPT5 | Exocyst complex component 7 | EXOC7 | 1.10 | 1.57 | -0.02 | 0.77 | -2.51 | -0.26 | 0.37 | -0.59 | 0.81 | 0.78 | 0.13 | 0.51 |
| Q12768 | WASH complex subunit 5 | WASHC5 | 1.09 | 1.94 | -0.99 | -0.28 | 0.46 | 0.06 | -1.15 | 1.18 | 0.80 | -1.59 | 0.17 | 1.35 |
| P24928 | DNA-directed RNA polymerase II subunit RPB1 | POLR2A | 1.09 | 1.12 | 0.02 | 0.05 | -1.23 | 0.33 | -0.45 | 1.27 | 1.67 | -1.49 | 0.42 | -0.59 |
| Q8TD26 | Chromodomain-helicase-DNA-binding protein 6 | CHD6 | 1.09 | 1.21 | 0.49 | -0.86 | -1.01 | -0.88 | 0.93 | 0.32 | 0.98 | 1.70 | -0.90 | -0.77 |
| P52298 | Nuclear cap-binding protein subunit 2 | NCBP2 | 1.09 | 2.33 | 1.52 | -0.87 | -1.04 | -0.81 | -0.92 | 1.25 | 0.84 | 0.22 | -0.80 | 0.61 |
| P0C0L5 | Complement C4-B | C4B | 1.09 | 0.90 | -1.18 | -1.27 | 1.12 | 0.48 | -0.21 | -0.42 | -1.07 | 1.66 | 0.43 | 0.45 |
| Q13363 | C-terminal-binding protein 1 | CTBP1 | 1.09 | 0.98 | 0.55 | 0.59 | -2.77 | 0.29 | 0.16 | 0.42 | 0.53 | -0.15 | 0.29 | 0.08 |
| P12235 | ADP/ATP translocase 1 | SLC25A4 | 1.09 | 1.83 | -1.22 | -1.40 | 0.72 | -0.36 | 0.47 | -1.32 | 1.06 | 0.78 | 0.18 | 1.08 |
| P11413 | Glucose-6-phosphate 1-dehydrogenase | G6PD | 1.08 | 4.59 | -0.61 | -0.30 | -0.18 | -0.88 | -1.26 | -0.09 | 0.53 | 0.24 | 0.16 | 2.40 |
| Q5SRE7 | Phytanoyl-CoA dioxygenase domain-containing protein 1 | PHYHD1 | 1.08 | 1.08 | 0.35 | -0.16 | -2.25 | 0.80 | 0.04 | 0.78 | -0.31 | -0.96 | 1.00 | 0.71 |
| Q01995 | Transgelin | TAGLN | 1.08 | 1.62 | 0.10 | 0.07 | -1.22 | -0.34 | -0.31 | -1.59 | 0.48 | 1.33 | -0.17 | 1.65 |
| Q8NFP9 | Neurobeachin | NBEA | 1.08 | 0.58 | 0.87 | 0.05 | -1.54 | -0.15 | 0.03 | 1.58 | 1.25 | -0.75 | -0.26 | -1.08 |
| P02679 | Fibrinogen gamma chain | FGG | 1.08 | 4.97 | -0.73 | -0.90 | 0.45 | -1.13 | -0.93 | 0.77 | 0.87 | 1.19 | -0.90 | 1.31 |
| P14550 | Aldo-keto reductase family 1 member A1 | AKR1A1 | 1.08 | 3.14 | -1.16 | -0.21 | 0.08 | -0.10 | -1.20 | 1.15 | -1.42 | 0.98 | 0.68 | 1.19 |
| P00325 | All-trans-retinol dehydrogenase [NAD(+)] ADH1B | ADH1B | 1.08 | 1.11 | 0.20 | -1.77 | 1.17 | 0.14 | -1.00 | -0.55 | -0.56 | 1.58 | 0.51 | 0.28 |
| P38919 | Eukaryotic initiation factor 4A-III | EIF4A3 | 1.08 | 3.00 | 0.25 | -0.49 | -0.58 | 0.38 | -2.02 | 0.20 | -0.78 | 0.84 | 0.68 | 1.53 |
| O43813 | Glutathione S-transferase LANCL1 | LANCL1 | 1.08 | 5.64 | -0.37 | -1.06 | -0.63 | 0.39 | -1.73 | 0.65 | -0.36 | 1.55 | 0.68 | 0.88 |
| Q9Y247 | Protein FAM50B | FAM50B | 1.08 | 2.69 | 0.24 | -1.48 | 0.73 | -0.27 | -1.53 | -0.81 | 0.19 | 0.58 | 1.09 | 1.25 |
| O43809 | Cleavage and polyadenylation specificity factor subunit 5 | NUDT21 | 1.07 | 1.08 | 0.24 | -0.67 | -1.10 | 0.16 | 0.13 | -1.31 | 2.34 | 0.06 | 0.20 | -0.05 |
| Q9BZC7 | ATP-binding cassette sub-family A member 2 | ABCA2 | 1.07 | 0.89 | 0.97 | -0.43 | -1.34 | 0.42 | -0.66 | 1.65 | 0.73 | -1.46 | 0.25 | -0.11 |
| Q12789 | General transcription factor 3C polypeptide 1 | GTF3C1 | 1.07 | 0.89 | 1.27 | -0.81 | -0.97 | -0.36 | -0.18 | 1.54 | 1.25 | -1.00 | 0.10 | -0.84 |
| P11940 | Polyadenylate-binding protein 1 | PABPC1 | 1.07 | 2.74 | 1.38 | -0.96 | -0.75 | -0.54 | -1.50 | 1.18 | 1.08 | 0.58 | -0.08 | -0.40 |
| O60934 | Nibrin | NBN | 1.07 | 1.87 | -0.49 | -0.64 | -0.89 | 0.98 | -0.79 | -0.69 | 1.52 | -0.99 | 1.33 | 0.67 |
| Q96T58 | Msx2-interacting protein | SPEN | 1.07 | 1.81 | 0.70 | -1.96 | -0.50 | -0.20 | 0.17 | 0.15 | 2.06 | -0.04 | -0.14 | -0.25 |
| Q96PM5 | RING finger and CHY zinc finger domain-containing protein 1 | RCHY1 | 1.07 | 2.27 | 0.55 | -0.77 | -1.05 | 0.05 | -0.87 | 2.33 | 0.32 | -0.81 | 0.17 | 0.09 |
| Q96HC4 | PDZ and LIM domain protein 5 | PDLIM5 | 1.07 | 1.56 | 1.47 | -0.95 | -0.92 | -0.37 | -0.83 | 1.36 | 1.31 | -0.77 | -0.34 | 0.04 |
| Q9BUP3 | Oxidoreductase HTATIP2 | HTATIP2 | 1.07 | 1.18 | -0.01 | 1.23 | 0.04 | -1.30 | -1.27 | 0.65 | -1.33 | 0.90 | -0.01 | 1.09 |
| P39060 | Collagen alpha-1(XVIII) chain | COL18A1 | 1.07 | 1.79 | 0.16 | -1.29 | 1.25 | -1.76 | -0.15 | -0.63 | 0.54 | 0.23 | 0.32 | 1.33 |
| Q9P2T1 | GMP reductase 2 | GMPR2 | 1.07 | 1.69 | -0.92 | -1.20 | 0.78 | 0.24 | -0.61 | 0.82 | -1.53 | 1.51 | 0.46 | 0.45 |
| Q8TER5 | Rho guanine nucleotide exchange factor 40 | ARHGEF40 | 1.07 | 2.13 | -0.76 | 0.43 | -1.11 | -0.39 | -0.21 | -0.03 | -0.87 | 2.43 | 0.13 | 0.38 |
| O95861 | 3'(2'),5'-bisphosphate nucleotidase 1 | BPNT1 | 1.07 | 2.70 | -0.60 | 0.11 | -0.04 | 0.56 | -2.39 | -0.14 | 0.62 | 1.34 | 0.61 | -0.06 |
| Q14558 | Phosphoribosyl pyrophosphate synthase-associated protein 1 | PRPSAP1 | 1.07 | 1.87 | 0.59 | -2.23 | 0.27 | 0.39 | -0.87 | 1.22 | 0.25 | 0.94 | -0.05 | -0.51 |
| Q9NXD2 | Myotubularin-related protein 10 | MTMR10 | 1.06 | 1.66 | 0.20 | -1.01 | 0.07 | 0.28 | -1.24 | 1.35 | -1.70 | 0.91 | 0.33 | 0.82 |
| Q12802 | A-kinase anchor protein 13 | AKAP13 | 1.06 | 1.97 | 0.78 | 0.16 | -2.47 | 0.12 | -0.53 | 0.62 | 0.61 | -0.54 | 0.36 | 0.89 |
| O95628 | CCR4-NOT transcription complex subunit 4 | CNOT4 | 1.06 | 1.23 | -0.12 | -0.24 | -0.45 | -0.24 | -0.36 | -0.23 | 2.82 | -0.61 | -0.33 | -0.23 |
| P04899 | Guanine nucleotide-binding protein G(i) subunit alpha-2 | GNAI2 | 1.06 | 3.36 | -1.44 | 0.08 | -0.10 | 0.59 | -1.79 | 1.52 | 0.28 | -0.41 | 0.73 | 0.54 |
| Q16890 | Tumor protein D53 | TPD52L1 | 1.06 | 1.39 | 0.18 | -0.78 | -1.85 | 0.50 | 0.47 | 0.74 | 0.40 | -1.34 | 0.32 | 1.36 |
| P57052 | Splicing regulator RBM11 | RBM11 | 1.06 | 0.98 | 0.45 | -0.79 | -0.93 | 1.02 | -0.88 | 0.45 | -0.86 | -1.07 | 1.23 | 1.38 |
| Q6SPF0 | Atherin | SAMD1 | 1.06 | 2.18 | -0.18 | -1.52 | -1.52 | 0.49 | 0.71 | -0.54 | -0.39 | 0.59 | 1.26 | 1.10 |
| P27797 | Calreticulin | CALR | 1.06 | 2.60 | -1.38 | -0.05 | 1.12 | -0.39 | -1.58 | 1.13 | 0.65 | -0.64 | 0.10 | 1.04 |
| P31948 | Stress-induced-phosphoprotein 1 | STIP1 | 1.06 | 1.85 | -0.82 | -1.63 | -0.26 | 0.00 | 0.90 | -0.47 | 0.08 | 2.13 | -0.01 | 0.08 |

| | | | | | | | | | | | | | | |
|---|---|---|---|---|---|---|---|---|---|---|---|---|---|---|
| Q13228 | Methanethiol oxidase | SELENBP1 | 1.06 | 1.94 | -1.89 | -0.52 | -0.13 | 0.03 | 0.65 | -0.95 | 0.22 | 1.86 | 0.23 | 0.52 |
| Q562E7 | WD repeat-containing protein 81 | WDR81 | 1.05 | 1.54 | 0.45 | 0.45 | -2.04 | 0.21 | -0.66 | 0.85 | 1.04 | -1.29 | 0.44 | 0.56 |
| Q9P2J5 | Leucine--tRNA ligase, cytoplasmic | LARS | 1.05 | 1.57 | 0.60 | -1.50 | -1.73 | 0.64 | 0.37 | 0.35 | 0.99 | -0.97 | 0.58 | 0.69 |
| Q9BU02 | Thiamine-triphosphatase | THTPA | 1.05 | 1.30 | -0.76 | 0.95 | -1.11 | 0.45 | -0.94 | -0.86 | -0.95 | 1.36 | 0.90 | 0.96 |
| P35244 | Replication protein A 14 kDa subunit | RPA3 | 1.05 | 2.00 | -0.05 | 0.30 | -0.36 | 0.28 | -2.07 | 1.12 | 1.30 | -1.09 | 0.05 | 0.53 |
| Q96T76 | MMS19 nucleotide excision repair protein homolog | MMS19 | 1.04 | 2.95 | 0.53 | -1.67 | -0.68 | -0.05 | -0.57 | -0.19 | 2.22 | 0.05 | -0.05 | 0.41 |
| P09110 | 3-ketoacyl-CoA thiolase, peroxisomal | ACAA1 | 1.04 | 1.44 | -0.83 | -0.90 | 0.51 | 0.64 | -0.95 | -0.95 | -0.96 | 1.43 | 1.13 | 0.87 |
| Q9H4A6 | Golgi phosphoprotein 3 | GOLPH3 | 1.04 | 1.32 | -0.42 | -0.54 | -0.77 | -0.62 | 0.92 | -0.61 | -0.49 | 0.79 | -0.53 | 2.28 |
| O95870 | Phosphatidylserine lipase ABHD16A | ABHD16A | 1.04 | 1.52 | -1.64 | 0.35 | -1.86 | 0.50 | 1.03 | 0.03 | 0.20 | -0.20 | 0.94 | 0.66 |
| P23515 | Oligodendrocyte-myelin glycoprotein | OMG | 1.04 | 1.60 | -0.75 | -1.19 | -0.29 | 0.17 | 0.39 | -0.53 | 0.75 | 2.23 | 0.19 | -0.96 |
| Q9UHB9 | Signal recognition particle subunit SRP68 | SRP68 | 1.04 | 1.61 | 0.80 | -0.99 | 0.22 | 0.00 | -1.67 | 1.08 | 1.09 | -1.16 | -0.25 | 0.88 |
| Q6PML9 | Zinc transporter 9 | SLC30A9 | 1.04 | 1.78 | -1.29 | -0.67 | 1.16 | -0.82 | -0.14 | -1.17 | 1.56 | 0.87 | -0.02 | 0.52 |
| P51570 | Galactokinase | GALK1 | 1.03 | 2.56 | -0.21 | -0.02 | -1.24 | 0.04 | -0.89 | 1.93 | 0.59 | -1.33 | 0.25 | 0.87 |
| P48506 | Glutamate--cysteine ligase catalytic subunit | GCLC | 1.03 | 2.14 | -0.53 | 0.31 | -0.05 | 0.06 | -1.78 | 0.55 | 1.97 | -1.04 | 0.15 | 0.37 |
| Q9P2D3 | HEAT repeat-containing protein 5B | HEATR5B | 1.03 | 1.08 | 1.21 | 0.18 | -1.16 | -0.38 | -1.07 | 1.21 | 1.18 | -1.39 | -0.06 | 0.27 |
| P09874 | Poly [ADP-ribose] polymerase 1 | PARP1 | 1.03 | 5.47 | -1.10 | -1.28 | -0.43 | 0.38 | -0.94 | 0.63 | 1.83 | 0.51 | 0.87 | -0.46 |
| P28482 | Mitogen-activated protein kinase 1 | MAPK1 | 1.03 | 8.72 | -1.15 | -0.35 | -0.70 | -0.34 | -1.46 | 0.73 | 1.21 | 1.59 | -0.04 | 0.53 |
| Q9NZJ7 | Mitochondrial carrier homolog 1 | MTCH1 | 1.03 | 1.86 | 0.57 | -0.98 | 0.79 | -0.37 | -1.84 | 1.31 | 0.52 | -0.94 | 0.09 | 0.85 |
| Q15181 | Inorganic pyrophosphatase | PPA1 | 1.03 | 4.16 | -1.27 | -1.20 | 0.51 | -0.24 | -0.75 | 1.65 | 0.52 | 1.24 | 0.23 | -0.69 |
| P61956 | Small ubiquitin-related modifier 2 | SUMO2 | 1.03 | 2.41 | -2.25 | -0.23 | -0.53 | 0.25 | 0.59 | -0.04 | -0.43 | 1.54 | 0.63 | 0.46 |
| O15056 | Synaptojanin-2 | SYNJ2 | 1.03 | 1.20 | 1.30 | -1.04 | -1.09 | 0.54 | -1.04 | 1.32 | 0.76 | -0.41 | 0.58 | -0.92 |
| P42785 | Lysosomal Pro-X carboxypeptidase | PRCP | 1.03 | 1.27 | -0.73 | -0.65 | -0.38 | -0.48 | 0.83 | -0.63 | -0.65 | 2.28 | -0.47 | 0.87 |
| Q96PV6 | Leukocyte receptor cluster member 8 | LENG8 | 1.02 | 1.07 | -0.85 | -1.05 | -1.21 | 0.63 | 1.26 | -0.31 | -1.06 | 1.29 | 0.66 | 0.63 |
| P45973 | Chromobox protein homolog 5 | CBX5 | 1.02 | 2.17 | 0.19 | -1.56 | -1.38 | 0.18 | 0.56 | 1.94 | -0.12 | -0.50 | 0.23 | 0.47 |
| Q9Y2D5 | A-kinase anchor protein 2 | AKAP2 | 1.02 | 0.68 | 1.04 | -0.62 | -0.89 | 0.02 | -0.41 | 1.75 | 1.31 | -0.99 | -0.79 | -0.44 |
| P19174 | 1-phosphatidylinositol 4,5-bisphosphate phosphodiesterase gamma | PLCG1 | 1.02 | 3.56 | -0.14 | -0.70 | -0.98 | -1.19 | 0.23 | 0.79 | 1.97 | 1.00 | -0.69 | -0.29 |
| Q96BR1 | Serine/threonine-protein kinase Sgk3 | SGK3 | 1.02 | 1.44 | 1.00 | -0.87 | -1.08 | 0.50 | -1.05 | 1.28 | 0.04 | -1.21 | 0.14 | 1.27 |
| O94952 | F-box only protein 21 | FBXO21 | 1.02 | 1.38 | -0.45 | -0.64 | -0.79 | -0.62 | 1.02 | -0.57 | -0.60 | 1.64 | -0.60 | 1.61 |
| Q2TAY7 | WD40 repeat-containing protein SMU1 | SMU1 | 1.02 | 2.00 | 0.60 | -2.11 | -1.11 | 0.29 | 0.39 | 0.83 | 0.62 | 0.04 | 1.10 | -0.65 |
| P18887 | DNA repair protein XRCC1 | XRCC1 | 1.02 | 1.37 | 1.03 | -1.09 | -2.38 | 0.25 | 0.65 | 0.34 | 0.43 | 0.51 | 0.19 | 0.07 |
| Q8IX03 | Protein KIBRA | WWC1 | 1.02 | 1.75 | -0.55 | -0.66 | -0.92 | 1.07 | -0.69 | 1.67 | -0.63 | -1.02 | 1.03 | 0.71 |
| O95373 | Importin-7 | IPO7 | 1.02 | 2.05 | 1.46 | -0.29 | -1.92 | -0.17 | -1.09 | 1.17 | -0.09 | 0.03 | 0.22 | 0.67 |
| Q9NYS7 | WD repeat and SOCS box-containing protein 2 | WSB2 | 1.02 | 1.28 | 1.08 | -0.59 | -0.70 | -0.59 | -0.60 | -0.54 | 1.07 | 2.04 | -0.64 | -0.52 |
| P61129 | Zinc finger CCCH domain-containing protein 6 | ZC3H6 | 1.02 | 1.02 | 0.50 | -0.54 | -0.74 | -0.47 | 0.05 | 0.55 | 2.49 | -0.76 | -0.54 | -0.54 |
| G9CGD6 | CNK3/IPCEF1 fusion protein | CNK3/IPCEF1 | 1.01 | 1.50 | 1.44 | -1.28 | -0.52 | 0.05 | -1.25 | 0.75 | -1.20 | 0.50 | 0.64 | 0.88 |
| Q01629 | Interferon-induced transmembrane protein 2 | IFITM2 | 1.01 | 1.31 | -1.32 | -0.04 | 0.68 | 0.68 | -1.41 | -1.41 | 0.64 | 1.12 | 0.79 | 0.27 |
| O14727 | Apoptotic protease-activating factor 1 | APAF1 | 1.01 | 0.76 | 1.31 | -0.42 | -0.45 | -0.86 | -0.50 | 1.53 | 1.46 | -0.56 | -0.81 | -0.69 |
| Q9H269 | Vacuolar protein sorting-associated protein 16 homolog | VPS16 | 1.01 | 2.98 | 0.47 | 0.00 | -0.68 | -0.85 | -1.41 | 1.09 | 0.81 | 1.74 | -0.64 | -0.53 |
| P08238 | Heat shock protein HSP 90-beta | HSP90AB1 | 1.01 | 4.13 | -0.79 | 0.31 | -0.71 | -0.49 | -1.32 | 1.00 | 1.10 | -0.75 | -0.09 | 1.74 |
| O15347 | High mobility group protein B3 | HMGB3 | 1.01 | 1.13 | 0.61 | -0.14 | 0.74 | 0.18 | -2.70 | 0.57 | 0.01 | -0.17 | 0.48 | 0.43 |
| P17302 | Gap junction alpha-1 protein | GJA1 | 1.01 | 2.24 | -0.08 | -1.20 | -1.73 | -0.16 | 1.10 | 1.08 | -0.23 | -0.16 | -0.05 | 1.43 |
| Q15149 | Plectin | PLEC | 1.01 | 2.79 | 0.53 | -0.21 | -1.11 | -0.20 | -1.38 | 2.19 | -0.46 | 0.55 | -0.26 | 0.36 |
| P27694 | Replication protein A 70 kDa DNA-binding subunit | RPA1 | 1.01 | 3.97 | 0.76 | -1.23 | -1.69 | -0.14 | -0.59 | 1.72 | 0.40 | -0.11 | 0.15 | 0.73 |
| P50479 | PDZ and LIM domain protein 4 | PDLIM4 | 1.01 | 2.68 | 0.40 | 0.58 | -1.47 | 0.02 | -1.86 | 1.39 | 0.62 | 0.06 | -0.37 | 0.65 |
| P36871 | Phosphoglucomutase-1 | PGM1 | 1.00 | 1.61 | 1.13 | -1.32 | -0.31 | -0.65 | -0.50 | 1.51 | 1.40 | 0.12 | -0.67 | -0.72 |
| P34896 | Serine hydroxymethyltransferase, cytosolic | SHMT1 | 1.00 | 2.56 | 0.24 | -1.00 | -0.21 | -0.69 | -0.67 | 1.17 | 2.24 | -0.54 | -0.52 | -0.03 |
| O14773 | Tripeptidyl-peptidase 1 | TPP1 | 1.00 | 2.84 | -0.20 | -1.74 | -1.18 | -0.35 | 1.07 | -0.30 | 0.57 | 1.61 | 0.00 | 0.53 |
| P78508 | ATP-sensitive inward rectifier potassium channel 10 | KCNJ10 | 1.00 | 0.94 | 0.94 | -0.39 | 0.73 | -0.36 | -2.01 | 0.74 | 1.19 | -1.00 | -0.28 | 0.46 |
| Q03135 | Caveolin-1 | CAV1 | 1.00 | 1.78 | -0.32 | -1.00 | 1.10 | 0.29 | -1.83 | 0.26 | 1.47 | 0.32 | -0.80 | 0.52 |
| P55087 | Aquaporin-4 | AQP4 | 1.00 | 2.90 | 0.05 | -1.03 | -0.06 | 0.48 | -1.86 | 1.55 | -0.47 | 1.28 | 0.06 | 0.00 |
| Q9HCM4 | Band 4.1-like protein 5 | EPB41L5 | 1.00 | 1.52 | 0.36 | -0.82 | -1.62 | 0.84 | -0.33 | 1.29 | 0.03 | -1.33 | 0.96 | 0.62 |
| Q9GZT8 | NIF3-like protein 1 | NIF3L1 | 1.00 | 2.11 | 1.03 | -0.80 | -1.03 | 0.74 | -1.99 | 0.73 | 0.69 | 0.82 | 0.02 | -0.21 |
| Q6DKJ4 | Nucleoredoxin | NXN | 1.00 | 2.02 | -0.22 | -0.43 | -0.64 | -0.29 | -0.47 | -0.24 | -0.41 | -0.86 | 1.15 | 2.40 |
| Q13907 | Isopentenyl-diphosphate Delta-isomerase 1 | IDI1 | 1.00 | 1.85 | 0.64 | -1.97 | -0.65 | 1.19 | -1.08 | 0.78 | -0.21 | 0.24 | 0.99 | 0.06 |
| Q9H5N1 | Rab GTPase-binding effector protein 2 | RABEP2 | 0.99 | 0.87 | 1.21 | -0.33 | -0.69 | -0.58 | -0.64 | 0.92 | 2.02 | -0.85 | -0.59 | -0.46 |
| P09488 | Glutathione S-transferase Mu 1 | GSTM1 | 0.99 | 2.77 | -1.04 | 0.65 | -1.75 | 0.37 | -0.58 | 1.43 | -0.58 | -0.18 | 0.57 | 1.10 |
| Q9Y4P1 | Cysteine protease ATG4B | ATG4B | 0.99 | 1.73 | 0.81 | 0.56 | -1.10 | -0.14 | -1.87 | 1.36 | 0.47 | -0.90 | 0.15 | 0.65 |
| O94903 | Pyridoxal phosphate homeostasis protein | PLPBP | 0.99 | 3.40 | -0.34 | 0.44 | -1.45 | -0.60 | -0.73 | 0.50 | -0.18 | 2.32 | 0.11 | -0.07 |
| Q15349 | Ribosomal protein S6 kinase alpha-2 | RPS6KA2 | 0.99 | 3.94 | 0.30 | -1.13 | -1.15 | -0.45 | -0.50 | 0.64 | 0.61 | 2.20 | -0.48 | -0.04 |
| O43747 | AP-1 complex subunit gamma-1 | AP1G1 | 0.99 | 1.66 | -0.02 | -0.17 | -0.35 | -0.25 | -0.97 | 0.37 | 2.56 | -1.03 | -0.29 | 0.15 |
| P62136 | Serine/threonine-protein phosphatase PP1-alpha catalytic subunit | PPP1CA | 0.99 | 3.14 | 0.07 | -1.01 | -2.22 | 0.84 | -0.32 | 0.54 | 1.02 | -0.18 | 0.81 | 0.46 |
| Q9Y530 | ADP-ribose glycohydrolase OARD1 | OARD1 | 0.99 | 1.68 | 0.35 | -0.74 | -1.01 | 0.39 | -0.72 | -0.74 | 1.37 | -1.23 | 1.03 | 1.30 |
| P41226 | Ubiquitin-like modifier-activating enzyme 7 | UBA7 | 0.99 | 1.01 | -0.36 | -0.39 | -0.36 | -0.04 | -0.06 | -1.03 | -0.45 | -0.91 | 1.83 | 1.78 |
| P23526 | Adenosylhomocysteinase | AHCY | 0.99 | 1.87 | -0.32 | -0.95 | 0.54 | -0.60 | -0.54 | 0.50 | -0.64 | 2.48 | 0.06 | -0.52 |
| Q9GZP4 | PITH domain-containing protein 1 | PITHD1 | 0.99 | 1.75 | 1.13 | 0.25 | -1.15 | -0.77 | -1.20 | -0.39 | -0.26 | 1.93 | -0.12 | 0.59 |
| P55345 | Protein arginine N-methyltransferase 2 | PRMT2 | 0.99 | 2.49 | 0.01 | 0.06 | 0.71 | -0.69 | -2.34 | 0.56 | -0.58 | 0.47 | 0.74 | 1.06 |
| P54136 | Arginine--tRNA ligase, cytoplasmic | RARS | 0.98 | 2.19 | -1.64 | -0.59 | 1.20 | -0.13 | -0.87 | -0.53 | -0.16 | 1.22 | 0.10 | 1.39 |
| P35555 | Fibrillin-1 | FBN1 | 0.98 | 0.72 | 0.66 | -1.19 | -0.56 | -0.63 | 0.85 | 1.89 | 0.81 | -0.84 | -0.86 | -0.12 |
| P00747 | Plasminogen | PLG | 0.98 | 1.42 | 0.36 | 0.14 | -0.07 | 0.09 | -2.01 | 1.47 | -1.22 | 0.06 | 0.13 | 1.06 |
| P46976 | Glycogenin-1 | GYG1 | 0.98 | 1.05 | -0.48 | -1.78 | -0.78 | 1.01 | 0.84 | -0.11 | 0.48 | 0.27 | 1.46 | -0.91 |
| Q92736 | Ryanodine receptor 2 | RYR2 | 0.98 | 1.51 | 1.26 | -1.25 | -0.18 | 0.26 | -1.67 | 0.98 | 1.06 | -0.85 | -0.02 | 0.42 |
| P16930 | Fumarylacetoacetase | FAH | 0.98 | 1.17 | 0.29 | -0.63 | -1.16 | 0.60 | -0.43 | -1.00 | -0.66 | 2.21 | 0.33 | 0.43 |
| P50395 | Rab GDP dissociation inhibitor beta | GDI2 | 0.98 | 3.13 | -0.29 | -0.78 | 0.42 | -0.17 | -1.71 | 1.57 | 0.72 | 1.14 | 0.03 | -0.94 |
| P23396 | 40S ribosomal protein S3 | RPS3 | 0.98 | 5.18 | 0.21 | -1.18 | -0.80 | 0.16 | -1.66 | 1.50 | 1.23 | -0.15 | 0.11 | 0.57 |
| P04080 | Cystatin-B | CSTB | 0.98 | 3.93 | -0.88 | -0.65 | -0.39 | 0.32 | -1.32 | 0.80 | -1.05 | 1.74 | 0.80 | 0.64 |
| P38571 | Lysosomal acid lipase/cholesteryl ester hydrolase | LIPA | 0.98 | 1.40 | 0.44 | -1.50 | -1.79 | 0.40 | 0.95 | 0.04 | 0.40 | 1.08 | 0.69 | -0.71 |
| P0DI82 | Trafficking protein particle complex subunit 2B | TRAPPC2B | 0.98 | 1.54 | -0.59 | -0.60 | -0.91 | 1.15 | -0.63 | 0.63 | -0.71 | -1.05 | 1.30 | 1.43 |
| Q9H1K0 | Rabenosyn-5 | RBSN | 0.98 | 1.37 | 1.27 | -0.77 | -1.97 | 0.06 | -0.06 | 0.77 | 1.25 | 0.40 | -0.20 | -0.75 |
| Q99598 | Translin-associated protein X | TSNAX | 0.98 | 1.81 | -0.58 | 0.70 | -1.07 | 0.12 | -0.96 | -0.70 | -0.91 | 1.82 | 1.14 | 0.45 |
| Q9H9A6 | Leucine-rich repeat-containing protein 40 | LRRC40 | 0.97 | 0.74 | 1.44 | 0.39 | -1.69 | -0.12 | -0.93 | 1.30 | 0.19 | -1.09 | 0.18 | 0.32 |
| Q8N8L2 | Zinc finger protein 491 | ZNF491 | 0.97 | 1.22 | 0.58 | -1.32 | -1.44 | 0.19 | 0.65 | 0.43 | 1.51 | 0.36 | 0.35 | -1.30 |
| P16885 | 1-phosphatidylinositol 4,5-bisphosphate phosphodiesterase gamma | PLCG2 | 0.97 | 1.92 | -2.29 | 1.17 | 0.35 | -0.76 | -0.40 | 0.06 | 1.13 | 0.14 | 0.17 | 0.44 |
| Q8N6N7 | Acyl-CoA-binding domain-containing protein 7 | ACBD7 | 0.97 | 1.41 | -1.04 | -0.50 | -1.05 | -1.07 | 2.11 | -0.18 | 0.35 | 0.40 | 0.22 | 0.76 |

| | | | | | | | | | | | | | | |
|---|---|---|---|---|---|---|---|---|---|---|---|---|---|---|
| P49821 | NADH dehydrogenase [ubiquinone] flavoprotein 1, mitochondrial | NDUFV1 | 0.97 | 1.01 | -0.67 | -0.28 | 1.39 | -0.79 | -0.81 | 1.32 | -0.72 | -1.11 | 0.40 | 1.27 |
| P17066 | Heat shock 70 kDa protein 6 | HSPA6 | 0.96 | 1.38 | -0.31 | 0.65 | -0.74 | -0.47 | -0.64 | -0.52 | -0.45 | -0.95 | 1.92 | 1.51 |
| Q9ULP9 | TBC1 domain family member 24 | TBC1D24 | 0.96 | 1.97 | -0.30 | 0.28 | -1.60 | 0.03 | -0.32 | -1.14 | -0.16 | 1.52 | 0.11 | 1.59 |
| Q9Y478 | 5'-AMP-activated protein kinase subunit beta-1 | PRKAB1 | 0.96 | 1.17 | 0.73 | -0.08 | -2.05 | 0.26 | -0.16 | 1.33 | 1.25 | -0.50 | -0.01 | -0.77 |
| Q9Y5B8 | Nucleoside diphosphate kinase 7 | NME7 | 0.96 | 2.28 | 0.04 | -1.72 | -1.11 | 0.04 | 0.66 | 0.81 | 1.58 | -0.78 | -0.26 | 0.72 |
| Q9BR76 | Coronin-1B | CORO1B | 0.96 | 4.06 | -0.86 | -1.22 | -0.48 | -0.92 | 0.54 | 0.60 | 1.92 | 1.04 | -0.54 | -0.08 |
| Q9UK41 | Vacuolar protein sorting-associated protein 28 homolog | VPS28 | 0.96 | 1.70 | 0.84 | 0.26 | -2.56 | 0.43 | -0.76 | 0.67 | 0.07 | 0.14 | 0.59 | 0.32 |
| P52306 | Rap1 GTPase-GDP dissociation stimulator 1 | RAP1GDS1 | 0.96 | 2.34 | -1.75 | 1.18 | -0.29 | -0.61 | -0.64 | 0.85 | 1.51 | -0.51 | -0.31 | 0.58 |
| P78386 | Keratin, type II cuticular Hb5 | KRT85 | 0.96 | 0.71 | 0.82 | 1.21 | -1.10 | -0.83 | -0.98 | 0.70 | 0.62 | 1.28 | -0.92 | -0.81 |
| Q9NRZ7 | 1-acyl-sn-glycerol-3-phosphate acyltransferase gamma | AGPAT3 | 0.96 | 2.94 | -0.61 | -0.80 | 0.67 | 0.06 | -1.75 | -0.42 | 0.84 | -0.18 | 0.33 | 1.86 |
| P10599 | Thioredoxin | TXN | 0.96 | 0.88 | -0.16 | 0.37 | 0.15 | -1.23 | -0.19 | 0.13 | 0.25 | 2.36 | -0.52 | -1.16 |
| P04196 | Histidine-rich glycoprotein | HRG | 0.96 | 1.41 | 1.06 | -0.65 | -0.69 | -0.58 | -0.65 | -0.49 | -0.61 | 1.58 | -0.63 | 1.65 |
| P23469 | Receptor-type tyrosine-protein phosphatase epsilon | PTPRE | 0.96 | 1.30 | 0.13 | -0.15 | 1.39 | -1.16 | -1.62 | 0.54 | 1.58 | -0.32 | -0.38 | -0.01 |
| Q6P6C2 | RNA demethylase ALKBH5 | ALKBH5 | 0.95 | 1.46 | 1.17 | -0.80 | -1.04 | -0.78 | -0.09 | -0.91 | -0.86 | 0.86 | 1.25 | 1.19 |
| Q5HYI7 | Metaxin-3 | MTX3 | 0.95 | 1.36 | 0.77 | 1.07 | -0.14 | -1.56 | -1.60 | 0.86 | 1.16 | -0.08 | -0.37 | -0.10 |
| Q96LD4 | E3 ubiquitin-protein ligase TRIM47 | TRIM47 | 0.95 | 1.35 | 0.76 | -0.43 | 0.30 | -0.73 | -1.37 | 1.53 | -1.41 | 0.40 | -0.18 | 1.10 |
| Q8N3F0 | Maturin | MTURN | 0.95 | 1.59 | 0.16 | 0.83 | -0.70 | 0.56 | -2.55 | 0.14 | -0.10 | 0.61 | 0.46 | 0.60 |
| Q13402 | Unconventional myosin-VIIa | MYO7A | 0.95 | 0.82 | 1.43 | -0.89 | -0.09 | -0.89 | -0.54 | 1.36 | 0.85 | -1.11 | -0.84 | 0.72 |
| P28838 | Cytosol aminopeptidase | LAP3 | 0.95 | 3.73 | -0.47 | -0.12 | -0.62 | -1.20 | -0.53 | 1.42 | -0.30 | 1.97 | -0.65 | 0.51 |
| Q9BZJ0 | Crooked neck-like protein 1 | CRNKL1 | 0.95 | 1.31 | 1.36 | -0.43 | -2.07 | -0.33 | 0.04 | 1.29 | 0.73 | -0.59 | 0.10 | -0.10 |
| P46100 | Transcriptional regulator ATRX | ATRX | 0.95 | 0.71 | 1.17 | -0.96 | -1.57 | 0.05 | 0.43 | 1.07 | 1.28 | -0.98 | 0.08 | -0.57 |
| Q9BXW6 | Oxysterol-binding protein-related protein 1 | OSBPL1A | 0.95 | 3.41 | -0.45 | -0.82 | -0.11 | 0.77 | -2.07 | 0.34 | 0.29 | -0.22 | 0.70 | 1.58 |
| O15355 | Protein phosphatase 1G | PPM1G | 0.95 | 2.91 | 0.83 | -1.38 | -0.89 | -0.33 | -0.65 | 1.59 | 0.24 | -0.44 | -0.42 | 1.46 |
| P07738 | Bisphosphoglycerate mutase | BPGM | 0.95 | 1.00 | -0.90 | 0.09 | -1.73 | 0.53 | 0.86 | -1.51 | 0.46 | 0.89 | 0.76 | 0.54 |
| P16402 | Histone H1.3 | HIST1H1D | 0.95 | 2.09 | 0.22 | -1.39 | 0.87 | 0.19 | -1.88 | 1.13 | -0.32 | 0.30 | -0.20 | 1.09 |
| P61457 | Pterin-4-alpha-carbinolamine dehydratase | PCBD1 | 0.95 | 1.32 | -0.10 | -0.35 | -0.44 | -0.22 | -0.39 | -0.29 | -0.38 | 2.83 | -0.30 | -0.37 |
| O60883 | G-protein coupled receptor 37-like 1 | GPR37L1 | 0.94 | 1.85 | -1.15 | -0.15 | -0.63 | 1.16 | -1.03 | -0.20 | -0.93 | 1.43 | 1.38 | 0.14 |
| Q09161 | Nuclear cap-binding protein subunit 1 | NCBP1 | 0.94 | 1.36 | -0.41 | -0.49 | -0.70 | 0.64 | -0.53 | -0.53 | -0.51 | -0.91 | 1.49 | 1.96 |
| Q8NI27 | THO complex subunit 2 | THOC2 | 0.94 | 2.62 | -1.09 | -1.06 | 0.63 | -1.28 | 0.53 | 1.10 | -0.17 | 1.54 | -0.70 | 0.50 |
| P20338 | Ras-related protein Rab-4A | RAB4A | 0.94 | 3.47 | -0.02 | -1.25 | -1.62 | 0.95 | -0.78 | 0.96 | 1.36 | 0.03 | 0.67 | -0.32 |
| P16401 | Histone H1.5 | HIST1H1B | 0.94 | 4.40 | -0.64 | -0.83 | 0.74 | -0.69 | -1.60 | 0.62 | 0.48 | 0.31 | -0.26 | 1.88 |
| O95831 | Apoptosis-inducing factor 1, mitochondrial | AIFM1 | 0.94 | 2.04 | -0.27 | -0.12 | -0.69 | -0.61 | -0.35 | -0.47 | -0.41 | 1.45 | -0.74 | 2.21 |
| Q13451 | Peptidyl-prolyl cis-trans isomerase FKBP5 | FKBP5 | 0.94 | 3.00 | -1.97 | -0.53 | 0.27 | 0.17 | -0.41 | 0.75 | -0.96 | 1.59 | 0.45 | 0.63 |
| P49789 | Bis(5'-adenosyl)-triphosphatase | FHIT | 0.94 | 0.96 | 1.74 | -1.33 | -0.51 | -0.61 | -0.41 | 1.10 | 1.11 | 0.24 | -0.56 | -0.77 |
| P34913 | Bifunctional epoxide hydrolase 2 | EPHX2 | 0.94 | 1.67 | -0.25 | 0.25 | -0.69 | -0.47 | -0.61 | -0.46 | -0.54 | 2.53 | 0.78 | -0.56 |
| Q8N3E9 | 1-phosphatidylinositol 4,5-bisphosphate phosphodiesterase delta-3 | PLCD3 | 0.94 | 2.25 | 0.56 | -1.49 | -1.25 | 0.65 | -0.54 | 1.60 | -0.33 | -0.63 | 0.97 | 0.43 |
| P10768 | S-formylglutathione hydrolase | ESD | 0.94 | 1.59 | -1.43 | -0.38 | 0.18 | -0.01 | -0.02 | 0.28 | -1.23 | 2.25 | 0.04 | 0.31 |
| P12931 | Proto-oncogene tyrosine-protein kinase Src | SRC | 0.93 | 2.05 | -1.56 | -0.01 | -0.94 | 0.51 | 0.06 | -0.39 | 1.03 | -0.97 | 0.54 | 1.73 |
| P01861 | Immunoglobulin heavy constant gamma 4 | IGHG4 | 0.93 | 1.23 | 0.78 | -0.79 | 0.06 | -0.70 | -0.71 | 1.79 | -0.01 | -0.98 | -0.82 | 1.39 |
| P98082 | Disabled homolog 2 | DAB2 | 0.93 | 0.98 | -0.74 | 0.96 | 0.23 | -0.68 | -0.91 | 0.40 | 0.96 | -1.13 | -0.85 | 1.77 |
| Q66K14 | TBC1 domain family member 9B | TBC1D9B | 0.93 | 1.81 | 1.14 | -0.61 | -1.98 | 0.15 | -0.48 | 1.00 | 1.41 | -0.37 | -0.04 | -0.20 |
| P00738 | Haptoglobin | HP | 0.93 | 1.41 | -1.34 | 0.11 | 1.10 | 0.08 | -1.45 | 0.35 | -1.02 | 1.54 | 0.05 | 0.57 |
| Q00587 | Cdc42 effector protein 1 | CDC42EP1 | 0.93 | 0.81 | -0.34 | -1.16 | 0.40 | 1.29 | -1.16 | 0.24 | -1.12 | 0.13 | -0.03 | 1.74 |
| Q9Y5Y2 | Cytosolic Fe-S cluster assembly factor NUBP2 | NUBP2 | 0.93 | 1.71 | 1.46 | -0.38 | -1.47 | -0.26 | -1.06 | 1.43 | -0.07 | -0.61 | -0.01 | 0.97 |
| Q09028 | Histone-binding protein RBBP4 | RBBP4 | 0.93 | 2.21 | -0.38 | -0.53 | -1.64 | 1.07 | -0.56 | 0.54 | -1.16 | 0.97 | 0.38 | 1.30 |
| P60002 | Transcription elongation factor 1 homolog | ELOF1 | 0.93 | 1.42 | 0.82 | -0.87 | -1.13 | 0.57 | -0.89 | 0.92 | -0.86 | 1.44 | -0.86 | 0.87 |
| P31749 | RAC-alpha serine/threonine-protein kinase | AKT1 | 0.93 | 1.03 | 1.23 | -1.43 | -0.47 | 0.14 | -0.65 | 1.57 | 0.90 | -0.33 | 0.20 | -1.16 |
| Q9H3F6 | BTB/POZ domain-containing adapter for CUL3-mediated RhoA deg | KCTD10 | 0.93 | 1.48 | 1.44 | -0.16 | -1.49 | -0.43 | -0.91 | 0.71 | 0.64 | 0.53 | -1.27 | 0.93 |
| Q15554 | Telomeric repeat-binding factor 2 | TERF2 | 0.93 | 2.06 | -0.56 | -0.64 | -0.91 | 0.90 | -0.75 | -0.81 | -0.74 | 0.58 | 1.82 | 1.11 |
| P30086 | Phosphatidylethanolamine-binding protein 1 | PEBP1 | 0.93 | 1.53 | -0.37 | -0.28 | 0.06 | -0.74 | -0.32 | -0.12 | -0.45 | 2.78 | -0.37 | -0.19 |
| Q7L576 | Cytoplasmic FMR1-interacting protein 1 | CYFIP1 | 0.93 | 2.68 | 0.55 | -1.79 | -0.24 | 0.77 | -1.70 | 0.90 | 0.11 | 0.02 | 0.99 | 0.39 |
| P54687 | Branched-chain-amino-acid aminotransferase, cytosolic | BCAT1 | 0.93 | 1.62 | -0.12 | -0.30 | -0.53 | -0.30 | -0.50 | -0.31 | -0.27 | 2.83 | -0.25 | -0.25 |
| P08134 | Rho-related GTP-binding protein RhoC | RHOC | 0.92 | 3.11 | -0.86 | 0.69 | -0.41 | -0.33 | -1.60 | 1.88 | -0.52 | -0.15 | 0.28 | 1.01 |
| P39748 | Flap endonuclease 1 | FEN1 | 0.92 | 1.66 | 1.31 | -2.16 | 0.04 | 0.12 | -1.05 | 0.33 | 0.83 | -0.25 | 0.82 | 0.01 |
| P00966 | Argininosuccinate synthase | ASS1 | 0.92 | 3.20 | 0.06 | 0.07 | -0.60 | -0.04 | -2.04 | 0.68 | 0.78 | -0.47 | -0.21 | 1.77 |
| P00813 | Adenosine deaminase | ADA | 0.92 | 1.15 | -0.43 | -0.60 | -0.81 | -0.62 | 1.17 | -0.56 | -0.68 | 1.91 | 1.16 | -0.54 |
| Q9NVZ3 | Adaptin ear-binding coat-associated protein 2 | NECAP2 | 0.92 | 2.05 | -0.66 | -0.49 | 1.03 | 0.03 | -1.85 | -1.00 | 0.38 | 0.72 | 0.42 | 1.41 |
| Q14232 | Translation initiation factor eIF-2B subunit alpha | EIF2B1 | 0.92 | 3.94 | -1.41 | 0.22 | -0.01 | 0.15 | -1.83 | -0.07 | 0.62 | 1.49 | -0.13 | 0.98 |
| P53611 | Geranylgeranyl transferase type-2 subunit beta | RABGGTB | 0.92 | 3.00 | 0.21 | -0.45 | -0.98 | 0.64 | -1.88 | 0.88 | 1.00 | -0.92 | 0.54 | 0.95 |
| Q9Y6X9 | ATPase MORC2 | MORC2 | 0.92 | 0.73 | 0.68 | -0.32 | -0.70 | -0.14 | -0.43 | 1.63 | 1.62 | -1.34 | -0.19 | -0.81 |
| P12956 | X-ray repair cross-complementing protein 6 | XRCC6 | 0.92 | 3.07 | 0.59 | -1.70 | -1.34 | 0.51 | -0.58 | 1.45 | -0.20 | -0.24 | 0.64 | 0.88 |
| Q7L2H7 | Eukaryotic translation initiation factor 3 subunit M | EIF3M | 0.91 | 1.09 | 0.80 | -0.64 | -1.45 | 0.02 | 0.04 | 1.54 | 1.20 | -1.22 | -0.65 | 0.37 |
| O00264 | Membrane-associated progesterone receptor component 1 | PGRMC1 | 0.91 | 1.90 | -0.81 | 0.92 | 0.50 | -0.21 | -2.31 | 0.81 | 0.39 | -0.41 | 0.19 | 0.92 |
| Q15345 | Leucine-rich repeat-containing protein 41 | LRRC41 | 0.91 | 1.39 | 1.48 | -0.74 | -0.79 | -0.70 | -0.72 | 1.26 | 0.97 | -1.00 | -0.60 | 0.86 |
| P39880 | Homeobox protein cut-like 1 | CUX1 | 0.91 | 1.56 | -0.33 | -0.67 | -0.74 | 0.71 | -0.60 | -0.50 | 2.05 | 1.33 | -0.66 | -0.58 |
| O95678 | Keratin, type II cytoskeletal 75 | KRT75 | 0.91 | 0.71 | -0.55 | 1.57 | -0.66 | -0.62 | -0.62 | -0.65 | -0.54 | 1.86 | 0.75 | -0.54 |
| P43121 | Cell surface glycoprotein MUC18 | MCAM | 0.91 | 1.40 | -0.86 | 0.95 | -0.52 | 0.02 | -1.08 | -0.95 | -0.93 | 1.45 | 1.28 | 0.64 |
| P41218 | Myeloid cell nuclear differentiation antigen | MNDA | 0.91 | 1.62 | 0.28 | -0.69 | -0.89 | 0.70 | -1.06 | 0.19 | -1.44 | 1.58 | 1.17 | 0.16 |
| Q9NR30 | Nucleolar RNA helicase 2 | DDX21 | 0.91 | 3.28 | 0.02 | -0.54 | 0.03 | 0.17 | -2.28 | -0.01 | 0.21 | 1.80 | 0.38 | 0.23 |
| Q5VZE5 | N-alpha-acetyltransferase 35, NatC auxiliary subunit | NAA35 | 0.91 | 0.85 | 0.66 | 0.57 | -1.27 | 0.23 | -1.21 | 1.00 | 1.46 | -1.38 | 0.16 | -0.22 |
| O60763 | General vesicular transport factor p115 | USO1 | 0.90 | 0.94 | 1.38 | 0.01 | -0.79 | -0.32 | -1.37 | 1.23 | 1.26 | -1.04 | -0.52 | 0.17 |
| O95671 | Probable bifunctional dTTP/UTP pyrophosphatase/methyltransfera | ASMTL | 0.90 | 1.11 | 1.57 | -1.92 | -0.59 | -0.03 | -0.29 | 0.81 | 1.08 | -0.53 | 0.33 | -0.44 |
| Q6BCY4 | NADH-cytochrome b5 reductase 2 | CYB5R2 | 0.90 | 1.16 | -0.22 | -0.33 | -0.46 | -0.35 | 0.02 | -0.39 | -0.34 | 2.82 | -0.36 | -0.39 |
| P28702 | Retinoic acid receptor RXR-beta | RXRB | 0.90 | 1.60 | 0.21 | -1.01 | -1.21 | -1.03 | 1.40 | -0.97 | 0.40 | 0.05 | 1.15 | 1.02 |
| P63208 | S-phase kinase-associated protein 1 | SKP1 | 0.90 | 1.04 | 0.17 | 0.27 | -0.62 | -1.44 | 0.43 | -0.79 | -0.12 | 2.24 | -0.61 | 0.48 |
| Q8IXI1 | Mitochondrial Rho GTPase 2 | RHOT2 | 0.90 | 1.19 | -0.38 | -0.56 | -0.66 | -0.54 | 0.79 | -0.54 | -0.53 | -0.91 | 1.28 | 2.04 |
| P09871 | Complement C1s subcomponent | C1S | 0.90 | 1.18 | 0.13 | 0.97 | -1.01 | -0.38 | -1.01 | -0.93 | 1.29 | 0.62 | -1.04 | 1.37 |
| P27824 | Calnexin | CANX | 0.90 | 2.72 | 0.34 | -0.04 | -0.48 | -0.43 | -1.72 | 1.41 | 0.56 | -1.06 | -0.02 | 1.44 |
| P18669 | Phosphoglycerate mutase 1 | PGAM1 | 0.89 | 4.13 | -1.45 | 0.52 | 0.05 | -0.23 | -1.95 | 1.01 | 0.89 | 0.44 | -0.13 | 0.86 |
| P07093 | Glia-derived nexin | SERPINE2 | 0.89 | 1.47 | -0.02 | -0.78 | -2.00 | -0.22 | 1.46 | -0.58 | 0.52 | 1.12 | 0.59 | -0.08 |
| Q5TEA3 | Uncharacterized protein C20orf194 | C20orf194 | 0.89 | 0.86 | 0.42 | 0.72 | 0.41 | 0.02 | -2.61 | 0.53 | 0.40 | -0.69 | 0.18 | 0.63 |
| P50502 | Hsc70-interacting protein | ST13 | 0.89 | 2.06 | -0.58 | -1.30 | -0.19 | -0.67 | 0.77 | 0.35 | 1.25 | 1.71 | -1.11 | -0.24 |
| P14317 | Hematopoietic lineage cell-specific protein | HCLS1 | 0.89 | 0.72 | 1.11 | 0.40 | -1.98 | -0.15 | -0.26 | 1.10 | 1.24 | -0.77 | -0.29 | -0.40 |

| | | | | | | | | | | | | | | |
|---|---|---|---|---|---|---|---|---|---|---|---|---|---|---|
| Q15661 | Tryptase alpha/beta-1 | TPSAB1 | 0.89 | 1.31 | -0.03 | -0.30 | -0.55 | -0.23 | -0.37 | 2.81 | -0.24 | -0.60 | -0.21 | -0.27 |
| O15173 | Membrane-associated progesterone receptor component 2 | PGRMC2 | 0.89 | 2.11 | -0.43 | 0.09 | -0.14 | -1.26 | -0.30 | 1.23 | -0.72 | -0.55 | -0.10 | 2.19 |
| Q14320 | Protein FAM50A | FAM50A | 0.89 | 1.59 | 0.40 | -0.55 | -1.78 | 0.31 | -0.02 | -0.65 | -0.48 | 0.45 | 0.21 | 2.09 |
| O75563 | Src kinase-associated phosphoprotein 2 | SKAP2 | 0.89 | 0.98 | 0.31 | 0.84 | -1.84 | 0.24 | -0.68 | 1.12 | 0.95 | -1.33 | -0.19 | 0.58 |
| Q9BV20 | Methylthioribose-1-phosphate isomerase | MRI1 | 0.89 | 1.96 | 1.21 | -1.14 | -1.59 | 0.32 | -0.69 | 1.38 | -0.30 | 0.66 | 0.67 | -0.51 |
| Q9Y237 | Peptidyl-prolyl cis-trans isomerase NIMA-interacting 4 | PIN4 | 0.88 | 2.02 | -0.66 | 0.06 | 0.83 | 0.19 | -2.38 | -0.53 | 0.41 | 0.75 | 1.03 | 0.31 |
| Q99816 | Tumor susceptibility gene 101 protein | TSG101 | 0.88 | 3.27 | 0.15 | -0.34 | 0.07 | -1.03 | -1.46 | 1.17 | 1.35 | -1.10 | 0.07 | 1.13 |
| O14733 | Dual specificity mitogen-activated protein kinase kinase 7 | MAP2K7 | 0.88 | 0.88 | 1.02 | -0.64 | -2.43 | 0.41 | 0.58 | 0.63 | 0.40 | -0.54 | 0.52 | 0.06 |
| Q14914 | Prostaglandin reductase 1 | PTGR1 | 0.88 | 4.52 | -0.73 | -0.45 | -0.24 | -0.01 | -1.67 | 1.09 | 0.95 | 1.57 | -0.86 | 0.35 |
| A1IGU5 | Rho guanine nucleotide exchange factor 37 | ARHGEF37 | 0.88 | 1.30 | 0.79 | -0.53 | -0.63 | -0.56 | -0.51 | 1.28 | 2.05 | -0.87 | -0.52 | -0.51 |
| Q96RT1 | Erbin | ERBIN | 0.88 | 1.98 | -1.45 | 0.96 | -0.02 | 0.39 | -1.80 | -0.41 | 0.36 | 0.05 | 0.52 | 1.42 |
| O14618 | Copper chaperone for superoxide dismutase | CCS | 0.88 | 2.44 | -0.23 | 0.72 | -1.72 | -0.11 | -0.83 | 1.62 | 0.75 | -0.99 | 0.01 | 0.78 |
| Q07954 | Prolow-density lipoprotein receptor-related protein 1 | LRP1 | 0.88 | 1.63 | 0.65 | 1.16 | -0.87 | -1.88 | -0.75 | -0.46 | 0.65 | -0.24 | 0.53 | 1.20 |
| O95394 | Phosphoacetylglucosamine mutase | PGM3 | 0.88 | 2.03 | -1.35 | -0.60 | 0.41 | 1.16 | -1.58 | 0.88 | -0.86 | 0.69 | 0.47 | 0.79 |
| P40306 | Proteasome subunit beta type-10 | PSMB10 | 0.88 | 2.51 | -0.70 | -2.02 | 0.38 | 0.25 | -0.11 | 0.67 | -0.99 | 1.44 | 0.31 | 0.78 |
| Q14571 | Inositol 1,4,5-trisphosphate receptor type 2 | ITPR2 | 0.87 | 1.39 | 0.52 | -1.43 | -2.08 | 0.27 | 1.18 | 0.70 | -0.09 | 0.16 | 0.16 | 0.61 |
| P49336 | Cyclin-dependent kinase 8 | CDK8 | 0.87 | 1.17 | 1.26 | -0.57 | -0.81 | -0.49 | -0.69 | 0.82 | -0.48 | 2.05 | -0.61 | -0.48 |
| P32455 | Guanylate-binding protein 1 | GBP1 | 0.87 | 1.21 | 0.46 | 1.08 | -0.35 | -1.16 | -1.37 | 1.34 | 0.93 | 0.05 | -1.25 | 0.26 |
| Q8NFZ8 | Cell adhesion molecule 4 | CADM4 | 0.87 | 1.03 | -0.91 | 0.23 | 0.60 | 0.06 | -1.18 | -0.88 | 0.51 | 2.18 | 0.14 | -0.76 |
| Q96N67 | Dedicator of cytokinesis protein 7 | DOCK7 | 0.87 | 1.52 | -2.35 | 0.69 | -0.76 | 0.72 | 0.09 | 1.14 | 0.57 | -0.35 | -0.10 | 0.35 |
| P13010 | X-ray repair cross-complementing protein 5 | XRCC5 | 0.87 | 2.05 | 0.39 | -1.49 | -0.75 | 0.52 | -0.60 | 1.42 | -1.44 | 0.50 | 0.76 | 0.71 |
| Q9UIA0 | Cytohesin-4 | CYTH4 | 0.87 | 0.79 | 0.92 | 0.45 | -0.86 | -0.70 | -0.77 | 1.45 | 1.52 | -1.02 | -0.19 | -0.79 |
| Q9UKF7 | Cytoplasmic phosphatidylinositol transfer protein 1 | PITPNC1 | 0.87 | 1.18 | -0.02 | 0.86 | -1.05 | 0.35 | -1.44 | 0.90 | 0.80 | -1.65 | 0.73 | 0.52 |
| P54198 | Protein HIRA | HIRA | 0.87 | 1.26 | 1.35 | -0.58 | -0.90 | -0.52 | -0.72 | 1.17 | 1.75 | -0.37 | -0.64 | -0.53 |
| Q8N5D0 | WD and tetratricopeptide repeats protein 1 | WDTC1 | 0.87 | 1.24 | 1.07 | 0.37 | -1.09 | -0.66 | -1.05 | 0.92 | 1.80 | -0.07 | -0.47 | -0.82 |
| Q13618 | Cullin-3 | CUL3 | 0.87 | 1.34 | 1.18 | -0.46 | -0.39 | -0.65 | -1.12 | 1.55 | 1.51 | -0.44 | -0.59 | -0.58 |
| O14497 | AT-rich interactive domain-containing protein 1A | ARID1A | 0.87 | 2.41 | -0.55 | -0.67 | 0.41 | -0.55 | -0.87 | -0.83 | -0.88 | 0.84 | 1.49 | 1.62 |
| Q71F56 | Mediator of RNA polymerase II transcription subunit 13-like | MED13L | 0.87 | 1.48 | -0.09 | -0.26 | -0.60 | -0.32 | -0.35 | -0.19 | -0.40 | 2.82 | -0.38 | -0.22 |
| Q13642 | Four and a half LIM domains protein 1 | FHL1 | 0.87 | 2.01 | 0.32 | -0.72 | -1.83 | -0.02 | 0.34 | 1.55 | 0.62 | -1.19 | 0.11 | 0.83 |
| O75083 | WD repeat-containing protein 1 | WDR1 | 0.87 | 2.83 | -1.54 | -0.25 | -0.73 | 0.40 | -0.29 | 1.99 | -0.45 | -0.52 | 0.26 | 1.13 |
| Q9H0W9 | Ester hydrolase C11orf54 | C11orf54 | 0.86 | 1.15 | 1.13 | -0.54 | -0.68 | -0.55 | -0.65 | 0.55 | -0.55 | 2.27 | -0.45 | -0.53 |
| O75190 | DnaJ homolog subfamily B member 6 | DNAJB6 | 0.86 | 1.58 | 0.00 | -1.36 | -0.59 | 0.24 | 0.05 | -0.71 | -1.04 | 2.00 | 0.42 | 0.98 |
| Q9P0V9 | Septin-10 | SEPTIN10 | 0.86 | 3.28 | -0.46 | -1.45 | 0.57 | -1.57 | 0.31 | 1.42 | 0.83 | -0.08 | -0.48 | 0.91 |
| Q8TAD8 | Smad nuclear-interacting protein 1 | SNIP1 | 0.86 | 1.69 | 1.00 | -1.05 | -1.26 | -0.55 | 0.16 | 1.39 | 1.40 | 0.11 | -0.18 | -1.02 |
| P30043 | Flavin reductase (NADPH) | BLVRB | 0.86 | 2.30 | 0.15 | -0.44 | -0.95 | 0.10 | -1.01 | 1.52 | 1.11 | -1.60 | 0.36 | 0.77 |
| P10398 | Serine/threonine-protein kinase A-Raf | ARAF | 0.86 | 1.22 | -0.64 | 0.03 | -1.15 | -0.90 | 1.33 | -0.82 | -0.81 | 1.16 | 0.49 | 1.33 |
| P21810 | Biglycan | BGN | 0.86 | 1.06 | 0.58 | 0.42 | 0.33 | -0.99 | -1.55 | -1.47 | 0.46 | 0.74 | 0.04 | 1.44 |
| P68402 | Platelet-activating factor acetylhydrolase IB subunit beta | PAFAH1B2 | 0.86 | 5.06 | -0.09 | -1.75 | -0.48 | 0.23 | -1.15 | -0.16 | 1.04 | 1.73 | 0.34 | 0.29 |
| Q86X55 | Histone-arginine methyltransferase CARM1 | CARM1 | 0.86 | 2.99 | -0.42 | 0.61 | -0.81 | -0.21 | -1.62 | 1.63 | 1.28 | -0.82 | 0.42 | -0.05 |
| Q9Y333 | U6 snRNA-associated Sm-like protein LSm2 | LSM2 | 0.86 | 1.27 | -0.15 | -0.23 | -0.55 | -0.17 | -0.36 | 2.81 | -0.24 | -0.61 | -0.23 | -0.28 |
| Q8TF39 | Zinc finger protein 483 | ZNF483 | 0.86 | 1.34 | 1.36 | -0.14 | -1.69 | 0.54 | -1.54 | 0.67 | 0.21 | -0.54 | 0.87 | 0.27 |
| Q9P0K9 | DOMON domain-containing protein FRRS1L | FRRS1L | 0.86 | 1.29 | 1.18 | -0.65 | -0.80 | -0.59 | -0.55 | 1.28 | -0.50 | 1.81 | -0.69 | -0.51 |
| Q9UBC2 | Epidermal growth factor receptor substrate 15-like 1 | EPS15L1 | 0.86 | 1.23 | 0.87 | 0.22 | -1.25 | -0.47 | -0.73 | 0.61 | 2.04 | -1.15 | 0.00 | -0.14 |
| Q8TCU6 | Phosphatidylinositol 3,4,5-trisphosphate-dependent Rac exchanger | PREX1 | 0.86 | 0.80 | 1.30 | -0.51 | -1.41 | -0.32 | -0.03 | 1.50 | 1.05 | -1.24 | -0.10 | -0.24 |
| Q15293 | Reticulocalbin-1 | RCN1 | 0.86 | 0.93 | -0.67 | 0.59 | 0.58 | -0.12 | -1.46 | 1.01 | 0.86 | -1.84 | 0.47 | 0.60 |
| P78371 | T-complex protein 1 subunit beta | CCT2 | 0.85 | 2.80 | 0.26 | -0.21 | -1.20 | -0.37 | -0.91 | 0.35 | 0.74 | 2.28 | -0.76 | -0.19 |
| P22234 | Multifunctional protein ADE2 | PAICS | 0.85 | 1.48 | -0.10 | -1.85 | 0.32 | -0.92 | 1.01 | 1.26 | -0.25 | 1.28 | -0.40 | -0.34 |
| P53602 | Diphosphomevalonate decarboxylase | MVD | 0.85 | 2.92 | -1.69 | 0.41 | -0.47 | 0.75 | -1.46 | 0.44 | 1.16 | -0.60 | 0.95 | 0.50 |
| P49419 | Alpha-aminoadipic semialdehyde dehydrogenase | ALDH7A1 | 0.85 | 1.27 | -0.33 | -0.71 | -0.16 | -0.37 | 0.14 | 0.64 | -1.34 | 2.43 | -0.01 | -0.28 |
| P12532 | Creatine kinase U-type, mitochondrial | CKMT1A | 0.85 | 1.70 | -0.81 | -0.74 | -0.16 | -0.54 | 0.49 | -0.16 | -0.39 | 2.64 | -0.31 | -0.02 |
| P24534 | Elongation factor 1-beta | EEF1B2 | 0.85 | 2.41 | 0.13 | -0.45 | -1.01 | -0.36 | -0.57 | 1.47 | 1.17 | -1.54 | -0.02 | 1.18 |
| Q8N944 | APC membrane recruitment protein 3 | AMER3 | 0.85 | 1.79 | -0.25 | 0.10 | -0.80 | -0.40 | -0.51 | 2.43 | 0.97 | -0.91 | -0.34 | -0.29 |
| Q99973 | Telomerase protein component 1 | TEP1 | 0.85 | 1.19 | 1.40 | -0.88 | -0.10 | -0.89 | -0.83 | 1.26 | 0.92 | 0.88 | -0.84 | -0.91 |
| Q9HCG8 | Pre-mRNA-splicing factor CWC22 homolog | CWC22 | 0.85 | 1.99 | 0.51 | -0.60 | -0.84 | -0.50 | -0.53 | 1.33 | 1.94 | -1.10 | 0.33 | -0.54 |
| O14672 | Disintegrin and metalloproteinase domain-containing protein 10 | ADAM10 | 0.85 | 1.60 | -0.20 | -0.40 | -0.64 | -0.21 | -0.27 | -0.38 | -0.21 | 2.82 | -0.34 | -0.17 |
| P49720 | Proteasome subunit beta type-3 | PSMB3 | 0.85 | 2.05 | -0.62 | -1.85 | -0.03 | -0.41 | 0.96 | 0.04 | -0.63 | 1.86 | 0.20 | 0.47 |
| Q9UBB6 | Neurochondrin | NCDN | 0.85 | 2.86 | -0.83 | 0.02 | 0.70 | -0.76 | -1.54 | 0.61 | 1.82 | -0.26 | -0.63 | 0.85 |
| P05141 | ADP/ATP translocase 2 | SLC25A5 | 0.85 | 1.63 | 1.92 | -1.26 | -1.14 | -0.95 | -0.28 | 0.86 | -0.13 | 0.43 | -0.14 | 0.69 |
| P30530 | Tyrosine-protein kinase receptor UFO | AXL | 0.85 | 1.17 | -0.38 | -0.51 | -0.69 | 0.95 | -0.67 | -0.53 | -0.56 | -0.87 | 1.74 | 1.54 |
| O00422 | Histone deacetylase complex subunit SAP18 | SAP18 | 0.85 | 0.90 | 1.27 | -0.50 | -0.61 | -0.58 | -0.63 | 1.61 | 1.43 | -0.86 | -0.56 | -0.57 |
| P29762 | Cellular retinoic acid-binding protein 1 | CRABP1 | 0.85 | 1.04 | -0.06 | -0.18 | -0.35 | -0.61 | -0.02 | 1.14 | 1.71 | -1.58 | -0.95 | 0.91 |
| P55081 | Microfibrillar-associated protein 1 | MFAP1 | 0.85 | 1.08 | -0.36 | -0.96 | -1.33 | 0.58 | 0.84 | -0.66 | -0.88 | 1.62 | 1.15 | -0.01 |
| P25787 | Proteasome subunit alpha type-2 | PSMA2 | 0.85 | 3.25 | -1.16 | -2.03 | 0.14 | -0.24 | 0.66 | 0.17 | 0.26 | 1.50 | -0.13 | 0.82 |
| Q96CB8 | Integrator complex subunit 12 | INTS12 | 0.85 | 1.10 | 0.04 | -1.21 | -0.09 | -0.70 | 0.71 | 1.68 | 1.23 | -1.46 | 0.08 | -0.28 |
| P48637 | Glutathione synthetase | GSS | 0.85 | 1.16 | 0.56 | 0.35 | -1.74 | -1.02 | 0.55 | 0.75 | 0.70 | 1.39 | -0.67 | -0.88 |
| Q13325 | Interferon-induced protein with tetratricopeptide repeats 5 | IFIT5 | 0.85 | 1.24 | 0.03 | -1.44 | -1.66 | 0.92 | 0.77 | 0.63 | 0.47 | -1.07 | 0.87 | 0.47 |
| Q15435 | Protein phosphatase 1 regulatory subunit 7 | PPP1R7 | 0.85 | 2.47 | 0.29 | -1.20 | -0.63 | 0.74 | -1.38 | -1.17 | 0.59 | 1.25 | 1.07 | 0.44 |
| P55036 | 26S proteasome non-ATPase regulatory subunit 4 | PSMD4 | 0.85 | 1.87 | 0.84 | 0.21 | -1.77 | 0.41 | -1.53 | 0.38 | 0.39 | 1.42 | 0.16 | -0.50 |
| Q86UX7 | Fermitin family homolog 3 | FERMT3 | 0.84 | 2.20 | -0.54 | 0.81 | -0.69 | -0.34 | -1.29 | 2.30 | -0.19 | -0.25 | -0.37 | 0.55 |
| O75821 | Eukaryotic translation initiation factor 3 subunit G | EIF3G | 0.84 | 0.98 | 1.39 | -0.08 | -1.04 | -0.24 | -1.16 | 1.42 | 1.07 | -1.10 | -0.43 | 0.17 |
| P17661 | Desmin | DES | 0.84 | 0.54 | 1.31 | -0.71 | -0.82 | 0.28 | -0.74 | 1.24 | 1.56 | -0.82 | -0.65 | -0.64 |
| Q6GMV2 | SET and MYND domain-containing protein 5 | SMYD5 | 0.84 | 1.83 | 1.29 | -1.23 | -0.62 | -0.46 | -0.77 | 1.47 | 1.40 | -0.44 | -0.54 | -0.10 |
| P30038 | Delta-1-pyrroline-5-carboxylate dehydrogenase, mitochondrial | ALDH4A1 | 0.84 | 1.85 | -1.23 | 0.16 | 0.23 | -0.78 | -0.22 | 2.00 | -0.84 | 1.35 | -0.38 | -0.28 |
| Q3KQV9 | UDP-N-acetylhexosamine pyrophosphorylase-like protein 1 | UAP1L1 | 0.84 | 3.36 | -0.16 | -2.26 | 0.86 | -0.60 | -0.58 | 1.10 | 0.34 | 0.31 | -0.01 | 1.00 |
| P06132 | Uroporphyrinogen decarboxylase | UROD | 0.84 | 1.50 | -0.10 | -0.37 | -0.50 | -0.39 | -0.28 | -0.40 | -0.24 | 2.83 | -0.32 | -0.23 |
| P48059 | LIM and senescent cell antigen-like-containing domain protein 1 | LIMS1 | 0.84 | 3.97 | -1.92 | -0.34 | -1.09 | 0.31 | 0.15 | 0.97 | -0.59 | 0.24 | 1.14 | 1.12 |
| Q9Y6Y1 | Calmodulin-binding transcription activator 1 | CAMTA1 | 0.84 | 0.80 | 1.44 | -0.58 | -0.72 | -0.51 | -0.58 | 1.34 | 1.55 | -0.80 | -0.56 | -0.56 |
| Q9UHL4 | Dipeptidyl peptidase 2 | DPP7 | 0.84 | 0.99 | 0.71 | -0.43 | -0.58 | -0.39 | -0.48 | -0.40 | -0.47 | 2.64 | -0.49 | -0.11 |
| Q6PJ69 | Tripartite motif-containing protein 65 | TRIM65 | 0.84 | 0.60 | -1.03 | 0.52 | 0.45 | 0.44 | -1.14 | -1.15 | -1.15 | 1.19 | 0.65 | 1.22 |
| Q5VZK9 | F-actin-uncapping protein LRRC16A | CARMIL1 | 0.84 | 1.47 | 1.23 | 0.23 | -1.42 | -0.92 | -0.66 | 0.49 | 0.55 | 0.24 | -1.20 | 1.45 |
| P12268 | Inosine-5'-monophosphate dehydrogenase 2 | IMPDH2 | 0.84 | 1.22 | 0.23 | 0.04 | -1.16 | 0.24 | -0.72 | 2.18 | 0.41 | -1.44 | 0.06 | 0.17 |
| P51149 | Ras-related protein Rab-7a | RAB7A | 0.84 | 1.40 | 1.30 | -1.21 | -0.31 | 0.04 | -1.30 | 1.98 | -0.31 | -0.17 | 0.11 | -0.13 |
| P08603 | Complement factor H | CFH | 0.84 | 1.06 | -1.34 | -1.44 | 1.02 | 0.85 | -0.30 | -0.20 | -0.96 | 1.27 | 0.74 | 0.36 |

| | | | | | | | | | | | | | | |
|---|---|---|---|---|---|---|---|---|---|---|---|---|---|---|
| P00736 | Complement C1r subcomponent | C1R | 0.84 | 1.62 | -0.56 | 0.81 | -1.14 | 0.15 | -0.94 | 1.23 | -0.87 | -0.64 | 0.18 | 1.77 |
| Q9Y508 | E3 ubiquitin-protein ligase RNF114 | RNF114 | 0.84 | 0.95 | 0.50 | -0.37 | -1.51 | 0.70 | -0.42 | 1.26 | -0.15 | -1.64 | 0.73 | 0.90 |
| Q9NVS9 | Pyridoxine-5'-phosphate oxidase | PNPO | 0.84 | 1.07 | -1.16 | 0.42 | -0.50 | 0.34 | -0.34 | 0.55 | -1.92 | 1.58 | 0.45 | 0.58 |
| Q96Q11 | CCA tRNA nucleotidyltransferase 1, mitochondrial | TRNT1 | 0.84 | 1.28 | -1.20 | -0.12 | 0.26 | 1.03 | -1.36 | -1.39 | 0.59 | 0.62 | 1.37 | 0.20 |
| P30622 | CAP-Gly domain-containing linker protein 1 | CLIP1 | 0.84 | 0.87 | 0.52 | 0.37 | -1.22 | -0.99 | 0.28 | 1.51 | 1.20 | -1.37 | -0.60 | 0.30 |
| Q96JM3 | Chromosome alignment-maintaining phosphoprotein 1 | CHAMP1 | 0.83 | 0.71 | 1.08 | -1.22 | -1.41 | 0.38 | 0.30 | 0.98 | 0.43 | -1.49 | 0.08 | 0.88 |
| P01602 | Immunoglobulin kappa variable 1-5 | IGKV1-5 | 0.83 | 1.15 | -0.01 | -0.26 | -0.54 | -0.15 | -0.37 | 2.80 | -0.22 | -0.60 | -0.34 | -0.32 |
| P13693 | Translationally-controlled tumor protein | TPT1 | 0.83 | 4.11 | -0.39 | 0.64 | -0.65 | -0.90 | -1.62 | 0.91 | 0.26 | 1.61 | -0.66 | 0.81 |
| P62937 | Peptidyl-prolyl cis-trans isomerase A | PPIA | 0.83 | 2.64 | -1.63 | 0.48 | 0.77 | -0.05 | -1.99 | 0.51 | 0.87 | 0.10 | 0.34 | 0.62 |
| P15121 | Aldo-keto reductase family 1 member B1 | AKR1B1 | 0.83 | 1.32 | -0.94 | -1.56 | 1.02 | 0.80 | -0.74 | 0.38 | -1.08 | 1.01 | 1.05 | 0.07 |
| P55884 | Eukaryotic translation initiation factor 3 subunit B | EIF3B | 0.83 | 2.31 | -1.95 | 0.90 | -0.64 | 0.07 | -0.49 | 0.77 | -0.64 | 0.02 | 0.33 | 1.62 |
| P40121 | Macrophage-capping protein | CAPG | 0.83 | 1.31 | -1.64 | 0.98 | -0.75 | 0.53 | -0.53 | 1.36 | -0.28 | -1.09 | 0.53 | 0.90 |
| P49736 | DNA replication licensing factor MCM2 | MCM2 | 0.83 | 1.01 | 0.28 | 0.25 | -1.18 | -1.09 | 0.57 | 0.27 | -0.04 | 2.17 | -0.16 | -1.07 |
| Q96MU7 | YTH domain-containing protein 1 | YTHDC1 | 0.83 | 0.96 | 0.95 | -1.56 | -0.46 | -0.61 | 0.56 | 0.79 | 1.16 | 0.77 | -0.16 | -1.46 |
| Q13501 | Sequestosome-1 | SQSTM1 | 0.83 | 1.55 | -1.14 | 0.00 | -0.21 | -0.58 | 0.30 | -0.52 | 0.51 | 2.50 | -0.60 | -0.26 |
| Q66PJ3 | ADP-ribosylation factor-like protein 6-interacting protein 4 | ARL6IP4 | 0.82 | 1.36 | 0.15 | -2.05 | -1.00 | -0.07 | 1.48 | 0.24 | 1.14 | 0.40 | -0.07 | -0.22 |
| Q86T65 | Disheveled-associated activator of morphogenesis 2 | DAAM2 | 0.82 | 2.31 | -2.10 | -0.24 | -0.20 | 1.04 | -0.61 | -0.49 | 0.38 | 1.00 | 1.29 | -0.05 |
| Q07890 | Son of sevenless homolog 2 | SOS2 | 0.82 | 0.65 | 0.70 | 1.05 | -1.31 | 0.00 | -1.25 | 0.72 | 0.99 | -1.46 | -0.12 | 0.68 |
| Q8IZ81 | ELMO domain-containing protein 2 | ELMOD2 | 0.82 | 1.54 | 0.31 | -0.07 | -2.44 | 0.29 | 0.30 | 1.17 | -0.36 | -0.55 | 0.50 | 0.85 |
| Q7Z7L1 | Schlafen family member 11 | SLFN11 | 0.82 | 1.12 | -0.06 | -0.30 | -0.46 | -0.20 | -0.29 | -0.25 | -0.24 | -0.68 | -0.33 | 2.81 |
| P21953 | 2-oxoisovalerate dehydrogenase subunit beta, mitochondrial | BCKDHB | 0.82 | 1.90 | 0.42 | -1.37 | -0.17 | 0.63 | -1.36 | 0.57 | 0.80 | 0.58 | 1.24 | -1.35 |
| P37837 | Transaldolase | TALDO1 | 0.82 | 1.27 | -0.49 | -0.50 | -0.06 | -0.01 | -0.40 | -0.56 | -0.48 | 2.74 | 0.23 | -0.48 |
| Q13424 | Alpha-1-syntrophin | SNTA1 | 0.82 | 2.38 | -1.97 | -0.90 | 0.57 | 0.14 | 0.02 | 0.41 | -0.88 | 1.57 | 0.59 | 0.44 |
| Q9NRX1 | RNA-binding protein PNO1 | PNO1 | 0.82 | 0.85 | 0.52 | -1.01 | -1.14 | 0.65 | -0.04 | 1.77 | 0.52 | -1.15 | 0.73 | -0.85 |
| O75947 | ATP synthase subunit d, mitochondrial | ATP5PD | 0.82 | 0.84 | 1.37 | -0.71 | -0.82 | -0.66 | -0.19 | 1.36 | 1.53 | -0.87 | -0.37 | -0.64 |
| Q93100 | Phosphorylase b kinase regulatory subunit beta | PHKB | 0.82 | 1.24 | -0.02 | 0.31 | -1.58 | 0.39 | -0.46 | 1.71 | -1.51 | 0.21 | 0.07 | 0.88 |
| P36955 | Pigment epithelium-derived factor | SERPINF1 | 0.82 | 1.27 | 1.41 | 0.03 | -1.49 | -0.76 | -0.56 | 0.84 | 0.14 | 1.39 | -1.11 | 0.12 |
| O43414 | ERI1 exoribonuclease 3 | ERI3 | 0.82 | 1.88 | 0.92 | -1.11 | -1.49 | 0.85 | -1.02 | -0.82 | 1.00 | 0.17 | 0.99 | 0.51 |
| Q9Y3A5 | Ribosome maturation protein SBDS | SBDS | 0.82 | 2.92 | -0.21 | -1.38 | 0.67 | 0.12 | -1.63 | 0.93 | 1.72 | -0.24 | 0.20 | -0.18 |
| Q9BXC9 | Bardet-Biedl syndrome 2 protein | BBS2 | 0.81 | 1.46 | 0.54 | 0.21 | -2.51 | 0.06 | 0.15 | 0.79 | -0.75 | 0.99 | 0.12 | 0.41 |
| Q12765 | Secernin-1 | SCRN1 | 0.81 | 4.08 | -1.30 | -0.52 | 0.25 | 0.43 | -1.81 | 1.74 | 0.05 | 0.40 | 0.49 | 0.27 |
| Q9NTM9 | Copper homeostasis protein cutC homolog | CUTC | 0.81 | 4.12 | 0.85 | -1.30 | -1.72 | -0.40 | -0.41 | 1.14 | 1.21 | -0.10 | 0.71 | 0.02 |
| Q9HCS7 | Pre-mRNA-splicing factor SYF1 | XAB2 | 0.81 | 1.81 | 0.86 | -1.95 | -0.17 | -0.61 | 0.09 | 0.78 | 1.44 | 0.72 | -0.25 | -0.90 |
| Q86YQ8 | Copine-8 | CPNE8 | 0.81 | 1.88 | -0.23 | -0.43 | -0.53 | -0.36 | -0.39 | -0.34 | 2.16 | -0.98 | -0.41 | 1.51 |
| Q9UBQ5 | Eukaryotic translation initiation factor 3 subunit K | EIF3K | 0.81 | 0.96 | 1.36 | -1.26 | 0.04 | 0.01 | -1.26 | 1.11 | -1.18 | -0.27 | 0.37 | 1.08 |
| Q92609 | TBC1 domain family member 5 | TBC1D5 | 0.81 | 0.99 | 1.38 | 0.23 | -1.32 | -0.44 | -0.99 | -1.15 | 1.22 | 0.36 | -0.32 | 1.04 |
| O95210 | Starch-binding domain-containing protein 1 | STBD1 | 0.81 | 0.81 | -0.60 | 0.57 | -1.59 | 0.87 | -0.21 | -0.04 | 1.37 | 0.37 | 0.80 | -1.53 |
| O43924 | Retinal rod rhodopsin-sensitive cGMP 3',5'-cyclic phosphodiestera | PDE6D | 0.81 | 2.61 | -0.41 | -1.58 | -0.01 | 0.47 | -0.75 | 0.44 | -1.18 | 1.04 | 0.30 | 1.68 |
| Q96A65 | Exocyst complex component 4 | EXOC4 | 0.81 | 1.67 | 1.01 | 0.15 | -2.15 | 0.29 | -1.02 | 0.50 | 1.24 | -0.52 | 0.35 | 0.16 |
| P55263 | Adenosine kinase | ADK | 0.80 | 0.99 | -0.43 | -0.81 | -0.90 | 0.47 | 0.51 | -1.86 | 0.03 | 1.49 | 0.57 | 0.93 |
| O00505 | Importin subunit alpha-4 | KPNA3 | 0.80 | 3.47 | -1.22 | -1.41 | -0.92 | 0.13 | 0.74 | 1.02 | 0.04 | -0.67 | 1.21 | 1.08 |
| P61513 | 60S ribosomal protein L37a | RPL37A | 0.80 | 4.31 | 0.45 | -1.51 | -1.42 | 0.14 | -0.66 | 1.04 | 1.38 | -0.46 | 0.13 | 0.91 |
| Q86UK7 | E3 ubiquitin-protein ligase ZNF598 | ZNF598 | 0.80 | 1.59 | 1.46 | -0.27 | -2.29 | -0.39 | -0.20 | 0.42 | 0.89 | -0.32 | 0.43 | 0.28 |
| Q96ME1 | F-box/LRR-repeat protein 18 | FBXL18 | 0.80 | 1.19 | -0.72 | 0.72 | -1.06 | 0.63 | -0.89 | -0.91 | 0.83 | 1.79 | 0.45 | -0.84 |
| P51606 | N-acylglucosamine 2-epimerase | RENBP | 0.80 | 1.19 | 1.18 | 0.24 | -0.66 | -1.01 | -1.06 | 0.65 | -0.99 | 1.30 | -0.78 | 1.13 |
| Q9BSJ8 | Extended synaptotagmin-1 | ESYT1 | 0.80 | 3.37 | -0.81 | 0.32 | -0.48 | 0.20 | -1.86 | 1.99 | -0.30 | 0.01 | 0.32 | 0.61 |
| Q9UBU9 | Nuclear RNA export factor 1 | NXF1 | 0.80 | 1.17 | 0.23 | -1.35 | -0.50 | -0.45 | 0.77 | -0.40 | -1.15 | 2.06 | 0.24 | 0.55 |
| P10644 | cAMP-dependent protein kinase type I-alpha regulatory subunit | PRKAR1A | 0.80 | 2.17 | -0.31 | 0.21 | -0.52 | 0.12 | -1.54 | 1.94 | -0.27 | -1.06 | 0.40 | 1.04 |
| P02675 | Fibrinogen beta chain | FGB | 0.80 | 3.77 | -1.13 | -0.47 | 0.87 | -1.11 | -0.95 | 0.75 | 1.12 | 0.91 | -0.98 | 0.99 |
| P28799 | Progranulin | GRN | 0.80 | 1.56 | -1.00 | -1.58 | 0.63 | -0.65 | 0.99 | 0.22 | -0.52 | 1.28 | -0.57 | 1.20 |
| Q9UGK8 | Secretion-regulating guanine nucleotide exchange factor | SERGEF | 0.80 | 1.17 | 1.29 | -0.53 | -0.84 | -0.59 | -0.63 | -0.55 | -0.62 | 1.64 | -0.55 | 1.38 |
| Q29RF7 | Sister chromatid cohesion protein PDS5 homolog A | PDS5A | 0.80 | 0.83 | 0.83 | -0.31 | -0.04 | -0.28 | -1.20 | 1.27 | 1.58 | -1.57 | 0.06 | -0.33 |
| P49116 | Nuclear receptor subfamily 2 group C member 2 | NR2C2 | 0.80 | 1.41 | -0.21 | -0.92 | 0.03 | 0.05 | -0.50 | 0.34 | -0.21 | 2.63 | -0.75 | -0.46 |
| Q9HBG6 | Intraflagellar transport protein 122 homolog | IFT122 | 0.80 | 1.02 | 1.16 | -0.83 | -2.07 | 0.33 | 0.23 | 0.70 | -0.32 | -0.58 | 0.11 | 1.26 |
| Q9UH62 | Armadillo repeat-containing X-linked protein 3 | ARMCX3 | 0.80 | 2.14 | -0.08 | -0.59 | -0.61 | -0.47 | -0.37 | -0.33 | -0.58 | 0.81 | -0.36 | 2.59 |
| Q9NVT9 | Armadillo repeat-containing protein 1 | ARMC1 | 0.80 | 0.70 | 1.67 | -0.66 | -0.70 | -0.59 | -0.59 | -0.55 | -0.60 | 1.38 | -0.64 | 1.28 |
| Q9GZM7 | Tubulointerstitial nephritis antigen-like | TINAGL1 | 0.80 | 0.70 | -0.97 | -0.65 | 1.84 | -0.99 | -0.09 | -0.99 | 0.79 | 0.47 | -0.49 | 1.09 |
| P30626 | Sorcin | SRI | 0.80 | 2.91 | 0.32 | -1.19 | -0.66 | -1.23 | 0.32 | 1.16 | 1.58 | 0.93 | -0.60 | -0.63 |
| Q00059 | Transcription factor A, mitochondrial | TFAM | 0.80 | 1.11 | 1.28 | -1.22 | 0.24 | -0.55 | -0.99 | 0.90 | 1.31 | 0.73 | -0.70 | -0.99 |
| O75340 | Programmed cell death protein 6 | PDCD6 | 0.80 | 3.55 | -1.38 | -1.37 | -0.60 | -0.09 | 0.74 | 0.03 | -0.29 | 1.57 | 0.08 | 1.32 |
| Q13362 | Serine/threonine-protein phosphatase 2A 56 kDa regulatory subuni | PPP2R5C | 0.79 | 1.28 | -0.11 | -0.31 | -0.53 | -0.27 | -0.24 | -0.24 | 2.81 | -0.66 | -0.27 | -0.17 |
| P98175 | RNA-binding protein 10 | RBM10 | 0.79 | 1.01 | 1.04 | -0.87 | -2.34 | 0.48 | 0.50 | 0.46 | 0.98 | -0.13 | 0.05 | -0.17 |
| O95398 | Rap guanine nucleotide exchange factor 3 | RAPGEF3 | 0.79 | 0.81 | -0.40 | -0.55 | -0.73 | 1.29 | -0.58 | 1.73 | -0.58 | -0.80 | 1.27 | -0.64 |
| Q07157 | Tight junction protein ZO-1 | TJP1 | 0.79 | 2.00 | -2.14 | -0.25 | 0.85 | 0.61 | -1.03 | -0.37 | 0.03 | 0.40 | 0.70 | 1.19 |
| Q07866 | Kinesin light chain 1 | KLC1 | 0.79 | 1.95 | -2.05 | 0.67 | -1.14 | 0.55 | 0.07 | -0.58 | 0.84 | 0.09 | 0.30 | 1.26 |
| P55084 | Trifunctional enzyme subunit beta, mitochondrial | HADHB | 0.79 | 0.63 | 1.39 | -0.46 | -0.16 | 0.04 | -1.59 | 0.98 | 1.42 | -1.02 | -0.06 | -0.52 |
| P02760 | Protein AMBP | AMBP | 0.79 | 1.47 | -0.19 | -0.21 | -0.60 | -0.28 | -0.33 | -0.40 | -0.29 | 2.82 | -0.34 | -0.18 |
| P53778 | Mitogen-activated protein kinase 12 | MAPK12 | 0.79 | 1.02 | -0.37 | -0.65 | -0.78 | 1.21 | -0.59 | -0.48 | -0.59 | -0.83 | 1.45 | 1.63 |
| P49773 | Histidine triad nucleotide-binding protein 1 | HINT1 | 0.79 | 1.94 | -0.92 | -1.30 | 0.44 | -0.16 | 0.04 | -0.39 | 0.43 | 2.36 | 0.06 | -0.57 |
| P10809 | 60 kDa heat shock protein, mitochondrial | HSPD1 | 0.79 | 1.65 | -0.63 | -0.83 | 0.54 | -0.78 | -0.01 | 1.63 | 1.11 | 0.97 | -0.66 | -1.33 |
| A0AV96 | RNA-binding protein 47 | RBM47 | 0.79 | 1.14 | -0.47 | 0.57 | -0.74 | -0.51 | -0.15 | -0.57 | 1.71 | -0.93 | -0.66 | 1.75 |
| P35573 | Glycogen debranching enzyme | AGL | 0.79 | 1.46 | 1.83 | -1.08 | -1.12 | -0.09 | -1.10 | 1.41 | 0.02 | 0.11 | 0.06 | -0.04 |
| Q96GG9 | DCN1-like protein 1 | DCUN1D1 | 0.78 | 2.73 | -1.51 | -0.78 | 0.60 | 0.35 | -0.99 | -0.09 | -0.87 | 1.10 | 0.69 | 1.49 |
| P28062 | Proteasome subunit beta type-8 | PSMB8 | 0.78 | 1.68 | -1.19 | -1.79 | 0.63 | 0.24 | 0.42 | 0.53 | -1.05 | 1.42 | 0.50 | 0.30 |
| O94985 | Calsyntenin-1 | CLSTN1 | 0.78 | 0.79 | 1.24 | 0.09 | -1.88 | -0.15 | -0.24 | 1.23 | 0.93 | 0.30 | -0.41 | -1.10 |
| Q09666 | Neuroblast differentiation-associated protein AHNAK | AHNAK | 0.78 | 0.86 | -0.03 | -1.86 | 1.57 | -0.55 | -0.16 | 1.04 | 0.07 | -0.64 | -0.46 | 1.02 |
| P51888 | Prolargin | PRELP | 0.78 | 2.48 | -0.25 | -0.59 | -0.51 | -0.29 | -0.70 | 1.26 | -0.63 | -1.21 | 1.39 | 1.54 |
| Q8N6Y2 | Leucine-rich repeat-containing protein 17 | LRRC17 | 0.78 | 1.39 | -0.14 | -0.25 | -0.54 | -0.24 | -0.38 | -0.35 | -0.27 | 2.83 | -0.39 | -0.27 |
| Q9BX67 | Junctional adhesion molecule C | JAM3 | 0.78 | 1.46 | -0.12 | -0.39 | -0.51 | -0.31 | -0.29 | -0.34 | -0.23 | 2.83 | -0.31 | -0.33 |
| P30084 | Enoyl-CoA hydratase, mitochondrial | ECHS1 | 0.78 | 1.14 | -0.48 | 0.20 | 1.02 | -1.19 | -0.82 | -0.13 | -0.60 | 2.12 | -0.65 | 0.54 |
| P55011 | Solute carrier family 12 member 2 | SLC12A2 | 0.78 | 2.09 | 0.07 | 1.20 | -1.59 | -0.26 | -1.40 | 0.82 | -0.15 | -0.43 | 0.33 | 1.41 |
| Q86XN8 | RNA-binding protein MEX3D | MEX3D | 0.78 | 0.95 | 1.09 | 0.57 | -1.00 | -0.84 | -0.93 | 0.70 | 1.74 | 0.29 | -0.89 | -0.73 |
| Q9UBS8 | E3 ubiquitin-protein ligase RNF14 | RNF14 | 0.78 | 0.85 | 0.24 | -1.50 | -1.63 | 1.03 | 0.83 | -0.09 | 0.32 | -0.87 | 0.96 | 0.69 |

| | | | | | | | | | | | | | | |
|---|---|---|---|---|---|---|---|---|---|---|---|---|---|---|
| Q02153 | Guanylate cyclase soluble subunit beta-1 | GUCY1B1 | 0.78 | 2.03 | 0.44 | -2.24 | -0.26 | 1.00 | -0.92 | 0.35 | -0.33 | 1.08 | 0.62 | 0.27 |
| Q9HB07 | UPF0160 protein MYG1, mitochondrial | C12orf10 | 0.78 | 1.44 | 0.27 | -0.56 | 0.78 | -1.29 | -0.73 | 1.63 | 1.13 | -0.12 | -1.35 | 0.24 |
| P12081 | Histidine--tRNA ligase, cytoplasmic | HARS | 0.77 | 4.13 | -1.54 | -1.23 | 0.82 | -0.02 | -1.00 | 0.45 | 0.23 | 1.16 | -0.19 | 1.34 |
| O00754 | Lysosomal alpha-mannosidase | MAN2B1 | 0.77 | 1.40 | -0.20 | 0.00 | -0.61 | -0.42 | -0.32 | -0.24 | -0.29 | 2.81 | -0.46 | -0.26 |
| Q6DD88 | Atlastin-3 | ATL3 | 0.77 | 0.77 | 0.67 | -0.70 | 0.57 | 0.27 | -1.74 | 1.40 | 0.27 | -1.51 | 0.47 | 0.31 |
| Q8TBC4 | NEDD8-activating enzyme E1 catalytic subunit | UBA3 | 0.77 | 1.70 | 0.25 | -0.71 | -1.12 | -1.29 | 1.17 | 0.12 | 0.25 | 0.15 | -0.71 | 1.88 |
| Q7KZI7 | Serine/threonine-protein kinase MARK2 | MARK2 | 0.77 | 1.34 | 1.05 | 0.07 | -1.17 | -0.48 | -0.91 | 1.42 | 1.17 | 0.61 | -0.62 | -1.14 |
| Q6P9B6 | MTOR-associated protein MEAK7 | MEAK7 | 0.77 | 0.86 | -0.42 | -0.55 | -0.76 | -0.61 | 1.31 | 1.66 | -0.56 | -0.82 | -0.59 | 1.33 |
| O75167 | Phosphatase and actin regulator 2 | PHACTR2 | 0.77 | 0.55 | 0.80 | 0.30 | -0.87 | -0.11 | -0.82 | 1.49 | 1.63 | -0.97 | -0.73 | -0.71 |
| Q9Y6F9 | Protein Wnt-6 | WNT6 | 0.77 | 1.18 | -0.01 | -0.37 | -0.57 | -0.18 | -0.23 | -0.19 | 2.79 | -0.71 | -0.30 | -0.22 |
| O60841 | Eukaryotic translation initiation factor 5B | EIF5B | 0.77 | 3.58 | -0.10 | -1.08 | 0.59 | -0.26 | -1.87 | 0.16 | 0.07 | 1.16 | -0.23 | 1.57 |
| Q9HD89 | Resistin | RETN | 0.76 | 1.05 | -0.68 | -0.69 | -0.88 | -0.68 | 1.75 | -0.69 | 1.47 | 0.45 | -0.70 | 0.65 |
| Q15046 | Lysine--tRNA ligase | KARS | 0.76 | 4.36 | -1.47 | -1.09 | -0.30 | 0.68 | -0.83 | -0.53 | 0.99 | 1.65 | 0.50 | 0.40 |
| P62829 | 60S ribosomal protein L23 | RPL23 | 0.76 | 2.72 | 0.95 | -1.61 | -0.87 | 0.43 | -1.26 | 0.48 | 0.88 | -0.67 | 0.57 | 1.10 |
| Q5EBM0 | UMP-CMP kinase 2, mitochondrial | CMPK2 | 0.76 | 0.83 | -0.55 | 0.46 | -1.71 | 0.12 | 0.68 | -0.31 | 0.70 | 1.39 | -1.46 | 0.67 |
| Q9HB90 | Ras-related GTP-binding protein C | RRAGC | 0.76 | 2.60 | 1.33 | -1.61 | -0.26 | -0.64 | -1.11 | 0.05 | 1.44 | -0.38 | 0.50 | 0.68 |
| Q8WUB8 | PHD finger protein 10 | PHF10 | 0.76 | 1.07 | 0.51 | -0.31 | -1.26 | -1.08 | 0.92 | 0.19 | 1.57 | -1.35 | 0.85 | -0.05 |
| O75396 | Vesicle-trafficking protein SEC22b | SEC22B | 0.76 | 1.40 | -0.16 | 0.03 | -0.61 | 0.08 | -0.87 | 0.63 | -1.29 | 2.30 | -0.54 | 0.44 |
| Q92598 | Heat shock protein 105 kDa | HSPH1 | 0.76 | 3.94 | -0.66 | -0.19 | 0.50 | -0.64 | -1.87 | 0.85 | 1.78 | 0.19 | -0.45 | 0.49 |
| Q9Y262 | Eukaryotic translation initiation factor 3 subunit L | EIF3L | 0.76 | 1.50 | 1.51 | -1.77 | -0.38 | -0.22 | -0.73 | 0.06 | 0.96 | 1.20 | 0.06 | -0.71 |
| Q9Y2S2 | Lambda-crystallin homolog | CRYL1 | 0.75 | 1.66 | -0.34 | -1.11 | -0.18 | 0.32 | -0.41 | 0.64 | -1.70 | 1.91 | 0.56 | 0.31 |
| P41229 | Lysine-specific demethylase 5C | KDM5C | 0.75 | 2.70 | -0.51 | -0.90 | 0.51 | 0.14 | -1.55 | 2.08 | -0.52 | -0.28 | 0.59 | 0.44 |
| Q8N8N7 | Prostaglandin reductase 2 | PTGR2 | 0.75 | 3.24 | 0.01 | -0.19 | -0.56 | -0.35 | -1.55 | 2.18 | -0.57 | -0.08 | 1.00 | 0.09 |
| P02511 | Alpha-crystallin B chain | CRYAB | 0.75 | 2.18 | 0.19 | -0.98 | -0.19 | 0.53 | -1.57 | 1.82 | -0.74 | -0.53 | 0.72 | 0.75 |
| Q96EQ0 | Small glutamine-rich tetratricopeptide repeat-containing protein b | SGTB | 0.75 | 0.54 | 1.19 | 0.47 | -1.11 | -0.16 | -1.08 | 1.06 | 1.33 | 0.28 | -1.03 | -0.94 |
| P04066 | Tissue alpha-L-fucosidase | FUCA1 | 0.75 | 1.62 | -0.04 | -0.33 | -0.72 | -0.33 | -0.32 | -0.40 | -0.20 | 2.80 | -0.29 | -0.18 |
| O15061 | Synemin | SYNM | 0.75 | 0.56 | 1.31 | 0.06 | -1.38 | 0.16 | -0.87 | 1.61 | -0.75 | -0.92 | 0.01 | 0.76 |
| Q9UJC5 | SH3 domain-binding glutamic acid-rich-like protein 2 | SH3BGRL2 | 0.75 | 2.43 | -0.27 | -1.01 | -0.16 | 0.09 | -0.90 | -0.80 | -0.10 | 0.06 | 0.64 | 2.45 |
| P32929 | Cystathionine gamma-lyase | CTH | 0.75 | 1.44 | -0.09 | -0.43 | -0.51 | -0.24 | -0.31 | -0.28 | -0.45 | 2.82 | -0.29 | -0.21 |
| P68104 | Elongation factor 1-alpha 1 | EEF1A1 | 0.75 | 2.73 | -1.02 | -0.17 | -1.67 | 0.46 | 0.08 | 1.30 | -0.47 | -0.58 | 0.53 | 1.55 |
| Q99733 | Nucleosome assembly protein 1-like 4 | NAP1L4 | 0.75 | 4.03 | -0.85 | 0.48 | -0.62 | -0.33 | -1.59 | 0.60 | 1.60 | -0.66 | 0.08 | 1.28 |
| P11766 | Alcohol dehydrogenase class-3 | ADH5 | 0.75 | 3.17 | -1.05 | -1.39 | 0.74 | 0.22 | -1.05 | 0.14 | -0.59 | 1.78 | 0.54 | 0.68 |
| P45984 | Mitogen-activated protein kinase 9 | MAPK9 | 0.75 | 1.55 | 1.34 | 0.14 | -0.60 | -0.27 | -2.26 | 1.11 | 0.62 | -0.18 | 0.02 | 0.08 |
| Q7Z3J2 | VPS35 endosomal protein sorting factor-like | VPS35L | 0.74 | 0.88 | 0.71 | -0.11 | -1.57 | 0.24 | -0.31 | 1.07 | 0.79 | -1.79 | 0.02 | 0.95 |
| O00214 | Galectin-8 | LGALS8 | 0.74 | 3.33 | -0.87 | -0.66 | -0.73 | 0.24 | -0.72 | -0.78 | -0.12 | 2.24 | 0.88 | 0.53 |
| Q15102 | Platelet-activating factor acetylhydrolase IB subunit gamma | PAFAH1B3 | 0.74 | 1.95 | -1.74 | 0.77 | -0.39 | 0.21 | -0.72 | 0.99 | 0.59 | -1.13 | -0.01 | 1.43 |
| O94856 | Neurofascin | NFASC | 0.74 | 1.50 | -0.39 | -1.76 | -0.37 | 0.02 | 0.95 | 0.89 | -0.16 | 1.61 | 0.31 | -1.09 |
| Q9BTZ2 | Dehydrogenase/reductase SDR family member 4 | DHRS4 | 0.74 | 0.91 | 0.66 | 0.17 | -1.33 | 0.67 | -1.23 | 1.16 | 0.57 | -1.63 | 0.40 | 0.56 |
| P25398 | 40S ribosomal protein S12 | RPS12 | 0.74 | 4.30 | -0.45 | -0.89 | -0.78 | -1.16 | 0.19 | 1.54 | 1.57 | 0.84 | -0.63 | -0.22 |
| P25311 | Zinc-alpha-2-glycoprotein | AZGP1 | 0.74 | 1.38 | 0.00 | -0.26 | -0.46 | -0.35 | -0.47 | -0.34 | -0.27 | 2.82 | -0.40 | -0.27 |
| P49758 | Regulator of G-protein signaling 6 | RGS6 | 0.74 | 2.05 | -0.43 | 0.40 | -0.11 | -0.73 | -1.12 | 1.37 | 1.17 | -1.45 | -0.28 | 1.18 |
| P52888 | Thimet oligopeptidase | THOP1 | 0.74 | 2.49 | 0.31 | -0.99 | 0.45 | -0.08 | -1.89 | 0.38 | 0.90 | 1.68 | -0.16 | -0.59 |
| Q9NUP9 | Protein lin-7 homolog C | LIN7C | 0.74 | 0.76 | -1.17 | 0.22 | 0.61 | 0.83 | -1.41 | 0.81 | 0.54 | -1.68 | 0.65 | 0.60 |
| P23142 | Fibulin-1 | FBLN1 | 0.74 | 0.81 | -0.04 | 0.18 | -0.65 | -0.50 | 0.00 | 0.56 | -2.22 | 1.47 | 0.29 | 0.91 |
| Q15032 | R3H domain-containing protein 1 | R3HDM1 | 0.74 | 2.12 | 0.03 | -0.52 | -0.54 | -0.49 | -0.58 | 2.07 | -0.41 | -0.85 | -0.32 | 1.61 |
| P21964 | Catechol O-methyltransferase | COMT | 0.74 | 1.57 | -1.55 | 0.24 | -1.00 | 1.04 | -0.33 | 0.98 | 0.24 | -1.35 | 0.95 | 0.78 |
| O92997 | Segment polarity protein dishevelled homolog DVL-3 | DVL3 | 0.73 | 1.87 | 0.01 | -0.79 | -0.45 | -0.60 | -0.08 | 0.18 | -0.06 | 2.69 | -0.72 | -0.17 |
| P62736 | Actin, aortic smooth muscle | ACTA2 | 0.73 | 0.79 | -0.55 | 1.08 | 0.29 | 0.34 | -2.12 | -0.75 | -0.41 | 0.99 | 0.08 | 1.05 |
| Q9Y6R7 | IgGFc-binding protein | FCGBP | 0.73 | 2.01 | 0.93 | 0.32 | -1.44 | -0.24 | -1.48 | 0.80 | 0.66 | -0.93 | 0.06 | 1.33 |
| Q03519 | Antigen peptide transporter 2 | TAP2 | 0.73 | 1.13 | -0.32 | -0.44 | -0.69 | 0.83 | -0.67 | 1.04 | -0.54 | -0.92 | -0.45 | 2.16 |
| Q9BZG1 | Ras-related protein Rab-34 | RAB34 | 0.73 | 1.85 | -0.86 | -0.35 | -0.54 | -0.02 | -0.13 | 0.87 | -1.15 | 0.04 | -0.24 | 2.38 |
| Q8NFH3 | Nucleoporin Nup43 | NUP43 | 0.73 | 1.33 | -0.14 | -0.69 | -1.10 | 0.72 | -0.24 | 1.04 | -0.76 | -1.35 | 1.08 | 1.44 |
| Q13480 | GRB2-associated-binding protein 1 | GAB1 | 0.73 | 1.46 | -0.02 | -1.31 | -0.01 | 0.37 | -0.59 | 0.97 | -1.15 | -0.94 | 1.54 | 1.14 |
| O14562 | Ubiquitin domain-containing protein UBFD1 | UBFD1 | 0.73 | 1.91 | -1.46 | -1.02 | 0.02 | 0.03 | 0.57 | -0.77 | 1.64 | 1.35 | 0.24 | -0.59 |
| P13639 | Elongation factor 2 | EEF2 | 0.73 | 2.50 | 0.31 | -0.46 | -0.34 | 0.29 | -2.01 | 1.57 | 1.13 | -0.79 | -0.01 | 0.30 |
| Q6ZMI3 | Gliomedin | GLDN | 0.72 | 1.13 | -0.66 | -0.50 | -0.21 | 0.32 | -0.27 | -2.16 | 1.07 | 0.85 | 0.37 | 1.20 |
| P13591 | Neural cell adhesion molecule 1 | NCAM1 | 0.72 | 2.73 | -0.48 | 0.20 | -0.47 | 0.23 | -1.81 | 1.36 | 0.24 | 1.31 | 0.54 | -1.13 |
| P30046 | D-dopachrome decarboxylase | DDT | 0.72 | 1.01 | 0.08 | -0.75 | 0.28 | -0.38 | -0.42 | -0.19 | -1.61 | 2.25 | 0.20 | 0.54 |
| Q658Y4 | Protein FAM91A1 | FAM91A1 | 0.72 | 0.91 | -1.36 | 0.48 | 0.87 | -1.42 | 0.37 | 0.74 | -1.51 | 0.79 | 0.57 | 0.48 |
| P30520 | Adenylosuccinate synthetase isozyme 2 | ADSS | 0.72 | 0.87 | 1.29 | -0.17 | -1.13 | -0.52 | -0.51 | 1.37 | 1.28 | 0.31 | -0.80 | -1.12 |
| O75832 | 26S proteasome non-ATPase regulatory subunit 10 | PSMD10 | 0.72 | 1.43 | 0.33 | -0.23 | -0.27 | 0.48 | -1.82 | 1.64 | -0.80 | -0.78 | 0.40 | 1.06 |
| Q9UN36 | Protein NDRG2 | NDRG2 | 0.72 | 1.20 | -1.12 | -0.68 | -0.05 | 0.31 | 0.19 | 0.22 | -1.01 | 2.42 | 0.16 | -0.43 |
| Q07812 | Apoptosis regulator BAX | BAX | 0.72 | 0.84 | 1.13 | 0.11 | -1.03 | -0.27 | -0.95 | 1.37 | 1.51 | -1.00 | -0.67 | -0.22 |
| Q9Y4B5 | Microtubule cross-linking factor 1 | MTCL1 | 0.72 | 0.74 | 1.64 | -0.90 | -1.02 | -0.21 | -0.41 | 1.12 | 1.27 | -0.27 | -0.09 | -1.14 |
| Q16401 | 26S proteasome non-ATPase regulatory subunit 5 | PSMD5 | 0.72 | 0.78 | 0.57 | 0.31 | -1.81 | 0.70 | -0.71 | 1.23 | -1.07 | -0.72 | 0.66 | 0.84 |
| Q01826 | DNA-binding protein SATB1 | SATB1 | 0.72 | 0.87 | 0.14 | -0.64 | -0.86 | 0.97 | -0.65 | -0.65 | 0.77 | -0.66 | -0.58 | 2.16 |
| O75815 | Breast cancer anti-estrogen resistance protein 3 | BCAR3 | 0.72 | 0.83 | 0.65 | 0.62 | -1.28 | 0.25 | -1.23 | 0.99 | 1.27 | -1.53 | 0.20 | 0.07 |
| P01009 | Alpha-1-antitrypsin | SERPINA1 | 0.72 | 1.24 | -0.49 | -0.49 | 0.77 | -1.23 | 0.07 | -0.27 | -0.07 | 2.23 | -1.04 | 0.52 |
| Q9Y5X1 | Sorting nexin-9 | SNX9 | 0.72 | 0.53 | 1.06 | 0.45 | -1.54 | -0.35 | -0.31 | 1.30 | 0.81 | -1.63 | -0.10 | 0.31 |
| Q13576 | Ras GTPase-activating-like protein IQGAP2 | IQGAP2 | 0.72 | 0.67 | 1.14 | -0.98 | 1.01 | -0.97 | -1.04 | 1.39 | 0.33 | -1.21 | -0.12 | 0.45 |
| Q13129 | Zinc finger protein Rlf | RLF | 0.72 | 1.45 | -0.17 | -0.29 | -0.49 | -0.24 | -0.43 | -0.36 | -0.28 | 2.83 | -0.28 | -0.31 |
| O00622 | CCN family member 1 | CCN1 | 0.71 | 1.48 | -0.05 | -0.36 | -0.63 | -0.29 | -0.30 | -0.21 | -0.35 | 2.82 | -0.25 | -0.38 |
| Q9UM19 | Hippocalcin-like protein 4 | HPCAL4 | 0.71 | 0.61 | 1.43 | -0.87 | 0.39 | -0.80 | -0.91 | 1.27 | 0.94 | -1.02 | -0.89 | 0.49 |
| Q9HCD6 | Protein TANC2 | TANC2 | 0.71 | 1.62 | -0.86 | -0.01 | -0.67 | 0.74 | -0.88 | -0.32 | -1.39 | 1.79 | 0.71 | 0.88 |
| Q8IY95 | Transmembrane protein 192 | TMEM192 | 0.71 | 0.80 | -0.72 | 1.15 | -1.01 | 0.45 | -0.84 | 1.08 | 1.35 | -1.17 | 0.50 | -0.80 |
| O43143 | Pre-mRNA-splicing factor ATP-dependent RNA helicase DHX15 | DHX15 | 0.71 | 3.54 | 0.02 | -2.35 | -0.70 | -0.16 | 0.41 | 0.12 | -0.08 | 0.64 | 0.79 | 1.31 |
| P16070 | CD44 antigen | CD44 | 0.71 | 0.70 | -1.34 | -1.41 | 1.13 | 0.25 | 0.51 | -0.12 | -0.69 | 1.76 | 0.06 | -0.14 |
| Q9BRS8 | La-related protein 6 | LARP6 | 0.71 | 3.42 | 0.69 | -0.94 | -0.68 | 0.33 | -2.23 | 0.52 | 0.38 | 1.20 | 0.40 | 0.32 |
| Q9NXR8 | Inhibitor of growth protein 3 | ING3 | 0.71 | 0.81 | 0.65 | -0.35 | -0.56 | -0.39 | -0.35 | 2.66 | -0.36 | -0.72 | -0.31 | -0.27 |
| O15013 | Rho guanine nucleotide exchange factor 10 | ARHGEF10 | 0.71 | 0.83 | 0.84 | -1.16 | -0.73 | -0.75 | 0.80 | 1.03 | 1.12 | -1.60 | -0.13 | 0.56 |
| A6NGB9 | WAS/WASL-interacting protein family member 3 | WIPF3 | 0.71 | 0.89 | 0.81 | 0.80 | -2.20 | 0.33 | -0.80 | -0.71 | 1.03 | -0.18 | 0.23 | 0.68 |
| Q8NBS9 | Thioredoxin domain-containing protein 5 | TXNDC5 | 0.71 | 0.81 | -0.79 | -0.97 | 0.35 | 1.35 | -0.92 | 1.23 | -0.98 | 0.40 | 1.15 | -0.83 |
| Q38SD2 | Leucine-rich repeat serine/threonine-protein kinase 1 | LRRK1 | 0.71 | 0.77 | 0.96 | -0.59 | -0.84 | 0.13 | -0.60 | 2.28 | -0.66 | 0.52 | -0.59 | -0.61 |

| | | | | | | | | | | | | | | |
|---|---|---|---|---|---|---|---|---|---|---|---|---|---|---|
| P98095 | Fibulin-2 | FBLN2 | 0.71 | 1.20 | -0.16 | -0.35 | -0.39 | -0.17 | -0.31 | -0.14 | -0.29 | -0.64 | -0.36 | 2.82 |
| P08123 | Collagen alpha-2(I) chain | COL1A2 | 0.70 | 0.80 | 0.13 | 0.10 | 1.18 | -0.12 | -2.26 | 0.20 | 0.82 | -0.85 | -0.22 | 1.01 |
| P14868 | Aspartate--tRNA ligase, cytoplasmic | DARS | 0.70 | 2.41 | -0.04 | -1.61 | -0.22 | -0.15 | -0.18 | 2.19 | 0.12 | -0.83 | -0.17 | 0.88 |
| P00352 | Retinal dehydrogenase 1 | ALDH1A1 | 0.70 | 1.05 | 0.07 | -2.67 | 0.15 | 0.00 | 1.21 | 0.29 | -0.01 | 0.30 | 0.22 | 0.43 |
| P61970 | Nuclear transport factor 2 | NUTF2 | 0.70 | 1.40 | -1.18 | -0.17 | -0.12 | 0.47 | -0.52 | -0.94 | -0.71 | 2.24 | 0.81 | 0.12 |
| Q3LXA3 | Triokinase/FMN cyclase | TKFC | 0.70 | 2.38 | -0.07 | -1.82 | -0.06 | -0.01 | -0.18 | 1.83 | -1.07 | 0.94 | 0.49 | -0.04 |
| Q86VQ1 | Glucocorticoid-induced transcript 1 protein | GLCCI1 | 0.70 | 0.61 | -0.33 | 0.90 | -0.50 | -0.45 | -0.40 | -0.48 | -0.46 | 2.58 | -0.47 | -0.39 |
| P78563 | Double-stranded RNA-specific editase 1 | ADARB1 | 0.70 | 2.18 | 0.60 | -1.50 | 0.93 | -0.88 | -1.18 | 0.26 | 0.85 | 1.12 | -0.90 | 0.70 |
| Q14644 | Ras GTPase-activating protein 3 | RASA3 | 0.70 | 0.89 | 0.30 | 0.22 | -1.45 | 0.17 | -0.31 | 0.74 | 0.10 | -1.94 | 1.08 | 1.08 |
| Q9Y4X5 | E3 ubiquitin-protein ligase ARIH1 | ARIH1 | 0.70 | 1.86 | -0.26 | -1.56 | -1.38 | 0.09 | 1.28 | 0.14 | -0.21 | 1.68 | 0.25 | -0.02 |
| O75643 | U5 small nuclear ribonucleoprotein 200 kDa helicase | SNRNP200 | 0.70 | 2.65 | -1.14 | -0.55 | 0.87 | 0.13 | -1.59 | -0.05 | -0.65 | 0.72 | 0.61 | 1.66 |
| Q13404 | Ubiquitin-conjugating enzyme E2 variant 1 | UBE2V1 | 0.70 | 2.91 | 0.78 | -0.33 | 0.01 | -0.58 | -2.42 | 0.59 | 0.34 | 1.19 | -0.07 | 0.48 |
| Q9BQG0 | Myb-binding protein 1A | MYBBP1A | 0.70 | 0.88 | 1.35 | -0.69 | 0.74 | -2.14 | -0.33 | 0.41 | 0.51 | 0.43 | -0.78 | 0.48 |
| P35268 | 60S ribosomal protein L22 | RPL22 | 0.70 | 6.09 | -1.39 | -0.50 | -1.18 | 0.32 | -0.76 | -0.40 | 0.75 | 0.47 | 1.03 | 1.66 |
| Q6ZRS2 | Helicase SRCAP | SRCAP | 0.69 | 0.78 | 1.43 | -0.52 | -0.71 | -0.59 | -0.54 | 1.10 | 1.75 | -0.82 | -0.50 | -0.58 |
| P62906 | 60S ribosomal protein L10a | RPL10A | 0.69 | 2.18 | -0.93 | -1.50 | -0.80 | 0.50 | 0.71 | -0.49 | 1.37 | 1.43 | 0.24 | -0.53 |
| P98170 | E3 ubiquitin-protein ligase XIAP | XIAP | 0.69 | 0.72 | 1.07 | 0.27 | -1.44 | -0.48 | -0.32 | 0.95 | -0.28 | -1.44 | 0.14 | 1.51 |
| Q9NTZ6 | RNA-binding protein 12 | RBM12 | 0.69 | 0.94 | 1.65 | 0.40 | -1.83 | -0.53 | -0.81 | 0.89 | 0.76 | 0.29 | -0.36 | -0.48 |
| P16435 | NADPH--cytochrome P450 reductase | POR | 0.69 | 1.70 | -0.35 | 0.62 | -0.56 | -0.18 | -1.28 | 1.93 | 0.36 | -0.87 | -0.81 | 1.12 |
| P30101 | Protein disulfide-isomerase A3 | PDIA3 | 0.69 | 1.81 | -1.24 | 0.44 | 0.40 | -0.26 | -1.13 | 1.33 | 0.64 | -1.50 | 0.18 | 1.15 |
| P12814 | Alpha-actinin-1 | ACTN1 | 0.69 | 0.61 | -0.13 | 0.92 | -2.03 | 0.04 | 0.44 | -0.90 | 1.34 | 0.96 | -0.15 | -0.49 |
| P08246 | Neutrophil elastase | ELANE | 0.69 | 0.82 | 0.35 | -1.18 | 0.39 | -1.04 | 0.49 | 0.91 | -1.07 | 1.32 | -1.15 | 0.98 |
| P02768 | Serum albumin | ALB | 0.69 | 0.92 | -0.20 | -0.12 | 0.19 | -0.84 | -0.14 | 0.16 | -1.17 | 2.53 | -0.58 | 0.17 |
| P07711 | Cathepsin L1 | CTSL | 0.69 | 1.02 | -0.47 | -0.55 | -0.76 | -0.62 | 1.22 | -0.59 | -0.63 | 1.86 | -0.65 | 1.18 |
| Q53GT1 | Kelch-like protein 22 | KLHL22 | 0.69 | 0.78 | 0.94 | -0.13 | -2.57 | 0.60 | 0.19 | -0.01 | -0.32 | 0.86 | 0.48 | -0.04 |
| Q9Y4C2 | TRPM8 channel-associated factor 1 | TCAF1 | 0.69 | 0.99 | 0.65 | 0.73 | -1.78 | -0.54 | -0.20 | -0.17 | 1.89 | 0.54 | -0.62 | -0.50 |
| Q9H0U4 | Ras-related protein Rab-1B | RAB1B | 0.69 | 4.75 | -1.00 | -0.31 | -1.33 | -0.62 | 0.04 | 1.13 | 1.36 | 0.55 | -0.97 | 1.17 |
| O95163 | Elongator complex protein 1 | ELP1 | 0.69 | 0.86 | -0.40 | -0.08 | -0.96 | 1.27 | -0.86 | -0.93 | -0.84 | -0.04 | 1.37 | 1.46 |
| P62280 | 40S ribosomal protein S11 | RPS11 | 0.68 | 3.50 | -1.04 | -1.85 | -0.88 | 1.22 | -0.31 | 0.56 | 0.19 | 0.46 | 1.13 | 0.53 |
| P15559 | NAD(P)H dehydrogenase [quinone] 1 | NQO1 | 0.68 | 5.39 | -1.99 | -0.99 | 0.21 | -0.20 | -0.39 | 0.52 | 0.34 | 1.74 | 0.16 | 0.61 |
| P02649 | Apolipoprotein E | APOE | 0.68 | 1.08 | 0.03 | 0.30 | -2.16 | -0.47 | 1.07 | -0.09 | -0.30 | 1.17 | -0.58 | 1.03 |
| P07947 | Tyrosine-protein kinase Yes | YES1 | 0.68 | 0.80 | -0.99 | -0.57 | 0.31 | 0.68 | -0.40 | 1.08 | -2.11 | 0.66 | 0.75 | 0.59 |
| Q53H82 | Endoribonuclease LACTB2 | LACTB2 | 0.68 | 1.40 | 1.15 | -0.57 | -0.91 | -0.63 | -0.53 | 1.54 | -0.49 | 1.59 | -0.70 | -0.45 |
| Q8IVF2 | Protein AHNAK2 | AHNAK2 | 0.68 | 0.32 | 0.73 | -1.43 | 0.67 | 0.76 | -1.17 | 0.61 | -0.41 | 0.79 | 0.89 | -1.44 |
| Q14160 | Protein scribble homolog | SCRIB | 0.68 | 0.79 | 1.52 | -0.18 | -0.24 | -0.12 | -1.93 | 1.08 | 0.86 | -0.97 | -0.16 | 0.15 |
| Q6VEQ5 | WAS protein family homolog 2 | WASH2P | 0.68 | 1.91 | 0.61 | 0.79 | -1.26 | -0.93 | -1.05 | 0.81 | 0.08 | 0.70 | -1.15 | 1.41 |
| Q13137 | Calcium-binding and coiled-coil domain-containing protein 2 | CALCOCO2 | 0.68 | 0.96 | 0.58 | -0.45 | -1.05 | -0.81 | 0.60 | 1.37 | 0.56 | -1.23 | -0.92 | 1.34 |
| Q86VB7 | Scavenger receptor cysteine-rich type 1 protein M130 | CD163 | 0.68 | 0.91 | -0.46 | 1.57 | -0.81 | -0.67 | -0.70 | -0.12 | -0.73 | 1.52 | -0.75 | 1.14 |
| Q9Y316 | Protein MEMO1 | MEMO1 | 0.68 | 1.32 | -0.15 | -0.31 | -0.60 | -0.21 | -0.21 | -0.21 | -0.32 | -0.62 | -0.17 | 2.81 |
| Q9Y315 | Deoxyribose-phosphate aldolase | DERA | 0.68 | 1.09 | 2.06 | -1.02 | -0.44 | -1.28 | -0.57 | -0.18 | 0.60 | 0.80 | -0.50 | 0.53 |
| Q9UMX0 | Ubiquilin-1 | UBQLN1 | 0.68 | 0.94 | 0.00 | 0.62 | -1.82 | -0.01 | 0.12 | -1.72 | 1.12 | 0.30 | 0.63 | 0.77 |
| Q96AC6 | Kinesin-like protein KIFC2 | KIFC2 | 0.68 | 1.07 | 0.80 | -1.79 | -0.66 | 0.36 | 0.08 | -0.79 | 1.19 | 1.41 | 0.14 | -0.73 |
| Q12929 | Epidermal growth factor receptor kinase substrate 8 | EPS8 | 0.68 | 0.92 | -0.31 | 0.09 | 0.72 | -0.12 | -1.46 | 1.51 | 0.54 | -1.74 | -0.03 | 0.80 |
| Q9HCU5 | Prolactin regulatory element-binding protein | PREB | 0.68 | 1.01 | 0.06 | 0.19 | 0.38 | 0.25 | -2.04 | 1.70 | 0.15 | -1.22 | 0.41 | 0.12 |
| Q9UHQ9 | NADH-cytochrome b5 reductase 1 | CYB5R1 | 0.68 | 1.57 | -1.97 | 0.52 | 0.69 | 0.46 | -1.34 | -0.56 | -0.17 | 0.50 | 1.04 | 0.83 |
| Q9BSA4 | Protein tweety homolog 2 | TTYH2 | 0.67 | 0.53 | -0.31 | -1.18 | -0.50 | 0.64 | 0.67 | -1.23 | 1.24 | 1.00 | 0.91 | -1.23 |
| O00231 | 26S proteasome non-ATPase regulatory subunit 11 | PSMD11 | 0.67 | 2.06 | 0.47 | 0.68 | -1.31 | -1.46 | -0.34 | 1.60 | 0.46 | -1.02 | 0.16 | 0.74 |
| Q15262 | Receptor-type tyrosine-protein phosphatase kappa | PTPRK | 0.67 | 0.77 | -1.92 | 0.27 | 0.59 | 0.32 | -0.19 | -0.68 | -1.06 | 1.63 | 0.52 | 0.53 |
| Q15386 | Ubiquitin-protein ligase E3C | UBE3C | 0.67 | 1.56 | -1.05 | 0.17 | -0.88 | 0.24 | -0.12 | -1.41 | 0.84 | 2.07 | 0.13 | 0.01 |
| O95147 | Dual specificity protein phosphatase 14 | DUSP14 | 0.67 | 1.26 | -0.02 | -0.35 | -0.61 | -0.27 | -0.19 | -0.26 | -0.11 | -0.81 | -0.15 | 2.76 |
| Q9UBQ7 | Glyoxylate reductase/hydroxypyruvate reductase | GRHPR | 0.67 | 2.65 | -1.34 | -1.31 | 0.22 | 0.20 | -0.06 | -0.13 | 0.00 | 2.24 | 0.52 | -0.34 |
| Q06323 | Proteasome activator complex subunit 1 | PSME1 | 0.67 | 0.74 | 0.82 | -0.65 | -1.17 | -0.41 | 0.49 | 0.68 | -1.38 | 1.43 | 1.01 | -0.83 |
| Q709C8 | Vacuolar protein sorting-associated protein 13C | VPS13C | 0.67 | 1.00 | -0.58 | -0.29 | 0.01 | 0.00 | -0.33 | -1.11 | -0.81 | 2.52 | 0.14 | 0.44 |
| Q9P1Z0 | Zinc finger and BTB domain-containing protein 4 | ZBTB4 | 0.67 | 0.88 | 1.31 | -0.61 | -0.88 | -0.70 | -0.16 | -0.56 | -0.69 | 1.43 | 1.52 | -0.67 |
| O75521 | Enoyl-CoA delta isomerase 2, mitochondrial | ECI2 | 0.67 | 1.04 | -0.81 | -0.27 | 1.23 | -0.95 | -0.39 | -1.01 | -0.89 | 1.34 | 0.33 | 1.42 |
| Q53SF7 | Cordon-bleu protein-like 1 | COBLL1 | 0.67 | 0.96 | -0.64 | -0.54 | -0.85 | 0.68 | 0.22 | -1.04 | -1.34 | 1.38 | 0.94 | 1.18 |
| Q9NRV9 | Heme-binding protein 1 | HEBP1 | 0.67 | 5.15 | -0.78 | -1.80 | -0.27 | -0.58 | 0.17 | 0.00 | 1.45 | 1.59 | -0.02 | 0.24 |
| Q9UM54 | Unconventional myosin-VI | MYO6 | 0.67 | 0.92 | -0.30 | -0.31 | -0.08 | -0.07 | -0.36 | 2.72 | -0.37 | -1.07 | -0.09 | -0.06 |
| Q9UHY7 | Enolase-phosphatase E1 | ENOPH1 | 0.66 | 1.00 | -2.26 | 0.20 | 0.08 | 0.67 | 0.16 | -0.26 | -1.08 | 0.65 | 0.84 | 1.01 |
| P06454 | Prothymosin alpha | PTMA | 0.66 | 0.86 | -0.17 | 0.26 | -0.56 | -0.17 | -0.42 | -0.33 | -0.33 | 2.76 | -0.61 | -0.44 |
| Q9Y6P5 | Sestrin-1 | SESN1 | 0.66 | 0.80 | 1.19 | -0.84 | -1.31 | -0.15 | 0.16 | 1.61 | 0.48 | -1.39 | -0.27 | 0.53 |
| P18077 | 60S ribosomal protein L35a | RPL35A | 0.66 | 2.08 | 0.09 | -1.99 | -0.06 | 0.45 | -0.45 | 0.71 | 0.90 | -1.24 | 0.30 | 1.30 |
| Q9NRW7 | Vacuolar protein sorting-associated protein 45 | VPS45 | 0.66 | 1.06 | 1.33 | -0.49 | -1.45 | 0.07 | -0.66 | 1.69 | -1.11 | -0.10 | 0.16 | 0.57 |
| Q9Y547 | Intraflagellar transport protein 25 homolog | HSPB11 | 0.66 | 1.16 | 0.02 | -0.15 | -0.57 | -0.30 | -0.35 | -0.30 | -0.28 | -0.60 | -0.28 | 2.80 |
| Q16720 | Plasma membrane calcium-transporting ATPase 3 | ATP2B3 | 0.66 | 1.52 | -1.71 | 0.95 | 0.31 | -0.49 | -0.65 | 0.26 | 0.83 | -0.83 | -0.38 | 1.69 |
| P46734 | Dual specificity mitogen-activated protein kinase kinase 3 | MAP2K3 | 0.66 | 0.51 | 1.50 | -0.20 | -1.18 | 0.31 | -1.09 | 1.45 | -0.28 | -1.25 | 0.35 | 0.40 |
| Q8WUY8 | N-acetyltransferase 14 | NAT14 | 0.66 | 2.16 | -0.23 | 0.33 | -2.10 | 0.57 | -0.58 | 1.33 | -0.37 | -0.56 | 0.44 | 1.16 |
| Q13114 | TNF receptor-associated factor 3 | TRAF3 | 0.66 | 0.52 | 1.53 | -0.16 | -1.13 | -0.22 | -0.69 | 1.38 | 1.22 | -0.60 | -0.37 | -0.96 |
| P17858 | ATP-dependent 6-phosphofructokinase, liver type | PFKL | 0.66 | 4.04 | -1.31 | -1.14 | -0.05 | 0.86 | -1.30 | 0.43 | 1.57 | 0.28 | 0.88 | -0.23 |
| Q8IUZ5 | 5-phosphohydroxy-L-lysine phospho-lyase | PHYKPL | 0.66 | 0.74 | -0.80 | -0.86 | -0.97 | 0.61 | 1.12 | 0.44 | 0.26 | 1.85 | -0.86 | -0.79 |
| Q6ICL3 | Transport and Golgi organization protein 2 homolog | TANGO2 | 0.66 | 1.33 | 0.73 | -0.68 | -0.81 | 0.02 | -0.71 | -0.65 | -0.72 | 2.37 | -0.10 | 0.55 |
| P34932 | Heat shock 70 kDa protein 4 | HSPA4 | 0.66 | 3.93 | -0.85 | 0.26 | -0.32 | -0.45 | -1.53 | -0.10 | 0.43 | 2.27 | 0.46 | -0.16 |
| Q9NZL9 | Methionine adenosyltransferase 2 subunit beta | MAT2B | 0.65 | 1.22 | 0.06 | -0.57 | -0.07 | 0.08 | -0.89 | 0.12 | -1.15 | 2.55 | -0.16 | 0.03 |
| P05091 | Aldehyde dehydrogenase, mitochondrial | ALDH2 | 0.65 | 0.88 | -1.60 | -0.21 | 1.34 | -0.19 | -0.37 | 0.74 | -1.51 | 0.95 | 0.00 | 0.84 |
| O00743 | Serine/threonine-protein phosphatase 6 catalytic subunit | PPP6C | 0.65 | 1.05 | 0.38 | -1.06 | -0.48 | 0.36 | -0.41 | 1.61 | 0.72 | -1.94 | 0.33 | 0.49 |
| O75936 | Gamma-butyrobetaine dioxygenase | BBOX1 | 0.65 | 1.59 | -2.26 | -0.08 | 1.12 | 0.23 | -0.66 | 0.97 | -0.15 | -0.45 | 0.37 | 0.91 |
| Q16891 | MICOS complex subunit MIC60 | IMMT | 0.65 | 1.14 | 1.97 | -0.87 | -0.88 | -0.72 | -0.79 | 0.28 | -0.83 | 1.20 | 0.17 | 0.47 |
| Q9Y2T2 | AP-3 complex subunit mu-1 | AP3M1 | 0.65 | 1.21 | -0.36 | 0.36 | -2.38 | 0.83 | 0.20 | 0.59 | -0.08 | -0.79 | 0.61 | 1.01 |
| Q9UBX1 | Cathepsin F | CTSF | 0.65 | 0.50 | -0.33 | -0.39 | -0.52 | -0.42 | 1.00 | -0.42 | 2.53 | -0.65 | -0.42 | -0.39 |
| Q8WUY3 | Protein prune homolog 2 | PRUNE2 | 0.65 | 0.78 | 0.15 | 0.90 | -2.60 | 0.72 | -0.13 | 0.00 | -0.13 | -0.14 | 0.46 | 0.79 |
| O60262 | Guanine nucleotide-binding protein G(I)/G(S)/G(O) subunit gamma | GNG7 | 0.65 | 1.18 | -0.30 | -0.39 | -0.84 | -0.56 | 0.77 | 2.23 | -0.39 | -1.01 | -0.40 | 0.90 |
| Q6XQN6 | Nicotinate phosphoribosyltransferase | NAPRT | 0.65 | 2.42 | -0.17 | -0.88 | -0.36 | 0.59 | -1.36 | 1.41 | 0.55 | 0.98 | -1.46 | 0.70 |
| Q13627 | Dual specificity tyrosine-phosphorylation-regulated kinase 1A | DYRK1A | 0.65 | 0.77 | 1.43 | -1.18 | -1.85 | -0.12 | 0.77 | 0.89 | -0.26 | -0.53 | 0.26 | 0.59 |

| | | | | | | | | | | | | | | |
|---|---|---|---|---|---|---|---|---|---|---|---|---|---|---|
| Q8N5X7 | Eukaryotic translation initiation factor 4E type 3 | EIF4E3 | 0.65 | 1.00 | -0.06 | -0.14 | -0.47 | -0.24 | -0.28 | 2.80 | -0.29 | -0.74 | -0.28 | -0.29 |
| Q9BYM8 | RanBP-type and C3HC4-type zinc finger-containing protein 1 | RBCK1 | 0.65 | 0.80 | 0.59 | 0.57 | -1.22 | 0.17 | -1.08 | 1.44 | -0.61 | -1.39 | 0.96 | 0.58 |
| P11216 | Glycogen phosphorylase, brain form | PYGB | 0.65 | 3.96 | -0.87 | -0.31 | 0.03 | -0.27 | -1.51 | 1.15 | -0.44 | 2.07 | -0.02 | 0.19 |
| Q92784 | Zinc finger protein DPF3 | DPF3 | 0.64 | 1.18 | -0.12 | -0.26 | -0.40 | -0.19 | -0.40 | -0.25 | 2.80 | -0.73 | -0.36 | -0.08 |
| P52895 | Aldo-keto reductase family 1 member C2 | AKR1C2 | 0.64 | 0.87 | 0.69 | 0.62 | -0.01 | 0.32 | -2.68 | 0.46 | -0.21 | 0.15 | -0.10 | 0.76 |
| P32969 | 60S ribosomal protein L9 | RPL9 | 0.64 | 5.35 | -0.43 | -1.40 | 0.18 | 0.06 | -1.78 | -0.13 | 1.21 | 0.29 | 0.79 | 1.21 |
| Q9UI15 | Transgelin-3 | TAGLN3 | 0.64 | 1.09 | -0.76 | 0.23 | -0.86 | -0.46 | 0.61 | -1.67 | 1.57 | 1.19 | -0.32 | 0.47 |
| O14818 | Proteasome subunit alpha type-7 | PSMA7 | 0.64 | 1.69 | -0.86 | -1.69 | -0.56 | 0.12 | 1.29 | -0.13 | -0.55 | 1.69 | 0.40 | 0.30 |
| Q6UN15 | Pre-mRNA 3'-end-processing factor FIP1 | FIP1L1 | 0.64 | 0.55 | 0.17 | -0.02 | -0.84 | -0.78 | 0.77 | -0.78 | 1.96 | 1.14 | -0.83 | -0.78 |
| Q12849 | G-rich sequence factor 1 | GRSF1 | 0.64 | 0.99 | 1.06 | -0.28 | -1.29 | 0.53 | -1.16 | 0.52 | 0.03 | -1.46 | 1.11 | 0.94 |
| Q04446 | 1,4-alpha-glucan-branching enzyme | GBE1 | 0.64 | 1.54 | -0.22 | -0.08 | -1.58 | 0.33 | -0.06 | 0.40 | -1.76 | 1.11 | 0.66 | 1.19 |
| Q9H165 | B-cell lymphoma/leukemia 11A | BCL11A | 0.64 | 1.05 | 1.09 | -0.63 | -0.75 | -0.40 | -0.51 | -0.54 | -0.58 | 0.41 | 2.33 | -0.42 |
| Q9HC35 | Echinoderm microtubule-associated protein-like 4 | EML4 | 0.64 | 1.33 | -0.06 | -0.26 | -0.54 | -0.31 | -0.34 | -0.21 | -0.10 | -0.70 | -0.28 | 2.79 |
| Q9UBR2 | Cathepsin Z | CTSZ | 0.64 | 0.83 | -0.45 | 1.29 | -0.76 | -0.46 | -0.62 | -0.60 | -0.61 | 2.04 | -0.65 | 0.82 |
| Q5VZL5 | Zinc finger MYM-type protein 4 | ZMYM4 | 0.64 | 0.75 | 0.43 | -0.04 | -1.14 | -0.69 | 0.52 | 0.40 | 2.29 | -0.17 | -0.94 | -0.66 |
| Q7L1I2 | Synaptic vesicle glycoprotein 2B | SV2B | 0.64 | 1.23 | 0.44 | 0.95 | -2.03 | -0.28 | -0.43 | 0.17 | 0.72 | 1.43 | 0.00 | -0.97 |
| Q04828 | Aldo-keto reductase family 1 member C1 | AKR1C1 | 0.64 | 1.55 | -0.29 | -0.94 | -0.86 | 0.62 | -0.15 | 1.67 | -1.36 | -0.52 | 0.57 | 1.27 |
| P27361 | Mitogen-activated protein kinase 3 | MAPK3 | 0.64 | 1.60 | -0.57 | 0.91 | -0.31 | -0.04 | -1.63 | 0.75 | 1.60 | -1.32 | 0.10 | 0.51 |
| P20023 | Complement receptor type 2 | CR2 | 0.63 | 0.74 | 0.48 | -0.59 | 0.59 | -0.68 | -0.72 | 2.06 | -0.67 | -0.89 | 1.08 | -0.67 |
| P26447 | Protein S100-A4 | S100A4 | 0.63 | 1.05 | -0.26 | -0.46 | -0.70 | -0.41 | 0.61 | -0.31 | -0.40 | 2.67 | -0.30 | -0.44 |
| O76021 | Ribosomal L1 domain-containing protein 1 | RSL1D1 | 0.63 | 0.75 | 1.06 | -1.83 | -1.20 | 0.01 | 1.04 | 1.21 | -0.57 | 0.43 | -0.17 | 0.03 |
| Q9Y4Z0 | U6 snRNA-associated Sm-like protein LSm4 | LSM4 | 0.63 | 0.74 | -0.62 | 0.76 | 0.54 | -0.73 | -0.86 | -0.69 | -0.78 | 1.69 | -0.72 | 1.41 |
| O75146 | Huntingtin-interacting protein 1-related protein | HIP1R | 0.63 | 2.16 | 0.53 | -0.21 | -0.62 | 0.19 | -1.90 | -0.03 | -0.98 | 1.64 | 0.80 | 0.59 |
| Q5VTR2 | E3 ubiquitin-protein ligase BRE1A | RNF20 | 0.63 | 0.73 | -0.74 | -1.00 | -1.04 | 0.85 | 1.03 | -0.92 | -0.98 | 0.59 | 1.12 | 1.09 |
| O43293 | Death-associated protein kinase 3 | DAPK3 | 0.63 | 1.14 | 1.57 | -0.73 | -0.84 | -0.61 | -0.66 | 1.06 | 0.64 | -0.99 | -0.67 | 1.24 |
| Q9NQA3 | WAS protein family homolog 6 | WASH6P | 0.63 | 1.30 | -0.02 | -0.17 | -0.67 | -0.37 | -0.24 | 2.77 | -0.24 | -0.72 | -0.22 | -0.13 |
| P13798 | Acylamino-acid-releasing enzyme | APEH | 0.63 | 1.07 | -0.54 | -0.36 | 0.46 | -0.47 | -0.33 | 0.53 | -1.51 | 2.34 | -0.05 | -0.06 |
| Q16363 | Laminin subunit alpha-4 | LAMA4 | 0.63 | 0.72 | -1.55 | 0.43 | 0.23 | 0.63 | -0.62 | -1.78 | 0.15 | 1.27 | 0.66 | 0.57 |
| P35080 | Profilin-2 | PFN2 | 0.63 | 3.30 | 0.14 | -1.08 | -1.23 | 0.57 | -1.00 | 1.59 | 0.70 | -1.02 | 0.60 | 0.74 |
| P49754 | Vacuolar protein sorting-associated protein 41 homolog | VPS41 | 0.63 | 0.59 | 1.80 | -0.67 | -0.84 | -0.35 | -0.69 | 1.33 | 0.98 | -0.96 | -0.01 | -0.60 |
| P30044 | Peroxiredoxin-5, mitochondrial | PRDX5 | 0.63 | 2.58 | -1.64 | -0.58 | 0.62 | -0.95 | 0.30 | 1.15 | 0.65 | -0.59 | -0.47 | 1.51 |
| P0DMV9 | Heat shock 70 kDa protein 1B | HSPA1B | 0.63 | 3.14 | -0.66 | 0.82 | 0.36 | -1.19 | -1.93 | 0.58 | 0.88 | 0.78 | -0.46 | 0.83 |
| O14576 | Cytoplasmic dynein 1 intermediate chain 1 | DYNC1I1 | 0.63 | 1.45 | -1.21 | 0.61 | 0.19 | 0.76 | -1.89 | -0.80 | -0.24 | 1.09 | 0.74 | 0.74 |
| A6NHL2 | Tubulin alpha chain-like 3 | TUBAL3 | 0.63 | 0.37 | -0.84 | -0.91 | -1.09 | 1.07 | 1.27 | -0.91 | -0.91 | 0.86 | 0.89 | 0.57 |
| P07951 | Tropomyosin beta chain | TPM2 | 0.63 | 0.84 | 1.09 | -0.15 | 0.81 | -1.75 | -1.02 | 0.85 | 0.63 | 0.92 | -0.48 | -0.91 |
| Q9BSJ2 | Gamma-tubulin complex component 2 | TUBGCP2 | 0.63 | 0.75 | -0.44 | -0.77 | -0.69 | 0.59 | 0.38 | 1.15 | 1.60 | -1.79 | -0.31 | 0.28 |
| P29218 | Inositol monophosphatase 1 | IMPA1 | 0.63 | 0.69 | -0.82 | -0.16 | -0.53 | -0.25 | 0.89 | -0.69 | -1.01 | 2.38 | 0.16 | 0.03 |
| Q9NY33 | Dipeptidyl peptidase 3 | DPP3 | 0.62 | 0.59 | 0.74 | -0.44 | 0.52 | -0.53 | -1.05 | 1.42 | -0.99 | 1.57 | -0.18 | -1.06 |
| Q9NZ43 | Vesicle transport protein USE1 | USE1 | 0.62 | 0.80 | 0.57 | 0.82 | -1.94 | 0.60 | -1.01 | 0.03 | -0.92 | -0.13 | 0.89 | 1.10 |
| P29401 | Transketolase | TKT | 0.62 | 1.10 | -1.24 | -0.94 | 0.41 | -0.61 | 1.15 | 0.65 | -0.78 | 1.88 | -0.22 | -0.30 |
| P62979 | Ubiquitin-40S ribosomal protein S27a | RPS27A | 0.62 | 4.77 | -1.84 | -0.62 | 0.17 | -0.68 | -0.19 | -0.38 | 0.89 | 1.83 | 0.19 | 0.61 |
| Q8TDQ7 | Glucosamine-6-phosphate isomerase 2 | GNPDA2 | 0.62 | 0.56 | -0.81 | -0.25 | 0.44 | -0.09 | -0.03 | -0.90 | -0.93 | 2.45 | -0.39 | 0.51 |
| P62258 | 14-3-3 protein epsilon | YWHAE | 0.62 | 1.91 | 0.28 | 1.18 | -0.65 | -1.26 | -1.39 | 1.39 | 0.02 | 0.99 | -0.78 | 0.24 |
| P02671 | Fibrinogen alpha chain | FGA | 0.62 | 1.17 | -1.37 | -0.46 | 2.07 | -0.47 | -1.10 | 0.99 | -0.06 | 0.45 | -0.11 | 0.07 |
| Q86X95 | Corepressor interacting with RBPJ 1 | CIR1 | 0.62 | 0.68 | 0.10 | -1.09 | -1.13 | -0.42 | 1.69 | 0.37 | -1.06 | 1.21 | -0.43 | 0.75 |
| Q9H553 | Alpha-1,3/1,6-mannosyltransferase ALG2 | ALG2 | 0.62 | 0.76 | 0.42 | -0.19 | -0.54 | -0.73 | 0.09 | 0.40 | 1.84 | -2.03 | 0.27 | 0.47 |
| Q8N1G0 | Zinc finger protein 687 | ZNF687 | 0.62 | 0.70 | 0.23 | -0.45 | -0.81 | -0.35 | 0.50 | 0.54 | -1.06 | 2.38 | -0.21 | -0.76 |
| P14618 | Pyruvate kinase PKM | PKM | 0.62 | 1.59 | -0.38 | -1.40 | 1.04 | 0.06 | -0.96 | 0.10 | -0.89 | 1.87 | 0.74 | -0.18 |
| Q9UHA4 | Ragulator complex protein LAMTOR3 | LAMTOR3 | 0.62 | 1.01 | -0.37 | -0.58 | -0.91 | 1.47 | -0.76 | -0.54 | -0.60 | 1.65 | -0.52 | 1.16 |
| P62888 | 60S ribosomal protein L30 | RPL30 | 0.62 | 2.23 | -1.03 | -1.43 | 0.33 | 0.75 | -0.66 | -0.75 | -0.28 | 1.80 | 0.87 | 0.41 |
| O43427 | Acidic fibroblast growth factor intracellular-binding protein | FIBP | 0.62 | 0.78 | -0.71 | -0.51 | 0.10 | 1.16 | -0.98 | -0.87 | 0.56 | -1.27 | 1.12 | 1.41 |
| O75208 | Ubiquinone biosynthesis protein COQ9, mitochondrial | COQ9 | 0.62 | 1.34 | -0.06 | -0.42 | -0.51 | -0.28 | -0.24 | -0.12 | 2.79 | -0.74 | -0.27 | -0.15 |
| P62841 | 40S ribosomal protein S15 | RPS15 | 0.61 | 1.03 | 0.26 | -1.48 | 1.46 | -0.11 | -1.31 | -0.13 | 1.35 | -0.60 | -0.23 | 0.79 |
| Q7L523 | Ras-related GTP-binding protein A | RRAGA | 0.61 | 1.37 | 0.25 | -0.50 | -2.34 | 0.52 | 0.60 | 0.43 | -0.74 | -0.11 | 0.90 | 0.99 |
| P25789 | Proteasome subunit alpha type-4 | PSMA4 | 0.61 | 1.59 | -0.67 | -1.67 | 0.07 | -0.19 | 0.84 | -0.41 | -0.62 | 2.07 | 0.39 | 0.21 |
| O95865 | N(G),N(G)-dimethylarginine dimethylaminohydrolase 2 | DDAH2 | 0.61 | 1.19 | -0.61 | -1.32 | -0.26 | 0.01 | 0.85 | 0.30 | -0.91 | 2.23 | 0.12 | -0.42 |
| P10746 | Uroporphyrinogen-III synthase | UROS | 0.61 | 0.95 | -0.15 | 1.30 | -0.22 | 0.06 | -2.10 | 0.75 | 1.36 | -0.56 | -0.15 | -0.28 |
| Q9Y485 | DmX-like protein 1 | DMXL1 | 0.61 | 1.98 | -2.13 | 0.67 | -0.15 | 0.17 | -0.46 | -0.35 | 0.18 | 1.27 | -0.52 | 1.31 |
| Q16698 | 2,4-dienoyl-CoA reductase, mitochondrial | DECR1 | 0.61 | 0.50 | -1.62 | -0.02 | 0.00 | 0.46 | 0.53 | -1.73 | 0.33 | 1.61 | 0.44 | 0.00 |
| Q02108 | Guanylate cyclase soluble subunit alpha-1 | GUCY1A1 | 0.61 | 0.90 | 0.80 | 0.65 | -1.35 | 0.59 | -1.77 | 1.15 | -0.94 | 0.15 | 0.66 | 0.05 |
| Q07955 | Serine/arginine-rich splicing factor 1 | SRSF1 | 0.61 | 0.94 | -1.93 | 0.13 | 0.72 | -0.49 | 0.47 | -0.47 | -0.18 | 1.89 | -0.54 | 0.39 |
| O75116 | Rho-associated protein kinase 2 | ROCK2 | 0.61 | 1.10 | 1.22 | -0.04 | -1.20 | -0.82 | -0.41 | 0.29 | 1.92 | -1.01 | -0.43 | 0.48 |
| Q8WUA4 | General transcription factor 3C polypeptide 2 | GTF3C2 | 0.61 | 0.66 | 0.08 | -0.48 | -1.75 | 0.67 | 0.67 | -0.21 | -1.55 | 1.07 | 0.92 | 0.58 |
| P41567 | Eukaryotic translation initiation factor 1 | EIF1 | 0.60 | 0.47 | 0.17 | -1.42 | 1.49 | -1.36 | 0.50 | 0.19 | -0.33 | 1.47 | -0.57 | -0.14 |
| P25788 | Proteasome subunit alpha type-3 | PSMA3 | 0.60 | 1.31 | -0.81 | -1.41 | 0.16 | -0.33 | 0.99 | -0.43 | -0.67 | 2.12 | 0.09 | 0.30 |
| P17174 | Aspartate aminotransferase, cytoplasmic | GOT1 | 0.60 | 0.69 | -0.60 | -0.37 | 0.46 | -0.37 | 0.00 | -0.33 | -0.81 | 2.67 | -0.23 | -0.43 |
| Q9UH65 | Switch-associated protein 70 | SWAP70 | 0.60 | 1.46 | -2.13 | 0.52 | 0.10 | 0.88 | -0.91 | 0.38 | -0.18 | -0.41 | 0.29 | 1.47 |
| Q14677 | Clathrin interactor 1 | CLINT1 | 0.60 | 1.28 | -0.23 | -0.01 | -1.09 | -0.16 | 0.06 | 1.11 | 1.18 | -1.54 | -0.78 | 1.45 |
| Q5FWE3 | Proline-rich transmembrane protein 3 | PRRT3 | 0.60 | 0.94 | 0.97 | 0.92 | -1.32 | -0.96 | -0.72 | 0.99 | 1.52 | -0.64 | -0.24 | -0.54 |
| P51911 | Calponin-1 | CNN1 | 0.60 | 1.11 | 1.49 | -0.42 | -1.64 | -0.58 | -0.10 | 1.12 | 0.92 | -0.89 | -0.53 | 0.62 |
| Q92597 | Protein NDRG1 | NDRG1 | 0.60 | 1.00 | -1.79 | 0.08 | -0.12 | -0.89 | 1.57 | 0.06 | -0.97 | 1.01 | 0.61 | 0.44 |
| Q9C0K0 | B-cell lymphoma/leukemia 11B | BCL11B | 0.60 | 0.39 | 1.81 | -0.55 | -0.68 | -0.52 | -0.59 | 1.66 | 0.68 | -0.78 | -0.55 | -0.48 |
| Q96KP4 | Cytosolic non-specific dipeptidase | CNDP2 | 0.60 | 2.76 | -0.83 | -0.08 | 0.64 | -0.24 | -1.82 | 0.69 | -0.85 | 1.73 | 0.46 | 0.31 |
| Q99436 | Proteasome subunit beta type-7 | PSMB7 | 0.60 | 1.63 | -0.97 | -1.58 | 0.29 | -0.43 | 1.03 | 0.09 | -0.35 | 1.99 | -0.28 | 0.20 |
| P07900 | Heat shock protein HSP 90-alpha | HSP90AA1 | 0.60 | 1.91 | -0.05 | -0.41 | 0.19 | -0.39 | -1.25 | 1.46 | 1.27 | -1.39 | -0.51 | 1.06 |
| Q8NDV7 | Trinucleotide repeat-containing gene 6A protein | TNRC6A | 0.60 | 0.63 | 0.64 | 0.80 | -0.93 | -0.63 | -0.69 | -0.73 | -0.64 | 2.00 | -0.71 | 0.87 |
| P48643 | T-complex protein 1 subunit epsilon | CCT5 | 0.60 | 1.45 | 1.51 | -0.63 | -1.59 | -0.66 | -0.16 | 1.49 | 0.42 | 0.64 | -0.52 | -0.50 |
| P11586 | C-1-tetrahydrofolate synthase, cytoplasmic | MTHFD1 | 0.60 | 1.49 | -0.14 | 0.23 | -1.27 | 0.28 | -0.68 | 1.27 | -0.37 | -1.43 | 0.45 | 1.66 |
| Q8TCD5 | 5'(3')-deoxyribonucleotidase, cytosolic type | NT5C | 0.60 | 1.08 | 0.79 | -1.89 | 0.21 | -0.01 | -0.33 | 1.26 | -1.34 | -0.16 | 0.45 | 1.02 |
| P42677 | 40S ribosomal protein S27 | RPS27 | 0.60 | 5.35 | -0.53 | -0.46 | -0.90 | -0.20 | -1.37 | 1.16 | 1.10 | -0.63 | 0.15 | 1.67 |
| Q9HC78 | Zinc finger and BTB domain-containing protein 20 | ZBTB20 | 0.60 | 0.53 | 0.41 | -1.17 | -2.01 | 0.82 | 1.26 | 0.61 | -0.56 | -0.16 | 0.64 | 0.16 |
| Q96NN9 | Apoptosis-inducing factor 3 | AIFM3 | 0.60 | 1.59 | -2.05 | -0.25 | -0.46 | 0.34 | 0.78 | -0.51 | 0.27 | -0.57 | 1.49 | 0.96 |
| P13796 | Plastin-2 | LCP1 | 0.60 | 0.82 | -0.93 | 0.70 | -0.80 | -0.29 | 0.32 | -0.09 | -0.65 | 2.46 | -0.13 | -0.58 |

| | | | | | | | | | | | | | | |
|---|---|---|---|---|---|---|---|---|---|---|---|---|---|---|
| Q9UGI8 | Testin | TES | 0.60 | 1.20 | -0.01 | -0.32 | -0.60 | -0.20 | -0.25 | -0.16 | -0.26 | -0.85 | -0.11 | 2.76 |
| Q9UPQ0 | LIM and calponin homology domains-containing protein 1 | LIMCH1 | 0.60 | 0.67 | -2.23 | 0.83 | -0.43 | 0.72 | 0.28 | -0.91 | -0.42 | 0.71 | 0.70 | 0.75 |
| Q9NRN5 | Olfactomedin-like protein 3 | OLFML3 | 0.60 | 1.03 | -0.21 | 0.89 | -0.80 | -0.43 | -0.64 | 1.38 | 1.85 | -1.04 | -0.61 | -0.40 |
| Q86XL3 | Ankyrin repeat and LEM domain-containing protein 2 | ANKLE2 | 0.60 | 0.74 | 1.86 | -0.63 | -1.02 | -0.24 | -0.88 | 1.49 | -0.50 | 0.51 | -0.73 | 0.14 |
| Q9Y3P9 | Rab GTPase-activating protein 1 | RABGAP1 | 0.60 | 0.48 | 0.38 | 0.06 | -1.48 | 0.09 | 0.32 | 1.15 | 1.21 | -1.93 | -0.02 | 0.22 |
| P22676 | Calretinin | CALB2 | 0.60 | 0.68 | 0.40 | -0.49 | 1.00 | -1.88 | 0.12 | 0.24 | 0.29 | 1.45 | -1.33 | 0.19 |
| Q8ND56 | Protein LSM14 homolog A | LSM14A | 0.60 | 0.69 | 1.09 | -1.52 | -1.67 | 0.74 | 0.50 | -0.84 | -0.08 | 0.32 | 0.84 | 0.63 |
| O15021 | Microtubule-associated serine/threonine-protein kinase 4 | MAST4 | 0.60 | 0.55 | 0.80 | 0.10 | -1.63 | 0.62 | -0.60 | 1.00 | 0.38 | -1.78 | 0.51 | 0.60 |
| P51957 | Serine/threonine-protein kinase Nek4 | NEK4 | 0.60 | 1.20 | -0.14 | -0.26 | -0.45 | -0.33 | -0.20 | 2.80 | -0.18 | -0.74 | -0.27 | -0.23 |
| O43488 | Aflatoxin B1 aldehyde reductase member 2 | AKR7A2 | 0.60 | 1.06 | -1.03 | 0.27 | -0.59 | 0.57 | -0.45 | -0.99 | -1.28 | 1.61 | 0.94 | 0.95 |
| O43314 | Inositol hexakisphosphate and diphosphoinositol-pentakisphosphat | PPIP5K2 | 0.59 | 0.33 | 1.42 | 0.05 | -0.62 | -0.43 | -0.87 | 1.35 | 1.37 | -1.04 | -0.38 | -0.85 |
| P16615 | Sarcoplasmic/endoplasmic reticulum calcium ATPase 2 | ATP2A2 | 0.59 | 1.53 | 0.19 | 0.07 | 0.32 | -0.21 | -1.96 | 0.61 | 1.42 | -1.21 | -0.26 | 1.03 |
| Q8TF68 | Zinc finger protein 384 | ZNF384 | 0.59 | 0.78 | 0.09 | -1.11 | -1.22 | 0.84 | 0.45 | 1.13 | -1.07 | 0.62 | 1.26 | -0.99 |
| O75864 | Protein phosphatase 1 regulatory subunit 37 | PPP1R37 | 0.59 | 0.72 | -0.02 | -0.24 | -1.57 | -0.37 | 1.32 | -1.28 | 1.49 | 0.84 | -0.09 | -0.08 |
| P24539 | ATP synthase F(0) complex subunit B1, mitochondrial | ATP5PB | 0.59 | 1.07 | -0.69 | -1.08 | 0.81 | 0.78 | -1.03 | 0.77 | -1.00 | 1.37 | 0.92 | -0.84 |
| Q76N89 | E3 ubiquitin-protein ligase HECW1 | HECW1 | 0.59 | 0.49 | 0.49 | 0.54 | -0.92 | -0.46 | -0.28 | 1.45 | 1.14 | 0.60 | -1.68 | -0.86 |
| Q96SI9 | Spermatid perinuclear RNA-binding protein | STRBP | 0.59 | 0.75 | -1.33 | 0.37 | 1.02 | 0.48 | -1.46 | -1.32 | 1.15 | 0.72 | 0.04 | 0.32 |
| P07737 | Profilin-1 | PFN1 | 0.59 | 3.22 | -0.68 | -0.32 | 0.72 | -0.12 | -2.20 | 1.28 | 0.29 | -0.44 | 0.56 | 0.92 |
| Q2TAA2 | Isoamyl acetate-hydrolyzing esterase 1 homolog | IAH1 | 0.59 | 0.57 | -1.02 | 0.25 | 0.41 | 0.72 | -1.09 | -1.11 | -1.14 | 1.57 | 0.58 | 0.82 |
| Q9P253 | Vacuolar protein sorting-associated protein 18 homolog | VPS18 | 0.59 | 1.67 | -0.27 | -0.62 | 0.97 | -1.20 | -0.58 | 0.04 | 2.01 | 0.41 | -1.20 | 0.44 |
| O14950 | Myosin regulatory light chain 12B | MYL12B | 0.59 | 2.23 | -2.46 | 0.51 | 0.52 | -0.22 | -0.46 | -0.33 | 0.54 | 1.11 | 0.08 | 0.70 |
| Q9NR31 | GTP-binding protein SAR1a | SAR1A | 0.59 | 2.17 | 0.23 | 0.50 | -1.99 | 0.34 | -1.10 | 0.38 | -0.96 | 1.03 | 0.94 | 0.63 |
| Q9BQA1 | Methylosome protein 50 | WDR77 | 0.59 | 0.50 | 0.64 | -1.60 | 0.09 | -0.23 | 0.46 | 0.80 | -1.83 | 1.30 | 0.19 | 0.19 |
| Q9UHC6 | Contactin-associated protein-like 2 | CNTNAP2 | 0.59 | 1.88 | 0.03 | -1.95 | -1.41 | 1.22 | 0.21 | 0.49 | 0.02 | -0.13 | 1.13 | 0.38 |
| O15354 | Prosaposin receptor GPR37 | GPR37 | 0.59 | 0.66 | 0.10 | -1.29 | -1.11 | 0.52 | 0.95 | -0.88 | -0.44 | 1.52 | 1.16 | -0.54 |
| P20674 | Cytochrome c oxidase subunit 5A, mitochondrial | COX5A | 0.59 | 0.37 | 1.53 | -0.46 | -0.90 | 0.09 | -0.76 | 1.04 | 1.53 | -0.94 | -0.81 | -0.32 |
| P83916 | Chromobox protein homolog 1 | CBX1 | 0.59 | 1.17 | 0.27 | -0.89 | -0.39 | -0.34 | 0.02 | 1.37 | 0.62 | 0.13 | -2.02 | 1.23 |
| P08865 | 40S ribosomal protein SA | RPSA | 0.59 | 1.00 | 1.30 | -1.21 | -0.06 | -0.47 | -0.71 | 0.71 | 1.81 | 0.08 | -0.33 | -1.11 |
| O43852 | Calumenin | CALU | 0.59 | 2.12 | -0.11 | -0.52 | -0.74 | -0.38 | -0.36 | 1.85 | 1.74 | -1.18 | -0.12 | -0.18 |
| O75940 | Survival of motor neuron-related-splicing factor 30 | SMNDC1 | 0.58 | 1.04 | 0.11 | -0.33 | -0.49 | -0.23 | -0.29 | -0.30 | -0.30 | -0.77 | -0.18 | 2.78 |
| A7E2V4 | Zinc finger SWIM domain-containing protein 8 | ZSWIM8 | 0.58 | 1.45 | 1.05 | -0.23 | -0.88 | -1.52 | 0.06 | 0.83 | 0.55 | 1.57 | -0.33 | -1.10 |
| P02745 | Complement C1q subcomponent subunit A | C1QA | 0.58 | 2.93 | 0.26 | -1.49 | 0.16 | -1.10 | -0.27 | 1.14 | 1.21 | -0.94 | -0.27 | 1.30 |
| Q9UBK8 | Methionine synthase reductase | MTRR | 0.58 | 0.84 | -0.44 | -0.51 | -0.79 | 1.37 | -0.64 | -0.48 | -0.46 | -0.97 | 1.43 | 1.49 |
| P48444 | Coatomer subunit delta | ARCN1 | 0.58 | 1.26 | -0.38 | 0.74 | -0.56 | 0.57 | -1.74 | -0.18 | -0.06 | -0.73 | 0.36 | 1.98 |
| Q86X76 | Deaminated glutathione amidase | NIT1 | 0.58 | 2.05 | 1.34 | -0.99 | -1.62 | 0.51 | -1.24 | 1.18 | 0.40 | -0.19 | 0.43 | 0.18 |
| P49327 | Fatty acid synthase | FASN | 0.58 | 0.90 | 1.20 | -0.30 | -1.22 | -0.57 | -0.17 | 1.06 | 1.76 | -1.08 | -0.46 | -0.21 |
| Q13428 | Treacle protein | TCOF1 | 0.58 | 2.13 | -1.10 | 0.03 | -0.16 | 0.88 | -1.64 | -0.05 | -0.88 | 0.38 | 0.98 | 1.55 |
| Q9P2W9 | Syntaxin-18 | STX18 | 0.58 | 1.73 | 1.24 | 0.41 | -1.75 | 0.02 | -1.73 | 0.82 | 0.26 | 0.42 | 0.53 | -0.22 |
| Q9BVK6 | Transmembrane emp24 domain-containing protein 9 | TMED9 | 0.58 | 0.58 | 1.36 | 0.69 | -1.02 | -0.83 | -0.94 | 1.02 | -0.94 | 1.18 | 0.28 | -0.81 |
| P62861 | 40S ribosomal protein S30 | FAU | 0.58 | 2.09 | -0.27 | -1.24 | 1.28 | -1.10 | -0.64 | -0.09 | 1.68 | 0.08 | -0.64 | 0.93 |
| Q9H329 | Band 4.1-like protein 4B | EPB41L4B | 0.58 | 1.00 | -0.40 | -2.25 | -0.23 | 0.86 | 0.85 | 0.22 | -0.82 | 0.86 | 0.89 | 0.02 |
| O75676 | Ribosomal protein S6 kinase alpha-4 | RPS6KA4 | 0.57 | 0.38 | 1.34 | -0.57 | -1.57 | 0.12 | 0.17 | 1.17 | 0.89 | -0.20 | 0.11 | -1.46 |
| P32121 | Beta-arrestin-2 | ARRB2 | 0.57 | 2.05 | -1.43 | -1.31 | 0.46 | 0.79 | -0.45 | -0.15 | -0.88 | 0.45 | 1.20 | 1.32 |
| Q93088 | Betaine--homocysteine S-methyltransferase 1 | BHMT | 0.57 | 0.75 | 0.20 | 1.04 | -1.45 | 0.60 | -1.30 | 0.22 | -1.29 | 1.25 | 0.07 | 0.67 |
| Q8N1W1 | Rho guanine nucleotide exchange factor 28 | ARHGEF28 | 0.57 | 1.47 | -0.09 | -0.37 | -0.44 | -0.30 | -0.41 | -0.04 | 2.76 | -0.85 | -0.12 | -0.13 |
| Q96DI7 | U5 small nuclear ribonucleoprotein 40 kDa protein | SNRNP40 | 0.57 | 0.74 | -0.02 | 0.27 | -2.43 | 1.01 | 0.26 | -0.25 | -0.59 | -0.02 | 0.90 | 0.86 |
| P60900 | Proteasome subunit alpha type-6 | PSMA6 | 0.57 | 1.18 | -0.64 | -2.13 | 0.21 | -0.03 | 1.27 | -0.07 | -0.25 | 1.45 | 0.43 | -0.24 |
| P51692 | Signal transducer and activator of transcription 5B | STAT5B | 0.57 | 1.43 | 0.16 | -1.28 | -2.26 | 0.83 | 0.96 | 0.48 | 0.20 | 0.40 | 0.45 | 0.05 |
| Q13838 | Spliceosome RNA helicase DDX39B | DDX39B | 0.57 | 1.50 | 1.07 | -0.41 | -1.30 | 0.28 | -1.19 | 1.20 | 0.77 | -1.40 | 0.57 | 0.42 |
| P05413 | Fatty acid-binding protein, heart | FABP3 | 0.57 | 0.85 | -0.40 | 0.46 | -0.75 | -0.80 | 0.46 | -0.22 | -0.70 | 2.53 | -0.53 | -0.06 |
| P26599 | Polypyrimidine tract-binding protein 1 | PTBP1 | 0.57 | 1.04 | -1.34 | -0.86 | 0.32 | 0.78 | -0.08 | 0.08 | -1.73 | 1.02 | 1.08 | 0.74 |
| P29728 | 2'-5'-oligoadenylate synthase 2 | OAS2 | 0.57 | 0.87 | -0.31 | 1.21 | -0.80 | -0.44 | -0.69 | -0.43 | -0.55 | -0.89 | 0.87 | 2.03 |
| P54577 | Tyrosine--tRNA ligase, cytoplasmic | YARS | 0.57 | 1.61 | 0.37 | -0.43 | -1.13 | -0.51 | 0.01 | 1.58 | 1.61 | -1.24 | -0.64 | 0.37 |
| P37235 | Hippocalcin-like protein 1 | HPCAL1 | 0.57 | 1.05 | -0.46 | -0.62 | -0.89 | -0.52 | 1.28 | -0.64 | -0.66 | 1.74 | 1.26 | -0.50 |
| P05090 | Apolipoprotein D | APOD | 0.57 | 1.19 | 0.57 | -0.32 | -1.97 | -0.45 | 0.85 | 0.59 | -0.83 | 1.50 | -0.47 | 0.53 |
| P50542 | Peroxisomal targeting signal 1 receptor | PEX5 | 0.57 | 1.45 | 0.75 | -2.43 | 0.38 | 0.02 | -0.27 | 1.18 | -0.63 | 0.70 | 0.21 | 0.09 |
| P62987 | Ubiquitin-60S ribosomal protein L40 | UBA52 | 0.57 | 1.55 | 0.10 | -2.36 | 1.05 | -0.05 | -0.36 | -0.65 | 0.32 | 0.16 | 0.86 | 0.94 |
| P52292 | Importin subunit alpha-1 | KPNA2 | 0.57 | 0.82 | -0.70 | 0.75 | -2.52 | 0.47 | 0.99 | 0.12 | 0.39 | 0.38 | 0.23 | -0.11 |
| P50440 | Glycine amidinotransferase, mitochondrial | GATM | 0.57 | 0.66 | -0.51 | -0.98 | 0.27 | -0.40 | 0.79 | -0.58 | -0.96 | 2.33 | 0.36 | -0.31 |
| Q9NVX7 | Kelch repeat and BTB domain-containing protein 4 | KBTBD4 | 0.57 | 0.68 | -0.85 | -0.99 | 0.96 | 1.04 | -1.00 | -0.88 | -0.87 | 0.36 | 0.85 | 1.39 |
| Q69YQ0 | Cytospin-A | SPECC1L | 0.56 | 0.83 | -0.46 | 1.71 | -0.89 | -0.71 | -0.64 | 0.79 | -0.69 | -1.02 | 0.66 | 1.26 |
| P48729 | Casein kinase I isoform alpha | CSNK1A1 | 0.56 | 0.89 | 1.13 | -1.63 | -0.89 | 0.72 | -0.37 | 0.84 | -0.26 | -1.20 | 0.71 | 0.95 |
| O14795 | Protein unc-13 homolog B | UNC13B | 0.56 | 0.91 | -0.32 | -0.42 | -0.84 | 0.99 | -0.48 | -0.58 | -0.45 | -0.90 | 0.85 | 2.17 |
| Q9UBL6 | Copine-7 | CPNE7 | 0.56 | 0.99 | -1.49 | -0.13 | 0.29 | -0.70 | 0.88 | 0.11 | -0.39 | 2.17 | -0.73 | -0.02 |
| Q8IY45 | Protein AMN1 homolog | AMN1 | 0.56 | 0.66 | -1.21 | 0.20 | 0.73 | 0.99 | -1.53 | -0.02 | -1.36 | 0.99 | 0.39 | 0.82 |
| O94832 | Unconventional myosin-Id | MYO1D | 0.56 | 1.54 | -0.60 | 0.15 | -1.02 | 0.44 | -0.59 | 0.48 | -1.79 | 1.61 | 0.92 | 0.41 |
| P35606 | Coatomer subunit beta' | COPB2 | 0.56 | 1.31 | 1.34 | -0.96 | -0.90 | -0.21 | -0.68 | 1.68 | 1.17 | -0.58 | -0.62 | -0.23 |
| Q8N3U4 | Cohesin subunit SA-2 | STAG2 | 0.56 | 1.49 | 0.38 | -1.42 | -1.64 | 0.22 | 0.89 | 0.92 | 1.03 | -1.08 | 0.36 | 0.34 |
| P56556 | NADH dehydrogenase [ubiquinone] 1 alpha subcomplex subunit 6 | NDUFA6 | 0.56 | 0.62 | -0.44 | -0.54 | 1.50 | -0.65 | -0.65 | -0.59 | -0.66 | 2.12 | -0.30 | 0.20 |
| P18074 | General transcription and DNA repair factor IIH helicase subunit : | ERCC2 | 0.56 | 0.53 | -0.05 | 0.19 | -1.11 | 0.13 | 0.14 | -0.55 | -1.46 | 0.51 | -0.04 | 2.24 |
| O43615 | Mitochondrial import inner membrane translocase subunit TIM44 | TIMM44 | 0.56 | 0.73 | -0.58 | -0.73 | 1.41 | -0.66 | -0.34 | -0.77 | 1.78 | -1.04 | 0.07 | 0.86 |
| Q86U44 | N6-adenosine-methyltransferase catalytic subunit | METTL3 | 0.56 | 1.02 | 1.05 | -1.38 | 0.00 | -0.91 | 0.07 | -0.66 | 2.16 | -0.11 | -0.06 | -0.16 |
| P22059 | Oxysterol-binding protein 1 | OSBP | 0.56 | 2.33 | -0.88 | 0.53 | 0.69 | -0.21 | -2.31 | 0.28 | 0.87 | 0.27 | -0.29 | 1.04 |
| O95169 | NADH dehydrogenase [ubiquinone] 1 beta subcomplex subunit 8, r | NDUFB8 | 0.56 | 1.50 | -0.79 | -0.88 | -1.25 | 0.46 | 0.90 | -0.78 | 1.60 | 0.78 | 0.76 | -0.81 |
| Q9BRA2 | Thioredoxin domain-containing protein 17 | TXNDC17 | 0.56 | 1.23 | -0.87 | -0.54 | -0.51 | -0.34 | 0.90 | 1.04 | -1.93 | 1.05 | 0.36 | 0.84 |
| P01011 | Alpha-1-antichymotrypsin | SERPINA3 | 0.56 | 0.77 | 0.10 | -0.80 | 1.04 | -1.76 | 0.50 | -0.18 | 0.24 | 1.53 | -1.12 | 0.47 |
| P02792 | Ferritin light chain | FTL | 0.56 | 2.36 | -0.15 | -1.09 | -1.48 | -0.84 | 1.40 | -0.25 | 0.74 | 1.42 | -0.25 | 0.50 |
| Q86UV5 | Ubiquitin carboxyl-terminal hydrolase 48 | USP48 | 0.56 | 0.55 | -0.14 | -1.15 | -1.38 | 0.51 | 1.46 | -1.14 | 1.26 | 0.35 | 0.53 | -0.30 |
| Q14696 | LRP chaperone MESD | MESD | 0.56 | 0.74 | -0.92 | 0.35 | 1.90 | -1.09 | -1.15 | 0.72 | 0.99 | 0.01 | -0.18 | -0.62 |
| Q7Z3C6 | Autophagy-related protein 9A | ATG9A | 0.55 | 3.13 | -0.20 | -0.51 | -1.65 | -0.32 | 0.12 | 0.40 | 0.74 | 1.37 | -1.26 | 1.30 |
| P08567 | Pleckstrin | PLEK | 0.55 | 0.54 | 0.74 | 0.23 | 0.78 | -0.42 | -2.02 | 0.64 | 1.22 | -1.19 | -0.33 | 0.35 |
| O94906 | Pre-mRNA-processing factor 6 | PRPF6 | 0.55 | 0.75 | 0.82 | -0.53 | -1.34 | 0.28 | -0.15 | 0.43 | 1.73 | -1.60 | 0.60 | -0.23 |
| P78318 | Immunoglobulin-binding protein 1 | IGBP1 | 0.55 | 0.51 | 0.03 | -0.72 | 1.35 | 0.66 | -1.97 | -0.54 | -0.82 | 0.73 | 0.63 | 0.66 |

| | | | | | | | | | | | | | | |
|---|---|---|---|---|---|---|---|---|---|---|---|---|---|---|
| Q9Y303 | N-acetylglucosamine-6-phosphate deacetylase | AMDHD2 | 0.55 | 0.90 | -0.18 | 0.61 | -0.83 | 0.77 | -1.43 | -0.40 | -1.44 | 1.04 | 1.24 | 0.63 |
| Q96T23 | Remodeling and spacing factor 1 | RSF1 | 0.55 | 0.49 | 1.70 | 0.07 | -2.04 | -0.39 | 0.02 | 0.83 | 0.77 | 0.05 | -0.44 | -0.57 |
| Q9UI08 | Ena/VASP-like protein | EVL | 0.55 | 0.88 | -0.17 | -0.19 | -2.40 | 1.05 | 0.64 | -0.68 | 0.18 | 0.90 | 0.61 | 0.05 |
| Q13976 | cGMP-dependent protein kinase 1 | PRKG1 | 0.55 | 0.71 | 0.86 | -0.39 | 1.12 | -1.23 | -1.24 | 1.35 | -1.14 | -0.11 | -0.05 | 0.81 |
| Q86XE0 | Sorting nexin-32 | SNX32 | 0.55 | 0.69 | 0.91 | 1.06 | -0.97 | -1.06 | -0.79 | 0.35 | 0.50 | -0.01 | -1.40 | 1.41 |
| Q15334 | Lethal(2) giant larvae protein homolog 1 | LLGL1 | 0.55 | 1.65 | 0.07 | -2.28 | -0.22 | 0.28 | 0.47 | 1.38 | -0.94 | 0.07 | 0.57 | 0.59 |
| P05230 | Fibroblast growth factor 1 | FGF1 | 0.55 | 1.28 | -0.27 | -1.23 | 0.99 | 0.72 | -1.60 | 1.23 | -0.63 | -0.53 | 1.03 | 0.30 |
| O43164 | E3 ubiquitin-protein ligase Praja-2 | PJA2 | 0.55 | 0.70 | 0.56 | -1.33 | -1.59 | 0.62 | 0.87 | -1.20 | 0.63 | -0.08 | 1.13 | 0.38 |
| Q9Y388 | RNA-binding motif protein, X-linked 2 | RBMX2 | 0.55 | 0.76 | 1.05 | 0.07 | -1.19 | 0.30 | -1.15 | 0.26 | 1.82 | -0.60 | 0.48 | -1.05 |
| Q9BQ67 | Glutamate-rich WD repeat-containing protein 1 | GRWD1 | 0.55 | 0.50 | -0.12 | -0.26 | -2.11 | 0.90 | 0.94 | -0.61 | -0.89 | 0.75 | 0.82 | 0.58 |
| Q86UW7 | Calcium-dependent secretion activator 2 | CADPS2 | 0.55 | 0.61 | -0.22 | 1.52 | -2.18 | 0.14 | -0.04 | -0.26 | 0.92 | -0.72 | 0.24 | 0.60 |
| Q9HC38 | Glyoxalase domain-containing protein 4 | GLOD4 | 0.55 | 1.00 | -0.17 | -0.08 | -0.40 | -0.32 | -0.24 | -1.66 | 0.38 | 2.39 | 0.17 | -0.07 |
| P00568 | Adenylate kinase isoenzyme 1 | AK1 | 0.55 | 1.33 | 0.47 | 0.19 | -0.99 | -0.15 | -0.98 | 1.99 | -0.77 | -0.95 | 0.03 | 1.15 |
| P51809 | Vesicle-associated membrane protein 7 | VAMP7 | 0.55 | 1.29 | -2.12 | 0.44 | -0.67 | 0.54 | 0.41 | 0.77 | -1.20 | 0.93 | 0.66 | 0.24 |
| Q9UMS6 | Synaptopodin-2 | SYNPO2 | 0.55 | 0.75 | 0.19 | 0.97 | -1.27 | 0.37 | -1.17 | 1.27 | -0.29 | -1.34 | 0.08 | 1.19 |
| Q14999 | Cullin-7 | CUL7 | 0.55 | 0.48 | 0.86 | 0.66 | -1.18 | 0.17 | -1.14 | 1.34 | 1.14 | -1.38 | -0.16 | -0.31 |
| O43633 | Charged multivesicular body protein 2a | CHMP2A | 0.55 | 0.70 | 1.41 | -1.46 | -1.67 | 0.60 | 0.25 | -0.75 | 0.09 | 0.36 | 0.96 | 0.21 |
| P51151 | Ras-related protein Rab-9A | RAB9A | 0.54 | 1.60 | 0.76 | -0.53 | -1.98 | 0.63 | -0.52 | 0.98 | -0.05 | -1.02 | 1.01 | 0.72 |
| Q9P0K7 | Ankycorbin | RAI14 | 0.54 | 0.80 | -0.27 | -0.43 | 0.44 | -0.32 | -0.42 | -0.40 | -0.27 | -0.74 | -0.31 | 2.72 |
| Q96HD9 | N-acyl-aromatic-L-amino acid amidohydrolase (carboxylate-formi | ACY3 | 0.54 | 1.34 | 0.00 | -0.34 | -0.64 | -0.17 | -0.35 | -0.35 | -0.40 | 2.80 | -0.44 | -0.11 |
| O15394 | Neural cell adhesion molecule 2 | NCAM2 | 0.54 | 0.52 | -1.11 | 0.53 | -0.06 | 0.69 | -0.73 | -1.12 | -1.23 | 1.55 | 1.06 | 0.42 |
| P62140 | Serine/threonine-protein phosphatase PP1-beta catalytic subunit | PPP1CB | 0.54 | 2.21 | -0.01 | -0.72 | -1.99 | 0.33 | 0.36 | 1.09 | 0.16 | -1.09 | 0.67 | 1.20 |
| Q96IU4 | Protein ABHD14B | ABHD14B | 0.54 | 1.29 | -1.49 | 0.25 | 0.17 | 0.62 | -0.94 | 0.15 | -1.52 | 0.34 | 1.08 | 1.34 |
| P48147 | Prolyl endopeptidase | PREP | 0.54 | 0.21 | -1.56 | 0.62 | 0.54 | 0.61 | -0.51 | -0.68 | -1.60 | 0.88 | 0.68 | 1.01 |
| P04792 | Heat shock protein beta-1 | HSPB1 | 0.54 | 1.09 | 0.09 | 0.25 | -0.42 | -0.22 | -0.96 | 1.64 | -0.09 | -1.59 | -0.27 | 1.57 |
| Q8IYI6 | Exocyst complex component 8 | EXOC8 | 0.54 | 0.62 | 1.36 | 0.00 | -1.14 | -0.68 | -0.32 | 1.38 | 1.40 | -0.75 | -0.79 | -0.46 |
| P10412 | Histone H1.4 | HIST1H1E | 0.54 | 1.76 | 0.89 | -1.39 | -0.09 | 0.65 | -1.84 | -0.47 | -0.16 | 0.42 | 1.13 | 0.86 |
| P16083 | Ribosyldihydronicotinamide dehydrogenase [quinone] | NQO2 | 0.54 | 0.48 | 1.22 | -0.09 | -0.13 | -0.76 | -0.87 | 0.15 | 0.27 | 2.08 | -0.93 | -0.94 |
| Q9NP58 | ATP-binding cassette sub-family B member 6, mitochondrial | ABCB6 | 0.54 | 0.80 | 0.79 | -0.80 | -0.95 | -0.32 | 0.32 | 1.60 | -0.65 | -1.16 | -0.30 | 1.49 |
| Q96KQ4 | Apoptosis-stimulating of p53 protein 1 | PPP1R13B | 0.54 | 0.61 | 0.67 | 0.71 | -1.53 | -0.59 | -0.04 | 1.15 | 0.60 | -1.79 | 0.14 | 0.67 |
| O95376 | E3 ubiquitin-protein ligase ARIH2 | ARIH2 | 0.54 | 0.66 | 1.10 | 0.46 | -2.63 | 0.34 | -0.12 | 0.18 | 0.37 | 0.12 | 0.51 | -0.32 |
| A4D1P6 | WD repeat-containing protein 91 | WDR91 | 0.54 | 0.63 | 1.85 | -0.34 | -2.11 | -0.34 | 0.14 | 0.62 | 0.34 | -0.48 | -0.04 | 0.37 |
| Q9P2F6 | Rho GTPase-activating protein 20 | ARHGAP20 | 0.53 | 1.18 | 1.00 | -1.39 | -0.59 | 1.02 | -1.36 | 0.05 | -0.83 | 1.14 | 0.92 | 0.04 |
| P54105 | Methylosome subunit pICln | CLNS1A | 0.53 | 1.21 | 0.52 | 0.19 | -0.63 | -0.32 | -1.11 | 0.90 | -0.35 | -1.70 | 1.17 | 1.33 |
| O14662 | Syntaxin-16 | STX16 | 0.53 | 0.64 | -0.66 | 1.11 | -1.29 | 0.42 | -0.38 | 0.69 | 0.91 | -1.44 | -0.62 | 1.26 |
| Q9NV70 | Exocyst complex component 1 | EXOC1 | 0.53 | 1.22 | -1.93 | -0.17 | -1.21 | 1.00 | 0.95 | 0.34 | -0.25 | -0.42 | 1.06 | 0.64 |
| P08236 | Beta-glucuronidase | GUSB | 0.53 | 0.71 | -0.31 | -0.60 | 1.13 | -0.58 | -0.52 | -0.60 | -0.59 | 2.37 | -0.53 | 0.23 |
| Q7LBC6 | Lysine-specific demethylase 3B | KDM3B | 0.53 | 0.56 | 1.14 | -0.34 | -1.99 | 0.06 | 0.42 | 1.40 | 0.22 | -1.17 | 0.27 | -0.01 |
| Q9P2Q2 | FERM domain-containing protein 4A | FRMD4A | 0.53 | 3.14 | 0.10 | -0.03 | -1.10 | 0.40 | -1.91 | -0.02 | 1.44 | 1.36 | -0.03 | -0.22 |
| Q9UP95 | Solute carrier family 12 member 4 | SLC12A4 | 0.53 | 1.18 | -0.30 | -0.51 | -0.68 | 0.55 | -0.40 | -0.31 | -0.36 | -0.93 | 0.39 | 2.55 |
| Q5SSJ5 | Heterochromatin protein 1-binding protein 3 | HP1BP3 | 0.53 | 1.13 | -0.31 | -1.32 | 0.84 | 0.58 | -1.04 | -0.29 | -1.16 | 1.70 | 0.83 | 0.17 |
| O43896 | Kinesin-like protein KIF1C | KIF1C | 0.53 | 0.91 | 0.45 | 0.02 | -0.71 | 0.79 | -1.61 | 1.06 | 1.24 | -1.52 | 0.29 | 0.00 |
| O15382 | Branched-chain-amino-acid aminotransferase, mitochondrial | BCAT2 | 0.53 | 0.97 | -0.01 | -0.21 | -0.43 | -0.26 | -0.26 | -0.23 | -0.21 | -0.84 | -0.33 | 2.78 |
| Q96FV2 | Secernin-2 | SCRN2 | 0.53 | 1.02 | -1.49 | 0.21 | 0.18 | 0.12 | -0.19 | 1.41 | -1.83 | 0.13 | 0.40 | 1.07 |
| Q9Y266 | Nuclear migration protein nudC | NUDC | 0.53 | 1.07 | 1.17 | 0.38 | -0.69 | -0.13 | -1.95 | 1.29 | 0.62 | -0.94 | -0.21 | 0.46 |
| Q6P589 | Tumor necrosis factor alpha-induced protein 8-like protein 2 | TNFAIP8L2 | 0.53 | 1.13 | -0.42 | -0.58 | -0.84 | 1.18 | -0.60 | -0.67 | -0.64 | 1.60 | -0.54 | 1.53 |
| P53367 | Arfaptin-1 | ARFIP1 | 0.53 | 0.57 | 1.56 | -0.53 | -0.69 | -0.49 | -0.57 | 1.59 | -0.49 | -0.93 | -0.58 | 1.14 |
| Q92794 | Histone acetyltransferase KAT6A | KAT6A | 0.53 | 0.51 | 1.28 | -0.90 | -1.06 | -0.90 | 0.93 | 0.58 | 0.75 | 1.12 | -0.94 | -0.85 |
| P62910 | 60S ribosomal protein L32 | RPL32 | 0.53 | 0.72 | 1.01 | -1.63 | -0.92 | -0.75 | 1.41 | 0.71 | 0.96 | -0.06 | -0.72 | 0.01 |
| Q9Y2H6 | Fibronectin type-III domain-containing protein 3A | FNDC3A | 0.53 | 0.55 | 0.76 | 0.31 | -1.74 | 0.18 | -0.22 | 0.31 | 0.93 | -1.84 | 0.58 | 0.72 |
| P45381 | Aspartoacylase | ASPA | 0.53 | 0.47 | 0.47 | -1.65 | 0.82 | 0.72 | -0.97 | -0.06 | -0.11 | 1.26 | 0.82 | -1.29 |
| Q9BRF8 | Serine/threonine-protein phosphatase CPPED1 | CPPED1 | 0.53 | 0.41 | 1.18 | -1.12 | 0.33 | 0.15 | -1.08 | -1.09 | -0.85 | 1.69 | 0.41 | 0.38 |
| Q9P287 | BRCA2 and CDKN1A-interacting protein | BCCIP | 0.53 | 0.59 | 0.55 | -0.77 | -1.04 | 1.30 | -0.80 | 1.34 | -0.89 | -1.07 | 0.53 | 0.83 |
| O60942 | mRNA-capping enzyme | RNGTT | 0.53 | 0.52 | 1.43 | -1.21 | 0.51 | -0.13 | -1.28 | 0.97 | 1.20 | -0.50 | -0.96 | -0.04 |
| O00560 | Syntenin-1 | SDCBP | 0.52 | 2.48 | -1.92 | -0.10 | 0.66 | -0.63 | -0.21 | -0.24 | 1.96 | 0.68 | 0.00 | -0.20 |
| Q96AC1 | Fermitin family homolog 2 | FERMT2 | 0.52 | 2.57 | 1.22 | -1.66 | -0.68 | -0.34 | -0.79 | 1.78 | 0.19 | 0.50 | 0.10 | -0.31 |
| Q15759 | Mitogen-activated protein kinase 11 | MAPK11 | 0.52 | 0.42 | 0.70 | -0.04 | -0.85 | 0.10 | -0.47 | 1.20 | 1.69 | -1.61 | 0.09 | -0.81 |
| P17655 | Calpain-2 catalytic subunit | CAPN2 | 0.52 | 0.99 | 0.99 | -0.98 | -0.11 | -0.07 | -0.97 | 1.73 | -1.61 | 0.61 | 0.10 | 0.32 |
| Q9HCJ3 | Ribonucleoprotein PTB-binding 2 | RAVER2 | 0.52 | 0.53 | 0.80 | 0.63 | -0.24 | -0.90 | -0.97 | 0.05 | -0.87 | -1.25 | 1.21 | 1.55 |
| P11310 | Medium-chain specific acyl-CoA dehydrogenase, mitochondrial | ACADM | 0.52 | 0.50 | 0.68 | -1.70 | 0.98 | -0.62 | 0.02 | 0.14 | -0.55 | 1.88 | -0.61 | -0.23 |
| P62380 | TATA box-binding protein-like protein 1 | TBPL1 | 0.52 | 0.69 | -0.70 | -0.72 | -1.08 | 0.56 | 1.10 | 0.45 | 0.20 | 1.90 | -0.90 | -0.80 |
| Q9H3P2 | Negative elongation factor A | NELFA | 0.52 | 0.61 | -0.29 | -1.22 | -1.36 | 1.07 | 1.03 | -0.62 | -0.89 | 0.25 | 1.15 | 0.88 |
| Q99959 | Plakophilin-2 | PKP2 | 0.52 | 0.19 | -1.08 | 0.54 | 0.93 | -1.24 | 0.59 | -1.14 | -1.12 | 1.15 | 0.71 | 0.66 |
| P18206 | Vinculin | VCL | 0.52 | 0.61 | 0.63 | -0.13 | -2.21 | -0.34 | 1.27 | 0.50 | 0.40 | 0.81 | -0.95 | 0.02 |
| P28290 | Protein ITPRID2 | ITPRID2 | 0.52 | 1.02 | -0.12 | -0.33 | -0.43 | -0.07 | -0.27 | 2.80 | -0.32 | -0.73 | -0.21 | -0.32 |
| Q04759 | Protein kinase C theta type | PRKCQ | 0.52 | 0.68 | 1.88 | -0.68 | -0.72 | -0.63 | -0.70 | 0.49 | 0.82 | -1.00 | 1.14 | -0.61 |
| O14744 | Protein arginine N-methyltransferase 5 | PRMT5 | 0.52 | 0.44 | -0.05 | -0.56 | 0.83 | -1.18 | 0.37 | 0.51 | -1.89 | 1.56 | -0.03 | 0.44 |
| Q5M775 | Cytospin-B | SPECC1 | 0.52 | 0.48 | 0.72 | 0.25 | -1.39 | -0.03 | -0.18 | 0.24 | 2.17 | -1.01 | 0.04 | -0.82 |
| O15034 | RIMS-binding protein 2 | RIMBP2 | 0.52 | 0.66 | -0.88 | 0.93 | -1.17 | 0.14 | 0.15 | 0.75 | 1.63 | 0.61 | -1.08 | -1.08 |
| Q9UKY7 | Protein CDV3 homolog | CDV3 | 0.52 | 0.36 | 0.59 | 0.20 | 0.32 | -1.59 | -0.01 | 1.28 | 1.21 | -0.20 | -1.68 | -0.12 |
| Q96IZ0 | PRKC apoptosis WT1 regulator protein | PAWR | 0.52 | 1.06 | 0.10 | -0.22 | -0.55 | -0.22 | -0.36 | -0.29 | -0.26 | -0.68 | -0.31 | 2.78 |
| P35813 | Protein phosphatase 1A | PPM1A | 0.52 | 1.24 | -2.14 | 0.71 | 0.59 | 0.27 | -0.78 | -0.87 | 1.31 | 0.52 | 0.02 | 0.38 |
| P28070 | Proteasome subunit beta type-4 | PSMB4 | 0.52 | 1.63 | -0.88 | -1.78 | 0.42 | -0.17 | 0.76 | -0.07 | -0.33 | 2.04 | 0.07 | -0.06 |
| Q96GX9 | Methylthioribulose-1-phosphate dehydratase | APIP | 0.51 | 0.75 | -0.67 | 0.40 | -1.46 | 0.51 | 0.29 | 0.41 | -1.67 | 1.68 | 0.14 | 0.37 |
| Q15233 | Non-POU domain-containing octamer-binding protein | NONO | 0.51 | 2.47 | 0.94 | 0.13 | -1.24 | -0.27 | -1.77 | 1.40 | 0.35 | -0.69 | 0.30 | 0.84 |
| Q5VWZ2 | Lysophospholipase-like protein 1 | LYPLAL1 | 0.51 | 0.72 | 0.88 | 0.07 | -1.79 | 0.81 | -0.85 | 1.42 | -0.73 | -0.78 | 0.45 | 0.51 |
| P78406 | mRNA export factor | RAE1 | 0.51 | 0.80 | -0.47 | 0.70 | -0.75 | -0.50 | 0.06 | -0.68 | -0.54 | 2.56 | -0.43 | 0.07 |
| Q4J6C6 | Prolyl endopeptidase-like | PREPL | 0.51 | 1.79 | -0.01 | -1.75 | 0.95 | 0.15 | -1.12 | -0.04 | 1.81 | 0.49 | -0.48 | -0.01 |
| Q8NFW9 | Rab effector MyRIP | MYRIP | 0.51 | 0.55 | 0.04 | 0.68 | -2.01 | -0.06 | 0.66 | -0.27 | 1.17 | 1.15 | -0.25 | -1.10 |
| Q5H9L2 | Transcription elongation factor A protein-like 5 | TCEAL5 | 0.51 | 1.01 | -0.10 | -0.30 | -0.42 | -0.23 | -0.16 | -0.13 | -1.00 | 2.75 | -0.21 | -0.19 |
| P60228 | Eukaryotic translation initiation factor 3 subunit E | EIF3E | 0.51 | 1.53 | 0.29 | -0.27 | -0.36 | -0.28 | -1.02 | 1.69 | 0.94 | -1.70 | -0.24 | 0.95 |
| P14621 | Acylphosphatase-2 | ACYP2 | 0.51 | 1.16 | -1.06 | -1.22 | 0.38 | 0.14 | 0.46 | 0.22 | -1.74 | 0.99 | 0.51 | 1.31 |
| Q96T49 | Protein phosphatase 1 regulatory inhibitor subunit 16B | PPP1R16B | 0.50 | 0.53 | -0.47 | 0.70 | -1.59 | 0.73 | -0.05 | -1.87 | 0.53 | 0.68 | 0.89 | 0.45 |

| | | | | | | | | | | | | | | |
|---|---|---|---|---|---|---|---|---|---|---|---|---|---|---|
| O75508 | Claudin-11 | CLDN11 | 0.50 | 0.58 | 1.40 | -0.21 | -0.41 | -0.82 | -0.70 | 1.55 | 1.29 | -0.47 | -1.02 | -0.61 |
| P22392 | Nucleoside diphosphate kinase B | NME2 | 0.50 | 1.29 | -0.56 | 0.81 | -1.52 | -0.80 | 0.66 | 0.94 | -0.36 | 1.68 | -0.91 | 0.05 |
| Q01469 | Fatty acid-binding protein 5 | FABP5 | 0.50 | 0.73 | 0.50 | -0.59 | -0.38 | -0.61 | 0.17 | 0.57 | -1.29 | 2.34 | -0.10 | -0.60 |
| Q9BS26 | Endoplasmic reticulum resident protein 44 | ERP44 | 0.50 | 1.04 | -0.32 | 0.89 | -0.71 | -0.51 | -0.55 | 1.73 | -0.46 | -0.98 | -0.64 | 1.56 |
| O60547 | GDP-mannose 4,6 dehydratase | GMDS | 0.50 | 0.80 | 0.04 | 0.36 | 0.44 | 0.17 | -1.97 | -1.47 | -0.15 | 1.32 | 0.80 | 0.45 |
| Q14185 | Dedicator of cytokinesis protein 1 | DOCK1 | 0.50 | 0.79 | -0.84 | -0.69 | 1.44 | -0.38 | -0.48 | 1.00 | 0.11 | -1.68 | 1.21 | 0.31 |
| Q9Y3S1 | Serine/threonine-protein kinase WNK2 | WNK2 | 0.50 | 0.67 | -2.14 | 0.07 | -0.37 | 1.01 | 0.59 | -1.14 | 0.39 | 1.02 | 0.65 | -0.08 |
| Q9H1K1 | Iron-sulfur cluster assembly enzyme ISCU, mitochondrial | ISCU | 0.50 | 1.45 | 0.45 | -0.31 | -2.41 | 0.03 | 0.70 | -0.69 | 0.95 | 0.78 | -0.13 | 0.63 |
| Q14576 | ELAV-like protein 3 | ELAVL3 | 0.50 | 1.85 | -0.32 | -1.87 | -1.24 | 1.01 | 0.59 | 0.05 | 1.08 | -0.45 | 1.04 | 0.11 |
| P25685 | DnaJ homolog subfamily B member 1 | DNAJB1 | 0.50 | 0.79 | -0.87 | 1.18 | -0.63 | -0.15 | -0.49 | -0.18 | -0.07 | -1.53 | 1.19 | 1.55 |
| P04271 | Protein S100-B | S100B | 0.50 | 0.17 | -1.13 | 0.42 | 0.90 | -1.17 | 0.73 | -1.16 | -1.14 | 0.73 | 0.92 | 0.89 |
| Q9H0C8 | Integrin-linked kinase-associated serine/threonine phosphatase 2C | ILKAP | 0.50 | 0.59 | 0.17 | 0.11 | -1.77 | 0.75 | -0.01 | 0.88 | 0.36 | -1.86 | 0.74 | 0.63 |
| Q96TC7 | Regulator of microtubule dynamics protein 3 | RMDN3 | 0.50 | 0.84 | -1.41 | 1.16 | 0.59 | -0.65 | -0.68 | 1.51 | -0.63 | -1.04 | 0.58 | 0.57 |
| O75822 | Eukaryotic translation initiation factor 3 subunit J | EIF3J | 0.50 | 2.03 | -2.28 | -0.26 | 0.13 | 0.24 | 0.24 | 1.17 | 0.48 | 0.12 | 1.07 | -0.92 |
| O00442 | RNA 3'-terminal phosphate cyclase | RTCA | 0.50 | 0.32 | -1.28 | 0.41 | 1.00 | -1.03 | 0.45 | -1.09 | -1.06 | 1.38 | 0.63 | 0.58 |
| Q96KR1 | Zinc finger RNA-binding protein | ZFR | 0.50 | 1.49 | 0.19 | -2.00 | -0.92 | 1.31 | -0.16 | 0.23 | -0.73 | 0.42 | 1.19 | 0.47 |
| P63220 | 40S ribosomal protein S21 | RPS21 | 0.50 | 2.21 | -0.08 | 0.32 | -0.72 | -0.91 | -0.71 | -1.49 | 1.42 | 1.20 | -0.19 | 1.15 |
| O95622 | Adenylate cyclase type 5 | ADCY5 | 0.49 | 1.59 | -0.75 | 1.22 | -2.17 | -0.30 | 0.35 | 0.12 | 1.01 | 0.23 | -0.51 | 0.82 |
| P27348 | 14-3-3 protein theta | YWHAQ | 0.49 | 2.03 | 0.40 | 0.39 | -0.35 | -0.64 | -1.73 | 1.41 | 1.29 | -0.89 | -0.53 | 0.64 |
| Q9ULE6 | Paladin | PALD1 | 0.49 | 0.93 | 0.35 | -2.75 | 0.35 | 0.09 | 0.84 | 0.04 | 0.00 | 0.14 | 0.53 | 0.42 |
| Q9UBF8 | Phosphatidylinositol 4-kinase beta | PI4KB | 0.49 | 1.71 | 0.09 | -0.49 | -0.93 | -0.17 | -0.29 | -0.16 | -0.20 | -1.29 | 1.43 | 2.00 |
| Q96PU4 | E3 ubiquitin-protein ligase UHRF2 | UHRF2 | 0.49 | 0.78 | 0.87 | 0.18 | -1.01 | -0.90 | -0.08 | -0.71 | 1.87 | -0.22 | 1.08 | -1.07 |
| Q8NC51 | Plasminogen activator inhibitor 1 RNA-binding protein | SERBP1 | 0.49 | 0.48 | 0.59 | 0.72 | 0.51 | -2.17 | -0.28 | 0.43 | 1.01 | -0.27 | -1.17 | 0.64 |
| A0MZ66 | Shootin-1 | SHTN1 | 0.49 | 0.67 | 1.40 | -0.08 | -0.64 | -0.82 | -0.69 | 2.00 | 0.38 | -0.44 | -0.04 | -1.07 |
| Q93074 | Mediator of RNA polymerase II transcription subunit 12 | MED12 | 0.49 | 0.71 | 1.10 | 0.52 | -1.34 | 0.02 | -1.17 | 1.12 | -1.12 | 0.65 | 0.94 | -0.72 |
| P26012 | Integrin beta-8 | ITGB8 | 0.49 | 0.72 | -1.19 | -0.05 | -1.16 | 0.91 | 0.61 | -1.21 | 0.40 | -0.68 | 0.94 | 1.43 |
| P16219 | Short-chain specific acyl-CoA dehydrogenase, mitochondrial | ACADS | 0.49 | 0.47 | -1.65 | -0.33 | 0.15 | 0.64 | 0.57 | -0.12 | -1.78 | 1.00 | 0.94 | 0.58 |
| Q14258 | E3 ubiquitin/ISG15 ligase TRIM25 | TRIM25 | 0.49 | 0.98 | 0.51 | 0.51 | -2.46 | 0.00 | 0.31 | 1.01 | 0.75 | -0.70 | -0.30 | 0.39 |
| P46108 | Adapter molecule crk | CRK | 0.49 | 4.97 | -1.39 | -0.79 | -1.07 | 0.15 | -0.15 | 1.20 | -0.18 | 1.67 | -0.38 | 0.93 |
| P08237 | ATP-dependent 6-phosphofructokinase, muscle type | PFKM | 0.48 | 3.33 | -1.01 | -1.62 | 0.63 | 0.30 | -0.91 | -0.02 | 0.16 | 1.93 | 0.50 | 0.04 |
| P07998 | Ribonuclease pancreatic | RNASE1 | 0.48 | 0.40 | -0.15 | -0.54 | 0.69 | 0.65 | -1.19 | -1.17 | -1.10 | 1.74 | 0.86 | 0.22 |
| Q14195 | Dihydropyrimidinase-related protein 3 | DPYSL3 | 0.48 | 2.30 | -0.61 | -0.86 | 0.46 | -0.87 | -0.27 | 1.58 | -1.05 | 1.78 | -0.21 | 0.05 |
| P50895 | Basal cell adhesion molecule | BCAM | 0.48 | 0.78 | 1.41 | -0.53 | -0.61 | -0.81 | -0.41 | 1.45 | -0.99 | 1.15 | -1.01 | 0.35 |
| Q02790 | Peptidyl-prolyl cis-trans isomerase FKBP4 | FKBP4 | 0.48 | 1.15 | -2.01 | 1.19 | 0.25 | 0.22 | -0.94 | 1.08 | -0.17 | -0.70 | 0.16 | 0.93 |
| Q8IXF0 | Neuronal PAS domain-containing protein 3 | NPAS3 | 0.48 | 1.10 | -0.04 | -0.26 | -0.46 | -0.09 | -0.44 | -0.07 | 2.78 | -0.75 | -0.37 | -0.29 |
| Q59EK9 | RUN domain-containing protein 3A | RUNDC3A | 0.48 | 0.60 | -0.15 | 1.01 | -2.45 | 0.36 | 0.46 | -0.85 | 0.11 | 0.69 | 0.23 | 0.59 |
| P61966 | AP-1 complex subunit sigma-1A | AP1S1 | 0.48 | 0.48 | -0.10 | 0.46 | -0.97 | -0.93 | 0.91 | 0.43 | 1.82 | -1.24 | 0.52 | -0.91 |
| Q9C026 | E3 ubiquitin-protein ligase TRIM9 | TRIM9 | 0.48 | 0.66 | -1.45 | -1.40 | 0.08 | 0.48 | 1.46 | -0.77 | -0.60 | 0.74 | 0.71 | 0.75 |
| P11182 | Lipoamide acyltransferase component of branched-chain alpha-ke | DBT | 0.48 | 0.68 | 0.61 | -1.49 | 1.26 | -1.53 | 0.30 | -0.28 | -0.06 | -0.60 | 0.49 | 1.31 |
| Q9BXJ9 | N-alpha-acetyltransferase 15, NatA auxiliary subunit | NAA15 | 0.48 | 0.56 | -2.32 | 0.26 | 0.81 | 0.48 | 0.06 | -1.20 | 0.00 | 0.63 | 0.58 | 0.70 |
| O75592 | E3 ubiquitin-protein ligase MYCBP2 | MYCBP2 | 0.48 | 0.78 | 1.13 | -0.86 | -1.30 | 0.19 | -0.10 | 0.94 | 1.14 | -1.53 | -0.36 | 0.76 |
| P15880 | 40S ribosomal protein S2 | RPS2 | 0.48 | 1.74 | -0.54 | -1.45 | 0.84 | -0.10 | -0.49 | -0.11 | -0.36 | 2.30 | -0.36 | 0.26 |
| P30041 | Peroxiredoxin-6 | PRDX6 | 0.48 | 1.64 | -0.57 | -1.11 | -0.74 | 0.55 | 0.18 | 1.10 | -1.84 | 0.76 | 0.87 | 0.79 |
| O75874 | Isocitrate dehydrogenase [NADP] cytoplasmic | IDH1 | 0.48 | 1.57 | -0.32 | 1.06 | -1.61 | -0.24 | -0.50 | -0.72 | -0.05 | 2.05 | 0.37 | -0.03 |
| O00161 | Synaptosomal-associated protein 23 | SNAP23 | 0.48 | 0.65 | -0.63 | 0.92 | -1.12 | 0.84 | -0.83 | 0.60 | -0.80 | -1.23 | 0.91 | 1.33 |
| Q9NYB9 | Abl interactor 2 | ABI2 | 0.48 | 0.58 | 0.33 | 0.90 | -2.58 | -0.03 | 0.63 | 0.52 | 0.59 | 0.36 | -0.31 | -0.41 |
| Q9HBF4 | Zinc finger FYVE domain-containing protein 1 | ZFYVE1 | 0.48 | 0.82 | -0.21 | -0.22 | -0.44 | 0.24 | -0.38 | -1.89 | -0.39 | 1.94 | 0.45 | 0.91 |
| P09936 | Ubiquitin carboxyl-terminal hydrolase isozyme L1 | UCHL1 | 0.48 | 1.92 | -0.54 | 0.33 | 0.08 | -0.27 | -1.48 | 0.18 | 2.37 | 0.23 | -0.11 | -0.79 |
| Q9Y5U9 | Immediate early response 3-interacting protein 1 | IER3IP1 | 0.48 | 0.98 | -0.09 | -0.32 | -0.37 | -0.09 | -0.33 | -0.30 | -0.34 | -0.66 | -0.33 | 2.81 |
| Q9UQ80 | Proliferation-associated protein 2G4 | PA2G4 | 0.48 | 1.58 | 0.13 | -0.18 | 0.39 | 0.47 | -2.47 | 0.72 | 0.73 | -0.78 | 0.07 | 0.91 |
| P07195 | L-lactate dehydrogenase B chain | LDHB | 0.47 | 1.12 | -0.52 | -0.74 | 0.32 | 0.03 | -0.39 | -0.42 | -0.53 | 2.61 | 0.37 | -0.73 |
| P07384 | Calpain-1 catalytic subunit | CAPN1 | 0.47 | 1.36 | 1.85 | -1.08 | -0.53 | -0.28 | -1.44 | 0.02 | -0.14 | 1.36 | -0.08 | 0.32 |
| Q92575 | UBX domain-containing protein 4 | UBXN4 | 0.47 | 1.05 | 0.12 | -0.36 | -0.45 | -0.30 | -0.25 | -0.30 | -0.17 | -0.88 | -0.16 | 2.75 |
| P07305 | Histone H1.0 | H1F0 | 0.47 | 1.43 | 0.71 | -0.91 | 0.89 | -0.03 | -2.21 | 0.08 | -0.46 | 0.34 | 0.34 | 1.24 |
| Q5HYK7 | SH3 domain-containing protein 19 | SH3D19 | 0.47 | 0.34 | 1.56 | -0.03 | -1.02 | -0.89 | -0.09 | 1.28 | 1.05 | -1.10 | -0.87 | 0.10 |
| P63165 | Small ubiquitin-related modifier 1 | SUMO1 | 0.47 | 0.44 | -0.82 | -0.92 | -1.03 | 0.63 | 1.56 | -0.92 | -0.90 | 0.67 | 0.91 | 0.80 |
| P02749 | Beta-2-glycoprotein 1 | APOH | 0.47 | 1.60 | -1.32 | -1.63 | 1.35 | -0.55 | 0.48 | 0.03 | 0.22 | 0.47 | -0.40 | 1.34 |
| P54829 | Tyrosine-protein phosphatase non-receptor type 5 | PTPN5 | 0.47 | 1.44 | -0.34 | -0.33 | -0.73 | 0.55 | -0.70 | 1.75 | 1.22 | -0.07 | 0.32 | -1.67 |
| Q15121 | Astrocytic phosphoprotein PEA-15 | PEA15 | 0.47 | 1.17 | 0.50 | 0.63 | -0.54 | -1.34 | -0.57 | 1.39 | 1.04 | -0.98 | -1.06 | 0.91 |
| P49792 | E3 SUMO-protein ligase RanBP2 | RANBP2 | 0.47 | 0.61 | -0.04 | 1.12 | -0.39 | -1.58 | 0.13 | -0.26 | 1.83 | -1.15 | 0.54 | -0.20 |
| Q9H7E2 | Tudor domain-containing protein 3 | TDRD3 | 0.47 | 0.66 | 0.84 | 0.16 | -0.22 | -0.26 | -1.35 | 1.33 | 0.69 | 0.43 | 0.32 | -1.95 |
| Q9NQ88 | Fructose-2,6-bisphosphatase TIGAR | TIGAR | 0.47 | 1.11 | -1.34 | 0.15 | 0.16 | 0.82 | -1.03 | 0.90 | -1.25 | -0.62 | 0.79 | 1.44 |
| Q07352 | mRNA decay activator protein ZFP36L1 | ZFP36L1 | 0.47 | 0.73 | -0.96 | 0.29 | -1.38 | -0.26 | 1.41 | 1.28 | -0.23 | 1.14 | -0.32 | -0.98 |
| Q9NVH1 | DnaJ homolog subfamily C member 11 | DNAJC11 | 0.47 | 0.62 | 0.76 | 1.31 | -1.01 | -0.85 | -0.99 | 0.66 | -0.79 | 0.25 | -0.82 | 1.48 |
| Q8IY17 | Neuropathy target esterase | PNPLA6 | 0.47 | 1.60 | 1.09 | -1.66 | -0.03 | 0.46 | -1.51 | -0.42 | 0.21 | -0.26 | 1.09 | 1.04 |
| Q96G46 | tRNA-dihydrouridine(47) synthase [NAD(P)(+)]-like | DUS3L | 0.47 | 0.73 | 0.79 | -0.69 | -0.96 | -0.75 | 0.71 | -0.68 | 1.41 | 1.57 | -0.73 | -0.67 |
| P51148 | Ras-related protein Rab-5C | RAB5C | 0.47 | 3.54 | 0.30 | -1.75 | 0.28 | 0.11 | -1.72 | -0.21 | 0.94 | 0.16 | 1.16 | 0.73 |
| P35711 | Transcription factor SOX-5 | SOX5 | 0.46 | 0.41 | -0.77 | 1.01 | -0.98 | 1.14 | -0.95 | 0.75 | -0.80 | -1.16 | 1.17 | 0.58 |
| Q70E73 | Ras-associated and pleckstrin homology domains-containing prote | RAPH1 | 0.46 | 0.55 | 0.37 | 0.44 | -2.68 | 0.55 | 0.60 | 0.01 | 0.00 | -0.40 | 0.49 | 0.63 |
| Q14019 | Coactosin-like protein | COTL1 | 0.46 | 1.84 | -0.39 | -0.80 | 0.75 | 0.62 | -1.99 | 0.67 | -1.07 | 0.78 | 0.95 | 0.47 |
| Q66K74 | Microtubule-associated protein 1S | MAP1S | 0.46 | 1.37 | -0.25 | -0.50 | -1.41 | -0.72 | 1.42 | -0.05 | 0.05 | 0.79 | -0.95 | 1.63 |
| Q9P2R6 | Arginine-glutamic acid dipeptide repeats protein | RERE | 0.46 | 2.68 | -0.81 | 0.44 | -0.05 | -2.39 | 0.43 | 0.82 | 0.72 | -0.42 | 0.85 | 0.41 |
| Q99543 | DnaJ homolog subfamily C member 2 | DNAJC2 | 0.46 | 0.38 | 0.70 | -0.83 | -1.28 | -0.26 | 1.16 | 0.75 | 1.62 | -0.14 | -1.05 | -0.68 |
| Q9Y224 | RNA transcription, translation and transport factor protein | RTRAF | 0.46 | 0.61 | -0.90 | -0.28 | -0.86 | -0.39 | 1.67 | 0.85 | -1.06 | -0.09 | -0.47 | 1.53 |
| P19404 | NADH dehydrogenase [ubiquinone] flavoprotein 2, mitochondrial | NDUFV2 | 0.46 | 0.38 | -0.91 | 0.61 | -0.21 | 1.11 | -1.11 | -1.08 | -1.06 | 1.07 | 1.34 | 0.23 |
| P61077 | Ubiquitin-conjugating enzyme E2 D3 | UBE2D3 | 0.46 | 0.96 | -0.28 | -0.10 | -2.47 | 0.92 | 0.79 | -0.06 | -0.35 | 0.57 | 0.96 | 0.02 |
| P52948 | Nuclear pore complex protein Nup98-Nup96 | NUP98 | 0.46 | 0.66 | -0.35 | -0.60 | -0.70 | 1.38 | -0.55 | -0.61 | -0.51 | -0.95 | 1.65 | 1.25 |
| O60674 | Tyrosine-protein kinase JAK2 | JAK2 | 0.46 | 0.59 | -0.30 | -0.46 | -0.81 | 1.47 | -0.64 | -0.47 | -0.62 | -0.96 | 1.52 | 1.28 |
| O94819 | Kelch repeat and BTB domain-containing protein 11 | KBTBD11 | 0.46 | 1.87 | -1.33 | -0.26 | 0.35 | 1.15 | -1.76 | -0.46 | 0.44 | 0.87 | 1.19 | -0.19 |
| P68871 | Hemoglobin subunit beta | HBB | 0.46 | 1.28 | -0.10 | -0.94 | -0.56 | -0.09 | 0.26 | -1.47 | 0.53 | 2.28 | 0.26 | -0.17 |
| P17844 | Probable ATP-dependent RNA helicase DDX5 | DDX5 | 0.46 | 0.66 | 1.43 | -1.64 | -1.21 | -0.30 | 0.89 | 0.86 | 0.53 | -0.90 | 0.20 | 0.15 |
| P40925 | Malate dehydrogenase, cytoplasmic | MDH1 | 0.46 | 0.55 | -0.19 | -0.10 | -0.01 | -0.38 | -0.05 | -0.02 | -1.21 | 2.66 | -0.22 | -0.47 |
| Q9Y6F6 | Protein MRVI1 | MRVI1 | 0.45 | 0.50 | 1.33 | 0.41 | -0.93 | -0.53 | -0.92 | 1.27 | 1.23 | -1.10 | -0.84 | 0.08 |

| | | | | | | | | | | | | | | |
|---|---|---|---|---|---|---|---|---|---|---|---|---|---|---|
| O95571 | Persulfide dioxygenase ETHE1, mitochondrial | ETHE1 | 0.45 | 0.49 | -0.76 | 0.90 | 1.23 | -0.95 | -1.06 | -0.98 | -0.92 | 1.05 | 0.67 | 0.82 |
| Q6IN85 | Serine/threonine-protein phosphatase 4 regulatory subunit 3A | PPP4R3A | 0.45 | 0.56 | 1.46 | -1.04 | -1.21 | -0.15 | 0.22 | -0.36 | 0.88 | -1.37 | 0.41 | 1.14 |
| Q8N1G2 | Cap-specific mRNA (nucleoside-2'-O-)-methyltransferase 1 | CMTR1 | 0.45 | 0.56 | 1.44 | -1.08 | -1.23 | 1.29 | -1.16 | 0.60 | -0.27 | 0.03 | 0.80 | -0.43 |
| Q9UPU9 | Protein Smaug homolog 1 | SAMD4A | 0.45 | 0.65 | 0.60 | -0.25 | -0.54 | -0.28 | -0.35 | 2.66 | -0.37 | -0.82 | -0.35 | -0.30 |
| P49321 | Nuclear autoantigenic sperm protein | NASP | 0.45 | 0.50 | -0.47 | -0.62 | -0.80 | 1.85 | -0.61 | -0.61 | -0.50 | 1.27 | -0.66 | 1.15 |
| P30519 | Heme oxygenase 2 | HMOX2 | 0.45 | 0.61 | 0.24 | 1.10 | 0.12 | 0.19 | -2.44 | 0.66 | 0.69 | -0.89 | 0.17 | 0.15 |
| Q9BQ90 | Kelch domain-containing protein 3 | KLHDC3 | 0.45 | 0.47 | 1.10 | -0.79 | -0.65 | -0.33 | 0.06 | 1.28 | 1.73 | -0.98 | -0.75 | -0.67 |
| P49757 | Protein numb homolog | NUMB | 0.45 | 0.69 | -0.51 | 1.27 | -1.06 | 0.25 | -0.81 | -0.82 | 0.69 | -1.14 | 0.53 | 1.60 |
| Q9BWU0 | Kanadaptin | SLC4A1AP | 0.45 | 1.48 | 0.59 | 0.68 | -1.63 | -0.29 | -0.90 | 0.11 | 0.67 | -0.90 | -0.17 | 1.83 |
| Q8WVV9 | Heterogeneous nuclear ribonucleoprotein L-like | HNRNPLL | 0.45 | 0.91 | 1.26 | -2.01 | -0.95 | 0.25 | 0.37 | 0.78 | 1.00 | -0.35 | 0.32 | -0.67 |
| P61221 | ATP-binding cassette sub-family E member 1 | ABCE1 | 0.45 | 1.80 | -0.52 | 0.20 | -1.68 | 0.16 | 0.04 | 0.33 | 0.52 | 1.87 | 0.40 | -1.34 |
| P62328 | Thymosin beta-4 | TMSB4X | 0.45 | 0.91 | 0.12 | -1.03 | -1.20 | 0.10 | 0.94 | -0.99 | -0.89 | 1.75 | 0.45 | 0.75 |
| Q9NR45 | Sialic acid synthase | NANS | 0.45 | 1.49 | -1.82 | 1.56 | -0.14 | -0.36 | -0.82 | 1.12 | -0.02 | -0.67 | 0.37 | 0.78 |
| Q9UPR0 | Inactive phospholipase C-like protein 2 | PLCL2 | 0.45 | 0.79 | 1.18 | -1.40 | 0.20 | -0.45 | -0.49 | 1.87 | -0.18 | 0.59 | -0.20 | -1.12 |
| Q96HU1 | Small G protein signaling modulator 3 | SGSM3 | 0.45 | 0.55 | 1.20 | -0.96 | -1.13 | -0.90 | 1.08 | 0.58 | -0.04 | -1.21 | 1.26 | 0.12 |
| Q9UPY8 | Microtubule-associated protein RP/EB family member 3 | MAPRE3 | 0.45 | 0.70 | -0.19 | 0.43 | -0.33 | -0.30 | -0.50 | -0.55 | 2.65 | -0.94 | -0.33 | 0.07 |
| P55209 | Nucleosome assembly protein 1-like 1 | NAP1L1 | 0.45 | 1.51 | 0.07 | -0.31 | -1.28 | 0.50 | -0.59 | 1.63 | 0.70 | -1.73 | 0.66 | 0.33 |
| Q9BYG3 | MKI67 FHA domain-interacting nucleolar phosphoprotein | NIFK | 0.45 | 1.04 | 0.20 | -0.13 | -0.70 | -0.14 | -0.46 | -0.14 | -0.24 | -0.81 | -0.30 | 2.72 |
| Q92743 | Serine protease HTRA1 | HTRA1 | 0.44 | 0.53 | 1.46 | -0.14 | -0.64 | -1.04 | -0.32 | 1.77 | -0.67 | -0.21 | -0.99 | 0.79 |
| Q14624 | Inter-alpha-trypsin inhibitor heavy chain H4 | ITIH4 | 0.44 | 1.53 | -1.74 | -0.30 | -0.81 | 1.84 | -0.62 | -0.03 | 0.26 | 0.02 | 1.14 | 0.26 |
| P24821 | Tenascin | TNC | 0.44 | 0.50 | -0.69 | -1.36 | 0.33 | 0.91 | 0.16 | 0.22 | -1.63 | 1.17 | 1.23 | -0.34 |
| Q9UKU0 | Long-chain-fatty-acid--CoA ligase 6 | ACSL6 | 0.44 | 0.83 | -0.24 | 0.81 | -0.50 | 0.16 | -1.23 | 0.72 | 0.68 | -1.97 | 0.33 | 1.24 |
| P40939 | Trifunctional enzyme subunit alpha, mitochondrial | HADHA | 0.44 | 1.83 | -0.10 | -2.30 | 0.33 | 0.92 | -0.69 | 0.59 | 0.01 | -0.03 | 1.39 | -0.13 |
| P30048 | Thioredoxin-dependent peroxide reductase, mitochondrial | PRDX3 | 0.44 | 0.34 | -1.77 | 0.03 | 0.54 | 0.31 | 0.43 | -1.85 | 0.17 | 1.17 | 0.48 | 0.49 |
| Q9NRX4 | 14 kDa phosphohistidine phosphatase | PHPT1 | 0.44 | 0.95 | 0.81 | 0.28 | -0.81 | 0.38 | -1.75 | 1.20 | 0.25 | -1.49 | 0.50 | 0.64 |
| Q15853 | Upstream stimulatory factor 2 | USF2 | 0.44 | 0.65 | 0.70 | -0.80 | -1.29 | -0.60 | 1.18 | -0.05 | -1.09 | -0.38 | 0.72 | 1.61 |
| Q9UJ70 | N-acetyl-D-glucosamine kinase | NAGK | 0.44 | 2.38 | -0.03 | -1.84 | 0.50 | 0.30 | -1.06 | 0.95 | -1.10 | 0.66 | 0.43 | 1.20 |
| P51784 | Ubiquitin carboxyl-terminal hydrolase 11 | USP11 | 0.44 | 0.30 | 0.32 | -0.72 | -0.21 | -0.33 | 0.53 | 0.64 | -0.97 | 0.49 | -1.65 | 1.91 |
| P23193 | Transcription elongation factor A protein 1 | TCEA1 | 0.44 | 0.67 | -0.95 | -0.16 | -1.33 | 1.04 | 0.57 | -1.03 | -1.00 | 0.78 | 0.89 | 1.20 |
| Q8N2G6 | Zinc finger CCHC domain-containing protein 24 | ZCCHC24 | 0.44 | 2.23 | -0.12 | -0.22 | -0.86 | -0.18 | -0.78 | -0.56 | -0.20 | 0.74 | 2.55 | -0.38 |
| Q8IYB7 | DIS3-like exonuclease 2 | DIS3L2 | 0.44 | 1.27 | -1.81 | 1.05 | -0.35 | 0.32 | -0.59 | 1.77 | -0.05 | -0.85 | 0.09 | 0.42 |
| Q9H2G4 | Testis-specific Y-encoded-like protein 2 | TSPYL2 | 0.44 | 0.96 | -0.23 | -0.70 | 1.24 | -0.64 | -0.79 | -0.60 | -0.71 | 1.90 | 1.07 | -0.54 |
| P07339 | Cathepsin D | CTSD | 0.44 | 0.72 | -0.44 | -0.57 | -0.73 | -1.02 | 1.88 | -0.84 | 0.92 | 1.06 | -0.71 | 0.45 |
| O75150 | E3 ubiquitin-protein ligase BRE1B | RNF40 | 0.44 | 0.31 | 1.07 | -1.02 | 0.23 | -0.81 | 0.10 | 2.14 | -0.22 | 0.15 | -1.03 | -0.61 |
| P23434 | Glycine cleavage system H protein, mitochondrial | GCSH | 0.44 | 0.38 | -0.35 | -0.44 | 1.23 | -0.44 | -0.51 | -0.45 | -0.53 | 2.41 | -0.49 | -0.43 |
| Q15847 | Adipogenesis regulatory factor | ADIRF | 0.44 | 0.32 | -0.46 | -0.58 | -0.67 | -0.56 | 1.84 | -0.57 | 1.82 | 0.29 | -0.54 | -0.56 |
| Q96C24 | Synaptotagmin-like protein 4 | SYTL4 | 0.44 | 0.37 | 0.11 | 0.81 | -1.27 | 0.58 | -0.73 | 1.23 | -1.05 | -1.35 | 0.83 | 0.84 |
| O75964 | ATP synthase subunit g, mitochondrial | ATP5MG | 0.44 | 0.63 | 1.00 | -0.84 | 0.72 | -0.82 | -0.84 | 1.84 | 0.59 | -1.26 | -0.24 | -0.13 |
| Q13303 | Voltage-gated potassium channel subunit beta-2 | KCNAB2 | 0.43 | 0.91 | 0.97 | 0.23 | 0.01 | -1.10 | -1.18 | -0.18 | 1.05 | 0.88 | -1.62 | 0.95 |
| O14929 | Histone acetyltransferase type B catalytic subunit | HAT1 | 0.43 | 0.94 | 0.09 | -0.18 | -0.56 | -0.06 | -0.42 | -0.26 | -0.29 | -0.88 | -0.16 | 2.74 |
| Q06830 | Peroxiredoxin-1 | PRDX1 | 0.43 | 0.99 | -1.16 | -1.39 | 0.45 | -0.51 | 1.46 | 0.87 | -0.66 | 1.34 | -0.33 | -0.08 |
| Q9NPH2 | Inositol-3-phosphate synthase 1 | ISYNA1 | 0.43 | 0.40 | -0.97 | -0.08 | -0.40 | 0.41 | 0.50 | -1.01 | -1.09 | 2.25 | 0.42 | -0.03 |
| P62244 | 40S ribosomal protein S15a | RPS15A | 0.43 | 1.70 | -0.02 | -1.88 | 0.34 | 0.22 | -0.37 | 0.66 | -1.55 | 1.10 | 0.73 | 0.76 |
| Q6P2M8 | Calcium/calmodulin-dependent protein kinase type 1B | PNCK | 0.43 | 0.41 | 0.61 | -1.69 | 0.26 | 0.12 | 0.16 | 0.39 | 1.56 | 0.09 | 0.24 | -1.73 |
| P02774 | Vitamin D-binding protein | GC | 0.43 | 0.70 | 1.49 | -0.83 | -1.04 | 0.31 | -0.80 | 0.26 | 0.38 | 1.71 | -0.84 | -0.66 |
| Q9Y6D9 | Mitotic spindle assembly checkpoint protein MAD1 | MAD1L1 | 0.43 | 0.70 | 1.12 | 0.16 | -2.19 | -0.14 | 0.18 | -0.30 | -0.21 | 1.63 | 0.02 | -0.28 |
| O43684 | Mitotic checkpoint protein BUB3 | BUB3 | 0.43 | 2.62 | -1.44 | 0.40 | -1.20 | -0.31 | 0.28 | 0.50 | -0.47 | -0.46 | 0.70 | 2.00 |
| P04259 | Keratin, type II cytoskeletal 6B | KRT6B | 0.43 | 0.72 | 0.22 | -1.33 | 0.47 | -1.18 | 0.93 | -0.36 | 0.18 | 1.84 | -1.04 | 0.25 |
| P00441 | Superoxide dismutase [Cu-Zn] | SOD1 | 0.43 | 0.37 | -0.21 | 0.21 | 0.62 | -0.79 | -0.34 | -0.02 | -1.32 | 2.39 | -0.56 | 0.02 |
| Q2TAC6 | Kinesin-like protein KIF19 | KIF19 | 0.43 | 0.40 | 0.05 | -0.67 | -0.83 | 1.51 | -0.59 | -0.53 | -0.66 | -0.96 | 1.40 | 1.28 |
| O14986 | Phosphatidylinositol 4-phosphate 5-kinase type-1 beta | PIP5K1B | 0.43 | 0.73 | 0.70 | 1.00 | -0.94 | -0.76 | -0.89 | 0.84 | 0.26 | -1.09 | -0.76 | 1.64 |
| Q9HAB8 | Phosphopantothenate--cysteine ligase | PPCS | 0.43 | 0.47 | -1.54 | 1.10 | -0.19 | -0.03 | 0.04 | 0.85 | 0.96 | -1.87 | 0.32 | 0.36 |
| P21291 | Cysteine and glycine-rich protein 1 | CSRP1 | 0.43 | 1.20 | -0.26 | -0.11 | -1.95 | 0.59 | 0.41 | 1.09 | -1.25 | -0.37 | 0.83 | 1.02 |
| O43598 | 2'-deoxynucleoside 5'-phosphate N-hydrolase 1 | DNPH1 | 0.43 | 0.40 | -0.72 | -0.86 | -0.98 | 0.91 | 1.12 | -0.82 | -0.24 | 1.51 | 0.95 | -0.88 |
| Q14765 | Signal transducer and activator of transcription 4 | STAT4 | 0.43 | 0.97 | 0.70 | -0.59 | -0.87 | 0.54 | -0.90 | 1.64 | 0.68 | -1.41 | 0.87 | -0.65 |
| Q9Y5S2 | Serine/threonine-protein kinase MRCK beta | CDC42BPB | 0.43 | 0.67 | 0.62 | 0.29 | -1.31 | 0.27 | -0.70 | 1.95 | 0.71 | -1.02 | 0.12 | -0.93 |
| P49407 | Beta-arrestin-1 | ARRB1 | 0.43 | 1.20 | -1.30 | -1.12 | 0.84 | 0.84 | -0.58 | -0.60 | -0.72 | 1.73 | 0.60 | 0.32 |
| Q9UM22 | Mammalian ependymin-related protein 1 | EPDR1 | 0.43 | 1.00 | 0.98 | -1.08 | -1.25 | -0.89 | 1.09 | 0.54 | 1.18 | 0.82 | -0.71 | -0.68 |
| P62993 | Growth factor receptor-bound protein 2 | GRB2 | 0.43 | 1.12 | -0.48 | 0.40 | -0.47 | 0.21 | -0.94 | 0.25 | -1.67 | 2.03 | 0.71 | -0.03 |
| Q15034 | Probable E3 ubiquitin-protein ligase HERC3 | HERC3 | 0.43 | 0.73 | 0.65 | -0.79 | -1.10 | 0.01 | 0.34 | 0.35 | 1.75 | -1.42 | 0.95 | -0.73 |
| Q7Z392 | Trafficking protein particle complex subunit 11 | TRAPPC11 | 0.42 | 0.84 | 0.45 | 1.11 | -2.03 | 0.37 | -0.92 | 0.15 | 1.36 | -0.68 | -0.14 | 0.32 |
| P61586 | Transforming protein RhoA | RHOA | 0.42 | 1.87 | 0.21 | -0.12 | -0.12 | 0.34 | -2.13 | 1.88 | -0.69 | -0.02 | 0.41 | 0.24 |
| O95848 | Uridine diphosphate glucose pyrophosphatase NUDT14 | NUDT14 | 0.42 | 1.09 | 1.55 | 0.03 | -0.32 | -1.72 | -0.77 | 0.78 | -0.52 | 0.91 | 0.81 | -0.76 |
| P52565 | Rho GDP-dissociation inhibitor 1 | ARHGDIA | 0.42 | 1.42 | -0.96 | 1.32 | -0.04 | 0.24 | -2.10 | 0.32 | 1.33 | -0.12 | 0.02 | -0.03 |
| P13489 | Ribonuclease inhibitor | RNH1 | 0.42 | 1.35 | 0.21 | -0.45 | 0.56 | 0.75 | -2.55 | 0.76 | -0.31 | 0.08 | 0.84 | 0.12 |
| Q10567 | AP-1 complex subunit beta-1 | AP1B1 | 0.42 | 1.98 | -0.38 | 0.87 | -0.58 | -0.21 | -1.59 | 1.12 | 1.17 | -1.41 | 0.33 | 0.69 |
| P05556 | Integrin beta-1 | ITGB1 | 0.42 | 0.74 | 0.89 | 0.02 | -1.35 | 0.62 | -1.08 | 1.38 | 0.33 | 1.01 | -1.18 | -0.64 |
| P17026 | Zinc finger protein 22 | ZNF22 | 0.42 | 1.77 | 0.55 | -0.46 | -1.62 | 0.78 | -1.01 | -0.42 | -0.77 | 0.88 | 0.51 | 1.55 |
| Q9NZN5 | Rho guanine nucleotide exchange factor 12 | ARHGEF12 | 0.42 | 1.73 | -1.84 | -0.02 | 0.32 | 0.05 | -0.24 | -1.28 | 0.92 | -0.12 | 0.60 | 1.61 |
| Q13510 | Acid ceramidase | ASAH1 | 0.42 | 0.86 | -1.43 | -0.49 | -0.61 | 0.32 | 1.20 | -0.12 | -1.45 | 1.34 | 0.40 | 0.85 |
| B5ME19 | Eukaryotic translation initiation factor 3 subunit C-like protein | EIF3CL | 0.42 | 1.02 | 0.13 | -0.12 | -0.71 | -0.08 | -0.43 | -0.28 | -0.26 | -0.86 | -0.10 | 2.72 |
| P12036 | Neurofilament heavy polypeptide | NEFH | 0.42 | 1.23 | -0.94 | -1.89 | 0.58 | -0.54 | 1.42 | 0.60 | -0.58 | 1.06 | 0.01 | 0.28 |
| P41252 | Isoleucine--tRNA ligase, cytoplasmic | IARS | 0.42 | 2.45 | 0.21 | -0.82 | -1.76 | -0.03 | 0.22 | 0.56 | 1.27 | -0.89 | -0.29 | 1.53 |
| P22570 | NADPH:adrenodoxin oxidoreductase, mitochondrial | FDXR | 0.42 | 0.53 | -0.28 | -0.72 | 2.12 | -1.14 | -0.68 | 0.73 | 1.01 | -0.43 | 0.00 | -0.63 |
| Q92973 | Transportin-1 | TNPO1 | 0.42 | 1.01 | -0.88 | -0.48 | -0.38 | 0.32 | 0.22 | 0.71 | -1.70 | -0.35 | 0.55 | 1.98 |
| P28066 | Proteasome subunit alpha type-5 | PSMA5 | 0.42 | 1.12 | -1.13 | -1.51 | -0.02 | 0.04 | 1.36 | -0.24 | -0.33 | 1.83 | 0.21 | -0.22 |
| P61201 | COP9 signalosome complex subunit 2 | COPS2 | 0.42 | 0.68 | -1.14 | 1.37 | -1.77 | 0.82 | -0.14 | -0.50 | 0.98 | -0.55 | 0.64 | 0.29 |
| P52758 | 2-iminobutanoate/2-iminopropanoate deaminase | RIDA | 0.42 | 0.41 | -0.85 | -0.75 | -0.53 | 0.85 | 0.74 | -0.85 | -1.51 | 1.42 | 0.91 | 0.57 |
| P62942 | Peptidyl-prolyl cis-trans isomerase FKBP1A | FKBP1A | 0.42 | 1.17 | 0.46 | 0.44 | -1.38 | 0.11 | -0.94 | -1.37 | 1.28 | 1.19 | 0.74 | -0.54 |
| Q86VW0 | SEC14 domain and spectrin repeat-containing protein 1 | SESTD1 | 0.41 | 0.98 | 0.77 | -0.53 | 0.53 | -0.30 | -1.61 | 0.33 | 1.95 | -1.12 | 0.05 | -0.08 |
| P61247 | 40S ribosomal protein S3a | RPS3A | 0.41 | 0.78 | 1.06 | -0.85 | 0.56 | -0.35 | -1.36 | 1.18 | 0.32 | 0.98 | 0.01 | -1.55 |
| Q9NZD2 | Glycolipid transfer protein | GLTP | 0.41 | 0.44 | 0.72 | -1.02 | -1.19 | 0.56 | 0.35 | -0.25 | -1.03 | 2.08 | 0.12 | -0.34 |
| P57721 | Poly(rC)-binding protein 3 | PCBP3 | 0.41 | 0.75 | 0.91 | -2.06 | -0.89 | 0.52 | 0.60 | 0.81 | 0.11 | -1.08 | 0.32 | 0.75 |

| | | | | | | | | | | | | | | |
|---|---|---|---|---|---|---|---|---|---|---|---|---|---|---|
| P0DP04 | Immunoglobulin heavy variable 3-43D | IGHV3-43D | 0.41 | 0.38 | -1.18 | 0.49 | 0.47 | 0.39 | -0.69 | -1.26 | -1.28 | 1.23 | 0.64 | 1.18 |
| P21399 | Cytoplasmic aconitate hydratase | ACO1 | 0.41 | 1.39 | -0.31 | 0.88 | -1.72 | 1.03 | -1.38 | -0.81 | 0.36 | 0.58 | 0.39 | 0.98 |
| P53041 | Serine/threonine-protein phosphatase 5 | PPP5C | 0.41 | 0.83 | 0.34 | -0.32 | -0.75 | 0.22 | -0.51 | -1.35 | 0.03 | 2.47 | -0.05 | -0.07 |
| P60660 | Myosin light polypeptide 6 | MYL6 | 0.41 | 2.20 | -0.91 | 1.72 | -1.38 | -0.79 | -0.73 | 0.77 | 0.38 | 0.12 | -0.32 | 1.14 |
| Q00796 | Sorbitol dehydrogenase | SORD | 0.41 | 1.01 | 0.89 | -0.42 | 0.91 | -0.70 | -1.84 | 1.67 | 0.08 | -0.39 | -0.52 | 0.32 |
| Q96P70 | Importin-9 | IPO9 | 0.41 | 0.57 | 1.18 | -1.23 | -0.62 | 0.04 | -0.10 | 1.26 | -1.42 | 1.25 | 0.30 | -0.67 |
| Q07021 | Complement component 1 Q subcomponent-binding protein, mito | C1QBP | 0.41 | 0.48 | 0.98 | -1.16 | -0.74 | -0.61 | 0.91 | 0.79 | -1.39 | 1.46 | -0.46 | 0.23 |
| Q9NP72 | Ras-related protein Rab-18 | RAB18 | 0.41 | 1.74 | -0.92 | 0.68 | 0.31 | 0.29 | -2.11 | -0.69 | 1.49 | 0.20 | 0.55 | 0.20 |
| Q14204 | Cytoplasmic dynein 1 heavy chain 1 | DYNC1H1 | 0.41 | 0.87 | 0.54 | 0.35 | -1.52 | 0.57 | -0.98 | 1.57 | 0.26 | -1.45 | 0.62 | 0.04 |
| P53396 | ATP-citrate synthase | ACLY | 0.41 | 0.55 | -0.61 | -0.39 | 0.36 | -0.20 | 0.13 | -0.74 | -0.92 | -0.15 | -0.09 | 2.62 |
| Q6L8Q7 | 2',5'-phosphodiesterase 12 | PDE12 | 0.41 | 0.81 | 0.78 | 0.43 | -0.24 | 0.35 | -2.30 | 1.10 | 0.39 | -1.09 | 0.39 | 0.19 |
| Q96F63 | Coiled-coil domain-containing protein 97 | CCDC97 | 0.41 | 0.75 | 1.29 | -0.53 | -0.74 | -0.37 | -0.56 | 2.14 | -0.53 | -0.80 | -0.46 | 0.57 |
| Q9NVP1 | ATP-dependent RNA helicase DDX18 | DDX18 | 0.41 | 0.81 | 0.27 | 0.37 | -1.05 | 0.28 | -0.86 | 0.57 | -0.89 | 1.77 | 0.90 | -1.36 |
| P49748 | Very long-chain specific acyl-CoA dehydrogenase, mitochondrial | ACADVL | 0.41 | 1.67 | -1.89 | -1.54 | 1.04 | 0.94 | -0.32 | 0.18 | -0.01 | 0.76 | 0.50 | 0.33 |
| Q8NBF2 | NHL repeat-containing protein 2 | NHLRC2 | 0.41 | 0.47 | -1.21 | 0.29 | 0.81 | 0.26 | -0.77 | 1.28 | 0.40 | -1.98 | 0.41 | 0.51 |
| P20618 | Proteasome subunit beta type-1 | PSMB1 | 0.41 | 1.05 | -0.97 | -1.43 | 0.31 | -0.10 | 0.99 | -0.62 | -0.61 | 2.02 | 0.31 | 0.10 |
| Q7RTV0 | PHD finger-like domain-containing protein 5A | PHF5A | 0.40 | 1.33 | 0.92 | 0.98 | -1.93 | -0.32 | -1.10 | 0.52 | 0.18 | -0.54 | 0.04 | 1.26 |
| Q9Y6C9 | Mitochondrial carrier homolog 2 | MTCH2 | 0.40 | 3.75 | 0.23 | -0.69 | -1.05 | -0.27 | -1.09 | -0.20 | -0.16 | 1.87 | -0.22 | 1.58 |
| Q12860 | Contactin-1 | CNTN1 | 0.40 | 1.38 | -0.35 | 0.53 | -0.84 | -0.08 | -0.77 | -1.41 | 0.70 | 2.06 | 0.60 | -0.44 |
| Q96HY6 | DDRGK domain-containing protein 1 | DDRGK1 | 0.40 | 0.40 | 0.77 | 0.64 | -1.35 | 0.64 | -1.23 | 0.49 | 0.10 | -1.63 | 0.54 | 1.03 |
| Q02543 | 60S ribosomal protein L18a | RPL18A | 0.40 | 1.08 | -0.25 | -1.87 | -1.06 | 0.11 | 1.80 | 0.01 | 0.40 | -0.27 | 0.24 | 0.89 |
| Q03591 | Complement factor H-related protein 1 | CFHR1 | 0.40 | 0.44 | 1.10 | -0.21 | -1.34 | 0.36 | -0.49 | 0.79 | 0.62 | -1.76 | -0.26 | 1.19 |
| O95502 | Neuronal pentraxin receptor | NPTXR | 0.40 | 1.19 | 0.67 | 0.50 | -0.93 | -0.40 | -1.16 | 1.53 | -0.42 | 1.40 | 0.02 | -1.21 |
| P21359 | Neurofibromin | NF1 | 0.40 | 0.73 | 0.70 | -0.17 | -1.21 | 0.68 | -0.88 | 1.16 | 1.40 | -0.82 | 0.43 | -1.27 |
| Q9P0L0 | Vesicle-associated membrane protein-associated protein A | VAPA | 0.40 | 1.67 | 0.55 | -0.19 | -1.32 | 0.06 | -0.81 | -0.12 | 1.75 | 1.43 | -0.35 | -1.01 |
| O43426 | Synaptojanin-1 | SYNJ1 | 0.40 | 3.77 | -0.77 | -0.76 | -0.73 | -0.69 | -0.05 | 1.37 | 2.11 | -0.15 | 0.30 | -0.62 |
| P24666 | Low molecular weight phosphotyrosine protein phosphatase | ACP1 | 0.40 | 0.92 | -0.53 | -0.92 | 0.39 | -1.00 | 0.97 | -1.50 | 0.24 | 1.55 | -0.23 | 1.02 |
| Q06278 | Aldehyde oxidase | AOX1 | 0.39 | 1.01 | -0.20 | -0.68 | 0.92 | -0.41 | -0.80 | 0.28 | -0.43 | 2.43 | -0.71 | -0.40 |
| Q567U6 | Coiled-coil domain-containing protein 93 | CCDC93 | 0.39 | 0.74 | -0.46 | 1.41 | -0.27 | 0.56 | -2.16 | 0.86 | 0.52 | -0.82 | 0.10 | 0.25 |
| Q5JSH3 | WD repeat-containing protein 44 | WDR44 | 0.39 | 1.14 | 1.29 | -0.60 | -0.77 | -0.08 | -1.11 | 0.68 | 1.97 | -0.77 | -0.27 | -0.35 |
| Q96S15 | GATOR complex protein WDR24 | WDR24 | 0.39 | 0.96 | 0.03 | -0.38 | -0.52 | -0.04 | -0.24 | -0.35 | -0.25 | -0.71 | -0.31 | 2.78 |
| P50225 | Sulfotransferase 1A1 | SULT1A1 | 0.39 | 0.60 | -1.09 | 0.54 | -0.85 | 0.94 | -0.30 | 0.20 | -1.74 | 0.03 | 0.83 | 1.44 |
| O00186 | Syntaxin-binding protein 3 | STXBP3 | 0.39 | 0.84 | 1.59 | -0.51 | -1.10 | 0.05 | -1.04 | 1.23 | 0.98 | -1.21 | 0.06 | -0.05 |
| P31930 | Cytochrome b-c1 complex subunit 1, mitochondrial | UQCRC1 | 0.39 | 0.86 | 0.35 | -0.21 | 0.42 | -0.17 | -1.42 | 1.33 | 1.38 | -1.71 | -0.03 | 0.06 |
| Q96BP3 | Peptidylprolyl isomerase domain and WD repeat-containing protei | PPWD1 | 0.39 | 0.75 | 0.30 | 1.13 | -0.71 | -1.54 | -0.10 | 0.58 | -1.70 | 0.37 | 0.73 | 0.93 |
| Q9H4G4 | Golgi-associated plant pathogenesis-related protein 1 | GLIPR2 | 0.39 | 0.87 | 0.33 | -0.12 | -0.59 | -2.10 | 1.43 | 0.90 | -0.08 | -0.80 | 0.72 | 0.31 |
| P52735 | Guanine nucleotide exchange factor VAV2 | VAV2 | 0.39 | 0.56 | 1.58 | -0.48 | -0.68 | -0.50 | -0.63 | 1.34 | -0.50 | -0.92 | -0.59 | 1.38 |
| O60216 | Double-strand-break repair protein rad21 homolog | RAD21 | 0.39 | 0.90 | 1.15 | -1.26 | -1.25 | 0.29 | 0.02 | 1.75 | 0.43 | -1.08 | 0.06 | -0.11 |
| Q9Y5V3 | Melanoma-associated antigen D1 | MAGED1 | 0.39 | 0.88 | 0.86 | 0.21 | 0.07 | -0.36 | -1.81 | 0.56 | 0.93 | -1.58 | 0.05 | 1.08 |
| Q9UGR2 | Zinc finger CCCH domain-containing protein 7B | ZC3H7B | 0.39 | 0.65 | 1.46 | -0.52 | -2.01 | 0.25 | -0.01 | 0.77 | 1.14 | -0.63 | 0.01 | -0.46 |
| Q96CX2 | BTB/POZ domain-containing protein KCTD12 | KCTD12 | 0.39 | 3.30 | -0.72 | 0.36 | 0.13 | -0.19 | -2.20 | 0.42 | 0.68 | 1.33 | 0.81 | -0.61 |
| Q92541 | RNA polymerase-associated protein RTF1 homolog | RTF1 | 0.38 | 0.55 | -0.40 | -0.48 | 1.35 | -0.54 | -0.64 | -0.47 | 2.17 | -0.88 | -0.50 | 0.39 |
| Q9UL15 | BAG family molecular chaperone regulator 5 | BAG5 | 0.38 | 0.45 | -1.55 | -0.63 | 0.29 | 0.17 | 1.13 | -1.74 | 0.64 | 1.11 | 0.36 | 0.22 |
| Q9BYV8 | Centrosomal protein of 41 kDa | CEP41 | 0.38 | 0.66 | 1.40 | 0.64 | -1.76 | -1.52 | 0.40 | -0.22 | 0.67 | 0.22 | 0.59 | -0.41 |
| P62256 | Ubiquitin-conjugating enzyme E2 H | UBE2H | 0.38 | 0.64 | 0.39 | 0.08 | -0.76 | 0.58 | -1.10 | 1.33 | -0.26 | -1.87 | 0.63 | 0.97 |
| P42025 | Beta-centractin | ACTR1B | 0.38 | 0.90 | -0.29 | 0.38 | -0.58 | -0.19 | -0.40 | -1.36 | -0.20 | 2.50 | 0.28 | -0.14 |
| O60343 | TBC1 domain family member 4 | TBC1D4 | 0.38 | 0.81 | -1.12 | -1.20 | 0.25 | 0.97 | 0.13 | -0.23 | -1.59 | 0.99 | 1.13 | 0.68 |
| P04406 | Glyceraldehyde-3-phosphate dehydrogenase | GAPDH | 0.38 | 0.65 | -0.99 | -0.83 | 0.38 | 0.94 | -0.31 | -0.05 | -1.34 | 1.76 | 1.01 | -0.56 |
| Q86TN4 | tRNA 2'-phosphotransferase 1 | TRPT1 | 0.38 | 0.45 | 0.64 | -0.81 | -0.94 | 1.37 | -0.86 | 0.29 | -0.72 | -1.19 | 1.14 | 1.06 |
| Q9H4M9 | EH domain-containing protein 1 | EHD1 | 0.38 | 0.40 | -2.27 | 1.54 | 0.15 | 0.24 | -0.20 | -0.71 | 0.32 | -0.24 | 0.54 | 0.63 |
| O75891 | Cytosolic 10-formyltetrahydrofolate dehydrogenase | ALDH1L1 | 0.38 | 0.66 | -1.13 | -1.97 | 0.58 | 0.08 | 1.59 | 0.02 | -0.42 | 0.09 | 0.32 | 0.84 |
| O14966 | Ras-related protein Rab-7L1 | RAB29 | 0.38 | 0.67 | 0.05 | -0.23 | -0.55 | 0.64 | -0.76 | 1.26 | -1.62 | -0.93 | 0.68 | 1.46 |
| Q02978 | Mitochondrial 2-oxoglutarate/malate carrier protein | SLC25A11 | 0.38 | 0.72 | 1.83 | -0.41 | -0.95 | -0.85 | -0.51 | -0.59 | -0.43 | -0.46 | 1.32 | 1.05 |
| O60888 | Protein CutA | CUTA | 0.38 | 0.28 | -0.61 | -0.74 | 0.93 | -0.68 | 0.71 | -0.66 | -0.73 | 2.15 | -0.72 | 0.35 |
| Q9H9C1 | Spermatogenesis-defective protein 39 homolog | VIPAS39 | 0.38 | 1.20 | -0.32 | 1.57 | -0.94 | -0.37 | -1.27 | -0.41 | 1.78 | -0.32 | -0.29 | 0.55 |
| Q9Y6U3 | Adseverin | SCIN | 0.38 | 0.55 | -0.18 | -0.20 | -1.82 | -0.33 | 1.83 | 1.16 | -0.39 | -0.19 | -0.48 | 0.61 |
| Q7Z3D6 | D-glutamate cyclase, mitochondrial | DGLUCY | 0.38 | 0.32 | -1.12 | 0.41 | 1.30 | 0.25 | -1.26 | -0.32 | 0.71 | -1.52 | 0.58 | 0.99 |
| Q8N0W3 | L-fucose kinase | FCSK | 0.38 | 0.65 | -0.03 | -0.91 | -0.10 | 0.80 | -0.58 | 0.35 | -1.86 | 1.86 | 0.37 | 0.10 |
| Q9NRY5 | Protein FAM114A2 | FAM114A2 | 0.38 | 0.54 | -0.92 | 1.22 | 0.12 | -0.36 | -0.75 | 0.13 | 1.42 | -1.78 | 0.07 | 0.86 |
| Q68E01 | Integrator complex subunit 3 | INTS3 | 0.38 | 0.55 | 0.63 | -1.12 | -0.54 | -0.27 | 0.59 | 2.00 | 0.89 | -0.32 | -0.98 | -0.89 |
| Q9H4A4 | Aminopeptidase B | RNPEP | 0.37 | 0.65 | -1.61 | 0.49 | 0.79 | -0.01 | -0.48 | 0.83 | -1.86 | 0.50 | 0.60 | 0.74 |
| Q9Y371 | Endophilin-B1 | SH3GLB1 | 0.37 | 0.55 | -0.09 | 1.30 | -2.29 | 0.69 | -0.33 | 0.40 | -0.22 | -0.68 | 0.72 | 0.50 |
| Q9UBF2 | Coatomer subunit gamma-2 | COPG2 | 0.37 | 0.68 | -1.69 | -0.17 | -0.35 | 0.56 | 0.80 | -1.48 | -0.34 | 0.99 | 1.25 | 0.43 |
| Q8N9F0 | N-acetylaspartate synthetase | NAT8L | 0.37 | 0.40 | 0.36 | 1.23 | -1.33 | 0.56 | -1.35 | 0.36 | 0.29 | -1.51 | 0.66 | 0.73 |
| O43242 | 26S proteasome non-ATPase regulatory subunit 3 | PSMD3 | 0.37 | 0.56 | 0.94 | -1.54 | 0.27 | -0.81 | 0.43 | -0.33 | 1.87 | 0.35 | -1.05 | -0.12 |
| Q9BT40 | Inositol polyphosphate 5-phosphatase K | INPP5K | 0.37 | 0.60 | 0.88 | 0.66 | -1.75 | 0.30 | -0.85 | 0.74 | 0.39 | -1.48 | 0.09 | 1.01 |
| A1L390 | Pleckstrin homology domain-containing family G member 3 | PLEKHG3 | 0.37 | 0.83 | 0.04 | -0.12 | -0.48 | -0.28 | -0.19 | -0.31 | -0.24 | -1.03 | -0.13 | 2.72 |
| P63244 | Receptor of activated protein C kinase 1 | RACK1 | 0.37 | 2.40 | -0.87 | -2.38 | -0.01 | 0.83 | 0.19 | 0.46 | 1.07 | 0.22 | 0.63 | -0.15 |
| P13987 | CD59 glycoprotein | CD59 | 0.37 | 1.34 | 0.11 | 1.45 | -2.14 | -0.27 | -0.61 | -0.22 | -0.11 | 0.70 | -0.04 | 1.14 |
| P63302 | Selenoprotein W | SELENOW | 0.37 | 0.78 | 0.93 | -0.24 | -2.21 | 0.49 | 0.09 | 0.97 | 1.04 | -0.97 | -0.08 | -0.01 |
| Q5VU43 | Myomegalin | PDE4DIP | 0.37 | 0.62 | -1.05 | -1.19 | -0.21 | 0.60 | 1.07 | -1.25 | -0.49 | 1.65 | 0.43 | 0.44 |
| O00541 | Pescadillo homolog | PES1 | 0.37 | 0.53 | -0.97 | 0.00 | -1.33 | 0.69 | 0.93 | -0.32 | 1.09 | -1.56 | 0.44 | 1.01 |
| O60504 | Vinexin | SORBS3 | 0.37 | 0.36 | 1.71 | -0.01 | -0.90 | 0.37 | -1.66 | -0.39 | 0.75 | -1.00 | 0.48 | 0.65 |
| O76013 | Keratin, type I cuticular Ha6 | KRT36 | 0.37 | 0.64 | 1.33 | 0.93 | -1.71 | -0.82 | -0.53 | 0.04 | 0.04 | 1.34 | -0.80 | 0.18 |
| Q05639 | Elongation factor 1-alpha 2 | EEF1A2 | 0.37 | 1.10 | -0.01 | -0.34 | -1.25 | 0.55 | -0.20 | 1.59 | -0.25 | -1.71 | 0.54 | 1.09 |
| Q13617 | Cullin-2 | CUL2 | 0.37 | 1.15 | 0.54 | -0.97 | -1.43 | 1.04 | -0.45 | -0.35 | 1.90 | -0.12 | 0.51 | -0.65 |
| P36405 | ADP-ribosylation factor-like protein 3 | ARL3 | 0.37 | 1.89 | -0.53 | 0.05 | -0.19 | -0.01 | -1.21 | 1.88 | 0.79 | -1.62 | 0.25 | 0.60 |
| O95834 | Echinoderm microtubule-associated protein-like 2 | EML2 | 0.37 | 0.65 | 0.35 | -0.99 | 1.78 | -0.91 | -1.04 | 0.61 | 0.00 | 1.34 | -0.57 | -0.56 |
| P59665 | Neutrophil defensin 1 | DEFA1 | 0.37 | 0.32 | 0.81 | -1.50 | -0.60 | -0.58 | 1.43 | 0.29 | -1.45 | 0.58 | 0.11 | 0.90 |
| Q14847 | LIM and SH3 domain protein 1 | LASP1 | 0.37 | 0.68 | 0.50 | 1.24 | -2.40 | -0.41 | 0.21 | -0.44 | 0.70 | 0.61 | -0.33 | 0.32 |
| Q15370 | Elongin-B | ELOB | 0.37 | 1.12 | -0.02 | 0.39 | -1.51 | 0.34 | -0.46 | 1.03 | 0.92 | -1.90 | 0.65 | 0.56 |
| Q06210 | Glutamine--fructose-6-phosphate aminotransferase [isomerizing] | GFPT1 | 0.37 | 0.36 | 0.89 | -0.13 | 0.33 | -1.60 | 0.01 | 0.94 | 0.65 | -1.93 | 0.69 | 0.13 |
| O15068 | Guanine nucleotide exchange factor DBS | MCF2L | 0.37 | 0.69 | 0.14 | 0.10 | -0.62 | 1.38 | -1.87 | -0.80 | -0.39 | -0.02 | 1.37 | 0.70 |

| | | | | | | | | | | | | | | |
|---|---|---|---|---|---|---|---|---|---|---|---|---|---|---|
| P06753 | Tropomyosin alpha-3 chain | TPM3 | 0.37 | 0.57 | -1.29 | 0.75 | -0.24 | -0.24 | 0.29 | -0.31 | 0.01 | 2.35 | -0.43 | -0.89 |
| Q8NBQ5 | Estradiol 17-beta-dehydrogenase 11 | HSD17B11 | 0.37 | 0.43 | -0.80 | 1.28 | -0.66 | 0.80 | -1.20 | 0.50 | -0.21 | -1.42 | 1.32 | 0.37 |
| P27338 | Amine oxidase [flavin-containing] B | MAOB | 0.37 | 0.88 | -0.87 | -0.79 | 0.90 | 0.61 | -0.89 | 0.81 | -1.77 | 0.17 | 1.18 | 0.65 |
| O76054 | SEC14-like protein 2 | SEC14L2 | 0.37 | 0.59 | -0.32 | 0.44 | -0.35 | 0.18 | -0.71 | 1.46 | -0.21 | -2.06 | 0.35 | 1.23 |
| Q6MZT1 | Regulator of G-protein signaling 7-binding protein | RGS7BP | 0.37 | 1.30 | 0.09 | -0.31 | -0.64 | -0.07 | -0.52 | 0.10 | -0.13 | -1.03 | -0.14 | 2.66 |
| P78417 | Glutathione S-transferase omega-1 | GSTO1 | 0.37 | 0.39 | -1.32 | -1.25 | 2.04 | 0.39 | -0.39 | -0.57 | -0.26 | 0.74 | 0.53 | 0.09 |
| O60749 | Sorting nexin-2 | SNX2 | 0.37 | 1.27 | -1.92 | 0.84 | 0.60 | 0.40 | -1.31 | 0.50 | 0.49 | -0.82 | 1.11 | 0.12 |
| P19823 | Inter-alpha-trypsin inhibitor heavy chain H2 | ITIH2 | 0.37 | 0.39 | -0.61 | -0.97 | 0.98 | 0.72 | -0.65 | 0.93 | 0.45 | 1.43 | -1.24 | -1.05 |
| P50238 | Cysteine-rich protein 1 | CRIP1 | 0.37 | 1.19 | 0.16 | -0.20 | -0.65 | -0.32 | -0.35 | -0.04 | -0.09 | -0.82 | -0.41 | 2.73 |
| Q9NWH9 | SAFB-like transcription modulator | SLTM | 0.37 | 0.98 | 1.58 | -0.07 | 0.11 | -1.20 | -1.56 | 1.13 | -0.73 | 0.88 | -0.01 | -0.12 |
| Q8WXI9 | Transcriptional repressor p66-beta | GATAD2B | 0.37 | 0.61 | 1.63 | -1.27 | -0.13 | -0.13 | -0.88 | 1.04 | 0.86 | 0.58 | -0.48 | -1.24 |
| Q460N5 | Protein mono-ADP-ribosyltransferase PARP14 | PARP14 | 0.36 | 0.58 | -0.07 | 0.56 | -0.84 | -0.06 | -0.35 | 0.95 | 2.19 | -1.19 | -0.71 | -0.48 |
| Q9Y265 | RuvB-like 1 | RUVBL1 | 0.36 | 1.67 | 0.02 | -0.75 | -1.40 | -0.84 | 1.29 | -0.67 | 1.22 | 1.13 | -0.67 | 0.67 |
| P62495 | Eukaryotic peptide chain release factor subunit 1 | ETF1 | 0.36 | 0.67 | -1.21 | -0.14 | 0.60 | 0.83 | -0.90 | -1.65 | -0.32 | 0.55 | 1.22 | 1.02 |
| Q6WCQ1 | Myosin phosphatase Rho-interacting protein | MPRIP | 0.36 | 1.24 | -0.30 | -1.19 | 1.00 | -0.40 | -0.49 | 0.45 | -0.95 | 2.18 | 0.14 | -0.45 |
| Q14966 | Zinc finger protein 638 | ZNF638 | 0.36 | 0.26 | 0.53 | 0.66 | -1.82 | 0.13 | 0.14 | 0.26 | 0.62 | 1.16 | 0.10 | -1.78 |
| O43837 | Isocitrate dehydrogenase [NAD] subunit beta, mitochondrial | IDH3B | 0.36 | 0.87 | 0.55 | -1.06 | 1.67 | -0.91 | -1.29 | 1.15 | 0.69 | 0.08 | -0.68 | -0.19 |
| O75995 | SAM and SH3 domain-containing protein 3 | SASH3 | 0.36 | 0.38 | -0.64 | 0.90 | -0.90 | 0.86 | -0.74 | -0.65 | -0.77 | 1.96 | 0.62 | -0.65 |
| Q6ZMI0 | Protein phosphatase 1 regulatory subunit 21 | PPP1R21 | 0.36 | 0.42 | -1.32 | 0.82 | 0.03 | 0.44 | -0.52 | 0.44 | 1.49 | -1.88 | 0.29 | 0.21 |
| P20962 | Parathymosin | PTMS | 0.36 | 0.56 | 0.11 | 0.64 | -1.98 | -0.21 | 0.73 | 0.27 | 1.24 | 0.83 | -0.33 | -1.30 |
| Q9UBF6 | RING-box protein 2 | RNF7 | 0.36 | 0.30 | 0.32 | 0.33 | -1.92 | 0.57 | 0.28 | 0.48 | -1.84 | 0.65 | 0.67 | 0.46 |
| P60842 | Eukaryotic initiation factor 4A-I | EIF4A1 | 0.36 | 1.29 | 0.57 | -0.65 | -0.15 | 0.62 | -1.79 | 1.08 | 0.09 | -1.42 | 0.59 | 1.06 |
| P16333 | Cytoplasmic protein NCK1 | NCK1 | 0.36 | 0.36 | 0.36 | 0.95 | -1.90 | 0.88 | -0.77 | 0.65 | -0.95 | -0.72 | 0.81 | 0.70 |
| Q9UG56 | Phosphatidylserine decarboxylase proenzyme, mitochondrial | PISD | 0.36 | 0.58 | 0.76 | 1.37 | -1.02 | -0.23 | -1.62 | -0.45 | 0.92 | -0.57 | -0.30 | 1.15 |
| P07197 | Neurofilament medium polypeptide | NEFM | 0.36 | 1.62 | -0.38 | -0.17 | 1.40 | -0.94 | -1.57 | 0.41 | 0.53 | 1.57 | -0.80 | -0.05 |
| Q13625 | Apoptosis-stimulating of p53 protein 2 | TP53BP2 | 0.36 | 0.47 | 0.12 | -1.66 | 1.28 | 0.06 | -0.42 | 1.09 | -1.58 | 0.01 | 0.31 | 0.79 |
| P38646 | Stress-70 protein, mitochondrial | HSPA9 | 0.36 | 0.67 | -1.14 | 0.06 | 1.86 | -1.46 | -0.17 | -0.78 | 0.78 | 0.41 | -0.33 | 0.76 |
| Q9UIC8 | Leucine carboxyl methyltransferase 1 | LCMT1 | 0.36 | 0.56 | -1.16 | -0.39 | -0.88 | 0.75 | 0.97 | -0.89 | -1.28 | 1.04 | 0.85 | 1.00 |
| Q05397 | Focal adhesion kinase 1 | PTK2 | 0.36 | 1.09 | -0.12 | -0.07 | 1.30 | -1.14 | -1.20 | -0.65 | 0.89 | 1.74 | -0.52 | -0.24 |
| Q9UEY8 | Gamma-adducin | ADD3 | 0.36 | 1.31 | -0.56 | -1.34 | -0.68 | 0.58 | 0.57 | 0.30 | -1.45 | 1.74 | 0.73 | 0.10 |
| Q13232 | Nucleoside diphosphate kinase 3 | NME3 | 0.36 | 0.44 | 0.26 | 0.68 | -1.66 | 0.11 | 0.01 | 0.87 | 0.01 | -1.86 | 1.15 | 0.41 |
| Q9HBH5 | Retinol dehydrogenase 14 | RDH14 | 0.35 | 0.54 | -0.29 | 1.61 | -0.87 | -0.51 | -0.64 | -0.47 | 1.52 | -0.92 | -0.54 | 1.11 |
| Q92994 | Transcription factor IIIB 90 kDa subunit | BRF1 | 0.35 | 0.60 | 0.87 | -0.26 | -0.62 | -0.25 | -0.49 | -0.37 | 2.54 | -0.83 | -0.22 | -0.36 |
| Q99584 | Protein S100-A13 | S100A13 | 0.35 | 0.71 | 0.03 | -1.49 | -1.49 | 0.16 | 1.89 | 0.12 | 0.16 | 0.66 | 0.46 | -0.50 |
| Q9Y646 | Carboxypeptidase Q | CPQ | 0.35 | 0.46 | 0.51 | 0.96 | -0.79 | -0.07 | -1.22 | 0.27 | -0.99 | 2.08 | -0.55 | -0.21 |
| P50750 | Cyclin-dependent kinase 9 | CDK9 | 0.35 | 0.59 | 1.41 | -0.37 | -0.81 | -0.53 | -0.44 | -0.43 | -0.56 | -0.10 | -0.41 | 2.25 |
| Q9BQQ3 | Golgi reassembly-stacking protein 1 | GORASP1 | 0.35 | 0.30 | 0.63 | -1.04 | -1.21 | 0.87 | 0.33 | 0.99 | -1.09 | -1.22 | 0.95 | 0.79 |
| P83881 | 60S ribosomal protein L36a | RPL36A | 0.35 | 1.58 | 0.18 | -2.50 | 0.00 | 0.48 | 0.19 | -0.33 | -0.06 | 0.13 | 0.46 | 1.45 |
| Q12913 | Receptor-type tyrosine-protein phosphatase eta | PTPRJ | 0.35 | 0.47 | -0.60 | 1.64 | -0.97 | 0.15 | -0.84 | -0.74 | 1.17 | -1.18 | 0.52 | 0.84 |
| O00308 | NEDD4-like E3 ubiquitin-protein ligase WWP2 | WWP2 | 0.35 | 0.39 | 0.22 | 0.17 | -0.54 | 0.72 | -1.10 | -0.19 | 1.38 | -1.86 | -0.03 | 1.23 |
| Q5T9A4 | ATPase family AAA domain-containing protein 3B | ATAD3B | 0.35 | 0.25 | 1.19 | -1.04 | 0.74 | -0.17 | -1.07 | 1.17 | 1.21 | -1.29 | -0.44 | -0.31 |
| P07858 | Cathepsin B | CTSB | 0.35 | 0.54 | -0.42 | -0.33 | 0.17 | -1.34 | 1.24 | -0.43 | 0.84 | 1.41 | -1.52 | 0.40 |
| Q9ULC4 | Malignant T-cell-amplified sequence 1 | MCTS1 | 0.35 | 0.89 | 0.57 | 0.48 | -0.14 | 0.23 | -2.19 | 0.38 | 1.59 | -0.90 | 0.21 | -0.23 |
| P80404 | 4-aminobutyrate aminotransferase, mitochondrial | ABAT | 0.35 | 0.84 | 0.03 | -0.94 | -0.63 | 0.06 | 0.46 | -0.23 | -0.68 | 2.57 | -0.03 | -0.62 |
| P62826 | GTP-binding nuclear protein Ran | RAN | 0.35 | 0.88 | 1.35 | -0.55 | -0.81 | -0.75 | -0.29 | 0.90 | 1.85 | -1.03 | -0.20 | -0.48 |
| P07602 | Prosaposin | PSAP | 0.35 | 0.45 | 0.15 | -1.12 | -1.09 | -0.64 | 2.10 | -0.66 | 0.88 | 0.45 | -0.44 | 0.37 |
| Q9Y2V2 | Calcium-regulated heat-stable protein 1 | CARHSP1 | 0.35 | 1.30 | 0.20 | -0.73 | -0.87 | 1.44 | -1.45 | 1.28 | 0.23 | -0.72 | 1.04 | -0.43 |
| Q8N9I0 | Synaptotagmin-2 | SYT2 | 0.35 | 0.40 | 0.65 | -0.36 | 0.24 | 0.16 | -1.23 | 1.31 | -1.85 | -0.43 | 0.34 | 1.17 |
| Q8IWX8 | Calcium homeostasis endoplasmic reticulum protein | CHERP | 0.35 | 0.38 | 1.38 | -1.14 | -1.30 | 0.19 | 0.36 | 1.13 | -0.05 | -1.46 | 0.68 | 0.22 |
| P46779 | 60S ribosomal protein L28 | RPL28 | 0.35 | 0.50 | -0.17 | -2.11 | -0.10 | -0.17 | 1.88 | 0.66 | 0.26 | -0.54 | -0.07 | 0.35 |
| Q9Y2T3 | Guanine deaminase | GDA | 0.34 | 0.68 | -0.60 | 0.04 | -0.42 | 0.53 | -0.41 | -1.03 | 0.68 | 2.01 | 0.63 | -1.43 |
| P62879 | Guanine nucleotide-binding protein G(I)/G(S)/G(T) subunit beta-2 | GNB2 | 0.34 | 1.60 | -1.07 | 0.81 | -1.50 | 0.50 | -0.38 | 0.81 | 0.52 | -1.43 | 0.66 | 1.08 |
| O15254 | Peroxisomal acyl-coenzyme A oxidase 3 | ACOX3 | 0.34 | 1.50 | 0.31 | -0.36 | -0.82 | -0.18 | -0.57 | -0.22 | -0.44 | 2.72 | -0.09 | -0.35 |
| Q8WUF8 | Cotranscriptional regulator FAM172A | FAM172A | 0.34 | 0.98 | 0.27 | -0.33 | -0.53 | -0.18 | -0.41 | 2.71 | -0.19 | -0.95 | -0.21 | -0.18 |
| Q32MZ4 | Leucine-rich repeat flightless-interacting protein 1 | LRRFIP1 | 0.34 | 0.87 | -0.42 | 1.78 | -1.13 | -0.59 | -0.67 | -0.77 | 1.40 | 0.47 | -0.67 | 0.62 |
| Q15833 | Syntaxin-binding protein 2 | STXBP2 | 0.34 | 0.46 | 1.45 | -0.83 | 0.81 | -0.19 | -1.84 | 0.50 | 1.09 | -0.69 | -0.44 | 0.15 |
| P61160 | Actin-related protein 2 | ACTR2 | 0.34 | 0.39 | 0.79 | 0.79 | -1.20 | 0.21 | -1.10 | 0.93 | 1.55 | -1.18 | -0.17 | -0.61 |
| P60763 | Ras-related C3 botulinum toxin substrate 3 | RAC3 | 0.34 | 0.36 | 0.63 | 1.22 | 0.07 | 0.12 | -2.53 | -0.28 | 0.61 | -0.30 | 0.09 | 0.37 |
| Q00325 | Phosphate carrier protein, mitochondrial | SLC25A3 | 0.34 | 0.47 | 0.70 | 0.71 | -1.73 | 0.37 | -0.66 | 0.83 | 0.78 | -1.50 | 0.88 | -0.37 |
| P63092 | Guanine nucleotide-binding protein G(s) subunit alpha isoforms sh | GNAS | 0.34 | 1.53 | -0.77 | 0.90 | -0.79 | 0.48 | -1.40 | 1.06 | -0.60 | -0.99 | 1.23 | 0.89 |
| P17931 | Galectin-3 | LGALS3 | 0.34 | 1.42 | 0.62 | 0.38 | -2.10 | 0.39 | -0.80 | 1.19 | 0.17 | -0.88 | 1.02 | 0.01 |
| Q9NZN4 | EH domain-containing protein 2 | EHD2 | 0.34 | 0.59 | -0.88 | 0.69 | 0.74 | 0.09 | -1.39 | -1.20 | 0.10 | -0.44 | 0.46 | 1.85 |
| P15328 | Folate receptor alpha | FOLR1 | 0.34 | 0.37 | 1.70 | -0.47 | -0.67 | -0.54 | -0.50 | 1.05 | -0.57 | -0.92 | -0.57 | 1.51 |
| P26639 | Threonine--tRNA ligase, cytoplasmic | TARS | 0.34 | 0.63 | -2.25 | 0.00 | 1.43 | 0.10 | -0.09 | 0.45 | -0.93 | 0.10 | 0.34 | 0.82 |
| Q15746 | Myosin light chain kinase, smooth muscle | MYLK | 0.34 | 0.63 | -1.14 | 0.35 | -0.36 | 1.47 | -1.10 | 0.56 | -0.30 | -1.36 | 1.12 | 0.77 |
| P15374 | Ubiquitin carboxyl-terminal hydrolase isozyme L3 | UCHL3 | 0.33 | 0.87 | -0.94 | 1.80 | -0.17 | 0.24 | -1.99 | -0.24 | 0.35 | -0.15 | 0.48 | 0.62 |
| Q1KMD3 | Heterogeneous nuclear ribonucleoprotein U-like protein 2 | HNRNPUL2 | 0.33 | 0.56 | 1.18 | -1.01 | -1.57 | 0.44 | 0.25 | 1.32 | 0.26 | -1.37 | 0.46 | 0.04 |
| P23588 | Eukaryotic translation initiation factor 4B | EIF4B | 0.33 | 0.63 | 0.84 | 0.71 | -0.03 | -0.50 | -1.82 | 0.97 | 1.15 | -0.74 | -1.09 | 0.50 |
| Q9UL26 | Ras-related protein Rab-22A | RAB22A | 0.33 | 0.54 | 1.43 | -0.11 | -0.97 | -0.18 | -0.87 | 1.10 | 1.40 | -1.37 | -0.01 | -0.42 |
| P51610 | Host cell factor 1 | HCFC1 | 0.33 | 0.80 | 1.46 | -0.89 | -1.22 | 0.20 | -0.52 | -0.16 | 1.91 | 0.20 | -0.34 | -0.66 |
| Q8IWB7 | WD repeat and FYVE domain-containing protein 1 | WDFY1 | 0.33 | 0.23 | 1.95 | -0.42 | -1.86 | -0.14 | 0.15 | 0.86 | 0.09 | -0.84 | -0.02 | 0.24 |
| P00387 | NADH-cytochrome b5 reductase 3 | CYB5R3 | 0.33 | 0.64 | -1.66 | -0.92 | 1.10 | -0.21 | 0.90 | -1.19 | 0.88 | 1.06 | -0.09 | 0.14 |
| Q9NSD9 | Phenylalanine--tRNA ligase beta subunit | FARSB | 0.33 | 0.56 | 1.95 | -0.91 | 0.06 | -0.45 | -1.37 | -0.54 | 0.71 | 1.10 | 0.00 | -0.55 |
| P02747 | Complement C1q subcomponent subunit C | C1QC | 0.33 | 2.22 | -2.20 | 0.75 | 0.09 | -0.75 | 0.05 | -0.12 | 1.28 | -0.08 | -0.17 | 1.16 |
| P10253 | Lysosomal alpha-glucosidase | GAA | 0.33 | 0.26 | -0.61 | 1.30 | -0.80 | -0.70 | 0.45 | -0.66 | -0.68 | 2.05 | -0.64 | 0.29 |
| O94973 | AP-2 complex subunit alpha-2 | AP2A2 | 0.33 | 0.73 | 0.41 | 0.91 | -1.29 | -0.37 | -0.56 | 0.30 | 2.17 | -0.83 | -0.61 | -0.13 |
| Q3SXM5 | Inactive hydroxysteroid dehydrogenase-like protein 1 | HSDL1 | 0.33 | 0.37 | -0.89 | 0.93 | 1.02 | -0.84 | -0.73 | -0.93 | 1.25 | 0.28 | -1.18 | 1.08 |
| Q16760 | Diacylglycerol kinase delta | DGKD | 0.33 | 0.78 | 0.56 | 0.09 | -0.94 | 0.50 | -1.17 | -1.64 | 1.53 | 0.79 | 0.62 | -0.36 |
| Q13310 | Polyadenylate-binding protein 4 | PABPC4 | 0.33 | 0.65 | 1.00 | 0.76 | -0.45 | 0.32 | -2.45 | 0.24 | -0.66 | 0.58 | 0.21 | 0.47 |
| Q8TAT6 | Nuclear protein localization protein 4 homolog | NPLOC4 | 0.33 | 1.19 | -0.82 | 1.48 | -1.54 | 0.53 | -0.98 | 0.49 | -0.65 | -0.28 | 0.55 | 1.22 |
| A3KMH1 | von Willebrand factor A domain-containing protein 8 | VWA8 | 0.33 | 0.39 | 1.19 | -0.62 | -0.82 | 0.34 | -0.61 | 1.56 | 1.30 | -1.00 | -0.72 | -0.62 |
| O60941 | Dystrobrevin beta | DTNB | 0.33 | 0.46 | -0.10 | -0.45 | -0.64 | 0.92 | -0.34 | -0.60 | -0.20 | -0.78 | -0.32 | 2.51 |
| Q9H7Z7 | Prostaglandin E synthase 2 | PTGES2 | 0.33 | 0.39 | -0.56 | -0.74 | -1.04 | 0.27 | 1.55 | -0.31 | -0.81 | 1.36 | 1.12 | -0.84 |

| | | | | | | | | | | | | | | |
|---|---|---|---|---|---|---|---|---|---|---|---|---|---|---|
| O75899 | Gamma-aminobutyric acid type B receptor subunit 2 | GABBR2 | 0.33 | 0.60 | -0.01 | 0.46 | -1.02 | 1.28 | -1.46 | -1.52 | 0.73 | 0.14 | 0.94 | 0.47 |
| P20839 | Inosine-5'-monophosphate dehydrogenase 1 | IMPDH1 | 0.33 | 1.82 | 0.46 | -0.63 | -0.81 | 0.41 | -1.24 | 1.35 | 1.56 | -0.64 | 0.56 | -1.02 |
| Q16678 | Cytochrome P450 1B1 | CYP1B1 | 0.33 | 0.47 | 0.86 | -1.10 | 0.86 | 0.58 | -1.82 | -0.28 | 0.71 | -1.10 | 0.51 | 0.78 |
| P09493 | Tropomyosin alpha-1 chain | TPM1 | 0.33 | 0.26 | 0.64 | 0.69 | 0.22 | -1.91 | 0.01 | -0.01 | -0.72 | 1.68 | -0.96 | 0.36 |
| Q9H4M3 | F-box only protein 44 | FBXO44 | 0.33 | 0.32 | 0.92 | 0.24 | -1.17 | 0.57 | -1.00 | -1.02 | 0.34 | -1.19 | 0.88 | 1.44 |
| P61254 | 60S ribosomal protein L26 | RPL26 | 0.33 | 0.83 | 0.73 | -1.87 | 0.57 | -0.04 | -0.38 | 0.33 | 1.07 | -1.38 | -0.17 | 1.14 |
| Q08117 | TLE family member 5 | TLE5 | 0.32 | 0.40 | -0.28 | -0.48 | -0.72 | -0.52 | 1.47 | -0.43 | -0.44 | 2.24 | -0.49 | -0.34 |
| P49593 | Protein phosphatase 1F | PPM1F | 0.32 | 1.75 | -1.87 | 0.09 | 0.63 | 0.02 | -0.60 | -0.53 | 1.28 | 1.47 | 0.27 | -0.75 |
| Q9UHX1 | Poly(U)-binding-splicing factor PUF60 | PUF60 | 0.32 | 0.61 | -1.42 | 1.82 | -1.42 | 0.62 | -0.38 | -0.41 | 0.48 | -0.29 | 0.78 | 0.22 |
| Q13496 | Myotubularin | MTM1 | 0.32 | 0.36 | -2.16 | 0.75 | 0.17 | 0.82 | -0.06 | -0.21 | -1.23 | 0.20 | 0.90 | 0.82 |
| Q8WXI2 | Connector enhancer of kinase suppressor of ras 2 | CNKSR2 | 0.32 | 0.53 | 0.12 | 0.53 | -2.36 | 0.37 | 0.66 | -0.81 | 0.76 | 1.02 | 0.20 | -0.49 |
| Q7Z5R6 | Amyloid beta A4 precursor protein-binding family B member 1-in | APBB1IP | 0.32 | 0.62 | -0.27 | 0.66 | -1.91 | 0.94 | -0.19 | 0.30 | -0.18 | -1.34 | 1.08 | 0.91 |
| Q99829 | Copine-1 | CPNE1 | 0.32 | 0.47 | -1.01 | 0.95 | -0.24 | -0.29 | -0.03 | 1.49 | -1.82 | -0.33 | 0.17 | 1.12 |
| P31150 | Rab GDP dissociation inhibitor alpha | GDI1 | 0.32 | 0.95 | -0.28 | -0.22 | 1.18 | 0.32 | -2.11 | -0.79 | -0.03 | 1.39 | 0.60 | -0.07 |
| P15104 | Glutamine synthetase | GLUL | 0.32 | 0.52 | -0.34 | -0.99 | 0.19 | -0.50 | 0.95 | -0.31 | -0.90 | 2.31 | -0.65 | 0.23 |
| Q14678 | KN motif and ankyrin repeat domain-containing protein 1 | KANK1 | 0.32 | 0.56 | 0.22 | -0.44 | 0.68 | -0.59 | -0.59 | 2.50 | -0.54 | -0.98 | -0.33 | 0.07 |
| Q8TD55 | Pleckstrin homology domain-containing family O member 2 | PLEKHO2 | 0.32 | 0.44 | -1.28 | 0.51 | -0.14 | 1.45 | -1.13 | -0.20 | -1.24 | 0.99 | 1.07 | -0.05 |
| Q01196 | Runt-related transcription factor 1 | RUNX1 | 0.32 | 0.29 | -0.25 | -0.41 | -0.55 | -0.40 | 1.22 | -0.39 | 2.40 | -0.72 | -0.49 | -0.40 |
| Q9UGJ0 | 5'-AMP-activated protein kinase subunit gamma-2 | PRKAG2 | 0.32 | 0.30 | -0.39 | 1.83 | -0.74 | -0.46 | -0.64 | 0.77 | -0.48 | -0.88 | -0.56 | 1.55 |
| P45983 | Mitogen-activated protein kinase 8 | MAPK8 | 0.32 | 0.38 | 0.95 | 0.37 | -2.34 | -0.18 | 0.68 | 0.73 | 0.95 | -0.26 | -0.24 | -0.66 |
| O75251 | NADH dehydrogenase [ubiquinone] iron-sulfur protein 7, mitochon | NDUFS7 | 0.32 | 0.32 | 0.13 | 0.52 | 1.32 | -1.21 | -1.19 | -0.10 | 0.02 | -1.41 | 0.60 | 1.32 |
| Q9BXT4 | Tudor domain-containing protein 1 | TDRD1 | 0.32 | 0.32 | -0.37 | -0.49 | -0.56 | 1.51 | -0.51 | -0.42 | -0.47 | 2.23 | -0.51 | -0.39 |
| P54727 | UV excision repair protein RAD23 homolog B | RAD23B | 0.32 | 0.71 | 1.11 | -0.33 | -1.39 | -0.77 | 0.51 | 1.54 | 0.48 | 0.73 | -0.87 | -1.00 |
| Q9H7D0 | Dedicator of cytokinesis protein 5 | DOCK5 | 0.32 | 0.32 | -0.84 | -0.61 | -1.14 | 1.10 | 1.06 | -0.97 | -1.05 | 0.42 | 0.97 | 1.07 |
| P12110 | Collagen alpha-2(VI) chain | COL6A2 | 0.32 | 0.59 | -1.48 | 1.05 | 1.47 | -0.43 | -1.38 | -0.11 | 0.88 | 0.13 | -0.64 | 0.49 |
| Q14315 | Filamin-C | FLNC | 0.32 | 0.40 | 1.01 | 0.61 | -0.17 | -0.50 | -1.48 | 1.07 | 1.26 | -1.47 | -0.48 | 0.15 |
| Q6NUP7 | Serine/threonine-protein phosphatase 4 regulatory subunit 4 | PPP4R4 | 0.32 | 0.35 | 1.30 | 0.00 | 0.11 | -0.37 | -1.52 | 0.74 | 1.03 | -1.74 | 0.46 | -0.01 |
| P60201 | Myelin proteolipid protein | PLP1 | 0.31 | 0.40 | -0.21 | -0.45 | 0.98 | -0.40 | -0.48 | -0.12 | -1.64 | 2.15 | -0.12 | 0.27 |
| Q14643 | Inositol 1,4,5-trisphosphate receptor type 1 | ITPR1 | 0.31 | 0.96 | -1.66 | -0.85 | 0.83 | 0.63 | -0.07 | -1.08 | 1.26 | -0.61 | 1.00 | 0.54 |
| P16403 | Histone H1.2 | HIST1H1C | 0.31 | 0.68 | 0.75 | -1.50 | 1.32 | 0.18 | -1.61 | 0.27 | -0.12 | 0.04 | -0.53 | 1.19 |
| Q9BZ67 | FERM domain-containing protein 8 | FRMD8 | 0.31 | 0.50 | 1.02 | -0.28 | -0.68 | -0.35 | -0.36 | 2.48 | -0.28 | -0.81 | -0.34 | -0.38 |
| O95931 | Chromobox protein homolog 7 | CBX7 | 0.31 | 0.50 | 1.01 | -0.35 | -0.61 | -0.36 | -0.35 | 2.45 | -0.41 | -0.95 | -0.36 | -0.07 |
| P31946 | 14-3-3 protein beta/alpha | YWHAB | 0.31 | 0.51 | -0.19 | 0.45 | -0.17 | -0.58 | -0.18 | 2.48 | 0.28 | -1.19 | -0.84 | -0.06 |
| P23458 | Tyrosine-protein kinase JAK1 | JAK1 | 0.31 | 0.36 | 1.28 | -1.48 | -0.32 | 0.19 | -0.15 | 1.08 | 1.13 | -1.59 | -0.15 | 0.01 |
| Q3ZCW2 | Galectin-related protein | LGALSL | 0.31 | 1.19 | -0.14 | -0.58 | -1.11 | 0.30 | 0.18 | -1.53 | 0.45 | 2.17 | 0.09 | 0.16 |
| P51858 | Hepatoma-derived growth factor | HDGF | 0.31 | 0.63 | -0.79 | -0.29 | 2.15 | -0.60 | -1.27 | -0.46 | -0.34 | 0.70 | -0.02 | 0.93 |
| P05204 | Non-histone chromosomal protein HMG-17 | HMGN2 | 0.31 | 0.80 | 0.25 | -0.27 | 0.92 | -0.17 | -1.68 | -0.87 | -0.67 | 1.78 | -0.16 | 0.87 |
| P00918 | Carbonic anhydrase 2 | CA2 | 0.31 | 1.42 | -0.70 | -1.02 | 1.48 | 0.52 | -1.82 | 0.07 | -0.49 | 0.84 | 0.80 | 0.32 |
| Q9NX46 | ADP-ribose glycohydrolase ARH3 | ADPRHL2 | 0.31 | 1.55 | 0.40 | -0.50 | -1.66 | -0.70 | 0.86 | 1.52 | -0.19 | 1.29 | -0.36 | -0.67 |
| P46782 | 40S ribosomal protein S5 | RPS5 | 0.31 | 0.50 | 0.01 | 0.37 | 1.01 | -0.53 | -1.51 | -0.50 | -1.31 | 1.74 | 0.17 | 0.54 |
| P01116 | GTPase KRas | KRAS | 0.31 | 0.38 | -0.28 | 1.14 | -2.12 | 0.67 | 0.09 | -0.90 | -0.65 | 0.63 | 0.81 | 0.62 |
| Q8IXH7 | Negative elongation factor C/D | NELFCD | 0.31 | 0.26 | 1.28 | 0.03 | -1.34 | -0.26 | -0.08 | 1.21 | 1.25 | -1.47 | -0.11 | -0.53 |
| P05198 | Eukaryotic translation initiation factor 2 subunit 1 | EIF2S1 | 0.31 | 0.89 | 0.46 | 0.41 | -0.01 | -0.48 | -1.44 | 0.06 | 2.26 | -1.06 | -0.24 | 0.04 |
| Q9HA64 | Ketosamine-3-kinase | FN3KRP | 0.31 | 1.33 | -0.30 | -0.87 | 0.61 | 0.97 | -1.83 | -0.46 | -0.37 | 0.33 | 1.73 | 0.21 |
| Q9Y4K3 | TNF receptor-associated factor 6 | TRAF6 | 0.31 | 0.30 | 0.00 | -0.48 | -1.97 | 1.34 | 0.71 | 0.09 | -0.42 | -0.86 | 1.24 | 0.35 |
| Q9Y5B9 | FACT complex subunit SPT16 | SUPT16H | 0.31 | 0.24 | 2.03 | -0.73 | -0.56 | -0.28 | -0.79 | 1.53 | -0.03 | -0.99 | -0.12 | -0.06 |
| Q9Y5P6 | Mannose-1-phosphate guanyltransferase beta | GMPPB | 0.30 | 1.10 | -0.89 | -0.01 | -0.74 | 1.55 | -1.15 | -0.32 | 1.47 | -0.64 | 1.08 | -0.35 |
| Q8TC12 | Retinol dehydrogenase 11 | RDH11 | 0.30 | 0.54 | 0.91 | 0.31 | -0.96 | 0.11 | -1.05 | 1.49 | 1.22 | -1.36 | 0.03 | -0.69 |
| Q03426 | Mevalonate kinase | MVK | 0.30 | 0.32 | 0.75 | -0.02 | -1.86 | 1.03 | -0.35 | 0.46 | -1.46 | -0.23 | 0.89 | 0.78 |
| Q13029 | PR domain zinc finger protein 2 | PRDM2 | 0.30 | 0.50 | 1.09 | -0.91 | -1.13 | 0.33 | -0.03 | -0.84 | 0.62 | -1.33 | 0.68 | 1.51 |
| O43581 | Synaptotagmin-7 | SYT7 | 0.30 | 0.93 | 0.37 | -0.15 | -1.60 | 0.80 | -0.51 | -1.56 | 0.84 | 0.27 | 1.48 | 0.06 |
| Q99836 | Myeloid differentiation primary response protein MyD88 | MYD88 | 0.30 | 0.89 | -0.23 | 0.06 | 0.58 | -0.68 | -0.80 | -1.00 | -0.97 | 1.76 | -0.27 | 1.55 |
| P30153 | Serine/threonine-protein phosphatase 2A 65 kDa regulatory subuni | PPP2R1A | 0.30 | 0.93 | -1.17 | 1.53 | -0.31 | 0.19 | -1.33 | 0.10 | 1.46 | -1.04 | 0.19 | 0.38 |
| P50914 | 60S ribosomal protein L14 | RPL14 | 0.30 | 1.08 | 0.97 | -0.39 | 0.27 | -0.54 | -1.54 | 0.82 | 0.73 | -1.43 | -0.27 | 1.37 |
| Q9UBB4 | Ataxin-10 | ATXN10 | 0.30 | 0.34 | -1.73 | 0.01 | 0.61 | 0.28 | 0.35 | -0.93 | -1.28 | 1.38 | 0.34 | 0.96 |
| Q5T8P6 | RNA-binding protein 26 | RBM26 | 0.30 | 0.33 | 1.06 | -0.21 | -1.20 | -0.98 | 0.89 | -0.04 | 1.77 | -0.51 | -1.07 | 0.31 |
| Q8N461 | F-box/LRR-repeat protein 16 | FBXL16 | 0.30 | 0.61 | 0.73 | -0.79 | -0.67 | 0.45 | -0.50 | 1.00 | 1.91 | -1.29 | -0.04 | -0.81 |
| Q9Y285 | Phenylalanine--tRNA ligase alpha subunit | FARSA | 0.30 | 0.50 | 0.93 | -1.98 | -0.88 | 0.76 | 0.53 | 0.01 | 0.76 | -1.19 | 0.47 | 0.59 |
| Q9BWM7 | Sideroflexin-3 | SFXN3 | 0.30 | 0.37 | -0.77 | 1.12 | 0.01 | 0.17 | -1.03 | -0.93 | 1.63 | -0.41 | -0.99 | 1.19 |
| O60832 | H/ACA ribonucleoprotein complex subunit DKC1 | DKC1 | 0.30 | 0.68 | -0.21 | 0.20 | 1.19 | 0.05 | -2.07 | -0.55 | 0.24 | 1.51 | -0.68 | 0.33 |
| P50583 | Bis(5'-nucleosyl)-tetraphosphatase [asymmetrical] | NUDT2 | 0.30 | 0.59 | 0.37 | 0.26 | -1.38 | 0.98 | -0.98 | 0.61 | -1.73 | 0.09 | 0.94 | 0.83 |
| P08319 | All-trans-retinol dehydrogenase [NAD(+)] ADH4 | ADH4 | 0.30 | 0.24 | -0.43 | -0.44 | 1.60 | -0.45 | -0.61 | -0.53 | -0.50 | 2.14 | -0.19 | -0.59 |
| O95081 | Arf-GAP domain and FG repeat-containing protein 2 | AGFG2 | 0.30 | 0.31 | 0.89 | 0.36 | -2.43 | 0.58 | 0.17 | -0.24 | -0.72 | -0.16 | 0.87 | 0.68 |
| P35637 | RNA-binding protein FUS | FUS | 0.30 | 1.10 | 0.61 | -1.62 | -1.87 | 0.76 | 0.86 | 0.34 | 0.89 | 0.37 | 0.06 | -0.39 |
| Q7Z4W1 | L-xylulose reductase | DCXR | 0.30 | 0.31 | -0.45 | -0.58 | 0.13 | 0.18 | 0.29 | -0.90 | -1.51 | 0.36 | 0.24 | 2.25 |
| Q9Y3E1 | Hepatoma-derived growth factor-related protein 3 | HDGFL3 | 0.30 | 0.62 | 1.04 | -0.15 | 1.06 | -0.95 | -1.78 | -0.07 | 0.41 | -0.64 | -0.35 | 1.43 |
| Q9Y6B6 | GTP-binding protein SAR1b | SAR1B | 0.30 | 0.31 | -0.50 | 0.52 | -1.65 | 0.71 | 0.51 | -0.49 | -1.41 | 0.05 | 0.87 | 1.41 |
| P39019 | 40S ribosomal protein S19 | RPS19 | 0.30 | 0.29 | 0.41 | -0.33 | -1.29 | 0.49 | 0.32 | 0.56 | -2.14 | 1.23 | 0.45 | 0.30 |
| Q9HCE5 | N6-adenosine-methyltransferase non-catalytic subunit | METTL14 | 0.30 | 0.39 | 1.27 | -1.25 | -1.44 | 0.44 | 0.45 | 0.32 | 1.15 | 0.21 | -1.35 | 0.19 |
| Q96D71 | RalBP1-associated Eps domain-containing protein 1 | REPS1 | 0.30 | 1.23 | 0.04 | 1.16 | -1.60 | -0.64 | -0.30 | -1.01 | 0.32 | 1.82 | -0.10 | 0.33 |
| Q02880 | DNA topoisomerase 2-beta | TOP2B | 0.29 | 0.33 | 1.92 | -0.77 | -0.56 | -0.81 | -0.24 | 1.36 | 0.87 | -0.59 | -0.75 | -0.43 |
| P62249 | 40S ribosomal protein S16 | RPS16 | 0.29 | 1.13 | -0.55 | -1.86 | 1.61 | -0.30 | -0.17 | 0.59 | -0.41 | -0.49 | 0.30 | 1.30 |
| Q4G0N4 | NAD kinase 2, mitochondrial | NADK2 | 0.29 | 1.86 | -0.65 | -1.39 | 1.17 | 0.20 | -1.15 | 1.05 | -0.03 | -1.05 | 0.82 | 1.02 |
| Q9H3P7 | Golgi resident protein GCP60 | ACBD3 | 0.29 | 0.29 | 0.58 | 0.11 | -1.40 | 0.23 | 0.08 | -0.22 | 2.06 | -1.52 | -0.09 | 0.18 |
| Q99459 | Cell division cycle 5-like protein | CDC5L | 0.29 | 0.72 | 1.12 | -0.65 | -0.36 | 0.14 | -1.14 | 1.07 | -1.43 | 1.15 | 0.88 | -0.78 |
| P09960 | Leukotriene A-4 hydrolase | LTA4H | 0.29 | 3.04 | 0.61 | -1.79 | -0.81 | 0.64 | -1.17 | -0.59 | 0.81 | 1.15 | 0.61 | 0.55 |
| Q96HS1 | Serine/threonine-protein phosphatase PGAM5, mitochondrial | PGAM5 | 0.29 | 0.66 | 1.22 | -0.75 | -1.10 | -0.26 | 0.06 | 0.90 | 1.55 | -1.57 | -0.05 | -0.01 |
| Q12830 | Nucleosome-remodeling factor subunit BPTF | BPTF | 0.29 | 0.27 | 1.56 | -1.12 | -1.29 | -0.11 | 0.59 | 0.62 | 1.17 | -1.06 | 0.23 | -0.59 |
| Q9NSK0 | Kinesin light chain 4 | KLC4 | 0.29 | 0.75 | -1.28 | 0.94 | -1.76 | 0.70 | 0.47 | -0.73 | -0.26 | 0.79 | 1.20 | -0.09 |
| Q9NPJ3 | Acyl-coenzyme A thioesterase 13 | ACOT13 | 0.29 | 0.89 | -0.34 | -0.68 | 1.72 | -0.26 | -1.48 | 1.01 | 0.47 | -1.23 | 0.18 | 0.62 |
| O94919 | Endonuclease domain-containing 1 protein | ENDOD1 | 0.29 | 0.64 | 0.36 | -2.57 | 0.26 | 0.87 | 0.26 | 0.47 | -0.65 | 0.16 | 0.85 | -0.01 |
| P22694 | cAMP-dependent protein kinase catalytic subunit beta | PRKACB | 0.29 | 0.89 | 1.20 | -0.29 | -0.55 | 0.26 | -1.66 | 1.28 | 0.69 | -1.39 | -0.07 | 0.52 |
| Q8IYD1 | Eukaryotic peptide chain release factor GTP-binding subunit ERF3I | GSPT2 | 0.29 | 0.86 | 0.75 | 0.49 | -1.20 | 0.18 | -1.23 | 0.61 | 1.31 | -1.66 | 0.19 | 0.57 |

| | | | | | | | | | | | | | | |
|---|---|---|---|---|---|---|---|---|---|---|---|---|---|---|
| P55010 | Eukaryotic translation initiation factor 5 | EIF5 | 0.29 | 0.52 | 0.23 | 0.97 | -0.94 | 0.33 | -1.27 | 1.09 | 0.79 | -1.84 | 0.27 | 0.36 |
| P62633 | Cellular nucleic acid-binding protein | CNBP | 0.29 | 0.40 | 1.07 | 0.98 | -0.18 | -0.72 | -1.69 | 0.78 | 1.23 | -0.49 | -1.06 | 0.08 |
| P24386 | Rab proteins geranylgeranyltransferase component A 1 | CHM | 0.29 | 0.32 | -1.01 | 0.75 | 0.48 | 0.55 | -1.19 | -1.12 | -1.24 | 0.95 | 0.68 | 1.16 |
| P19623 | Spermidine synthase | SRM | 0.29 | 0.78 | 1.95 | -1.23 | -0.49 | -0.29 | -0.89 | 1.49 | 0.22 | -0.49 | -0.05 | -0.23 |
| Q96II8 | DISP complex protein LRCH3 | LRCH3 | 0.28 | 0.30 | -0.45 | 1.83 | -0.69 | -0.53 | -0.56 | 0.25 | -0.64 | -0.90 | 1.76 | -0.07 |
| P04004 | Vitronectin | VTN | 0.28 | 0.21 | -0.67 | 0.23 | 1.60 | -0.75 | -0.71 | -0.74 | -0.76 | 1.09 | -0.71 | 1.42 |
| Q7Z2Z2 | Elongation factor-like GTPase 1 | EFL1 | 0.28 | 1.24 | 0.14 | -1.09 | -0.25 | -0.69 | 0.51 | -1.01 | 0.83 | 0.89 | -1.13 | 1.80 |
| O00159 | Unconventional myosin-Ic | MYO1C | 0.28 | 0.57 | 0.77 | -0.05 | -0.94 | 0.11 | -0.63 | 1.57 | -1.89 | -0.14 | 0.16 | 1.02 |
| P55265 | Double-stranded RNA-specific adenosine deaminase | ADAR | 0.28 | 0.29 | 1.86 | -0.67 | -1.13 | -0.02 | -0.43 | 1.66 | -0.39 | -0.86 | -0.04 | 0.04 |
| Q2M3C7 | A-kinase anchor protein SPHKAP | SPHKAP | 0.28 | 0.26 | 0.56 | -1.05 | 0.24 | 0.96 | -1.07 | -0.89 | -0.33 | 1.63 | 0.95 | -0.99 |
| P49418 | Amphiphysin | AMPH | 0.28 | 0.45 | -2.44 | 0.43 | 0.14 | 0.39 | 0.89 | -0.87 | 0.18 | 1.02 | 0.26 | 0.01 |
| P46821 | Microtubule-associated protein 1B | MAP1B | 0.28 | 0.54 | 1.01 | -0.60 | 0.35 | 0.05 | -1.50 | -0.17 | 1.97 | -1.15 | -0.04 | 0.07 |
| Q96JI7 | Spatacsin | SPG11 | 0.28 | 0.38 | 1.11 | -0.41 | -1.38 | 0.53 | -0.35 | 0.41 | 0.29 | -1.76 | 0.20 | 1.36 |
| P19338 | Nucleolin | NCL | 0.28 | 0.47 | -1.04 | 0.24 | 0.16 | 0.41 | -0.38 | -0.14 | -2.15 | 1.04 | 0.81 | 1.07 |
| Q6ZNW5 | GDP-D-glucose phosphorylase 1 | GDPGP1 | 0.28 | 0.31 | -0.56 | 1.73 | -0.95 | 0.03 | -0.67 | 0.61 | 1.61 | -0.97 | -0.21 | -0.62 |
| O00629 | Importin subunit alpha-3 | KPNA4 | 0.28 | 0.64 | -0.65 | 1.01 | -0.98 | 0.63 | -0.81 | -0.73 | 0.58 | -1.22 | 0.42 | 1.74 |
| Q9BSW7 | Synaptotagmin-17 | SYT17 | 0.28 | 0.46 | -1.04 | 1.54 | -1.21 | -0.62 | 0.72 | -0.25 | 0.08 | 1.62 | -0.69 | -0.16 |
| Q76NI1 | Kinase non-catalytic C-lobe domain-containing protein 1 | KNDC1 | 0.28 | 0.44 | 1.01 | -0.09 | -0.89 | 0.19 | -0.80 | -0.02 | 2.26 | -1.13 | -0.23 | -0.30 |
| P84243 | Histone H3.3 | H3F3A | 0.28 | 0.41 | 0.08 | -1.79 | 1.86 | 0.09 | -0.80 | 0.57 | -0.99 | 0.18 | 0.21 | 0.58 |
| O76041 | Nebulette | NEBL | 0.28 | 0.67 | 0.68 | 0.65 | -2.27 | 0.30 | -0.20 | 1.09 | -0.19 | -1.00 | 0.17 | 0.76 |
| Q9NTX5 | Ethylmalonyl-CoA decarboxylase | ECHDC1 | 0.28 | 0.42 | -0.44 | -1.66 | -0.27 | 0.78 | 1.02 | -0.89 | -1.04 | 1.21 | 0.95 | 0.33 |
| P02746 | Complement C1q subcomponent subunit B | C1QB | 0.28 | 1.64 | -1.20 | 0.20 | 0.30 | -0.40 | -0.60 | 0.27 | -0.36 | -0.81 | 0.14 | 2.45 |
| Q9BW85 | Splicing factor YJU2 | YJU2 | 0.28 | 0.27 | 1.24 | -1.26 | -1.45 | 0.52 | 0.58 | 1.08 | -1.30 | 0.25 | 0.48 | -0.14 |
| Q7Z4K8 | Tripartite motif-containing protein 46 | TRIM46 | 0.28 | 0.51 | 1.04 | -0.88 | -1.82 | 0.76 | 0.24 | 0.82 | 0.80 | -1.30 | -0.08 | 0.43 |
| P10909 | Clusterin | CLU | 0.28 | 0.32 | 1.76 | 0.26 | -0.81 | -0.84 | -0.80 | 0.94 | 1.32 | -0.65 | -0.79 | -0.38 |
| Q7Z333 | Probable helicase senataxin | SETX | 0.28 | 0.24 | 1.07 | 0.68 | 0.00 | -1.01 | -1.08 | 1.54 | -0.36 | -1.25 | -0.53 | 0.93 |
| P31939 | Bifunctional purine biosynthesis protein PURH | ATIC | 0.28 | 0.93 | 1.08 | -1.84 | -0.34 | 0.73 | -0.72 | 0.31 | -1.13 | 0.48 | 1.29 | 0.14 |
| P20340 | Ras-related protein Rab-6A | RAB6A | 0.28 | 0.31 | 0.28 | -1.03 | 1.93 | -0.43 | -1.18 | -0.20 | 0.40 | 1.30 | -0.35 | -0.72 |
| Q15651 | High mobility group nucleosome-binding domain-containing protei | HMGN3 | 0.28 | 0.37 | 1.09 | -0.16 | 1.14 | -0.87 | -1.70 | 0.64 | -0.83 | 0.88 | -0.79 | 0.60 |
| Q9BRJ7 | Tudor-interacting repair regulator protein | NUDT16L1 | 0.28 | 0.24 | -1.18 | -0.30 | 0.26 | 0.70 | 0.18 | -1.30 | -1.42 | 1.30 | 0.92 | 0.82 |
| Q9UHI6 | Probable ATP-dependent RNA helicase DDX20 | DDX20 | 0.28 | 0.24 | 1.51 | -0.83 | -1.02 | -0.42 | 0.42 | 0.43 | 1.46 | -1.13 | -0.90 | 0.48 |
| O95757 | Heat shock 70 kDa protein 4L | HSPA4L | 0.28 | 0.47 | 0.66 | 0.77 | -1.29 | 0.18 | -0.92 | 1.20 | 0.71 | -1.78 | -0.06 | 0.55 |
| Q7Z699 | Sprouty-related, EVH1 domain-containing protein 1 | SPRED1 | 0.28 | 0.45 | -0.33 | 1.34 | -0.90 | -0.05 | -0.65 | -0.46 | 2.10 | -0.91 | 0.38 | -0.52 |
| Q92619 | Rho GTPase-activating protein 45 | ARHGAP45 | 0.28 | 0.26 | 1.50 | -0.15 | -0.74 | -0.28 | -0.69 | 2.06 | -0.54 | -0.86 | 0.27 | -0.56 |
| P48739 | Phosphatidylinositol transfer protein beta isoform | PITPNB | 0.27 | 0.39 | 1.32 | -0.55 | -1.00 | 0.46 | -0.74 | 1.62 | -0.28 | 0.29 | 0.38 | -1.50 |
| P51970 | NADH dehydrogenase [ubiquinone] 1 alpha subcomplex subunit 8 | NDUFA8 | 0.27 | 0.49 | 1.29 | -0.39 | -1.64 | 0.71 | -0.60 | 1.34 | -0.53 | -0.61 | -0.52 | 0.95 |
| P21333 | Filamin-A | FLNA | 0.27 | 0.32 | 1.79 | -0.33 | -0.71 | -0.77 | -0.41 | 1.63 | 0.39 | -0.23 | -1.24 | -0.13 |
| O43526 | Potassium voltage-gated channel subfamily KQT member 2 | KCNQ2 | 0.27 | 0.93 | 0.00 | 1.44 | -0.30 | -0.41 | -1.82 | -0.83 | 1.54 | 0.43 | 0.23 | -0.27 |
| P12004 | Proliferating cell nuclear antigen | PCNA | 0.27 | 0.39 | -0.55 | -0.53 | 0.95 | -0.50 | 0.10 | -0.43 | -1.50 | 1.30 | -0.53 | 1.68 |
| Q16773 | Kynurenine--oxoglutarate transaminase 1 | KYAT1 | 0.27 | 0.26 | 0.63 | 0.45 | -1.17 | 0.44 | -0.70 | 1.18 | -2.09 | 0.55 | 0.38 | 0.35 |
| Q92522 | Histone H1x | H1FX | 0.27 | 0.60 | -0.59 | -0.70 | 0.82 | 0.67 | -0.96 | -0.92 | -1.13 | 0.06 | 1.04 | 1.71 |
| Q99435 | Protein kinase C-binding protein NELL2 | NELL2 | 0.27 | 0.41 | -0.94 | -0.19 | 0.50 | 0.82 | -0.72 | -0.14 | -1.74 | 1.76 | 0.70 | -0.04 |
| P60983 | Glia maturation factor beta | GMFB | 0.27 | 1.41 | 0.73 | 0.64 | -2.28 | -0.44 | -0.15 | 0.63 | 0.93 | 0.90 | -0.74 | -0.21 |
| O75326 | Semaphorin-7A | SEMA7A | 0.27 | 1.18 | 1.59 | -0.88 | -0.56 | -0.14 | -1.33 | 0.30 | -0.52 | 1.62 | -0.60 | 0.51 |
| O75369 | Filamin-B | FLNB | 0.27 | 1.13 | 0.48 | -0.18 | 0.12 | -0.16 | -1.54 | 0.05 | 2.18 | -0.30 | -1.12 | 0.48 |
| P34949 | Mannose-6-phosphate isomerase | MPI | 0.27 | 0.55 | -0.51 | -0.18 | 1.12 | 0.68 | -1.80 | 1.57 | 0.16 | 0.44 | -0.74 | -0.72 |
| O14807 | Ras-related protein M-Ras | MRAS | 0.27 | 1.02 | -0.25 | 0.03 | -0.64 | 0.53 | -0.85 | 0.49 | -0.01 | -1.84 | 0.72 | 1.83 |
| P09913 | Interferon-induced protein with tetratricopeptide repeats 2 | IFIT2 | 0.27 | 0.28 | -0.16 | 2.06 | -1.37 | -0.24 | -0.68 | 0.99 | -0.68 | -0.51 | -0.17 | 0.76 |
| Q9H6R3 | Acyl-CoA synthetase short-chain family member 3, mitochondrial | ACSS3 | 0.27 | 0.35 | 0.03 | -1.21 | 1.20 | 0.24 | -0.73 | 1.25 | -0.55 | -1.63 | 0.63 | 0.77 |
| Q5H9R4 | Armadillo repeat-containing X-linked protein 4 | ARMCX4 | 0.27 | 1.01 | 0.25 | -0.22 | -0.86 | -0.22 | -0.15 | -0.02 | 2.64 | -0.99 | -0.32 | -0.13 |
| Q96Q42 | Alsin | ALS2 | 0.27 | 0.45 | -0.96 | 0.28 | 1.16 | -1.91 | 0.83 | -0.52 | 0.99 | -0.31 | -0.45 | 0.88 |
| Q8WXX5 | DnaJ homolog subfamily C member 9 | DNAJC9 | 0.26 | 0.29 | -0.41 | 1.89 | -0.77 | -0.59 | -0.53 | -0.54 | -0.59 | -0.81 | 1.25 | 1.08 |
| Q9BVG4 | Protein PBDC1 | PBDC1 | 0.26 | 0.46 | -0.36 | -0.37 | -0.82 | -0.40 | 1.34 | 2.11 | -0.40 | -1.03 | -0.50 | 0.42 |
| Q86UR5 | Regulating synaptic membrane exocytosis protein 1 | RIMS1 | 0.26 | 2.00 | 1.14 | -1.74 | -1.05 | -0.08 | -0.19 | 0.29 | -0.56 | 1.74 | 0.18 | 0.26 |
| Q6N069 | N-alpha-acetyltransferase 16, NatA auxiliary subunit | NAA16 | 0.26 | 0.81 | 0.04 | -0.18 | -0.42 | -0.15 | -0.30 | 2.67 | -0.32 | -1.20 | -0.05 | -0.09 |
| Q9ULV4 | Coronin-1C | CORO1C | 0.26 | 0.72 | -1.56 | -0.58 | 0.62 | -0.01 | 0.65 | -0.67 | -1.08 | 1.87 | 0.42 | 0.34 |
| Q15018 | BRISC complex subunit Abraxas 2 | ABRAXAS2 | 0.26 | 0.49 | -1.40 | 1.07 | 0.86 | 0.81 | -1.99 | -0.09 | 0.36 | 0.28 | 0.45 | -0.34 |
| O14841 | 5-oxoprolinase | OPLAH | 0.26 | 0.35 | 1.26 | -0.77 | -0.91 | -0.72 | 0.67 | 1.26 | -0.24 | -1.51 | -0.08 | 1.05 |
| Q9BW62 | Katanin p60 ATPase-containing subunit A-like 1 | KATNAL1 | 0.26 | 0.38 | 0.60 | 0.76 | 0.22 | 0.39 | -2.47 | -0.16 | 0.73 | -0.76 | 0.77 | -0.07 |
| P18433 | Receptor-type tyrosine-protein phosphatase alpha | PTPRA | 0.26 | 0.46 | -1.97 | 0.94 | -0.01 | 1.09 | -0.66 | -0.40 | -0.71 | 0.18 | 1.36 | 0.18 |
| P36873 | Serine/threonine-protein phosphatase PP1-gamma catalytic subuni | PPP1CC | 0.26 | 1.19 | -0.72 | 0.11 | -1.63 | 1.24 | -0.32 | 1.21 | -0.10 | -1.09 | 1.22 | 0.07 |
| O00330 | Pyruvate dehydrogenase protein X component, mitochondrial | PDHX | 0.26 | 0.59 | -0.50 | -0.33 | 0.10 | 0.58 | -0.62 | -1.55 | -1.14 | 1.46 | 0.78 | 1.21 |
| O60641 | Clathrin coat assembly protein AP180 | SNAP91 | 0.26 | 0.48 | -0.90 | 0.41 | -0.85 | 0.12 | 0.59 | -1.65 | 0.53 | 1.94 | 0.19 | -0.37 |
| Q92610 | Zinc finger protein 592 | ZNF592 | 0.26 | 0.24 | -0.28 | -0.53 | -0.61 | -0.46 | 1.53 | -0.44 | -0.36 | -0.67 | 2.19 | -0.38 |
| Q9BY11 | Protein kinase C and casein kinase substrate in neurons protein 1 | PACSIN1 | 0.26 | 0.40 | -1.24 | -0.51 | 0.14 | -0.01 | 1.09 | -1.42 | 0.35 | 1.95 | -0.14 | -0.19 |
| O95674 | Phosphatidate cytidylyltransferase 2 | CDS2 | 0.26 | 0.20 | -0.41 | 0.61 | -0.61 | 0.64 | -0.52 | -0.41 | 2.48 | -0.76 | -0.53 | -0.49 |
| Q08380 | Galectin-3-binding protein | LGALS3BP | 0.26 | 0.43 | 0.03 | -1.44 | 0.15 | 0.46 | 0.24 | -0.45 | -1.50 | 1.84 | 0.88 | -0.20 |
| P25786 | Proteasome subunit alpha type-1 | PSMA1 | 0.26 | 0.47 | -0.95 | -1.36 | 0.39 | 0.04 | 1.27 | -0.66 | -0.71 | 1.83 | 0.38 | -0.23 |
| O00160 | Unconventional myosin-If | MYO1F | 0.26 | 0.36 | -2.00 | 0.95 | -0.07 | 0.75 | -0.12 | 0.39 | -0.93 | -0.80 | 0.57 | 1.25 |
| P52597 | Heterogeneous nuclear ribonucleoprotein F | HNRNPF | 0.26 | 0.43 | 0.55 | -1.38 | -1.43 | 0.83 | 0.86 | 0.70 | -1.24 | -0.39 | 0.80 | 0.71 |
| Q13126 | S-methyl-5'-thioadenosine phosphorylase | MTAP | 0.26 | 0.20 | 0.04 | -0.38 | 0.89 | -1.48 | 0.64 | -1.11 | -1.07 | 1.44 | 0.99 | 0.04 |
| Q7Z6Z7 | E3 ubiquitin-protein ligase HUWE1 | HUWE1 | 0.25 | 0.40 | -0.49 | -1.69 | 1.11 | -0.29 | 0.82 | -0.26 | -0.08 | 1.75 | 0.00 | -0.87 |
| P49721 | Proteasome subunit beta type-2 | PSMB2 | 0.25 | 0.68 | -0.84 | -1.72 | 0.74 | -0.34 | 1.32 | -0.27 | -0.48 | 1.61 | 0.17 | -0.18 |
| Q92769 | Histone deacetylase 2 | HDAC2 | 0.25 | 1.35 | 1.44 | -1.12 | -1.61 | 0.03 | -0.19 | 0.65 | -0.82 | 0.03 | 0.23 | 1.36 |
| Q5SW79 | Centrosomal protein of 170 kDa | CEP170 | 0.25 | 0.56 | 1.33 | -0.11 | 0.25 | 0.00 | -2.18 | 0.98 | 0.28 | 0.71 | -0.42 | -0.83 |
| Q99715 | Collagen alpha-1(XII) chain | COL12A1 | 0.25 | 0.17 | 0.92 | -0.35 | 0.69 | -1.34 | -0.16 | 0.95 | 1.31 | -1.57 | -0.75 | 0.30 |
| P48443 | Retinoic acid receptor RXR-gamma | RXRG | 0.25 | 0.50 | -0.12 | -0.86 | 2.02 | -0.78 | -0.91 | 0.46 | -0.34 | 1.34 | -0.03 | -0.77 |
| P17028 | Zinc finger protein 24 | ZNF24 | 0.25 | 0.55 | 0.44 | 0.45 | -1.68 | 0.30 | -0.21 | 1.68 | 0.61 | -1.57 | 0.06 | -0.09 |
| P26232 | Catenin alpha-2 | CTNNA2 | 0.25 | 0.26 | -2.53 | 0.57 | 0.71 | 0.48 | 0.40 | -0.72 | 0.60 | -0.38 | 0.40 | 0.46 |
| P05164 | Myeloperoxidase | MPO | 0.25 | 0.32 | 0.16 | -1.25 | -1.44 | 0.91 | 1.18 | -0.95 | -0.33 | 1.43 | 0.07 | 0.21 |
| Q7Z3E5 | LisH domain-containing protein ARMC9 | ARMC9 | 0.25 | 0.20 | -0.96 | -0.28 | -1.18 | 0.83 | 1.32 | -1.01 | -1.05 | 1.02 | 0.91 | 0.41 |
| Q9HCM2 | Plexin-A4 | PLXNA4 | 0.25 | 0.31 | 0.06 | 1.44 | -0.04 | 0.21 | -2.10 | 1.43 | -0.33 | -0.33 | 0.11 | -0.46 |
| P28288 | ATP-binding cassette sub-family D member 3 | ABCD3 | 0.25 | 0.39 | -0.88 | 0.01 | 0.41 | -0.21 | 0.14 | 0.91 | -1.05 | -1.57 | 0.39 | 1.86 |

| | | | | | | | | | | | | | | |
|---|---|---|---|---|---|---|---|---|---|---|---|---|---|---|
| Q9BQI9 | Nuclear receptor-interacting protein 2 | NRIP2 | 0.25 | 0.30 | -0.95 | -1.12 | 1.09 | 0.42 | 0.15 | -1.05 | -1.05 | 1.59 | 0.87 | 0.05 |
| Q6NYC1 | Bifunctional arginine demethylase and lysyl-hydroxylase JMJD6 | JMJD6 | 0.25 | 0.46 | 0.34 | -0.94 | -1.44 | 0.35 | 1.08 | 0.38 | -1.29 | -0.67 | 0.98 | 1.19 |
| Q03518 | Antigen peptide transporter 1 | TAP1 | 0.25 | 0.35 | -0.26 | 1.19 | -0.63 | -0.39 | -0.38 | -0.29 | -0.34 | -0.82 | -0.47 | 2.40 |
| P63096 | Guanine nucleotide-binding protein G(i) subunit alpha-1 | GNAI1 | 0.25 | 0.40 | -0.12 | 1.78 | -2.16 | 0.47 | -0.51 | -0.17 | 0.53 | -0.29 | 0.55 | -0.07 |
| Q9ULA0 | Aspartyl aminopeptidase | DNPEP | 0.25 | 0.38 | 0.96 | -1.65 | 0.64 | -0.65 | 0.20 | 1.72 | 0.33 | 0.01 | -0.47 | -1.09 |
| Q8IUX7 | Adipocyte enhancer-binding protein 1 | AEBP1 | 0.25 | 0.23 | 1.70 | -0.32 | 0.33 | -0.55 | -1.49 | 1.31 | 0.11 | -0.72 | -0.92 | 0.55 |
| P02730 | Band 3 anion transport protein | SLC4A1 | 0.24 | 0.16 | 1.52 | -0.11 | -1.00 | -0.95 | 0.30 | 1.51 | 0.55 | -1.12 | -0.90 | 0.19 |
| Q96FC7 | Phytanoyl-CoA hydroxylase-interacting protein-like | PHYHIPL | 0.24 | 0.63 | 0.66 | -1.25 | -0.33 | 0.51 | -0.39 | 1.66 | -0.77 | -1.48 | 0.70 | 0.68 |
| P27815 | cAMP-specific 3',5'-cyclic phosphodiesterase 4A | PDE4A | 0.24 | 0.22 | 0.31 | 0.19 | 0.48 | 0.82 | -2.11 | -1.21 | -0.66 | 0.51 | 0.80 | 0.86 |
| P00338 | L-lactate dehydrogenase A chain | LDHA | 0.24 | 0.65 | -0.08 | -0.01 | -0.48 | 0.60 | -0.85 | -0.56 | -0.28 | 2.37 | 0.48 | -1.19 |
| Q9UG63 | ATP-binding cassette sub-family F member 2 | ABCF2 | 0.24 | 0.70 | 1.13 | -1.99 | -1.07 | 0.67 | 0.39 | 0.61 | -0.51 | -0.62 | 0.86 | 0.51 |
| P50453 | Serpin B9 | SERPINB9 | 0.24 | 0.21 | -0.05 | -1.13 | 0.85 | -0.06 | 0.08 | -1.17 | -0.34 | 1.26 | -1.09 | 1.65 |
| O43823 | A-kinase anchor protein 8 | AKAP8 | 0.24 | 0.34 | 0.97 | -2.01 | -0.38 | 0.03 | 0.92 | 1.43 | -0.68 | -0.60 | 0.38 | -0.07 |
| Q9UQE7 | Structural maintenance of chromosomes protein 3 | SMC3 | 0.24 | 0.32 | 1.32 | 0.17 | -0.19 | -0.25 | -1.48 | 1.30 | 1.06 | -1.33 | 0.00 | -0.60 |
| Q00978 | Interferon regulatory factor 9 | IRF9 | 0.24 | 0.19 | 0.38 | -0.72 | -0.74 | -0.66 | 1.47 | -0.67 | 0.75 | -0.94 | 1.72 | -0.59 |
| P06400 | Retinoblastoma-associated protein | RB1 | 0.24 | 0.27 | 1.25 | -0.32 | -1.42 | 0.05 | 0.07 | 0.82 | 1.28 | 0.58 | -1.14 | -1.17 |
| Q8N3R9 | MAGUK p55 subfamily member 5 | MPP5 | 0.24 | 0.26 | 0.70 | 0.64 | -1.46 | -0.17 | -0.07 | -1.20 | -1.30 | 0.74 | 1.04 | 1.08 |
| Q9HAV4 | Exportin-5 | XPO5 | 0.23 | 0.30 | -0.28 | -0.33 | -0.55 | 1.17 | -0.43 | -0.41 | -0.37 | -0.78 | -0.44 | 2.42 |
| Q14331 | Protein FRG1 | FRG1 | 0.23 | 0.28 | 0.81 | -0.28 | 0.34 | 0.59 | -1.85 | 0.17 | -1.68 | 0.19 | 0.71 | 1.00 |
| Q8N1N4 | Keratin, type II cytoskeletal 78 | KRT78 | 0.23 | 0.44 | -0.57 | -0.15 | 0.85 | -0.78 | 0.07 | -0.54 | -0.15 | 2.42 | -1.07 | -0.07 |
| Q7L5N7 | Lysophosphatidylcholine acyltransferase 2 | LPCAT2 | 0.23 | 0.43 | 1.34 | 0.13 | -0.86 | -1.91 | 0.73 | 1.34 | -0.10 | -0.39 | -0.51 | 0.23 |
| Q5T5C0 | Syntaxin-binding protein 5 | STXBP5 | 0.23 | 0.46 | -0.40 | 1.41 | -0.71 | 0.18 | -1.09 | 0.76 | 1.18 | -1.75 | 0.13 | 0.28 |
| P61081 | NEDD8-conjugating enzyme Ubc12 | UBE2M | 0.23 | 0.81 | 0.48 | -0.43 | -1.17 | 0.67 | -0.53 | -0.45 | -0.55 | -0.83 | 0.60 | 2.22 |
| Q9Y2H5 | Pleckstrin homology domain-containing family A member 6 | PLEKHA6 | 0.23 | 0.28 | 1.65 | -0.28 | -0.32 | 0.02 | -1.45 | 1.34 | 0.82 | -1.15 | -0.33 | -0.29 |
| P99999 | Cytochrome c | CYCS | 0.23 | 0.34 | -0.86 | -0.39 | -0.36 | -0.55 | 1.69 | -1.30 | 0.04 | 1.77 | -0.05 | 0.01 |
| P53004 | Biliverdin reductase A | BLVRA | 0.23 | 1.33 | 0.37 | -1.99 | -1.26 | 0.72 | 0.71 | -0.47 | -0.03 | -0.05 | 1.20 | 0.80 |
| P42229 | Signal transducer and activator of transcription 5A | STAT5A | 0.23 | 0.31 | -0.54 | 0.47 | -0.86 | 1.28 | -0.77 | -0.58 | -0.67 | -1.02 | 1.25 | 1.45 |
| Q92783 | Signal transducing adapter molecule 1 | STAM | 0.23 | 0.66 | -1.21 | 2.01 | -0.11 | -0.43 | -1.09 | 0.31 | 1.00 | -0.91 | 0.02 | 0.41 |
| P63241 | Eukaryotic translation initiation factor 5A-1 | EIF5A | 0.23 | 0.92 | -0.73 | -1.52 | -0.26 | 0.72 | 0.71 | 1.26 | -0.59 | -1.26 | 1.09 | 0.58 |
| Q99962 | Endophilin-A1 | SH3GL2 | 0.23 | 0.75 | -1.40 | 0.53 | 0.29 | -0.21 | -0.13 | -0.57 | 2.45 | -0.33 | -0.34 | -0.28 |
| A1Z1Q3 | ADP-ribose glycohydrolase MACROD2 | MACROD2 | 0.23 | 0.27 | -1.94 | 0.61 | 0.78 | -0.76 | 0.93 | -0.72 | -0.07 | 0.47 | -0.61 | 1.30 |
| Q13043 | Serine/threonine-protein kinase 4 | STK4 | 0.23 | 0.33 | 0.97 | 0.74 | -1.10 | 0.25 | -1.30 | 1.31 | -0.19 | -1.54 | 0.26 | 0.60 |
| Q9P227 | Rho GTPase-activating protein 23 | ARHGAP23 | 0.23 | 0.24 | 0.04 | 1.28 | 0.21 | 0.56 | -2.43 | -0.46 | -0.46 | 0.11 | 0.66 | 0.49 |
| O95996 | Adenomatous polyposis coli protein 2 | APC2 | 0.23 | 0.37 | -1.54 | 0.42 | 0.64 | -0.14 | 0.12 | -1.91 | -0.01 | 0.83 | 0.32 | 1.26 |
| Q8TDJ6 | DmX-like protein 2 | DMXL2 | 0.23 | 0.25 | 1.38 | 0.39 | -1.89 | 0.03 | -0.25 | 1.27 | 0.52 | -1.19 | -0.17 | -0.08 |
| O95140 | Mitofusin-2 | MFN2 | 0.22 | 0.43 | 1.49 | -1.38 | -0.41 | 0.16 | -0.42 | -0.26 | 1.33 | -1.47 | 0.32 | 0.65 |
| O95573 | Long-chain-fatty-acid--CoA ligase 3 | ACSL3 | 0.22 | 0.36 | -0.15 | 1.26 | -0.36 | 0.38 | -1.61 | 0.80 | 0.65 | -1.72 | 0.05 | 0.70 |
| P40926 | Malate dehydrogenase, mitochondrial | MDH2 | 0.22 | 0.38 | 1.03 | -0.36 | 0.15 | -0.75 | -0.57 | 0.33 | 0.48 | 1.91 | -0.62 | -1.59 |
| Q13190 | Syntaxin-5 | STX5 | 0.22 | 0.40 | 0.63 | 0.67 | -0.25 | 0.84 | -2.42 | -0.66 | -0.14 | -0.13 | 0.69 | 0.78 |
| Q14123 | Calcium/calmodulin-dependent 3',5'-cyclic nucleotide phosphodiest | PDE1C | 0.22 | 0.21 | 1.22 | 0.02 | -0.60 | 0.55 | -1.48 | 0.96 | 1.05 | -1.41 | 0.39 | -0.71 |
| Q9UPP5 | AP2-interacting clathrin-endocytosis protein | KIAA1107 | 0.22 | 0.60 | 0.27 | 0.27 | -1.71 | 0.40 | 0.01 | 0.78 | 0.62 | -1.97 | 0.61 | 0.73 |
| Q71UI9 | Histone H2A.V | H2AFV | 0.22 | 0.31 | 0.38 | -1.96 | 0.80 | 0.20 | 0.16 | 0.27 | -1.68 | 0.87 | 0.81 | 0.15 |
| P04075 | Fructose-bisphosphate aldolase A | ALDOA | 0.22 | 0.37 | -0.37 | 0.00 | 0.28 | -0.90 | 0.49 | 0.01 | -0.63 | 2.52 | -0.65 | -0.75 |
| Q76G19 | PDZ domain-containing protein 4 | PDZD4 | 0.22 | 0.22 | -1.55 | 0.48 | 0.40 | 0.44 | -0.08 | -1.29 | 1.86 | -0.78 | 0.52 | 0.00 |
| Q9UQD0 | Sodium channel protein type 8 subunit alpha | SCN8A | 0.22 | 0.17 | 1.72 | 0.16 | -0.88 | -0.35 | -0.90 | 0.90 | 1.38 | -1.04 | -0.43 | -0.57 |
| Q9Y3B4 | Splicing factor 3B subunit 6 | SF3B6 | 0.22 | 0.22 | -1.00 | 0.89 | 0.46 | 0.47 | -1.13 | -1.19 | -1.19 | 0.68 | 0.67 | 1.33 |
| P09104 | Gamma-enolase | ENO2 | 0.22 | 0.30 | -0.21 | 0.21 | 0.21 | -0.99 | 0.37 | -0.08 | -0.61 | 2.52 | -0.58 | -0.84 |
| Q9UJW0 | Dynactin subunit 4 | DCTN4 | 0.22 | 0.40 | 0.57 | -0.07 | -1.68 | 0.32 | 0.33 | 0.92 | 1.50 | -1.56 | 0.00 | -0.32 |
| Q00722 | 1-phosphatidylinositol 4,5-bisphosphate phosphodiesterase beta-2 | PLCB2 | 0.22 | 0.22 | 1.55 | -0.48 | -0.65 | -0.35 | -0.38 | 2.17 | -0.40 | -0.74 | -0.33 | -0.38 |
| Q9UKN8 | General transcription factor 3C polypeptide 4 | GTF3C4 | 0.22 | 0.43 | 1.26 | -0.64 | -1.15 | -0.60 | 0.56 | 0.63 | -0.75 | -1.36 | 1.25 | 0.80 |
| Q14697 | Neutral alpha-glucosidase AB | GANAB | 0.22 | 0.45 | -0.39 | -0.18 | 2.04 | -0.23 | -1.84 | 0.63 | 0.45 | -0.71 | 0.34 | -0.11 |
| Q6UVK1 | Chondroitin sulfate proteoglycan 4 | CSPG4 | 0.21 | 0.30 | -0.68 | -0.68 | 0.13 | 1.66 | -0.85 | -0.70 | -0.79 | -0.27 | 1.81 | 0.37 |
| Q15208 | Serine/threonine-protein kinase 38 | STK38 | 0.21 | 0.32 | -0.60 | -0.39 | -0.78 | 0.72 | 0.62 | -0.73 | -0.77 | -1.09 | 1.33 | 1.70 |
| P06733 | Alpha-enolase | ENO1 | 0.21 | 0.34 | -0.34 | -1.09 | 0.97 | -0.18 | 0.19 | -0.34 | -1.23 | 2.21 | 0.18 | -0.36 |
| Q9P2A4 | ABI gene family member 3 | ABI3 | 0.21 | 0.20 | 0.96 | 0.85 | -1.70 | 0.11 | -0.50 | 1.66 | -0.75 | -0.89 | 0.25 | 0.01 |
| Q9P258 | Protein RCC2 | RCC2 | 0.21 | 0.21 | 0.67 | -0.68 | 1.15 | -2.17 | 0.73 | -0.27 | -0.16 | 0.55 | -0.66 | 0.83 |
| P50749 | Ras association domain-containing protein 2 | RASSF2 | 0.21 | 0.24 | -1.09 | 0.30 | -1.72 | 1.08 | 1.08 | -0.54 | 0.57 | -0.96 | 0.87 | 0.40 |
| Q9P121 | Neurotrimin | NTM | 0.21 | 0.36 | 0.49 | 1.31 | 0.04 | -0.53 | -1.80 | -0.07 | -0.58 | 1.73 | -0.45 | -0.14 |
| Q9UL25 | Ras-related protein Rab-21 | RAB21 | 0.21 | 0.91 | -0.55 | 0.99 | 0.49 | 0.44 | -2.46 | 0.02 | -0.44 | 0.10 | 0.64 | 0.78 |
| Q9GZR7 | ATP-dependent RNA helicase DDX24 | DDX24 | 0.21 | 0.36 | -0.27 | 0.98 | -0.93 | -0.22 | -0.05 | -0.69 | -1.85 | 0.79 | 1.03 | 1.21 |
| Q6PCB7 | Long-chain fatty acid transport protein 1 | SLC27A1 | 0.21 | 0.34 | 0.69 | -1.22 | 0.16 | -0.62 | 0.54 | 1.05 | 1.01 | -2.00 | -0.03 | 0.43 |
| P06732 | Creatine kinase M-type | CKM | 0.21 | 0.16 | 0.76 | -0.65 | -0.71 | 0.98 | -0.62 | 0.21 | 2.14 | -0.84 | -0.65 | -0.62 |
| P46109 | Crk-like protein | CRKL | 0.21 | 0.91 | -0.65 | 0.77 | -0.74 | 0.51 | -0.96 | 0.81 | -0.77 | -1.40 | 1.04 | 1.40 |
| P68400 | Casein kinase II subunit alpha | CSNK2A1 | 0.21 | 0.51 | 0.65 | -0.44 | 0.00 | 1.07 | -1.94 | 0.63 | 0.08 | -1.41 | 0.94 | 0.42 |
| O14910 | Protein lin-7 homolog A | LIN7A | 0.21 | 0.44 | 0.41 | -0.07 | -1.31 | 0.18 | 0.20 | 1.08 | 1.48 | -1.86 | 0.28 | -0.38 |
| Q9Y4L1 | Hypoxia up-regulated protein 1 | HYOU1 | 0.21 | 0.23 | -1.65 | 0.50 | 0.73 | 0.56 | -0.47 | -0.79 | -0.72 | -0.56 | 0.65 | 1.75 |
| P23528 | Cofilin-1 | CFL1 | 0.21 | 1.52 | 0.65 | -1.56 | -1.40 | 0.67 | 0.06 | -0.17 | -0.72 | 0.93 | 1.53 | 0.00 |
| Q9NSC5 | Homer protein homolog 3 | HOMER3 | 0.21 | 0.27 | -0.54 | 0.77 | -1.06 | -0.70 | 1.16 | -0.67 | -0.76 | 1.15 | -0.81 | 1.47 |
| P29992 | Guanine nucleotide-binding protein subunit alpha-11 | GNA11 | 0.21 | 0.54 | -1.85 | -0.02 | 0.56 | 0.90 | -0.28 | -0.74 | -0.83 | -0.24 | 1.24 | 1.25 |
| Q9P0J1 | [Pyruvate dehydrogenase [acetyl-transferring]]-phosphatase 1, mi | PDP1 | 0.20 | 0.22 | 0.23 | -0.89 | 0.83 | 0.61 | -1.09 | 1.29 | 1.36 | -1.35 | -0.31 | -0.68 |
| Q92845 | Kinesin-associated protein 3 | KIFAP3 | 0.20 | 0.11 | 1.21 | -0.24 | -1.23 | 0.15 | -0.05 | 0.80 | 1.51 | -1.69 | -0.09 | -0.37 |
| O60346 | PH domain leucine-rich repeat-containing protein phosphatase 1 | PHLPP1 | 0.20 | 0.20 | -0.76 | -0.87 | 1.56 | 0.84 | -1.04 | -0.86 | 0.43 | 0.74 | 0.98 | -1.00 |
| O14879 | Interferon-induced protein with tetratricopeptide repeats 3 | IFIT3 | 0.20 | 0.24 | 0.85 | 1.29 | -1.19 | -0.07 | -1.22 | 0.79 | 0.77 | -1.56 | 0.20 | 0.13 |
| O94887 | FERM, ARHGEF and pleckstrin domain-containing protein 2 | FARP2 | 0.20 | 0.27 | 0.54 | -0.23 | -0.93 | 1.08 | -0.83 | -1.35 | -0.87 | 0.00 | 1.32 | 1.27 |
| O15126 | Secretory carrier-associated membrane protein 1 | SCAMP1 | 0.20 | 0.20 | 0.65 | 1.72 | -1.51 | -0.90 | -0.24 | 0.33 | 0.49 | -0.10 | -1.22 | 0.79 |
| Q92889 | DNA repair endonuclease XPF | ERCC4 | 0.20 | 0.23 | -0.40 | 1.97 | -0.71 | -0.51 | -0.67 | -0.54 | -0.64 | 0.33 | 1.65 | -0.47 |
| Q9GZN8 | UPF0687 protein C20orf27 | C20orf27 | 0.20 | 1.10 | -1.50 | -0.99 | 1.04 | -0.45 | 0.66 | -1.22 | -0.06 | 1.06 | 1.16 | 0.29 |
| P69905 | Hemoglobin subunit alpha | HBA1 | 0.20 | 0.57 | -0.29 | -1.34 | -0.26 | 0.76 | 0.40 | -1.84 | 0.33 | 1.58 | 0.58 | 0.08 |
| Q15813 | Tubulin-specific chaperone E | TBCE | 0.20 | 0.32 | -0.42 | 0.92 | -0.16 | 1.26 | -2.03 | -0.06 | -0.11 | -0.92 | 1.10 | 0.43 |
| Q9NXV6 | CDKN2A-interacting protein | CDKN2AIP | 0.20 | 0.26 | 0.95 | -0.40 | -1.09 | -0.78 | 0.96 | -0.03 | 1.59 | -1.18 | -0.82 | 0.79 |
| Q9NUU7 | ATP-dependent RNA helicase DDX19A | DDX19A | 0.20 | 0.56 | 0.64 | 0.95 | -1.14 | 0.33 | -1.49 | 1.58 | -0.36 | -1.09 | 0.10 | 0.49 |
| Q9UDT6 | CAP-Gly domain-containing linker protein 2 | CLIP2 | 0.20 | 0.12 | 1.53 | -0.12 | 0.26 | -0.78 | -1.07 | 0.72 | 1.51 | -1.33 | -0.41 | -0.30 |
| O95487 | Protein transport protein Sec24B | SEC24B | 0.20 | 0.43 | -0.05 | -0.78 | -0.80 | 0.30 | 0.74 | 0.60 | -2.21 | 0.75 | 0.36 | 1.08 |

| | | | | | | | | | | | | | | |
|---|---|---|---|---|---|---|---|---|---|---|---|---|---|---|
| Q15052 | Rho guanine nucleotide exchange factor 6 | ARHGEF6 | 0.20 | 0.50 | -0.06 | -0.04 | -0.44 | 0.17 | -0.29 | 0.59 | 1.83 | -2.21 | 0.19 | 0.27 |
| P31146 | Coronin-1A | CORO1A | 0.19 | 0.53 | -0.52 | 1.80 | -0.15 | -0.16 | -1.64 | -0.35 | 1.42 | -0.54 | -0.30 | 0.45 |
| Q9Y6K5 | 2'-5'-oligoadenylate synthase 3 | OAS3 | 0.19 | 0.25 | 1.31 | 0.71 | -0.31 | -0.10 | -1.95 | 0.72 | -0.64 | -0.14 | -0.75 | 1.16 |
| P01112 | GTPase HRas | HRAS | 0.19 | 0.52 | -0.54 | 1.06 | -0.12 | -0.42 | -0.66 | -1.44 | 2.20 | 0.13 | -0.02 | -0.20 |
| P60891 | Ribose-phosphate pyrophosphokinase 1 | PRPS1 | 0.19 | 0.56 | -0.64 | 0.52 | 0.45 | 0.30 | -1.35 | 0.12 | -0.38 | -0.21 | 2.22 | -1.03 |
| Q9Y4D1 | Disheveled-associated activator of morphogenesis 1 | DAAM1 | 0.19 | 0.20 | -1.23 | 1.30 | 0.75 | 0.39 | -1.49 | 0.29 | -1.39 | 0.10 | 0.52 | 0.76 |
| P50607 | Tubby protein homolog | TUB | 0.19 | 0.45 | 0.38 | -1.54 | -0.72 | 0.69 | 0.60 | -1.53 | 0.00 | 0.91 | -0.18 | 1.39 |
| Q9UQN3 | Charged multivesicular body protein 2b | CHMP2B | 0.19 | 0.37 | 1.47 | 0.10 | -0.02 | 0.12 | -2.18 | 0.15 | 0.94 | -1.03 | 0.26 | 0.18 |
| Q9Y4G6 | Talin-2 | TLN2 | 0.19 | 0.32 | -1.12 | 0.97 | -0.26 | 0.25 | -0.28 | -1.82 | 1.17 | 1.34 | 0.05 | -0.32 |
| Q8WYP5 | Protein ELYS | AHCTF1 | 0.19 | 0.31 | 0.92 | -1.01 | -0.88 | 0.83 | -0.28 | -0.35 | -0.68 | -1.16 | 1.00 | 1.63 |
| O43491 | Band 4.1-like protein 2 | EPB41L2 | 0.19 | 0.60 | -0.13 | -0.47 | 0.14 | 0.17 | -0.50 | 1.62 | 1.45 | -1.81 | 0.16 | -0.63 |
| P43003 | Excitatory amino acid transporter 1 | SLC1A3 | 0.19 | 0.42 | -1.11 | 0.04 | -0.79 | 0.26 | 1.04 | -1.15 | -0.95 | 1.82 | 0.25 | 0.60 |
| Q99497 | Protein/nucleic acid deglycase DJ-1 | PARK7 | 0.19 | 0.33 | -0.90 | -0.46 | 0.36 | -0.27 | 0.82 | -1.47 | -0.16 | 2.22 | -0.03 | -0.11 |
| O95251 | Histone acetyltransferase KAT7 | KAT7 | 0.19 | 0.18 | 0.83 | -0.09 | -1.69 | 0.33 | 0.37 | 1.00 | 0.70 | -1.90 | 0.32 | 0.14 |
| Q9P1A6 | Disks large-associated protein 2 | DLGAP2 | 0.19 | 0.10 | -0.27 | 0.48 | -0.16 | -0.38 | 0.18 | -0.45 | 2.54 | -1.28 | -0.35 | -0.31 |
| P23743 | Diacylglycerol kinase alpha | DGKA | 0.19 | 0.36 | -1.18 | 1.29 | -0.40 | -0.28 | 0.10 | -1.88 | 0.79 | -0.14 | 1.07 | 0.63 |
| Q96NW4 | Ankyrin repeat domain-containing protein 27 | ANKRD27 | 0.19 | 0.27 | 0.62 | 0.58 | -2.62 | 0.45 | 0.58 | 0.20 | -0.30 | -0.41 | 0.75 | 0.14 |
| P02790 | Hemopexin | HPX | 0.19 | 0.16 | 0.96 | 0.43 | 0.19 | -0.32 | -1.48 | -0.40 | 0.01 | 1.81 | -1.44 | 0.25 |
| P40429 | 60S ribosomal protein L13a | RPL13A | 0.19 | 0.27 | -0.60 | -1.58 | -0.66 | 0.29 | 2.17 | -0.68 | 0.03 | 0.21 | 0.21 | 0.62 |
| P00450 | Ceruloplasmin | CP | 0.19 | 0.33 | 1.30 | -0.86 | -0.03 | -1.14 | 0.28 | 1.28 | 1.10 | 0.20 | -1.04 | -1.10 |
| Q92620 | Pre-mRNA-splicing factor ATP-dependent RNA helicase PRP16 | DHX38 | 0.19 | 0.25 | 1.70 | -0.88 | -1.07 | 0.27 | -0.37 | 0.75 | -0.85 | -1.14 | 0.56 | 1.02 |
| P21926 | CD9 antigen | CD9 | 0.19 | 0.26 | -0.28 | -0.45 | 0.99 | 0.18 | -0.81 | -0.50 | -1.34 | 2.19 | 0.30 | -0.29 |
| P00488 | Coagulation factor XIII A chain | F13A1 | 0.19 | 0.29 | -0.80 | 0.46 | 1.48 | 0.46 | -1.99 | -0.83 | -0.43 | 0.60 | 0.57 | 0.49 |
| P35580 | Myosin-10 | MYH10 | 0.19 | 0.28 | -1.40 | -0.66 | 0.22 | 1.10 | 0.36 | -1.08 | -0.90 | 1.05 | 1.45 | -0.12 |
| Q15436 | Protein transport protein Sec23A | SEC23A | 0.18 | 0.32 | 1.35 | -0.22 | -1.53 | 0.31 | -0.35 | 0.56 | 1.39 | -1.53 | -0.02 | 0.04 |
| Q8N2F6 | Armadillo repeat-containing protein 10 | ARMC10 | 0.18 | 0.17 | 2.03 | -0.54 | -0.71 | -0.48 | -0.54 | 1.58 | -0.08 | -0.88 | -0.51 | 0.13 |
| O60333 | Kinesin-like protein KIF1B | KIF1B | 0.18 | 0.17 | 0.11 | 1.07 | -1.41 | 1.21 | -1.23 | 0.69 | 0.23 | -1.43 | 0.27 | 0.48 |
| P09382 | Galectin-1 | LGALS1 | 0.18 | 0.53 | -0.57 | -0.36 | 0.48 | 0.43 | -0.67 | 0.13 | -1.31 | 2.26 | 0.42 | -0.80 |
| Q9UNH7 | Sorting nexin-6 | SNX6 | 0.18 | 0.23 | -1.35 | 1.29 | -0.13 | 0.49 | -0.62 | 0.05 | -1.63 | 0.42 | 0.08 | 1.42 |
| Q9NPF4 | Probable tRNA N6-adenosine threonylcarbamoyltransferase | OSGEP | 0.18 | 0.32 | -1.02 | -0.09 | 1.18 | 0.23 | -0.74 | -1.64 | 0.27 | 1.72 | 0.37 | -0.29 |
| P35250 | Replication factor C subunit 2 | RFC2 | 0.18 | 0.19 | 0.53 | -0.71 | -1.42 | 0.90 | 0.43 | -1.15 | -1.18 | 0.50 | 1.26 | 0.82 |
| P84098 | 60S ribosomal protein L19 | RPL19 | 0.18 | 0.22 | -0.28 | -1.86 | 0.10 | -0.09 | 1.83 | 1.32 | -0.21 | -0.30 | -0.05 | -0.45 |
| Q9HC98 | Serine/threonine-protein kinase Nek6 | NEK6 | 0.18 | 0.32 | -0.16 | -0.22 | -0.20 | -0.10 | 0.24 | 1.48 | -0.16 | -2.33 | 0.47 | 0.98 |
| P11142 | Heat shock cognate 71 kDa protein | HSPA8 | 0.18 | 0.77 | -0.91 | 0.43 | 1.57 | -0.09 | -1.95 | -0.62 | 0.45 | 1.05 | -0.03 | 0.10 |
| P68133 | Actin, alpha skeletal muscle | ACTA1 | 0.18 | 0.51 | -0.18 | 1.19 | -0.87 | 0.29 | -1.09 | 0.44 | 0.92 | -1.85 | 0.13 | 1.02 |
| Q05682 | Caldesmon | CALD1 | 0.18 | 0.34 | 0.32 | 0.65 | 1.16 | -0.77 | -1.83 | 0.86 | -0.06 | -0.63 | -0.86 | 1.15 |
| Q8IZ07 | Ankyrin repeat domain-containing protein 13A | ANKRD13A | 0.18 | 0.20 | 0.55 | -0.98 | 0.94 | 0.19 | -0.99 | -0.97 | 0.26 | -1.38 | 1.43 | 0.94 |
| Q9Y3B2 | Exosome complex component CSL4 | EXOSC1 | 0.18 | 0.21 | 1.27 | -0.90 | -0.94 | 1.06 | -0.79 | 0.73 | -0.89 | -1.14 | 0.94 | 0.65 |
| Q04206 | Transcription factor p65 | RELA | 0.18 | 0.22 | -0.68 | 0.79 | -1.92 | 0.89 | 0.62 | 0.63 | -1.01 | -0.20 | 1.22 | -0.34 |
| Q96P53 | WD repeat and FYVE domain-containing protein 2 | WDFY2 | 0.18 | 0.15 | -1.65 | -0.21 | 0.61 | 0.60 | 0.43 | -0.57 | -1.65 | 0.92 | 1.11 | 0.42 |
| O00154 | Cytosolic acyl coenzyme A thioester hydrolase | ACOT7 | 0.18 | 0.44 | 0.74 | -1.45 | 0.39 | -0.18 | -0.09 | 0.14 | -0.49 | 2.08 | 0.08 | -1.24 |
| Q9Y6I3 | Epsin-1 | EPN1 | 0.18 | 0.41 | -1.68 | 1.37 | -0.40 | -0.15 | 0.31 | -1.03 | 1.56 | -0.43 | -0.15 | 0.60 |
| Q5TA50 | Ceramide-1-phosphate transfer protein | CPTP | 0.18 | 0.21 | -0.14 | 0.63 | 0.14 | 0.70 | -1.63 | 0.78 | 0.31 | -1.90 | 0.07 | 1.04 |
| O75380 | NADH dehydrogenase [ubiquinone] iron-sulfur protein 6, mitochon | NDUFS6 | 0.18 | 0.29 | -2.57 | 0.84 | 0.97 | 0.60 | -0.25 | 0.32 | 0.39 | -0.23 | 0.04 | -0.11 |
| Q9Y566 | SH3 and multiple ankyrin repeat domains protein 1 | SHANK1 | 0.18 | 0.22 | -0.11 | 1.20 | -0.63 | -0.44 | -0.34 | -0.43 | 2.38 | -0.80 | -0.46 | -0.37 |
| O14787 | Transportin-2 | TNPO2 | 0.18 | 0.21 | 0.76 | 0.69 | -1.53 | 0.78 | -1.00 | 0.66 | 0.12 | -1.68 | 0.55 | 0.64 |
| Q9NVI7 | ATPase family AAA domain-containing protein 3A | ATAD3A | 0.18 | 0.17 | 0.66 | -0.81 | 1.54 | -0.25 | -1.39 | 0.44 | 0.43 | -1.62 | 0.39 | 0.62 |
| Q9HAV0 | Guanine nucleotide-binding protein subunit beta-4 | GNB4 | 0.18 | 0.45 | -0.60 | 1.77 | -1.91 | 0.77 | -0.63 | 0.47 | -0.15 | -0.54 | 0.36 | 0.46 |
| Q9H9E3 | Conserved oligomeric Golgi complex subunit 4 | COG4 | 0.18 | 0.27 | -0.24 | -0.36 | -0.75 | -0.23 | 1.21 | -0.34 | -0.44 | -0.75 | -0.48 | 2.37 |
| P46777 | 60S ribosomal protein L5 | RPL5 | 0.18 | 0.51 | -0.89 | -1.48 | 2.13 | -0.73 | 0.30 | -0.35 | 0.69 | 0.47 | -0.16 | 0.02 |
| Q13322 | Growth factor receptor-bound protein 10 | GRB10 | 0.17 | 0.21 | -0.16 | -0.23 | -1.99 | 0.85 | 1.22 | 0.49 | 0.91 | -1.04 | 0.53 | -0.59 |
| Q96RL7 | Vacuolar protein sorting-associated protein 13A | VPS13A | 0.17 | 0.32 | 0.06 | 0.24 | -1.79 | 0.20 | 0.87 | -1.53 | 0.31 | -0.29 | 1.49 | 0.45 |
| Q12792 | Twinfilin-1 | TWF1 | 0.17 | 0.66 | -0.25 | -0.74 | 1.38 | 0.22 | -1.44 | 1.27 | 0.93 | -1.36 | 0.13 | -0.15 |
| Q13247 | Serine/arginine-rich splicing factor 6 | SRSF6 | 0.17 | 0.28 | -0.78 | 1.28 | 0.14 | -2.02 | 1.00 | -0.59 | 0.63 | 0.25 | -0.60 | 0.69 |
| Q9BXF6 | Rab11 family-interacting protein 5 | RAB11FIP5 | 0.17 | 0.32 | 0.50 | 0.68 | -2.32 | 0.80 | -0.09 | 0.79 | 0.25 | -1.19 | 0.23 | 0.36 |
| Q8IWR0 | Zinc finger CCCH domain-containing protein 7A | ZC3H7A | 0.17 | 0.33 | -0.35 | -0.21 | 0.22 | -0.32 | 0.20 | -1.40 | -0.10 | 2.55 | -0.23 | -0.35 |
| P60709 | Actin, cytoplasmic 1 | ACTB | 0.17 | 0.69 | -0.70 | 1.39 | -0.73 | 0.79 | -1.62 | 0.42 | 1.16 | -1.07 | 0.01 | 0.34 |
| O15372 | Eukaryotic translation initiation factor 3 subunit H | EIF3H | 0.17 | 0.56 | -0.98 | 1.19 | -0.42 | 0.41 | -0.92 | -0.80 | -1.34 | 1.23 | 0.50 | 1.11 |
| Q99590 | Protein SCAF11 | SCAF11 | 0.17 | 0.34 | -0.33 | -0.42 | -1.01 | 1.86 | -0.56 | -0.55 | -0.56 | 0.52 | 1.64 | -0.58 |
| Q9BRZ2 | E3 ubiquitin-protein ligase TRIM56 | TRIM56 | 0.17 | 0.17 | 0.32 | 0.03 | -1.88 | 0.24 | 1.04 | 0.53 | -1.75 | 0.14 | 0.60 | 0.72 |
| P14174 | Macrophage migration inhibitory factor | MIF | 0.17 | 0.31 | -0.56 | -0.08 | 1.29 | -0.27 | -0.80 | -1.08 | -0.22 | 2.23 | -0.19 | -0.30 |
| O43149 | Zinc finger ZZ-type and EF-hand domain-containing protein 1 | ZZEF1 | 0.17 | 0.31 | -1.47 | 0.41 | 0.15 | -0.47 | 0.95 | -1.72 | -0.36 | 1.08 | 0.33 | 1.09 |
| Q8TDB6 | E3 ubiquitin-protein ligase DTX3L | DTX3L | 0.17 | 0.21 | 1.37 | -0.62 | -0.93 | -0.07 | -0.05 | -0.65 | 2.20 | -0.19 | -0.42 | -0.63 |
| Q96GK7 | Fumarylacetoacetate hydrolase domain-containing protein 2A | FAHD2A | 0.17 | 0.15 | -1.61 | -0.30 | 1.38 | 0.15 | 0.16 | -1.64 | 0.28 | 1.23 | 0.28 | 0.07 |
| Q03154 | Aminoacylase-1 | ACY1 | 0.17 | 0.27 | -0.20 | -0.30 | 1.10 | -0.47 | -0.51 | -0.07 | -0.48 | 2.40 | -0.93 | -0.55 |
| Q9UHY1 | Nuclear receptor-binding protein | NRBP1 | 0.17 | 0.21 | -1.01 | -0.45 | 0.15 | 0.88 | 0.13 | -1.78 | -0.66 | 1.57 | 0.91 | 0.26 |
| Q08357 | Sodium-dependent phosphate transporter 2 | SLC20A2 | 0.17 | 0.26 | -0.21 | 1.64 | -0.72 | -0.48 | -0.59 | 0.25 | -0.51 | -0.88 | -0.45 | 1.96 |
| Q8N568 | Serine/threonine-protein kinase DCLK2 | DCLK2 | 0.17 | 0.22 | 0.77 | -0.30 | -1.07 | 0.36 | -0.05 | 0.31 | 0.00 | -1.84 | 1.89 | -0.06 |
| P07099 | Epoxide hydrolase 1 | EPHX1 | 0.16 | 0.53 | -0.85 | -0.37 | 1.48 | 0.63 | -1.58 | -1.01 | -0.48 | 0.95 | 0.86 | 0.37 |
| Q9UHD1 | Cysteine and histidine-rich domain-containing protein 1 | CHORDC1 | 0.16 | 1.01 | -1.81 | 0.35 | -1.43 | 1.26 | 0.45 | 0.25 | 0.49 | -0.39 | 1.11 | -0.28 |
| Q13144 | Translation initiation factor eIF-2B subunit epsilon | EIF2B5 | 0.16 | 0.62 | 0.06 | -0.20 | -0.30 | 0.63 | -0.97 | 0.91 | 0.36 | -2.05 | 0.03 | 1.54 |
| Q9HAS0 | Protein Njmu-R1 | C17orf75 | 0.16 | 0.16 | -0.56 | 0.70 | -0.87 | 1.21 | -0.70 | -0.58 | -0.75 | -1.07 | 1.27 | 1.36 |
| Q13023 | A-kinase anchor protein 6 | AKAP6 | 0.16 | 0.25 | -0.53 | 1.42 | -0.97 | 0.70 | -0.97 | -0.78 | 0.65 | -1.23 | 0.45 | 1.26 |
| P35908 | Keratin, type II cytoskeletal 2 epidermal | KRT2 | 0.16 | 0.22 | -0.71 | -1.45 | 1.38 | -0.19 | 0.66 | -0.85 | 0.59 | 1.52 | -0.16 | -0.78 |
| Q9BWH6 | RNA polymerase II-associated protein 1 | RPAP1 | 0.16 | 0.31 | 0.04 | -0.34 | 0.67 | 0.68 | -1.46 | -0.55 | -1.70 | 0.51 | 1.18 | 0.98 |
| O75937 | DnaJ homolog subfamily C member 8 | DNAJC8 | 0.16 | 0.34 | -1.99 | -0.48 | 1.00 | 0.63 | 0.38 | -0.62 | -0.54 | -0.53 | 0.97 | 1.18 |
| O95478 | Ribosome biogenesis protein NSA2 homolog | NSA2 | 0.16 | 0.14 | -0.62 | -0.73 | -0.96 | 0.07 | 2.04 | 1.26 | 0.30 | 0.23 | -0.86 | -0.71 |
| Q9NXG2 | THUMP domain-containing protein 1 | THUMPD1 | 0.16 | 0.21 | -0.61 | -0.01 | 1.22 | 0.02 | -0.92 | -0.87 | 0.95 | 1.83 | -0.87 | -0.74 |
| O00294 | Tubby-related protein 1 | TULP1 | 0.16 | 0.15 | 1.24 | -0.84 | 1.13 | -0.78 | -0.96 | 0.69 | 1.14 | -1.16 | -0.83 | 0.36 |
| Q15717 | ELAV-like protein 1 | ELAVL1 | 0.16 | 0.44 | 0.88 | 0.49 | -1.37 | 0.51 | -1.08 | 1.02 | -1.16 | -0.92 | 0.60 | 1.04 |
| P61225 | Ras-related protein Rap-2b | RAP2B | 0.16 | 0.09 | 1.08 | 0.55 | -1.02 | 0.22 | -0.96 | 1.30 | 1.24 | -1.15 | -0.94 | -0.33 |
| P53384 | Cytosolic Fe-S cluster assembly factor NUBP1 | NUBP1 | 0.16 | 0.23 | 0.62 | 1.38 | 0.02 | -1.37 | -0.97 | 0.65 | -1.39 | -0.48 | 0.55 | 1.00 |
| Q92859 | Neogenin | NEO1 | 0.15 | 0.27 | -0.13 | 0.80 | -0.85 | 0.38 | -0.57 | -0.56 | -0.38 | 2.46 | -0.62 | -0.51 |

| | | | | | | | | | | | | | | |
|---|---|---|---|---|---|---|---|---|---|---|---|---|---|---|
| Q92562 | Polyphosphoinositide phosphatase | FIG4 | 0.15 | 0.29 | 1.56 | 0.46 | -0.37 | -0.12 | -1.94 | 0.41 | 1.29 | -0.45 | -0.30 | -0.55 |
| Q9UKM9 | RNA-binding protein Raly | RALY | 0.15 | 0.13 | 0.56 | -2.17 | -0.24 | 0.91 | 0.75 | 0.09 | -1.26 | 0.10 | 0.90 | 0.35 |
| O75170 | Serine/threonine-protein phosphatase 6 regulatory subunit 2 | PPP6R2 | 0.15 | 0.20 | -1.25 | 1.72 | -0.77 | 0.64 | -0.61 | 0.51 | -1.34 | -0.09 | 1.00 | 0.20 |
| O75140 | GATOR complex protein DEPDC5 | DEPDC5 | 0.15 | 0.15 | -1.36 | -0.55 | 0.63 | 0.67 | 0.40 | -2.10 | 0.65 | 0.73 | 0.65 | 0.27 |
| Q15369 | Elongin-C | ELOC | 0.15 | 0.42 | 0.14 | 0.72 | -0.38 | 0.04 | -1.07 | 1.28 | 0.97 | -2.10 | -0.01 | 0.42 |
| Q9UK99 | F-box only protein 3 | FBXO3 | 0.15 | 0.18 | -0.34 | 1.88 | -0.78 | -0.48 | -0.53 | -0.48 | -0.56 | -1.01 | 1.08 | 1.23 |
| Q9UPN3 | Microtubule-actin cross-linking factor 1, isoforms 1/2/3/5 | MACF1 | 0.15 | 0.18 | -1.49 | -0.08 | 0.79 | 0.54 | -0.02 | -1.62 | 0.08 | 1.56 | 0.73 | -0.50 |
| Q9H9S4 | Calcium-binding protein 39-like | CAB39L | 0.15 | 0.16 | -0.50 | -0.68 | -0.84 | 0.19 | 1.61 | -0.67 | -0.71 | 1.86 | -0.63 | 0.37 |
| Q86YT6 | E3 ubiquitin-protein ligase MIB1 | MIB1 | 0.15 | 0.13 | 1.70 | 0.23 | -1.23 | -0.18 | -0.71 | 0.72 | 1.15 | -1.45 | -0.30 | 0.07 |
| O75886 | Signal transducing adapter molecule 2 | STAM2 | 0.15 | 0.19 | -0.16 | -0.15 | 0.06 | -0.08 | 0.04 | -2.56 | 1.02 | 0.76 | 0.86 | 0.21 |
| Q5VT25 | Serine/threonine-protein kinase MRCK alpha | CDC42BPA | 0.15 | 0.32 | 1.71 | 0.56 | -0.98 | -0.47 | -1.26 | 1.12 | 0.94 | -0.51 | -0.56 | -0.54 |
| Q7RTN6 | STE20-related kinase adapter protein alpha | STRADA | 0.15 | 0.37 | 0.91 | 0.41 | -0.06 | -0.02 | -1.73 | 0.11 | 2.00 | -0.82 | -0.44 | -0.35 |
| Q8IYU2 | E3 ubiquitin-protein ligase HACE1 | HACE1 | 0.15 | 0.22 | 0.07 | 1.38 | -0.90 | -0.32 | -0.53 | 1.38 | -0.13 | -1.79 | -0.03 | 0.88 |
| O75128 | Protein cordon-bleu | COBL | 0.15 | 0.15 | 0.27 | -0.18 | -1.99 | 1.06 | 0.62 | -0.41 | -1.07 | -0.30 | 0.90 | 1.10 |
| P42345 | Serine/threonine-protein kinase mTOR | MTOR | 0.15 | 0.22 | -1.14 | 0.48 | 0.14 | 0.87 | -0.67 | -1.03 | -1.38 | 1.47 | 1.03 | 0.22 |
| P46778 | 60S ribosomal protein L21 | RPL21 | 0.15 | 0.48 | -0.60 | -1.88 | -0.09 | 0.69 | 1.25 | 0.13 | 0.85 | -1.33 | 0.34 | 0.64 |
| P50990 | T-complex protein 1 subunit theta | CCT8 | 0.15 | 0.41 | 0.55 | 0.15 | 0.35 | 0.08 | -1.67 | 1.06 | 1.57 | -1.34 | -0.16 | -0.58 |
| O95292 | Vesicle-associated membrane protein-associated protein B/C | VAPB | 0.15 | 0.14 | -1.33 | 0.72 | 0.10 | 0.39 | -0.09 | -1.34 | -1.16 | 1.70 | 0.45 | 0.56 |
| O43707 | Alpha-actinin-4 | ACTN4 | 0.15 | 0.22 | -1.06 | -0.05 | 0.84 | -0.10 | 0.07 | -0.41 | -1.23 | 2.32 | -0.07 | -0.31 |
| Q8N335 | Glycerol-3-phosphate dehydrogenase 1-like protein | GPD1L | 0.15 | 0.12 | 0.21 | -1.10 | -0.88 | 0.78 | 0.82 | -0.24 | -0.71 | 1.68 | 0.77 | -1.32 |
| Q8WUX9 | Charged multivesicular body protein 7 | CHMP7 | 0.15 | 0.16 | -0.12 | 1.14 | -0.33 | 0.54 | -1.46 | 0.45 | 0.52 | -1.87 | 0.04 | 1.09 |
| Q92499 | ATP-dependent RNA helicase DDX1 | DDX1 | 0.15 | 0.28 | 0.94 | -0.29 | -1.74 | 1.04 | -0.35 | 0.61 | -1.03 | -0.85 | 1.20 | 0.46 |
| A0A087WW87 | Immunoglobulin kappa variable 2-40 | IGKV2-40 | 0.14 | 0.10 | 0.43 | 0.89 | 0.47 | -1.78 | -0.15 | 0.64 | -0.09 | 0.74 | -1.80 | 0.64 |
| Q13813 | Spectrin alpha chain, non-erythrocytic 1 | SPTAN1 | 0.14 | 0.62 | 0.83 | 0.16 | -0.10 | -1.44 | -0.24 | 0.36 | 1.61 | 0.98 | -1.33 | -0.83 |
| P05166 | Propionyl-CoA carboxylase beta chain, mitochondrial | PCCB | 0.14 | 0.24 | -0.97 | -0.99 | 1.92 | -1.23 | 0.92 | -0.20 | -0.27 | 0.49 | -0.41 | 0.72 |
| Q9Y4D7 | Plexin-D1 | PLXND1 | 0.14 | 0.08 | -1.20 | 1.47 | -1.33 | 0.70 | 0.24 | -0.58 | -1.22 | 0.50 | 0.62 | 0.80 |
| Q9NSI8 | SAM domain-containing protein SAMSN-1 | SAMSN1 | 0.14 | 0.18 | -0.01 | 1.66 | -1.04 | -0.02 | -0.84 | 0.84 | -0.79 | -1.25 | 0.22 | 1.23 |
| Q8N9R8 | Protein SCAI | SCAI | 0.14 | 0.15 | -0.12 | 1.50 | -0.19 | -0.52 | -0.89 | -0.65 | 1.65 | -1.46 | 0.08 | 0.61 |
| Q9BPY3 | Protein FAM118B | FAM118B | 0.14 | 0.20 | -0.16 | 0.32 | -1.74 | 0.69 | 0.62 | 1.23 | -1.65 | 0.19 | -0.29 | 0.80 |
| P30039 | Phenazine biosynthesis-like domain-containing protein | PBLD | 0.14 | 0.10 | 1.81 | -0.82 | 0.57 | -0.87 | -0.84 | 0.85 | -0.82 | -1.07 | 0.49 | 0.69 |
| Q13042 | Cell division cycle protein 16 homolog | CDC16 | 0.14 | 1.42 | -1.27 | -1.43 | -0.59 | 1.34 | 0.45 | 0.36 | -0.85 | 1.32 | 0.06 | 0.63 |
| P26358 | DNA (cytosine-5)-methyltransferase 1 | DNMT1 | 0.14 | 0.21 | -0.83 | -0.75 | -0.43 | 0.16 | 1.56 | -0.57 | -1.44 | 1.45 | 0.09 | 0.77 |
| P46937 | Transcriptional coactivator YAP1 | YAP1 | 0.14 | 0.10 | -1.15 | 1.00 | -1.38 | 0.82 | 0.57 | -1.26 | -0.15 | -0.42 | 0.88 | 1.09 |
| Q9UKK3 | Protein mono-ADP-ribosyltransferase PARP4 | PARP4 | 0.14 | 0.32 | 2.15 | -0.77 | -0.15 | -0.01 | -1.67 | 0.57 | -0.65 | 0.00 | 0.02 | 0.50 |
| Q96FN4 | Copine-2 | CPNE2 | 0.14 | 0.11 | -0.03 | -0.17 | -0.35 | 0.59 | -0.21 | 1.16 | -2.51 | 0.65 | 0.48 | 0.39 |
| Q9P1Z2 | Calcium-binding and coiled-coil domain-containing protein 1 | CALCOCO1 | 0.14 | 0.14 | 0.33 | 1.34 | -0.58 | 0.08 | -1.37 | 1.03 | 0.80 | -1.78 | 0.11 | 0.04 |
| P29966 | Myristoylated alanine-rich C-kinase substrate | MARCKS | 0.14 | 0.31 | -0.57 | -0.33 | 1.85 | -1.41 | 0.04 | 0.65 | 0.43 | 0.35 | -1.48 | 0.47 |
| O00571 | ATP-dependent RNA helicase DDX3X | DDX3X | 0.14 | 0.75 | 0.87 | 0.10 | -0.20 | -0.22 | -1.47 | 1.48 | -0.28 | -1.36 | -0.26 | 1.34 |
| Q6UWR7 | Ectonucleotide pyrophosphatase/phosphodiesterase family membe | ENPP6 | 0.14 | 0.14 | -0.03 | -0.01 | 0.15 | 0.41 | -0.72 | 0.75 | -2.34 | 1.25 | 0.80 | -0.25 |
| Q5VWX1 | KH domain-containing, RNA-binding, signal transduction-associate | KHDRBS2 | 0.14 | 0.24 | 1.27 | -0.28 | -0.71 | -0.28 | -0.33 | 2.32 | -0.43 | -0.92 | -0.29 | -0.35 |
| Q9BQI7 | PH and SEC7 domain-containing protein 2 | PSD2 | 0.14 | 0.27 | -1.72 | 0.89 | 0.16 | 0.71 | -0.40 | 0.19 | -0.58 | -1.37 | 0.93 | 1.20 |
| P02794 | Ferritin heavy chain | FTH1 | 0.14 | 0.29 | -0.96 | -0.89 | -0.81 | 0.05 | 2.20 | -0.88 | 0.10 | 0.88 | 0.45 | -0.14 |
| Q02218 | 2-oxoglutarate dehydrogenase, mitochondrial | OGDH | 0.14 | 0.21 | 0.14 | -0.18 | 1.61 | -1.03 | -0.83 | 0.45 | 1.75 | -0.84 | -0.70 | -0.37 |
| P52788 | Spermine synthase | SMS | 0.14 | 0.27 | -1.78 | 0.15 | 0.69 | 0.35 | 0.21 | 1.08 | -1.65 | -0.46 | 0.52 | 0.89 |
| Q9C0C9 | (E3-independent) E2 ubiquitin-conjugating enzyme | UBE2O | 0.14 | 0.37 | -0.08 | 1.92 | -1.56 | 0.28 | -1.06 | -0.01 | 0.76 | 0.64 | -0.07 | -0.81 |
| P34897 | Serine hydroxymethyltransferase, mitochondrial | SHMT2 | 0.14 | 0.11 | -0.83 | 0.16 | 0.78 | 0.62 | -0.90 | -0.80 | -0.86 | 2.01 | -0.82 | 0.64 |
| P11047 | Laminin subunit gamma-1 | LAMC1 | 0.14 | 0.41 | -0.80 | 0.13 | 0.98 | -0.16 | -0.70 | -0.90 | -0.80 | 2.26 | 0.40 | -0.41 |
| P48047 | ATP synthase subunit O, mitochondrial | ATP5PO | 0.14 | 0.21 | 1.69 | -0.60 | -0.22 | -0.52 | -0.64 | 1.29 | 1.24 | -1.05 | -0.73 | -0.46 |
| O95219 | Sorting nexin-4 | SNX4 | 0.14 | 0.18 | 1.22 | 1.32 | -0.19 | -0.92 | -1.69 | -0.04 | 0.86 | -0.92 | -0.25 | 0.61 |
| Q9BTD8 | RNA-binding protein 42 | RBM42 | 0.13 | 0.13 | 0.11 | -0.26 | -0.35 | 0.35 | -0.04 | -0.10 | -2.39 | 1.48 | 0.67 | 0.53 |
| Q53FT3 | Protein Hikeshi | HIKESHI | 0.13 | 0.12 | 1.01 | -1.07 | -1.28 | 0.46 | 0.71 | 1.04 | 0.67 | 0.69 | -1.12 | -1.10 |
| Q8ND71 | GTPase IMAP family member 8 | GIMAP8 | 0.13 | 0.16 | -0.87 | 0.84 | -1.92 | 0.99 | 0.73 | -0.85 | -0.60 | 0.66 | 0.89 | 0.13 |
| Q13572 | Inositol-tetrakisphosphate 1-kinase | ITPK1 | 0.13 | 0.26 | -2.07 | 0.61 | -0.26 | 0.53 | 0.82 | 0.14 | -1.46 | 0.17 | 0.84 | 0.66 |
| O96028 | Histone-lysine N-methyltransferase NSD2 | NSD2 | 0.13 | 0.16 | -0.21 | -0.43 | -0.68 | 1.53 | -0.45 | -0.37 | 2.17 | -0.72 | -0.48 | -0.37 |
| Q7L9L4 | MOB kinase activator 1B | MOB1B | 0.13 | 0.39 | -0.30 | -1.39 | 1.75 | 0.08 | -0.67 | 0.11 | -1.17 | 1.44 | 0.08 | 0.06 |
| Q9NXC5 | GATOR complex protein MIOS | MIOS | 0.13 | 0.14 | -0.53 | -0.45 | -0.66 | 0.86 | 0.57 | -1.23 | -1.41 | 1.62 | 0.88 | 0.34 |
| Q5JR12 | Protein phosphatase 1J | PPM1J | 0.13 | 0.27 | -0.11 | 1.68 | -0.74 | -0.55 | -0.65 | 1.36 | -0.20 | -1.21 | -0.63 | 1.06 |
| Q9BSQ5 | Cerebral cavernous malformations 2 protein | CCM2 | 0.13 | 0.17 | 0.32 | -0.52 | 0.60 | 0.91 | -1.56 | -1.76 | -0.27 | 0.55 | 0.75 | 0.99 |
| Q969Q0 | 60S ribosomal protein L36a-like | RPL36AL | 0.13 | 0.54 | -0.55 | -0.86 | 1.03 | -0.26 | -0.05 | -0.23 | 1.67 | -1.45 | -0.56 | 1.26 |
| Q6Y7W6 | GRB10-interacting GYF protein 2 | GIGYF2 | 0.13 | 0.09 | 0.30 | -0.13 | -1.51 | 0.43 | 0.78 | 0.83 | 1.41 | -1.58 | 0.24 | -0.77 |
| O43464 | Serine protease HTRA2, mitochondrial | HTRA2 | 0.13 | 0.31 | -0.27 | -0.44 | 1.92 | -1.47 | -0.15 | 0.31 | -1.33 | 0.68 | 0.01 | 0.75 |
| Q12972 | Nuclear inhibitor of protein phosphatase 1 | PPP1R8 | 0.13 | 0.10 | 1.02 | -1.29 | -1.37 | 0.78 | 0.71 | 0.30 | 0.41 | -1.55 | 0.79 | 0.20 |
| Q5JTJ3 | Cytochrome c oxidase assembly factor 6 homolog | COA6 | 0.13 | 0.12 | -1.06 | 1.05 | -0.05 | -1.09 | 0.98 | -1.18 | -1.09 | 0.70 | 0.79 | 0.96 |
| Q9Y4I1 | Unconventional myosin-Va | MYO5A | 0.13 | 0.11 | 1.38 | 0.17 | -0.99 | -0.27 | -0.45 | 1.09 | 1.49 | -1.33 | -0.40 | -0.68 |
| Q13546 | Receptor-interacting serine/threonine-protein kinase 1 | RIPK1 | 0.13 | 0.14 | 0.09 | 1.61 | -0.64 | -0.45 | -0.82 | 1.64 | -0.05 | -1.22 | -0.76 | 0.59 |
| P54619 | 5'-AMP-activated protein kinase subunit gamma-1 | PRKAG1 | 0.13 | 0.11 | 0.46 | 0.29 | -1.87 | 0.47 | 0.48 | -1.68 | -0.33 | 0.30 | 0.89 | 0.97 |
| Q969E8 | Pre-rRNA-processing protein TSR2 homolog | TSR2 | 0.13 | 0.14 | 1.16 | 0.43 | -1.03 | 0.75 | -1.52 | 0.15 | 0.34 | -1.59 | 0.64 | 0.66 |
| Q9NZ01 | Very-long-chain enoyl-CoA reductase | TECR | 0.13 | 0.17 | -1.45 | -0.22 | -0.56 | 0.91 | 1.08 | -0.95 | -0.87 | -0.30 | 1.07 | 1.29 |
| P52756 | RNA-binding protein 5 | RBM5 | 0.13 | 0.16 | 0.90 | 0.19 | -2.54 | 0.15 | 1.07 | -0.11 | 0.18 | -0.49 | 0.46 | 0.19 |
| Q9H3Q1 | Cdc42 effector protein 4 | CDC42EP4 | 0.12 | 0.20 | 0.46 | 1.22 | -1.05 | -0.77 | -0.14 | 1.48 | -1.05 | -1.27 | 0.39 | 0.73 |
| P30040 | Endoplasmic reticulum resident protein 29 | ERP29 | 0.12 | 0.15 | 0.21 | -0.54 | 0.68 | -0.09 | -0.49 | -1.38 | -1.21 | 1.98 | 0.03 | 0.80 |
| Q86SK9 | Stearoyl-CoA desaturase 5 | SCD5 | 0.12 | 0.14 | -0.80 | -0.79 | 0.34 | 1.27 | -0.22 | -0.07 | -0.91 | -1.37 | 1.26 | 1.30 |
| Q92828 | Coronin-2A | CORO2A | 0.12 | 0.24 | 0.34 | 1.52 | -0.29 | 0.47 | -2.37 | 0.36 | 0.39 | -0.47 | 0.31 | -0.27 |
| P40616 | ADP-ribosylation factor-like protein 1 | ARL1 | 0.12 | 0.17 | 0.15 | -2.10 | 0.70 | 0.09 | 0.93 | -1.17 | -0.60 | 0.77 | 0.38 | 0.86 |
| P63151 | Serine/threonine-protein phosphatase 2A 55 kDa regulatory subuni | PPP2R2A | 0.12 | 0.34 | -1.11 | -1.38 | -0.40 | 1.39 | 1.03 | -0.76 | -0.34 | -0.32 | 1.22 | 0.65 |
| Q96C19 | EF-hand domain-containing protein D2 | EFHD2 | 0.12 | 0.28 | 1.11 | -0.79 | -0.07 | -1.52 | 0.89 | 1.04 | 0.84 | 0.34 | -1.40 | -0.44 |
| P05109 | Protein S100-A8 | S100A8 | 0.12 | 0.08 | -1.05 | 0.85 | -1.26 | 0.55 | 0.80 | -1.17 | -1.10 | 1.14 | 0.55 | 0.69 |
| Q9Y6R0 | Numb-like protein | NUMBL | 0.12 | 0.08 | 1.21 | 0.12 | -1.50 | -0.15 | 0.20 | 0.66 | 1.49 | -1.56 | -0.19 | -0.28 |
| O00764 | Pyridoxal kinase | PDXK | 0.12 | 0.30 | -0.03 | 1.24 | -1.23 | 0.78 | -1.17 | 0.93 | -0.09 | -1.55 | 0.36 | 0.75 |
| Q01814 | Plasma membrane calcium-transporting ATPase 2 | ATP2B2 | 0.12 | 0.16 | 1.05 | 0.17 | -1.36 | -0.61 | 0.52 | 1.70 | 0.78 | -0.95 | -0.40 | -0.91 |
| Q15424 | Scaffold attachment factor B1 | SAFB | 0.12 | 0.18 | 0.75 | -0.12 | 0.01 | -0.20 | -0.69 | 1.70 | -1.20 | -1.08 | -0.61 | 1.43 |
| P61964 | WD repeat-containing protein 5 | WDR5 | 0.12 | 0.11 | 1.72 | -0.30 | -1.11 | -0.02 | -0.45 | 1.64 | -0.85 | -0.99 | 0.03 | 0.34 |
| Q9Y4W6 | AFG3-like protein 2 | AFG3L2 | 0.12 | 0.09 | -1.59 | 0.91 | 0.94 | 0.49 | -0.89 | -1.31 | -0.61 | 0.42 | 0.58 | 1.05 |

| | | | | | | | | | | | | | | |
|---|---|---|---|---|---|---|---|---|---|---|---|---|---|---|
| P26196 | Probable ATP-dependent RNA helicase DDX6 | DDX6 | 0.11 | 0.54 | -1.12 | -1.71 | 1.36 | 0.59 | 0.17 | -0.31 | 0.00 | -0.47 | -0.04 | 1.51 |
| P51674 | Neuronal membrane glycoprotein M6-a | GPM6A | 0.11 | 0.19 | 0.09 | 1.02 | -2.06 | 0.71 | -0.04 | -0.84 | -0.46 | 1.13 | 0.90 | -0.45 |
| Q8WVT3 | Trafficking protein particle complex subunit 12 | TRAPPC12 | 0.11 | 0.13 | -0.42 | 2.04 | -0.72 | -0.56 | -0.54 | -0.45 | -0.57 | -0.87 | 0.82 | 1.25 |
| P60033 | CD81 antigen | CD81 | 0.11 | 0.31 | -0.13 | 0.99 | 0.17 | 0.21 | -1.66 | -0.61 | -1.08 | 1.84 | 0.46 | -0.19 |
| Q14683 | Structural maintenance of chromosomes protein 1A | SMC1A | 0.11 | 0.21 | -0.18 | -0.59 | -0.67 | 1.76 | -0.62 | -0.55 | -0.55 | -1.01 | 1.47 | 0.94 |
| P61163 | Alpha-centractin | ACTR1A | 0.11 | 0.23 | 0.40 | -0.68 | 0.48 | 0.62 | -1.14 | 0.89 | -2.18 | 0.55 | 0.47 | 0.60 |
| O75110 | Probable phospholipid-transporting ATPase IIA | ATP9A | 0.11 | 0.13 | 0.57 | 0.34 | 0.01 | 0.70 | -1.82 | 1.07 | -0.19 | -1.73 | 0.66 | 0.39 |
| O95486 | Protein transport protein Sec24A | SEC24A | 0.11 | 0.12 | 0.29 | 0.21 | -1.05 | 0.68 | -0.30 | 0.27 | -0.21 | -2.08 | 0.69 | 1.51 |
| P09417 | Dihydropteridine reductase | QDPR | 0.11 | 0.20 | -0.52 | -1.10 | 1.23 | 0.70 | -0.59 | -1.07 | -0.06 | 1.62 | 0.72 | -0.93 |
| Q96HN2 | Adenosylhomocysteinase 3 | AHCYL2 | 0.11 | 0.37 | 0.32 | -1.07 | -0.53 | -0.17 | 0.95 | -1.68 | -0.19 | 1.80 | -0.09 | 0.66 |
| Q9Y3U8 | 60S ribosomal protein L36 | RPL36 | 0.11 | 0.14 | -0.07 | -2.04 | -0.50 | 0.49 | 1.91 | -0.38 | -0.26 | 0.30 | 0.61 | -0.07 |
| P55268 | Laminin subunit beta-2 | LAMB2 | 0.11 | 0.25 | 0.48 | -1.95 | 0.28 | -0.17 | 1.01 | 0.75 | 0.33 | -1.62 | 0.16 | 0.73 |
| Q96PU5 | E3 ubiquitin-protein ligase NEDD4-like | NEDD4L | 0.11 | 0.11 | 0.72 | 1.13 | -0.21 | 0.58 | -2.38 | -0.03 | 0.35 | -0.88 | 0.42 | 0.30 |
| Q9NSY0 | Nuclear receptor-binding protein 2 | NRBP2 | 0.11 | 0.22 | 0.07 | 0.62 | -0.81 | 0.54 | -0.73 | 1.64 | 0.29 | -2.05 | 0.03 | 0.40 |
| Q8WX93 | Palladin | PALLD | 0.11 | 0.26 | -0.38 | 0.69 | 0.24 | 0.01 | -0.93 | 1.64 | 0.17 | -2.12 | 0.22 | 0.44 |
| Q9BYW2 | Histone-lysine N-methyltransferase SETD2 | SETD2 | 0.11 | 0.12 | -0.76 | 1.10 | -1.07 | 0.65 | -0.10 | -0.38 | -0.79 | -1.15 | 0.78 | 1.71 |
| O95741 | Copine-6 | CPNE6 | 0.11 | 0.27 | -0.55 | 1.24 | -0.62 | 0.49 | -0.92 | 0.95 | -0.56 | -1.69 | 0.51 | 1.17 |
| P20020 | Plasma membrane calcium-transporting ATPase 1 | ATP2B1 | 0.11 | 0.22 | 0.02 | 1.72 | 0.12 | -0.79 | -1.37 | 0.44 | 1.09 | -1.15 | -0.68 | 0.61 |
| Q5HYI8 | Rab-like protein 3 | RABL3 | 0.11 | 0.11 | 0.60 | 0.24 | -1.96 | 0.55 | 0.40 | 0.48 | 0.25 | -1.80 | 0.52 | 0.70 |
| Q9Y6I9 | Testis-expressed protein 264 | TEX264 | 0.11 | 0.13 | 0.22 | 1.39 | -0.91 | -0.78 | -0.11 | 1.59 | -0.65 | -1.09 | -0.65 | 0.99 |
| Q96G03 | Phosphoglucomutase-2 | PGM2 | 0.11 | 0.10 | -1.73 | 0.54 | 0.23 | 0.47 | 0.35 | -0.82 | -1.41 | 0.51 | 1.51 | 0.34 |
| Q969P0 | Immunoglobulin superfamily member 8 | IGSF8 | 0.11 | 0.13 | -0.43 | -0.37 | 0.40 | 1.15 | -0.95 | -0.93 | -1.20 | 1.53 | 1.21 | -0.41 |
| Q9UJ41 | Rab5 GDP/GTP exchange factor | RABGEF1 | 0.11 | 0.10 | -1.45 | -0.04 | 1.69 | -0.40 | 0.06 | -0.81 | -1.08 | 1.32 | 0.52 | 0.19 |
| O43290 | U4/U6.U5 tri-snRNP-associated protein 1 | SART1 | 0.11 | 0.32 | -0.61 | 0.37 | -1.14 | 0.39 | 0.56 | -1.37 | 1.75 | -1.02 | 0.27 | 0.81 |
| Q8NBF6 | Late secretory pathway protein AVL9 homolog | AVL9 | 0.11 | 0.21 | -0.04 | 0.54 | -0.18 | -0.44 | -0.18 | 2.47 | 0.14 | -0.40 | -0.56 | -1.35 |
| Q15691 | Microtubule-associated protein RP/EB family member 1 | MAPRE1 | 0.11 | 0.38 | -1.61 | -0.80 | 0.24 | 0.46 | 1.21 | 0.93 | -1.52 | -0.20 | 0.64 | 0.66 |
| Q71UM5 | 40S ribosomal protein S27-like | RPS27L | 0.10 | 0.38 | 1.08 | -1.34 | -0.50 | 0.63 | -0.38 | 1.00 | 0.90 | -1.44 | -0.80 | 0.84 |
| P55735 | Protein SEC13 homolog | SEC13 | 0.10 | 0.25 | 0.90 | 1.08 | -1.52 | 0.20 | -1.01 | 0.18 | 0.04 | -1.47 | 0.53 | 1.07 |
| Q86XK2 | F-box only protein 11 | FBXO11 | 0.10 | 0.16 | -0.28 | -0.64 | -0.95 | 1.49 | 0.14 | -0.66 | -0.47 | -1.04 | 1.82 | 0.58 |
| Q15582 | Transforming growth factor-beta-induced protein ig-h3 | TGFBI | 0.10 | 0.10 | -0.37 | -0.55 | 1.78 | -0.44 | -0.57 | -0.54 | -0.47 | 2.00 | -0.44 | -0.40 |
| P49915 | GMP synthase [glutamine-hydrolyzing] | GMPS | 0.10 | 0.64 | 1.12 | -0.77 | -1.44 | 0.45 | -0.16 | 0.13 | -1.63 | 1.29 | 0.49 | 0.53 |
| P54289 | Voltage-dependent calcium channel subunit alpha-2/delta-1 | CACNA2D1 | 0.10 | 0.10 | 0.46 | 1.46 | -1.16 | 0.13 | -1.05 | 0.05 | 0.43 | 1.51 | -0.87 | -0.98 |
| P61266 | Syntaxin-1B | STX1B | 0.10 | 0.26 | -0.93 | 2.22 | -0.50 | -0.04 | -1.12 | -0.53 | 0.84 | -0.67 | 0.50 | 0.22 |
| Q969V6 | Myocardin-related transcription factor A | MRTFA | 0.10 | 0.07 | 0.98 | 0.63 | -1.07 | 0.32 | -0.96 | 0.33 | 1.93 | -0.53 | -0.70 | -0.93 |
| Q9Y6A2 | Cholesterol 24-hydroxylase | CYP46A1 | 0.10 | 0.31 | 0.00 | -0.32 | 0.19 | 0.40 | -0.69 | -1.76 | -0.31 | 2.19 | 0.46 | -0.15 |
| Q96F86 | Enhancer of mRNA-decapping protein 3 | EDC3 | 0.10 | 0.14 | 1.87 | -1.46 | -0.78 | 0.59 | -0.43 | 0.49 | 0.98 | -0.76 | 0.15 | -0.66 |
| Q8NEZ4 | Histone-lysine N-methyltransferase 2C | KMT2C | 0.10 | 0.08 | 1.00 | -0.74 | -0.85 | 1.25 | -0.78 | 1.32 | -0.71 | -0.91 | 1.05 | -0.63 |
| O15226 | NF-kappa-B-repressing factor | NKRF | 0.10 | 0.09 | -0.33 | -0.81 | 1.28 | -0.72 | 0.44 | -0.71 | 2.15 | -0.15 | -0.41 | -0.75 |
| O75153 | Clustered mitochondria protein homolog | CLUH | 0.10 | 0.24 | -0.66 | 1.03 | -0.34 | -0.38 | 0.01 | 1.41 | -0.33 | -2.01 | 0.25 | 1.02 |
| P33121 | Long-chain-fatty-acid--CoA ligase 1 | ACSL1 | 0.10 | 0.08 | -1.18 | 0.55 | -0.13 | 0.71 | -0.06 | -1.21 | -1.22 | 0.04 | 0.66 | 1.84 |
| P52564 | Dual specificity mitogen-activated protein kinase kinase 6 | MAP2K6 | 0.10 | 0.13 | 0.13 | 0.88 | -0.14 | -0.16 | -0.91 | -0.11 | -1.98 | 1.77 | -0.01 | 0.52 |
| Q9HAP6 | Protein lin-7 homolog B | LIN7B | 0.10 | 0.11 | 1.64 | -0.40 | -0.56 | -0.42 | -0.42 | -0.39 | 2.11 | -0.73 | -0.47 | -0.36 |
| Q6X4W1 | NMDA receptor synaptonuclear signaling and neuronal migration f | NSMF | 0.10 | 0.11 | 2.11 | -0.51 | -0.65 | -0.50 | -0.61 | -0.40 | 1.14 | -0.88 | 0.85 | -0.54 |
| O15069 | NAC-alpha domain-containing protein 1 | NACAD | 0.10 | 0.16 | 0.60 | -1.36 | 1.90 | -0.03 | -1.35 | -0.51 | 0.39 | 0.79 | 0.13 | -0.56 |
| P14735 | Insulin-degrading enzyme | IDE | 0.10 | 0.13 | -0.78 | 0.62 | 0.29 | 0.55 | -0.86 | -0.75 | -0.95 | 0.57 | -0.80 | 2.11 |
| Q8TB24 | Ras and Rab interactor 3 | RIN3 | 0.10 | 0.22 | 0.01 | 1.70 | -0.92 | -0.64 | -0.46 | -0.41 | -0.50 | -1.17 | 1.23 | 1.16 |
| Q96AQ6 | Pre-B-cell leukemia transcription factor-interacting protein 1 | PBXIP1 | 0.10 | 0.14 | -0.49 | -0.63 | -0.35 | 0.77 | 0.51 | 0.85 | -2.38 | 0.46 | 0.61 | 0.65 |
| P18085 | ADP-ribosylation factor 4 | ARF4 | 0.10 | 0.32 | -0.58 | 0.67 | -0.76 | 0.92 | -0.69 | 0.36 | -0.21 | -1.92 | 1.15 | 1.06 |
| Q9H7P6 | Multivesicular body subunit 12B | MVB12B | 0.10 | 0.11 | -1.73 | -0.46 | 0.73 | 1.28 | 0.02 | 0.75 | -1.20 | -0.69 | 1.03 | 0.26 |
| P35237 | Serpin B6 | SERPINB6 | 0.09 | 0.10 | -0.66 | 1.15 | 0.07 | 0.26 | -0.97 | 0.32 | -2.04 | 1.35 | 0.19 | 0.33 |
| Q9ULC3 | Ras-related protein Rab-23 | RAB23 | 0.09 | 0.33 | 0.08 | 1.15 | -0.63 | 0.03 | -1.09 | 2.11 | -0.46 | -1.17 | 0.17 | -0.18 |
| P51808 | Dynein light chain Tctex-type 3 | DYNLT3 | 0.09 | 0.13 | -0.52 | -1.95 | 0.38 | 0.84 | 1.07 | -0.36 | -1.19 | 0.32 | 0.34 | 1.09 |
| Q53EL6 | Programmed cell death protein 4 | PDCD4 | 0.09 | 0.10 | 0.22 | -0.08 | -1.20 | 0.97 | -0.06 | 0.57 | -1.03 | -1.42 | 0.28 | 1.75 |
| Q9HBI0 | Gamma-parvin | PARVG | 0.09 | 0.08 | 1.70 | -0.72 | 0.18 | -0.55 | -0.73 | 1.54 | 0.80 | -0.91 | -0.66 | -0.65 |
| P19634 | Sodium/hydrogen exchanger 1 | SLC9A1 | 0.09 | 0.10 | -1.47 | 1.36 | -0.41 | 1.06 | -0.69 | -0.65 | 0.17 | -1.11 | 0.78 | 0.97 |
| P49770 | Translation initiation factor eIF-2B subunit beta | EIF2B2 | 0.09 | 0.11 | 1.35 | 0.32 | -1.15 | 0.51 | -1.18 | 1.13 | -0.52 | -1.36 | 0.08 | 0.82 |
| Q8WVC0 | RNA polymerase-associated protein LEO1 | LEO1 | 0.09 | 0.11 | 0.06 | 1.02 | -2.12 | 0.83 | 0.04 | 0.24 | 0.38 | -0.93 | 1.13 | -0.66 |
| Q8IWI9 | MAX gene-associated protein | MGA | 0.09 | 0.11 | -1.94 | -0.37 | 0.57 | 0.56 | 1.01 | 0.86 | -0.17 | -1.48 | 0.52 | 0.43 |
| Q12974 | Protein tyrosine phosphatase type IVA 2 | PTP4A2 | 0.09 | 0.06 | 1.21 | -0.08 | 0.26 | -0.36 | -1.12 | 1.45 | 1.07 | -1.34 | -0.08 | -1.02 |
| Q99572 | P2X purinoceptor 7 | P2RX7 | 0.09 | 0.22 | -0.17 | -0.66 | 1.72 | -0.58 | -0.62 | -0.68 | -0.75 | 0.57 | 1.77 | -0.60 |
| P36543 | V-type proton ATPase subunit E 1 | ATP6V1E1 | 0.09 | 0.27 | 0.01 | 1.07 | -0.19 | 0.84 | -2.10 | -0.76 | -0.55 | 1.23 | 0.53 | -0.07 |
| Q7L3B6 | Hsp90 co-chaperone Cdc37-like 1 | CDC37L1 | 0.09 | 0.11 | -0.18 | 1.51 | -0.61 | -0.46 | -0.41 | -0.35 | -0.38 | -0.82 | -0.47 | 2.18 |
| Q9P215 | Pogo transposable element with KRAB domain | POGK | 0.09 | 0.10 | 1.02 | -0.91 | -1.09 | -0.13 | 0.96 | 0.61 | 1.28 | -1.42 | 0.51 | -0.83 |
| Q15714 | TSC22 domain family protein 1 | TSC22D1 | 0.09 | 0.05 | -1.46 | 0.78 | -1.02 | 0.70 | 0.93 | -0.38 | -1.53 | 0.47 | 0.77 | 0.76 |
| Q8TDX7 | Serine/threonine-protein kinase Nek7 | NEK7 | 0.09 | 0.10 | 0.81 | 0.42 | -1.55 | 0.37 | -0.20 | 1.07 | -1.71 | -0.64 | 0.49 | 0.94 |
| P26641 | Elongation factor 1-gamma | EEF1G | 0.09 | 0.19 | 0.89 | -0.18 | 0.91 | -0.21 | -1.68 | 0.99 | 0.78 | -1.67 | -0.13 | 0.30 |
| Q8WU90 | Zinc finger CCCH domain-containing protein 15 | ZC3H15 | 0.09 | 0.20 | 1.39 | -0.25 | -1.68 | 0.49 | -0.23 | 0.41 | -0.29 | -1.51 | 0.91 | 0.75 |
| Q9Y3D6 | Mitochondrial fission 1 protein | FIS1 | 0.09 | 0.20 | 0.00 | 0.08 | -0.99 | -0.52 | 1.15 | -0.84 | -1.69 | 0.54 | 1.12 | 1.15 |
| Q6P587 | Acylpyruvase FAHD1, mitochondrial | FAHD1 | 0.09 | 0.08 | -0.28 | -0.49 | -0.62 | -0.53 | 1.81 | -0.47 | -0.49 | 1.97 | -0.43 | -0.47 |
| P43304 | Glycerol-3-phosphate dehydrogenase, mitochondrial | GPD2 | 0.09 | 0.22 | -0.07 | 0.13 | -0.97 | -0.49 | 1.09 | -0.35 | 1.84 | -1.30 | -0.78 | 0.90 |
| A0A0B4J2D5 | Glutamine amidotransferase-like class 1 domain-containing protei | GATD3B | 0.09 | 0.33 | -0.62 | 0.04 | 1.39 | -1.30 | 0.05 | 1.95 | -0.35 | -0.14 | -1.03 | 0.02 |
| P22314 | Ubiquitin-like modifier-activating enzyme 1 | UBA1 | 0.08 | 0.12 | -2.35 | 0.43 | 0.13 | 1.00 | 0.61 | -0.43 | 0.35 | -0.86 | 0.91 | 0.21 |
| Q96JG6 | Syndetin | VPS50 | 0.08 | 0.11 | -0.19 | 1.58 | 0.21 | -1.41 | -0.35 | -1.32 | 0.58 | 0.66 | -0.82 | 1.06 |
| Q969G3 | SWI/SNF-related matrix-associated actin-dependent regulator of c | SMARCE1 | 0.08 | 0.11 | -0.19 | -0.50 | -0.52 | 1.62 | -0.57 | -0.44 | -0.45 | -0.72 | -0.34 | 2.12 |
| Q16718 | NADH dehydrogenase [ubiquinone] 1 alpha subcomplex subunit 5 | NDUFA5 | 0.08 | 0.35 | 0.70 | -0.03 | -0.84 | -0.28 | -0.03 | 0.24 | -0.47 | -1.52 | -0.04 | 2.27 |
| P02748 | Complement component C9 | C9 | 0.08 | 0.07 | -0.58 | -0.10 | -0.75 | 0.53 | 0.80 | -0.72 | -0.66 | -0.95 | 0.14 | 2.30 |
| P41222 | Prostaglandin-H2 D-isomerase | PTGDS | 0.08 | 0.08 | -0.52 | -1.02 | 1.23 | 0.21 | -0.01 | -0.65 | -1.44 | 1.84 | 0.47 | -0.11 |
| P07948 | Tyrosine-protein kinase Lyn | LYN | 0.08 | 0.12 | -1.97 | 1.40 | 0.22 | 0.56 | -0.38 | -0.48 | -1.06 | 0.14 | 0.61 | 0.96 |
| P46060 | Ran GTPase-activating protein 1 | RANGAP1 | 0.08 | 0.12 | 1.20 | -0.21 | -1.31 | -1.12 | 1.27 | 0.80 | 0.82 | -0.18 | -1.25 | -0.01 |
| Q9NX58 | Cell growth-regulating nucleolar protein | LYAR | 0.08 | 0.12 | -0.85 | 0.63 | 1.80 | -1.28 | -0.48 | -0.81 | -0.64 | 0.15 | 0.19 | 1.28 |
| Q96CS3 | FAS-associated factor 2 | FAF2 | 0.08 | 0.12 | 0.65 | 0.92 | -1.66 | 0.48 | -0.55 | 0.53 | 0.83 | -1.79 | 0.07 | 0.54 |
| Q53G44 | Interferon-induced protein 44-like | IFI44L | 0.08 | 0.11 | 1.02 | -1.09 | -1.44 | 0.71 | 0.65 | -0.46 | 1.62 | -0.01 | -0.02 | -0.97 |
| P35754 | Glutaredoxin-1 | GLRX | 0.08 | 0.06 | -0.52 | 0.00 | -0.67 | -0.62 | 1.72 | -0.61 | -0.11 | 1.97 | -0.54 | -0.62 |

| | | | | | | | | | | | | | | |
|---|---|---|---|---|---|---|---|---|---|---|---|---|---|---|
| Q93052 | Lipoma-preferred partner | LPP | 0.08 | 0.08 | 0.11 | 0.85 | -1.27 | 0.28 | -0.08 | 1.25 | 0.02 | -2.00 | -0.14 | 0.98 |
| P49848 | Transcription initiation factor TFIID subunit 6 | TAF6 | 0.08 | 0.36 | 0.55 | 0.04 | -0.94 | 0.19 | -0.33 | -0.30 | 0.08 | -1.45 | -0.18 | 2.34 |
| P29590 | Protein PML | PML | 0.08 | 0.12 | 0.45 | 0.24 | 1.06 | -0.74 | -1.17 | 0.39 | -1.64 | -0.73 | 1.13 | 1.02 |
| Q63HM9 | PI-PLC X domain-containing protein 3 | PLCXD3 | 0.08 | 0.08 | -1.10 | -0.50 | 0.64 | 0.40 | 0.44 | -1.36 | -1.29 | 1.58 | 0.42 | 0.77 |
| O14964 | Hepatocyte growth factor-regulated tyrosine kinase substrate | HGS | 0.08 | 0.15 | -0.03 | 1.20 | -0.27 | 0.56 | -1.68 | -0.07 | 1.33 | -1.48 | 0.59 | -0.14 |
| Q6GYQ0 | Ral GTPase-activating protein subunit alpha-1 | RALGAPA1 | 0.08 | 0.06 | 1.10 | 0.71 | -1.81 | 0.02 | -0.11 | 0.68 | 1.24 | -1.38 | -0.20 | -0.25 |
| P78347 | General transcription factor II-I | GTF2I | 0.08 | 0.13 | -0.01 | 0.95 | -1.73 | 1.36 | -0.75 | -1.04 | -0.08 | -0.30 | 1.12 | 0.49 |
| P85037 | Forkhead box protein K1 | FOXK1 | 0.08 | 0.10 | 0.29 | 1.09 | -1.10 | -0.89 | 0.46 | 1.69 | 0.71 | -1.17 | -0.85 | -0.25 |
| Q8N573 | Oxidation resistance protein 1 | OXR1 | 0.08 | 0.14 | 0.79 | 0.09 | -0.47 | -0.64 | 0.02 | 2.37 | 0.26 | -0.56 | -0.98 | -0.88 |
| P61204 | ADP-ribosylation factor 3 | ARF3 | 0.08 | 0.21 | 0.69 | -1.98 | -0.70 | 0.96 | 0.73 | -1.07 | -0.27 | 0.64 | 1.01 | -0.02 |
| Q13045 | Protein flightless-1 homolog | FLII | 0.08 | 0.15 | 0.79 | 1.17 | -1.61 | 0.25 | -0.82 | 0.88 | -0.13 | -1.52 | 0.28 | 0.71 |
| P07585 | Decorin | DCN | 0.08 | 0.11 | 0.80 | -0.03 | -0.88 | 0.78 | -0.84 | -2.10 | 0.07 | 0.69 | 0.42 | 1.09 |
| Q5T6J7 | Probable gluconokinase | IDNK | 0.08 | 0.09 | 1.48 | -0.32 | -0.61 | -0.30 | -0.37 | 2.21 | -0.44 | -0.84 | -0.50 | -0.30 |
| O15111 | Inhibitor of nuclear factor kappa-B kinase subunit alpha | CHUK | 0.08 | 0.09 | 0.63 | -0.31 | -1.61 | 1.31 | -0.15 | 0.86 | -0.08 | -1.68 | 0.68 | 0.35 |
| Q9Y3F4 | Serine-threonine kinase receptor-associated protein | STRAP | 0.08 | 0.09 | 1.52 | -0.02 | -0.60 | -0.42 | -0.60 | 1.81 | 0.57 | -1.33 | -0.50 | -0.43 |
| Q14119 | Vascular endothelial zinc finger 1 | VEZF1 | 0.08 | 0.12 | 0.97 | -1.14 | -1.20 | 0.67 | 0.53 | 0.01 | -0.95 | 0.85 | 1.35 | -1.08 |
| Q9BYR9 | Keratin-associated protein 2-4 | KRTAP2-4 | 0.08 | 0.05 | -0.42 | 1.92 | -0.56 | -0.48 | -0.53 | -0.39 | -0.46 | 1.87 | -0.47 | -0.48 |
| P08195 | 4F2 cell-surface antigen heavy chain | SLC3A2 | 0.07 | 0.12 | 0.58 | 0.73 | 0.78 | -0.04 | -2.24 | 0.83 | 0.53 | 0.40 | -0.55 | -1.03 |
| Q9UBQ0 | Vacuolar protein sorting-associated protein 29 | VPS29 | 0.07 | 0.22 | -0.59 | 0.63 | -0.97 | -0.12 | 0.74 | 1.14 | 0.71 | -2.17 | 0.47 | 0.16 |
| Q7L266 | Isoaspartyl peptidase/L-asparaginase | ASRGL1 | 0.07 | 0.08 | -0.38 | 0.20 | -0.06 | -0.52 | 0.64 | 0.37 | -1.64 | 2.22 | -0.48 | -0.36 |
| Q96S19 | Methyltransferase-like 26 | METTL26 | 0.07 | 0.10 | 1.37 | 1.00 | -0.58 | 0.23 | -2.17 | 0.04 | 0.39 | 0.16 | 0.39 | -0.82 |
| Q9UQ03 | Coronin-2B | CORO2B | 0.07 | 0.14 | 0.92 | -0.79 | -0.85 | 0.77 | -0.25 | 1.57 | 0.40 | -1.86 | -0.12 | 0.22 |
| Q13009 | T-lymphoma invasion and metastasis-inducing protein 1 | TIAM1 | 0.07 | 0.06 | 1.25 | -0.01 | -1.24 | 0.11 | -0.20 | 0.73 | 1.35 | -1.86 | 0.00 | -0.14 |
| Q14194 | Dihydropyrimidinase-related protein 1 | CRMP1 | 0.07 | 0.14 | 0.69 | -1.00 | -0.50 | -1.00 | 1.62 | 0.40 | 0.33 | 1.28 | -0.71 | -1.11 |
| O60826 | Coiled-coil domain-containing protein 22 | CCDC22 | 0.07 | 0.08 | 1.52 | -0.11 | -1.07 | 0.41 | -0.87 | 1.18 | 1.11 | -1.25 | -0.53 | -0.39 |
| Q00005 | Serine/threonine-protein phosphatase 2A 55 kDa regulatory subuni | PPP2R2B | 0.07 | 0.07 | 1.26 | -0.33 | -1.54 | 0.33 | 0.18 | 1.09 | 0.40 | -1.75 | 0.58 | -0.21 |
| P62263 | 40S ribosomal protein S14 | RPS14 | 0.07 | 0.16 | 0.77 | -0.54 | 1.24 | -0.88 | -0.82 | 0.82 | 0.35 | -1.42 | -0.81 | 1.30 |
| P17252 | Protein kinase C alpha type | PRKCA | 0.07 | 0.16 | 0.48 | -0.33 | -1.06 | 0.86 | -0.19 | 1.36 | -0.28 | -2.00 | 0.89 | 0.26 |
| Q9H299 | SH3 domain-binding glutamic acid-rich-like protein 3 | SH3BGRL3 | 0.07 | 0.14 | 0.96 | 1.28 | -1.23 | 0.16 | -1.37 | 0.80 | 0.65 | -1.30 | -0.32 | 0.37 |
| Q9NTK5 | Obg-like ATPase 1 | OLA1 | 0.07 | 0.17 | -0.46 | 0.77 | 0.64 | -0.49 | -0.69 | 1.11 | 0.89 | -2.10 | -0.37 | 0.71 |
| Q8N9N7 | Leucine-rich repeat-containing protein 57 | LRRC57 | 0.07 | 0.17 | -0.27 | 1.27 | -2.33 | 0.40 | 0.70 | -0.05 | 0.79 | -0.65 | 0.36 | -0.20 |
| Q7L1Q6 | Basic leucine zipper and W2 domain-containing protein 1 | BZW1 | 0.07 | 0.08 | 0.26 | 0.27 | -0.18 | 0.05 | -0.51 | 1.25 | -0.35 | -2.34 | 0.47 | 1.09 |
| Q14525 | Keratin, type I cuticular Ha3-II | KRT33B | 0.07 | 0.06 | -0.27 | 1.77 | -0.64 | -0.43 | -0.52 | -0.50 | -0.49 | 2.00 | -0.55 | -0.38 |
| Q9P246 | Stromal interaction molecule 2 | STIM2 | 0.07 | 0.11 | -0.24 | 1.53 | -0.55 | -0.41 | -0.49 | -0.39 | -0.34 | -0.88 | -0.39 | 2.16 |
| Q05469 | Hormone-sensitive lipase | LIPE | 0.07 | 0.15 | -0.94 | -0.86 | 1.37 | -0.03 | 0.25 | -1.59 | -0.56 | 0.08 | 1.12 | 1.16 |
| P26885 | Peptidyl-prolyl cis-trans isomerase FKBP2 | FKBP2 | 0.07 | 0.07 | 0.74 | 0.95 | -1.60 | 0.25 | -0.45 | 1.02 | 0.10 | -1.83 | 0.40 | 0.41 |
| P61916 | NPC intracellular cholesterol transporter 2 | NPC2 | 0.07 | 0.06 | -0.43 | -0.51 | -0.05 | -0.56 | 1.47 | -0.54 | -0.50 | 2.22 | -0.63 | -0.47 |
| Q5JS13 | Ras-specific guanine nucleotide-releasing factor RalGPS1 | RALGPS1 | 0.07 | 0.11 | -0.28 | 1.60 | -0.54 | -0.49 | -0.44 | -0.45 | 2.12 | -0.82 | -0.31 | -0.39 |
| Q9NPQ8 | Synembryn-A | RIC8A | 0.07 | 0.10 | 0.24 | 1.07 | -1.67 | 0.79 | -0.58 | 1.04 | -0.07 | -1.62 | 0.43 | 0.36 |
| P61224 | Ras-related protein Rap-1b | RAP1B | 0.07 | 0.28 | 0.07 | -2.45 | 0.33 | 0.23 | 1.42 | 0.15 | -0.51 | 0.22 | 0.68 | -0.16 |
| P48507 | Glutamate--cysteine ligase regulatory subunit | GCLM | 0.07 | 0.13 | -0.39 | 0.75 | -0.02 | 0.81 | -1.33 | 1.40 | -1.21 | -1.08 | 1.13 | -0.06 |
| P02765 | Alpha-2-HS-glycoprotein | AHSG | 0.07 | 0.07 | 0.96 | -0.43 | 0.72 | 0.33 | -1.68 | 0.65 | 0.54 | 0.96 | -0.44 | -1.62 |
| Q14498 | RNA-binding protein 39 | RBM39 | 0.07 | 0.07 | 1.03 | -0.58 | -0.81 | -0.44 | 0.71 | 1.13 | 0.82 | -1.99 | -0.33 | 0.48 |
| Q15811 | Intersectin-1 | ITSN1 | 0.07 | 0.10 | 1.51 | 0.13 | -1.08 | -0.34 | -0.37 | 1.27 | 1.11 | -1.40 | -0.18 | -0.66 |
| Q8IUD2 | ELKS/Rab6-interacting/CAST family member 1 | ERC1 | 0.07 | 0.16 | -1.48 | 0.64 | 0.97 | 0.13 | -0.49 | -0.71 | 1.37 | -1.49 | 0.47 | 0.59 |
| Q13315 | Serine-protein kinase ATM | ATM | 0.07 | 0.09 | 1.09 | -0.10 | -2.28 | 1.01 | 0.14 | -0.44 | 0.73 | -0.68 | 0.62 | -0.09 |
| Q99447 | Ethanolamine-phosphate cytidylyltransferase | PCYT2 | 0.07 | 0.25 | -1.05 | -1.53 | 0.67 | 1.12 | 0.44 | -0.96 | -0.31 | -0.16 | 1.61 | 0.17 |
| P22695 | Cytochrome b-c1 complex subunit 2, mitochondrial | UQCRC2 | 0.07 | 0.19 | -0.68 | -0.87 | 2.30 | -0.41 | -0.61 | -1.00 | 0.05 | 0.91 | 0.32 | -0.01 |
| P20592 | Interferon-induced GTP-binding protein Mx2 | MX2 | 0.07 | 0.05 | 0.96 | 1.56 | -0.93 | -0.76 | -0.91 | 0.81 | 1.15 | -0.26 | -0.84 | -0.79 |
| Q99963 | Endophilin-A3 | SH3GL3 | 0.07 | 0.13 | -0.22 | 0.07 | 1.53 | -0.27 | -1.30 | 0.39 | 1.41 | 0.18 | -0.18 | -1.63 |
| Q6IA69 | Glutamine-dependent NAD(+) synthetase | NADSYN1 | 0.06 | 0.08 | -0.68 | -0.99 | 1.53 | 1.06 | -1.03 | -0.84 | -0.85 | 0.98 | 0.02 | 0.81 |
| Q7Z3U7 | Protein MON2 homolog | MON2 | 0.06 | 0.12 | -0.46 | 1.06 | -1.05 | 1.37 | -1.09 | 0.18 | -1.24 | -0.08 | 1.38 | -0.06 |
| Q9HCB6 | Spondin-1 | SPON1 | 0.06 | 0.14 | -0.83 | -0.77 | 0.80 | 0.01 | 0.58 | -0.60 | -0.99 | 2.19 | -0.71 | 0.31 |
| O94827 | Pleckstrin homology domain-containing family G member 5 | PLEKHG5 | 0.06 | 0.06 | 1.75 | -1.06 | -0.04 | -0.18 | -0.56 | -0.97 | -1.03 | 1.37 | -0.06 | 0.77 |
| O43815 | Striatin | STRN | 0.06 | 0.34 | -0.08 | 0.14 | -1.58 | -0.98 | 2.04 | 0.19 | 0.43 | 0.12 | 0.60 | -0.88 |
| Q15365 | Poly(rC)-binding protein 1 | PCBP1 | 0.06 | 0.17 | 0.28 | 0.12 | -2.29 | 1.27 | 0.38 | 0.00 | -0.21 | -0.79 | 1.11 | 0.14 |
| P61803 | Dolichyl-diphosphooligosaccharide--protein glycosyltransferase sul | DAD1 | 0.06 | 0.06 | 0.75 | -1.15 | -1.30 | 1.07 | 0.54 | -1.04 | -1.11 | 0.78 | 0.71 | 0.76 |
| P61313 | 60S ribosomal protein L15 | RPL15 | 0.06 | 0.06 | -0.82 | -1.48 | -0.01 | -0.13 | 2.35 | -0.32 | -0.32 | 0.63 | 0.02 | 0.08 |
| O75494 | Serine/arginine-rich splicing factor 10 | SRSF10 | 0.06 | 0.20 | -2.10 | -0.05 | 0.07 | 0.84 | 0.95 | -0.54 | 0.54 | -0.91 | 1.25 | -0.06 |
| Q96PU8 | Protein quaking | QKI | 0.06 | 0.13 | 0.01 | -0.25 | -1.13 | 0.66 | 0.52 | 0.45 | -1.88 | -0.62 | 1.51 | 0.72 |
| Q14240 | Eukaryotic initiation factor 4A-II | EIF4A2 | 0.06 | 0.18 | 0.07 | -1.03 | 0.51 | 0.27 | -0.08 | 0.55 | 1.26 | -2.30 | 0.09 | 0.65 |
| P11021 | Endoplasmic reticulum chaperone BiP | HSPA5 | 0.06 | 0.20 | -0.28 | 1.60 | 1.07 | -1.30 | -1.37 | -0.02 | -0.14 | 0.18 | -0.76 | 1.02 |
| P47914 | 60S ribosomal protein L29 | RPL29 | 0.06 | 0.20 | 0.03 | -0.52 | 0.81 | -1.62 | 1.01 | -0.73 | 1.13 | -0.09 | -1.16 | 1.13 |
| Q99714 | 3-hydroxyacyl-CoA dehydrogenase type-2 | HSD17B10 | 0.06 | 0.13 | 0.13 | -0.61 | 1.65 | 0.02 | -1.37 | 1.33 | 0.84 | -1.04 | -0.46 | -0.49 |
| Q15020 | Squamous cell carcinoma antigen recognized by T-cells 3 | SART3 | 0.06 | 0.07 | -1.73 | 0.63 | 0.71 | -0.04 | 0.32 | -1.71 | 1.43 | 0.14 | 0.13 | 0.11 |
| Q9GZQ8 | Microtubule-associated proteins 1A/1B light chain 3B | MAP1LC3B | 0.06 | 0.24 | 1.22 | -0.30 | -1.24 | 0.65 | -0.66 | -0.92 | 0.86 | -0.51 | 1.63 | -0.73 |
| Q9P0L2 | Serine/threonine-protein kinase MARK1 | MARK1 | 0.06 | 0.13 | -0.16 | -0.75 | 0.05 | 0.11 | 0.56 | 2.31 | -1.41 | 0.21 | -0.08 | -0.84 |
| Q9ULH1 | Arf-GAP with SH3 domain, ANK repeat and PH domain-containing | ASAP1 | 0.06 | 0.04 | -0.44 | 1.02 | -1.42 | 0.54 | 0.24 | -0.65 | 1.54 | -1.53 | 0.28 | 0.44 |
| Q15907 | Ras-related protein Rab-11B | RAB11B | 0.06 | 0.27 | -0.18 | -0.79 | -0.09 | 1.45 | -0.77 | -0.05 | 0.45 | -1.90 | 1.12 | 0.76 |
| O60884 | DnaJ homolog subfamily A member 2 | DNAJA2 | 0.06 | 0.14 | 0.85 | 0.74 | -1.30 | 0.79 | -1.29 | 0.68 | -0.92 | -1.08 | 0.53 | 1.00 |
| P06730 | Eukaryotic translation initiation factor 4E | EIF4E | 0.06 | 0.13 | -0.78 | 0.20 | -0.17 | 0.77 | -0.20 | -1.90 | -0.92 | 0.64 | 1.03 | 1.33 |
| Q8NBU5 | ATPase family AAA domain-containing protein 1 | ATAD1 | 0.06 | 0.08 | -0.80 | 0.30 | -1.07 | 1.17 | 0.28 | 0.45 | -0.56 | -1.77 | 1.14 | 0.86 |
| Q9Y281 | Cofilin-2 | CFL2 | 0.06 | 0.17 | -0.59 | -0.37 | 1.16 | 0.85 | -1.29 | -0.19 | -1.36 | -0.51 | 1.21 | 1.09 |
| O43583 | Density-regulated protein | DENR | 0.06 | 0.16 | 0.91 | -0.07 | 1.19 | -1.02 | -1.24 | -0.44 | 1.15 | 1.12 | -1.06 | -0.54 |
| Q96IJ6 | Mannose-1-phosphate guanyltransferase alpha | GMPPA | 0.05 | 0.21 | 1.12 | 0.24 | 0.22 | 0.26 | -2.13 | 0.65 | 0.19 | -1.39 | 0.04 | 0.80 |
| O14639 | Actin-binding LIM protein 1 | ABLIM1 | 0.05 | 0.09 | -0.62 | 1.44 | -0.71 | 0.13 | -0.38 | 0.30 | -1.37 | -0.93 | 0.43 | 1.70 |
| Q13509 | Tubulin beta-3 chain | TUBB3 | 0.05 | 0.14 | 1.12 | -0.11 | 0.08 | 1.06 | -2.35 | 0.54 | -0.44 | 0.10 | 0.53 | -0.52 |
| Q9BQ70 | Transcription factor 25 | TCF25 | 0.05 | 0.09 | -0.25 | 0.07 | -1.35 | 1.16 | 0.23 | 1.55 | -1.46 | -0.53 | 0.89 | -0.31 |
| Q92625 | Ankyrin repeat and SAM domain-containing protein 1A | ANKS1A | 0.05 | 0.08 | 1.03 | -0.79 | -1.07 | -0.68 | 1.40 | 0.75 | -0.75 | -1.27 | 0.48 | 0.90 |
| Q8WVF1 | Protein OSCP1 | OSCP1 | 0.05 | 0.06 | 1.67 | -0.42 | -0.55 | -0.38 | -0.41 | 2.07 | -0.36 | -0.86 | -0.45 | -0.31 |
| P20810 | Calpastatin | CAST | 0.05 | 0.10 | 1.42 | -0.82 | 0.95 | -1.01 | -0.70 | 1.02 | 0.97 | -1.09 | -0.84 | 0.09 |
| O95433 | Activator of 90 kDa heat shock protein ATPase homolog 1 | AHSA1 | 0.05 | 0.10 | 1.12 | -0.18 | -0.49 | 0.24 | -0.83 | 1.05 | 1.01 | -2.12 | -0.07 | 0.27 |
| P62857 | 40S ribosomal protein S28 | RPS28 | 0.05 | 0.19 | 1.14 | -0.16 | -0.36 | -1.49 | 0.60 | -1.07 | 0.39 | -0.09 | -0.73 | 1.77 |

| | | | | | | | | | | | | | | |
|---|---|---|---|---|---|---|---|---|---|---|---|---|---|---|
| P36578 | 60S ribosomal protein L4 | RPL4 | 0.05 | 0.07 | -0.67 | -1.41 | -0.40 | -0.04 | 2.42 | -0.45 | 0.17 | 0.26 | -0.28 | 0.40 |
| Q03001 | Dystonin | DST | 0.05 | 0.10 | -0.07 | -1.17 | 0.47 | 0.10 | 0.52 | -0.34 | -0.99 | 2.29 | 0.08 | -0.89 |
| Q7Z569 | BRCA1-associated protein | BRAP | 0.05 | 0.04 | 0.89 | 1.06 | -1.15 | -0.86 | 0.00 | 1.08 | -1.02 | -1.22 | 0.10 | 1.13 |
| P46063 | ATP-dependent DNA helicase Q1 | RECQL | 0.05 | 0.04 | -0.39 | -1.18 | 0.43 | 0.66 | 0.42 | 0.25 | -2.16 | 1.27 | 0.17 | 0.53 |
| P32321 | Deoxycytidylate deaminase | DCTD | 0.05 | 0.06 | 0.80 | -0.67 | -0.89 | 1.39 | -0.71 | -0.77 | -0.83 | 1.25 | 1.15 | -0.72 |
| Q9H0R4 | Haloacid dehalogenase-like hydrolase domain-containing protein 2 | HDHD2 | 0.05 | 0.06 | -0.61 | -0.76 | 0.70 | 0.55 | 0.05 | -0.79 | -1.82 | 1.59 | 0.85 | 0.26 |
| P29692 | Elongation factor 1-delta | EEF1D | 0.05 | 0.04 | -1.35 | 0.18 | 0.87 | 0.76 | -0.52 | -1.17 | -1.31 | 1.24 | 0.86 | 0.43 |
| Q9Y697 | Cysteine desulfurase, mitochondrial | NFS1 | 0.05 | 0.05 | -1.22 | 0.27 | 0.78 | 0.21 | -0.11 | -1.90 | 0.90 | -0.79 | 0.92 | 0.94 |
| P28072 | Proteasome subunit beta type-6 | PSMB6 | 0.05 | 0.09 | -0.90 | -1.07 | 0.68 | -0.34 | 1.49 | -0.86 | -0.41 | 1.78 | 0.03 | -0.40 |
| Q7KZ85 | Transcription elongation factor SPT6 | SUPT6H | 0.05 | 0.03 | -1.57 | 1.19 | 0.15 | 0.53 | -0.35 | -1.62 | -0.42 | 0.49 | 0.37 | 1.22 |
| Q9NZM3 | Intersectin-2 | ITSN2 | 0.05 | 0.07 | 1.30 | -0.95 | -0.34 | -0.21 | 0.10 | 1.66 | 0.88 | -1.50 | -0.42 | -0.52 |
| Q05707 | Collagen alpha-1(XIV) chain | COL14A1 | 0.05 | 0.02 | -1.23 | 0.49 | 1.15 | -0.37 | -0.07 | -1.26 | 1.74 | -0.13 | -0.91 | 0.58 |
| Q15628 | Tumor necrosis factor receptor type 1-associated DEATH domain | TRADD | 0.04 | 0.05 | -0.54 | 0.49 | 1.33 | -0.67 | -0.69 | -0.65 | -0.78 | -1.09 | 1.01 | 1.59 |
| Q9ULP0 | Protein NDRG4 | NDRG4 | 0.04 | 0.07 | 0.59 | 1.22 | 0.45 | -0.19 | -2.16 | -0.27 | -0.39 | 1.27 | 0.12 | -0.63 |
| Q8NDY3 | [Protein ADP-ribosylarginine] hydrolase-like protein 1 | ADPRHL1 | 0.04 | 0.07 | 1.56 | -0.34 | -0.66 | -0.36 | -0.31 | 2.13 | -0.43 | -0.88 | -0.28 | -0.44 |
| P14854 | Cytochrome c oxidase subunit 6B1 | COX6B1 | 0.04 | 0.08 | -0.20 | 1.48 | -1.34 | -0.26 | 0.21 | -0.92 | -1.23 | 0.68 | 0.17 | 1.41 |
| O76024 | Wolframin | WFS1 | 0.04 | 0.04 | 0.19 | 0.87 | 0.10 | 0.21 | -1.42 | -0.26 | -1.35 | -0.93 | 1.24 | 1.36 |
| Q04609 | Glutamate carboxypeptidase 2 | FOLH1 | 0.04 | 0.07 | -0.40 | -0.91 | 2.00 | -0.67 | -0.12 | -0.90 | -0.80 | 1.38 | 0.43 | 0.00 |
| Q13163 | Dual specificity mitogen-activated protein kinase kinase 5 | MAP2K5 | 0.04 | 0.10 | -0.35 | 1.63 | -1.22 | 0.20 | -0.40 | -1.43 | -0.47 | 1.18 | -0.07 | 0.94 |
| P49750 | YLP motif-containing protein 1 | YLPM1 | 0.04 | 0.03 | 0.72 | 0.44 | -1.19 | 1.02 | -1.05 | 0.97 | -1.04 | -1.29 | 0.88 | 0.53 |
| Q13347 | Eukaryotic translation initiation factor 3 subunit I | EIF3I | 0.04 | 0.09 | 0.32 | -0.42 | 0.50 | 0.07 | -0.60 | 0.45 | 1.51 | -2.32 | 0.03 | 0.46 |
| O75312 | Zinc finger protein ZPR1 | ZPR1 | 0.04 | 0.06 | 0.73 | 0.55 | -0.85 | -0.81 | 0.29 | 1.57 | -0.64 | -1.26 | -0.83 | 1.25 |
| O60890 | Oligophrenin-1 | OPHN1 | 0.04 | 0.05 | 1.82 | -0.68 | -1.02 | -0.74 | 0.54 | 1.29 | 0.65 | -0.61 | -0.42 | -0.84 |
| O00534 | von Willebrand factor A domain-containing protein 5A | VWA5A | 0.04 | 0.07 | -0.42 | -0.44 | -0.17 | -1.26 | 2.18 | 0.28 | 1.21 | -0.40 | -0.33 | -0.66 |
| P55072 | Transitional endoplasmic reticulum ATPase | VCP | 0.04 | 0.02 | 1.12 | 0.10 | 0.59 | -1.70 | -0.15 | 0.26 | 0.83 | 1.12 | -0.88 | -1.31 |
| O00233 | 26S proteasome non-ATPase regulatory subunit 9 | PSMD9 | 0.04 | 0.04 | -0.60 | 0.77 | -0.93 | -0.63 | 1.33 | -0.76 | -0.80 | 1.49 | -0.82 | 0.95 |
| P20073 | Annexin A7 | ANXA7 | 0.04 | 0.12 | -0.38 | 1.73 | -0.37 | -0.77 | -0.38 | 0.22 | 0.16 | 1.72 | -1.21 | -0.71 |
| Q96DT6 | Cysteine protease ATG4C | ATG4C | 0.04 | 0.03 | 0.64 | 0.69 | -1.22 | 0.86 | -1.02 | 1.02 | -1.01 | -1.35 | 0.65 | 0.73 |
| Q08462 | Adenylate cyclase type 2 | ADCY2 | 0.04 | 0.04 | -0.08 | -1.39 | 2.50 | -0.68 | -0.42 | -0.17 | -0.08 | 0.24 | 0.22 | -0.15 |
| P46087 | Probable 28S rRNA (cytosine(4447)-C(5))-methyltransferase | NOP2 | 0.04 | 0.05 | -1.23 | 0.55 | 0.11 | 0.02 | 0.48 | -0.90 | 1.42 | -1.76 | 0.39 | 0.92 |
| P50579 | Methionine aminopeptidase 2 | METAP2 | 0.04 | 0.05 | -0.67 | -0.61 | 0.95 | -0.07 | 0.32 | -1.50 | -0.87 | 2.01 | 0.25 | 0.19 |
| Q8NE71 | ATP-binding cassette sub-family F member 1 | ABCF1 | 0.04 | 0.04 | -0.62 | -0.52 | 0.50 | 0.08 | 0.50 | -1.05 | -1.78 | 1.68 | 0.59 | 0.63 |
| P78344 | Eukaryotic translation initiation factor 4 gamma 2 | EIF4G2 | 0.04 | 0.06 | 0.85 | 0.44 | -0.65 | 0.37 | -1.10 | 0.88 | -0.14 | -2.06 | 0.35 | 1.07 |
| Q96KN4 | Protein LRATD1 | LRATD1 | 0.04 | 0.03 | 1.26 | -0.67 | -0.26 | -0.82 | 0.44 | 1.33 | -1.41 | 1.11 | 0.03 | -1.01 |
| Q6U841 | Sodium-driven chloride bicarbonate exchanger | SLC4A10 | 0.04 | 0.05 | -0.94 | 0.62 | -0.76 | 1.01 | 0.01 | -1.14 | 0.01 | -1.30 | 1.32 | 1.18 |
| Q8WY54 | Protein phosphatase 1E | PPM1E | 0.04 | 0.13 | -1.16 | 0.85 | -0.12 | 0.62 | -0.39 | -1.73 | 1.63 | 0.11 | 0.69 | -0.51 |
| P50552 | Vasodilator-stimulated phosphoprotein | VASP | 0.04 | 0.04 | -1.02 | 0.47 | 1.15 | 0.37 | -1.03 | 0.57 | -2.00 | 0.67 | 0.18 | 0.65 |
| O95168 | NADH dehydrogenase [ubiquinone] 1 beta subcomplex subunit 4 | NDUFB4 | 0.04 | 0.06 | -0.15 | -0.38 | -0.64 | -0.43 | 1.52 | -0.40 | -0.25 | -1.05 | -0.32 | 2.12 |
| O15231 | Zinc finger protein 185 | ZNF185 | 0.04 | 0.04 | -0.29 | 1.67 | -0.55 | -0.41 | -0.48 | -0.36 | -0.37 | -0.78 | -0.49 | 2.08 |
| A2RRP1 | Neuroblastoma-amplified sequence | NBAS | 0.04 | 0.10 | 0.82 | -0.91 | -0.81 | 1.24 | -0.48 | 0.12 | 0.25 | -1.83 | 1.22 | 0.40 |
| Q15147 | 1-phosphatidylinositol 4,5-bisphosphate phosphodiesterase beta-4 | PLCB4 | 0.03 | 0.04 | 1.26 | -1.94 | 0.57 | -0.55 | 0.61 | 0.84 | 0.45 | -1.24 | 0.32 | -0.31 |
| P55290 | Cadherin-13 | CDH13 | 0.03 | 0.03 | -2.06 | 0.46 | 0.12 | 0.69 | 0.74 | -1.00 | -0.97 | 0.33 | 0.74 | 0.94 |
| P15927 | Replication protein A 32 kDa subunit | RPA2 | 0.03 | 0.10 | 1.02 | 0.05 | -0.18 | -0.24 | -0.79 | 0.41 | -0.58 | -1.99 | 0.91 | 1.39 |
| Q13561 | Dynactin subunit 2 | DCTN2 | 0.03 | 0.09 | 0.57 | 1.36 | -1.12 | -0.96 | 0.03 | -0.06 | 1.64 | -1.14 | -0.71 | 0.41 |
| Q13769 | THO complex subunit 5 homolog | THOC5 | 0.03 | 0.06 | -0.43 | -0.59 | -0.80 | -0.45 | 2.18 | -0.48 | -0.77 | 0.89 | 0.99 | -0.55 |
| Q8NHU6 | Tudor domain-containing protein 7 | TDRD7 | 0.03 | 0.05 | -0.57 | 0.85 | -1.17 | 1.83 | -1.01 | -0.71 | -0.88 | 0.62 | 0.58 | 0.45 |
| Q9UPW5 | Cytosolic carboxypeptidase 1 | AGTPBP1 | 0.03 | 0.08 | -0.62 | -0.07 | 0.16 | 0.24 | 0.18 | -1.35 | 2.51 | -0.30 | -0.37 | -0.37 |
| Q9BR01 | Sulfotransferase 4A1 | SULT4A1 | 0.03 | 0.06 | -0.75 | -0.70 | -0.55 | 0.66 | 1.25 | 0.39 | -0.57 | 1.06 | 0.96 | -1.76 |
| Q9NUQ9 | Protein FAM49B | FAM49B | 0.03 | 0.06 | 0.64 | 0.17 | 0.13 | -0.48 | -0.54 | -1.30 | 0.45 | 2.25 | -0.32 | -0.99 |
| Q9Y4C0 | Neurexin-3 | NRXN3 | 0.03 | 0.04 | -1.02 | 1.08 | 1.22 | -0.40 | -0.94 | -1.83 | 0.47 | 0.30 | 0.60 | 0.52 |
| Q9UBI9 | Headcase protein homolog | HECA | 0.03 | 0.03 | 0.05 | -1.96 | 0.18 | 0.84 | 0.85 | -1.30 | 0.19 | 0.83 | 0.94 | -0.62 |
| P52757 | Beta-chimaerin | CHN2 | 0.03 | 0.03 | -0.24 | 0.59 | -1.67 | 0.63 | 0.65 | -1.43 | -0.23 | 1.59 | -0.37 | 0.49 |
| Q9NZB2 | Constitutive coactivator of PPAR-gamma-like protein 1 | FAM120A | 0.03 | 0.03 | -0.90 | -0.40 | 1.79 | -0.07 | -0.46 | -0.61 | 1.91 | -0.17 | -0.45 | -0.62 |
| Q9NWW6 | Nicotinamide riboside kinase 1 | NMRK1 | 0.03 | 0.03 | 1.73 | -0.46 | -0.58 | -0.29 | -0.44 | 2.03 | -0.42 | -0.74 | -0.45 | -0.37 |
| Q13153 | Serine/threonine-protein kinase PAK 1 | PAK1 | 0.03 | 0.08 | -0.29 | 1.21 | -0.78 | -0.79 | 0.55 | -1.36 | 1.44 | 1.14 | -0.40 | -0.71 |
| Q9Y5Z4 | Heme-binding protein 2 | HEBP2 | 0.03 | 0.03 | -1.18 | 0.87 | -0.51 | 0.53 | 0.25 | 0.54 | -2.20 | 0.51 | 0.33 | 0.86 |
| Q9H2X9 | Solute carrier family 12 member 5 | SLC12A5 | 0.03 | 0.04 | 1.05 | -1.86 | -0.08 | 0.74 | 0.09 | -0.05 | 1.36 | -1.21 | 0.52 | -0.55 |
| P11532 | Dystrophin | DMD | 0.03 | 0.05 | -1.05 | 1.47 | -0.17 | 0.32 | -0.64 | -0.50 | -1.06 | -0.68 | 0.57 | 1.74 |
| Q9Y2H9 | Microtubule-associated serine/threonine-protein kinase 1 | MAST1 | 0.03 | 0.06 | -0.09 | 0.42 | -2.32 | 1.02 | 0.88 | -0.33 | -0.80 | 0.06 | 0.86 | 0.31 |
| P56192 | Methionine--tRNA ligase, cytoplasmic | MARS | 0.03 | 0.13 | -0.29 | 0.81 | -1.14 | 0.66 | -0.23 | -1.25 | -0.58 | -0.50 | 0.50 | 2.02 |
| P42766 | 60S ribosomal protein L35 | RPL35 | 0.03 | 0.04 | -0.55 | -1.53 | 0.77 | -0.41 | 1.66 | -1.02 | 0.69 | 0.32 | -0.75 | 0.82 |
| O75157 | TSC22 domain family protein 2 | TSC22D2 | 0.03 | 0.02 | 0.20 | 0.76 | -2.37 | 0.90 | 0.47 | 0.33 | 0.09 | -1.09 | 0.68 | 0.03 |
| O00148 | ATP-dependent RNA helicase DDX39A | DDX39A | 0.03 | 0.03 | 1.16 | -1.71 | 1.55 | -0.08 | -0.97 | 0.26 | -1.04 | 0.26 | 0.30 | 0.27 |
| Q8WXE9 | Stonin-2 | STON2 | 0.03 | 0.02 | 0.86 | -0.58 | -0.94 | 0.59 | 0.05 | 0.59 | 0.84 | -2.26 | 0.74 | 0.11 |
| O00232 | 26S proteasome non-ATPase regulatory subunit 12 | PSMD12 | 0.02 | 0.07 | 0.09 | 1.50 | -1.21 | -0.42 | -0.06 | 0.37 | 0.27 | -1.96 | 0.79 | 0.65 |
| Q8WWI5 | Choline transporter-like protein 1 | SLC44A1 | 0.02 | 0.03 | 0.12 | -1.12 | 0.94 | 0.37 | -0.37 | -1.61 | 0.46 | 1.69 | 0.38 | -0.88 |
| Q8N8A6 | ATP-dependent RNA helicase DDX51 | DDX51 | 0.02 | 0.03 | 0.56 | -1.26 | 0.23 | 0.02 | 0.40 | -1.13 | 0.88 | -1.60 | 0.45 | 1.44 |
| Q15785 | Mitochondrial import receptor subunit TOM34 | TOMM34 | 0.02 | 0.03 | 0.95 | -0.10 | -0.56 | 0.48 | -0.81 | 0.40 | 0.70 | -2.31 | 0.40 | 0.85 |
| P62316 | Small nuclear ribonucleoprotein Sm D2 | SNRPD2 | 0.02 | 0.04 | 1.99 | -1.25 | 0.28 | -0.52 | -0.56 | 1.17 | -1.13 | -0.01 | -0.30 | 0.32 |
| Q6PJI9 | GATOR complex protein WDR59 | WDR59 | 0.02 | 0.02 | -1.13 | -0.14 | 0.80 | 0.62 | -0.18 | -1.31 | -1.33 | 0.26 | 0.91 | 1.51 |
| Q9BQS8 | FYVE and coiled-coil domain-containing protein 1 | FYCO1 | 0.02 | 0.02 | 1.27 | 0.05 | -1.01 | -0.03 | -0.30 | 0.89 | 1.59 | -1.61 | -0.37 | -0.46 |
| Q13367 | AP-3 complex subunit beta-2 | AP3B2 | 0.02 | 0.08 | -0.24 | -0.55 | -1.18 | 0.41 | 1.44 | -0.55 | -0.32 | 1.65 | 0.58 | -1.24 |
| P14920 | D-amino-acid oxidase | DAO | 0.02 | 0.03 | -0.88 | 0.04 | 1.38 | 0.66 | -1.25 | -1.06 | 0.86 | 0.33 | 1.04 | -1.13 |
| Q9P0U4 | CXXC-type zinc finger protein 1 | CXXC1 | 0.02 | 0.02 | 1.46 | -0.67 | -0.74 | 0.56 | -0.65 | -0.66 | 1.77 | -0.97 | 0.49 | -0.61 |
| Q9UPZ9 | Serine/threonine-protein kinase ICK | ICK | 0.02 | 0.03 | -0.38 | -0.51 | 1.94 | -0.49 | -0.60 | -0.38 | -0.42 | 1.84 | -0.57 | -0.44 |
| Q9BXP5 | Serrate RNA effector molecule homolog | SRRT | 0.02 | 0.03 | 0.93 | 0.35 | -1.28 | 0.00 | -0.05 | 0.86 | 0.93 | -2.17 | 0.22 | 0.21 |
| O00213 | Amyloid-beta A4 precursor protein-binding family B member 1 | APBB1 | 0.02 | 0.04 | -0.05 | 0.97 | -1.08 | 0.53 | -0.44 | 0.85 | -0.28 | -2.03 | 0.36 | 1.17 |
| P42224 | Signal transducer and activator of transcription 1-alpha/beta | STAT1 | 0.02 | 0.03 | 1.31 | 0.27 | -1.96 | 0.43 | -0.09 | 0.11 | 0.52 | -1.55 | 0.56 | 0.40 |
| Q9Y3E7 | Charged multivesicular body protein 3 | CHMP3 | 0.02 | 0.03 | -1.54 | -0.23 | 1.02 | 0.64 | 0.08 | -1.03 | -1.38 | 0.54 | 1.08 | 0.84 |
| Q15075 | Early endosome antigen 1 | EEA1 | 0.02 | 0.05 | 0.97 | 0.48 | 0.19 | -2.22 | 0.51 | -0.11 | 1.15 | 0.45 | -0.89 | -0.54 |
| P22830 | Ferrochelatase, mitochondrial | FECH | 0.02 | 0.03 | -1.51 | 0.46 | 0.76 | 0.88 | -0.65 | -1.29 | -0.78 | 0.12 | 0.53 | 1.46 |
| Q13416 | Origin recognition complex subunit 2 | ORC2 | 0.02 | 0.02 | 1.24 | -0.66 | -0.79 | 0.86 | -0.68 | -0.61 | -0.65 | 2.01 | -0.60 | -0.12 |
| Q7LG56 | Ribonucleoside-diphosphate reductase subunit M2 B | RRM2B | 0.02 | 0.03 | -0.40 | 0.08 | -0.70 | 0.72 | 0.25 | 0.20 | -1.71 | -1.08 | 1.05 | 1.59 |

| | | | | | | | | | | | | | | |
|---|---|---|---|---|---|---|---|---|---|---|---|---|---|---|
| Q7Z6J4 | FYVE, RhoGEF and PH domain-containing protein 2 | FGD2 | 0.02 | 0.03 | -0.43 | -0.93 | -1.05 | 0.84 | 1.54 | -0.80 | -0.75 | 1.52 | 0.38 | -0.32 |
| Q13033 | Striatin-3 | STRN3 | 0.02 | 0.01 | 0.95 | 0.15 | -1.62 | -0.16 | 0.66 | 1.21 | 0.74 | -1.72 | -0.12 | -0.09 |
| O15031 | Plexin-B2 | PLXNB2 | 0.02 | 0.01 | 0.49 | 1.03 | -1.22 | 0.29 | -0.62 | 1.46 | -1.06 | -1.39 | 0.64 | 0.37 |
| P05067 | Amyloid-beta precursor protein | APP | 0.02 | 0.01 | -0.17 | -0.37 | -0.71 | -1.02 | 2.25 | -0.91 | 0.87 | 0.60 | 0.00 | -0.52 |
| Q13057 | Bifunctional coenzyme A synthase | COASY | 0.02 | 0.03 | 0.91 | -0.22 | -0.77 | 0.43 | -0.40 | 1.42 | -0.05 | -2.17 | 0.17 | 0.67 |
| O14936 | Peripheral plasma membrane protein CASK | CASK | 0.02 | 0.03 | -2.18 | 1.37 | 0.30 | 0.76 | -0.28 | -0.12 | -0.73 | -0.31 | 0.97 | 0.23 |
| Q9H9T3 | Elongator complex protein 3 | ELP3 | 0.02 | 0.02 | -0.53 | 1.06 | -0.91 | 1.09 | -0.74 | -0.62 | -0.75 | -1.05 | 1.25 | 1.19 |
| Q8N5S9 | Calcium/calmodulin-dependent protein kinase kinase 1 | CAMKK1 | 0.02 | 0.03 | 0.43 | -2.03 | 1.25 | 0.32 | -0.02 | -0.66 | -0.79 | 0.99 | 0.91 | -0.40 |
| P61978 | Heterogeneous nuclear ribonucleoprotein K | HNRNPK | 0.02 | 0.06 | 2.06 | -0.12 | -1.51 | -0.26 | -0.24 | -0.08 | -0.38 | -0.91 | 0.33 | 1.12 |
| P54652 | Heat shock-related 70 kDa protein 2 | HSPA2 | 0.02 | 0.07 | -0.99 | 0.18 | 0.01 | 0.66 | 0.04 | -0.95 | -1.79 | 0.23 | 1.27 | 1.33 |
| Q86VM9 | Zinc finger CCCH domain-containing protein 18 | ZC3H18 | 0.02 | 0.03 | 0.47 | -1.29 | -1.50 | 0.48 | 1.78 | -0.32 | -0.78 | 0.27 | 0.04 | 0.82 |
| P33176 | Kinesin-1 heavy chain | KIF5B | 0.01 | 0.06 | 1.88 | -0.53 | 0.46 | -0.62 | -1.28 | 1.33 | -0.24 | 0.35 | -0.89 | -0.47 |
| P63173 | 60S ribosomal protein L38 | RPL38 | 0.01 | 0.02 | -0.17 | -2.05 | 0.09 | 0.82 | 1.28 | 0.30 | -0.87 | -0.74 | 0.38 | 0.97 |
| P46781 | 40S ribosomal protein S9 | RPS9 | 0.01 | 0.04 | -0.02 | -1.97 | 1.65 | 0.28 | 0.01 | -0.97 | 0.68 | -0.66 | 0.50 | 0.51 |
| Q8NCA5 | Protein FAM98A | FAM98A | 0.01 | 0.01 | 1.52 | -0.74 | -1.04 | -0.72 | 0.96 | 1.11 | -0.75 | -1.10 | -0.03 | 0.80 |
| P10155 | 60 kDa SS-A/Ro ribonucleoprotein | RO60 | 0.01 | 0.01 | -1.46 | 0.32 | 0.65 | 0.24 | 0.23 | -0.89 | -1.59 | 1.57 | 0.58 | 0.35 |
| Q8TCE6 | DENN domain-containing protein 10 | DENND10 | 0.01 | 0.02 | -1.04 | 0.74 | -0.72 | 1.46 | -0.46 | -0.65 | -0.79 | -0.81 | 1.46 | 0.82 |
| P53999 | Activated RNA polymerase II transcriptional coactivator p15 | SUB1 | 0.01 | 0.01 | -0.64 | -0.64 | 0.27 | 0.67 | 0.32 | -1.61 | -0.87 | 2.01 | 0.42 | 0.07 |
| P54725 | UV excision repair protein RAD23 homolog A | RAD23A | 0.01 | 0.01 | -0.69 | -0.80 | -0.85 | 1.40 | 0.93 | -0.72 | -0.81 | 1.19 | 1.10 | -0.74 |
| Q7Z4V5 | Hepatoma-derived growth factor-related protein 2 | HDGFL2 | 0.01 | 0.02 | 1.73 | 0.10 | 0.19 | -0.21 | -1.84 | -0.52 | 1.09 | -0.24 | -0.85 | 0.55 |
| Q1L5Z9 | LON peptidase N-terminal domain and RING finger protein 2 | LONRF2 | 0.01 | 0.01 | 1.95 | -0.13 | -0.46 | 0.08 | -1.45 | 0.77 | 0.58 | 0.02 | 0.01 | -1.38 |
| O60927 | E3 ubiquitin-protein ligase PPP1R11 | PPP1R11 | 0.01 | 0.01 | -0.15 | 0.12 | 1.60 | -1.49 | -0.09 | -0.33 | -0.08 | 0.75 | -1.48 | 1.15 |
| Q96EM0 | Trans-3-hydroxy-L-proline dehydratase | L3HYPDH | 0.01 | 0.01 | 0.54 | -0.90 | -1.00 | 0.99 | 0.36 | -0.86 | -0.88 | 1.45 | 1.18 | -0.88 |
| Q9UNF1 | Melanoma-associated antigen D2 | MAGED2 | 0.01 | 0.01 | -0.80 | 1.11 | 0.05 | 1.13 | -1.50 | -0.25 | -0.30 | -1.30 | 0.93 | 0.93 |
| Q01484 | Ankyrin-2 | ANK2 | 0.01 | 0.01 | -1.35 | 0.77 | 0.22 | 0.47 | -0.12 | -1.02 | -1.63 | 1.15 | 0.55 | 0.97 |
| Q8N584 | Tetratricopeptide repeat protein 39C | TTC39C | 0.01 | 0.01 | 0.65 | 0.15 | 0.46 | -0.10 | -1.18 | 0.68 | 1.22 | 0.26 | 0.08 | -2.22 |
| P40692 | DNA mismatch repair protein Mlh1 | MLH1 | 0.01 | 0.00 | 1.21 | -1.36 | -0.50 | 0.41 | 0.24 | 0.98 | 1.16 | -1.53 | 0.14 | -0.75 |
| P24298 | Alanine aminotransferase 1 | GPT | 0.01 | 0.01 | 1.77 | -1.97 | -0.58 | 0.49 | 0.27 | -0.58 | 0.79 | -0.38 | 0.42 | -0.24 |
| P78357 | Contactin-associated protein 1 | CNTNAP1 | 0.01 | 0.01 | 0.80 | 0.57 | 0.24 | 0.29 | -1.92 | -1.58 | -0.44 | 0.41 | 0.76 | 0.88 |
| O75489 | NADH dehydrogenase [ubiquinone] iron-sulfur protein 3, mitochon | NDUFS3 | 0.01 | 0.01 | 1.08 | -1.22 | 1.46 | -1.20 | -0.12 | 0.47 | -1.30 | -0.33 | 0.40 | 0.77 |
| Q9P2X3 | Protein IMPACT | IMPACT | 0.01 | 0.01 | 0.27 | -1.44 | 0.48 | 1.01 | -0.34 | 0.72 | 0.96 | -1.99 | 0.21 | 0.12 |
| Q13564 | NEDD8-activating enzyme E1 regulatory subunit | NAE1 | 0.01 | 0.02 | 1.01 | -0.56 | 0.41 | 1.15 | -2.03 | 1.03 | -0.94 | 0.11 | 0.09 | -0.27 |
| Q9H0A0 | RNA cytidine acetyltransferase | NAT10 | 0.00 | 0.00 | -1.30 | 0.43 | 0.19 | 0.01 | 0.67 | -1.33 | -1.39 | 1.17 | 0.33 | 1.21 |
| Q6ICG6 | Uncharacterized protein KIAA0930 | KIAA0930 | 0.00 | 0.00 | -0.58 | -0.76 | -0.81 | 0.97 | 1.18 | -0.69 | -0.66 | -1.08 | 1.36 | 1.08 |
| Q92930 | Ras-related protein Rab-8B | RAB8B | 0.00 | 0.02 | 0.34 | 0.01 | 0.62 | -0.28 | -0.72 | 1.51 | 1.25 | -1.67 | 0.08 | -1.12 |
| Q92797 | Symplekin | SYMPK | 0.00 | 0.01 | 1.51 | -0.68 | -0.87 | 0.74 | -0.70 | 0.58 | -0.66 | -1.25 | -0.13 | 1.45 |
| Q8WWI1 | LIM domain only protein 7 | LMO7 | 0.00 | 0.00 | 1.68 | -0.07 | -0.76 | -0.05 | -0.81 | 0.54 | 0.78 | -1.96 | 0.36 | 0.29 |
| P21283 | V-type proton ATPase subunit C 1 | ATP6V1C1 | 0.00 | 0.01 | 0.21 | 1.62 | -0.43 | 0.21 | -1.62 | -1.26 | -0.68 | 0.62 | 0.37 | 0.95 |
| Q14974 | Importin subunit beta-1 | KPNB1 | 0.00 | 0.01 | -1.47 | 1.49 | 0.41 | 0.27 | -0.72 | 0.87 | -0.63 | -1.38 | 0.30 | 0.84 |
| Q9BVM2 | Protein DPCD | DPCD | 0.00 | 0.00 | 0.68 | 0.32 | -1.78 | 0.56 | 0.21 | 0.45 | 0.07 | -1.92 | 0.61 | 0.79 |
| Q96BF6 | Nucleus accumbens-associated protein 2 | NACC2 | 0.00 | 0.00 | -0.32 | -0.49 | -0.61 | -0.49 | 1.91 | -0.46 | -0.50 | 1.87 | -0.44 | -0.46 |
| Q9NQ66 | 1-phosphatidylinositol 4,5-bisphosphate phosphodiesterase beta-1 | PLCB1 | 0.00 | 0.00 | -0.93 | 0.37 | 0.43 | 0.89 | -0.76 | -1.93 | -0.58 | 0.79 | 1.25 | 0.47 |
| Q96BY7 | Autophagy-related protein 2 homolog B | ATG2B | 0.00 | 0.00 | 0.77 | 0.42 | 0.65 | -0.61 | -1.23 | -0.46 | -0.07 | -1.15 | -0.39 | 2.06 |
| Q07889 | Son of sevenless homolog 1 | SOS1 | 0.00 | 0.00 | 0.39 | 0.66 | -2.31 | 0.79 | 0.46 | 0.21 | -0.03 | -1.17 | 0.91 | 0.08 |
| Q9BPW8 | Protein NipSnap homolog 1 | NIPSNAP1 | 0.00 | 0.00 | 0.50 | -0.73 | 1.64 | 0.17 | -1.58 | -1.41 | 0.41 | -0.01 | 0.11 | 0.91 |
| O00499 | Myc box-dependent-interacting protein 1 | BIN1 | 0.00 | 0.00 | 0.38 | 0.25 | -0.33 | 0.02 | -0.32 | -1.91 | 0.81 | 1.63 | 0.58 | -1.12 |
| Q86UL8 | Membrane-associated guanylate kinase, WW and PDZ domain-con | MAGI2 | 0.00 | 0.00 | 0.41 | 1.00 | -1.70 | 0.10 | 0.19 | -0.01 | 1.19 | -1.76 | 0.62 | -0.04 |
| P31689 | DnaJ homolog subfamily A member 1 | DNAJA1 | 0.00 | 0.00 | -0.91 | 0.32 | 1.56 | 0.75 | -1.71 | -1.26 | 0.08 | 0.14 | 0.48 | 0.55 |
| Q8NFW8 | N-acylneuraminate cytidylyltransferase | CMAS | 0.00 | 0.00 | 1.57 | -2.21 | -0.20 | 0.76 | 0.09 | -0.33 | -0.21 | 0.63 | 0.43 | -0.53 |
| Q9NR22 | Protein arginine N-methyltransferase 8 | PRMT8 | 0.00 | 0.00 | 0.29 | 0.29 | -1.62 | 0.83 | 0.22 | -0.47 | 0.07 | -1.72 | 0.89 | 1.22 |
| P08708 | 40S ribosomal protein S17 | RPS17 | 0.00 | 0.00 | 0.88 | -0.45 | -0.52 | -0.28 | 0.37 | 1.21 | 1.70 | -0.58 | -1.42 | -0.92 |
| O14972 | Vacuolar protein sorting-associated protein 26C | VPS26C | 0.00 | 0.00 | 1.59 | -0.30 | -1.38 | 0.15 | -0.05 | 1.60 | -1.22 | -0.51 | -0.18 | 0.31 |
| Q9BT73 | Proteasome assembly chaperone 3 | PSMG3 | 0.00 | 0.01 | 0.09 | -0.43 | -0.75 | 1.36 | -0.25 | -0.16 | -0.42 | -1.13 | -0.46 | 2.16 |
| O60282 | Kinesin heavy chain isoform 5C | KIF5C | 0.00 | 0.00 | 0.05 | 1.93 | -0.54 | -0.08 | -1.34 | 0.59 | 0.99 | -1.30 | -0.39 | 0.09 |
| Q6PCE3 | Glucose 1,6-bisphosphate synthase | PGM2L1 | 0.00 | 0.01 | 1.48 | -0.90 | -0.37 | -0.22 | 0.02 | 1.62 | 0.15 | -0.10 | 0.11 | -1.78 |
| P30740 | Leukocyte elastase inhibitor | SERPINB1 | 0.00 | 0.01 | -1.27 | 0.37 | 0.28 | -0.16 | 0.79 | -1.10 | -1.49 | 1.47 | 0.16 | 0.96 |
| Q9BT78 | COP9 signalosome complex subunit 4 | COPS4 | 0.00 | 0.01 | 0.42 | 0.84 | -2.46 | 0.43 | 0.78 | 0.00 | 0.45 | 0.37 | -0.95 | 0.12 |
| P02042 | Hemoglobin subunit delta | HBD | -0.01 | 0.01 | -0.15 | -0.29 | 0.01 | 0.39 | 0.05 | -1.93 | -1.04 | 1.77 | 0.80 | 0.39 |
| O95503 | Chromobox protein homolog 6 | CBX6 | -0.01 | 0.01 | 1.27 | -0.70 | -0.77 | -0.64 | 0.84 | 0.71 | 1.64 | -1.05 | -0.78 | -0.53 |
| P36894 | Bone morphogenetic protein receptor type-1A | BMPR1A | -0.01 | 0.01 | -0.50 | 1.02 | -0.92 | -0.65 | 1.05 | 1.80 | -0.64 | -0.97 | -0.69 | 0.49 |
| P51693 | Amyloid-like protein 1 | APLP1 | -0.01 | 0.01 | -1.02 | 0.87 | 0.49 | 0.47 | -0.80 | -0.97 | -1.50 | 1.58 | 0.41 | 0.47 |
| Q676U5 | Autophagy-related protein 16-1 | ATG16L1 | -0.01 | 0.01 | -0.46 | -0.39 | 0.59 | 0.51 | -0.22 | -1.37 | -1.66 | 1.13 | 0.52 | 1.35 |
| P17096 | High mobility group protein HMG-I/HMG-Y | HMGA1 | -0.01 | 0.01 | -0.90 | 0.71 | 1.31 | 0.76 | -1.86 | -0.53 | -0.04 | -0.78 | 0.39 | 0.94 |
| Q9HBD1 | Roquin-2 | RC3H2 | -0.01 | 0.01 | 0.47 | 0.95 | -1.43 | 0.68 | -0.65 | 1.09 | -0.58 | -1.57 | 0.08 | 0.96 |
| P17301 | Integrin alpha-2 | ITGA2 | -0.01 | 0.06 | 0.90 | 0.92 | -1.47 | -0.04 | -0.22 | 0.46 | 0.81 | -2.00 | 0.16 | 0.49 |
| Q8NF50 | Dedicator of cytokinesis protein 8 | DOCK8 | -0.01 | 0.01 | -0.94 | -0.64 | -0.30 | 0.43 | 1.46 | 0.74 | -2.00 | 0.60 | 0.64 | 0.01 |
| Q13283 | Ras GTPase-activating protein-binding protein 1 | G3BP1 | -0.01 | 0.01 | -1.53 | 0.32 | 0.11 | 1.25 | -0.14 | -0.75 | -1.25 | -0.32 | 1.37 | 0.92 |
| P49585 | Choline-phosphate cytidylyltransferase A | PCYT1A | -0.01 | 0.01 | -1.14 | -0.35 | -0.98 | 1.23 | 1.25 | -1.58 | 0.45 | -0.05 | 0.87 | 0.29 |
| Q15366 | Poly(rC)-binding protein 2 | PCBP2 | -0.01 | 0.01 | 0.57 | -1.00 | -1.45 | 0.94 | 0.96 | 0.91 | -1.26 | -0.82 | 0.75 | 0.39 |
| Q7Z401 | C-myc promoter-binding protein | DENND4A | -0.01 | 0.01 | -0.26 | 1.72 | -0.54 | -0.42 | -0.49 | -0.33 | -0.39 | -0.86 | -0.44 | 2.02 |
| P52294 | Importin subunit alpha-5 | KPNA1 | -0.01 | 0.01 | 0.45 | 0.28 | -0.24 | -0.31 | -0.17 | 1.64 | 0.68 | -2.32 | -0.07 | 0.06 |
| O00305 | Voltage-dependent L-type calcium channel subunit beta-4 | CACNB4 | -0.01 | 0.01 | 0.24 | 1.44 | -1.53 | 1.00 | -1.13 | -1.16 | 0.89 | -0.10 | 0.34 | 0.01 |
| Q96MC5 | bMERB domain-containing protein 1 | BMERB1 | -0.01 | 0.01 | 0.67 | 1.40 | -1.22 | -1.00 | 0.17 | 0.72 | 1.08 | 0.33 | -1.12 | -1.02 |
| Q70Z53 | Protein FRA10AC1 | FRA10AC1 | -0.01 | 0.08 | 1.13 | -0.08 | -0.72 | 0.42 | -0.63 | -0.02 | 0.74 | -2.25 | 0.81 | 0.61 |
| Q8N302 | Angiogenic factor with G patch and FHA domains 1 | AGGF1 | -0.01 | 0.09 | 1.11 | -0.12 | -0.80 | 0.00 | -0.06 | 0.64 | 0.98 | -2.27 | -0.22 | 0.74 |
| Q92841 | Probable ATP-dependent RNA helicase DDX17 | DDX17 | -0.01 | 0.03 | 0.43 | -0.26 | -0.59 | 0.70 | -0.24 | -0.03 | -1.82 | -0.83 | 0.88 | 1.76 |
| Q9NQE9 | Histidine triad nucleotide-binding protein 3 | HINT3 | -0.01 | 0.01 | -1.50 | -0.08 | 0.44 | 0.86 | 0.29 | -1.23 | -1.28 | 1.16 | 1.08 | 0.25 |
| Q9H8H3 | Methyltransferase-like protein 7A | METTL7A | -0.01 | 0.02 | 0.75 | -0.16 | -1.45 | -0.72 | 1.61 | 0.40 | 0.84 | -1.31 | -0.54 | 0.59 |
| O15511 | Actin-related protein 2/3 complex subunit 5 | ARPC5 | -0.01 | 0.02 | 0.26 | 1.34 | -2.09 | 0.42 | 0.09 | 0.20 | 1.05 | -1.10 | -0.43 | 0.25 |
| Q13098 | COP9 signalosome complex subunit 1 | GPS1 | -0.02 | 0.03 | -2.30 | 0.80 | -0.18 | 0.65 | 1.07 | -1.04 | 0.29 | 0.34 | 0.44 | -0.08 |
| Q03468 | DNA excision repair protein ERCC-6 | ERCC6 | -0.02 | 0.12 | 1.03 | 0.41 | -1.00 | -0.07 | -0.19 | 0.40 | 0.82 | -2.33 | 0.50 | 0.43 |
| P18621 | 60S ribosomal protein L17 | RPL17 | -0.02 | 0.02 | 0.81 | -0.88 | 0.54 | -0.54 | 0.11 | 0.04 | 1.51 | -1.90 | -0.56 | 0.88 |
| A4D1U4 | DENN domain-containing protein 11 | DENND11 | -0.02 | 0.02 | 0.19 | -1.41 | -0.92 | 0.29 | 1.88 | -0.44 | -0.70 | -0.10 | 1.33 | -0.12 |

| | | | | | | | | | | | | | | |
|---|---|---|---|---|---|---|---|---|---|---|---|---|---|---|
| O60583 | Cyclin-T2 | CCNT2 | -0.02 | 0.12 | 1.16 | -0.35 | -1.56 | 0.63 | 0.29 | 0.52 | 0.42 | -1.95 | 0.52 | 0.33 |
| Q86Y82 | Syntaxin-12 | STX12 | -0.02 | 0.02 | -1.69 | 1.17 | -0.70 | -0.10 | 1.35 | -1.32 | 0.20 | 0.14 | 0.14 | 0.79 |
| Q9UHL0 | ATP-dependent RNA helicase DDX25 | DDX25 | -0.02 | 0.02 | 1.96 | -0.20 | -0.95 | 0.37 | -1.15 | 0.11 | 1.29 | -0.97 | -0.13 | -0.33 |
| Q5TA45 | Integrator complex subunit 11 | INTS11 | -0.02 | 0.01 | -0.63 | -0.73 | -0.84 | 1.26 | 0.95 | -0.72 | -0.65 | -1.00 | 0.90 | 1.45 |
| Q9BVL4 | Protein adenylyltransferase SelO, mitochondrial | SELENOO | -0.02 | 0.01 | 1.49 | -0.50 | -1.36 | -0.04 | 0.41 | 0.82 | 1.27 | -1.40 | -0.31 | -0.40 |
| O00635 | E3 ubiquitin-protein ligase TRIM38 | TRIM38 | -0.02 | 0.02 | -0.47 | 0.33 | -0.77 | 1.68 | -0.74 | -0.65 | -0.51 | -0.97 | 1.72 | 0.38 |
| Q96N66 | Lysophospholipid acyltransferase 7 | MBOAT7 | -0.02 | 0.05 | 0.20 | 1.68 | 0.02 | -0.03 | -1.80 | 0.80 | 0.18 | 0.30 | -1.46 | 0.10 |
| Q8N5A5 | Zinc finger CCCH-type with G patch domain-containing protein | ZGPAT | -0.02 | 0.02 | 1.43 | -0.57 | -1.00 | -0.85 | 1.02 | 1.10 | 0.10 | -1.19 | -0.82 | 0.77 |
| P84090 | Enhancer of rudimentary homolog | ERH | -0.02 | 0.02 | -0.30 | -0.41 | -0.68 | -0.51 | 1.94 | -0.39 | -0.49 | 1.84 | -0.50 | -0.50 |
| Q6ZT12 | E3 ubiquitin-protein ligase UBR3 | UBR3 | -0.02 | 0.03 | 0.11 | 1.20 | -1.04 | 0.00 | -0.23 | -1.30 | 2.02 | -0.79 | 0.11 | -0.09 |
| Q9BQ24 | Zinc finger FYVE domain-containing protein 21 | ZFYVE21 | -0.02 | 0.05 | 0.17 | -0.28 | 1.40 | 0.56 | -1.77 | -0.49 | -1.39 | 0.26 | 0.62 | 0.92 |
| Q8TF42 | Ubiquitin-associated and SH3 domain-containing protein B | UBASH3B | -0.02 | 0.02 | -0.36 | 1.27 | -0.98 | 0.94 | -0.84 | 0.87 | -1.11 | -1.25 | 0.56 | 0.91 |
| P05114 | Non-histone chromosomal protein HMG-14 | HMGN1 | -0.02 | 0.03 | 0.96 | 0.17 | 1.07 | 0.16 | -2.31 | -0.14 | 0.69 | -0.98 | 0.06 | 0.32 |
| O60486 | Plexin-C1 | PLXNC1 | -0.02 | 0.03 | 1.82 | -0.29 | -1.01 | -0.63 | 0.15 | 0.63 | 1.29 | -1.39 | -0.22 | -0.35 |
| Q8WYL5 | Protein phosphatase Slingshot homolog 1 | SSH1 | -0.02 | 0.02 | -1.02 | 1.06 | -1.26 | 0.21 | 1.04 | -1.07 | -1.05 | 1.13 | 0.72 | 0.24 |
| Q9NZD8 | Maspardin | SPG21 | -0.02 | 0.02 | 0.18 | 1.08 | -1.50 | 0.25 | 0.03 | 0.34 | -0.70 | -1.68 | 0.82 | 1.18 |
| P02766 | Transthyretin | TTR | -0.02 | 0.02 | 1.51 | 0.26 | -0.65 | -0.56 | -0.53 | -0.51 | -0.52 | 2.12 | -0.61 | -0.51 |
| Q5VUB5 | Protein FAM171A1 | FAM171A1 | -0.02 | 0.06 | -0.73 | -0.14 | 0.66 | 0.52 | -0.23 | -0.67 | 1.51 | -0.55 | 1.33 | -1.70 |
| C9J798 | Ras GTPase-activating protein 4B | RASA4B | -0.02 | 0.02 | 1.37 | -0.53 | -0.44 | -0.16 | -0.22 | 1.16 | 1.10 | -1.95 | 0.14 | -0.48 |
| P54578 | Ubiquitin carboxyl-terminal hydrolase 14 | USP14 | -0.02 | 0.07 | 0.10 | 1.95 | -0.78 | -0.14 | -1.02 | -0.05 | 1.05 | -1.53 | 0.24 | 0.19 |
| O15131 | Importin subunit alpha-6 | KPNA5 | -0.02 | 0.05 | -0.11 | -0.38 | -0.87 | 1.81 | -0.38 | -0.42 | -0.19 | -0.91 | 1.86 | -0.41 |
| Q15185 | Prostaglandin E synthase 3 | PTGES3 | -0.02 | 0.06 | 0.70 | -0.44 | -1.66 | 1.18 | 0.30 | 0.58 | -0.08 | -1.72 | 0.84 | 0.30 |
| Q9H115 | Beta-soluble NSF attachment protein | NAPB | -0.03 | 0.06 | 0.76 | -0.06 | -0.23 | 0.09 | -0.47 | 1.92 | 0.71 | -1.87 | -0.23 | -0.62 |
| Q8TDY4 | Arf-GAP with SH3 domain, ANK repeat and PH domain-containing | ASAP3 | -0.03 | 0.03 | 0.41 | -0.06 | -0.07 | -0.04 | -0.18 | 1.37 | -0.29 | -2.44 | 0.64 | 0.67 |
| P26992 | Ciliary neurotrophic factor receptor subunit alpha | CNTFR | -0.03 | 0.03 | -0.68 | 1.56 | 1.03 | -0.87 | -0.99 | -0.94 | -0.92 | 1.02 | 0.72 | 0.08 |
| Q5TCZ1 | SH3 and PX domain-containing protein 2A | SH3PXD2A | -0.03 | 0.03 | 0.07 | 1.28 | -1.86 | 0.90 | -0.35 | 0.02 | 0.03 | -1.43 | 0.73 | 0.61 |
| Q9UKD1 | Glucocorticoid modulatory element-binding protein 2 | GMEB2 | -0.03 | 0.18 | 1.03 | 0.54 | -0.85 | 0.01 | -0.47 | 0.85 | 0.83 | -2.27 | -0.07 | 0.40 |
| Q96MM6 | Heat shock 70 kDa protein 12B | HSPA12B | -0.03 | 0.14 | 0.80 | -0.04 | -0.92 | 0.31 | 0.04 | 0.43 | -0.12 | -2.17 | 0.07 | 1.58 |
| O95989 | Diphosphoinositol polyphosphate phosphohydrolase 1 | NUDT3 | -0.03 | 0.12 | 0.67 | -0.18 | 0.05 | 1.28 | -1.65 | -0.01 | -1.20 | -0.38 | 1.60 | -0.19 |
| Q92900 | Regulator of nonsense transcripts 1 | UPF1 | -0.03 | 0.02 | 1.54 | -0.50 | -1.82 | 0.29 | 0.51 | 1.23 | -0.28 | -1.02 | 0.20 | -0.16 |
| P35251 | Replication factor C subunit 1 | RFC1 | -0.03 | 0.02 | -0.04 | -0.44 | -1.01 | 0.80 | 0.72 | 1.59 | -0.84 | -1.10 | 1.18 | -0.87 |
| P13807 | Glycogen [starch] synthase, muscle | GYS1 | -0.03 | 0.05 | -0.94 | 0.40 | 0.35 | 1.23 | -0.98 | 0.84 | -0.71 | -1.73 | 0.71 | 0.81 |
| P57088 | Transmembrane protein 33 | TMEM33 | -0.03 | 0.03 | 1.04 | -0.36 | -1.78 | 0.73 | 0.42 | 0.86 | -1.15 | -1.01 | 0.65 | 0.62 |
| Q9BV86 | N-terminal Xaa-Pro-Lys N-methyltransferase 1 | NTMT1 | -0.03 | 0.27 | 1.45 | 0.13 | -1.44 | 0.08 | 0.16 | -0.05 | 0.10 | -1.91 | 0.87 | 0.62 |
| Q9H479 | Fructosamine-3-kinase | FN3K | -0.03 | 0.11 | 0.64 | -0.76 | 1.64 | 0.47 | -1.82 | -0.70 | -0.67 | -0.09 | 0.70 | 0.59 |
| O15083 | ERC protein 2 | ERC2 | -0.03 | 0.02 | -0.05 | -0.95 | 1.02 | -0.80 | 0.82 | 1.07 | 0.21 | -1.22 | -1.29 | 1.19 |
| P28074 | Proteasome subunit beta type-5 | PSMB5 | -0.03 | 0.06 | -1.38 | -0.70 | 0.48 | 0.47 | 1.22 | -1.40 | -0.84 | 1.26 | 0.60 | 0.29 |
| Q15596 | Nuclear receptor coactivator 2 | NCOA2 | -0.03 | 0.02 | 1.40 | -0.98 | -1.07 | -0.24 | 0.93 | 0.72 | 1.36 | -0.77 | -0.38 | -0.95 |
| Q9ULN7 | Paraneoplastic antigen-like protein 8B | PNMA8B | -0.03 | 0.04 | -1.00 | -1.05 | -0.41 | 0.61 | 1.91 | -1.12 | 0.09 | 0.79 | 0.72 | -0.54 |
| Q92540 | Protein SMG7 | SMG7 | -0.03 | 0.05 | -0.92 | -1.08 | -0.50 | 0.99 | 1.58 | 0.07 | 1.00 | -1.48 | 0.25 | 0.09 |
| Q9NZQ3 | NCK-interacting protein with SH3 domain | NCKIPSD | -0.03 | 0.04 | 1.12 | 0.75 | -1.74 | 0.71 | -0.78 | -0.60 | -0.79 | 1.21 | 0.60 | -0.48 |
| Q5MNZ6 | WD repeat domain phosphoinositide-interacting protein 3 | WDR45B | -0.03 | 0.03 | -0.66 | -0.25 | 0.50 | -0.78 | 1.24 | 0.18 | -0.75 | 2.07 | -0.80 | -0.75 |
| Q8TEH3 | DENN domain-containing protein 1A | DENND1A | -0.03 | 0.05 | -1.67 | -0.03 | 1.69 | -1.09 | 1.17 | -0.49 | -0.32 | 0.52 | 0.42 | -0.20 |
| Q86Y13 | E3 ubiquitin-protein ligase DZIP3 | DZIP3 | -0.03 | 0.26 | 1.10 | 0.90 | -1.37 | 0.17 | -0.44 | 0.35 | 0.57 | -2.03 | 0.47 | 0.28 |
| Q8IU81 | Interferon regulatory factor 2-binding protein 1 | IRF2BP1 | -0.03 | 0.03 | 1.93 | -0.88 | -0.82 | 0.56 | -0.74 | 0.76 | 0.37 | -1.00 | 0.71 | -0.89 |
| P01008 | Antithrombin-III | SERPINC1 | -0.03 | 0.12 | -0.70 | 1.71 | -0.12 | -0.63 | -0.09 | -0.69 | -1.48 | 1.54 | 0.26 | 0.20 |
| Q13586 | Stromal interaction molecule 1 | STIM1 | -0.04 | 0.05 | 0.04 | 2.19 | -0.83 | -0.65 | -0.68 | -0.65 | 0.85 | -1.06 | 0.52 | 0.27 |
| O43347 | RNA-binding protein Musashi homolog 1 | MSI1 | -0.04 | 0.04 | -0.23 | 0.87 | -0.25 | -1.83 | 1.51 | 0.40 | -0.51 | 0.05 | -1.02 | 1.02 |
| P68366 | Tubulin alpha-4A chain | TUBA4A | -0.04 | 0.09 | 1.23 | 0.84 | -2.09 | 0.62 | -0.47 | 0.05 | 0.13 | -1.10 | 0.77 | 0.02 |
| Q9Y490 | Talin-1 | TLN1 | -0.04 | 0.04 | -1.70 | 0.45 | -0.47 | 1.07 | 0.70 | -1.06 | -1.00 | 0.86 | 1.08 | 0.06 |
| Q8WYA6 | Beta-catenin-like protein 1 | CTNNBL1 | -0.04 | 0.04 | -0.45 | -0.96 | -0.15 | 0.80 | 0.82 | -1.09 | -0.12 | -1.33 | 0.75 | 1.73 |
| P17010 | Zinc finger X-chromosomal protein | ZFX | -0.04 | 0.06 | -2.07 | 0.11 | -0.01 | 0.82 | 1.24 | -1.36 | 0.03 | 0.36 | 0.67 | 0.22 |
| Q14156 | Protein EFR3 homolog A | EFR3A | -0.04 | 0.04 | 0.25 | 1.02 | 0.22 | 0.29 | -1.73 | 0.52 | 0.38 | -1.96 | 0.56 | 0.45 |
| Q9NT99 | Leucine-rich repeat-containing protein 4B | LRRC4B | -0.04 | 0.04 | 0.31 | -0.89 | 0.92 | 0.60 | -0.87 | -0.89 | -0.77 | 2.01 | 0.35 | -0.77 |
| Q6IBS0 | Twinfilin-2 | TWF2 | -0.04 | 0.08 | 0.16 | 0.45 | -1.24 | 0.49 | 0.26 | 0.07 | 1.55 | -2.09 | 0.36 | -0.01 |
| Q8IUC1 | Keratin-associated protein 11-1 | KRTAP11-1 | -0.04 | 0.05 | -0.16 | 1.81 | -0.58 | -0.53 | -0.47 | -0.54 | -0.53 | 1.96 | -0.50 | -0.46 |
| P43034 | Platelet-activating factor acetylhydrolase IB subunit alpha | PAFAH1B1 | -0.04 | 0.15 | 0.45 | -1.49 | 0.28 | 1.67 | -0.70 | -0.43 | 0.43 | -1.41 | 0.89 | 0.29 |
| Q9Y2S6 | Translation machinery-associated protein 7 | TMA7 | -0.04 | 0.10 | 0.67 | -0.53 | 1.83 | 0.09 | -1.92 | -0.42 | 0.06 | -0.75 | 0.41 | 0.56 |
| Q08379 | Golgin subfamily A member 2 | GOLGA2 | -0.04 | 0.03 | 1.01 | -0.57 | -0.33 | -0.92 | 0.87 | 0.80 | 1.21 | -1.43 | -1.21 | 0.58 |
| O95182 | NADH dehydrogenase [ubiquinone] 1 alpha subcomplex subunit 7 | NDUFA7 | -0.04 | 0.32 | 1.83 | 0.24 | -1.15 | 0.23 | -0.71 | 0.14 | 0.15 | -1.65 | -0.05 | 0.98 |
| Q6NUQ1 | RAD50-interacting protein 1 | RINT1 | -0.04 | 0.05 | 0.54 | 0.21 | 0.08 | 0.74 | -1.48 | -0.03 | -0.13 | -1.95 | 0.94 | 1.09 |
| Q06124 | Tyrosine-protein phosphatase non-receptor type 11 | PTPN11 | -0.04 | 0.18 | 0.92 | 0.90 | 0.66 | 0.33 | -2.54 | -0.05 | -0.06 | 0.11 | 0.23 | -0.50 |
| P62854 | 40S ribosomal protein S26 | RPS26 | -0.04 | 0.06 | -1.28 | -0.99 | 0.43 | -0.15 | 2.07 | -0.94 | -0.15 | 0.28 | -0.21 | 0.94 |
| Q5TC12 | ATP synthase mitochondrial F1 complex assembly factor 1 | ATPAF1 | -0.04 | 0.03 | 1.12 | -0.09 | -0.87 | 0.21 | -0.32 | 1.16 | 1.23 | -1.93 | 0.01 | -0.52 |
| Q9UBE0 | SUMO-activating enzyme subunit 1 | SAE1 | -0.04 | 0.08 | 1.08 | -1.08 | -0.18 | -0.75 | 1.06 | 1.35 | 0.92 | -1.16 | -0.89 | -0.35 |
| A7XYQ1 | Sine oculis-binding protein homolog | SOBP | -0.04 | 0.43 | 1.33 | 0.24 | -1.10 | -0.10 | 0.19 | 0.73 | -0.03 | -2.22 | 0.51 | 0.44 |
| O75112 | LIM domain-binding protein 3 | LDB3 | -0.04 | 0.05 | -0.23 | -0.42 | -0.57 | 1.78 | -0.51 | -0.43 | -0.35 | -0.78 | 1.97 | -0.47 |
| P50148 | Guanine nucleotide-binding protein G(q) subunit alpha | GNAQ | -0.04 | 0.14 | -0.28 | 1.43 | 0.00 | 0.46 | -1.40 | 0.68 | -0.07 | -1.88 | 0.82 | 0.24 |
| P83731 | 60S ribosomal protein L24 | RPL24 | -0.04 | 0.11 | 0.41 | -0.87 | 0.54 | -0.36 | 0.43 | -0.43 | 1.46 | -1.69 | -0.78 | 1.30 |
| Q96N96 | Spermatogenesis-associated protein 13 | SPATA13 | -0.04 | 0.05 | -0.58 | 1.45 | -0.78 | 0.80 | -0.81 | -0.75 | -0.66 | -0.97 | 0.98 | 1.32 |
| Q96LR5 | Ubiquitin-conjugating enzyme E2 E2 | UBE2E2 | -0.04 | 0.36 | 1.16 | 0.44 | -1.67 | 0.70 | -0.14 | 0.62 | 0.05 | -1.86 | 0.20 | 0.52 |
| O60610 | Protein diaphanous homolog 1 | DIAPH1 | -0.04 | 0.05 | -1.43 | 1.14 | -0.50 | 0.69 | 0.17 | -1.62 | 1.24 | -0.49 | 0.70 | 0.10 |
| Q04637 | Eukaryotic translation initiation factor 4 gamma 1 | EIF4G1 | -0.04 | 0.15 | 0.33 | 1.37 | -0.35 | 0.44 | -1.58 | 0.33 | 0.86 | -1.71 | -0.28 | 0.59 |
| Q15631 | Translin | TSN | -0.04 | 0.14 | 1.68 | -1.18 | 1.13 | 0.18 | -1.60 | -0.71 | 0.28 | 0.50 | -0.28 | 0.01 |
| P50897 | Palmitoyl-protein thioesterase 1 | PPT1 | -0.04 | 0.06 | 0.10 | 0.49 | -0.55 | -1.30 | 1.35 | -0.68 | 1.80 | 0.01 | -1.10 | -0.10 |
| Q13162 | Peroxiredoxin-4 | PRDX4 | -0.05 | 0.06 | -1.16 | 1.38 | -0.71 | 0.41 | 0.16 | 1.26 | 0.26 | -0.79 | 0.71 | -1.51 |
| Q9H8Y8 | Golgi reassembly-stacking protein 2 | GORASP2 | -0.05 | 0.06 | 0.58 | 0.21 | -0.73 | 0.30 | -0.26 | 0.50 | 0.39 | -2.46 | 0.31 | 1.17 |
| Q0VDF9 | Heat shock 70 kDa protein 14 | HSPA14 | -0.05 | 0.05 | -0.16 | 0.25 | -0.64 | 1.04 | -0.42 | -1.45 | -1.45 | 1.35 | 0.96 | 0.52 |
| O94916 | Nuclear factor of activated T-cells 5 | NFAT5 | -0.05 | 0.04 | 0.31 | -0.92 | -1.08 | 0.79 | 0.97 | 1.48 | 0.78 | -1.21 | -0.08 | -1.03 |
| P08235 | Mineralocorticoid receptor | NR3C2 | -0.05 | 0.14 | -0.08 | -0.25 | -0.59 | 1.74 | -0.62 | 1.82 | -0.23 | -1.23 | -0.54 | -0.02 |
| Q9BYB4 | Guanine nucleotide-binding protein subunit beta-like protein 1 | GNB1L | -0.05 | 0.06 | 0.59 | -0.39 | -1.33 | 0.29 | 0.93 | 1.29 | 0.70 | -1.54 | 0.48 | -1.02 |
| P38405 | Guanine nucleotide-binding protein G(olf) subunit alpha | GNAL | -0.05 | 0.06 | 1.96 | -0.41 | -0.61 | -0.32 | -0.53 | 1.77 | -0.36 | -0.87 | -0.37 | -0.26 |
| P06241 | Tyrosine-protein kinase Fyn | FYN | -0.05 | 0.11 | -0.43 | 1.51 | -0.76 | 0.34 | -0.51 | 0.96 | -0.09 | -2.02 | 0.33 | 0.67 |

| | | | | | | | | | | | | | | |
|---|---|---|---|---|---|---|---|---|---|---|---|---|---|---|
| Q9UBN7 | Histone deacetylase 6 | HDAC6 | -0.05 | 0.08 | 0.23 | 1.14 | -1.01 | 0.35 | -0.58 | 0.17 | -0.22 | -2.04 | 0.92 | 1.05 |
| Q0VDG4 | Secernin-3 | SCRN3 | -0.05 | 0.07 | 2.15 | -0.54 | -0.68 | -0.33 | -0.51 | -0.54 | -0.40 | 1.59 | -0.42 | -0.33 |
| Q9BQ39 | ATP-dependent RNA helicase DDX50 | DDX50 | -0.05 | 0.06 | 1.31 | -0.91 | -1.06 | 0.21 | 0.54 | 0.34 | 1.79 | -0.55 | -0.85 | -0.81 |
| Q9HC52 | Chromobox protein homolog 8 | CBX8 | -0.05 | 0.05 | -0.59 | -0.62 | -0.95 | 0.59 | 1.64 | -0.64 | -0.58 | -1.07 | 1.19 | 1.01 |
| Q13185 | Chromobox protein homolog 3 | CBX3 | -0.05 | 0.12 | -0.15 | -0.38 | 0.69 | -0.05 | 0.07 | 1.02 | -2.50 | -0.10 | 0.52 | 0.88 |
| Q99614 | Tetratricopeptide repeat protein 1 | TTC1 | -0.05 | 0.05 | 0.67 | -0.70 | -0.86 | -0.66 | 1.62 | -0.77 | 0.98 | 1.24 | -0.74 | -0.78 |
| Q9UNX3 | 60S ribosomal protein L26-like 1 | RPL26L1 | -0.05 | 0.18 | -0.30 | -0.80 | 1.25 | -0.14 | 0.25 | -1.08 | 0.21 | 0.39 | -1.51 | 1.73 |
| Q5T200 | Zinc finger CCCH domain-containing protein 13 | ZC3H13 | -0.05 | 0.37 | 1.42 | 0.47 | -1.04 | 0.26 | -0.62 | 0.46 | 0.56 | -2.08 | -0.06 | 0.62 |
| P32119 | Peroxiredoxin-2 | PRDX2 | -0.05 | 0.06 | -0.94 | -0.07 | -0.19 | -0.26 | 1.55 | -0.68 | -0.90 | 2.03 | -0.46 | -0.07 |
| Q8WU79 | Stromal membrane-associated protein 2 | SMAP2 | -0.05 | 0.08 | 1.42 | 0.13 | -1.14 | 0.04 | -0.34 | 1.14 | 1.10 | -1.44 | 0.08 | -0.98 |
| Q9UNE7 | E3 ubiquitin-protein ligase CHIP | STUB1 | -0.05 | 0.08 | 0.00 | 1.58 | -0.49 | -0.23 | -0.75 | 0.80 | 1.02 | -1.98 | -0.15 | 0.19 |
| Q8IW45 | ATP-dependent (S)-NAD(P)H-hydrate dehydratase | NAXD | -0.05 | 0.04 | -1.23 | -0.03 | 0.65 | -0.52 | 1.20 | -0.41 | -1.35 | 1.83 | 0.00 | -0.12 |
| Q8WW12 | PEST proteolytic signal-containing nuclear protein | PCNP | -0.05 | 0.17 | 0.91 | -1.90 | 0.88 | 0.68 | -0.32 | 0.65 | -1.60 | 0.13 | 0.45 | 0.12 |
| Q16181 | Septin-7 | SEPTIN7 | -0.05 | 0.27 | 1.04 | 0.83 | -1.42 | -0.75 | 0.68 | 0.10 | 1.43 | -0.90 | -1.14 | 0.14 |
| Q9BW83 | Intraflagellar transport protein 27 homolog | IFT27 | -0.05 | 0.09 | 0.72 | 0.37 | -0.82 | 0.93 | -1.08 | 0.42 | -0.69 | -1.78 | 1.02 | 0.89 |
| Q16795 | NADH dehydrogenase [ubiquinone] 1 alpha subcomplex subunit 9, | NDUFA9 | -0.05 | 0.05 | -0.71 | 0.86 | 1.57 | -0.83 | -0.82 | -0.82 | -0.56 | -0.71 | 0.55 | 1.46 |
| Q9BPZ7 | Target of rapamycin complex 2 subunit MAPKAP1 | MAPKAP1 | -0.05 | 0.06 | 2.17 | -0.58 | -0.70 | -0.11 | -0.67 | -0.46 | -0.49 | -0.85 | 0.43 | 1.28 |
| P55060 | Exportin-2 | CSE1L | -0.05 | 0.04 | 0.78 | -0.75 | 0.18 | -0.28 | 0.13 | 0.79 | -2.36 | 1.10 | -0.10 | 0.50 |
| P27707 | Deoxycytidine kinase | DCK | -0.05 | 0.06 | 0.75 | 1.57 | -0.89 | -0.53 | -0.80 | 1.22 | -0.66 | -0.96 | -0.67 | 0.98 |
| Q15750 | TGF-beta-activated kinase 1 and MAP3K7-binding protein 1 | TAB1 | -0.05 | 0.10 | 0.72 | 1.08 | -1.02 | 1.08 | -1.71 | -0.04 | -0.33 | -0.99 | 1.04 | 0.18 |
| Q7Z3G6 | Prickle-like protein 2 | PRICKLE2 | -0.05 | 0.06 | 1.48 | -0.12 | -0.98 | -0.12 | -0.16 | 1.05 | 1.35 | -0.20 | -1.46 | -0.84 |
| P56182 | Ribosomal RNA processing protein 1 homolog A | RRP1 | -0.05 | 0.13 | -0.05 | -0.43 | -0.96 | -0.49 | 2.11 | -0.30 | -0.39 | 1.55 | -0.45 | -0.60 |
| P06727 | Apolipoprotein A-IV | APOA4 | -0.05 | 0.05 | -0.61 | 0.20 | 1.43 | -0.02 | -0.92 | -0.83 | -0.91 | 1.50 | -0.90 | 1.06 |
| Q5JVS0 | Intracellular hyaluronan-binding protein 4 | HABP4 | -0.05 | 0.07 | 2.34 | -0.15 | -0.63 | -1.25 | -0.20 | -0.43 | 0.62 | -0.77 | -0.10 | 0.58 |
| P98160 | Basement membrane-specific heparan sulfate proteoglycan core | HSPG2 | -0.06 | 0.07 | 1.50 | -2.13 | 1.08 | -0.04 | -0.30 | 0.50 | 0.38 | -0.07 | -0.71 | -0.21 |
| Q92882 | Osteoclast-stimulating factor 1 | OSTF1 | -0.06 | 0.05 | 0.38 | 0.30 | -0.58 | 0.13 | -0.15 | 2.39 | -0.19 | -1.53 | -0.39 | -0.36 |
| Q12857 | Nuclear factor 1 A-type | NFIA | -0.06 | 0.08 | 0.44 | -0.11 | -0.46 | 1.18 | -0.93 | -0.92 | -0.92 | 1.12 | 1.57 | -0.96 |
| P62306 | Small nuclear ribonucleoprotein F | SNRPF | -0.06 | 0.11 | 1.20 | 0.11 | 0.66 | -1.57 | -0.23 | -0.44 | 0.78 | -0.81 | -1.08 | 1.40 |
| Q659C4 | La-related protein 1B | LARP1B | -0.06 | 0.12 | -0.90 | 1.05 | 0.00 | 1.27 | -1.24 | 0.04 | -0.97 | -1.01 | 1.34 | 0.43 |
| O75306 | NADH dehydrogenase [ubiquinone] iron-sulfur protein 2, mitochon | NDUFS2 | -0.06 | 0.05 | -1.22 | -0.05 | 0.29 | 0.82 | 0.23 | -1.28 | 0.65 | -1.55 | 0.93 | 1.17 |
| P05023 | Sodium/potassium-transporting ATPase subunit alpha-1 | ATP1A1 | -0.06 | 0.09 | 1.56 | 0.87 | -1.74 | -0.03 | -0.53 | 1.26 | -0.72 | -0.20 | -0.58 | 0.10 |
| Q9UMY4 | Sorting nexin-12 | SNX12 | -0.06 | 0.20 | -0.65 | 1.45 | -0.24 | -0.04 | -0.24 | -0.09 | 0.81 | -2.05 | -0.16 | 1.21 |
| Q01130 | Serine/arginine-rich splicing factor 2 | SRSF2 | -0.06 | 0.09 | 0.78 | 0.79 | -0.37 | -1.48 | 0.41 | -1.29 | -0.10 | 0.19 | -0.68 | 1.75 |
| P49917 | DNA ligase 4 | LIG4 | -0.06 | 0.38 | 1.61 | -0.17 | -1.02 | 0.49 | -0.40 | 0.60 | 0.36 | -2.06 | 0.26 | 0.34 |
| P03897 | NADH-ubiquinone oxidoreductase chain 3 | MT-ND3 | -0.06 | 0.50 | 1.32 | 0.08 | -0.27 | -0.07 | -0.40 | 0.94 | 0.01 | -2.33 | 0.83 | -0.09 |
| Q9Y5K5 | Ubiquitin carboxyl-terminal hydrolase isozyme L5 | UCHL5 | -0.06 | 0.09 | 0.87 | -0.56 | -1.88 | 0.88 | 0.83 | 0.05 | 0.48 | -1.37 | 0.88 | -0.17 |
| Q9BRL6 | Serine/arginine-rich splicing factor 8 | SRSF8 | -0.06 | 0.60 | 0.79 | -0.54 | -0.88 | 0.90 | 0.49 | -0.28 | 0.87 | -2.24 | 0.50 | 0.39 |
| Q8N0X7 | Spartin | SPART | -0.06 | 0.11 | 0.04 | 1.22 | -1.11 | 0.47 | -0.46 | 0.61 | 0.03 | -2.06 | 0.16 | 1.09 |
| P57772 | Selenocysteine-specific elongation factor | EEFSEC | -0.06 | 0.08 | 1.01 | -0.29 | -1.70 | 0.66 | 0.43 | 0.48 | 0.17 | -1.86 | 0.68 | 0.42 |
| Q9NRG7 | Epimerase family protein SDR39U1 | SDR39U1 | -0.06 | 0.08 | -2.08 | 0.32 | 0.44 | 0.54 | 0.91 | -1.27 | -0.51 | 1.10 | 0.27 | 0.28 |
| Q8TF40 | Folliculin-interacting protein 1 | FNIP1 | -0.06 | 0.68 | 1.48 | -0.28 | -0.53 | 0.45 | -0.27 | -0.18 | 0.58 | -2.16 | -0.15 | 1.06 |
| Q14257 | Reticulocalbin-2 | RCN2 | -0.06 | 0.09 | 1.43 | 0.77 | 0.56 | -1.85 | -0.76 | 0.57 | 0.69 | -0.93 | -0.60 | 0.14 |
| P62266 | 40S ribosomal protein S23 | RPS23 | -0.06 | 0.08 | -0.50 | -1.61 | -0.01 | -0.20 | 2.44 | -0.18 | 0.24 | 0.08 | -0.27 | 0.01 |
| O15145 | Actin-related protein 2/3 complex subunit 3 | ARPC3 | -0.06 | 0.11 | 0.06 | 1.62 | -0.91 | 0.89 | -1.51 | -0.20 | 0.72 | -1.30 | 0.46 | 0.16 |
| Q08AD1 | Calmodulin-regulated spectrin-associated protein 2 | CAMSAP2 | -0.06 | 0.25 | 0.27 | 0.97 | -2.08 | 1.31 | -0.12 | -0.84 | -0.74 | 0.20 | 0.74 | 0.29 |
| Q6VY07 | Phosphofurin acidic cluster sorting protein 1 | PACS1 | -0.06 | 0.14 | 0.22 | 0.26 | -0.40 | 1.01 | -0.88 | -1.30 | 0.31 | -1.45 | 0.59 | 1.64 |
| Q92538 | Golgi-specific brefeldin A-resistance guanine nucleotide exchange | GBF1 | -0.07 | 0.73 | 1.00 | -0.39 | -0.89 | 0.49 | 0.69 | 1.30 | 0.35 | -2.09 | -0.41 | -0.05 |
| P09471 | Guanine nucleotide-binding protein G(o) subunit alpha | GNAO1 | -0.07 | 0.17 | -0.73 | 2.08 | -0.02 | 0.35 | -1.43 | -0.07 | 0.20 | -1.22 | 0.64 | 0.20 |
| Q8WUD1 | Ras-related protein Rab-2B | RAB2B | -0.07 | 0.06 | 0.22 | 1.06 | -1.48 | 0.87 | -0.58 | -1.20 | 0.79 | 0.61 | 0.87 | -1.16 |
| Q6UXN9 | WD repeat-containing protein 82 | WDR82 | -0.07 | 0.15 | 1.29 | -0.72 | -1.01 | 0.76 | -0.10 | 0.58 | -0.61 | -1.71 | 0.31 | 1.21 |
| P52434 | DNA-directed RNA polymerases I, II, and III subunit RPABC3 | POLR2H | -0.07 | 0.06 | 1.22 | -1.36 | 1.28 | 0.23 | -1.28 | 0.45 | 0.09 | 0.32 | -1.37 | 0.42 |
| Q9H313 | Protein tweety homolog 1 | TTYH1 | -0.07 | 0.18 | -0.58 | 0.56 | 0.77 | 0.78 | -1.28 | -1.16 | -0.28 | 1.55 | 0.72 | -1.09 |
| Q8IY57 | YY1-associated factor 2 | YAF2 | -0.07 | 0.63 | 1.48 | -0.17 | -1.28 | 0.72 | 0.04 | -0.43 | 0.68 | -1.90 | 0.49 | 0.37 |
| Q16643 | Drebrin | DBN1 | -0.07 | 0.13 | 0.75 | 1.31 | -0.62 | 0.05 | -1.31 | 0.59 | 1.21 | -1.63 | -0.44 | 0.09 |
| O43299 | AP-5 complex subunit zeta-1 | AP5Z1 | -0.07 | 0.52 | 1.12 | 0.68 | -0.83 | -0.13 | -0.17 | 0.16 | 0.32 | -2.38 | 0.67 | 0.56 |
| Q3ZCQ8 | Mitochondrial import inner membrane translocase subunit TIM50 | TIMM50 | -0.07 | 0.07 | -0.50 | 1.15 | -0.81 | -0.73 | 0.99 | -0.67 | 1.47 | -1.08 | -0.76 | 0.94 |
| P10828 | Thyroid hormone receptor beta | THRB | -0.07 | 0.53 | 1.54 | -0.32 | -1.08 | 0.70 | -0.16 | 0.39 | 0.30 | -2.05 | 0.05 | 0.64 |
| P40818 | Ubiquitin carboxyl-terminal hydrolase 8 | USP8 | -0.07 | 0.18 | 0.95 | 0.74 | -0.10 | 0.53 | -1.86 | -0.26 | -1.57 | 0.05 | 0.98 | 0.55 |
| P09429 | High mobility group protein B1 | HMGB1 | -0.07 | 0.17 | 0.47 | -1.55 | 1.29 | 0.71 | -0.69 | -1.56 | 0.25 | 0.01 | 1.11 | -0.06 |
| Q9NYU2 | UDP-glucose:glycoprotein glucosyltransferase 1 | UGGT1 | -0.07 | 0.11 | 0.89 | 0.88 | 0.25 | -0.04 | -1.82 | 1.64 | -0.82 | -0.81 | 0.01 | -0.19 |
| P20794 | Serine/threonine-protein kinase MAK | MAK | -0.07 | 0.17 | -0.23 | 2.42 | -0.94 | -0.39 | -0.61 | -0.55 | 0.28 | 0.92 | -0.29 | -0.61 |
| Q96JC1 | Vam6/Vps39-like protein | VPS39 | -0.07 | 0.14 | 0.20 | -0.56 | -1.41 | 1.28 | 0.70 | 0.47 | -1.68 | -0.48 | 1.01 | 0.48 |
| Q15154 | Pericentriolar material 1 protein | PCM1 | -0.07 | 0.13 | -1.10 | 1.14 | 1.33 | -0.43 | -0.75 | -0.58 | 0.16 | -0.44 | -0.92 | 1.59 |
| Q6PJT7 | Zinc finger CCCH domain-containing protein 14 | ZC3H14 | -0.07 | 0.12 | -1.15 | 0.97 | -1.66 | 0.73 | 1.28 | -0.33 | -0.18 | -0.90 | 0.43 | 0.80 |
| Q9H0E9 | Bromodomain-containing protein 8 | BRD8 | -0.07 | 0.08 | 2.05 | -0.78 | -0.91 | -0.79 | 0.55 | 0.32 | 1.00 | -1.11 | 0.03 | -0.35 |
| Q7LDG7 | RAS guanyl-releasing protein 2 | RASGRP2 | -0.07 | 0.07 | 0.40 | 0.29 | -1.92 | 1.02 | 0.31 | 0.37 | -1.78 | 0.59 | 0.42 | 0.29 |
| Q58FF6 | Putative heat shock protein HSP 90-beta 4 | HSP90AB4P | -0.07 | 0.04 | -1.58 | 0.55 | 0.36 | 1.04 | -0.32 | -1.10 | -1.10 | -0.07 | 1.04 | 1.17 |
| Q12866 | Tyrosine-protein kinase Mer | MERTK | -0.07 | 0.07 | 0.34 | 0.11 | -1.24 | 0.73 | 0.16 | 0.00 | -1.14 | -1.50 | 1.21 | 1.32 |
| Q13885 | Tubulin beta-2A chain | TUBB2A | -0.07 | 0.22 | -0.05 | 1.90 | -1.06 | 0.70 | -1.17 | 0.76 | -0.24 | -1.26 | 0.42 | 0.01 |
| O00562 | Membrane-associated phosphatidylinositol transfer protein 1 | PITPNM1 | -0.07 | 0.12 | 0.89 | 0.65 | -1.48 | 0.41 | -0.29 | 1.29 | -0.23 | -1.85 | 0.37 | 0.26 |
| P49903 | Selenide, water dikinase 1 | SEPHS1 | -0.07 | 0.10 | 0.80 | 0.04 | -1.05 | -0.78 | 1.14 | -0.82 | -0.71 | 1.84 | -0.80 | 0.35 |
| P27695 | DNA-(apurinic or apyrimidinic site) lyase | APEX1 | -0.07 | 0.36 | 0.18 | -0.16 | 1.07 | -0.13 | -0.46 | -0.01 | -2.18 | -0.13 | 0.16 | 1.67 |
| Q6ZNA1 | Zinc finger protein 836 | ZNF836 | -0.08 | 0.17 | 1.00 | -1.27 | -1.54 | 1.09 | 0.97 | 0.55 | -0.96 | -0.24 | 0.72 | -0.31 |
| P09661 | U2 small nuclear ribonucleoprotein A' | SNRPA1 | -0.08 | 0.08 | 1.16 | 0.55 | 0.19 | -0.82 | -0.96 | -0.84 | -0.87 | 1.66 | -0.89 | 0.82 |
| Q9BXK1 | Krueppel-like factor 16 | KLF16 | -0.08 | 0.53 | 1.54 | -0.15 | -0.93 | -0.02 | 0.24 | 0.70 | 0.17 | -2.22 | 0.40 | 0.26 |
| P05771 | Protein kinase C beta type | PRKCB | -0.08 | 0.41 | 2.05 | -1.29 | -0.36 | 0.90 | -0.74 | -0.68 | 0.14 | -0.27 | 0.85 | -0.60 |
| O43719 | HIV Tat-specific factor 1 | HTATSF1 | -0.08 | 0.04 | 1.60 | -0.98 | -1.07 | 0.07 | 0.45 | 0.97 | 1.18 | -0.70 | -0.45 | -1.06 |
| Q6IPX3 | Transcription elongation factor A protein-like 6 | TCEAL6 | -0.08 | 0.09 | 0.62 | 1.76 | -0.96 | -0.61 | -0.67 | 0.53 | 1.27 | -1.16 | -0.72 | -0.06 |
| O94929 | Actin-binding LIM protein 3 | ABLIM3 | -0.08 | 0.14 | -0.20 | 1.32 | -1.31 | -0.22 | 0.62 | 1.58 | -0.41 | -1.46 | -0.31 | 0.41 |
| P61758 | Prefoldin subunit 3 | VBP1 | -0.08 | 0.22 | 0.17 | -0.06 | 1.42 | -0.08 | -1.14 | 1.52 | 0.87 | -1.15 | -0.94 | -0.62 |
| P84095 | Rho-related GTP-binding protein RhoG | RHOG | -0.08 | 0.40 | -0.42 | -0.63 | -0.89 | 1.56 | 0.92 | -1.13 | -0.48 | 0.26 | 1.52 | -0.68 |
| Q9UID3 | Vacuolar protein sorting-associated protein 51 homolog | VPS51 | -0.08 | 0.22 | -1.55 | 0.82 | 0.62 | 0.00 | 0.42 | -1.89 | -0.22 | 0.02 | 0.59 | 1.18 |
| P21397 | Amine oxidase [flavin-containing] A | MAOA | -0.08 | 0.58 | 1.67 | 0.11 | -0.89 | 0.24 | -0.39 | 0.68 | 0.42 | -2.12 | 0.19 | 0.09 |

| | | | | | | | | | | | | | | | |
|---|---|---|---|---|---|---|---|---|---|---|---|---|---|---|---|
| P29597 | Non-receptor tyrosine-protein kinase TYK2 | TYK2 | | -0.08 | 0.09 | -0.56 | 0.22 | -1.53 | 1.24 | 0.76 | -0.16 | -1.27 | -0.69 | 1.18 | 0.82 |
| Q5TEJ8 | Protein THEMIS2 | THEMIS2 | | -0.08 | 0.14 | -0.43 | 1.64 | -0.74 | 0.52 | -0.79 | -1.15 | 0.42 | -1.18 | 0.46 | 1.25 |
| Q6PIL6 | Kv channel-interacting protein 4 | KCNIP4 | | -0.08 | 0.04 | -1.05 | 0.23 | -1.24 | 1.10 | 1.02 | -1.11 | -1.06 | 0.39 | 1.05 | 0.67 |
| Q13740 | CD166 antigen | ALCAM | | -0.08 | 0.10 | 1.41 | 0.60 | -0.74 | -0.43 | -0.69 | -0.45 | -0.54 | 2.05 | -0.61 | -0.61 |
| Q5VT66 | Mitochondrial amidoxime-reducing component 1 | | 1-Mar | -0.08 | 0.07 | 1.68 | 0.29 | 0.01 | 0.07 | -1.96 | 0.23 | 1.13 | -0.75 | -0.10 | -0.60 |
| Q96PK6 | RNA-binding protein 14 | RBM14 | | -0.08 | 0.12 | 1.03 | -1.06 | -1.61 | 0.67 | 1.14 | 0.65 | -0.89 | -0.89 | 0.48 | 0.49 |
| Q86VS8 | Protein Hook homolog 3 | HOOK3 | | -0.08 | 0.09 | 0.69 | -0.18 | -1.56 | 0.87 | 0.31 | 0.09 | 0.38 | -1.92 | 0.16 | 1.15 |
| Q92747 | Actin-related protein 2/3 complex subunit 1A | ARPC1A | | -0.08 | 0.53 | 0.01 | -0.74 | 0.61 | 0.73 | 0.08 | -1.92 | 0.17 | 1.52 | 0.61 | -1.07 |
| Q96RU3 | Formin-binding protein 1 | FNBP1 | | -0.08 | 0.13 | 1.12 | 0.03 | 0.96 | -0.91 | -1.02 | 1.33 | 0.84 | -1.41 | -0.53 | -0.41 |
| Q6ZN44 | Netrin receptor UNC5A | UNC5A | | -0.08 | 0.08 | -0.01 | -0.21 | -0.82 | -0.55 | 1.71 | -0.25 | 1.93 | -0.89 | -0.32 | -0.59 |
| P08621 | U1 small nuclear ribonucleoprotein 70 kDa | SNRNP70 | | -0.08 | 0.30 | 1.08 | -1.21 | 0.37 | -1.19 | 1.36 | -0.66 | 0.41 | -0.13 | -1.10 | 1.06 |
| P52907 | F-actin-capping protein subunit alpha-1 | CAPZA1 | | -0.08 | 0.12 | 1.55 | 0.69 | 0.03 | -0.48 | -1.61 | 0.20 | 1.34 | -0.26 | -0.32 | -1.14 |
| P62191 | 26S proteasome regulatory subunit 4 | PSMC1 | | -0.08 | 0.33 | 1.28 | 1.81 | -1.31 | -0.45 | -0.87 | -0.16 | -0.80 | -0.26 | 0.81 | -0.05 |
| P62081 | 40S ribosomal protein S7 | RPS7 | | -0.08 | 0.16 | -1.33 | -1.13 | 0.12 | 1.11 | 1.47 | 0.62 | -0.97 | -0.84 | 0.65 | 0.31 |
| P27144 | Adenylate kinase 4, mitochondrial | AK4 | | -0.08 | 0.10 | 0.19 | 0.77 | 0.83 | -1.86 | 0.20 | -0.83 | 0.75 | -1.41 | 0.55 | 0.80 |
| P23246 | Splicing factor, proline- and glutamine-rich | SFPQ | | -0.09 | 0.30 | 2.10 | -0.31 | -0.76 | -0.20 | -0.42 | 0.42 | -1.25 | -0.88 | 0.23 | 1.07 |
| Q8IZU2 | WD repeat-containing protein 17 | WDR17 | | -0.09 | 0.14 | -0.52 | 2.17 | -0.52 | -0.47 | -0.46 | 0.90 | 0.80 | -0.62 | -1.22 | -0.06 |
| A7MD48 | Serine/arginine repetitive matrix protein 4 | SRRM4 | | -0.09 | 0.05 | 1.02 | -0.34 | -1.56 | 0.03 | 0.92 | 1.17 | 0.97 | -0.45 | -0.39 | -1.38 |
| P28289 | Tropomodulin-1 | TMOD1 | | -0.09 | 0.11 | -0.85 | 0.87 | 0.40 | -1.03 | 0.78 | -0.88 | -0.85 | 1.18 | -0.97 | 1.36 |
| Q9BUF5 | Tubulin beta-6 chain | TUBB6 | | -0.09 | 0.16 | 0.15 | 0.48 | -1.51 | 0.95 | 0.16 | 1.57 | -0.02 | -1.58 | 0.44 | -0.63 |
| Q15019 | Septin-2 | SEPTIN2 | | -0.09 | 0.22 | 1.09 | 0.47 | -0.14 | -1.87 | 0.76 | 0.66 | 0.95 | -0.31 | -1.44 | -0.17 |
| P54753 | Ephrin type-B receptor 3 | EPHB3 | | -0.09 | 0.14 | 0.51 | 0.79 | -0.86 | 0.31 | -0.54 | 1.08 | 0.09 | -2.29 | 0.75 | 0.17 |
| P52943 | Cysteine-rich protein 2 | CRIP2 | | -0.09 | 0.23 | 0.36 | 0.59 | -1.09 | 0.40 | 0.06 | -1.00 | 2.02 | -1.33 | 0.39 | -0.40 |
| Q9GZL7 | Ribosome biogenesis protein WDR12 | WDR12 | | -0.09 | 0.90 | 1.29 | -0.24 | -1.15 | 0.67 | 0.49 | 0.25 | 0.23 | -2.12 | -0.20 | 0.78 |
| Q9BY42 | Replication termination factor 2 | RTF2 | | -0.09 | 0.09 | 1.48 | -1.07 | -1.63 | 0.32 | 1.03 | 0.37 | 0.08 | -1.19 | 0.08 | 0.53 |
| Q9UIF9 | Bromodomain adjacent to zinc finger domain protein 2A | BAZ2A | | -0.09 | 0.14 | 2.05 | -0.40 | -0.83 | 0.02 | -0.63 | 1.48 | -0.39 | -1.06 | 0.01 | -0.25 |
| P49959 | Double-strand break repair protein MRE11 | MRE11 | | -0.09 | 0.18 | 0.94 | 0.46 | -2.20 | 0.87 | 0.19 | 0.18 | -1.27 | 0.16 | -0.09 | 0.76 |
| Q9H4F8 | SPARC-related modular calcium-binding protein 1 | SMOC1 | | -0.09 | 0.22 | -0.35 | 1.64 | -0.95 | 0.96 | -0.98 | -0.50 | -0.62 | -0.99 | 0.58 | 1.21 |
| P23610 | Factor VIII intron 22 protein | F8A1 | | -0.09 | 0.11 | 0.70 | 1.00 | -0.65 | 0.38 | -1.28 | -0.50 | 1.75 | -0.64 | 0.41 | -1.18 |
| Q9ULR3 | Protein phosphatase 1H | PPM1H | | -0.09 | 0.34 | 1.06 | -0.52 | 0.19 | -0.10 | -0.16 | 1.98 | 0.10 | 0.02 | -0.89 | -1.68 |
| Q9UNT1 | Rab-like protein 2B | RABL2B | | -0.09 | 0.10 | 0.70 | -0.47 | -1.01 | 0.85 | 0.06 | -0.41 | 1.48 | -1.90 | 0.76 | -0.07 |
| Q14247 | Src substrate cortactin | CTTN | | -0.09 | 0.26 | 0.05 | 0.88 | 0.28 | 1.26 | -2.12 | -0.74 | -0.95 | 0.31 | 0.56 | 0.46 |
| P49458 | Signal recognition particle 9 kDa protein | SRP9 | | -0.09 | 0.28 | -0.13 | 0.33 | -1.14 | 1.30 | 0.02 | -1.23 | -1.50 | 0.35 | 1.08 | 0.91 |
| O15018 | PDZ domain-containing protein 2 | PDZD2 | | -0.09 | 0.22 | -0.40 | -0.82 | -0.01 | -0.61 | 2.16 | 0.72 | -1.32 | 0.55 | -0.64 | 0.38 |
| P00750 | Tissue-type plasminogen activator | PLAT | | -0.09 | 1.12 | 1.05 | 0.35 | -0.92 | 0.67 | 0.12 | 0.54 | 0.88 | -2.29 | -0.02 | -0.38 |
| P13647 | Keratin, type II cytoskeletal 5 | KRT5 | | -0.09 | 0.07 | -1.64 | 0.42 | 0.55 | -0.07 | 0.83 | -0.47 | -1.78 | 0.64 | 1.07 | 0.45 |
| Q9BRX2 | Protein pelota homolog | PELO | | -0.09 | 0.10 | -0.27 | -0.54 | 0.43 | 0.37 | 0.17 | -0.95 | -2.04 | 1.55 | 0.65 | 0.65 |
| Q9NQ79 | Cartilage acidic protein 1 | CRTAC1 | | -0.09 | 0.06 | -1.46 | -0.72 | 1.29 | 0.46 | 0.51 | -1.53 | -0.04 | 1.30 | 0.36 | -0.17 |
| P01023 | Alpha-2-macroglobulin | A2M | | -0.09 | 0.12 | -1.47 | 0.13 | 0.09 | 1.01 | 0.41 | -1.02 | -1.54 | 0.35 | 1.04 | 0.99 |
| Q5VWN6 | Protein TASOR 2 | TASOR2 | | -0.09 | 0.86 | 1.50 | -0.06 | -0.88 | 0.87 | -0.42 | 0.52 | -0.26 | -2.06 | 0.12 | 0.67 |
| O60266 | Adenylate cyclase type 3 | ADCY3 | | -0.09 | 0.07 | 1.98 | -0.38 | -0.56 | -0.45 | -0.51 | 1.79 | -0.38 | -0.71 | -0.39 | -0.40 |
| Q9UQ88 | Cyclin-dependent kinase 11A | CDK11A | | -0.09 | 0.14 | -1.09 | 0.13 | 0.33 | 0.02 | 0.82 | 1.13 | -2.31 | 0.13 | 0.56 | 0.28 |
| Q96S55 | ATPase WRNIP1 | WRNIP1 | | -0.09 | 0.10 | 1.21 | -1.93 | -0.34 | 0.11 | 1.10 | 0.60 | 1.01 | -0.77 | -0.36 | -0.62 |
| P49588 | Alanine--tRNA ligase, cytoplasmic | AARS | | -0.09 | 0.20 | 1.03 | -0.45 | 0.96 | 0.24 | -1.51 | 0.70 | 0.81 | -1.80 | -0.20 | 0.22 |
| P07196 | Neurofilament light polypeptide | NEFL | | -0.09 | 0.14 | 0.75 | -0.57 | 1.48 | -0.43 | -1.03 | 0.56 | 1.20 | -1.70 | -0.09 | -0.16 |
| Q68CZ2 | Tensin-3 | TNS3 | | -0.09 | 0.17 | 0.53 | 0.30 | -1.00 | 0.42 | 0.00 | 0.82 | -0.21 | -2.25 | 0.09 | 1.30 |
| Q96EE3 | Nucleoporin SEH1 | SEH1L | | -0.09 | 0.18 | 0.59 | -1.82 | -0.47 | 1.10 | 0.85 | -0.27 | -0.62 | -0.98 | 1.29 | 0.33 |
| P17152 | Transmembrane protein 11, mitochondrial | TMEM11 | | -0.09 | 0.11 | -0.88 | 0.75 | 0.41 | -0.18 | 0.06 | -0.26 | 1.41 | -1.94 | -0.52 | 1.16 |
| Q86U42 | Polyadenylate-binding protein 2 | PABPN1 | | -0.09 | 0.14 | 0.83 | -0.84 | -0.80 | 0.67 | 0.35 | 0.12 | -2.06 | -0.28 | 0.91 | 1.11 |
| P61106 | Ras-related protein Rab-14 | RAB14 | | -0.10 | 0.13 | 0.95 | 0.30 | -0.11 | -0.28 | -0.67 | -1.71 | -0.77 | 1.85 | -0.27 | 0.70 |
| Q99873 | Protein arginine N-methyltransferase 1 | PRMT1 | | -0.10 | 0.41 | 0.05 | -0.44 | 0.12 | 1.41 | -0.60 | 0.05 | -0.44 | -2.09 | 0.97 | 0.96 |
| P47813 | Eukaryotic translation initiation factor 1A, X-chromosomal | EIF1AX | | -0.10 | 0.18 | 1.02 | 0.09 | 0.69 | -1.41 | -0.14 | 0.79 | 0.78 | -2.05 | 0.09 | 0.13 |
| Q9H1P3 | Oxysterol-binding protein-related protein 2 | OSBPL2 | | -0.10 | 0.15 | 0.44 | 1.00 | 0.02 | 0.14 | -1.39 | 1.17 | -0.41 | -1.91 | 0.16 | 0.79 |
| Q14764 | Major vault protein | MVP | | -0.10 | 0.36 | -2.29 | 0.66 | 0.60 | 0.21 | 1.32 | -0.73 | -0.08 | 0.56 | 0.25 | -0.49 |
| Q15836 | Vesicle-associated membrane protein 3 | VAMP3 | | -0.10 | 0.10 | -1.09 | 0.85 | 0.65 | 0.93 | -1.18 | -1.21 | -1.14 | 0.67 | 0.85 | 0.67 |
| P49755 | Transmembrane emp24 domain-containing protein 10 | TMED10 | | -0.10 | 0.27 | -0.25 | 0.76 | -0.38 | 0.58 | -0.33 | -0.80 | -2.19 | 0.70 | 0.98 | 0.94 |
| Q96B97 | SH3 domain-containing kinase-binding protein 1 | SH3KBP1 | | -0.10 | 0.14 | 1.45 | -0.23 | 0.52 | -0.14 | -1.39 | 0.96 | 1.03 | -1.56 | -0.17 | -0.46 |
| P51452 | Dual specificity protein phosphatase 3 | DUSP3 | | -0.10 | 0.20 | -1.53 | 1.11 | 0.32 | 0.27 | 0.10 | -0.50 | 1.18 | -1.80 | 0.41 | 0.43 |
| Q15796 | Mothers against decapentaplegic homolog 2 | SMAD2 | | -0.10 | 0.20 | -0.04 | -0.45 | -0.94 | 1.32 | 0.38 | -0.69 | -1.47 | -0.58 | 1.13 | 1.33 |
| Q147X3 | N-alpha-acetyltransferase 30 | NAA30 | | -0.10 | 0.10 | -0.34 | 1.27 | 0.07 | 0.34 | -1.20 | 1.66 | 0.05 | -1.44 | -0.84 | 0.42 |
| Q8TEA8 | D-aminoacyl-tRNA deacylase 1 | DTD1 | | -0.10 | 0.33 | -0.30 | 0.74 | 1.61 | 0.17 | -1.77 | -1.44 | 0.44 | -0.24 | 0.41 | 0.38 |
| Q9NVW2 | E3 ubiquitin-protein ligase RLIM | RLIM | | -0.10 | 0.89 | 1.31 | 0.52 | -0.66 | 0.01 | -0.13 | 0.04 | 0.88 | -2.34 | -0.17 | 0.53 |
| P04156 | Major prion protein | PRNP | | -0.10 | 0.26 | -0.01 | 1.74 | -0.37 | -0.64 | -0.37 | -1.87 | 0.36 | 1.23 | -0.29 | 0.21 |
| O94875 | Sorbin and SH3 domain-containing protein 2 | SORBS2 | | -0.10 | 0.13 | 0.36 | 0.86 | -0.10 | -0.02 | -0.90 | -2.06 | 0.28 | 1.58 | -0.48 | 0.50 |
| O43237 | Cytoplasmic dynein 1 light intermediate chain 2 | DYNC1LI2 | | -0.10 | 0.24 | -1.84 | 0.98 | 0.94 | 0.86 | -0.61 | -0.91 | -0.91 | 0.74 | 0.65 | 0.10 |
| Q6NVY1 | 3-hydroxyisobutyryl-CoA hydrolase, mitochondrial | HIBCH | | -0.10 | 0.12 | -1.64 | -0.39 | 1.16 | 0.64 | 0.41 | -0.71 | -1.52 | 0.71 | 0.85 | 0.49 |
| Q8IU85 | Calcium/calmodulin-dependent protein kinase type 1D | CAMK1D | | -0.10 | 0.23 | 1.02 | 0.41 | -2.28 | 0.95 | 0.22 | -0.54 | -0.53 | -0.16 | 1.02 | -0.11 |
| Q9NX14 | NADH dehydrogenase [ubiquinone] 1 beta subcomplex subunit 11, | NDUFB11 | | -0.10 | 0.87 | 1.08 | 0.46 | -1.01 | 0.12 | 0.39 | 0.03 | 0.60 | -2.34 | -0.11 | 0.78 |
| O60241 | Adhesion G protein-coupled receptor B2 | ADGRB2 | | -0.10 | 0.70 | 1.41 | 0.57 | -0.56 | 0.02 | -0.58 | -0.06 | 0.40 | -2.29 | 0.47 | 0.62 |
| Q96AG4 | Leucine-rich repeat-containing protein 59 | LRRC59 | | -0.10 | 0.30 | -0.08 | -0.24 | 1.47 | -0.40 | -0.33 | -0.61 | 2.12 | -1.15 | -0.44 | -0.33 |
| P0DPB6 | DNA-directed RNA polymerases I and III subunit RPAC2 | POLR1D | | -0.10 | 0.94 | 1.11 | 0.46 | -0.88 | 0.22 | 0.20 | 0.23 | 0.56 | -2.45 | 0.59 | -0.03 |
| P61158 | Actin-related protein 3 | ACTR3 | | -0.10 | 0.21 | 0.38 | 1.78 | -1.58 | 0.36 | -0.64 | -0.43 | 1.28 | -0.91 | -0.09 | -0.15 |
| Q9UJD0 | Regulating synaptic membrane exocytosis protein 3 | RIMS3 | | -0.10 | 0.18 | 1.07 | 0.58 | -1.41 | -0.04 | 0.05 | 1.04 | 0.85 | -1.90 | 0.08 | -0.32 |
| Q8TAF3 | WD repeat-containing protein 48 | WDR48 | | -0.10 | 0.26 | -0.36 | -0.94 | -0.06 | 0.18 | 1.53 | -1.65 | 1.27 | 0.40 | 0.54 | -0.92 |
| P41250 | Glycine--tRNA ligase | GARS | | -0.10 | 0.28 | -0.48 | -0.05 | 2.02 | 0.51 | -1.60 | -0.14 | -0.88 | 0.53 | 0.68 | -0.58 |
| P12277 | Creatine kinase B-type | CKB | | -0.10 | 0.21 | 0.11 | 1.15 | -1.05 | 0.20 | -0.11 | 0.98 | -0.27 | -2.16 | 0.31 | 0.84 |
| O00445 | Synaptotagmin-5 | SYT5 | | -0.10 | 0.12 | 0.58 | 1.18 | -0.21 | -0.08 | -1.30 | 0.05 | 1.60 | -1.60 | -0.55 | 0.33 |
| P07355 | Annexin A2 | ANXA2 | | -0.10 | 0.16 | -0.68 | 1.23 | 1.22 | -0.29 | -1.24 | -0.33 | -1.21 | 1.35 | -0.52 | 0.47 |
| E9PQ53 | NADH dehydrogenase [ubiquinone] 1 subunit C2, isoform 2 | NDUFC2-KCTD14 | | -0.10 | 0.91 | 0.86 | 0.63 | -1.23 | 0.36 | 0.46 | 0.73 | -0.08 | -2.24 | -0.13 | 0.65 |
| Q9GZV7 | Hyaluronan and proteoglycan link protein 2 | HAPLN2 | | -0.10 | 0.10 | -0.44 | -1.03 | 2.09 | -0.18 | -0.30 | -0.07 | -1.27 | 1.28 | 0.05 | -0.13 |
| P10645 | Chromogranin-A | CHGA | | -0.10 | 0.19 | 0.94 | -0.46 | -0.23 | -1.12 | 1.13 | 0.63 | 1.09 | 0.57 | -1.56 | -0.99 |
| O43399 | Tumor protein D54 | TPD52L2 | | -0.10 | 0.12 | 0.81 | 0.28 | -2.53 | 0.67 | 0.94 | -0.40 | 0.04 | -0.35 | 0.46 | 0.08 |
| Q96B23 | Uncharacterized protein C18orf25 | C18orf25 | | -0.10 | 0.14 | 0.75 | 1.13 | 0.65 | -0.99 | -1.34 | 0.37 | 0.58 | -1.71 | -0.17 | 0.73 |

| | | | | | | | | | | | | | | |
|---|---|---|---|---|---|---|---|---|---|---|---|---|---|---|
| Q6PJG2 | ELM2 and SANT domain-containing protein 1 | ELMSAN1 | -0.10 | 0.20 | 2.01 | -0.31 | -0.64 | -0.22 | -0.55 | -0.45 | 1.68 | -0.97 | -0.30 | -0.24 |
| P56559 | ADP-ribosylation factor-like protein 4C | ARL4C | -0.10 | 0.11 | 2.23 | -0.47 | -0.59 | -0.48 | -0.54 | -0.43 | -0.45 | 1.50 | -0.45 | -0.33 |
| O00592 | Podocalyxin | PODXL | -0.10 | 0.18 | -0.29 | 2.29 | -0.70 | -0.45 | -0.60 | -0.42 | -0.47 | 1.39 | -0.51 | -0.24 |
| P16298 | Serine/threonine-protein phosphatase 2B catalytic subunit beta is | PPP3CB | -0.10 | 0.27 | -0.63 | 1.38 | 1.01 | 0.22 | -1.61 | -0.24 | -0.14 | 1.39 | -0.51 | -0.88 |
| P50548 | ETS domain-containing transcription factor ERF | ERF | -0.10 | 0.13 | 1.92 | -0.62 | -0.78 | 0.37 | -0.70 | -0.55 | -0.57 | -0.94 | 0.49 | 1.39 |
| Q8N3V7 | Synaptopodin | SYNPO | -0.11 | 0.12 | 0.28 | 0.66 | -0.32 | -0.27 | -0.17 | -1.55 | -0.12 | 2.35 | -0.48 | -0.38 |
| Q5T0F9 | Coiled-coil and C2 domain-containing protein 1B | CC2D1B | -0.11 | 0.17 | 0.80 | -0.22 | -1.66 | 0.56 | 0.75 | 0.43 | 0.26 | -1.97 | 0.39 | 0.65 |
| Q68EM7 | Rho GTPase-activating protein 17 | ARHGAP17 | -0.11 | 0.07 | -1.13 | 0.81 | -1.39 | 1.00 | 0.81 | -0.52 | -1.25 | -0.14 | 0.87 | 0.93 |
| Q02878 | 60S ribosomal protein L6 | RPL6 | -0.11 | 0.20 | -0.50 | -1.51 | 0.22 | -0.35 | 2.43 | -0.43 | 0.10 | 0.01 | -0.29 | 0.32 |
| Q96GW7 | Brevican core protein | BCAN | -0.11 | 0.17 | 0.27 | -1.03 | 0.37 | -0.35 | 0.98 | -0.59 | -0.88 | 2.16 | -0.02 | -0.92 |
| A6NNA2 | Serine/arginine repetitive matrix protein 3 | SRRM3 | -0.11 | 0.15 | -1.34 | -0.21 | -0.56 | 0.14 | 2.19 | -0.68 | -0.67 | 0.89 | -0.27 | 0.51 |
| Q16775 | Hydroxyacylglutathione hydrolase, mitochondrial | HAGH | -0.11 | 0.15 | -1.18 | 0.35 | 0.40 | 0.85 | -0.21 | -1.64 | -1.19 | 1.22 | 0.67 | 0.73 |
| P24588 | A-kinase anchor protein 5 | AKAP5 | -0.11 | 0.31 | -0.49 | 1.87 | -0.77 | -0.32 | 0.14 | 0.32 | -0.13 | -1.56 | -0.41 | 1.35 |
| Q96CT7 | Coiled-coil domain-containing protein 124 | CCDC124 | -0.11 | 0.16 | 0.18 | 1.70 | 1.02 | -1.02 | -1.65 | 0.40 | 0.05 | -0.82 | -0.41 | 0.53 |
| P62750 | 60S ribosomal protein L23a | RPL23A | -0.11 | 0.27 | 0.01 | -0.77 | 0.98 | -1.53 | 1.70 | 0.04 | 0.59 | 0.17 | -1.35 | 0.16 |
| Q9UNZ2 | NSFL1 cofactor p47 | NSFL1C | -0.11 | 0.21 | 1.41 | 0.88 | -0.33 | -1.51 | -0.15 | 0.56 | 0.99 | -1.55 | -0.37 | 0.07 |
| Q8WWM7 | Ataxin-2-like protein | ATXN2L | -0.11 | 0.07 | 1.20 | -0.56 | 0.58 | -0.60 | -0.51 | 1.34 | 1.27 | -1.43 | -0.75 | -0.54 |
| P51649 | Succinate-semialdehyde dehydrogenase, mitochondrial | ALDH5A1 | -0.11 | 0.15 | 0.89 | -0.53 | 0.49 | -1.04 | 0.41 | 0.48 | -0.69 | 1.90 | -1.33 | -0.58 |
| O94829 | Importin-13 | IPO13 | -0.11 | 1.04 | 1.67 | 0.40 | -0.85 | 0.31 | -0.34 | 0.09 | -0.19 | -2.11 | 0.74 | 0.28 |
| Q9UKB1 | F-box/WD repeat-containing protein 11 | FBXW11 | -0.11 | 0.14 | 0.48 | -0.19 | -1.61 | 0.85 | 0.68 | 0.12 | 0.89 | -1.85 | -0.18 | 0.83 |
| O43505 | Beta-1,4-glucuronyltransferase 1 | B4GAT1 | -0.11 | 0.09 | 0.41 | 0.63 | -1.06 | -0.89 | 1.03 | -0.83 | 1.18 | 1.32 | -0.94 | -0.87 |
| P06239 | Tyrosine-protein kinase Lck | LCK | -0.11 | 0.80 | 1.01 | 0.49 | -1.25 | 0.53 | 0.19 | 0.39 | 0.47 | -2.22 | -0.30 | 0.69 |
| Q5S007 | Leucine-rich repeat serine/threonine-protein kinase 2 | LRRK2 | -0.11 | 0.29 | -0.08 | -0.39 | -0.77 | 1.87 | -0.22 | -0.40 | -0.18 | -1.20 | 1.72 | -0.34 |
| Q96S97 | Myeloid-associated differentiation marker | MYADM | -0.11 | 0.35 | -1.67 | 1.10 | 0.13 | 0.94 | -0.03 | -1.88 | 0.11 | 0.53 | 0.36 | 0.42 |
| Q99570 | Phosphoinositide 3-kinase regulatory subunit 4 | PIK3R4 | -0.11 | 0.09 | 0.82 | -0.11 | -0.71 | 0.14 | -0.01 | 0.96 | 1.07 | -2.39 | 0.14 | 0.07 |
| O94806 | Serine/threonine-protein kinase D3 | PRKD3 | -0.11 | 0.36 | 0.17 | -0.23 | 1.27 | -0.12 | -0.60 | 2.08 | -0.29 | -1.49 | -0.35 | -0.44 |
| O43396 | Thioredoxin-like protein 1 | TXNL1 | -0.11 | 0.36 | -1.18 | 1.65 | 0.25 | 0.18 | -0.41 | -1.44 | -0.67 | -0.31 | 1.19 | 0.75 |
| Q13630 | GDP-L-fucose synthase | TSTA3 | -0.11 | 0.22 | 1.50 | 0.08 | 0.15 | -0.48 | -0.94 | -0.04 | 1.95 | -0.93 | -0.45 | -0.84 |
| Q01968 | Inositol polyphosphate 5-phosphatase OCRL | OCRL | -0.11 | 0.23 | 0.65 | 1.61 | -1.89 | 0.62 | -0.66 | 0.00 | 0.14 | -1.15 | 0.45 | 0.23 |
| Q86VV4 | Ran-binding protein 3-like | RANBP3L | -0.11 | 1.18 | 0.82 | 0.39 | -0.80 | 0.47 | 0.45 | 0.75 | -0.34 | -2.43 | -0.02 | 0.71 |
| Q9UNK9 | Protein angel homolog 1 | ANGEL1 | -0.11 | 0.18 | 1.96 | -0.34 | -0.71 | -0.25 | -0.40 | 1.74 | -0.33 | -0.97 | -0.42 | -0.27 |
| Q14344 | Guanine nucleotide-binding protein subunit alpha-13 | GNA13 | -0.11 | 0.11 | -1.64 | -0.76 | 1.47 | 0.29 | 0.80 | 0.07 | -1.41 | 0.76 | 0.50 | -0.08 |
| P07919 | Cytochrome b-c1 complex subunit 6, mitochondrial | UQCRH | -0.12 | 1.02 | 1.52 | 0.07 | -0.63 | -0.09 | 0.31 | 0.21 | 0.64 | -2.35 | 0.45 | -0.14 |
| Q04917 | 14-3-3 protein eta | YWHAH | -0.12 | 0.23 | 0.69 | 0.51 | -0.07 | -0.23 | -0.57 | 0.06 | 1.18 | -2.37 | -0.10 | 0.91 |
| Q9H3M7 | Thioredoxin-interacting protein | TXNIP | -0.12 | 0.23 | 0.42 | 0.13 | -0.87 | -0.03 | 0.68 | 1.61 | 0.99 | -1.50 | -0.10 | -1.33 |
| P14927 | Cytochrome b-c1 complex subunit 7 | UQCRB | -0.12 | 1.24 | 1.47 | 0.37 | -1.20 | 0.60 | 0.12 | -0.19 | 0.54 | -1.96 | -0.47 | 0.72 |
| Q8IWZ3 | Ankyrin repeat and KH domain-containing protein 1 | ANKHD1 | -0.12 | 0.16 | 1.49 | -1.34 | -1.17 | 0.42 | 0.84 | 0.76 | 0.73 | -0.75 | -1.04 | 0.06 |
| Q8NCM8 | Cytoplasmic dynein 2 heavy chain 1 | DYNC2H1 | -0.12 | 0.16 | 0.46 | -1.08 | -1.33 | 1.11 | 1.08 | 0.73 | -0.08 | -1.57 | 0.02 | 0.67 |
| P41091 | Eukaryotic translation initiation factor 2 subunit 3 | EIF2S3 | -0.12 | 0.42 | -0.11 | -1.00 | -0.18 | 2.02 | -0.16 | -0.77 | 0.55 | -1.36 | 1.06 | -0.03 |
| Q8TBK6 | Zinc finger CCHC domain-containing protein 10 | ZCCHC10 | -0.12 | 1.40 | 2.27 | -0.37 | -0.43 | 0.33 | -0.31 | -0.01 | -0.21 | -1.77 | 0.10 | 0.39 |
| Q9H4Z3 | mRNA (2'-O-methyladenosine-N(6)-)-methyltransferase | PCIF1 | -0.12 | 0.20 | 0.07 | -0.91 | 0.50 | 0.43 | 0.18 | -0.84 | -2.05 | 0.49 | 0.67 | 1.45 |
| O00170 | AH receptor-interacting protein | AIP | -0.12 | 0.16 | 0.60 | 0.22 | -1.34 | 1.09 | -0.33 | 0.10 | -0.13 | -1.80 | 0.11 | 1.49 |
| O95197 | Reticulon-3 | RTN3 | -0.12 | 0.20 | 0.22 | -0.85 | -1.09 | 0.99 | 1.02 | 1.57 | -1.37 | -0.25 | 0.43 | -0.66 |
| Q9BT88 | Synaptotagmin-11 | SYT11 | -0.12 | 0.19 | 0.23 | 1.64 | 0.46 | -0.07 | -1.98 | -0.29 | 0.79 | -0.98 | 0.56 | -0.36 |
| Q9UKK9 | ADP-sugar pyrophosphatase | NUDT5 | -0.12 | 0.09 | -0.48 | -0.13 | 0.66 | -0.54 | 0.63 | -0.64 | -0.66 | 2.45 | -0.66 | -0.63 |
| Q02252 | Methylmalonate-semialdehyde dehydrogenase [acylating], mitoch | ALDH6A1 | -0.12 | 0.23 | 1.56 | -1.68 | 0.89 | -0.57 | 0.12 | 0.23 | 0.97 | -1.27 | -0.17 | -0.09 |
| Q8N8K9 | Uncharacterized protein KIAA1958 | KIAA1958 | -0.12 | 1.16 | 1.14 | 0.87 | -0.34 | 0.10 | -0.47 | 0.18 | 0.15 | -2.47 | 0.60 | 0.24 |
| P07327 | Alcohol dehydrogenase 1A | ADH1A | -0.12 | 0.12 | -0.37 | -0.44 | 1.92 | -0.37 | -0.57 | -0.53 | -0.45 | 1.86 | -0.56 | -0.51 |
| P62318 | Small nuclear ribonucleoprotein Sm D3 | SNRPD3 | -0.12 | 0.26 | 0.65 | -1.68 | 1.03 | -0.61 | 0.97 | -1.07 | -0.81 | 0.47 | -0.12 | 1.17 |
| O00410 | Importin-5 | IPO5 | -0.12 | 0.24 | -1.35 | 0.31 | -0.25 | 0.62 | 1.01 | -1.78 | -0.12 | 1.54 | 0.19 | -0.17 |
| Q9GZU2 | Paternally-expressed gene 3 protein | PEG3 | -0.12 | 0.94 | 1.66 | 0.60 | -1.02 | 0.15 | -0.29 | 0.25 | 0.05 | -2.07 | 0.06 | 0.61 |
| P62269 | 40S ribosomal protein S18 | RPS18 | -0.12 | 0.26 | -0.67 | -1.33 | 1.60 | 0.15 | 0.61 | -1.33 | -0.49 | 1.28 | 0.39 | -0.21 |
| A6NFI3 | Zinc finger protein 316 | ZNF316 | -0.12 | 0.14 | 0.76 | -0.95 | -1.13 | 0.80 | 0.73 | 1.17 | 0.95 | -1.40 | -0.01 | -0.93 |
| P15170 | Eukaryotic peptide chain release factor GTP-binding subunit ERF3 | GSPT1 | -0.12 | 0.20 | -1.18 | -0.16 | 0.60 | -0.23 | 1.26 | -1.30 | -0.49 | 1.89 | -0.23 | -0.15 |
| Q8TF21 | Ankyrin repeat domain-containing protein 24 | ANKRD24 | -0.12 | 0.08 | 1.62 | 0.01 | -1.53 | 0.16 | -0.15 | 1.62 | -0.89 | -0.74 | 0.00 | -0.10 |
| P22061 | Protein-L-isoaspartate(D-aspartate) O-methyltransferase | PCMT1 | -0.12 | 0.41 | -0.59 | 1.94 | 0.44 | -0.13 | -1.13 | -1.06 | 1.29 | -0.18 | -0.75 | 0.15 |
| O14975 | Very long-chain acyl-CoA synthetase | SLC27A2 | -0.12 | 0.22 | -0.10 | 0.71 | -0.80 | -0.38 | 0.88 | -0.48 | -0.57 | -1.12 | -0.36 | 2.23 |
| O75390 | Citrate synthase, mitochondrial | CS | -0.12 | 0.14 | -1.02 | -0.19 | 0.56 | 0.36 | 0.50 | -1.22 | -1.42 | 1.88 | 0.44 | 0.12 |
| Q92734 | Protein TFG | TFG | -0.12 | 0.12 | 0.38 | -0.73 | -0.12 | -0.28 | 0.91 | 0.47 | -2.38 | 0.94 | 0.01 | 0.79 |
| P40227 | T-complex protein 1 subunit zeta | CCT6A | -0.12 | 0.39 | -1.34 | 1.82 | -0.12 | 0.60 | -0.43 | -0.49 | 1.12 | -1.20 | 0.48 | -0.44 |
| P43490 | Nicotinamide phosphoribosyltransferase | NAMPT | -0.12 | 0.37 | 1.09 | 0.71 | -1.33 | 0.03 | -0.01 | 1.15 | -0.05 | -2.02 | 0.30 | 0.12 |
| P51553 | Isocitrate dehydrogenase [NAD] subunit gamma, mitochondrial | IDH3G | -0.12 | 0.11 | -1.63 | -0.16 | 1.16 | 0.53 | 0.25 | -1.71 | 0.94 | -0.46 | 0.56 | 0.53 |
| Q6P1N0 | Coiled-coil and C2 domain-containing protein 1A | CC2D1A | -0.12 | 0.11 | -0.34 | -0.54 | -1.34 | -0.04 | 2.43 | 0.37 | -0.36 | 0.59 | -0.47 | -0.29 |
| P53805 | Calcipressin-1 | RCAN1 | -0.12 | 0.13 | -0.36 | 1.25 | 0.20 | -0.02 | -0.88 | 1.08 | -2.06 | -0.34 | 0.17 | 0.96 |
| Q9NZN3 | EH domain-containing protein 3 | EHD3 | -0.12 | 0.25 | -1.18 | 2.01 | -0.85 | 0.07 | 0.30 | 0.75 | 0.55 | -1.36 | -0.11 | -0.18 |
| Q86YR5 | G-protein-signaling modulator 1 | GPSM1 | -0.12 | 0.12 | 0.84 | -0.32 | -0.50 | 0.47 | -0.30 | -1.67 | -0.13 | 2.03 | 0.36 | -0.78 |
| Q9HCE0 | Ectopic P granules protein 5 homolog | EPG5 | -0.13 | 0.16 | 1.44 | -0.59 | -1.01 | 1.05 | -0.66 | 1.45 | -0.60 | -1.00 | -0.58 | 0.50 |
| Q99592 | Zinc finger and BTB domain-containing protein 18 | ZBTB18 | -0.13 | 0.96 | 1.40 | 0.54 | -1.10 | 0.44 | -0.17 | 0.04 | -0.04 | -2.18 | 0.41 | 0.64 |
| P51797 | Chloride transport protein 6 | CLCN6 | -0.13 | 0.14 | 1.09 | 0.44 | 0.28 | -0.57 | -1.04 | 1.43 | 0.56 | -1.40 | -1.26 | 0.47 |
| Q9UKV0 | Histone deacetylase 9 | HDAC9 | -0.13 | 0.27 | -0.16 | -0.41 | -0.77 | 2.01 | -0.30 | -0.22 | -0.42 | -1.07 | 1.62 | -0.28 |
| P54764 | Ephrin type-A receptor 4 | EPHA4 | -0.13 | 0.27 | -0.43 | 1.68 | -1.15 | 0.94 | -0.67 | -0.29 | 0.45 | -1.47 | 0.00 | 0.95 |
| Q92696 | Geranylgeranyl transferase type-2 subunit alpha | RABGGTA | -0.13 | 0.18 | -1.23 | -0.50 | 1.86 | 0.82 | -0.69 | -1.03 | -0.63 | -0.09 | 0.90 | 0.59 |
| Q9H0Q0 | Protein FAM49A | FAM49A | -0.13 | 0.25 | -0.29 | 1.17 | -0.09 | 0.19 | -0.63 | 0.53 | 0.59 | -2.45 | 0.58 | 0.39 |
| Q99729 | Heterogeneous nuclear ribonucleoprotein A/B | HNRNPAB | -0.13 | 0.61 | 1.95 | 0.03 | -1.72 | 0.26 | 0.26 | -0.60 | -1.08 | -0.10 | 0.34 | 0.67 |
| Q9UIJ7 | GTP:AMP phosphotransferase AK3, mitochondrial | AK3 | -0.13 | 0.15 | -0.98 | -0.33 | 1.00 | 0.08 | 0.45 | -1.92 | -0.89 | 0.72 | 0.91 | 0.95 |
| P42694 | Probable helicase with zinc finger domain | HELZ | -0.13 | 0.11 | 1.67 | -0.68 | -0.77 | -0.65 | 0.61 | 1.48 | 0.61 | -1.00 | -0.65 | -0.61 |
| Q07020 | 60S ribosomal protein L18 | RPL18 | -0.13 | 0.10 | -0.89 | -1.47 | 0.42 | 0.27 | 1.81 | -0.88 | -0.62 | 1.05 | 0.47 | -0.17 |
| O75182 | Paired amphipathic helix protein Sin3b | SIN3B | -0.13 | 0.21 | 2.07 | -0.27 | -0.73 | -0.38 | -0.38 | 1.63 | -0.35 | -0.91 | -0.27 | -0.40 |
| Q8N4Q0 | Prostaglandin reductase 3 | ZADH2 | -0.13 | 0.12 | -1.67 | 0.37 | 1.26 | 0.36 | -0.15 | -1.71 | 0.13 | -0.16 | 1.06 | 0.50 |
| P49257 | Protein ERGIC-53 | LMAN1 | -0.13 | 0.13 | -0.06 | -0.95 | 1.11 | 0.18 | -0.09 | 0.77 | 0.01 | -2.30 | 0.83 | 0.49 |
| P61604 | 10 kDa heat shock protein, mitochondrial | HSPE1 | -0.13 | 0.27 | -1.86 | 0.70 | 0.14 | -0.04 | 1.44 | -1.59 | 0.45 | 0.35 | 0.15 | 0.27 |
| Q12931 | Heat shock protein 75 kDa, mitochondrial | TRAP1 | -0.13 | 0.22 | -0.89 | -0.55 | 1.97 | 0.17 | -0.40 | -1.03 | 0.45 | -1.15 | 0.37 | 1.04 |
| P43487 | Ran-specific GTPase-activating protein | RANBP1 | -0.13 | 0.28 | 0.41 | 1.40 | -1.44 | 0.32 | -0.31 | 0.70 | 0.25 | -1.95 | 0.15 | 0.46 |

| | | | | | | | | | | | | | | |
|---|---|---|---|---|---|---|---|---|---|---|---|---|---|---|
| P26640 | Valine--tRNA ligase | VARS | -0.13 | 0.30 | 0.55 | -2.04 | 2.07 | -0.17 | 0.02 | 0.25 | -0.31 | -0.16 | -0.15 | -0.05 |
| P18507 | Gamma-aminobutyric acid receptor subunit gamma-2 | GABRG2 | -0.13 | 0.34 | 0.92 | -2.28 | 0.83 | 0.05 | 0.95 | 0.55 | -0.85 | 0.20 | 0.15 | -0.51 |
| Q01082 | Spectrin beta chain, non-erythrocytic 1 | SPTBN1 | -0.13 | 0.27 | -0.19 | 1.40 | 0.35 | -0.87 | -0.31 | -0.13 | 1.72 | -1.35 | -1.03 | 0.41 |
| P00558 | Phosphoglycerate kinase 1 | PGK1 | -0.13 | 0.27 | -0.29 | 1.04 | 0.26 | 0.25 | -0.88 | 0.12 | -2.35 | 1.00 | 0.55 | 0.30 |
| Q01167 | Forkhead box protein K2 | FOXK2 | -0.13 | 0.17 | -0.86 | -0.36 | 0.02 | 1.13 | 0.32 | -1.04 | -0.94 | 2.16 | -0.08 | -0.34 |
| Q9NR56 | Muscleblind-like protein 1 | MBNL1 | -0.13 | 0.13 | 1.21 | -1.11 | -1.63 | 0.93 | 0.78 | 0.40 | 0.02 | -1.19 | 0.75 | -0.16 |
| P26373 | 60S ribosomal protein L13 | RPL13 | -0.13 | 0.42 | -0.54 | -1.67 | 0.37 | 1.49 | 0.91 | -0.98 | -0.42 | -0.62 | 0.99 | 0.48 |
| Q8NC96 | Adaptin ear-binding coat-associated protein 1 | NECAP1 | -0.13 | 0.32 | 0.47 | 2.06 | -1.74 | -0.36 | 0.01 | -0.55 | 0.90 | -0.38 | -0.09 | -0.32 |
| Q8WVJ2 | NudC domain-containing protein 2 | NUDCD2 | -0.13 | 0.25 | -0.01 | 0.85 | -1.78 | 0.20 | 1.08 | 0.90 | -0.98 | -1.24 | 0.26 | 0.71 |
| Q96FW1 | Ubiquitin thioesterase OTUB1 | OTUB1 | -0.14 | 0.26 | 0.48 | 0.78 | -0.65 | 0.69 | -0.94 | 0.54 | 0.98 | -2.05 | -0.58 | 0.75 |
| Q7L0J3 | Synaptic vesicle glycoprotein 2A | SV2A | -0.14 | 0.28 | -0.48 | 0.23 | -0.94 | 1.52 | 0.06 | -0.91 | -0.24 | -1.34 | 1.60 | 0.52 |
| P46531 | Neurogenic locus notch homolog protein 1 | NOTCH1 | -0.14 | 0.15 | 0.53 | -1.76 | 1.09 | 0.74 | -0.38 | 0.43 | -1.72 | 0.20 | 0.74 | 0.15 |
| Q6H8Q1 | Actin-binding LIM protein 2 | ABLIM2 | -0.14 | 0.37 | 0.23 | 1.17 | -1.02 | 0.52 | -0.40 | 0.48 | 0.33 | -2.29 | 0.30 | 0.67 |
| Q14894 | Ketimine reductase mu-crystallin | CRYM | -0.14 | 0.46 | -0.11 | -0.27 | 0.68 | 0.52 | -0.21 | -2.37 | 0.86 | 0.71 | 0.88 | -0.70 |
| Q02224 | Centromere-associated protein E | CENPE | -0.14 | 0.25 | -0.03 | 0.10 | -2.10 | 0.91 | 1.46 | 0.70 | -0.54 | -0.79 | 0.43 | -0.14 |
| Q9UPA5 | Protein bassoon | BSN | -0.14 | 0.19 | 0.04 | -1.06 | -0.44 | -0.55 | 2.27 | 0.52 | 0.93 | -0.33 | -0.79 | -0.59 |
| Q8IXJ6 | NAD-dependent protein deacetylase sirtuin-2 | SIRT2 | -0.14 | 0.28 | -0.49 | -0.14 | 0.67 | 0.41 | -0.07 | -1.55 | -0.79 | 1.91 | 0.86 | -0.82 |
| Q8TAQ2 | SWI/SNF complex subunit SMARCC2 | SMARCC2 | -0.14 | 0.18 | 0.17 | -2.18 | 1.98 | 0.06 | 0.24 | -0.04 | -0.12 | -0.29 | 0.32 | -0.14 |
| Q9NVD7 | Alpha-parvin | PARVA | -0.14 | 0.21 | -0.35 | -1.16 | -0.27 | -0.46 | 2.54 | 0.50 | -0.22 | -0.46 | -0.42 | 0.31 |
| O95202 | Mitochondrial proton/calcium exchanger protein | LETM1 | -0.14 | 1.27 | 1.71 | 0.72 | -1.13 | -0.17 | 0.24 | -0.11 | 0.55 | -1.92 | -0.25 | 0.35 |
| Q13418 | Integrin-linked protein kinase | ILK | -0.14 | 0.11 | 0.20 | 0.75 | -0.94 | 0.65 | -0.50 | 0.99 | -2.13 | -0.47 | 0.53 | 0.92 |
| P27105 | Erythrocyte band 7 integral membrane protein | STOM | -0.14 | 0.12 | -1.27 | 0.43 | 0.09 | 0.74 | 0.17 | -1.40 | -1.28 | 1.53 | 0.82 | 0.16 |
| Q8WYN3 | Cysteine/serine-rich nuclear protein 3 | CSRNP3 | -0.14 | 0.17 | -0.27 | -0.51 | -0.69 | -0.37 | 2.08 | -0.51 | -0.54 | 1.68 | -0.44 | -0.43 |
| Q15413 | Ryanodine receptor 3 | RYR3 | -0.14 | 0.30 | -0.84 | -0.18 | 0.33 | 0.80 | 0.29 | -0.83 | -2.11 | 0.80 | 0.62 | 1.12 |
| Q96AE4 | Far upstream element-binding protein 1 | FUBP1 | -0.14 | 0.44 | 1.59 | -0.88 | -1.20 | 0.35 | 0.73 | -1.31 | -0.40 | -0.55 | 0.70 | 0.97 |
| O75475 | PC4 and SFRS1-interacting protein | PSIP1 | -0.14 | 0.26 | 1.67 | -0.69 | 0.59 | -0.36 | -0.85 | 0.66 | 0.98 | -1.74 | -0.28 | 0.02 |
| P08047 | Transcription factor Sp1 | SP1 | -0.14 | 0.19 | -0.32 | -0.41 | -0.66 | -0.37 | 2.02 | -0.37 | -0.42 | -0.76 | 1.73 | -0.44 |
| Q16630 | Cleavage and polyadenylation specificity factor subunit 6 | CPSF6 | -0.14 | 0.16 | -0.52 | -0.01 | 0.82 | 0.94 | -1.01 | -2.10 | -0.21 | 0.41 | 1.09 | 0.58 |
| P21579 | Synaptotagmin-1 | SYT1 | -0.14 | 0.28 | 0.58 | 0.73 | -0.02 | -0.93 | 0.02 | -0.98 | 2.20 | -0.85 | -0.85 | 0.10 |
| O94804 | Serine/threonine-protein kinase 10 | STK10 | -0.14 | 0.55 | -1.20 | -0.29 | 0.79 | 0.76 | 0.63 | -1.69 | 0.00 | 1.30 | 0.64 | -0.96 |
| Q8TB36 | Ganglioside-induced differentiation-associated protein 1 | GDAP1 | -0.14 | 0.38 | 0.51 | 0.77 | 0.33 | -0.93 | -0.16 | 0.06 | 1.86 | -1.84 | -0.53 | -0.06 |
| Q8N987 | N-terminal EF-hand calcium-binding protein 1 | NECAB1 | -0.14 | 0.16 | 0.25 | -0.10 | -0.86 | -0.60 | 1.54 | -0.40 | 1.19 | -1.85 | 0.20 | 0.64 |
| Q96L34 | MAP/microtubule affinity-regulating kinase 4 | MARK4 | -0.14 | 0.13 | -0.26 | 0.57 | -0.25 | 0.32 | -0.20 | 1.10 | 1.37 | -2.23 | 0.13 | -0.57 |
| Q9UH03 | Neuronal-specific septin-3 | SEPTIN3 | -0.14 | 0.52 | 0.07 | 1.46 | -0.24 | 0.27 | -0.88 | 0.17 | 1.70 | -1.51 | -0.17 | -0.86 |
| Q66LE6 | Serine/threonine-protein phosphatase 2A 55 kDa regulatory subuni | PPP2R2D | -0.14 | 0.29 | 0.82 | 0.44 | -1.44 | -0.25 | 0.84 | 1.23 | -1.86 | -0.33 | 0.09 | 0.47 |
| P05165 | Propionyl-CoA carboxylase alpha chain, mitochondrial | PCCA | -0.14 | 0.12 | -1.09 | -1.18 | 0.39 | 0.25 | 1.80 | -1.08 | -0.54 | 1.10 | 0.53 | -0.19 |
| Q13620 | Cullin-4B | CUL4B | -0.14 | 0.99 | 1.62 | 0.06 | 1.19 | -0.25 | -1.48 | -1.27 | 0.06 | 0.87 | -0.44 | -0.37 |
| Q9UDW1 | Cytochrome b-c1 complex subunit 9 | UQCR10 | -0.14 | 1.43 | 1.67 | 0.66 | -1.08 | 0.47 | -0.21 | 0.13 | 0.08 | -2.05 | 0.34 | -0.01 |
| Q9UQ90 | Paraplegin | SPG7 | -0.14 | 0.21 | -0.28 | -0.40 | 1.81 | -0.37 | -0.47 | 1.94 | -0.55 | -0.84 | -0.43 | -0.41 |
| O95352 | Ubiquitin-like modifier-activating enzyme ATG7 | ATG7 | -0.14 | 0.52 | 1.06 | -1.51 | 1.12 | -0.22 | 0.21 | -0.55 | -0.88 | 1.57 | -0.83 | 0.01 |
| Q9Y6M9 | NADH dehydrogenase [ubiquinone] 1 beta subcomplex subunit 9 | NDUFB9 | -0.14 | 1.76 | 1.88 | -0.22 | -0.94 | 0.87 | 0.15 | 0.04 | 0.43 | -1.85 | -0.38 | 0.00 |
| Q13765 | Nascent polypeptide-associated complex subunit alpha | NACA | -0.14 | 0.36 | 0.72 | 0.39 | 0.63 | -0.72 | -0.54 | -0.68 | 0.14 | -1.33 | -0.72 | 2.09 |
| Q6ZN08 | Putative zinc finger protein 66 | ZNF66 | -0.15 | 0.29 | 0.98 | -1.49 | -1.28 | 1.12 | 1.06 | 0.46 | -0.88 | -0.26 | 0.74 | -0.45 |
| P36404 | ADP-ribosylation factor-like protein 2 | ARL2 | -0.15 | 0.46 | 0.46 | 0.00 | 0.51 | 0.52 | -0.87 | 0.11 | -0.52 | -2.25 | 0.87 | 1.18 |
| Q96KQ7 | Histone-lysine N-methyltransferase EHMT2 | EHMT2 | -0.15 | 0.18 | 0.78 | 0.32 | -2.49 | 0.65 | 0.99 | -0.14 | -0.33 | -0.50 | 0.19 | 0.52 |
| Q5T4S7 | E3 ubiquitin-protein ligase UBR4 | UBR4 | -0.15 | 0.36 | -0.24 | -0.74 | 0.54 | -0.20 | 1.13 | -1.65 | -0.74 | 1.88 | -0.05 | 0.07 |
| Q8NCJ5 | SPRY domain-containing protein 3 | SPRYD3 | -0.15 | 0.15 | -1.17 | 0.71 | -0.12 | 0.79 | 0.01 | 1.37 | 0.86 | -1.57 | 0.27 | -1.15 |
| Q9C0I1 | Myotubularin-related protein 12 | MTMR12 | -0.15 | 0.33 | -0.86 | -0.74 | 0.96 | -0.74 | 1.82 | -0.25 | -1.28 | 1.05 | -0.26 | 0.30 |
| P61927 | 60S ribosomal protein L37 | RPL37 | -0.15 | 0.17 | -0.03 | -1.32 | -0.59 | -0.12 | 2.29 | -0.28 | 0.80 | -0.36 | -0.80 | 0.40 |
| O60299 | Leucine zipper putative tumor suppressor 3 | LZTS3 | -0.15 | 0.36 | 1.32 | 0.65 | -1.02 | -0.71 | 0.24 | -0.46 | 1.47 | -1.54 | 0.52 | -0.47 |
| O75914 | Serine/threonine-protein kinase PAK 3 | PAK3 | -0.15 | 0.45 | 1.62 | 1.26 | 0.27 | -1.41 | -1.13 | -0.42 | -0.15 | 0.22 | -0.90 | 0.65 |
| P49589 | Cysteine--tRNA ligase, cytoplasmic | CARS | -0.15 | 0.36 | 1.21 | -0.99 | -0.60 | -0.52 | 1.38 | 1.00 | -1.33 | -0.22 | 0.81 | -0.74 |
| Q93009 | Ubiquitin carboxyl-terminal hydrolase 7 | USP7 | -0.15 | 0.46 | -1.36 | -0.03 | -0.47 | 0.10 | 2.37 | 0.62 | -0.90 | 0.07 | -0.23 | -0.16 |
| P19838 | Nuclear factor NF-kappa-B p105 subunit | NFKB1 | -0.15 | 0.17 | -0.79 | -0.38 | -0.76 | 0.90 | 1.26 | -0.68 | -1.69 | 0.55 | 1.22 | 0.35 |
| Q9NWY4 | Histone PARylation factor 1 | HPF1 | -0.15 | 0.28 | 2.12 | -0.38 | -0.77 | -0.19 | -0.40 | 1.53 | -0.33 | -0.96 | -0.32 | -0.30 |
| O95071 | E3 ubiquitin-protein ligase UBR5 | UBR5 | -0.15 | 0.18 | 0.66 | -0.14 | -0.13 | 0.03 | -0.15 | -2.24 | 0.72 | 1.53 | 0.41 | -0.68 |
| Q16576 | Histone-binding protein RBBP7 | RBBP7 | -0.15 | 0.38 | -1.25 | -0.12 | 0.50 | 0.43 | 0.95 | -0.74 | -1.93 | 0.36 | 0.74 | 1.06 |
| Q9NQ29 | Putative RNA-binding protein Luc7-like 1 | LUC7L | -0.15 | 0.18 | 0.82 | -0.61 | -1.51 | 0.08 | 1.47 | 0.49 | -1.29 | -0.57 | -0.01 | 1.12 |
| P62873 | Guanine nucleotide-binding protein G(I)/G(S)/G(T) subunit beta-1 | GNB1 | -0.15 | 0.49 | -0.35 | 2.28 | -1.39 | 0.66 | -0.54 | -0.44 | -0.15 | -0.74 | 0.47 | 0.21 |
| Q8N138 | ORM1-like protein 3 | ORMDL3 | -0.15 | 0.19 | 1.28 | 1.25 | -0.96 | -0.54 | -0.76 | 1.31 | -0.57 | -1.03 | -0.67 | 0.70 |
| Q6IAA8 | Ragulator complex protein LAMTOR1 | LAMTOR1 | -0.15 | 0.20 | -0.25 | 1.93 | -0.62 | -0.31 | -0.47 | -0.37 | -0.47 | -0.81 | -0.45 | 1.81 |
| P38159 | RNA-binding motif protein, X chromosome | RBMX | -0.15 | 0.40 | 1.49 | -0.57 | -1.07 | 0.59 | 0.10 | -1.35 | 1.25 | -1.14 | 0.24 | 0.46 |
| O95630 | STAM-binding protein | STAMBP | -0.15 | 0.23 | -0.34 | 0.01 | -0.65 | -0.44 | 1.74 | 0.74 | -1.91 | 0.97 | -0.31 | 0.19 |
| Q15654 | Thyroid receptor-interacting protein 6 | TRIP6 | -0.15 | 0.20 | -0.18 | -0.38 | -0.99 | 0.94 | 0.89 | 0.65 | -0.67 | -1.96 | 0.85 | 0.85 |
| Q9Y4C8 | Probable RNA-binding protein 19 | RBM19 | -0.15 | 0.22 | -0.31 | 1.20 | -0.89 | 0.93 | -0.63 | -0.56 | -0.65 | 2.01 | -0.52 | -0.59 |
| P02549 | Spectrin alpha chain, erythrocytic 1 | SPTA1 | -0.15 | 0.40 | 1.87 | -0.60 | -0.08 | -1.25 | 0.60 | -1.27 | 0.64 | 0.75 | -0.79 | 0.13 |
| P55160 | Nck-associated protein 1-like | NCKAP1L | -0.15 | 0.30 | 1.12 | -0.63 | -0.88 | 1.68 | -0.87 | -0.54 | -0.69 | 1.26 | -0.74 | 0.29 |
| Q8NEN9 | PDZ domain-containing protein 8 | PDZD8 | -0.15 | 0.16 | 1.23 | 0.61 | -1.72 | 0.45 | -0.33 | 0.73 | -1.45 | 0.97 | 0.04 | -0.52 |
| O60784 | Target of Myb protein 1 | TOM1 | -0.15 | 0.28 | -0.45 | 1.56 | -0.28 | -0.42 | -0.03 | -0.02 | 1.82 | -1.34 | -0.98 | 0.12 |
| P43686 | 26S proteasome regulatory subunit 6B | PSMC4 | -0.16 | 0.56 | 1.89 | 0.07 | -0.42 | -0.23 | -0.60 | 0.88 | 1.13 | -0.44 | -1.14 | -1.14 |
| Q04726 | Transducin-like enhancer protein 3 | TLE3 | -0.16 | 0.20 | 0.79 | 0.27 | -2.26 | 0.57 | 0.92 | -0.05 | 0.12 | -1.13 | 0.86 | -0.09 |
| P53992 | Protein transport protein Sec24C | SEC24C | -0.16 | 0.53 | -0.04 | 1.66 | -0.50 | 0.45 | -0.88 | -0.59 | -0.69 | -1.14 | 0.04 | 1.70 |
| Q8N6W0 | CUGBP Elav-like family member 5 | CELF5 | -0.16 | 0.29 | 0.21 | -1.29 | -0.17 | 0.86 | 0.79 | -0.66 | -0.39 | 1.60 | 0.59 | -1.55 |
| P98171 | Rho GTPase-activating protein 4 | ARHGAP4 | -0.16 | 0.12 | -0.65 | 0.64 | -1.10 | -0.60 | 1.89 | 0.98 | 0.06 | -1.10 | -0.70 | 0.59 |
| Q96D09 | G-protein coupled receptor-associated sorting protein 2 | GPRASP2 | -0.16 | 0.16 | 1.41 | -0.99 | -1.16 | 0.33 | 0.64 | 0.18 | 1.61 | -0.62 | -0.39 | -1.01 |
| Q86WW8 | Cytochrome c oxidase assembly factor 5 | COA5 | -0.16 | 1.43 | 1.65 | 0.58 | -1.01 | 0.39 | -0.10 | 0.30 | -0.07 | -2.12 | -0.04 | 0.42 |
| P61769 | Beta-2-microglobulin | B2M | -0.16 | 0.33 | 0.97 | 0.03 | -2.02 | 0.01 | 1.46 | 0.35 | -0.77 | -0.80 | 0.70 | 0.06 |
| P09622 | Dihydrolipoyl dehydrogenase, mitochondrial | DLD | -0.16 | 0.35 | 1.24 | -1.04 | 1.27 | -0.46 | -0.54 | 1.42 | -1.19 | 0.44 | -0.51 | -0.63 |
| O75787 | Renin receptor | ATP6AP2 | -0.16 | 0.20 | -1.00 | 0.36 | -0.09 | -0.58 | 1.60 | 0.61 | -1.46 | 1.36 | -0.02 | -0.78 |
| P98174 | FYVE, RhoGEF and PH domain-containing protein 1 | FGD1 | -0.16 | 0.18 | -0.46 | 1.13 | -0.82 | 1.26 | -0.85 | -0.69 | 1.03 | -1.02 | 1.18 | -0.75 |
| P29972 | Aquaporin-1 | AQP1 | -0.16 | 0.25 | 0.07 | 0.04 | 1.98 | -0.66 | -1.09 | 0.73 | -1.57 | 0.29 | 0.53 | -0.33 |
| Q8N414 | PiggyBac transposable element-derived protein 5 | PGBD5 | -0.16 | 0.87 | 0.68 | 1.17 | -1.93 | 0.56 | 0.55 | -0.86 | 0.79 | 0.53 | -0.93 | -0.57 |
| Q13875 | Myelin-associated oligodendrocyte basic protein | MOBP | -0.16 | 0.29 | -0.79 | 0.11 | 1.67 | 0.26 | -0.85 | -1.82 | 0.29 | 1.05 | 0.42 | -0.34 |

| | | | | | | | | | | | | | | |
|---|---|---|---|---|---|---|---|---|---|---|---|---|---|---|
| Q08170 | Serine/arginine-rich splicing factor 4 | SRSF4 | -0.16 | 0.42 | 0.38 | 0.15 | -0.39 | -0.90 | 1.30 | 0.80 | 1.59 | -1.23 | -0.97 | -0.74 |
| Q7L775 | EPM2A-interacting protein 1 | EPM2AIP1 | -0.16 | 0.34 | 0.61 | 0.56 | -1.28 | 0.78 | -0.20 | -1.18 | 1.53 | -1.15 | 0.85 | -0.51 |
| O43447 | Peptidyl-prolyl cis-trans isomerase H | PPIH | -0.16 | 0.31 | -1.10 | 0.80 | 0.78 | -0.17 | 0.11 | -1.31 | -1.57 | 0.37 | 1.12 | 0.97 |
| Q8WZ19 | BTB/POZ domain-containing adapter for CUL3-mediated RhoA deg | KCTD13 | -0.16 | 1.32 | 1.41 | 0.74 | -1.08 | 0.09 | 0.27 | 0.24 | 0.08 | -2.17 | -0.21 | 0.63 |
| Q9BQ61 | Telomerase RNA component interacting RNase | TRIR | -0.16 | 0.26 | 1.12 | 1.54 | -0.82 | -0.66 | -0.82 | -0.74 | 0.18 | 1.48 | -0.61 | -0.66 |
| P49006 | MARCKS-related protein | MARCKSL1 | -0.16 | 0.43 | -1.35 | 0.74 | 2.19 | -0.61 | -0.39 | -0.85 | -0.29 | 0.59 | -0.27 | 0.23 |
| Q9GZZ1 | N-alpha-acetyltransferase 50 | NAA50 | -0.16 | 0.27 | -1.70 | 1.30 | -0.32 | 0.50 | 0.59 | -1.71 | -0.13 | 0.42 | 0.36 | 0.68 |
| Q9BVP2 | Guanine nucleotide-binding protein-like 3 | GNL3 | -0.17 | 0.29 | 0.04 | -0.08 | -0.43 | 1.24 | -0.37 | -0.08 | -1.86 | -0.83 | 1.50 | 0.87 |
| Q13442 | 28 kDa heat- and acid-stable phosphoprotein | PDAP1 | -0.17 | 0.28 | 0.97 | 0.47 | 1.50 | -0.99 | -1.55 | 0.38 | 0.16 | 0.50 | -1.33 | -0.10 |
| Q969E4 | Transcription elongation factor A protein-like 3 | TCEAL3 | -0.17 | 0.13 | 0.11 | 0.91 | 0.89 | -1.37 | -0.36 | 0.24 | -0.12 | 1.22 | -1.91 | 0.38 |
| O15084 | Serine/threonine-protein phosphatase 6 regulatory ankyrin repeat | ANKRD28 | -0.17 | 0.19 | 0.62 | 0.37 | -1.65 | 0.74 | 0.19 | 0.90 | -0.22 | -1.94 | 0.66 | 0.34 |
| O75323 | Protein NipSnap homolog 2 | NIPSNAP2 | -0.17 | 0.12 | -0.06 | 0.87 | 1.08 | -1.75 | 0.02 | 0.35 | 0.25 | 0.31 | -1.79 | 0.72 |
| P62424 | 60S ribosomal protein L7a | RPL7A | -0.17 | 0.49 | 0.16 | -1.21 | 2.01 | -0.80 | 0.49 | -0.66 | 0.46 | -0.34 | -1.01 | 0.91 |
| P27987 | Inositol-trisphosphate 3-kinase B | ITPKB | -0.17 | 0.54 | 0.04 | 1.42 | -0.84 | 0.84 | -0.77 | 0.17 | -1.94 | 0.87 | 0.56 | -0.34 |
| Q8N1B4 | Vacuolar protein sorting-associated protein 52 homolog | VPS52 | -0.17 | 0.27 | 1.31 | 1.22 | -1.86 | 0.14 | -0.43 | 0.62 | 0.38 | -1.26 | 0.08 | -0.18 |
| Q9H7V2 | Synapse differentiation-inducing gene protein 1 | SYNDIG1 | -0.17 | 0.10 | 1.31 | -1.09 | -1.10 | 0.59 | 0.44 | 0.95 | 0.78 | -1.26 | 0.43 | -1.03 |
| P23284 | Peptidyl-prolyl cis-trans isomerase B | PPIB | -0.17 | 0.82 | -0.30 | 0.83 | 1.32 | 0.49 | -1.37 | 0.52 | -0.08 | -1.00 | 1.02 | -1.43 |
| P62308 | Small nuclear ribonucleoprotein G | SNRPG | -0.17 | 0.38 | 1.30 | -1.60 | 1.19 | -0.65 | 0.25 | -0.55 | -1.17 | 0.38 | -0.16 | 1.00 |
| P60981 | Destrin | DSTN | -0.17 | 0.53 | 0.66 | 0.08 | -0.56 | 1.16 | -0.66 | -0.03 | 0.12 | -2.24 | 1.14 | 0.33 |
| P62847 | 40S ribosomal protein S24 | RPS24 | -0.17 | 0.27 | -1.76 | 0.53 | -0.34 | 0.02 | 1.92 | -1.07 | -0.47 | 0.45 | 0.38 | 0.34 |
| Q5JSL3 | Dedicator of cytokinesis protein 11 | DOCK11 | -0.17 | 0.20 | -0.45 | 1.23 | -0.84 | 1.17 | -0.83 | -0.69 | -0.67 | -1.10 | 0.96 | 1.21 |
| Q8NEL9 | Phospholipase DDHD1 | DDHD1 | -0.17 | 0.38 | -0.35 | 1.80 | -0.95 | 0.71 | -0.70 | 0.71 | 1.00 | -1.31 | -0.23 | -0.68 |
| P35269 | General transcription factor IIF subunit 1 | GTF2F1 | -0.17 | 0.45 | -0.06 | 0.78 | 0.92 | -0.58 | -0.46 | -0.72 | 1.95 | -1.45 | 0.31 | -0.69 |
| Q8IWB9 | Testis-expressed protein 2 | TEX2 | -0.17 | 0.18 | -2.05 | 0.18 | 1.19 | 0.77 | 0.16 | -0.65 | -1.14 | 0.56 | 0.82 | 0.15 |
| Q14978 | Nucleolar and coiled-body phosphoprotein 1 | NOLC1 | -0.17 | 0.40 | -0.92 | 1.29 | 0.21 | 0.86 | -0.92 | -0.45 | -1.74 | -0.12 | 0.96 | 0.82 |
| Q9C0C2 | 182 kDa tankyrase-1-binding protein | TNKS1BP1 | -0.17 | 0.13 | 1.47 | 0.15 | -0.93 | -0.03 | -0.47 | 1.57 | 0.90 | -1.11 | -0.79 | -0.76 |
| P11908 | Ribose-phosphate pyrophosphokinase 2 | PRPS2 | -0.17 | 0.25 | -0.13 | -0.40 | -0.69 | 2.07 | -0.50 | -0.36 | -0.43 | -0.91 | 1.61 | -0.26 |
| Q53HC9 | EARP and GARP complex-interacting protein 1 | EIPR1 | -0.17 | 0.28 | 1.90 | -0.29 | -0.61 | 0.45 | -1.06 | 1.22 | -0.01 | -1.04 | 0.39 | -0.95 |
| Q01970 | 1-phosphatidylinositol 4,5-bisphosphate phosphodiesterase beta-3 | PLCB3 | -0.18 | 0.20 | -0.75 | 0.45 | 0.76 | -1.39 | 1.22 | 0.61 | 0.97 | -1.65 | 0.16 | -0.38 |
| Q8N2C7 | Protein unc-80 homolog | UNC80 | -0.18 | 0.20 | 1.29 | -0.22 | -1.60 | 0.23 | 0.57 | 0.69 | 1.41 | -0.89 | -0.76 | -0.72 |
| P41594 | Metabotropic glutamate receptor 5 | GRM5 | -0.18 | 0.40 | 1.42 | -1.45 | -0.74 | 0.50 | 0.80 | -1.62 | -0.31 | 0.49 | 0.69 | 0.21 |
| Q9HD20 | Manganese-transporting ATPase 13A1 | ATP13A1 | -0.18 | 0.14 | 0.91 | 0.71 | -1.28 | 0.01 | -0.14 | 0.67 | 0.93 | -2.15 | 0.05 | 0.28 |
| Q9UHD8 | Septin-9 | SEPTIN9 | -0.18 | 0.75 | 0.20 | 1.85 | 0.65 | -0.20 | -1.58 | 0.68 | 0.48 | -0.77 | -1.11 | -0.19 |
| Q13094 | Lymphocyte cytosolic protein 2 | LCP2 | -0.18 | 0.23 | 1.62 | 1.07 | -0.91 | -0.63 | -0.82 | -0.51 | 0.97 | -1.00 | -0.64 | 0.86 |
| P27701 | CD82 antigen | CD82 | -0.18 | 0.19 | -0.92 | 0.45 | -0.08 | -0.51 | 1.34 | -0.49 | 0.14 | 1.96 | -0.81 | -1.07 |
| Q13619 | Cullin-4A | CUL4A | -0.18 | 0.77 | 0.86 | 1.25 | -0.76 | 0.56 | -0.97 | -0.04 | -0.13 | -1.94 | 0.16 | 1.02 |
| P54253 | Ataxin-1 | ATXN1 | -0.18 | 0.22 | 0.93 | -1.05 | -1.18 | 0.67 | 0.94 | 0.45 | -0.95 | -1.38 | 0.94 | 0.63 |
| P41227 | N-alpha-acetyltransferase 10 | NAA10 | -0.18 | 0.70 | -1.04 | 0.16 | 0.71 | -0.23 | 1.26 | -2.13 | -0.40 | 0.42 | 0.84 | 0.41 |
| P59998 | Actin-related protein 2/3 complex subunit 4 | ARPC4 | -0.18 | 0.45 | 0.49 | 1.66 | -1.22 | 0.93 | -1.27 | -0.37 | 0.56 | -1.12 | 0.54 | -0.20 |
| Q99943 | 1-acyl-sn-glycerol-3-phosphate acyltransferase alpha | AGPAT1 | -0.18 | 0.65 | 0.27 | 1.68 | -0.19 | 0.59 | -1.53 | -1.53 | -0.25 | 0.96 | 0.20 | -0.20 |
| Q15437 | Protein transport protein Sec23B | SEC23B | -0.18 | 0.79 | -0.01 | 0.83 | -1.62 | 0.61 | 1.13 | 0.07 | -0.14 | -1.87 | 0.59 | 0.40 |
| Q8NEY8 | Periphilin-1 | PPHLN1 | -0.18 | 1.24 | 0.43 | -0.25 | -0.99 | -0.06 | 2.23 | 0.37 | -0.32 | -1.59 | 0.09 | 0.08 |
| P28715 | DNA repair protein complementing XP-G cells | ERCC5 | -0.18 | 1.64 | 1.16 | 0.61 | -0.87 | 0.53 | 0.24 | 0.70 | 0.33 | -2.34 | -0.29 | -0.09 |
| Q9UFB7 | Zinc finger and BTB domain-containing protein 47 | ZBTB47 | -0.18 | 0.27 | 0.80 | -0.91 | -0.95 | 0.90 | 0.53 | -0.66 | -0.79 | -1.21 | 0.72 | 1.58 |
| O14531 | Dihydropyrimidinase-related protein 4 | DPYSL4 | -0.18 | 0.68 | 0.33 | 0.57 | -1.13 | -0.35 | 1.43 | 0.07 | 0.94 | 0.40 | -0.25 | -2.00 |
| O75569 | Interferon-inducible double-stranded RNA-dependent protein kinas | PRKRA | -0.18 | 0.53 | 1.33 | -1.12 | -1.09 | 1.08 | 0.49 | -1.19 | 0.93 | -0.78 | 0.65 | -0.30 |
| Q96BM9 | ADP-ribosylation factor-like protein 8A | ARL8A | -0.18 | 1.17 | -0.38 | 1.12 | 0.16 | 0.89 | -0.48 | -1.34 | 1.06 | -1.74 | 0.78 | -0.07 |
| P04264 | Keratin, type II cytoskeletal 1 | KRT1 | -0.19 | 0.17 | 0.09 | -1.17 | -0.32 | 1.03 | 0.61 | 1.63 | 0.90 | -0.96 | -0.71 | -1.09 |
| O60879 | Protein diaphanous homolog 2 | DIAPH2 | -0.19 | 0.23 | -1.36 | 1.12 | -0.16 | 0.72 | 0.00 | -0.45 | 0.90 | -1.90 | 0.36 | 0.77 |
| Q9Y2W6 | Tudor and KH domain-containing protein | TDRKH | -0.19 | 0.16 | 1.92 | 0.27 | -0.98 | -0.13 | -0.84 | 1.24 | 0.49 | -1.08 | -0.79 | -0.10 |
| O00459 | Phosphatidylinositol 3-kinase regulatory subunit beta | PIK3R2 | -0.19 | 0.20 | 2.06 | -0.47 | -0.56 | -0.33 | -0.41 | -0.46 | 1.70 | -0.72 | -0.44 | -0.37 |
| Q9NZW5 | MAGUK p55 subfamily member 6 | MPP6 | -0.19 | 0.32 | 0.68 | 0.35 | -0.82 | 0.54 | -0.32 | 0.75 | 0.59 | -2.48 | 0.23 | 0.47 |
| Q08211 | ATP-dependent RNA helicase A | DHX9 | -0.19 | 0.32 | -0.99 | 1.29 | -0.71 | 0.98 | -0.13 | -0.29 | -1.75 | -0.20 | 1.10 | 0.70 |
| P07203 | Glutathione peroxidase 1 | GPX1 | -0.19 | 0.14 | -0.37 | 0.26 | 1.20 | -0.28 | -0.60 | -1.17 | -1.26 | 1.96 | -0.10 | 0.35 |
| P54277 | PMS1 protein homolog 1 | PMS1 | -0.19 | 0.15 | -0.34 | -0.49 | 2.02 | -0.47 | -0.50 | -0.49 | -0.48 | 1.77 | -0.48 | -0.52 |
| P46736 | Lys-63-specific deubiquitinase BRCC36 | BRCC3 | -0.19 | 0.57 | -0.49 | -1.14 | 2.03 | 0.75 | -0.42 | -1.24 | -0.68 | 0.64 | 0.42 | 0.12 |
| O15371 | Eukaryotic translation initiation factor 3 subunit D | EIF3D | -0.19 | 0.48 | -0.76 | 1.30 | 0.12 | 0.56 | -0.61 | 0.19 | -0.27 | -2.14 | 0.66 | 0.94 |
| Q8WXA3 | RUN and FYVE domain-containing protein 2 | RUFY2 | -0.19 | 0.38 | 0.08 | -0.21 | -0.79 | 0.37 | 1.05 | 0.84 | -2.43 | 0.51 | 0.19 | 0.38 |
| P49711 | Transcriptional repressor CTCF | CTCF | -0.19 | 0.25 | 1.40 | -1.70 | -0.65 | 0.87 | 0.41 | 0.74 | 0.48 | -0.98 | 0.38 | -0.95 |
| Q00577 | Transcriptional activator protein Pur-alpha | PURA | -0.19 | 1.10 | 1.55 | -0.47 | -0.64 | 1.16 | -0.37 | -1.47 | 0.39 | -1.14 | 0.91 | 0.07 |
| Q9BYK8 | Helicase with zinc finger domain 2 | HELZ2 | -0.19 | 0.13 | 1.26 | -0.18 | -1.31 | -0.31 | 0.72 | 1.17 | 0.07 | 0.94 | -1.18 | -1.18 |
| Q9Y243 | RAC-gamma serine/threonine-protein kinase | AKT3 | -0.19 | 0.46 | -0.01 | 0.38 | 0.30 | 0.40 | -0.45 | -0.16 | -2.50 | 1.37 | 0.23 | 0.44 |
| P32004 | Neural cell adhesion molecule L1 | L1CAM | -0.19 | 0.43 | 1.22 | 1.00 | -0.03 | -1.71 | 0.10 | 0.60 | -0.07 | -1.01 | -1.10 | 1.01 |
| Q96RR4 | Calcium/calmodulin-dependent protein kinase kinase 2 | CAMKK2 | -0.19 | 0.34 | -0.04 | 0.38 | 0.24 | 0.18 | -0.29 | 2.27 | -1.57 | 0.15 | -0.35 | -0.97 |
| P20585 | DNA mismatch repair protein Msh3 | MSH3 | -0.19 | 1.74 | 1.50 | 0.60 | -0.89 | 0.16 | 0.36 | -0.14 | 0.29 | -2.26 | 0.49 | -0.11 |
| Q9UQB3 | Catenin delta-2 | CTNND2 | -0.19 | 0.46 | -2.06 | 1.38 | 1.37 | 0.27 | -0.36 | -0.26 | -0.71 | -0.09 | 0.01 | 0.43 |
| Q16620 | BDNF/NT-3 growth factors receptor | NTRK2 | -0.19 | 0.16 | -2.05 | 1.04 | -0.37 | 0.89 | 0.72 | -1.14 | 0.15 | -0.45 | 0.73 | 0.49 |
| O00399 | Dynactin subunit 6 | DCTN6 | -0.19 | 0.51 | 0.75 | 0.51 | -0.48 | 1.24 | -1.37 | 0.39 | -1.81 | -0.41 | 0.95 | 0.22 |
| P49841 | Glycogen synthase kinase-3 beta | GSK3B | -0.19 | 0.27 | -1.28 | 0.49 | 0.43 | 0.49 | 0.25 | -1.95 | 1.45 | -0.60 | 0.57 | 0.15 |
| Q9Y4K1 | Beta/gamma crystallin domain-containing protein 1 | CRYBG1 | -0.20 | 0.68 | 0.07 | -1.82 | 0.25 | 0.34 | 2.01 | -0.60 | -0.92 | 0.19 | 0.04 | 0.44 |
| P30405 | Peptidyl-prolyl cis-trans isomerase F, mitochondrial | PPIF | -0.20 | 0.29 | -1.62 | -0.35 | 0.80 | 0.73 | 0.83 | -1.13 | -1.28 | 0.37 | 0.75 | 0.89 |
| P84074 | Neuron-specific calcium-binding protein hippocalcin | HPCA | -0.20 | 0.21 | -1.89 | 0.63 | 0.16 | 0.23 | 1.16 | -1.59 | 0.49 | 0.91 | -0.13 | 0.02 |
| Q9Y3Q8 | TSC22 domain family protein 4 | TSC22D4 | -0.20 | 0.19 | 0.66 | 1.09 | -1.44 | 0.06 | -0.10 | -1.28 | -1.31 | 0.83 | 0.48 | 1.01 |
| Q9UPN6 | SR-related and CTD-associated factor 8 | SCAF8 | -0.20 | 0.28 | -1.20 | 0.23 | -0.41 | 1.05 | 0.71 | -1.35 | -1.41 | 0.83 | 0.64 | 0.89 |
| P02689 | Myelin P2 protein | PMP2 | -0.20 | 0.22 | -0.44 | -0.99 | 2.29 | 0.26 | -0.81 | -0.09 | -1.09 | 0.50 | 0.59 | -0.22 |
| P69892 | Hemoglobin subunit gamma-2 | HBG2 | -0.20 | 0.15 | -1.65 | 0.86 | 0.04 | 0.62 | 0.35 | -1.65 | -0.35 | 1.34 | 0.59 | -0.15 |
| Q58FF8 | Putative heat shock protein HSP 90-beta 2 | HSP90AB2P | -0.20 | 0.51 | -0.09 | -0.35 | -0.96 | 0.33 | 1.74 | -0.32 | -0.49 | -1.14 | -0.45 | 1.73 |
| O60861 | Growth arrest-specific protein 7 | GAS7 | -0.20 | 0.62 | -0.57 | 2.05 | 0.12 | -0.59 | -0.23 | -0.66 | 1.32 | -1.29 | -0.40 | 0.26 |
| Q9Y570 | Protein phosphatase methylesterase 1 | PPME1 | -0.20 | 0.54 | 0.06 | 1.58 | -0.31 | -0.23 | -0.42 | 0.48 | 0.87 | -2.25 | -0.12 | 0.33 |
| Q53T59 | HCLS1-binding protein 3 | HS1BP3 | -0.20 | 0.46 | -0.71 | 0.80 | -0.59 | 0.81 | 0.31 | -0.53 | -2.04 | -0.17 | 1.13 | 1.00 |
| Q14166 | Tubulin--tyrosine ligase-like protein 12 | TTLL12 | -0.20 | 0.39 | 1.17 | -0.31 | -1.67 | 1.14 | 0.19 | 0.51 | -1.45 | -0.54 | 0.84 | 0.13 |
| Q8WVM8 | Sec1 family domain-containing protein 1 | SCFD1 | -0.20 | 0.18 | -2.29 | 0.18 | 0.66 | 0.26 | 1.46 | -0.76 | -0.45 | 0.37 | 0.32 | 0.25 |
| O14682 | Ectoderm-neural cortex protein 1 | ENC1 | -0.20 | 0.13 | 0.28 | -0.63 | -0.67 | 0.41 | 0.81 | -0.63 | 2.36 | -0.79 | -0.59 | -0.55 |

| | | | | | | | | | | | | | | |
|---|---|---|---|---|---|---|---|---|---|---|---|---|---|---|
| Q9NWF9 | E3 ubiquitin-protein ligase RNF216 | RNF216 | -0.21 | 0.35 | -0.16 | -0.36 | -0.74 | -0.40 | 2.15 | -0.57 | -0.49 | 1.57 | -0.54 | -0.44 |
| Q15819 | Ubiquitin-conjugating enzyme E2 variant 2 | UBE2V2 | -0.21 | 0.81 | -0.51 | 1.48 | -0.48 | -0.22 | 0.72 | -1.92 | -0.45 | 1.36 | 0.02 | 0.02 |
| P05534 | HLA class I histocompatibility antigen, A-24 alpha chain | HLA-A | -0.21 | 0.17 | -0.50 | 1.45 | -0.05 | 0.84 | -1.51 | 0.45 | 0.24 | -1.71 | 0.69 | 0.09 |
| Q92974 | Rho guanine nucleotide exchange factor 2 | ARHGEF2 | -0.21 | 0.35 | 0.40 | 0.31 | -1.71 | 1.11 | 0.36 | 0.57 | -0.73 | -1.54 | 1.09 | 0.14 |
| O75436 | Vacuolar protein sorting-associated protein 26A | VPS26A | -0.21 | 1.20 | 1.24 | 1.33 | 0.62 | 0.26 | -2.09 | 0.14 | 0.04 | -0.52 | -0.67 | -0.37 |
| Q9NXA8 | NAD-dependent protein deacylase sirtuin-5, mitochondrial | SIRT5 | -0.21 | 1.07 | -0.04 | 1.46 | 0.83 | 0.76 | -1.78 | -0.16 | -1.36 | -0.34 | 0.68 | -0.06 |
| Q6YN16 | Hydroxysteroid dehydrogenase-like protein 2 | HSDL2 | -0.21 | 0.22 | -1.27 | 0.13 | 0.01 | 0.57 | 0.86 | -1.29 | -1.54 | 1.15 | 0.54 | 0.82 |
| A0FGR8 | Extended synaptotagmin-2 | ESYT2 | -0.21 | 0.30 | -0.42 | 0.21 | -0.45 | -0.18 | 1.24 | 0.87 | 0.39 | -2.41 | 0.45 | 0.29 |
| O75882 | Attractin | ATRN | -0.21 | 0.26 | 0.71 | 0.40 | -1.18 | -0.91 | 1.35 | -0.85 | 0.82 | 1.25 | -0.97 | -0.62 |
| Q9NP78 | ATP-binding cassette sub-family B member 9 | ABCB9 | -0.21 | 0.32 | -0.21 | 1.14 | -0.39 | 1.13 | -1.23 | -0.15 | -1.53 | -0.66 | 1.21 | 0.69 |
| Q9GZZ9 | Ubiquitin-like modifier-activating enzyme 5 | UBA5 | -0.21 | 0.41 | 0.64 | 1.24 | -1.22 | 0.55 | -0.67 | 0.60 | 0.65 | -1.99 | 0.23 | -0.04 |
| Q5VWJ9 | Sorting nexin-30 | SNX30 | -0.21 | 0.18 | 1.47 | 0.62 | -1.52 | -0.33 | 0.02 | 1.16 | 0.59 | -1.50 | -0.25 | -0.25 |
| P23443 | Ribosomal protein S6 kinase beta-1 | RPS6KB1 | -0.21 | 0.38 | -0.25 | 2.30 | -0.65 | -0.43 | -0.47 | -0.37 | -0.28 | -0.84 | -0.34 | 1.33 |
| Q9BTW9 | Tubulin-specific chaperone D | TBCD | -0.21 | 0.52 | 2.03 | 0.06 | -0.90 | -0.50 | -0.01 | 0.76 | 0.64 | -1.66 | -0.16 | -0.25 |
| Q9HAT2 | Sialate O-acetylesterase | SIAE | -0.21 | 0.23 | 1.33 | 0.06 | -2.01 | 0.48 | 0.45 | -1.10 | -0.36 | 1.19 | -0.07 | 0.01 |
| O75582 | Ribosomal protein S6 kinase alpha-5 | RPS6KA5 | -0.21 | 0.66 | 0.77 | -1.17 | -0.31 | 0.40 | 1.13 | -0.60 | -1.62 | 1.50 | -0.40 | 0.30 |
| Q9Y4P3 | Transducin beta-like protein 2 | TBL2 | -0.21 | 0.19 | 1.16 | -0.95 | -1.13 | 0.50 | 0.70 | 0.75 | 1.16 | -1.32 | -1.04 | 0.17 |
| Q96SW2 | Protein cereblon | CRBN | -0.21 | 0.65 | 1.33 | -0.59 | -0.65 | 1.01 | -0.29 | 0.68 | -1.40 | -1.29 | 1.12 | 0.08 |
| O60443 | Gasdermin-E | GSDME | -0.21 | 0.30 | -1.65 | 0.11 | 1.07 | 0.32 | 0.55 | -0.08 | -1.91 | 0.79 | 0.65 | 0.14 |
| Q5VWP3 | Muscular LMNA-interacting protein | MLIP | -0.21 | 0.26 | 1.66 | 1.10 | -1.22 | 0.06 | -1.24 | 0.24 | -1.14 | 0.77 | -0.29 | 0.06 |
| Q9UNZ5 | Leydig cell tumor 10 kDa protein homolog | C19orf53 | -0.21 | 0.38 | -1.02 | 0.53 | 0.46 | -0.25 | 0.78 | 1.63 | -1.84 | -0.04 | -0.73 | 0.47 |
| P00167 | Cytochrome b5 | CYB5A | -0.21 | 0.30 | -0.11 | 2.09 | -0.69 | -0.34 | -0.55 | -0.42 | -0.38 | -0.76 | -0.47 | 1.62 |
| Q9NZ52 | ADP-ribosylation factor-binding protein GGA3 | GGA3 | -0.21 | 0.46 | 0.74 | 0.70 | -0.66 | 1.69 | -1.85 | 0.36 | 0.32 | -0.30 | -0.03 | -0.96 |
| Q9NZR1 | Tropomodulin-2 | TMOD2 | -0.21 | 0.53 | 1.79 | 0.92 | -0.37 | -1.14 | -0.52 | 1.02 | 0.37 | -0.22 | -1.30 | -0.56 |
| P08263 | Glutathione S-transferase A1 | GSTA1 | -0.21 | 0.30 | 2.42 | -0.44 | -0.66 | -0.41 | -0.50 | -0.34 | -0.34 | 1.20 | -0.50 | -0.44 |
| P55786 | Puromycin-sensitive aminopeptidase | NPEPPS | -0.21 | 0.66 | 0.69 | 2.06 | -1.04 | -0.24 | -0.63 | 1.26 | -0.49 | -0.34 | -0.52 | -0.73 |
| Q8WUR7 | UPF0235 protein C15orf40 | C15orf40 | -0.21 | 0.24 | -1.30 | -0.02 | 0.90 | 0.40 | 0.36 | -1.40 | -1.40 | 0.82 | 1.17 | 0.47 |
| O00625 | Pirin | PIR | -0.21 | 0.70 | 0.18 | -0.50 | 1.76 | 0.08 | -0.65 | 1.25 | -1.78 | -0.12 | 0.36 | -0.58 |
| P55809 | Succinyl-CoA:3-ketoacid coenzyme A transferase 1, mitochondria | OXCT1 | -0.21 | 0.35 | -0.84 | 0.33 | 2.17 | -0.34 | -0.85 | -1.31 | 0.93 | -0.08 | -0.13 | 0.12 |
| O43822 | Cilia- and flagella-associated protein 410 | CFAP410 | -0.22 | 0.34 | 1.24 | 0.12 | -0.91 | -0.03 | 0.05 | 1.87 | -1.32 | -1.17 | 0.05 | 0.12 |
| Q9NZM1 | Myoferlin | MYOF | -0.22 | 0.70 | -0.61 | 1.49 | 0.09 | -1.10 | 0.98 | -0.88 | 1.16 | -0.82 | -0.99 | 0.68 |
| Q9BVC4 | Target of rapamycin complex subunit LST8 | MLST8 | -0.22 | 0.16 | 0.42 | 0.18 | -1.58 | 0.90 | 0.31 | 1.53 | -0.43 | -1.67 | 0.18 | 0.17 |
| Q7L7L0 | Histone H2A type 3 | HIST3H2A | -0.22 | 0.32 | 0.67 | -1.97 | 1.49 | 0.12 | 0.13 | 0.28 | -1.39 | -0.17 | 0.45 | 0.39 |
| P07996 | Thrombospondin-1 | THBS1 | -0.22 | 0.30 | 0.47 | 0.79 | -1.21 | 1.26 | -0.90 | 0.15 | -1.01 | -1.31 | 0.99 | 0.77 |
| Q53GG5 | PDZ and LIM domain protein 3 | PDLIM3 | -0.22 | 0.42 | 0.84 | 1.84 | -1.01 | -0.09 | -1.02 | -0.02 | -0.71 | -0.38 | -0.73 | 1.29 |
| P47756 | F-actin-capping protein subunit beta | CAPZB | -0.22 | 0.90 | -0.36 | 1.30 | 0.48 | 0.73 | -1.10 | -1.96 | -0.52 | 0.86 | 0.59 | -0.03 |
| Q16512 | Serine/threonine-protein kinase N1 | PKN1 | -0.22 | 0.26 | 0.14 | 0.56 | -1.24 | 0.71 | 0.20 | 1.32 | 0.07 | -1.48 | -1.28 | 1.01 |
| Q9NT62 | Ubiquitin-like-conjugating enzyme ATG3 | ATG3 | -0.22 | 0.76 | -0.48 | 1.37 | -0.87 | 1.03 | -0.14 | -0.37 | -1.01 | -1.16 | 0.03 | 1.59 |
| Q9NSB4 | Keratin, type II cuticular Hb2 | KRT82 | -0.22 | 0.14 | -0.73 | 1.58 | -0.89 | 0.28 | -0.04 | -0.81 | -0.82 | 1.84 | 0.16 | -0.57 |
| Q99615 | DnaJ homolog subfamily C member 7 | DNAJC7 | -0.22 | 0.46 | -0.13 | 1.11 | -0.83 | 0.39 | 0.07 | -0.16 | 0.78 | -2.34 | 0.24 | 0.87 |
| Q9Y279 | V-set and immunoglobulin domain-containing protein 4 | VSIG4 | -0.22 | 1.42 | 0.71 | 2.04 | -0.93 | -0.31 | 0.00 | -0.17 | -0.08 | -1.78 | 0.11 | 0.42 |
| O75683 | Surfeit locus protein 6 | SURF6 | -0.22 | 0.20 | -0.57 | -1.60 | 0.26 | 0.49 | 1.70 | 0.35 | 0.31 | 0.29 | 0.35 | -1.58 |
| Q9Y2Z0 | Protein SGT1 homolog | SUGT1 | -0.22 | 0.74 | -0.40 | 1.81 | 0.07 | 0.41 | -0.98 | -0.17 | 0.75 | -1.89 | 0.50 | -0.10 |
| O75145 | Liprin-alpha-3 | PPFIA3 | -0.22 | 0.31 | -1.16 | 0.90 | -0.14 | 0.83 | 0.00 | -0.69 | -1.81 | -0.08 | 0.94 | 1.22 |
| Q8TBF2 | Prostamide/prostaglandin F synthase | PRXL2B | -0.22 | 0.27 | -1.44 | 0.99 | 0.80 | 0.52 | -0.50 | 0.74 | 0.48 | -0.11 | 0.46 | -1.94 |
| P17812 | CTP synthase 1 | CTPS1 | -0.22 | 0.42 | 0.40 | 0.43 | -0.90 | 0.15 | 0.46 | -1.49 | 2.08 | -0.95 | 0.17 | -0.37 |
| P01034 | Cystatin-C | CST3 | -0.22 | 0.62 | 0.27 | 0.30 | -0.12 | 1.09 | -0.76 | -1.30 | -1.30 | 1.40 | 1.11 | -0.70 |
| Q8IZP0 | Abl interactor 1 | ABI1 | -0.22 | 0.74 | 1.24 | 0.52 | -0.48 | 0.15 | -0.52 | 1.90 | -0.52 | -1.19 | 0.06 | -1.18 |
| Q00765 | Receptor expression-enhancing protein 5 | REEP5 | -0.22 | 0.54 | 0.29 | -0.53 | -0.63 | 0.80 | 0.77 | -0.85 | -0.93 | 2.19 | -0.51 | -0.61 |
| O95232 | Luc7-like protein 3 | LUC7L3 | -0.22 | 0.50 | 0.30 | -0.87 | -0.89 | -0.36 | 2.49 | 0.06 | 0.29 | -0.45 | -0.83 | 0.26 |
| Q8IWZ6 | Bardet-Biedl syndrome 7 protein | BBS7 | -0.22 | 0.47 | 1.54 | -0.27 | -0.94 | 0.22 | 0.06 | -0.16 | -0.87 | -1.51 | 0.37 | 1.56 |
| Q8TB72 | Pumilio homolog 2 | PUM2 | -0.22 | 0.30 | 0.70 | -1.06 | -0.62 | 0.25 | 1.15 | -1.03 | 1.86 | -1.03 | -0.06 | -0.15 |
| Q99523 | Sortilin | SORT1 | -0.22 | 0.31 | 1.43 | -0.62 | 0.05 | 0.33 | -0.75 | -0.63 | -1.43 | 1.63 | 0.64 | -0.63 |
| P60903 | Protein S100-A10 | S100A10 | -0.22 | 0.19 | -0.91 | 1.37 | 0.62 | -1.07 | 0.26 | -1.10 | -1.08 | 1.09 | -0.22 | 1.05 |
| Q96B18 | Dapper homolog 3 | DACT3 | -0.22 | 0.29 | 2.19 | -0.50 | -0.53 | -0.28 | -0.48 | -0.35 | -0.44 | -0.74 | -0.40 | 1.54 |
| Q96L92 | Sorting nexin-27 | SNX27 | -0.22 | 0.59 | 1.34 | -0.24 | -0.87 | 0.80 | -0.27 | 0.74 | -1.07 | -1.76 | 0.72 | 0.62 |
| Q96RE7 | Nucleus accumbens-associated protein 1 | NACC1 | -0.22 | 0.21 | 1.05 | -0.74 | -0.85 | -0.61 | 1.44 | -0.71 | 1.14 | -1.04 | -0.64 | 0.95 |
| Q8IYB5 | Stromal membrane-associated protein 1 | SMAP1 | -0.23 | 0.50 | 0.14 | 1.58 | -1.16 | 0.60 | -0.53 | 0.23 | 0.79 | -1.90 | 0.42 | -0.19 |
| Q9NRS6 | Sorting nexin-15 | SNX15 | -0.23 | 0.33 | 1.40 | -0.62 | -1.05 | -0.14 | 0.85 | 0.60 | 0.12 | 0.93 | -0.20 | -1.88 |
| Q6P1J9 | Parafibromin | CDC73 | -0.23 | 0.68 | 0.66 | 0.84 | -0.97 | 0.33 | 0.00 | 0.91 | -0.11 | -2.39 | 0.26 | 0.48 |
| Q96PY5 | Formin-like protein 2 | FMNL2 | -0.23 | 0.41 | -1.23 | -0.80 | 1.99 | 0.13 | 0.46 | -0.29 | -1.45 | 0.50 | 0.37 | 0.33 |
| Q13555 | Calcium/calmodulin-dependent protein kinase type II subunit gam | CAMK2G | -0.23 | 0.41 | 1.53 | -1.09 | -1.41 | 0.65 | 0.86 | 0.15 | -0.31 | -1.32 | 0.56 | 0.37 |
| P21246 | Pleiotrophin | PTN | -0.23 | 0.29 | 0.81 | 1.03 | 0.68 | -0.29 | -1.84 | -0.52 | 0.88 | -1.13 | -0.50 | 0.87 |
| Q96GY0 | Zinc finger C2HC domain-containing protein 1A | ZC2HC1A | -0.23 | 0.34 | 1.18 | 1.22 | -0.71 | 0.42 | -1.65 | 0.09 | 0.91 | -1.35 | 0.07 | -0.19 |
| P26368 | Splicing factor U2AF 65 kDa subunit | U2AF2 | -0.23 | 0.61 | 0.19 | -0.85 | -0.95 | 0.12 | 2.27 | -0.87 | -0.77 | 0.86 | -0.21 | 0.22 |
| Q9BVA1 | Tubulin beta-2B chain | TUBB2B | -0.23 | 0.28 | -0.14 | 0.17 | 0.13 | -0.03 | 0.27 | 1.31 | -2.56 | -0.17 | 0.44 | 0.59 |
| Q92777 | Synapsin-2 | SYN2 | -0.23 | 0.38 | 0.27 | 1.53 | -0.96 | 0.21 | -0.54 | -1.35 | 1.71 | -0.75 | 0.04 | -0.16 |
| Q9UNW9 | RNA-binding protein Nova-2 | NOVA2 | -0.23 | 0.36 | 0.93 | -0.93 | -1.38 | 0.76 | 1.09 | -0.11 | -1.51 | 1.11 | 0.18 | -0.16 |
| Q9Y4J8 | Dystrobrevin alpha | DTNA | -0.23 | 0.64 | -0.56 | 1.84 | 0.59 | 0.52 | -1.58 | 0.78 | -0.52 | -1.14 | 0.07 | 0.01 |
| P50747 | Biotin--protein ligase | HLCS | -0.23 | 0.40 | -0.19 | 2.04 | -0.72 | 0.12 | -0.71 | -0.55 | 1.22 | -1.21 | -0.49 | 0.51 |
| Q9H4L5 | Oxysterol-binding protein-related protein 3 | OSBPL3 | -0.23 | 0.27 | 1.04 | -0.49 | -1.95 | 0.90 | 0.88 | 0.15 | 0.14 | -1.33 | 0.70 | -0.05 |
| Q9H2K8 | Serine/threonine-protein kinase TAO3 | TAOK3 | -0.23 | 0.21 | -1.75 | 1.00 | 1.14 | 0.38 | -0.48 | -1.05 | -1.03 | 0.40 | 0.64 | 0.74 |
| Q8IZF6 | Adhesion G-protein coupled receptor G4 | ADGRG4 | -0.23 | 0.46 | -0.66 | -0.71 | -0.19 | 0.11 | 2.05 | -0.61 | -1.42 | 1.08 | 0.47 | -0.12 |
| Q14721 | Potassium voltage-gated channel subfamily B member 1 | KCNB1 | -0.23 | 0.51 | 0.99 | -0.07 | 0.79 | -0.52 | -0.51 | 0.75 | 1.60 | -1.46 | -1.22 | -0.34 |
| P50570 | Dynamin-2 | DNM2 | -0.23 | 0.34 | -1.49 | 0.52 | 0.10 | 0.15 | 1.18 | -0.66 | -1.47 | 1.50 | 0.43 | -0.26 |
| P47985 | Cytochrome b-c1 complex subunit Rieske, mitochondrial | UQCRFS1 | -0.23 | 0.31 | -1.59 | 1.07 | 0.85 | -0.07 | 0.17 | 0.30 | -0.10 | -0.26 | 1.29 | -1.65 |
| Q13554 | Calcium/calmodulin-dependent protein kinase type II subunit beta | CAMK2B | -0.23 | 0.48 | 1.23 | -0.94 | -1.52 | 1.06 | 0.80 | -0.23 | 0.62 | -1.37 | 0.45 | -0.10 |
| P53609 | Geranylgeranyl transferase type-1 subunit beta | PGGT1B | -0.23 | 0.37 | -0.06 | 1.06 | 1.27 | -0.21 | -1.57 | 0.62 | -1.41 | -0.84 | 0.42 | 0.72 |
| P62820 | Ras-related protein Rab-1A | RAB1A | -0.23 | 0.91 | 1.14 | 1.77 | -1.34 | -0.33 | -0.17 | 0.26 | 0.88 | -0.74 | -1.02 | -0.46 |
| P84103 | Serine/arginine-rich splicing factor 3 | SRSF3 | -0.23 | 0.88 | 0.43 | -0.83 | -0.10 | -0.16 | 1.71 | -1.33 | -1.39 | 0.19 | 0.36 | 1.14 |
| Q9H492 | Microtubule-associated proteins 1A/1B light chain 3A | MAP1LC3A | -0.23 | 0.92 | -0.12 | 0.90 | 0.07 | 1.33 | -1.11 | -1.79 | -0.18 | 0.15 | 1.27 | -0.54 |
| Q92530 | Proteasome inhibitor PI31 subunit | PSMF1 | -0.24 | 0.72 | 0.10 | -0.37 | 0.38 | 0.97 | -0.18 | 0.81 | -1.59 | -1.65 | 1.33 | 0.22 |
| O43790 | Keratin, type II cuticular Hb6 | KRT86 | -0.24 | 0.15 | -0.39 | 1.86 | -0.20 | -0.45 | -0.60 | -0.51 | -0.56 | 1.91 | -0.55 | -0.50 |

| | | | | | | | | | | | | | | |
|---|---|---|---|---|---|---|---|---|---|---|---|---|---|---|
| P36776 | Lon protease homolog, mitochondrial | LONP1 | -0.24 | 0.69 | -0.56 | 0.71 | 0.48 | 0.35 | -0.12 | -1.28 | 0.96 | -1.95 | 0.20 | 1.20 |
| Q9P2P5 | E3 ubiquitin-protein ligase HECW2 | HECW2 | -0.24 | 0.33 | -0.25 | 1.07 | -1.10 | 0.96 | -0.23 | -0.84 | 1.26 | -1.38 | 1.05 | -0.54 |
| Q9UQF2 | C-Jun-amino-terminal kinase-interacting protein 1 | MAPK8IP1 | -0.24 | 0.38 | 1.06 | 0.08 | 0.85 | -0.67 | -0.81 | 1.33 | 0.80 | -1.82 | -0.40 | -0.42 |
| P16452 | Erythrocyte membrane protein band 4.2 | EPB42 | -0.24 | 0.36 | -0.99 | 1.39 | -1.38 | 0.75 | 0.71 | -1.21 | 0.83 | -0.49 | 0.76 | -0.37 |
| P40855 | Peroxisomal biogenesis factor 19 | PEX19 | -0.24 | 0.31 | 0.14 | 1.82 | -0.99 | 0.39 | -0.94 | -0.76 | -0.71 | 0.73 | -0.84 | 1.16 |
| Q8IXI2 | Mitochondrial Rho GTPase 1 | RHOT1 | -0.24 | 0.43 | 0.95 | 0.14 | -0.28 | 0.07 | -0.30 | 1.70 | -1.85 | -1.13 | 0.13 | 0.56 |
| Q5VIR6 | Vacuolar protein sorting-associated protein 53 homolog | VPS53 | -0.24 | 0.28 | -0.95 | 1.23 | -1.35 | 0.38 | 1.08 | -1.09 | 0.87 | -1.04 | 0.39 | 0.49 |
| Q9UEE9 | Craniofacial development protein 1 | CFDP1 | -0.24 | 0.37 | -0.19 | -0.25 | -0.53 | -0.80 | 2.27 | -0.41 | -0.42 | -0.76 | 1.35 | -0.25 |
| Q92608 | Dedicator of cytokinesis protein 2 | DOCK2 | -0.24 | 0.36 | 1.10 | 0.54 | -1.26 | 0.41 | -0.30 | -0.27 | 1.69 | -1.49 | 0.24 | -0.65 |
| Q01085 | Nucleolysin TIAR | TIAL1 | -0.24 | 0.62 | 1.09 | -0.59 | -1.12 | 0.13 | 1.27 | -0.40 | 0.78 | -1.87 | 0.18 | 0.52 |
| Q92804 | TATA-binding protein-associated factor 2N | TAF15 | -0.24 | 0.55 | 2.30 | -0.63 | -0.98 | 0.24 | -0.22 | -1.27 | 0.44 | 0.38 | 0.24 | -0.49 |
| Q96JB5 | CDK5 regulatory subunit-associated protein 3 | CDK5RAP3 | -0.24 | 0.39 | -0.61 | -0.40 | -0.56 | -0.63 | 2.72 | 0.05 | 0.24 | -0.47 | -0.24 | -0.10 |
| Q9NPI6 | mRNA-decapping enzyme 1A | DCP1A | -0.24 | 0.40 | 2.10 | -0.11 | -0.69 | -0.28 | -0.49 | -0.10 | -0.45 | -1.07 | 1.50 | -0.42 |
| Q9Y277 | Voltage-dependent anion-selective channel protein 3 | VDAC3 | -0.24 | 0.44 | 1.02 | -0.47 | 0.76 | 0.09 | -0.81 | -1.56 | 0.60 | -1.42 | 0.76 | 1.04 |
| Q8WXH2 | Junctophilin-3 | JPH3 | -0.24 | 0.36 | 1.11 | 0.51 | -0.91 | 1.22 | -1.44 | -0.41 | 0.56 | -1.49 | 0.30 | 0.56 |
| Q6NZI2 | Caveolae-associated protein 1 | CAVIN1 | -0.24 | 0.26 | 0.71 | 1.19 | 0.53 | -0.89 | -1.18 | 0.49 | -0.75 | -1.46 | 0.07 | 1.30 |
| P51798 | H(+)/Cl(-) exchange transporter 7 | CLCN7 | -0.24 | 0.40 | 0.51 | 0.02 | -1.61 | 0.72 | 0.89 | -0.13 | -0.47 | -1.69 | 0.54 | 1.21 |
| Q9Y250 | Leucine zipper putative tumor suppressor 1 | LZTS1 | -0.24 | 0.27 | 0.20 | -1.70 | -0.34 | 1.05 | 1.17 | -0.73 | -1.30 | 0.32 | 1.05 | 0.29 |
| Q9NRA0 | Sphingosine kinase 2 | SPHK2 | -0.24 | 0.26 | 0.31 | 0.51 | -1.71 | 0.80 | 0.45 | 0.18 | -0.26 | -1.86 | 0.98 | 0.60 |
| Q6SZW1 | Sterile alpha and TIR motif-containing protein 1 | SARM1 | -0.24 | 0.35 | -0.22 | 2.19 | -0.62 | -0.49 | -0.40 | 1.52 | -0.41 | -0.74 | -0.53 | -0.31 |
| Q3MHD2 | Protein LSM12 homolog | LSM12 | -0.24 | 0.24 | 0.75 | 0.56 | -1.94 | -0.02 | 0.98 | 0.78 | 0.43 | -1.44 | -0.57 | 0.46 |
| P15882 | N-chimaerin | CHN1 | -0.24 | 0.50 | 0.16 | 0.31 | -1.50 | 1.18 | 0.51 | -0.22 | -1.22 | -1.10 | 1.41 | 0.49 |
| Q9H9G7 | Protein argonaute-3 | AGO3 | -0.24 | 0.29 | 0.63 | 1.40 | -1.55 | -0.53 | 0.44 | -1.32 | 0.73 | -0.91 | 0.38 | 0.72 |
| O75061 | Putative tyrosine-protein phosphatase auxilin | DNAJC6 | -0.24 | 0.65 | 0.11 | 1.70 | -0.60 | 0.56 | -0.95 | -0.23 | 1.19 | -1.74 | -0.09 | 0.05 |
| Q4KMP7 | TBC1 domain family member 10B | TBC1D10B | -0.25 | 0.35 | -0.35 | 1.80 | -0.41 | 0.44 | -1.01 | -1.70 | -0.49 | 0.31 | 0.58 | 0.83 |
| Q8IZQ5 | Selenoprotein H | SELENOH | -0.25 | 0.70 | 0.70 | -1.41 | -0.62 | 0.35 | 1.85 | 0.96 | -0.24 | -1.24 | -0.07 | -0.28 |
| O00273 | DNA fragmentation factor subunit alpha | DFFA | -0.25 | 0.19 | -1.03 | 1.29 | 0.45 | 0.69 | -1.13 | -1.15 | -1.15 | 0.33 | 0.62 | 1.08 |
| P38935 | DNA-binding protein SMUBP-2 | IGHMBP2 | -0.25 | 0.26 | 0.61 | -0.98 | -0.35 | -0.19 | 1.27 | 0.24 | 1.79 | -1.29 | -0.14 | -0.97 |
| P36507 | Dual specificity mitogen-activated protein kinase kinase 2 | MAP2K2 | -0.25 | 0.45 | 0.66 | 0.55 | -1.20 | 0.72 | -0.13 | 0.48 | 0.39 | -2.35 | 0.42 | 0.46 |
| Q9NQG5 | Regulation of nuclear pre-mRNA domain-containing protein 1B | RPRD1B | -0.25 | 0.28 | 0.66 | 0.30 | -1.28 | 1.04 | -0.33 | 0.79 | -1.19 | -1.53 | 0.93 | 0.62 |
| Q9NZC7 | WW domain-containing oxidoreductase | WWOX | -0.25 | 0.35 | 1.04 | 1.11 | -1.01 | -0.61 | -0.06 | 1.59 | -0.66 | -1.14 | -0.83 | 0.57 |
| O15143 | Actin-related protein 2/3 complex subunit 1B | ARPC1B | -0.25 | 0.28 | 1.57 | 0.82 | -0.21 | -0.31 | -1.48 | 0.81 | 0.80 | -1.38 | -0.58 | -0.03 |
| P38606 | V-type proton ATPase catalytic subunit A | ATP6V1A | -0.25 | 0.80 | 0.87 | -0.28 | 0.35 | 0.66 | -0.62 | -0.92 | 1.37 | -0.36 | 0.84 | -1.92 |
| Q969M7 | NEDD8-conjugating enzyme UBE2F | UBE2F | -0.25 | 0.28 | -1.07 | -0.75 | 1.16 | 1.02 | 0.03 | -1.18 | -1.30 | 1.13 | 0.70 | 0.26 |
| O60502 | Protein O-GlcNAcase | OGA | -0.25 | 0.90 | 1.10 | 1.19 | -1.62 | 0.65 | -0.27 | -1.09 | 0.36 | 0.16 | 0.71 | -1.19 |
| Q5JSP0 | FYVE, RhoGEF and PH domain-containing protein 3 | FGD3 | -0.25 | 0.26 | 0.03 | -0.25 | 1.68 | -0.95 | -0.15 | 0.97 | 1.08 | -1.39 | -1.07 | 0.04 |
| O75179 | Ankyrin repeat domain-containing protein 17 | ANKRD17 | -0.25 | 0.14 | -0.95 | -1.05 | 0.69 | 1.08 | 0.43 | -1.46 | -1.07 | 0.66 | 1.00 | 0.68 |
| Q9UPY3 | Endoribonuclease Dicer | DICER1 | -0.25 | 0.54 | 0.08 | -0.48 | 0.95 | -0.90 | 1.04 | 1.23 | -0.23 | -1.75 | 0.84 | -0.79 |
| P28676 | Grancalcin | GCA | -0.25 | 0.34 | -1.21 | 0.75 | 0.63 | 0.19 | 0.10 | -1.43 | -1.54 | 0.77 | 0.94 | 0.80 |
| P36896 | Activin receptor type-1B | ACVR1B | -0.25 | 0.35 | 2.14 | -0.42 | -0.49 | -0.29 | -0.47 | -0.44 | -0.35 | -0.87 | 1.57 | -0.39 |
| Q9UHJ6 | Sedoheptulokinase | SHPK | -0.25 | 0.33 | -0.08 | -0.17 | 0.31 | 0.75 | -0.36 | 0.72 | -0.47 | -2.44 | 0.85 | 0.88 |
| O75368 | SH3 domain-binding glutamic acid-rich-like protein | SH3BGRL | -0.25 | 0.34 | 0.24 | 0.05 | 0.35 | 0.34 | -0.51 | -0.40 | -2.35 | 1.58 | 0.28 | 0.43 |
| P15498 | Proto-oncogene vav | VAV1 | -0.25 | 0.38 | 0.68 | 0.99 | -2.24 | 0.00 | 1.09 | -0.51 | -0.25 | -0.04 | 0.85 | -0.57 |
| Q8TCB0 | Interferon-induced protein 44 | IFI44 | -0.25 | 0.39 | 0.14 | -1.26 | 0.36 | 1.31 | -0.03 | 0.63 | -1.35 | 0.65 | 0.96 | -1.41 |
| P61086 | Ubiquitin-conjugating enzyme E2 K | UBE2K | -0.25 | 0.50 | 0.82 | -0.70 | 0.45 | -0.17 | 0.26 | -0.28 | -2.22 | 1.59 | 0.07 | 0.19 |
| P63279 | SUMO-conjugating enzyme UBC9 | UBE2I | -0.25 | 0.48 | -0.83 | -0.06 | -0.91 | 0.78 | 1.65 | -1.52 | -0.88 | 0.63 | 0.50 | 0.65 |
| Q92995 | Ubiquitin carboxyl-terminal hydrolase 13 | USP13 | -0.25 | 0.15 | 1.27 | -0.05 | 0.26 | -0.18 | -1.09 | 1.09 | 1.38 | -1.27 | -0.27 | -1.14 |
| P15311 | Ezrin | EZR | -0.26 | 0.36 | -0.61 | -0.88 | -0.39 | -0.30 | 2.69 | -0.43 | -0.25 | 0.28 | -0.17 | 0.08 |
| Q96A49 | Synapse-associated protein 1 | SYAP1 | -0.26 | 0.28 | -0.24 | 0.83 | 0.48 | 1.20 | -1.89 | -0.08 | 0.62 | -1.17 | 0.90 | -0.65 |
| Q96S90 | LysM and putative peptidoglycan-binding domain-containing protei | LYSMD1 | -0.26 | 0.35 | -1.25 | -0.24 | 0.92 | 0.86 | 0.20 | -0.92 | -1.78 | 0.54 | 0.84 | 0.85 |
| Q8WU76 | Sec1 family domain-containing protein 2 | SCFD2 | -0.26 | 0.34 | -1.46 | 0.46 | 0.17 | 1.46 | -0.16 | -0.14 | -1.43 | -0.75 | 0.69 | 1.18 |
| Q9H2H8 | Peptidyl-prolyl cis-trans isomerase-like 3 | PPIL3 | -0.26 | 0.46 | -0.46 | 1.64 | -0.85 | 0.72 | -0.45 | 0.66 | -0.46 | -1.84 | 0.73 | 0.31 |
| Q6PIU2 | Neutral cholesterol ester hydrolase 1 | NCEH1 | -0.26 | 0.27 | 1.41 | 0.69 | -0.11 | -0.12 | -1.49 | 0.34 | 1.13 | -1.69 | -0.26 | 0.10 |
| Q9BVA0 | Katanin p80 WD40 repeat-containing subunit B1 | KATNB1 | -0.26 | 0.84 | 1.20 | 0.72 | -0.97 | 0.53 | -0.47 | 0.56 | 0.40 | -2.25 | 0.28 | 0.01 |
| Q8N4C8 | Misshapen-like kinase 1 | MINK1 | -0.26 | 0.54 | -1.30 | 0.59 | 0.44 | 0.95 | 0.02 | -1.66 | -0.66 | -0.54 | 0.86 | 1.29 |
| P13667 | Protein disulfide-isomerase A4 | PDIA4 | -0.26 | 0.21 | -1.25 | 0.60 | 0.72 | 0.66 | -0.42 | -1.58 | -1.15 | 0.53 | 1.05 | 0.85 |
| Q15742 | NGFI-A-binding protein 2 | NAB2 | -0.26 | 0.38 | 2.05 | -0.19 | -1.12 | 0.26 | -0.48 | 0.84 | -0.75 | -1.30 | 0.23 | 0.46 |
| Q86YD1 | Prostate tumor-overexpressed gene 1 protein | PTOV1 | -0.26 | 0.74 | -0.54 | 0.03 | -0.68 | 2.55 | -0.44 | -0.13 | -0.30 | -0.89 | 0.73 | -0.34 |
| Q00839 | Heterogeneous nuclear ribonucleoprotein U | HNRNPU | -0.26 | 0.62 | 1.52 | -1.11 | -1.26 | 0.69 | 0.94 | 0.60 | -1.18 | -0.67 | 0.55 | -0.09 |
| O75037 | Kinesin-like protein KIF21B | KIF21B | -0.26 | 0.26 | 1.13 | 0.59 | -2.38 | 0.49 | 0.54 | 0.35 | -0.53 | -0.58 | 0.62 | -0.21 |
| Q96KG9 | N-terminal kinase-like protein | SCYL1 | -0.26 | 0.41 | 0.80 | 1.21 | -0.17 | -0.97 | -0.32 | 0.96 | 1.19 | -0.72 | -1.70 | -0.27 |
| P13929 | Beta-enolase | ENO3 | -0.26 | 0.37 | 0.07 | -0.49 | -0.14 | -0.14 | 1.21 | -0.29 | -0.93 | 2.26 | -0.72 | -0.82 |
| Q8IZD9 | Dedicator of cytokinesis protein 3 | DOCK3 | -0.26 | 0.20 | 0.77 | 0.65 | -1.15 | 0.41 | -0.40 | 1.07 | 1.05 | -1.79 | 0.29 | -0.89 |
| Q86YV0 | RAS protein activator like-3 | RASAL3 | -0.26 | 0.29 | 1.67 | 0.76 | -1.01 | -0.11 | -0.91 | 1.21 | 0.67 | -0.46 | -0.96 | -0.86 |
| P14415 | Sodium/potassium-transporting ATPase subunit beta-2 | ATP1B2 | -0.26 | 0.68 | -0.39 | 0.41 | 0.96 | 0.76 | -0.89 | 0.37 | -1.87 | 1.03 | 0.71 | -1.10 |
| Q9NVJ2 | ADP-ribosylation factor-like protein 8B | ARL8B | -0.27 | 1.13 | 1.15 | 1.33 | -1.24 | 0.73 | -0.71 | -0.10 | 0.29 | -1.75 | -0.10 | 0.39 |
| Q9NSC2 | Sal-like protein 1 | SALL1 | -0.27 | 0.29 | 0.49 | -0.74 | -0.96 | 0.09 | 1.51 | 1.18 | 1.09 | -1.05 | -0.77 | -0.85 |
| Q9NRR5 | Ubiquilin-4 | UBQLN4 | -0.27 | 0.35 | 1.46 | -0.76 | 1.26 | -0.61 | -0.88 | -0.71 | -0.83 | 1.21 | 0.58 | -0.73 |
| Q4AE62 | Glycosyltransferase-like domain-containing protein 1 | GTDC1 | -0.27 | 0.30 | 2.17 | -0.44 | -0.55 | -0.32 | -0.46 | 1.56 | -0.40 | -0.69 | -0.46 | -0.42 |
| Q9P2K5 | Myelin expression factor 2 | MYEF2 | -0.27 | 0.35 | 1.13 | -0.95 | -0.41 | 0.32 | 0.39 | 1.02 | -0.68 | -2.00 | 0.30 | 0.88 |
| Q9NNW7 | Thioredoxin reductase 2, mitochondrial | TXNRD2 | -0.27 | 0.50 | -0.40 | -0.05 | 1.55 | -0.27 | -0.18 | -0.24 | -1.91 | 0.68 | -0.55 | 1.36 |
| Q7Z739 | YTH domain-containing family protein 3 | YTHDF3 | -0.27 | 0.31 | -0.06 | -0.91 | -1.11 | 0.98 | 1.54 | -0.04 | 0.77 | -1.24 | -0.81 | 0.90 |
| Q8TF74 | WAS/WASL-interacting protein family member 2 | WIPF2 | -0.27 | 0.76 | -0.71 | 1.04 | -0.84 | -0.35 | 1.79 | 0.45 | -1.71 | 0.39 | -0.23 | 0.19 |
| Q9Y6D5 | Brefeldin A-inhibited guanine nucleotide-exchange protein 2 | ARFGEF2 | -0.27 | 0.54 | 0.55 | 0.08 | -1.17 | 1.87 | -0.64 | -1.30 | 0.33 | -0.80 | 0.97 | 0.11 |
| P62899 | 60S ribosomal protein L31 | RPL31 | -0.27 | 0.65 | -0.22 | -0.80 | 1.91 | 0.27 | -0.35 | -1.12 | 0.52 | -1.22 | -0.18 | 1.19 |
| Q15286 | Ras-related protein Rab-35 | RAB35 | -0.27 | 0.91 | 0.06 | 0.57 | 0.11 | 0.52 | -0.15 | -2.56 | 0.08 | -0.41 | 0.84 | 0.95 |
| Q14004 | Cyclin-dependent kinase 13 | CDK13 | -0.27 | 0.50 | -2.21 | 0.46 | 0.85 | 0.74 | 0.82 | -0.13 | -0.02 | -1.17 | 0.76 | -0.09 |
| Q16566 | Calcium/calmodulin-dependent protein kinase type IV | CAMK4 | -0.27 | 0.53 | 0.91 | -0.05 | -1.68 | 0.86 | 0.63 | 0.50 | 0.68 | -1.80 | 0.34 | -0.39 |
| P01024 | Complement C3 | C3 | -0.27 | 0.34 | -1.41 | -0.10 | 0.28 | -0.16 | 1.84 | -1.30 | -0.83 | 0.94 | 0.36 | 0.37 |
| P21741 | Midkine | MDK | -0.27 | 0.45 | 0.66 | 1.08 | -0.70 | -0.49 | 0.04 | 1.41 | -0.78 | 1.13 | -1.09 | -1.27 |
| Q9BU76 | Multiple myeloma tumor-associated protein 2 | MMTAG2 | -0.27 | 0.39 | 1.31 | -1.33 | -0.76 | -0.52 | 1.83 | -0.34 | 0.66 | 0.28 | -0.68 | -0.45 |
| P31327 | Carbamoyl-phosphate synthase [ammonia], mitochondrial | CPS1 | -0.27 | 0.22 | -0.86 | -0.21 | 2.27 | 0.21 | -1.09 | -0.29 | -1.10 | 0.64 | 0.36 | 0.08 |
| O95466 | Formin-like protein 1 | FMNL1 | -0.27 | 0.26 | 1.39 | 0.52 | 0.83 | -0.43 | -1.95 | 0.68 | -0.17 | -1.14 | -0.24 | 0.50 |

| | | | | | | | | | | | | | | |
|---|---|---|---|---|---|---|---|---|---|---|---|---|---|---|
| P61328 | Fibroblast growth factor 12 | FGF12 | -0.27 | 0.79 | -0.29 | 1.76 | 0.30 | 0.84 | -1.65 | -0.38 | -1.07 | -0.62 | 0.43 | 0.68 |
| Q14849 | StAR-related lipid transfer protein 3 | STARD3 | -0.27 | 0.59 | 0.62 | -0.67 | 1.24 | 0.48 | -0.93 | 1.26 | -0.90 | -1.35 | 0.92 | -0.68 |
| Q9NRZ5 | 1-acyl-sn-glycerol-3-phosphate acyltransferase delta | AGPAT4 | -0.27 | 0.39 | 2.47 | -0.77 | -0.04 | 0.07 | -1.19 | 0.20 | 0.42 | -0.73 | -0.33 | -0.09 |
| P78509 | Reelin | RELN | -0.27 | 0.30 | 1.09 | -1.14 | -1.25 | 0.86 | 0.85 | 0.93 | 0.86 | -0.60 | -0.44 | -1.15 |
| Q14980 | Nuclear mitotic apparatus protein 1 | NUMA1 | -0.27 | 0.20 | -1.38 | 0.66 | 0.27 | 0.26 | 0.48 | -1.99 | -0.51 | 1.27 | 0.46 | 0.48 |
| Q6P1X6 | UPF0598 protein C8orf82 | C8orf82 | -0.27 | 0.29 | -0.57 | 1.33 | -0.79 | 1.14 | -0.72 | -0.64 | -0.73 | -1.07 | 1.40 | 0.64 |
| Q8IWE2 | Protein NOXP20 | FAM114A1 | -0.27 | 0.44 | 0.52 | 1.15 | -1.03 | 0.65 | -0.71 | -0.73 | -0.77 | -1.26 | 0.69 | 1.49 |
| Q16851 | UTP--glucose-1-phosphate uridylyltransferase | UGP2 | -0.27 | 2.11 | 0.04 | 1.08 | 0.15 | 0.60 | 0.20 | -2.18 | -0.87 | 0.92 | 0.77 | -0.70 |
| Q9ULU4 | Protein kinase C-binding protein 1 | ZMYND8 | -0.27 | 0.56 | 0.65 | -1.99 | 0.79 | 0.26 | 1.00 | 0.45 | 0.12 | -1.55 | 0.49 | -0.23 |
| P62913 | 60S ribosomal protein L11 | RPL11 | -0.27 | 1.05 | 0.91 | -0.33 | 0.73 | 0.37 | -0.46 | -2.13 | -1.07 | 0.81 | 0.31 | 0.86 |
| O95777 | U6 snRNA-associated Sm-like protein LSm8 | LSM8 | -0.27 | 0.43 | 1.96 | -0.14 | -0.71 | -0.07 | -0.49 | 0.33 | -1.74 | 1.02 | -0.39 | 0.22 |
| Q9UPW0 | Forkhead box protein J3 | FOXJ3 | -0.27 | 0.80 | -0.41 | 0.84 | 0.42 | -0.84 | 0.96 | 1.25 | 0.81 | -1.53 | -1.31 | -0.18 |
| Q9NP79 | Vacuolar protein sorting-associated protein VTA1 homolog | VTA1 | -0.27 | 0.47 | -0.01 | 1.97 | -1.19 | 0.42 | -0.57 | 0.12 | 0.31 | -1.70 | 0.33 | 0.33 |
| O00182 | Galectin-9 | LGALS9 | -0.27 | 0.34 | 0.10 | 0.99 | -1.87 | 0.63 | 0.61 | 0.25 | -1.69 | -0.23 | 0.74 | 0.47 |
| Q96QG7 | Myotubularin-related protein 9 | MTMR9 | -0.27 | 0.69 | -1.62 | 0.97 | -0.36 | 0.88 | 0.99 | -0.75 | 0.22 | 0.17 | 0.94 | -1.42 |
| P52209 | 6-phosphogluconate dehydrogenase, decarboxylating | PGD | -0.27 | 0.61 | 0.01 | -0.76 | 0.85 | 0.09 | 0.59 | -0.27 | -2.02 | 1.69 | 0.31 | -0.50 |
| P52789 | Hexokinase-2 | HK2 | -0.27 | 0.38 | 1.80 | -0.56 | 1.26 | -1.03 | -0.95 | 0.61 | 0.33 | -0.64 | 0.19 | -1.00 |
| P42765 | 3-ketoacyl-CoA thiolase, mitochondrial | ACAA2 | -0.27 | 0.55 | 0.65 | -0.95 | 1.99 | 0.00 | -0.98 | -0.11 | -1.33 | 0.85 | 0.30 | -0.41 |
| Q9BZ23 | Pantothenate kinase 2, mitochondrial | PANK2 | -0.27 | 0.52 | 0.84 | 1.42 | 0.66 | -1.12 | -1.14 | -0.37 | 1.04 | -0.67 | -1.17 | 0.51 |
| Q9BZV1 | UBX domain-containing protein 6 | UBXN6 | -0.27 | 0.92 | 1.19 | 0.36 | -1.20 | 0.75 | -0.02 | -0.12 | -1.60 | 1.43 | 0.11 | -0.90 |
| Q9BZH6 | WD repeat-containing protein 11 | WDR11 | -0.28 | 0.26 | 0.98 | 0.51 | -1.19 | 0.57 | -0.51 | 1.10 | 0.79 | -1.47 | -1.25 | 0.47 |
| P15954 | Cytochrome c oxidase subunit 7C, mitochondrial | COX7C | -0.28 | 0.33 | -0.23 | -0.53 | 2.17 | -0.38 | -0.58 | -0.43 | -0.22 | -0.87 | -0.44 | 1.51 |
| Q9UKG1 | DCC-interacting protein 13-alpha | APPL1 | -0.28 | 0.74 | 0.43 | 1.59 | -0.69 | 0.03 | -0.46 | 0.13 | 0.33 | -2.24 | 0.24 | 0.63 |
| Q9BXP2 | Solute carrier family 12 member 9 | SLC12A9 | -0.28 | 0.36 | -0.73 | 0.54 | -1.00 | 1.27 | 0.41 | -0.77 | -0.86 | -1.15 | 1.36 | 0.94 |
| Q9Y2A7 | Nck-associated protein 1 | NCKAP1 | -0.28 | 0.68 | -1.02 | 0.68 | -0.72 | 0.13 | 1.78 | -1.30 | -0.61 | 1.29 | -0.05 | -0.17 |
| P04632 | Calpain small subunit 1 | CAPNS1 | -0.28 | 0.63 | -0.65 | 0.76 | 1.02 | 0.53 | -0.87 | -1.52 | -1.31 | 1.27 | 0.36 | 0.40 |
| Q96RY7 | Intraflagellar transport protein 140 homolog | IFT140 | -0.28 | 0.59 | -0.06 | -1.38 | 0.80 | 0.53 | 0.86 | -1.05 | 1.23 | -1.60 | 0.44 | 0.24 |
| Q9H598 | Vesicular inhibitory amino acid transporter | SLC32A1 | -0.28 | 0.36 | 1.36 | 1.63 | -0.61 | -0.96 | -0.93 | 0.51 | 0.65 | -1.23 | -0.36 | -0.05 |
| P17987 | T-complex protein 1 subunit alpha | TCP1 | -0.28 | 2.08 | 1.35 | -0.71 | 1.71 | 0.43 | -0.80 | -0.97 | -0.59 | -0.76 | 0.87 | -0.53 |
| O95747 | Serine/threonine-protein kinase OSR1 | OXSR1 | -0.28 | 1.13 | -0.18 | 0.51 | 0.41 | -0.08 | 0.66 | -0.67 | -2.32 | -0.28 | 0.59 | 1.37 |
| Q8N999 | Uncharacterized protein C12orf29 | C12orf29 | -0.28 | 0.47 | 0.48 | -0.56 | -0.79 | 1.97 | -0.50 | -0.40 | -0.50 | -0.91 | 1.53 | -0.33 |
| P18124 | 60S ribosomal protein L7 | RPL7 | -0.28 | 0.40 | -1.11 | -1.21 | 0.68 | 0.56 | 1.62 | -1.04 | -0.89 | 0.67 | 0.77 | -0.04 |
| P23381 | Tryptophan--tRNA ligase, cytoplasmic | WARS | -0.28 | 0.26 | -1.37 | -0.31 | 0.73 | -0.03 | 1.33 | -0.62 | -0.25 | 1.63 | 0.14 | -1.26 |
| P61020 | Ras-related protein Rab-5B | RAB5B | -0.28 | 1.63 | -0.82 | -0.85 | 1.65 | 0.93 | 0.75 | -1.38 | -0.62 | -0.40 | 0.98 | -0.24 |
| P35611 | Alpha-adducin | ADD1 | -0.28 | 2.75 | 1.48 | 0.78 | 0.27 | 0.65 | -0.85 | -0.90 | 0.39 | -0.46 | 0.52 | -1.88 |
| Q7Z460 | CLIP-associating protein 1 | CLASP1 | -0.28 | 0.26 | 0.48 | 0.52 | -0.22 | 0.08 | -0.50 | 0.96 | 0.72 | -2.54 | -0.07 | 0.56 |
| Q9Y4P8 | WD repeat domain phosphoinositide-interacting protein 2 | WIPI2 | -0.28 | 0.75 | 0.92 | -0.01 | -0.70 | 1.06 | -0.35 | 0.35 | -0.11 | -2.34 | 0.82 | 0.36 |
| Q9NRN7 | L-aminoadipate-semialdehyde dehydrogenase-phosphopantetheinyl | AASDHPPT | -0.28 | 0.76 | -0.47 | 0.53 | 0.59 | 1.21 | -0.94 | 0.29 | -1.20 | -1.66 | 1.12 | 0.51 |
| Q00688 | Peptidyl-prolyl cis-trans isomerase FKBP3 | FKBP3 | -0.28 | 0.62 | 0.47 | -0.87 | 1.59 | -0.36 | -0.04 | -1.99 | 1.17 | -0.08 | 0.06 | 0.06 |
| O14828 | Secretory carrier-associated membrane protein 3 | SCAMP3 | -0.28 | 0.22 | -1.50 | 0.23 | 0.23 | 0.67 | 0.69 | -1.17 | -1.53 | 0.54 | 0.85 | 1.00 |
| P54252 | Ataxin-3 | ATXN3 | -0.28 | 0.88 | -0.69 | 1.91 | -0.09 | 0.39 | -0.48 | -1.16 | -1.47 | 0.79 | 0.51 | 0.30 |
| P51659 | Peroxisomal multifunctional enzyme type 2 | HSD17B4 | -0.28 | 0.91 | 1.68 | -0.35 | -1.06 | 0.33 | 0.47 | 0.97 | -0.94 | -1.61 | 0.47 | 0.04 |
| P61018 | Ras-related protein Rab-4B | RAB4B | -0.28 | 0.44 | -0.02 | 1.81 | -0.22 | 0.68 | -1.67 | 0.31 | -1.30 | -0.49 | 0.43 | 0.48 |
| P53355 | Death-associated protein kinase 1 | DAPK1 | -0.28 | 0.34 | -0.14 | -0.93 | -0.87 | 1.25 | 1.15 | -1.07 | -1.06 | -0.34 | 1.06 | 0.96 |
| Q02952 | A-kinase anchor protein 12 | AKAP12 | -0.29 | 0.71 | 0.01 | 2.07 | 1.00 | -0.78 | -1.40 | -0.54 | 0.32 | -0.84 | -0.06 | 0.22 |
| Q99683 | Mitogen-activated protein kinase kinase kinase 5 | MAP3K5 | -0.29 | 0.46 | -1.33 | 0.72 | 0.63 | 1.03 | -0.44 | -0.82 | -1.59 | -0.11 | 1.06 | 0.85 |
| Q9GZS3 | WD repeat-containing protein 61 | WDR61 | -0.29 | 0.59 | 0.51 | 0.78 | -0.16 | 0.56 | -0.94 | 0.64 | -0.17 | -2.40 | 0.45 | 0.72 |
| O60825 | 6-phosphofructo-2-kinase/fructose-2,6-bisphosphatase 2 | PFKFB2 | -0.29 | 0.35 | -0.92 | 1.05 | 0.05 | 0.77 | -0.47 | -1.03 | -1.32 | -0.44 | 0.63 | 1.68 |
| Q9UJV9 | Probable ATP-dependent RNA helicase DDX41 | DDX41 | -0.29 | 0.23 | 1.94 | -0.90 | -0.31 | -0.31 | -0.10 | 1.56 | -0.90 | -0.35 | 0.25 | -0.89 |
| Q9UI10 | Translation initiation factor eIF-2B subunit delta | EIF2B4 | -0.29 | 0.29 | 1.09 | 0.60 | -1.37 | 0.21 | -0.13 | 1.40 | -0.01 | -1.85 | 0.26 | -0.20 |
| Q14699 | Raftlin | RFTN1 | -0.29 | 0.29 | 0.38 | 0.41 | -0.28 | 0.56 | -0.67 | 1.43 | -0.13 | -2.36 | 0.28 | 0.37 |
| P06756 | Integrin alpha-V | ITGAV | -0.29 | 0.33 | -0.74 | 1.02 | -1.07 | 0.71 | 0.54 | -0.85 | -0.84 | -1.12 | 1.11 | 1.25 |
| P82987 | ADAMTS-like protein 3 | ADAMTSL3 | -0.29 | 0.32 | 1.32 | -0.93 | -1.11 | 0.04 | 1.12 | 0.95 | 0.83 | -1.24 | -0.87 | -0.10 |
| O15020 | Spectrin beta chain, non-erythrocytic 2 | SPTBN2 | -0.29 | 0.44 | -2.06 | 0.57 | 0.37 | 1.10 | 0.60 | -0.73 | 0.22 | -1.17 | 0.85 | 0.26 |
| Q9UN81 | LINE-1 retrotransposable element ORF1 protein | L1RE1 | -0.29 | 0.34 | -2.19 | 0.57 | 0.85 | 0.17 | 1.06 | -0.57 | 0.85 | -0.95 | 0.01 | 0.21 |
| Q9P0K8 | Forkhead box protein J2 | FOXJ2 | -0.29 | 1.12 | 0.29 | -0.35 | -0.69 | 2.59 | -0.55 | 0.00 | -0.31 | -1.02 | 0.32 | -0.28 |
| Q96BY2 | Modulator of apoptosis 1 | MOAP1 | -0.29 | 1.45 | 0.19 | -0.36 | -0.46 | 2.58 | -0.39 | -0.09 | -0.05 | -1.30 | -0.27 | 0.14 |
| Q64LD2 | WD repeat-containing protein 25 | WDR25 | -0.29 | 0.36 | 0.86 | -0.72 | 1.81 | -0.73 | -0.75 | 0.71 | -0.75 | 1.07 | -0.80 | -0.71 |
| P39687 | Acidic leucine-rich nuclear phosphoprotein 32 family member A | ANP32A | -0.29 | 0.44 | 0.86 | 0.08 | 0.85 | -0.78 | -0.43 | -1.41 | -0.97 | 1.93 | 0.13 | -0.26 |
| Q8TDZ2 | [F-actin]-monooxygenase MICAL1 | MICAL1 | -0.29 | 0.57 | -0.36 | 0.85 | -0.13 | 0.13 | 0.25 | -0.91 | -1.69 | 2.01 | 0.32 | -0.47 |
| Q16204 | Coiled-coil domain-containing protein 6 | CCDC6 | -0.29 | 0.71 | 2.20 | -1.14 | -0.26 | 0.04 | 0.04 | 0.28 | 1.00 | -0.84 | -0.37 | -0.95 |
| Q8N8R5 | UPF0565 protein C2orf69 | C2orf69 | -0.29 | 0.32 | -0.59 | -0.75 | -0.92 | 1.46 | 1.22 | -0.62 | -0.59 | -1.02 | 1.24 | 0.56 |
| Q86VN1 | Vacuolar protein-sorting-associated protein 36 | VPS36 | -0.29 | 0.39 | 0.46 | 0.13 | -1.53 | 0.08 | 1.37 | 0.75 | 0.28 | -1.93 | -0.01 | 0.39 |
| Q9NWT6 | Hypoxia-inducible factor 1-alpha inhibitor | HIF1AN | -0.29 | 0.29 | 0.52 | 0.32 | 0.69 | 0.80 | -1.92 | 0.17 | -0.76 | -1.40 | 0.83 | 0.76 |
| O14810 | Complexin-1 | CPLX1 | -0.29 | 0.47 | -0.09 | -0.35 | -0.46 | -0.48 | 1.98 | 0.08 | -0.42 | 1.38 | -1.55 | -0.09 |
| Q12959 | Disks large homolog 1 | DLG1 | -0.29 | 0.56 | -0.49 | 0.16 | 0.05 | 0.85 | 0.16 | -2.44 | -0.54 | 0.70 | 0.64 | 0.92 |
| P26378 | ELAV-like protein 4 | ELAVL4 | -0.29 | 1.05 | -0.05 | -0.06 | 0.38 | 0.50 | 0.47 | -2.18 | -0.73 | -0.04 | -0.04 | 1.76 |
| Q9NTJ5 | Phosphatidylinositide phosphatase SAC1 | SACM1L | -0.29 | 1.44 | -0.18 | 1.26 | -1.02 | 0.41 | 1.04 | 0.07 | -1.78 | -0.99 | 0.23 | 0.95 |
| P63167 | Dynein light chain 1, cytoplasmic | DYNLL1 | -0.29 | 2.09 | 1.67 | 0.25 | -0.57 | 0.95 | -0.33 | -0.74 | -0.40 | -1.85 | 0.81 | 0.21 |
| Q9NZ56 | Formin-2 | FMN2 | -0.29 | 0.39 | -0.53 | 2.05 | -1.15 | 0.89 | -0.74 | -0.97 | -0.56 | -0.08 | 0.57 | 0.52 |
| P09543 | 2',3'-cyclic-nucleotide 3'-phosphodiesterase | CNP | -0.29 | 0.38 | -0.99 | -0.91 | 1.54 | 0.57 | 0.29 | -1.37 | -0.37 | 1.15 | 0.78 | -0.69 |
| Q9Y223 | Bifunctional UDP-N-acetylglucosamine 2-epimerase/N-acetylmann | GNE | -0.30 | 0.49 | -0.17 | -0.42 | -0.67 | -0.42 | 2.31 | -0.31 | -0.45 | -0.76 | -0.45 | 1.33 |
| Q7L014 | Probable ATP-dependent RNA helicase DDX46 | DDX46 | -0.30 | 0.60 | -0.50 | 0.29 | 0.65 | 0.45 | -0.11 | -0.52 | -2.39 | 0.11 | 1.02 | 1.01 |
| Q8TE77 | Protein phosphatase Slingshot homolog 3 | SSH3 | -0.30 | 0.36 | -0.01 | 0.61 | -1.13 | 0.19 | 0.82 | 0.40 | 1.09 | -1.64 | 0.96 | -1.29 |
| Q6PUV4 | Complexin-2 | CPLX2 | -0.30 | 0.26 | -0.26 | 0.70 | -1.35 | -0.60 | 1.88 | -0.27 | -0.41 | 1.32 | -0.15 | -0.85 |
| Q86TI0 | TBC1 domain family member 1 | TBC1D1 | -0.30 | 0.35 | 1.15 | 0.51 | -1.65 | 0.80 | -0.33 | 0.53 | -1.63 | -0.17 | -0.18 | 0.98 |
| O94925 | Glutaminase kidney isoform, mitochondrial | GLS | -0.30 | 0.37 | -0.47 | -0.58 | 1.80 | -0.19 | -0.07 | -0.97 | 1.48 | -1.37 | -0.06 | 0.42 |
| P07311 | Acylphosphatase-1 | ACYP1 | -0.30 | 2.75 | 0.94 | -0.35 | 1.67 | -0.54 | 0.61 | -1.80 | -0.65 | 0.55 | 0.26 | -0.69 |
| Q9ULU8 | Calcium-dependent secretion activator 1 | CADPS | -0.30 | 0.48 | -0.20 | 1.88 | 0.26 | -0.20 | -1.11 | -0.03 | 1.25 | -1.55 | -0.31 | 0.01 |
| Q01518 | Adenylyl cyclase-associated protein 1 | CAP1 | -0.30 | 1.28 | 0.16 | 1.62 | -0.46 | 0.70 | -0.63 | 0.57 | -1.94 | -0.12 | 0.81 | -0.71 |
| O60869 | Endothelial differentiation-related factor 1 | EDF1 | -0.30 | 0.88 | 1.63 | -0.33 | 0.99 | -0.85 | -0.41 | 0.43 | 1.00 | -1.63 | -0.67 | -0.16 |
| Q9BZF3 | Oxysterol-binding protein-related protein 6 | OSBPL6 | -0.30 | 0.31 | 0.76 | 0.40 | -1.78 | 0.80 | 0.24 | 0.24 | 0.44 | -1.90 | 0.76 | 0.04 |
| Q24JP5 | Transmembrane protein 132A | TMEM132A | -0.30 | 0.52 | -1.22 | 1.21 | 0.65 | 1.12 | -1.08 | -0.41 | -0.11 | 0.07 | 1.11 | -1.33 |

| | | | | | | | | | | | | | | |
|---|---|---|---|---|---|---|---|---|---|---|---|---|---|---|
| Q2LD37 | Transmembrane protein KIAA1109 | KIAA1109 | -0.30 | 0.38 | 0.09 | 1.97 | -1.07 | 0.45 | -0.93 | 0.85 | 0.52 | -1.32 | -0.29 | -0.27 |
| Q15014 | Mortality factor 4-like protein 2 | MORF4L2 | -0.30 | 0.24 | -0.99 | 1.25 | -1.19 | 0.99 | 0.27 | -1.04 | -1.11 | -0.02 | 0.88 | 0.96 |
| P26583 | High mobility group protein B2 | HMGB2 | -0.30 | 0.48 | -1.37 | 0.14 | 1.56 | 0.77 | -0.48 | -1.32 | -1.04 | 0.55 | 0.71 | 0.48 |
| O00203 | AP-3 complex subunit beta-1 | AP3B1 | -0.30 | 0.28 | -1.74 | 0.40 | 0.24 | 0.95 | 0.53 | -1.72 | 0.36 | -0.34 | 0.18 | 1.14 |
| Q96SZ5 | 2-aminoethanethiol dioxygenase | ADO | -0.30 | 0.35 | 0.45 | 1.14 | -0.88 | 0.37 | -0.61 | -0.89 | -1.74 | 0.25 | 0.45 | 1.45 |
| O15027 | Protein transport protein Sec16A | SEC16A | -0.30 | 0.59 | 1.21 | 0.40 | -2.12 | 0.75 | 0.52 | -0.26 | 0.38 | -1.28 | -0.01 | 0.41 |
| P51116 | Fragile X mental retardation syndrome-related protein 2 | FXR2 | -0.30 | 0.43 | 1.13 | -0.53 | -1.64 | 0.78 | 0.83 | 0.00 | 0.31 | -1.71 | 0.61 | 0.21 |
| O14920 | Inhibitor of nuclear factor kappa-B kinase subunit beta | IKBKB | -0.30 | 0.41 | 0.95 | -0.54 | 0.33 | -0.61 | 0.42 | 0.07 | -1.77 | 1.87 | -0.71 | -0.01 |
| O15075 | Serine/threonine-protein kinase DCLK1 | DCLK1 | -0.30 | 0.52 | 0.56 | 0.50 | -1.23 | 0.80 | 0.06 | 0.66 | 0.93 | -2.17 | 0.31 | -0.41 |
| Q9BRT6 | Protein LLP homolog | LLPH | -0.30 | 0.31 | -0.63 | -0.71 | -0.19 | -0.10 | 2.06 | -0.69 | -0.71 | 1.53 | 0.11 | -0.67 |
| Q00610 | Clathrin heavy chain 1 | CLTC | -0.31 | 0.77 | -0.53 | 1.63 | -1.27 | 1.74 | -0.61 | -0.47 | 0.26 | -0.86 | 0.12 | 0.00 |
| Q96KP1 | Exocyst complex component 2 | EXOC2 | -0.31 | 0.49 | -1.37 | 0.65 | -1.20 | 1.21 | 1.36 | -0.74 | -0.51 | -0.38 | 1.00 | -0.01 |
| Q9Y305 | Acyl-coenzyme A thioesterase 9, mitochondrial | ACOT9 | -0.31 | 0.85 | -0.04 | 0.04 | 1.15 | -1.11 | 0.98 | -0.25 | -2.11 | 0.75 | -0.02 | 0.63 |
| Q86YR7 | Probable guanine nucleotide exchange factor MCF2L2 | MCF2L2 | -0.31 | 0.40 | 0.62 | 0.97 | 0.90 | -0.93 | -1.04 | -0.85 | -0.93 | 0.69 | 1.44 | -0.89 |
| Q8N0X4 | Citramalyl-CoA lyase, mitochondrial | CLYBL | -0.31 | 0.33 | 1.14 | -0.93 | 1.42 | -0.25 | -0.94 | 0.07 | 1.06 | -1.29 | -0.87 | 0.57 |
| Q9BSU1 | UPF0183 protein C16orf70 | C16orf70 | -0.31 | 0.22 | 0.98 | 0.66 | -0.29 | 0.02 | -1.05 | 0.95 | 1.03 | -2.11 | -0.09 | -0.09 |
| Q96I99 | Succinate--CoA ligase [GDP-forming] subunit beta, mitochondrial | SUCLG2 | -0.31 | 0.31 | 0.57 | -0.15 | 1.63 | -0.71 | -0.92 | -0.36 | 0.74 | -1.23 | -0.87 | 1.31 |
| Q15751 | Probable E3 ubiquitin-protein ligase HERC1 | HERC1 | -0.31 | 0.22 | 1.42 | -0.35 | -1.43 | 0.47 | 0.19 | 1.42 | -1.23 | -0.04 | 0.44 | -0.90 |
| O00443 | Phosphatidylinositol 4-phosphate 3-kinase C2 domain-containing s | PIK3C2A | -0.31 | 0.87 | -0.61 | 0.14 | 0.80 | 1.15 | -0.44 | -2.00 | 0.97 | -0.76 | 0.95 | -0.19 |
| Q96EV8 | Dysbindin | DTNBP1 | -0.31 | 0.72 | -0.15 | 2.29 | -0.51 | -0.40 | -0.35 | -0.26 | 1.29 | -1.04 | -0.44 | -0.44 |
| Q14168 | MAGUK p55 subfamily member 2 | MPP2 | -0.31 | 1.14 | 0.32 | 1.20 | -0.71 | 0.20 | 0.27 | -1.26 | -0.57 | -1.40 | 0.23 | 1.71 |
| Q9ULD0 | 2-oxoglutarate dehydrogenase-like, mitochondrial | OGDHL | -0.31 | 0.53 | 0.54 | -1.25 | 0.05 | 0.57 | 0.78 | -1.61 | 1.62 | -0.97 | 0.05 | 0.23 |
| O14976 | Cyclin-G-associated kinase | GAK | -0.31 | 0.52 | 1.18 | 0.93 | -2.21 | 0.41 | 0.38 | -0.51 | 0.34 | -0.99 | 0.42 | 0.05 |
| Q9Y230 | RuvB-like 2 | RUVBL2 | -0.31 | 0.26 | 1.54 | -0.97 | -1.00 | 0.15 | 0.63 | 0.70 | 1.21 | -1.17 | -0.12 | -0.97 |
| Q99418 | Cytohesin-2 | CYTH2 | -0.31 | 1.06 | -0.53 | 1.56 | -0.57 | 0.22 | 0.52 | -1.68 | -1.07 | 0.29 | 0.00 | 1.26 |
| P60866 | 40S ribosomal protein S20 | RPS20 | -0.31 | 2.53 | 1.17 | -0.51 | 0.42 | 0.28 | 0.91 | -0.40 | -2.09 | 1.00 | -0.86 | 0.09 |
| Q8IWV7 | E3 ubiquitin-protein ligase UBR1 | UBR1 | -0.31 | 0.75 | 0.20 | -0.78 | -0.64 | 0.57 | 1.56 | 1.38 | -0.08 | -0.97 | 0.26 | -1.50 |
| Q9NYF8 | Bcl-2-associated transcription factor 1 | BCLAF1 | -0.31 | 1.18 | -0.68 | -0.46 | -0.65 | 1.59 | 1.52 | 0.19 | -0.37 | -1.40 | 0.81 | -0.55 |
| Q15545 | Transcription initiation factor TFIID subunit 7 | TAF7 | -0.31 | 0.42 | -0.57 | 1.31 | -0.97 | 0.55 | 0.25 | -0.74 | -0.85 | -1.19 | 0.63 | 1.60 |
| Q9Y4B6 | DDB1- and CUL4-associated factor 1 | DCAF1 | -0.31 | 0.29 | 1.24 | 0.48 | -1.29 | 0.01 | -0.04 | 1.66 | -1.02 | -1.29 | 0.00 | 0.26 |
| P61019 | Ras-related protein Rab-2A | RAB2A | -0.31 | 1.37 | 0.59 | 1.96 | -0.72 | 0.28 | -0.64 | -1.46 | 0.93 | -0.72 | 0.26 | -0.48 |
| Q8IWW6 | Rho GTPase-activating protein 12 | ARHGAP12 | -0.31 | 0.36 | -1.11 | -0.30 | 0.12 | 0.48 | 1.29 | -0.61 | -1.92 | 1.05 | 0.75 | 0.26 |
| Q9Y6W3 | Calpain-7 | CAPN7 | -0.31 | 0.40 | 0.37 | 0.70 | -1.53 | 1.04 | -0.06 | 0.17 | -1.33 | -1.17 | 0.65 | 1.16 |
| Q16543 | Hsp90 co-chaperone Cdc37 | CDC37 | -0.32 | 2.21 | -0.11 | 2.21 | -0.91 | -0.10 | 0.99 | -0.75 | 0.57 | -0.70 | -0.91 | -0.30 |
| Q8NEZ3 | WD repeat-containing protein 19 | WDR19 | -0.32 | 0.38 | 0.72 | -1.26 | -0.15 | 0.48 | 0.71 | -0.78 | 0.15 | -1.86 | 1.18 | 0.80 |
| O60313 | Dynamin-like 120 kDa protein, mitochondrial | OPA1 | -0.32 | 1.30 | -0.64 | 1.11 | 0.82 | -1.29 | 1.41 | -0.92 | -1.17 | 0.86 | -0.28 | 0.09 |
| Q7Z6E9 | E3 ubiquitin-protein ligase RBBP6 | RBBP6 | -0.32 | 0.26 | -0.62 | 0.17 | -0.96 | 0.33 | 1.43 | -0.82 | 1.88 | -1.02 | 0.12 | -0.51 |
| P0C0L4 | Complement C4-A | C4A | -0.32 | 0.21 | -0.73 | -0.48 | 1.08 | 0.34 | 0.09 | -0.31 | -2.16 | 1.35 | 0.51 | 0.31 |
| P32418 | Sodium/calcium exchanger 1 | SLC8A1 | -0.32 | 0.67 | -1.00 | 1.03 | -0.57 | 0.64 | 0.73 | 0.22 | 0.80 | -2.10 | -0.38 | 0.64 |
| Q9NZI7 | Upstream-binding protein 1 | UBP1 | -0.32 | 0.31 | -0.65 | -0.68 | -0.81 | 1.27 | 1.31 | -0.72 | 0.57 | -1.01 | 1.37 | -0.64 |
| Q6P1M3 | LLGL scribble cell polarity complex component 2 | LLGL2 | -0.32 | 0.45 | -0.25 | 0.67 | 1.22 | -0.45 | -0.61 | -0.50 | -0.59 | -0.99 | -0.62 | 2.11 |
| Q9BXS0 | Collagen alpha-1(XXV) chain | COL25A1 | -0.32 | 0.56 | 0.58 | 0.03 | 1.41 | -1.18 | -0.12 | -1.41 | 1.29 | -0.20 | 0.67 | -1.07 |
| Q13155 | Aminoacyl tRNA synthase complex-interacting multifunctional pr | AIMP2 | -0.32 | 0.38 | 1.53 | -1.12 | -1.18 | 0.17 | 1.10 | 0.26 | 0.24 | -1.45 | 0.71 | -0.27 |
| O60506 | Heterogeneous nuclear ribonucleoprotein Q | SYNCRIP | -0.32 | 1.01 | 1.43 | -1.26 | -0.33 | -0.53 | 1.88 | -0.54 | -0.85 | 0.37 | -0.47 | 0.30 |
| Q14642 | Inositol polyphosphate-5-phosphatase A | INPP5A | -0.32 | 0.35 | -0.27 | 2.52 | -0.72 | -0.53 | -0.51 | -0.44 | -0.52 | 0.23 | -0.57 | 0.82 |
| O43169 | Cytochrome b5 type B | CYB5B | -0.32 | 0.35 | 2.20 | -0.46 | -0.61 | -0.28 | -0.38 | 1.50 | -0.39 | -0.77 | -0.43 | -0.38 |
| Q8TBC5 | Zinc finger and SCAN domain-containing protein 18 | ZSCAN18 | -0.32 | 0.47 | 1.82 | -1.10 | -0.93 | -0.18 | 1.01 | 0.69 | 0.78 | -0.65 | -0.69 | -0.75 |
| P09669 | Cytochrome c oxidase subunit 6C | COX6C | -0.32 | 0.88 | -0.45 | 0.99 | 0.89 | 0.53 | -0.91 | -0.85 | -0.87 | -1.46 | 1.20 | 0.93 |
| Q8IZL8 | Proline-, glutamic acid- and leucine-rich protein 1 | PELP1 | -0.32 | 0.45 | 1.22 | -0.77 | -1.48 | 1.24 | 0.38 | -0.49 | 0.22 | 0.23 | 0.86 | -1.41 |
| P62714 | Serine/threonine-protein phosphatase 2A catalytic subunit beta is | PPP2CB | -0.32 | 1.72 | 0.97 | 0.06 | -1.21 | 0.24 | 1.66 | 0.02 | -0.38 | -1.86 | 0.38 | 0.12 |
| P48735 | Isocitrate dehydrogenase [NADP], mitochondrial | IDH2 | -0.32 | 1.63 | -0.77 | -0.27 | 2.00 | 0.17 | 0.54 | -0.95 | -0.41 | 1.29 | -0.62 | -0.96 |
| Q5TF21 | Protein SOGA3 | SOGA3 | -0.32 | 1.33 | 1.36 | -0.96 | -0.26 | 0.47 | 0.82 | -1.42 | -1.52 | 0.54 | 0.79 | 0.18 |
| P35241 | Radixin | RDX | -0.32 | 0.54 | -1.80 | -0.02 | 0.30 | 0.31 | 1.91 | -0.48 | -1.15 | 0.35 | 0.52 | 0.06 |
| P19021 | Peptidyl-glycine alpha-amidating monooxygenase | PAM | -0.32 | 0.57 | 0.73 | 0.01 | 0.69 | -0.43 | -0.27 | 1.35 | -2.37 | 0.52 | -0.08 | -0.16 |
| Q7Z7L7 | Protein zer-1 homolog | ZER1 | -0.32 | 0.35 | 2.02 | -1.04 | 0.50 | 0.06 | -1.07 | -0.96 | -0.92 | 0.43 | 0.58 | 0.40 |
| P98194 | Calcium-transporting ATPase type 2C member 1 | ATP2C1 | -0.33 | 0.44 | -1.30 | 1.49 | -0.46 | -0.31 | 1.16 | 1.17 | -1.42 | -0.05 | -0.15 | -0.13 |
| Q9Y3I0 | tRNA-splicing ligase RtcB homolog | RTCB | -0.33 | 1.17 | -0.28 | 0.40 | -0.50 | 0.54 | 1.16 | 0.49 | -1.23 | -1.85 | 1.31 | -0.04 |
| Q9H019 | Mitochondrial fission regulator 1-like | MTFR1L | -0.33 | 0.64 | -0.55 | 0.24 | -0.25 | 0.52 | 0.86 | -1.87 | -1.01 | -0.08 | 0.45 | 1.70 |
| O14908 | PDZ domain-containing protein GIPC1 | GIPC1 | -0.33 | 0.41 | 0.13 | 0.78 | -1.00 | 0.58 | 0.07 | 0.38 | -2.34 | -0.22 | 0.71 | 0.92 |
| Q9ULT8 | E3 ubiquitin-protein ligase HECTD1 | HECTD1 | -0.33 | 0.31 | 1.52 | 0.53 | -0.71 | 0.18 | -1.10 | 0.08 | 0.89 | -1.93 | 0.19 | 0.34 |
| Q9Y2L1 | Exosome complex exonuclease RRP44 | DIS3 | -0.33 | 0.52 | -1.79 | 1.28 | -0.37 | 0.71 | 0.83 | -0.71 | -1.06 | -0.34 | 0.75 | 0.70 |
| Q13557 | Calcium/calmodulin-dependent protein kinase type II subunit delt | CAMK2D | -0.33 | 0.79 | 0.51 | 0.12 | -0.64 | 0.48 | 0.51 | -0.40 | 1.60 | -2.23 | 0.25 | -0.19 |
| O14530 | Thioredoxin domain-containing protein 9 | TXNDC9 | -0.33 | 0.47 | -0.35 | 1.32 | 0.85 | -0.56 | -0.65 | -0.49 | 1.96 | -1.01 | -0.51 | -0.56 |
| Q9NX40 | OCIA domain-containing protein 1 | OCIAD1 | -0.33 | 0.33 | 1.49 | -0.89 | -1.20 | 0.48 | 0.56 | 0.47 | -0.98 | -1.34 | 0.57 | 0.84 |
| P36222 | Chitinase-3-like protein 1 | CHI3L1 | -0.33 | 0.27 | -1.55 | 0.82 | 1.43 | -0.09 | -0.23 | -0.14 | -1.60 | 1.03 | -0.09 | 0.42 |
| P61353 | 60S ribosomal protein L27 | RPL27 | -0.33 | 0.71 | -0.48 | -0.88 | -0.43 | -0.01 | 2.71 | -0.49 | -0.14 | -0.27 | -0.23 | 0.22 |
| Q9NZZ3 | Charged multivesicular body protein 5 | CHMP5 | -0.33 | 0.40 | 0.35 | 1.31 | 0.71 | 0.40 | -2.22 | -0.99 | 0.26 | -0.53 | 0.41 | 0.31 |
| O60341 | Lysine-specific histone demethylase 1A | KDM1A | -0.33 | 0.58 | 2.33 | -0.30 | -0.66 | -0.24 | -0.39 | 1.27 | -0.43 | -0.89 | -0.46 | -0.22 |
| Q06547 | GA-binding protein subunit beta-1 | GABPB1 | -0.33 | 0.52 | 1.83 | -0.58 | -0.79 | 0.96 | -0.75 | 1.05 | -0.64 | -1.06 | 0.52 | -0.54 |
| Q9BSD7 | Cancer-related nucleoside-triphosphatase | NTPCR | -0.33 | 0.64 | 0.76 | 0.20 | 0.64 | 0.27 | -1.06 | 0.98 | -2.37 | 0.09 | 0.43 | 0.06 |
| P48553 | Trafficking protein particle complex subunit 10 | TRAPPC10 | -0.33 | 0.48 | 0.93 | -0.02 | 0.06 | -0.28 | -0.06 | 0.91 | 1.06 | -2.43 | -0.37 | 0.20 |
| P07437 | Tubulin beta chain | TUBB | -0.33 | 1.10 | 0.31 | 1.92 | -0.40 | 0.24 | -0.83 | 0.87 | -0.85 | -1.66 | 0.14 | 0.27 |
| P22626 | Heterogeneous nuclear ribonucleoproteins A2/B1 | HNRNPA2B1 | -0.33 | 0.67 | 1.72 | -0.73 | -1.36 | 0.65 | 0.56 | 0.57 | -1.11 | -1.03 | 0.46 | 0.29 |
| P62745 | Rho-related GTP-binding protein RhoB | RHOB | -0.33 | 0.42 | -0.91 | 0.92 | -0.33 | 0.70 | 0.18 | -1.64 | -1.32 | 0.48 | 0.62 | 1.29 |
| P02545 | Prelamin-A/C | LMNA | -0.33 | 0.30 | -1.65 | 0.66 | -0.58 | 0.91 | 1.07 | -0.99 | -1.21 | 0.61 | 0.66 | 0.53 |
| Q8TB45 | DEP domain-containing mTOR-interacting protein | DEPTOR | -0.33 | 0.33 | 0.80 | -0.83 | -1.00 | 1.11 | 0.36 | -0.78 | 1.47 | -1.13 | 0.78 | -0.80 |
| P30419 | Glycylpeptide N-tetradecanoyltransferase 1 | NMT1 | -0.33 | 1.34 | 0.58 | 1.65 | 0.26 | -0.33 | -0.72 | 0.35 | 0.60 | -2.17 | -0.26 | 0.04 |
| Q5T447 | E3 ubiquitin-protein ligase HECTD3 | HECTD3 | -0.34 | 0.42 | 0.44 | 0.78 | 0.45 | 0.70 | -1.81 | 0.41 | 0.36 | -0.33 | 0.79 | -1.79 |
| Q9Y6Q5 | AP-1 complex subunit mu-2 | AP1M2 | -0.34 | 0.27 | 0.02 | 0.61 | 0.75 | 0.46 | -1.46 | -1.36 | -1.40 | 0.73 | 0.86 | 0.80 |
| Q9Y2H2 | Phosphatidylinositide phosphatase SAC2 | INPP5F | -0.34 | 0.42 | -0.50 | 0.75 | -0.95 | 1.42 | -0.18 | -0.80 | -0.80 | -1.18 | 1.34 | 0.89 |
| Q9UNA4 | DNA polymerase iota | POLI | -0.34 | 0.37 | 1.80 | -0.45 | -1.20 | 0.57 | -0.22 | 0.55 | 0.71 | -1.45 | 0.48 | -0.78 |
| Q92932 | Receptor-type tyrosine-protein phosphatase N2 | PTPRN2 | -0.34 | 0.43 | -0.22 | 1.20 | -0.37 | -0.23 | 0.21 | -1.93 | 1.66 | 0.61 | -0.38 | -0.54 |
| P14866 | Heterogeneous nuclear ribonucleoprotein L | HNRNPL | -0.34 | 1.74 | -0.33 | 0.98 | -0.01 | 0.66 | 0.48 | -0.67 | -2.14 | 1.12 | 0.64 | -0.74 |

| | | | | | | | | | | | | | | |
|---|---|---|---|---|---|---|---|---|---|---|---|---|---|---|
| P02751 | Fibronectin | FN1 | -0.34 | 0.48 | -1.54 | 0.55 | 0.07 | 0.45 | 1.10 | -1.87 | -0.55 | 0.66 | 0.40 | 0.73 |
| Q96JJ6 | Junctophilin-4 | JPH4 | -0.34 | 0.35 | 0.26 | 1.22 | -1.34 | -1.07 | 1.41 | 0.16 | 0.08 | -1.46 | 0.55 | 0.18 |
| P20393 | Nuclear receptor subfamily 1 group D member 1 | NR1D1 | -0.34 | 3.31 | 0.46 | -0.07 | 0.19 | -0.22 | 2.25 | -0.07 | -0.50 | -1.76 | -0.47 | 0.19 |
| Q5BKY9 | Protein FAM133B | FAM133B | -0.34 | 0.41 | -0.05 | -1.54 | 0.25 | 0.89 | 0.99 | -1.66 | -0.84 | 0.49 | 0.80 | 0.67 |
| P82909 | 28S ribosomal protein S36, mitochondrial | MRPS36 | -0.34 | 0.41 | -1.47 | 0.87 | 0.71 | 1.06 | -0.63 | -1.51 | -0.17 | -0.53 | 0.61 | 1.06 |
| P43004 | Excitatory amino acid transporter 2 | SLC1A2 | -0.34 | 1.19 | 0.31 | 1.93 | -1.46 | 0.07 | 0.47 | -0.97 | 0.36 | -1.18 | -0.14 | 0.60 |
| P78324 | Tyrosine-protein phosphatase non-receptor type substrate 1 | SIRPA | -0.34 | 0.43 | -0.83 | 1.82 | 0.31 | 0.21 | -0.94 | -0.34 | -0.14 | 1.57 | -0.78 | -0.89 |
| Q9UEW8 | STE20/SPS1-related proline-alanine-rich protein kinase | STK39 | -0.34 | 0.71 | 0.85 | 0.58 | -2.09 | 1.19 | 0.36 | -0.59 | -0.75 | -0.68 | 0.58 | 0.56 |
| P35998 | 26S proteasome regulatory subunit 7 | PSMC2 | -0.34 | 0.35 | -0.73 | 1.72 | 0.02 | 0.47 | -1.00 | 0.81 | -1.69 | -0.58 | 0.51 | 0.47 |
| Q8NF37 | Lysophosphatidylcholine acyltransferase 1 | LPCAT1 | -0.34 | 0.38 | 0.02 | -0.24 | -0.32 | -0.74 | 1.78 | 1.84 | -0.37 | -0.70 | -1.03 | -0.23 |
| Q8N6M0 | Deubiquitinase OTUD6B | OTUD6B | -0.34 | 0.90 | 0.58 | 1.19 | -1.62 | 1.09 | -0.18 | -0.41 | -1.19 | -0.66 | 1.19 | 0.01 |
| P48730 | Casein kinase I isoform delta | CSNK1D | -0.34 | 0.49 | -0.55 | 1.23 | -1.36 | 0.67 | 0.65 | -0.21 | -1.90 | 0.71 | 0.39 | 0.38 |
| Q8N612 | FTS and Hook-interacting protein | FAM160A2 | -0.34 | 0.43 | 1.49 | -0.99 | 0.46 | 0.71 | -1.10 | -0.96 | 1.14 | -0.55 | 0.72 | -0.91 |
| Q8IY67 | Ribonucleoprotein PTB-binding 1 | RAVER1 | -0.34 | 0.35 | 1.48 | -0.34 | -1.62 | -0.01 | 0.96 | 0.62 | 0.24 | -1.61 | 0.26 | 0.02 |
| Q5VTB9 | E3 ubiquitin-protein ligase RNF220 | RNF220 | -0.34 | 0.33 | 1.13 | 0.16 | -1.79 | 0.32 | 0.63 | 0.20 | -1.62 | 0.96 | 0.46 | -0.45 |
| O00139 | Kinesin-like protein KIF2A | KIF2A | -0.34 | 0.50 | -0.26 | 1.84 | -0.33 | 0.07 | -0.67 | -0.01 | 1.07 | -1.94 | 0.02 | 0.21 |
| Q9NRH2 | SNF-related serine/threonine-protein kinase | SNRK | -0.34 | 0.45 | -0.31 | 1.08 | -1.37 | 0.38 | 0.81 | -1.15 | 1.53 | 0.39 | -0.30 | -1.07 |
| O60268 | Uncharacterized protein KIAA0513 | KIAA0513 | -0.34 | 0.53 | -0.19 | 1.87 | 0.19 | -0.26 | -0.94 | -0.32 | 1.32 | -1.57 | -0.36 | 0.25 |
| Q15382 | GTP-binding protein Rheb | RHEB | -0.35 | 1.07 | 1.13 | 0.34 | -1.39 | 0.42 | 0.72 | 0.27 | 0.33 | -2.17 | 0.34 | 0.01 |
| Q9H4X1 | Regulator of cell cycle RGCC | RGCC | -0.35 | 0.36 | -0.29 | 2.45 | -0.70 | -0.44 | -0.54 | 0.54 | 0.74 | -0.77 | -0.47 | -0.52 |
| Q9NY65 | Tubulin alpha-8 chain | TUBA8 | -0.35 | 0.85 | 1.51 | 0.28 | -1.60 | 0.83 | 0.00 | -0.33 | 0.08 | -1.69 | 0.43 | 0.50 |
| P14324 | Farnesyl pyrophosphate synthase | FDPS | -0.35 | 0.52 | -0.20 | -0.22 | 1.45 | 0.07 | -0.42 | -1.27 | -1.13 | 1.92 | 0.01 | -0.20 |
| O14545 | TRAF-type zinc finger domain-containing protein 1 | TRAFD1 | -0.35 | 0.54 | -0.10 | 1.40 | 0.01 | -0.02 | -0.58 | 0.67 | -2.28 | -0.12 | 0.02 | 1.02 |
| Q9Y4E5 | E3 SUMO-protein ligase ZNF451 | ZNF451 | -0.35 | 0.68 | 1.14 | 0.70 | -0.66 | -0.92 | 0.58 | -1.80 | -0.26 | -0.60 | 0.53 | 1.28 |
| Q9UNN5 | FAS-associated factor 1 | FAF1 | -0.35 | 0.90 | 0.86 | 1.25 | 0.17 | 0.42 | -1.64 | 0.98 | -1.09 | -0.75 | 0.63 | -0.84 |
| Q5TCQ9 | Membrane-associated guanylate kinase, WW and PDZ domain-con | MAGI3 | -0.35 | 0.61 | -0.21 | 2.39 | -1.06 | -0.06 | -0.29 | 0.42 | -0.85 | 0.49 | -0.95 | 0.11 |
| Q6BDS2 | UHRF1-binding protein 1 | UHRF1BP1 | -0.35 | 0.36 | 0.33 | 1.53 | -1.52 | 0.53 | -0.38 | 0.92 | 0.32 | -1.67 | 0.10 | -0.16 |
| Q8TEQ0 | Sorting nexin-29 | SNX29 | -0.35 | 0.56 | -0.38 | 0.98 | -0.98 | 1.72 | -0.63 | -0.55 | -0.58 | -1.04 | 1.36 | 0.11 |
| Q8N1G4 | Leucine-rich repeat-containing protein 47 | LRRC47 | -0.35 | 0.82 | -0.42 | 1.17 | -0.40 | 1.15 | -0.51 | -1.30 | 0.07 | -1.49 | 1.25 | 0.48 |
| Q13595 | Transformer-2 protein homolog alpha | TRA2A | -0.35 | 0.43 | 0.07 | -0.79 | 1.01 | -0.59 | 0.89 | 0.45 | -2.07 | -0.59 | 0.87 | 0.78 |
| Q16836 | Hydroxyacyl-coenzyme A dehydrogenase, mitochondrial | HADH | -0.35 | 0.64 | -1.01 | -0.51 | 1.88 | 0.47 | -0.04 | -1.71 | -0.09 | 0.56 | 0.78 | -0.34 |
| Q9HAU5 | Regulator of nonsense transcripts 2 | UPF2 | -0.35 | 0.45 | -0.37 | 0.44 | 1.57 | 0.05 | -1.10 | 0.48 | 1.02 | -1.93 | -0.14 | -0.03 |
| Q3KQU3 | MAP7 domain-containing protein 1 | MAP7D1 | -0.35 | 2.16 | 1.42 | -0.12 | 0.58 | 0.31 | -0.14 | 0.56 | 0.62 | -2.33 | -0.43 | -0.47 |
| P47870 | Gamma-aminobutyric acid receptor subunit beta-2 | GABRB2 | -0.35 | 0.72 | 1.31 | -1.18 | -0.26 | 0.72 | 0.29 | 0.85 | 0.52 | -2.02 | 0.13 | -0.37 |
| Q14738 | Serine/threonine-protein phosphatase 2A 56 kDa regulatory subuni | PPP2R5D | -0.35 | 0.72 | 1.54 | 0.64 | -1.40 | 0.22 | -0.11 | 0.44 | 0.33 | -1.96 | 0.08 | 0.23 |
| P54278 | Mismatch repair endonuclease PMS2 | PMS2 | -0.35 | 0.54 | 0.62 | -0.95 | -1.21 | 1.38 | 0.84 | 0.55 | 0.16 | -1.36 | 0.83 | -0.87 |
| P62277 | 40S ribosomal protein S13 | RPS13 | -0.35 | 1.27 | 0.13 | -0.23 | 0.66 | 0.48 | 0.41 | -1.87 | -0.64 | 1.95 | -0.34 | -0.54 |
| Q969T9 | WW domain-binding protein 2 | WBP2 | -0.35 | 0.32 | -0.85 | -0.84 | 0.41 | 0.36 | 1.36 | -0.93 | -0.85 | 1.71 | 0.46 | -0.83 |
| Q9Y6E0 | Serine/threonine-protein kinase 24 | STK24 | -0.35 | 0.56 | 0.26 | 0.71 | -0.37 | 0.62 | -0.49 | 0.48 | 0.57 | -2.58 | 0.13 | 0.67 |
| Q5TGY3 | AT-hook DNA-binding motif-containing protein 1 | AHDC1 | -0.35 | 1.55 | 2.61 | -0.35 | -0.50 | -0.01 | -0.10 | -0.27 | -0.34 | -1.26 | 0.02 | 0.21 |
| Q96FJ0 | AMSH-like protease | STAMBPL1 | -0.35 | 0.71 | -0.44 | 0.72 | 0.60 | 0.26 | -0.24 | -0.23 | 1.63 | 0.30 | -0.39 | -2.20 |
| P26440 | Isovaleryl-CoA dehydrogenase, mitochondrial | IVD | -0.35 | 0.66 | -1.34 | 1.13 | 0.29 | 0.56 | 0.18 | -1.63 | -0.75 | 1.40 | 0.45 | -0.30 |
| Q14982 | Opioid-binding protein/cell adhesion molecule | OPCML | -0.36 | 0.27 | 0.19 | 1.35 | -1.59 | 0.29 | 0.12 | -1.75 | 0.11 | 1.17 | -0.13 | 0.24 |
| Q9UQ35 | Serine/arginine repetitive matrix protein 2 | SRRM2 | -0.36 | 1.60 | 0.79 | -0.14 | 0.66 | 0.40 | -0.02 | -2.33 | -1.11 | 0.34 | 0.83 | 0.57 |
| P00751 | Complement factor B | CFB | -0.36 | 0.42 | -1.47 | 1.01 | 0.45 | 0.87 | -0.30 | -0.55 | -1.80 | 0.25 | 0.79 | 0.75 |
| P46940 | Ras GTPase-activating-like protein IQGAP1 | IQGAP1 | -0.36 | 0.53 | 0.80 | 0.21 | 0.36 | 0.21 | -0.89 | -0.08 | -2.48 | 0.53 | 0.63 | 0.71 |
| Q9HCH3 | Copine-5 | CPNE5 | -0.36 | 0.74 | 0.53 | 0.29 | 0.24 | 0.32 | -0.45 | -2.04 | 1.44 | -0.37 | 0.99 | -0.96 |
| O15530 | 3-phosphoinositide-dependent protein kinase 1 | PDPK1 | -0.36 | 0.94 | -0.84 | 1.52 | 0.00 | 0.71 | -0.29 | -1.03 | 0.11 | -1.75 | 0.67 | 0.90 |
| Q86T03 | Type 1 phosphatidylinositol 4,5-bisphosphate 4-phosphatase | PIP4P1 | -0.36 | 0.36 | -1.52 | 1.47 | 0.60 | 0.42 | -0.50 | -1.42 | -0.79 | 0.37 | 0.81 | 0.55 |
| P29144 | Tripeptidyl-peptidase 2 | TPP2 | -0.36 | 0.62 | -1.22 | 0.99 | -0.09 | 0.82 | 0.28 | -1.33 | -1.58 | 0.67 | 0.68 | 0.77 |
| Q9C0D3 | Protein zyg-11 homolog B | ZYG11B | -0.36 | 0.40 | -0.74 | 0.55 | -1.09 | 1.14 | 0.68 | -0.80 | -0.81 | -1.13 | 0.79 | 1.42 |
| Q02750 | Dual specificity mitogen-activated protein kinase kinase 1 | MAP2K1 | -0.36 | 0.43 | 1.10 | 0.44 | 0.02 | 0.07 | -1.06 | 0.40 | 1.31 | -2.14 | 0.02 | -0.16 |
| Q13123 | Protein Red | IK | -0.36 | 0.48 | -0.94 | 0.72 | -0.89 | 0.78 | 0.96 | -0.63 | -1.64 | -0.37 | 1.08 | 0.93 |
| P62241 | 40S ribosomal protein S8 | RPS8 | -0.36 | 0.85 | -1.42 | -0.70 | 0.37 | 0.72 | 2.07 | -0.65 | -0.86 | -0.17 | 0.57 | 0.08 |
| Q8ND04 | Protein SMG8 | SMG8 | -0.36 | 0.45 | 1.36 | 0.49 | -1.01 | 0.71 | -0.95 | 0.22 | 1.51 | -1.19 | -0.82 | -0.31 |
| Q10570 | Cleavage and polyadenylation specificity factor subunit 1 | CPSF1 | -0.36 | 0.51 | 0.67 | -1.59 | -0.77 | 1.28 | 1.06 | -1.04 | 0.33 | -0.85 | 0.86 | 0.04 |
| P02462 | Collagen alpha-1(IV) chain | COL4A1 | -0.36 | 0.28 | -0.59 | -0.73 | 2.24 | -0.05 | -0.47 | -0.71 | 0.38 | -0.89 | -0.34 | 1.16 |
| O95685 | Protein phosphatase 1 regulatory subunit 3D | PPP1R3D | -0.36 | 1.90 | -1.01 | 0.91 | 1.56 | 0.20 | 0.20 | -0.35 | -1.95 | 0.71 | -0.41 | 0.17 |
| A6NIH7 | Protein unc-119 homolog B | UNC119B | -0.36 | 0.34 | 0.41 | 0.42 | -1.37 | 0.81 | 0.20 | 0.38 | -1.27 | -1.53 | 0.81 | 1.14 |
| P35579 | Myosin-9 | MYH9 | -0.36 | 0.60 | 0.39 | -0.90 | 0.19 | 0.45 | 0.63 | -0.22 | -2.38 | -0.02 | 0.93 | 0.92 |
| Q9HCD5 | Nuclear receptor coactivator 5 | NCOA5 | -0.36 | 0.42 | 2.30 | -0.37 | -0.54 | -0.34 | -0.50 | 1.37 | -0.34 | -0.74 | -0.41 | -0.44 |
| Q9BZQ8 | Protein Niban 1 | NIBAN1 | -0.36 | 0.37 | 0.91 | 1.54 | -0.39 | -0.57 | -0.99 | 0.26 | -0.86 | -1.18 | -0.17 | 1.45 |
| Q9BYB0 | SH3 and multiple ankyrin repeat domains protein 3 | SHANK3 | -0.37 | 0.48 | 1.39 | -0.31 | 1.03 | -0.46 | -1.02 | 0.98 | 1.02 | -0.63 | -0.63 | -1.37 |
| Q63HN8 | E3 ubiquitin-protein ligase RNF213 | RNF213 | -0.37 | 0.47 | -0.75 | -0.32 | -0.60 | 0.45 | 1.82 | -1.75 | -0.55 | 0.55 | 0.32 | 0.82 |
| P13645 | Keratin, type I cytoskeletal 10 | KRT10 | -0.37 | 0.79 | 0.79 | -1.46 | 1.36 | -1.08 | 1.36 | -0.26 | 0.26 | 0.47 | -0.78 | -0.65 |
| Q15126 | Phosphomevalonate kinase | PMVK | -0.37 | 0.62 | 0.99 | -0.64 | -0.82 | 0.88 | 0.36 | 0.83 | -1.01 | -1.86 | 0.84 | 0.41 |
| Q9HC56 | Protocadherin-9 | PCDH9 | -0.37 | 0.35 | 1.34 | 0.91 | -1.32 | 0.71 | -1.18 | 0.49 | 0.44 | -1.44 | -0.32 | 0.36 |
| P37108 | Signal recognition particle 14 kDa protein | SRP14 | -0.37 | 1.01 | -0.35 | -0.53 | 2.51 | 0.20 | -0.64 | -1.07 | -0.15 | -0.65 | 0.28 | 0.43 |
| Q9H0F7 | ADP-ribosylation factor-like protein 6 | ARL6 | -0.37 | 0.38 | 1.29 | 0.49 | -1.29 | 1.11 | -1.09 | 0.55 | -0.24 | -1.41 | 0.79 | -0.21 |
| Q96SU4 | Oxysterol-binding protein-related protein 9 | OSBPL9 | -0.37 | 0.42 | 1.20 | -0.33 | 1.05 | -0.28 | -1.09 | 1.14 | -1.13 | -1.45 | 0.54 | 0.34 |
| P61923 | Coatomer subunit zeta-1 | COPZ1 | -0.37 | 0.48 | 0.95 | 0.32 | -0.70 | 0.70 | -0.65 | 0.21 | 0.36 | -2.12 | -0.40 | 1.32 |
| Q02246 | Contactin-2 | CNTN2 | -0.37 | 1.22 | -0.06 | 0.07 | -0.60 | 0.58 | 1.37 | -1.86 | -0.89 | 1.18 | 0.72 | -0.50 |
| O75663 | TIP41-like protein | TIPRL | -0.37 | 1.38 | -0.73 | 0.26 | 1.67 | 0.43 | -0.16 | -1.34 | -1.56 | 1.01 | 0.40 | 0.01 |
| O00116 | Alkyldihydroxyacetonephosphate synthase, peroxisomal | AGPS | -0.37 | 0.45 | 0.90 | -1.49 | 0.86 | -0.76 | 1.08 | -0.10 | -1.19 | 1.32 | -0.57 | -0.04 |
| Q15008 | 26S proteasome non-ATPase regulatory subunit 6 | PSMD6 | -0.37 | 1.12 | -0.67 | -1.15 | 0.33 | 1.71 | 1.03 | -0.93 | -1.06 | -0.44 | 0.93 | 0.25 |
| Q15831 | Serine/threonine-protein kinase STK11 | STK11 | -0.37 | 0.53 | -0.06 | 1.12 | -0.82 | 0.35 | 0.10 | -0.66 | 1.97 | -1.58 | -0.04 | -0.37 |
| Q9Y2I1 | Nischarin | NISCH | -0.37 | 0.28 | 1.32 | -0.11 | -1.36 | 0.28 | 0.27 | 0.51 | 1.03 | -1.96 | 0.22 | -0.19 |
| Q9NP97 | Dynein light chain roadblock-type 1 | DYNLRB1 | -0.37 | 4.04 | 1.39 | 1.52 | -0.27 | -0.37 | 0.63 | -0.70 | -1.05 | -0.75 | -1.18 | 0.78 |
| Q9UF56 | F-box/LRR-repeat protein 17 | FBXL17 | -0.37 | 0.55 | 1.37 | -0.84 | -0.97 | 0.42 | 0.72 | 0.77 | 1.24 | -1.27 | -0.70 | -0.74 |
| Q9BUJ2 | Heterogeneous nuclear ribonucleoprotein U-like protein 1 | HNRNPUL1 | -0.37 | 0.58 | 1.77 | -0.76 | -1.53 | 0.48 | 0.78 | 0.47 | -0.34 | -0.99 | 0.68 | -0.57 |
| P07237 | Protein disulfide-isomerase | P4HB | -0.37 | 0.44 | -0.05 | 0.89 | 0.87 | -0.21 | -0.91 | -0.77 | -0.77 | 2.06 | -1.07 | -0.04 |
| P82979 | SAP domain-containing ribonucleoprotein | SARNP | -0.37 | 0.52 | -0.70 | -0.48 | 1.37 | 0.22 | 0.27 | -1.26 | -1.10 | 1.67 | 0.59 | -0.58 |
| Q08209 | Serine/threonine-protein phosphatase 2B catalytic subunit alpha i | PPP3CA | -0.37 | 0.78 | 0.32 | 0.10 | -0.55 | 0.24 | 0.85 | -1.55 | -1.20 | 1.94 | 0.23 | -0.39 |

| | | | | | | | | | | | | | | |
|---|---|---|---|---|---|---|---|---|---|---|---|---|---|---|
| P14543 | Nidogen-1 | NID1 | -0.37 | 0.52 | -0.48 | -0.12 | 1.02 | 0.54 | -0.28 | -0.04 | -2.41 | 0.02 | 0.98 | 0.77 |
| Q9H008 | Phospholysine phosphohistidine inorganic pyrophosphate phosphata | LHPP | -0.37 | 0.23 | -1.39 | 0.22 | 0.61 | 0.86 | 0.02 | -1.23 | -1.48 | 1.19 | 0.76 | 0.44 |
| Q9Y2L5 | Trafficking protein particle complex subunit 8 | TRAPPC8 | -0.37 | 0.53 | -1.26 | -0.07 | -0.25 | 0.31 | 1.95 | -1.42 | -0.72 | 0.83 | 0.37 | 0.26 |
| Q14689 | Disco-interacting protein 2 homolog A | DIP2A | -0.37 | 0.82 | -1.63 | 1.81 | 0.56 | 0.39 | -0.14 | -0.41 | -1.06 | 0.57 | 0.65 | -0.73 |
| Q9HCE6 | Rho guanine nucleotide exchange factor 10-like protein | ARHGEF10L | -0.38 | 0.49 | 0.40 | 0.23 | 0.40 | 0.30 | -0.69 | 0.38 | 0.15 | -2.57 | 0.32 | 1.08 |
| P38117 | Electron transfer flavoprotein subunit beta | ETFB | -0.38 | 1.00 | -0.52 | -0.38 | 0.34 | 0.28 | 1.44 | -2.13 | -0.78 | 0.82 | 0.63 | 0.31 |
| P14314 | Glucosidase 2 subunit beta | PRKCSH | -0.38 | 0.39 | -0.88 | 0.90 | 1.04 | -0.50 | -0.03 | -0.99 | -1.01 | 1.19 | -1.00 | 1.29 |
| Q9HDC5 | Junctophilin-1 | JPH1 | -0.38 | 0.33 | 1.14 | 0.94 | -0.88 | -0.80 | 0.05 | 1.43 | 0.88 | -1.12 | -0.82 | -0.83 |
| P27708 | CAD protein | CAD | -0.38 | 0.45 | 0.00 | 0.07 | 0.28 | -0.41 | 0.66 | -0.93 | -1.80 | 1.89 | -0.45 | 0.68 |
| Q15696 | U2 small nuclear ribonucleoprotein auxiliary factor 35 kDa subuni | ZRSR2 | -0.38 | 0.40 | -0.26 | 2.18 | -0.61 | -0.36 | -0.42 | -0.50 | -0.37 | -0.82 | -0.37 | 1.52 |
| Q9H7B4 | Histone-lysine N-methyltransferase SMYD3 | SMYD3 | -0.38 | 0.59 | 0.63 | -1.98 | 0.52 | 1.07 | 0.52 | -0.76 | -0.66 | 0.28 | 1.15 | -0.76 |
| P23634 | Plasma membrane calcium-transporting ATPase 4 | ATP2B4 | -0.38 | 0.76 | -0.83 | 1.32 | 1.52 | -0.32 | -0.77 | -1.49 | 0.95 | -0.44 | -0.35 | 0.40 |
| Q9H3H3 | UPF0696 protein C11orf68 | C11orf68 | -0.38 | 0.81 | 0.79 | 1.41 | -0.85 | 0.71 | -1.10 | -1.20 | -0.63 | 1.34 | 0.05 | -0.53 |
| O43741 | 5'-AMP-activated protein kinase subunit beta-2 | PRKAB2 | -0.38 | 0.29 | -1.43 | 0.85 | -0.38 | 0.70 | 0.66 | -1.15 | 0.63 | -1.41 | 1.12 | 0.42 |
| Q8N1F8 | Serine/threonine-protein kinase 11-interacting protein | STK11IP | -0.38 | 0.51 | 0.41 | 0.80 | -1.74 | 0.71 | 0.48 | -1.33 | -0.67 | -0.52 | 0.60 | 1.26 |
| Q9Y5S9 | RNA-binding protein 8A | RBM8A | -0.38 | 1.39 | 1.50 | -0.12 | 0.04 | 0.23 | -0.16 | -0.37 | -2.36 | -0.07 | 0.84 | 0.47 |
| Q7Z4L5 | Tetratricopeptide repeat protein 21B | TTC21B | -0.38 | 0.51 | 0.99 | -0.82 | -1.01 | 1.07 | 0.42 | -0.82 | -0.80 | -1.18 | 0.94 | 1.20 |
| Q9NVA2 | Septin-11 | SEPTIN11 | -0.38 | 1.11 | 1.04 | 1.05 | -1.58 | -0.59 | 1.34 | -0.59 | 0.85 | -0.24 | -0.98 | -0.31 |
| Q9Y5X3 | Sorting nexin-5 | SNX5 | -0.38 | 0.47 | -1.86 | 1.62 | 0.58 | 0.29 | -0.02 | -0.57 | -0.84 | -0.44 | 0.12 | 1.12 |
| Q9BQ87 | F-box-like/WD repeat-containing protein TBL1Y | TBL1Y | -0.38 | 1.32 | 2.65 | -0.08 | -0.48 | -0.19 | -0.44 | 0.08 | -0.31 | -1.19 | -0.01 | -0.04 |
| P42262 | Glutamate receptor 2 | GRIA2 | -0.38 | 1.22 | -0.78 | 0.93 | 0.44 | 1.04 | -0.28 | -2.14 | -0.31 | -0.40 | 1.07 | 0.44 |
| Q14103 | Heterogeneous nuclear ribonucleoprotein D0 | HNRNPD | -0.38 | 1.35 | 1.29 | 0.37 | -0.64 | 0.94 | -0.51 | 0.13 | -1.99 | -0.61 | 1.12 | -0.10 |
| Q96JP5 | E3 ubiquitin-protein ligase ZFP91 | ZFP91 | -0.38 | 0.53 | -0.89 | -0.20 | 0.86 | 0.33 | 0.58 | -1.27 | -1.24 | 1.91 | 0.27 | -0.36 |
| Q9UPU5 | Ubiquitin carboxyl-terminal hydrolase 24 | USP24 | -0.38 | 0.77 | -1.33 | 1.62 | 0.44 | 0.57 | -0.36 | -1.64 | -0.29 | -0.40 | 0.48 | 0.90 |
| Q5QNW6 | Histone H2B type 2-F | HIST2H2BF | -0.38 | 0.54 | 0.56 | -1.40 | 2.19 | -0.26 | -0.39 | 0.42 | -1.18 | -0.14 | -0.07 | 0.27 |
| Q8NDT2 | Putative RNA-binding protein 15B | RBM15B | -0.38 | 0.53 | 1.66 | -0.65 | -0.76 | -0.56 | 0.99 | 1.51 | -0.67 | -0.97 | -0.01 | -0.54 |
| Q05D32 | CTD small phosphatase-like protein 2 | CTDSPL2 | -0.38 | 0.33 | 0.67 | -1.22 | -0.26 | 0.70 | 0.55 | 1.48 | 0.35 | -1.51 | 0.42 | -1.20 |
| Q9H089 | Large subunit GTPase 1 homolog | LSG1 | -0.38 | 0.29 | 0.60 | 0.90 | -1.31 | 0.49 | -0.27 | 0.26 | -1.24 | -1.46 | 0.98 | 1.06 |
| Q15599 | Na(+)/H(+) exchange regulatory cofactor NHE-RF2 | SLC9A3R2 | -0.38 | 1.04 | -1.43 | 0.55 | 0.26 | 0.59 | 1.21 | -1.13 | -1.40 | 1.17 | 0.38 | -0.21 |
| O00255 | Menin | MEN1 | -0.39 | 0.49 | 0.57 | 0.95 | -1.75 | 1.25 | -0.38 | 0.51 | -1.43 | -0.41 | 0.72 | -0.04 |
| Q8IUK8 | Cerebellin-2 | CBLN2 | -0.39 | 0.42 | 1.00 | -0.61 | -0.73 | -0.53 | 1.43 | -0.55 | -0.67 | 1.83 | -0.62 | -0.54 |
| Q8IZQ1 | WD repeat and FYVE domain-containing protein 3 | WDFY3 | -0.39 | 0.44 | -1.37 | 1.00 | 0.08 | 0.24 | 0.62 | -1.89 | -0.23 | 0.02 | 0.09 | 1.44 |
| Q9H244 | P2Y purinoceptor 12 | P2RY12 | -0.39 | 0.45 | -1.69 | 0.68 | 0.24 | 0.51 | 0.84 | -0.68 | -1.62 | 1.09 | 0.63 | 0.01 |
| O95278 | Laforin | EPM2A | -0.39 | 0.72 | 0.63 | 0.24 | -1.78 | 0.92 | 0.88 | 0.15 | -0.97 | -1.37 | 0.52 | 0.79 |
| P61601 | Neurocalcin-delta | NCALD | -0.39 | 0.39 | -0.81 | -0.05 | -1.08 | 0.82 | 1.65 | -0.92 | -0.86 | 1.36 | 0.36 | -0.45 |
| P05387 | 60S acidic ribosomal protein P2 | RPLP2 | -0.39 | 0.36 | -0.69 | 0.00 | -0.38 | -0.27 | 1.82 | -1.78 | -0.41 | 1.25 | 0.24 | 0.20 |
| Q7Z5Q1 | Cytoplasmic polyadenylation element-binding protein 2 | CPEB2 | -0.39 | 0.53 | -0.63 | -1.03 | 0.65 | 1.09 | 0.60 | -1.04 | -1.69 | 0.52 | 1.07 | 0.47 |
| O75884 | Serine hydrolase RBBP9 | RBBP9 | -0.39 | 0.45 | -0.91 | 1.10 | 0.37 | 0.16 | -0.11 | -0.17 | -2.15 | -0.20 | 0.60 | 1.32 |
| Q8IYB8 | ATP-dependent RNA helicase SUPV3L1, mitochondrial | SUPV3L1 | -0.39 | 0.43 | -0.71 | -0.96 | 0.95 | 0.95 | 0.35 | -0.90 | -0.89 | -1.13 | 0.99 | 1.35 |
| Q9BRX8 | Peroxiredoxin-like 2A | PRXL2A | -0.39 | 2.04 | 2.65 | -0.01 | -0.61 | 0.20 | -0.21 | -0.69 | -0.95 | -0.31 | -0.23 | 0.15 |
| Q16555 | Dihydropyrimidinase-related protein 2 | DPYSL2 | -0.39 | 1.83 | -0.76 | -0.62 | 1.23 | 1.08 | 0.86 | -0.86 | -0.67 | 0.69 | 0.64 | -1.60 |
| P17612 | cAMP-dependent protein kinase catalytic subunit alpha | PRKACA | -0.39 | 1.45 | -1.05 | -0.33 | 0.79 | 1.10 | 1.02 | -1.04 | 0.64 | -1.52 | 0.92 | -0.52 |
| O43395 | U4/U6 small nuclear ribonucleoprotein Prp3 | PRPF3 | -0.39 | 0.75 | -0.48 | -1.00 | 0.73 | 0.71 | 0.95 | -0.82 | -0.86 | -1.42 | 0.95 | 1.23 |
| Q9H9P5 | Putative E3 ubiquitin-protein ligase UNKL | UNKL | -0.39 | 0.56 | 0.73 | 0.35 | -1.17 | 0.12 | 0.69 | 2.01 | -0.84 | -1.24 | -0.25 | -0.39 |
| Q9Y2Q3 | Glutathione S-transferase kappa 1 | GSTK1 | -0.39 | 2.08 | 0.63 | 0.47 | 1.15 | -0.73 | 0.46 | -1.46 | -1.40 | -0.40 | -0.16 | 1.43 |
| Q9UKY1 | Zinc fingers and homeoboxes protein 1 | ZHX1 | -0.39 | 0.62 | -0.49 | -0.79 | 1.63 | -0.64 | 1.07 | -0.73 | -0.67 | -1.11 | 1.20 | 0.53 |
| Q8WXG6 | MAP kinase-activating death domain protein | MADD | -0.39 | 0.86 | -0.80 | 0.98 | 0.26 | 0.97 | -0.39 | -2.03 | 0.03 | -0.76 | 0.88 | 0.86 |
| P50991 | T-complex protein 1 subunit delta | CCT4 | -0.39 | 1.48 | 0.25 | 0.27 | 0.37 | 0.64 | 0.09 | -2.22 | -1.22 | 1.18 | 0.70 | -0.06 |
| Q15878 | Voltage-dependent R-type calcium channel subunit alpha-1E | CACNA1E | -0.39 | 0.37 | 0.16 | 0.54 | -0.09 | 0.31 | -0.41 | 0.23 | 1.55 | -2.38 | 0.43 | -0.33 |
| P55327 | Tumor protein D52 | TPD52 | -0.39 | 0.54 | -0.36 | -0.26 | -1.24 | 1.36 | 1.19 | -1.42 | -0.61 | -0.24 | 1.18 | 0.40 |
| Q96F85 | CB1 cannabinoid receptor-interacting protein 1 | CNRIP1 | -0.39 | 1.58 | 0.52 | 1.39 | -1.80 | 0.76 | 0.78 | -1.08 | -0.55 | -0.54 | 0.75 | -0.23 |
| Q14141 | Septin-6 | SEPTIN6 | -0.39 | 1.21 | -0.16 | 2.06 | 0.09 | -0.25 | -0.41 | -1.50 | 0.50 | -1.03 | 0.93 | -0.24 |
| Q15119 | [Pyruvate dehydrogenase (acetyl-transferring)] kinase isozyme 2, | PDK2 | -0.40 | 0.61 | -0.32 | -0.32 | 2.34 | -0.36 | -0.57 | 1.31 | -0.43 | -0.73 | -0.48 | -0.44 |
| P53582 | Methionine aminopeptidase 1 | METAP1 | -0.40 | 0.44 | 0.86 | -0.16 | -0.38 | 1.08 | -0.82 | 0.14 | -2.25 | 0.28 | 1.03 | 0.21 |
| P60484 | Phosphatidylinositol 3,4,5-trisphosphate 3-phosphatase and dual-s | PTEN | -0.40 | 1.94 | -0.55 | 1.17 | 0.09 | 0.94 | 0.24 | -1.89 | -1.43 | 0.34 | 0.69 | 0.39 |
| P16870 | Carboxypeptidase E | CPE | -0.40 | 0.54 | 0.85 | 1.54 | -0.17 | -1.29 | -0.24 | 0.38 | 0.78 | 0.61 | -1.21 | -1.25 |
| Q8N8A2 | Serine/threonine-protein phosphatase 6 regulatory ankyrin repeat | ANKRD44 | -0.40 | 0.67 | -1.11 | 0.73 | 1.90 | 0.08 | -0.78 | 0.41 | -0.45 | -1.49 | 0.02 | 0.66 |
| P07686 | Beta-hexosaminidase subunit beta | HEXB | -0.40 | 0.23 | -1.32 | 0.62 | 0.45 | 0.51 | 0.07 | -1.35 | -1.37 | 0.28 | 0.61 | 1.50 |
| Q9HB71 | Calcyclin-binding protein | CACYBP | -0.40 | 1.68 | -0.96 | 1.50 | 0.14 | 0.75 | 0.27 | -1.72 | 0.58 | -1.21 | 0.63 | 0.03 |
| P05062 | Fructose-bisphosphate aldolase B | ALDOB | -0.40 | 1.57 | 0.83 | 0.37 | 2.01 | -0.34 | -1.22 | -0.88 | 0.01 | 0.65 | -0.28 | -1.12 |
| Q96MZ0 | Ganglioside-induced differentiation-associated protein 1-like 1 | GDAP1L1 | -0.40 | 0.50 | -0.47 | 0.81 | -1.34 | 0.67 | 0.98 | -0.48 | 0.65 | -1.92 | 0.53 | 0.57 |
| P10636 | Microtubule-associated protein tau | MAPT | -0.40 | 1.18 | 0.82 | 0.24 | 0.92 | -0.04 | -0.60 | -1.17 | 1.88 | -1.37 | -0.15 | -0.52 |
| Q93008 | Probable ubiquitin carboxyl-terminal hydrolase FAF-X | USP9X | -0.40 | 0.66 | 1.39 | -0.33 | 0.36 | -1.40 | 0.80 | 1.30 | -0.02 | 0.09 | -0.81 | -1.37 |
| Q9NZJ4 | Sacsin | SACS | -0.40 | 0.73 | 0.18 | 1.02 | -1.54 | 0.31 | 0.92 | 1.56 | -0.65 | -1.06 | 0.07 | -0.80 |
| P51114 | Fragile X mental retardation syndrome-related protein 1 | FXR1 | -0.40 | 0.62 | 1.18 | -0.77 | -0.78 | 0.25 | 0.89 | 0.55 | 0.82 | -2.14 | 0.05 | -0.05 |
| P61088 | Ubiquitin-conjugating enzyme E2 N | UBE2N | -0.40 | 2.08 | 0.33 | 1.07 | 0.87 | -1.25 | 0.94 | -1.55 | 0.07 | 0.54 | -1.32 | 0.30 |
| Q9UI12 | V-type proton ATPase subunit H | ATP6V1H | -0.40 | 2.12 | -0.06 | 1.83 | 0.71 | 0.61 | -1.10 | -1.33 | 0.39 | -0.54 | 0.51 | -1.03 |
| Q2TAZ0 | Autophagy-related protein 2 homolog A | ATG2A | -0.40 | 0.53 | 1.86 | 1.24 | -0.99 | -0.71 | -0.71 | -0.66 | -0.59 | 0.81 | 0.40 | -0.64 |
| Q7L2E3 | ATP-dependent RNA helicase DHX30 | DHX30 | -0.40 | 1.36 | -0.26 | 2.56 | -0.02 | 0.20 | -0.98 | -0.17 | -1.02 | -0.37 | -0.27 | 0.34 |
| P14678 | Small nuclear ribonucleoprotein-associated proteins B and B' | SNRPB | -0.40 | 1.35 | 0.34 | -0.13 | 1.17 | -0.56 | 0.65 | -1.83 | -0.93 | 0.29 | -0.50 | 1.49 |
| P54750 | Calcium/calmodulin-dependent 3',5'-cyclic nucleotide phosphodiest | PDE1A | -0.40 | 1.30 | 0.20 | 0.93 | -1.63 | 1.21 | 0.71 | -1.52 | 0.35 | -0.32 | 0.73 | -0.64 |
| Q96FJ2 | Dynein light chain 2, cytoplasmic | DYNLL2 | -0.40 | 3.09 | 1.23 | 1.33 | -1.06 | 0.08 | 0.93 | -1.85 | -0.11 | -0.16 | -0.51 | 0.11 |
| Q5VZF2 | Muscleblind-like protein 2 | MBNL2 | -0.40 | 0.68 | 1.24 | -1.10 | -1.52 | 0.95 | 1.29 | -0.11 | -0.26 | -0.98 | 0.58 | -0.08 |
| Q92599 | Septin-8 | SEPTIN8 | -0.40 | 2.24 | 0.27 | 0.04 | 2.32 | -0.62 | 0.06 | -1.57 | -0.70 | 0.34 | -0.22 | 0.09 |
| Q5VVW2 | GTPase-activating Rap/Ran-GAP domain-like protein 3 | GARNL3 | -0.41 | 1.35 | 0.84 | -0.58 | -1.08 | 0.77 | 1.49 | 0.58 | -0.62 | -1.48 | 0.76 | -0.69 |
| O95260 | Arginyl-tRNA--protein transferase 1 | ATE1 | -0.41 | 0.49 | 0.73 | 0.56 | -1.48 | 0.46 | 0.37 | 0.65 | 0.58 | -2.09 | -0.37 | 0.60 |
| O43670 | BUB3-interacting and GLEBS motif-containing protein ZNF207 | ZNF207 | -0.41 | 0.78 | 0.17 | -1.15 | -0.41 | 1.09 | 1.25 | -0.76 | -1.71 | 0.74 | 0.82 | -0.02 |
| Q9NZ63 | Telomere length and silencing protein 1 homolog | C9orf78 | -0.41 | 0.39 | 2.30 | -0.39 | -0.53 | -0.36 | -0.50 | 1.38 | -0.40 | -0.71 | -0.41 | -0.38 |
| Q8N0Z6 | Tetratricopeptide repeat protein 5 | TTC5 | -0.41 | 0.66 | -0.50 | 0.65 | -0.18 | 0.11 | 0.76 | 1.07 | 0.01 | -2.40 | -0.35 | 0.83 |
| Q9NX05 | Constitutive coactivator of PPAR-gamma-like protein 2 | FAM120C | -0.41 | 0.47 | 1.80 | 0.36 | -0.80 | -0.18 | -0.56 | 1.59 | -0.28 | -1.37 | -0.30 | -0.25 |
| Q13449 | Limbic system-associated membrane protein | LSAMP | -0.41 | 0.66 | -0.79 | 1.28 | -0.25 | 0.51 | 0.08 | -1.47 | -1.35 | 1.47 | 0.02 | 0.50 |
| P49790 | Nuclear pore complex protein Nup153 | NUP153 | -0.41 | 0.79 | 2.63 | -0.27 | -0.55 | -0.25 | -0.58 | 0.35 | -0.40 | -0.82 | 0.36 | -0.46 |
| Q9H0J9 | Protein mono-ADP-ribosyltransferase PARP12 | PARP12 | -0.41 | 0.69 | 0.37 | 1.49 | -0.83 | 0.50 | -0.68 | -0.68 | -0.81 | 1.24 | 0.81 | -1.39 |

| | | | | | | | | | | | | | | |
|---|---|---|---|---|---|---|---|---|---|---|---|---|---|---|
| P11177 | Pyruvate dehydrogenase E1 component subunit beta, mitochondria | PDHB | -0.41 | 1.54 | 0.29 | -0.74 | 1.63 | 1.16 | -0.75 | -0.61 | 1.29 | -0.96 | -0.69 | -0.62 |
| Q16822 | Phosphoenolpyruvate carboxykinase [GTP], mitochondrial | PCK2 | -0.41 | 0.39 | 1.16 | -0.74 | 1.41 | -0.63 | -0.68 | 1.39 | -0.76 | -0.95 | -0.74 | 0.54 |
| O15090 | Zinc finger protein 536 | ZNF536 | -0.41 | 1.78 | 0.49 | 2.04 | 0.60 | -0.38 | -0.98 | -1.15 | -0.88 | 0.58 | -0.73 | 0.40 |
| Q92551 | Inositol hexakisphosphate kinase 1 | IP6K1 | -0.41 | 0.86 | -0.14 | 1.71 | -0.86 | 0.80 | -0.49 | -0.38 | -0.73 | -0.92 | 1.60 | -0.60 |
| C9JTQ0 | Ankyrin repeat domain-containing protein 63 | ANKRD63 | -0.41 | 2.05 | 0.90 | 0.32 | -0.17 | 0.66 | 0.32 | 0.54 | -2.43 | -0.94 | 0.14 | 0.65 |
| Q96EP5 | DAZ-associated protein 1 | DAZAP1 | -0.41 | 1.04 | 0.40 | 0.07 | -0.29 | 0.92 | 0.12 | -0.57 | -2.47 | 0.13 | 0.84 | 0.85 |
| P35659 | Protein DEK | DEK | -0.41 | 0.63 | -0.68 | -0.18 | 0.19 | 0.70 | 0.76 | 0.50 | -2.16 | -0.53 | 1.49 | -0.09 |
| P16591 | Tyrosine-protein kinase Fer | FER | -0.41 | 0.38 | 1.49 | -0.64 | -0.87 | 0.45 | 0.08 | 1.02 | 0.36 | -2.02 | 0.32 | -0.18 |
| P00533 | Epidermal growth factor receptor | EGFR | -0.41 | 0.22 | -0.99 | -1.12 | 1.04 | 0.43 | 0.96 | -0.18 | -1.87 | 0.49 | 0.65 | 0.60 |
| Q9GZT9 | Egl nine homolog 1 | EGLN1 | -0.41 | 1.09 | 0.15 | -0.60 | 1.55 | 1.22 | -1.08 | -1.06 | -0.06 | -1.15 | 1.07 | -0.03 |
| Q8N5J2 | Ubiquitin carboxyl-terminal hydrolase MINDY-1 | MINDY1 | -0.41 | 0.56 | 0.88 | -0.09 | 0.62 | 0.59 | -1.29 | -0.54 | -1.81 | -0.40 | 1.13 | 0.91 |
| Q9NRW1 | Ras-related protein Rab-6B | RAB6B | -0.41 | 2.08 | 0.65 | 1.44 | -1.07 | 1.20 | -0.27 | -0.46 | -1.11 | -1.19 | 0.98 | -0.18 |
| Q15059 | Bromodomain-containing protein 3 | BRD3 | -0.42 | 0.64 | -1.06 | 1.30 | -0.17 | 0.76 | -0.03 | -1.24 | -1.13 | -0.60 | 1.16 | 1.02 |
| P62701 | 40S ribosomal protein S4, X isoform | RPS4X | -0.42 | 1.57 | 0.90 | 0.52 | 1.72 | -0.65 | -0.88 | 0.48 | -1.20 | 0.70 | -0.40 | -1.20 |
| Q9NP61 | ADP-ribosylation factor GTPase-activating protein 3 | ARFGAP3 | -0.42 | 0.94 | -0.39 | 0.28 | -0.38 | 1.39 | 0.22 | -0.61 | -1.55 | -1.17 | 1.24 | 0.98 |
| Q8N8R7 | ARL14 effector protein | ARL14EP | -0.42 | 1.05 | 1.75 | 1.28 | -0.91 | -0.37 | -0.56 | -0.52 | -0.42 | -1.04 | -0.37 | 1.16 |
| Q14008 | Cytoskeleton-associated protein 5 | CKAP5 | -0.42 | 0.69 | 0.51 | 1.80 | -0.69 | -0.19 | -0.57 | 0.04 | 0.48 | -1.99 | -0.05 | 0.67 |
| P48380 | Transcription factor RFX3 | RFX3 | -0.42 | 0.52 | -0.47 | -0.51 | -0.83 | 1.49 | 1.00 | -0.52 | 1.75 | -0.84 | -0.57 | -0.50 |
| Q7Z408 | CUB and sushi domain-containing protein 2 | CSMD2 | -0.42 | 0.57 | -0.53 | 0.59 | -0.17 | 1.15 | -0.31 | -0.45 | -0.10 | -2.16 | 1.19 | 0.78 |
| Q08752 | Peptidyl-prolyl cis-trans isomerase D | PPID | -0.42 | 0.97 | 0.75 | 1.34 | -0.98 | 0.62 | -0.61 | -0.59 | -1.46 | -0.89 | 0.74 | 1.07 |
| P61962 | DDB1- and CUL4-associated factor 7 | DCAF7 | -0.42 | 1.00 | 1.10 | -0.84 | -1.06 | 0.73 | 1.21 | -0.11 | 0.23 | -1.81 | -0.23 | 0.77 |
| Q5SQN1 | Synaptosomal-associated protein 47 | SNAP47 | -0.42 | 0.50 | 1.20 | 0.90 | -1.31 | 0.93 | -1.07 | 0.15 | 0.42 | -1.48 | 0.67 | -0.41 |
| P07814 | Bifunctional glutamate/proline--tRNA ligase | EPRS | -0.42 | 1.53 | -0.46 | 1.41 | 0.61 | 0.03 | 0.01 | 1.12 | -1.03 | -1.99 | -0.08 | 0.38 |
| P20936 | Ras GTPase-activating protein 1 | RASA1 | -0.42 | 1.15 | -0.95 | 1.97 | -0.54 | 0.62 | 0.17 | -0.57 | 0.49 | -1.68 | 0.27 | 0.21 |
| P49207 | 60S ribosomal protein L34 | RPL34 | -0.42 | 0.78 | -0.95 | -0.17 | -0.59 | 0.10 | 2.59 | -0.89 | -0.15 | -0.29 | 0.05 | 0.32 |
| Q16352 | Alpha-internexin | INA | -0.42 | 0.94 | 0.35 | 0.11 | 1.63 | -0.04 | -0.96 | 0.11 | 1.18 | -1.94 | -0.36 | -0.08 |
| Q92876 | Kallikrein-6 | KLK6 | -0.42 | 0.70 | -0.09 | -0.70 | 1.15 | 0.93 | -0.43 | -0.37 | -0.62 | 0.95 | 1.07 | -1.89 |
| Q3V6T2 | Girdin | CCDC88A | -0.42 | 1.23 | 0.94 | 0.05 | 0.50 | 0.41 | -0.51 | 0.67 | 1.52 | -1.70 | -0.76 | -1.11 |
| Q96Q15 | Serine/threonine-protein kinase SMG1 | SMG1 | -0.43 | 0.54 | 0.02 | 0.79 | 0.44 | -0.31 | -0.24 | 1.53 | -2.27 | -0.52 | 0.03 | 0.52 |
| P62834 | Ras-related protein Rap-1A | RAP1A | -0.43 | 0.52 | -0.27 | 0.33 | 0.49 | 0.54 | -0.40 | 0.28 | -2.64 | 0.67 | 0.78 | 0.23 |
| Q9NQR4 | Omega-amidase NIT2 | NIT2 | -0.43 | 0.62 | 0.11 | 0.07 | 0.92 | -0.06 | -0.24 | 0.95 | -2.60 | 0.14 | 0.09 | 0.62 |
| Q9Y4F9 | Rho family-interacting cell polarization regulator 2 | RIPOR2 | -0.43 | 1.14 | -0.02 | -0.37 | -0.59 | 2.74 | -0.44 | -0.22 | -0.04 | -0.84 | -0.10 | -0.11 |
| Q6V1X1 | Dipeptidyl peptidase 8 | DPP8 | -0.43 | 1.23 | 1.48 | -1.57 | 0.22 | 1.65 | -0.40 | -0.81 | -0.46 | 0.57 | -0.32 | -0.36 |
| O60285 | NUAK family SNF1-like kinase 1 | NUAK1 | -0.43 | 0.38 | 0.74 | 0.16 | -1.58 | 0.74 | 0.45 | 0.41 | 1.08 | -1.67 | 0.55 | -0.88 |
| Q9H425 | Uncharacterized protein C1orf198 | C1orf198 | -0.43 | 0.35 | -0.74 | 0.16 | -0.05 | 0.33 | 0.78 | -0.54 | -2.31 | 1.16 | 0.61 | 0.59 |
| P31323 | cAMP-dependent protein kinase type II-beta regulatory subunit | PRKAR2B | -0.43 | 0.96 | 0.50 | 0.23 | 0.02 | 1.15 | -0.77 | -1.29 | 0.65 | -1.81 | 1.18 | 0.13 |
| O43150 | Arf-GAP with SH3 domain, ANK repeat and PH domain-containing | ASAP2 | -0.43 | 0.55 | -0.02 | 0.80 | -1.63 | 1.09 | 0.47 | 0.15 | 0.57 | -1.82 | -0.33 | 0.73 |
| Q92667 | A-kinase anchor protein 1, mitochondrial | AKAP1 | -0.43 | 0.66 | 1.57 | -0.62 | -0.82 | 1.32 | -0.63 | -0.72 | -0.56 | -1.12 | 0.84 | 0.74 |
| P47736 | Rap1 GTPase-activating protein 1 | RAP1GAP | -0.43 | 1.83 | 0.80 | -0.18 | -0.89 | 1.78 | 0.29 | -1.33 | -0.67 | -1.10 | 0.63 | 0.68 |
| Q8TBX8 | Phosphatidylinositol 5-phosphate 4-kinase type-2 gamma | PIP4K2C | -0.43 | 0.82 | 0.51 | 0.94 | -1.00 | 0.85 | -0.32 | -0.04 | 1.12 | -2.12 | 0.42 | -0.37 |
| O00479 | High mobility group nucleosome-binding domain-containing protei | HMGN4 | -0.43 | 0.92 | 1.22 | 0.81 | 0.97 | -0.47 | -1.44 | 0.09 | 0.11 | -1.40 | -0.85 | 0.97 |
| P46527 | Cyclin-dependent kinase inhibitor 1B | CDKN1B | -0.43 | 0.64 | -1.20 | 1.96 | 0.51 | 0.54 | -1.01 | -0.98 | -0.75 | 0.15 | -0.01 | 0.78 |
| Q15477 | Helicase SKI2W | SKIV2L | -0.43 | 0.72 | -1.16 | -0.06 | -0.28 | 0.85 | 1.53 | 0.03 | -0.51 | 0.88 | 0.51 | -1.79 |
| Q9H0K1 | Serine/threonine-protein kinase SIK2 | SIK2 | -0.43 | 0.72 | 0.23 | 0.45 | -1.43 | 1.01 | 0.62 | 0.53 | -1.18 | -1.63 | 0.70 | 0.70 |
| Q9UF11 | Pleckstrin homology domain-containing family B member 1 | PLEKHB1 | -0.43 | 1.22 | -1.33 | 0.36 | 0.67 | 0.23 | 1.42 | -1.41 | -0.71 | 0.86 | 0.79 | -0.87 |
| Q16658 | Fascin | FSCN1 | -0.43 | 1.70 | -0.83 | 0.38 | 0.56 | 1.29 | 0.33 | -1.14 | 0.51 | -0.77 | 1.24 | -1.55 |
| Q14938 | Nuclear factor 1 X-type | NFIX | -0.43 | 0.55 | 1.05 | -0.89 | -1.53 | 0.84 | 1.24 | 0.20 | -1.20 | -0.72 | 0.64 | 0.36 |
| Q14157 | Ubiquitin-associated protein 2-like | UBAP2L | -0.43 | 1.24 | 1.13 | -1.62 | 1.48 | -0.11 | 0.49 | 0.89 | -0.67 | -1.11 | -0.42 | -0.06 |
| P36969 | Phospholipid hydroperoxide glutathione peroxidase | GPX4 | -0.43 | 0.58 | -0.90 | 0.44 | 0.51 | -0.03 | 0.73 | -1.02 | -1.83 | 1.64 | 0.28 | 0.18 |
| Q15042 | Rab3 GTPase-activating protein catalytic subunit | RAB3GAP1 | -0.43 | 1.33 | 1.44 | 0.92 | -0.51 | 0.16 | -0.57 | 0.71 | 0.00 | -2.11 | -0.53 | 0.50 |
| Q06203 | Amidophosphoribosyltransferase | PPAT | -0.43 | 0.52 | -0.98 | 0.48 | 0.26 | 0.52 | 0.39 | -0.77 | -2.19 | 1.11 | 0.66 | 0.51 |
| Q9UN70 | Protocadherin gamma-C3 | PCDHGC3 | -0.43 | 1.99 | 2.48 | 0.04 | -0.78 | 0.34 | -0.13 | 0.28 | -1.02 | -0.31 | 0.03 | -0.94 |
| O95625 | Zinc finger and BTB domain-containing protein 11 | ZBTB11 | -0.44 | 0.51 | -0.98 | 0.95 | -0.22 | 1.35 | -0.45 | -1.07 | -1.02 | -0.73 | 1.14 | 1.02 |
| Q00341 | Vigilin | HDLBP | -0.44 | 2.14 | 1.59 | 1.20 | 0.88 | -0.03 | -1.56 | -0.56 | -1.19 | -0.27 | 0.13 | -0.18 |
| P55039 | Developmentally-regulated GTP-binding protein 2 | DRG2 | -0.44 | 1.41 | 1.14 | 1.58 | -0.58 | -0.52 | -0.13 | -0.49 | -0.13 | 1.35 | -1.00 | -1.22 |
| Q92878 | DNA repair protein RAD50 | RAD50 | -0.44 | 0.38 | 1.06 | 1.01 | -2.07 | 0.47 | 0.04 | 0.95 | 0.27 | -0.65 | -0.11 | -0.96 |
| P50402 | Emerin | EMD | -0.44 | 0.96 | 0.34 | 0.78 | 0.25 | 0.71 | -0.95 | 1.15 | -0.84 | -2.13 | 0.23 | 0.46 |
| P16383 | GC-rich sequence DNA-binding factor 2 | GCFC2 | -0.44 | 0.40 | 1.04 | 0.47 | 0.76 | -0.89 | -0.85 | -0.81 | 1.66 | -1.10 | -0.82 | 0.54 |
| Q8NDA8 | Maestro heat-like repeat-containing protein family member 1 | MROH1 | -0.44 | 0.48 | -0.43 | 0.33 | -0.10 | 0.71 | 0.12 | -0.41 | -2.47 | 0.35 | 0.95 | 0.94 |
| Q7Z2T5 | TRMT1-like protein | TRMT1L | -0.44 | 1.12 | 1.87 | -0.38 | -0.87 | 1.25 | -0.60 | -0.52 | -0.28 | -1.20 | 0.93 | -0.20 |
| Q9UDY2 | Tight junction protein ZO-2 | TJP2 | -0.44 | 1.25 | -1.18 | -0.50 | 1.30 | 0.52 | 1.22 | -0.69 | -1.57 | 0.01 | 0.97 | -0.09 |
| Q9GZN7 | Protein rogdi homolog | ROGDI | -0.44 | 0.60 | 0.03 | 1.15 | 0.05 | 0.58 | -1.04 | -0.61 | -1.02 | -1.35 | 1.63 | 0.60 |
| P02452 | Collagen alpha-1(I) chain | COL1A1 | -0.44 | 0.32 | -0.02 | 0.51 | 1.72 | -0.89 | -0.88 | -0.88 | 1.45 | -1.03 | -0.34 | 0.37 |
| P61981 | 14-3-3 protein gamma | YWHAG | -0.44 | 3.15 | 0.09 | 2.42 | -0.28 | 0.04 | 0.31 | -1.26 | 0.25 | -1.07 | -0.14 | -0.36 |
| Q9ULK4 | Mediator of RNA polymerase II transcription subunit 23 | MED23 | -0.44 | 0.49 | 1.23 | 0.19 | -0.99 | -0.75 | 0.96 | 0.74 | 1.37 | -1.07 | -0.89 | -0.79 |
| Q9Y2K9 | Syntaxin-binding protein 5-like | STXBP5L | -0.44 | 0.51 | 0.90 | 0.65 | 0.09 | 0.41 | -1.38 | -0.15 | 1.11 | -2.07 | 0.10 | 0.35 |
| O60645 | Exocyst complex component 3 | EXOC3 | -0.44 | 0.85 | 0.08 | -0.13 | -1.45 | 1.34 | 1.18 | -0.12 | -0.94 | -1.05 | 1.28 | -0.18 |
| Q8N9M1 | Uncharacterized protein C19orf47 | C19orf47 | -0.44 | 0.71 | 2.53 | -0.48 | -0.53 | -0.27 | -0.37 | -0.38 | 0.94 | -0.80 | -0.36 | -0.28 |
| O43189 | PHD finger protein 1 | PHF1 | -0.44 | 0.51 | 1.43 | -0.25 | -1.58 | 0.29 | 0.78 | 0.17 | 0.97 | -1.60 | -0.35 | 0.14 |
| P78356 | Phosphatidylinositol 5-phosphate 4-kinase type-2 beta | PIP4K2B | -0.44 | 0.90 | 0.58 | 1.79 | -0.78 | 0.12 | -0.66 | 0.37 | 0.38 | -2.01 | 0.30 | -0.09 |
| P40145 | Adenylate cyclase type 8 | ADCY8 | -0.44 | 0.37 | -0.83 | -0.64 | -0.67 | 1.49 | 1.15 | -1.01 | -1.05 | 0.27 | 1.30 | -0.01 |
| Q92733 | Proline-rich protein PRCC | PRCC | -0.44 | 0.66 | 1.60 | -0.33 | -1.11 | 0.72 | -0.06 | 0.73 | -0.91 | -1.45 | 1.03 | -0.21 |
| Q8N9T8 | Protein KRI1 homolog | KRI1 | -0.44 | 0.62 | -0.52 | 1.25 | -0.80 | 0.12 | 0.73 | 1.97 | -0.50 | -1.06 | -0.53 | -0.66 |
| P0DP58 | Ly-6/neurotoxin-like protein 1 | LYNX1 | -0.44 | 1.43 | 0.71 | 2.21 | -0.72 | -0.91 | 0.24 | -1.18 | -0.15 | 0.60 | -0.63 | -0.18 |
| Q9BTE1 | Dynactin subunit 5 | DCTN5 | -0.44 | 1.74 | 0.50 | 1.03 | 0.36 | 0.49 | -0.60 | 0.29 | -0.01 | -2.39 | 0.90 | -0.57 |
| Q9BT23 | LIM domain-containing protein 2 | LIMD2 | -0.45 | 0.44 | -0.57 | 1.78 | -0.84 | -0.66 | 0.87 | -0.71 | -0.68 | -0.99 | 1.10 | 0.70 |
| Q13131 | 5'-AMP-activated protein kinase catalytic subunit alpha-1 | PRKAA1 | -0.45 | 1.07 | 0.51 | 1.99 | -1.26 | 0.47 | -0.48 | 0.32 | -0.56 | -1.48 | 0.38 | 0.12 |
| P62330 | ADP-ribosylation factor 6 | ARF6 | -0.45 | 1.11 | 0.82 | 0.00 | -0.88 | 0.70 | 0.62 | -0.21 | -0.59 | -2.19 | 0.78 | 0.94 |
| Q07960 | Rho GTPase-activating protein 1 | ARHGAP1 | -0.45 | 1.11 | -0.74 | 1.49 | -0.04 | 0.18 | 0.36 | -1.15 | 1.41 | -1.64 | 0.20 | -0.07 |
| Q8N5F7 | NF-kappa-B-activating protein | NKAP | -0.45 | 0.53 | 0.30 | -0.07 | -1.53 | 0.45 | 1.53 | 0.00 | 1.00 | -1.72 | 0.12 | -0.09 |
| O95970 | Leucine-rich glioma-inactivated protein 1 | LGI1 | -0.45 | 0.44 | -0.99 | 1.38 | 0.81 | 0.61 | -1.23 | -1.10 | -1.15 | 0.77 | 0.54 | 0.37 |
| P13637 | Sodium/potassium-transporting ATPase subunit alpha-3 | ATP1A3 | -0.45 | 1.25 | 1.29 | 1.23 | -0.71 | -1.10 | 0.66 | 0.07 | -1.22 | -0.46 | -0.86 | 1.11 |
| P18754 | Regulator of chromosome condensation | RCC1 | -0.45 | 0.49 | 1.12 | -0.03 | -0.33 | -0.13 | 0.02 | 1.50 | -2.25 | -0.28 | 0.46 | -0.08 |

| | | | | | | | | | | | | | | |
|---|---|---|---|---|---|---|---|---|---|---|---|---|---|---|
| O14548 | Cytochrome c oxidase subunit 7A-related protein, mitochondrial | COX7A2L | -0.45 | 1.50 | 0.35 | -0.22 | -0.66 | 2.69 | -0.52 | -0.22 | 0.02 | -0.81 | -0.38 | -0.23 |
| Q86V21 | Acetoacetyl-CoA synthetase | AACS | -0.45 | 0.37 | 0.96 | 0.78 | -1.75 | 0.29 | 0.23 | 0.71 | 0.17 | -1.90 | 0.32 | 0.20 |
| Q9Y2X3 | Nucleolar protein 58 | NOP58 | -0.45 | 0.62 | 0.60 | -1.51 | 0.62 | 0.75 | 0.33 | 0.26 | -1.80 | -0.84 | 0.80 | 0.80 |
| Q9UBT2 | SUMO-activating enzyme subunit 2 | UBA2 | -0.45 | 0.65 | 1.41 | -0.69 | -0.87 | 0.59 | 0.36 | 0.58 | -2.11 | 0.20 | 0.68 | -0.16 |
| Q9ULG1 | Chromatin-remodeling ATPase INO80 | INO80 | -0.45 | 0.45 | -0.74 | 0.83 | 0.72 | 0.62 | -0.84 | 1.74 | -0.80 | -1.16 | 0.54 | -0.92 |
| Q8N5M4 | Tetratricopeptide repeat protein 9C | TTC9C | -0.45 | 0.61 | -0.55 | 0.66 | -0.88 | -0.25 | 1.78 | -0.70 | -0.58 | -0.99 | -0.05 | 1.56 |
| P31483 | Nucleolysin TIA-1 isoform p40 | TIA1 | -0.45 | 1.77 | 0.05 | -0.06 | -0.49 | -0.40 | 2.72 | -0.34 | -0.14 | -0.96 | -0.34 | -0.05 |
| Q14527 | Helicase-like transcription factor | HLTF | -0.45 | 0.78 | -0.95 | 1.32 | -0.27 | 1.00 | -0.16 | -1.14 | -1.35 | -0.42 | 1.12 | 0.84 |
| A3KN83 | Protein strawberry notch homolog 1 | SBNO1 | -0.45 | 0.33 | 0.19 | 1.24 | -1.03 | -0.96 | 1.02 | 0.00 | 0.35 | -1.18 | 1.37 | -1.00 |
| Q9P2E3 | NFX1-type zinc finger-containing protein 1 | ZNFX1 | -0.46 | 0.58 | -1.51 | 0.62 | 0.89 | 0.28 | 0.45 | -2.14 | 0.14 | 0.62 | 0.16 | 0.49 |
| Q8NB37 | Glutamine amidotransferase-like class 1 domain-containing protei | GATD1 | -0.46 | 1.39 | -0.51 | -0.33 | 0.20 | 1.30 | 0.83 | -2.04 | 0.23 | -1.02 | 0.47 | 0.87 |
| Q9NR80 | Rho guanine nucleotide exchange factor 4 | ARHGEF4 | -0.46 | 0.22 | -0.85 | -0.37 | 1.88 | -0.02 | -0.33 | -0.77 | -0.97 | 1.70 | -0.01 | -0.26 |
| Q6DKI2 | Galectin-9C | LGALS9C | -0.46 | 1.07 | 0.10 | 1.79 | -1.55 | 0.30 | 0.57 | -0.13 | -1.27 | -0.87 | 0.60 | 0.46 |
| P22307 | Non-specific lipid-transfer protein | SCP2 | -0.46 | 1.20 | 0.44 | -1.15 | -0.19 | 0.32 | 1.91 | -1.55 | -0.80 | 0.56 | 0.57 | -0.11 |
| P09651 | Heterogeneous nuclear ribonucleoprotein A1 | HNRNPA1 | -0.46 | 1.56 | 1.64 | -0.39 | -0.77 | 0.74 | 0.38 | 0.38 | -0.87 | -1.90 | 0.55 | 0.23 |
| Q9HCK4 | Roundabout homolog 2 | ROBO2 | -0.46 | 1.06 | 0.19 | 1.04 | -1.66 | 0.67 | 0.97 | 0.22 | 0.33 | -1.86 | 0.27 | -0.17 |
| O43252 | Bifunctional 3'-phosphoadenosine 5'-phosphosulfate synthase 1 | PAPSS1 | -0.46 | 0.49 | 1.21 | -0.89 | 0.37 | 0.87 | -0.92 | 1.23 | -0.80 | -1.19 | 0.92 | -0.79 |
| O14776 | Transcription elongation regulator 1 | TCERG1 | -0.46 | 1.18 | 1.94 | -0.60 | -0.99 | 0.07 | 0.90 | 0.70 | -0.50 | -1.44 | 0.32 | -0.39 |
| Q9P242 | Neuronal tyrosine-phosphorylated phosphoinositide-3-kinase adapt | NYAP2 | -0.46 | 0.53 | 0.94 | 1.32 | -1.26 | 0.82 | -1.15 | 0.37 | -0.57 | -1.39 | 0.40 | 0.50 |
| Q9UGU0 | Transcription factor 20 | TCF20 | -0.46 | 0.58 | -0.47 | 1.83 | 0.77 | -0.68 | -0.71 | -0.66 | -0.62 | -1.01 | 0.15 | 1.41 |
| P04424 | Argininosuccinate lyase | ASL | -0.46 | 0.65 | -0.22 | -0.49 | 2.33 | -0.31 | -0.50 | -0.40 | -0.37 | -0.86 | 1.29 | -0.48 |
| P51608 | Methyl-CpG-binding protein 2 | MECP2 | -0.46 | 1.58 | 0.77 | 1.16 | 1.03 | 0.45 | -1.76 | -1.03 | -0.25 | -1.13 | 0.51 | 0.26 |
| Q765P7 | Protein MTSS 2 | MTSS2 | -0.46 | 0.70 | 0.26 | 0.88 | -1.54 | 1.09 | 0.18 | -1.15 | -1.32 | 0.56 | 1.08 | -0.03 |
| Q9HAN9 | Nicotinamide/nicotinic acid mononucleotide adenylyltransferase 1 | NMNAT1 | -0.46 | 1.06 | 1.52 | -0.77 | 1.11 | 0.11 | -0.77 | -0.82 | -0.68 | -1.33 | 0.94 | 0.69 |
| P51991 | Heterogeneous nuclear ribonucleoprotein A3 | HNRNPA3 | -0.46 | 0.77 | 2.11 | -1.28 | -0.44 | 0.05 | 0.51 | -0.20 | 0.84 | -1.29 | -0.14 | -0.16 |
| P53621 | Coatomer subunit alpha | COPA | -0.46 | 1.11 | -1.49 | 0.92 | 0.75 | 1.05 | 0.02 | -1.50 | -0.91 | -0.27 | 0.49 | 0.93 |
| Q13905 | Rap guanine nucleotide exchange factor 1 | RAPGEF1 | -0.46 | 1.34 | 0.13 | 2.64 | -0.73 | -0.07 | -0.48 | -0.29 | -0.15 | -1.03 | 0.14 | -0.15 |
| Q71U36 | Tubulin alpha-1A chain | TUBA1A | -0.46 | 1.70 | 0.73 | 1.68 | -0.90 | 0.10 | 0.10 | 0.18 | 0.06 | -2.11 | 0.49 | -0.34 |
| Q5U5Q3 | RNA-binding E3 ubiquitin-protein ligase MEX3C | MEX3C | -0.46 | 0.76 | -0.13 | -0.29 | -0.71 | -0.44 | 2.50 | -0.23 | -0.34 | -0.80 | -0.51 | 0.95 |
| O75534 | Cold shock domain-containing protein E1 | CSDE1 | -0.46 | 0.91 | 0.51 | 0.00 | -1.39 | 0.97 | 0.98 | -0.93 | -1.51 | -0.41 | 1.00 | 0.78 |
| Q13432 | Protein unc-119 homolog A | UNC119 | -0.46 | 0.44 | 0.68 | 0.93 | -1.77 | 0.70 | 0.04 | -0.14 | -1.73 | 0.67 | 0.75 | -0.12 |
| Q9P1Y6 | PHD and RING finger domain-containing protein 1 | PHRF1 | -0.46 | 1.31 | 2.78 | -0.17 | -0.64 | -0.18 | -0.31 | -0.32 | -0.07 | -0.70 | -0.12 | -0.26 |
| Q9Y4A5 | Transformation/transcription domain-associated protein | TRRAP | -0.46 | 0.94 | 0.64 | -0.56 | -1.10 | 1.87 | 0.25 | -0.43 | -0.71 | -1.28 | 1.07 | 0.25 |
| Q6ZSR9 | Uncharacterized protein FLJ45252 | Uncharacterized | -0.46 | 0.80 | -1.10 | 0.42 | 0.64 | 0.28 | 0.73 | -1.66 | -1.48 | 0.54 | 0.66 | 0.97 |
| Q8WWY3 | U4/U6 small nuclear ribonucleoprotein Prp31 | PRPF31 | -0.46 | 0.57 | -0.42 | -0.75 | 0.83 | 0.38 | 0.69 | -1.56 | -1.49 | 1.31 | 0.72 | 0.28 |
| P47755 | F-actin-capping protein subunit alpha-2 | CAPZA2 | -0.46 | 2.04 | 0.58 | 1.04 | 1.64 | 0.04 | -1.35 | -0.10 | -1.40 | -0.97 | 0.36 | 0.16 |
| P13861 | cAMP-dependent protein kinase type II-alpha regulatory subunit | PRKAR2A | -0.46 | 0.95 | -0.94 | 1.77 | 0.14 | 0.03 | 0.10 | -2.11 | 0.22 | 0.05 | 0.09 | 0.65 |
| Q96RK0 | Protein capicua homolog | CIC | -0.46 | 2.88 | 0.47 | -0.06 | -0.52 | 0.51 | 2.01 | 0.39 | -0.08 | -1.39 | -1.45 | 0.13 |
| Q6ZN55 | Zinc finger protein 574 | ZNF574 | -0.46 | 0.36 | -0.76 | -0.08 | 0.05 | 0.22 | 1.07 | -1.50 | -1.52 | 1.43 | 0.38 | 0.71 |
| Q68D91 | Metallo-beta-lactamase domain-containing protein 2 | MBLAC2 | -0.46 | 0.43 | -0.90 | 1.09 | -1.18 | 1.09 | 0.47 | -1.04 | -0.99 | -0.49 | 1.01 | 0.94 |
| O43752 | Syntaxin-6 | STX6 | -0.46 | 0.63 | 0.13 | 1.17 | -0.27 | 0.06 | -0.29 | -0.10 | 0.70 | -2.45 | 0.09 | 0.96 |
| Q9H9H5 | MAP6 domain-containing protein 1 | MAP6D1 | -0.47 | 0.77 | 1.85 | -1.35 | 0.50 | 0.14 | -0.22 | -0.35 | 1.13 | -1.12 | 0.24 | -0.83 |
| Q15080 | Neutrophil cytosol factor 4 | NCF4 | -0.47 | 0.72 | 1.22 | 1.14 | -1.80 | -0.25 | 0.58 | 1.05 | 0.01 | -0.96 | -0.63 | -0.35 |
| Q8WWQ0 | PH-interacting protein | PHIP | -0.47 | 0.45 | 1.78 | 0.14 | -0.45 | -0.20 | -0.69 | 1.21 | 0.55 | -1.78 | -0.30 | -0.26 |
| P51617 | Interleukin-1 receptor-associated kinase 1 | IRAK1 | -0.47 | 0.70 | 0.62 | 1.03 | -1.70 | 0.67 | 0.25 | 0.86 | -1.44 | -0.93 | 0.68 | -0.03 |
| Q01780 | Exosome component 10 | EXOSC10 | -0.47 | 1.14 | -1.04 | 1.20 | 0.99 | 0.83 | -0.72 | -0.63 | -0.13 | -1.76 | 0.60 | 0.65 |
| Q9BYH1 | Seizure 6-like protein | SEZ6L | -0.47 | 0.43 | -0.46 | -0.69 | 2.35 | 0.09 | -0.71 | -0.54 | -0.66 | 0.96 | -0.70 | 0.37 |
| O75915 | PRA1 family protein 3 | ARL6IP5 | -0.47 | 2.26 | 1.67 | 0.53 | -0.38 | -0.11 | 0.37 | -1.27 | -0.46 | -1.70 | 0.55 | 0.80 |
| O76003 | Glutaredoxin-3 | GLRX3 | -0.47 | 0.80 | 0.29 | 0.13 | 0.11 | 0.85 | -0.39 | -0.24 | -1.50 | -1.65 | 0.99 | 1.41 |
| Q9Y2K2 | Serine/threonine-protein kinase SIK3 | SIK3 | -0.47 | 0.70 | 0.20 | 1.01 | -1.38 | 0.52 | 0.52 | -1.62 | 1.13 | -1.12 | 0.20 | 0.54 |
| Q9Y3L5 | Ras-related protein Rap-2c | RAP2C | -0.47 | 1.03 | 0.12 | 1.94 | -1.15 | 0.79 | -0.51 | -0.72 | 0.55 | -1.44 | 0.33 | 0.08 |
| Q9BZX2 | Uridine-cytidine kinase 2 | UCK2 | -0.47 | 1.22 | 2.76 | -0.30 | -0.60 | -0.19 | -0.28 | -0.22 | -0.24 | -0.83 | -0.05 | -0.05 |
| O43676 | NADH dehydrogenase [ubiquinone] 1 beta subcomplex subunit 3 | NDUFB3 | -0.47 | 0.50 | 2.44 | -0.40 | -0.53 | -0.37 | -0.49 | -0.33 | 1.15 | -0.66 | -0.42 | -0.39 |
| P55769 | NHP2-like protein 1 | SNU13 | -0.47 | 0.54 | -0.77 | 0.57 | -1.04 | 0.74 | 1.18 | -0.89 | -0.83 | -1.10 | 1.26 | 0.87 |
| P30050 | 60S ribosomal protein L12 | RPL12 | -0.47 | 1.44 | -0.29 | 2.17 | 0.14 | -0.31 | -0.19 | -0.62 | 0.83 | -1.39 | 0.54 | -0.87 |
| Q9HA65 | TBC1 domain family member 17 | TBC1D17 | -0.47 | 1.61 | 0.47 | 1.28 | -1.09 | 0.04 | 0.93 | 0.35 | -0.23 | -2.19 | 0.18 | 0.25 |
| P52701 | DNA mismatch repair protein Msh6 | MSH6 | -0.47 | 1.14 | 0.80 | -0.12 | -1.70 | 0.75 | 1.55 | 0.09 | -1.51 | -0.07 | -0.20 | 0.40 |
| A5PKW4 | PH and SEC7 domain-containing protein 1 | PSD | -0.47 | 0.78 | 0.86 | -0.90 | -0.84 | 1.20 | 0.63 | -1.77 | 0.89 | -0.34 | 0.78 | -0.50 |
| Q13825 | Methylglutaconyl-CoA hydratase, mitochondrial | AUH | -0.47 | 0.80 | 1.13 | -0.96 | 1.59 | 0.14 | -0.93 | 1.29 | -0.79 | -0.87 | -0.57 | -0.02 |
| Q8IWV8 | E3 ubiquitin-protein ligase UBR2 | UBR2 | -0.47 | 0.72 | 2.01 | 0.99 | -0.79 | -0.67 | -0.65 | 0.21 | -0.50 | -1.11 | -0.40 | 0.90 |
| P45974 | Ubiquitin carboxyl-terminal hydrolase 5 | USP5 | -0.48 | 1.07 | 1.06 | 0.71 | -1.25 | 0.95 | -0.26 | 0.05 | 0.46 | -2.07 | 0.57 | -0.22 |
| P61006 | Ras-related protein Rab-8A | RAB8A | -0.48 | 1.69 | -0.68 | 0.95 | 0.91 | 1.36 | -0.84 | -1.17 | -1.51 | -0.10 | 0.61 | 0.47 |
| P36954 | DNA-directed RNA polymerase II subunit RPB9 | POLR2I | -0.48 | 0.70 | -0.47 | 0.08 | -1.03 | 1.55 | 0.73 | -0.71 | -0.83 | -1.28 | 1.00 | 0.96 |
| P49368 | T-complex protein 1 subunit gamma | CCT3 | -0.48 | 0.44 | -1.47 | 0.30 | 0.35 | 0.71 | 0.69 | -1.48 | -1.34 | 0.71 | 0.87 | 0.66 |
| Q96ID5 | Immunoglobulin superfamily member 21 | IGSF21 | -0.48 | 0.68 | -0.50 | 1.62 | -0.81 | -0.66 | 1.19 | -0.65 | -0.50 | 1.50 | -0.59 | -0.60 |
| Q7L4I2 | Arginine/serine-rich coiled-coil protein 2 | RSRC2 | -0.48 | 0.83 | 1.86 | 0.42 | -1.14 | 0.06 | -0.20 | 1.27 | -1.48 | -0.30 | -0.14 | -0.35 |
| Q8IYB4 | PEX5-related protein | PEX5L | -0.48 | 2.87 | 0.83 | 0.13 | 1.88 | -0.79 | 0.35 | -1.03 | 0.38 | -1.28 | -0.98 | 0.50 |
| Q9Y5J5 | Pleckstrin homology-like domain family A member 3 | PHLDA3 | -0.48 | 1.59 | 0.31 | -0.38 | -0.25 | 0.66 | 1.31 | 0.24 | -0.22 | -2.41 | 0.79 | -0.05 |
| Q5T1J5 | Putative coiled-coil-helix-coiled-coil-helix domain-containing protei | CHCHD2P9 | -0.48 | 0.49 | -0.37 | -0.42 | 2.33 | -0.40 | -0.51 | -0.42 | -0.46 | -0.71 | -0.38 | 1.34 |
| Q8IV08 | Phospholipase D3 | PLD3 | -0.48 | 0.76 | -0.37 | 0.06 | -0.48 | -0.23 | 1.93 | -1.51 | -0.65 | 1.00 | 0.87 | -0.63 |
| Q2M2I8 | AP2-associated protein kinase 1 | AAK1 | -0.48 | 0.83 | 0.27 | 1.66 | -0.89 | 0.47 | -0.53 | -0.19 | 0.88 | -1.98 | 0.37 | -0.06 |
| Q96A23 | Copine-4 | CPNE4 | -0.48 | 0.65 | 1.31 | 1.27 | -0.17 | -0.57 | -1.04 | 0.71 | 0.63 | -0.45 | 0.06 | -1.77 |
| Q9UPN7 | Serine/threonine-protein phosphatase 6 regulatory subunit 1 | PPP6R1 | -0.48 | 0.89 | 0.97 | 1.37 | -0.99 | 0.53 | -0.83 | 1.00 | 0.68 | -1.34 | -0.60 | -0.80 |
| P36957 | Dihydrolipoyllysine-residue succinyltransferase component of 2-o | DLST | -0.48 | 0.85 | -0.58 | -0.12 | 1.59 | -0.62 | 0.75 | -1.79 | -0.41 | 1.18 | -0.45 | 0.46 |
| P23763 | Vesicle-associated membrane protein 1 | VAMP1 | -0.48 | 1.06 | 1.10 | 1.07 | -0.38 | -0.49 | -0.08 | 0.02 | 1.27 | -2.11 | -0.43 | 0.04 |
| Q9UHD2 | Serine/threonine-protein kinase TBK1 | TBK1 | -0.48 | 0.36 | 1.13 | 0.35 | -1.24 | 0.38 | -0.13 | 1.29 | 1.13 | -1.28 | -0.86 | -0.76 |
| Q49MG5 | Microtubule-associated protein 9 | MAP9 | -0.48 | 2.39 | 1.94 | -0.47 | -0.65 | 1.64 | -0.24 | -0.11 | -0.48 | -1.22 | -0.14 | -0.28 |
| Q86WG3 | Caytaxin | ATCAY | -0.48 | 0.39 | -1.71 | 0.47 | 0.32 | 0.48 | 0.96 | -1.53 | -0.96 | 0.74 | 0.53 | 0.71 |
| Q8TDN6 | Ribosome biogenesis protein BRX1 homolog | BRIX1 | -0.48 | 0.57 | -1.07 | -1.38 | 1.39 | 0.78 | 1.01 | -0.35 | -0.17 | 0.11 | 0.88 | -1.20 |
| O15498 | Synaptobrevin homolog YKT6 | YKT6 | -0.48 | 0.77 | -0.87 | 0.00 | 2.49 | -0.03 | -0.65 | 0.45 | -0.83 | -0.71 | -0.24 | 0.39 |
| Q9BTC0 | Death-inducer obliterator 1 | DIDO1 | -0.48 | 1.28 | 2.78 | -0.25 | -0.52 | -0.25 | -0.31 | -0.28 | -0.23 | -0.77 | -0.19 | 0.01 |
| Q8WZ64 | Arf-GAP with Rho-GAP domain, ANK repeat and PH domain-contai | ARAP2 | -0.48 | 0.57 | 0.56 | -0.91 | -1.17 | 1.24 | 1.00 | 0.63 | -0.08 | -1.23 | 0.99 | -1.04 |
| Q2NL82 | Pre-rRNA-processing protein TSR1 homolog | TSR1 | -0.49 | 0.53 | 0.87 | 0.27 | -1.06 | 1.50 | -0.90 | -0.75 | -0.89 | 0.28 | 1.42 | -0.74 |

| | | | | | | | | | | | | | | |
|---|---|---|---|---|---|---|---|---|---|---|---|---|---|---|
| Q9C0B0 | RING finger protein unkempt homolog | UNK | -0.49 | 1.21 | 0.83 | 0.31 | -0.73 | 0.95 | 0.01 | -1.16 | -1.86 | 0.85 | -0.27 | 1.09 |
| P42356 | Phosphatidylinositol 4-kinase alpha | PI4KA | -0.49 | 0.79 | 1.29 | 0.39 | 0.29 | -0.21 | -0.80 | 1.29 | 0.67 | -1.96 | -0.41 | -0.54 |
| P17480 | Nucleolar transcription factor 1 | UBTF | -0.49 | 0.70 | 0.86 | -1.00 | -0.92 | 0.86 | 1.06 | -0.71 | 0.46 | -0.90 | 1.36 | -1.08 |
| Q8TB37 | Iron-sulfur protein NUBPL | NUBPL | -0.49 | 0.71 | -0.28 | -0.51 | 2.40 | -0.38 | -0.35 | -0.39 | -0.39 | -0.88 | -0.42 | 1.19 |
| P63104 | 14-3-3 protein zeta/delta | YWHAZ | -0.49 | 2.08 | -1.25 | 1.87 | 0.55 | 0.09 | 0.72 | -1.68 | -0.16 | 0.22 | 0.10 | -0.47 |
| Q9BTL3 | RNA guanine-N7 methyltransferase activating subunit | RAMAC | -0.49 | 0.47 | -1.28 | 0.61 | 0.58 | 0.92 | -0.22 | -1.40 | -1.38 | 0.27 | 0.87 | 1.03 |
| Q8TAE6 | Protein phosphatase 1 regulatory subunit 14C | PPP1R14C | -0.49 | 1.78 | 0.33 | 2.65 | -0.66 | -0.33 | -0.15 | -0.06 | -0.26 | -1.05 | -0.24 | -0.22 |
| Q15120 | [Pyruvate dehydrogenase (acetyl-transferring)] kinase isozyme 3, | PDK3 | -0.49 | 1.44 | -0.01 | -0.32 | -0.69 | -0.11 | 2.70 | -0.09 | -0.24 | -1.02 | -0.14 | -0.08 |
| P10114 | Ras-related protein Rap-2a | RAP2A | -0.49 | 1.79 | 0.65 | 1.37 | -1.24 | 0.93 | 0.05 | -0.56 | -0.06 | -1.86 | 0.71 | 0.01 |
| P52594 | Arf-GAP domain and FG repeat-containing protein 1 | AGFG1 | -0.49 | 1.21 | -0.72 | 1.54 | -1.06 | 0.78 | 0.80 | -0.62 | -0.36 | -1.47 | 1.02 | 0.10 |
| Q8IUQ0 | Clavesin-1 | CLVS1 | -0.49 | 0.52 | -1.14 | 1.46 | -1.06 | 1.18 | 0.23 | -0.85 | -0.08 | -0.17 | 1.26 | -0.81 |
| O43236 | Septin-4 | SEPTIN4 | -0.49 | 1.20 | 1.19 | 0.44 | 1.02 | -0.82 | -0.51 | 0.81 | 1.00 | -1.03 | -0.55 | -1.55 |
| Q9H3U1 | Protein unc-45 homolog A | UNC45A | -0.49 | 0.59 | 0.54 | 0.46 | -1.13 | 0.30 | 0.57 | 0.88 | -0.62 | -2.20 | 0.42 | 0.77 |
| P49441 | Inositol polyphosphate 1-phosphatase | INPP1 | -0.49 | 0.61 | -1.09 | 0.16 | 0.35 | 0.93 | 0.43 | -1.19 | -1.80 | 0.27 | 0.94 | 1.00 |
| Q8IV01 | Synaptotagmin-12 | SYT12 | -0.49 | 0.65 | -1.03 | 0.75 | 0.74 | 0.68 | -0.31 | -1.40 | -1.50 | 1.12 | 0.89 | 0.08 |
| Q13614 | Myotubularin-related protein 2 | MTMR2 | -0.49 | 0.91 | 0.59 | 0.00 | -0.05 | 0.86 | -0.30 | 1.19 | 0.21 | -2.51 | 0.20 | -0.19 |
| Q9Y2J8 | Protein-arginine deiminase type-2 | PADI2 | -0.49 | 0.42 | -1.79 | 1.19 | 0.10 | 0.81 | 0.24 | -0.74 | -1.24 | -0.29 | 0.85 | 0.87 |
| Q96KN1 | Protein LRATD2 | LRATD2 | -0.49 | 0.61 | 1.25 | -0.70 | -0.73 | -0.33 | 1.28 | 0.55 | 0.78 | -1.58 | -1.01 | 0.50 |
| P16455 | Methylated-DNA--protein-cysteine methyltransferase | MGMT | -0.49 | 0.53 | 0.17 | 0.79 | -1.24 | 0.16 | 0.81 | 0.37 | -1.15 | -1.49 | 0.03 | 1.56 |
| Q53RE8 | Ankyrin repeat domain-containing protein 39 | ANKRD39 | -0.49 | 1.81 | 0.19 | -0.32 | -0.66 | 0.01 | 2.64 | -0.03 | -0.40 | -1.09 | -0.28 | -0.05 |
| Q8WXF7 | Atlastin-1 | ATL1 | -0.49 | 0.64 | 1.00 | 0.85 | -0.16 | 0.11 | -1.00 | 0.97 | 1.17 | -1.75 | -0.33 | -0.87 |
| Q9H4A5 | Golgi phosphoprotein 3-like | GOLPH3L | -0.49 | 0.77 | -0.48 | 0.08 | 1.37 | 0.99 | -1.04 | 0.08 | -0.92 | -1.32 | -0.23 | 1.47 |
| Q6PI47 | BTB/POZ domain-containing protein KCTD18 | KCTD18 | -0.49 | 0.58 | 0.47 | 1.12 | -1.71 | 0.72 | 0.13 | -1.65 | -0.42 | -0.17 | 0.75 | 0.75 |
| P53779 | Mitogen-activated protein kinase 10 | MAPK10 | -0.50 | 1.07 | 0.54 | 1.20 | -1.13 | 0.53 | 0.07 | 0.01 | 0.88 | -2.21 | 0.20 | -0.08 |
| Q9UBP4 | Dickkopf-related protein 3 | DKK3 | -0.50 | 0.57 | 1.12 | -0.22 | 0.82 | -0.03 | -0.97 | -0.78 | -0.83 | 2.00 | -0.28 | -0.83 |
| Q86V97 | Kelch repeat and BTB domain-containing protein 6 | KBTBD6 | -0.50 | 0.47 | -0.06 | 1.47 | 0.71 | -1.68 | 0.16 | 0.33 | -0.10 | 0.49 | -1.72 | 0.38 |
| Q96M96 | FYVE, RhoGEF and PH domain-containing protein 4 | FGD4 | -0.50 | 0.40 | 1.89 | 0.49 | -1.32 | 0.05 | -0.58 | 0.84 | -0.30 | -1.47 | 0.00 | 0.40 |
| Q9P2E9 | Ribosome-binding protein 1 | RRBP1 | -0.50 | 0.60 | -0.01 | -0.36 | 0.41 | 0.90 | -0.17 | -1.79 | -1.54 | 0.92 | 0.98 | 0.67 |
| A2RUS2 | DENN domain-containing protein 3 | DENND3 | -0.50 | 0.63 | -0.53 | 1.58 | -0.94 | -0.79 | 1.47 | 0.89 | -0.78 | -1.02 | 0.30 | -0.19 |
| Q96EK5 | KIF1-binding protein | KIF1BP | -0.50 | 0.69 | 1.01 | -0.23 | -0.81 | 0.21 | 0.68 | 0.24 | 0.18 | -2.40 | 0.94 | 0.18 |
| Q96JM2 | Zinc finger protein 462 | ZNF462 | -0.50 | 0.99 | -0.04 | -0.38 | -0.54 | -0.37 | 2.49 | 0.89 | -0.23 | -1.01 | -0.45 | -0.37 |
| Q9Y2V7 | Conserved oligomeric Golgi complex subunit 6 | COG6 | -0.50 | 0.58 | -0.03 | 0.53 | -1.14 | 1.07 | 0.32 | 0.12 | -0.90 | -1.86 | 0.74 | 1.16 |
| Q03164 | Histone-lysine N-methyltransferase 2A | KMT2A | -0.50 | 0.87 | 0.21 | -0.50 | -0.72 | 2.46 | -0.40 | -0.53 | -0.49 | -0.90 | 0.79 | 0.09 |
| Q12986 | Transcriptional repressor NF-X1 | NFX1 | -0.50 | 0.58 | -0.98 | 0.50 | 0.41 | 0.52 | 0.29 | -2.28 | 1.40 | -0.14 | 0.05 | 0.23 |
| P61011 | Signal recognition particle 54 kDa protein | SRP54 | -0.50 | 0.75 | -0.49 | -0.60 | 0.89 | 0.87 | 0.24 | 0.40 | -0.30 | -2.31 | 0.19 | 1.10 |
| P18031 | Tyrosine-protein phosphatase non-receptor type 1 | PTPN1 | -0.50 | 0.71 | -0.33 | 1.12 | 0.19 | 0.53 | -0.63 | 0.05 | -0.40 | -2.19 | 0.27 | 1.38 |
| P02778 | C-X-C motif chemokine 10 | CXCL10 | -0.50 | 0.43 | -1.16 | 1.36 | 0.62 | -0.29 | 0.04 | -1.26 | -1.19 | 1.48 | 0.00 | 0.40 |
| Q00013 | 55 kDa erythrocyte membrane protein | MPP1 | -0.50 | 0.83 | 1.13 | 0.86 | -0.62 | 0.38 | -0.76 | 1.23 | -0.97 | -1.70 | 0.68 | -0.24 |
| P27816 | Microtubule-associated protein 4 | MAP4 | -0.50 | 1.03 | 1.95 | 0.04 | 0.65 | -0.50 | -0.97 | 0.79 | 0.39 | -1.58 | -0.35 | -0.42 |
| P46379 | Large proline-rich protein BAG6 | BAG6 | -0.50 | 0.49 | 1.22 | 0.24 | -0.66 | 0.17 | -0.33 | 1.23 | 0.93 | -2.05 | -0.32 | -0.44 |
| Q9UK58 | Cyclin-L1 | CCNL1 | -0.50 | 0.40 | 0.13 | -0.67 | -0.79 | -0.59 | 2.45 | -0.56 | 0.73 | -0.84 | 0.11 | 0.01 |
| Q04837 | Single-stranded DNA-binding protein, mitochondrial | SSBP1 | -0.51 | 1.60 | -0.43 | 0.05 | 2.09 | 0.28 | -0.35 | -1.79 | -0.34 | 0.90 | -0.06 | -0.35 |
| Q9C0A0 | Contactin-associated protein-like 4 | CNTNAP4 | -0.51 | 0.65 | -1.95 | 1.25 | 0.32 | 0.59 | 0.60 | -1.43 | -0.06 | -0.39 | 0.41 | 0.65 |
| P18615 | Negative elongation factor E | NELFE | -0.51 | 1.03 | 1.13 | -0.81 | 0.38 | 0.31 | 0.18 | 1.04 | -0.52 | -2.22 | 0.70 | -0.20 |
| Q96F07 | Cytoplasmic FMR1-interacting protein 2 | CYFIP2 | -0.51 | 0.88 | -0.40 | -0.44 | -0.50 | 0.14 | 2.25 | -0.46 | 1.15 | 0.10 | -0.72 | -1.12 |
| Q8TAC9 | Secretory carrier-associated membrane protein 5 | SCAMP5 | -0.51 | 0.36 | 0.90 | 1.46 | -1.27 | 0.61 | -1.21 | -0.25 | 0.69 | -1.38 | 0.38 | 0.09 |
| Q70CQ4 | Ubiquitin carboxyl-terminal hydrolase 31 | USP31 | -0.51 | 1.05 | 0.09 | 0.65 | -1.00 | 0.96 | 0.51 | 1.24 | -0.89 | -1.78 | 0.78 | -0.56 |
| P51812 | Ribosomal protein S6 kinase alpha-3 | RPS6KA3 | -0.51 | 1.04 | 0.19 | 1.06 | -0.52 | 0.44 | 0.03 | 0.35 | 0.23 | -2.62 | 0.50 | 0.34 |
| P51157 | Ras-related protein Rab-28 | RAB28 | -0.51 | 0.64 | -0.43 | 0.82 | -1.72 | 0.98 | 1.15 | 0.11 | -1.49 | 0.06 | 0.77 | -0.25 |
| Q92556 | Engulfment and cell motility protein 1 | ELMO1 | -0.51 | 1.15 | 0.54 | 0.73 | -0.81 | 0.82 | 0.02 | 0.55 | 0.00 | -2.17 | 1.11 | -0.79 |
| Q07666 | KH domain-containing, RNA-binding, signal transduction-associate | KHDRBS1 | -0.51 | 1.21 | 1.53 | -0.85 | -1.23 | 0.48 | 1.43 | -0.26 | -0.68 | -1.07 | 0.69 | -0.03 |
| P19784 | Casein kinase II subunit alpha' | CSNK2A2 | -0.51 | 0.63 | 0.03 | 1.79 | -0.23 | 0.23 | -1.02 | 0.86 | -0.14 | -1.95 | 0.17 | 0.28 |
| P07954 | Fumarate hydratase, mitochondrial | FH | -0.51 | 0.75 | -0.96 | 1.14 | 0.81 | 0.91 | -0.98 | -0.55 | -0.64 | 0.21 | -1.31 | 1.37 |
| A0A0J9YX94 | Paraneoplastic antigen Ma6F | PNMA6F | -0.51 | 0.80 | -0.22 | 2.57 | -0.65 | -0.36 | -0.36 | -0.31 | -0.42 | -0.82 | -0.23 | 0.80 |
| Q13485 | Mothers against decapentaplegic homolog 4 | SMAD4 | -0.51 | 0.62 | -1.60 | 0.70 | 1.06 | 0.74 | -0.12 | -0.39 | -1.84 | 0.52 | 0.56 | 0.38 |
| Q96QR8 | Transcriptional activator protein Pur-beta | PURB | -0.51 | 3.63 | 0.94 | 1.05 | -0.72 | 0.90 | 0.58 | -1.31 | 0.45 | -1.78 | 0.30 | -0.43 |
| Q9NRR3 | CDC42 small effector protein 2 | CDC42SE2 | -0.51 | 0.45 | 0.80 | 1.49 | -1.10 | 0.48 | -1.07 | 0.40 | 0.71 | -1.26 | 0.55 | -0.99 |
| Q96T51 | RUN and FYVE domain-containing protein 1 | RUFY1 | -0.51 | 0.75 | 0.12 | -0.21 | 0.55 | 0.18 | 0.31 | -0.08 | -2.29 | -0.57 | 0.32 | 1.68 |
| Q5T1M5 | FK506-binding protein 15 | FKBP15 | -0.51 | 0.78 | -0.24 | 1.12 | -0.62 | 0.52 | 0.18 | 0.01 | 0.36 | -2.42 | 0.10 | 0.99 |
| P19367 | Hexokinase-1 | HK1 | -0.51 | 0.54 | 1.09 | 0.97 | -1.56 | -0.76 | 0.96 | 0.72 | 0.70 | -0.16 | -0.86 | -1.10 |
| P60953 | Cell division control protein 42 homolog | CDC42 | -0.51 | 1.18 | 0.08 | 1.10 | -0.33 | 0.59 | -0.11 | -1.25 | -1.65 | 1.65 | 0.35 | -0.44 |
| Q17RD7 | Synaptotagmin-16 | SYT16 | -0.51 | 1.11 | 2.79 | -0.33 | -0.60 | -0.22 | -0.33 | -0.30 | -0.03 | -0.67 | -0.07 | -0.23 |
| Q9Y6Y8 | SEC23-interacting protein | SEC23IP | -0.51 | 0.75 | 0.53 | 0.46 | -0.24 | 0.60 | -0.43 | -0.89 | 1.32 | -2.24 | 0.46 | 0.42 |
| Q5TH69 | Brefeldin A-inhibited guanine nucleotide-exchange protein 3 | ARFGEF3 | -0.51 | 0.87 | 0.95 | 0.78 | -0.48 | 0.26 | -0.46 | 0.89 | 1.03 | -1.99 | 0.10 | -1.08 |
| Q02556 | Interferon regulatory factor 8 | IRF8 | -0.51 | 1.01 | 0.42 | 0.82 | -0.92 | -0.65 | 1.49 | -0.52 | -0.63 | -0.94 | -0.68 | 1.62 |
| Q08495 | Dematin | DMTN | -0.51 | 0.86 | 0.39 | 0.32 | -1.92 | 0.74 | 1.51 | -0.56 | 0.32 | -1.25 | 0.51 | -0.05 |
| Q07002 | Cyclin-dependent kinase 18 | CDK18 | -0.51 | 0.99 | 0.86 | -0.38 | -0.81 | 0.95 | 0.54 | -0.57 | 1.36 | -1.63 | 0.69 | -0.99 |
| Q9NUJ1 | Mycophenolic acid acyl-glucuronide esterase, mitochondrial | ABHD10 | -0.51 | 0.34 | -0.78 | -1.32 | 0.39 | 1.17 | 1.00 | -0.74 | -1.34 | -0.20 | 1.10 | 0.71 |
| Q9UKB3 | DnaJ homolog subfamily C member 12 | DNAJC12 | -0.51 | 0.93 | -0.46 | 1.73 | 0.48 | -0.82 | 0.15 | -0.89 | -0.76 | -1.21 | 0.41 | 1.37 |
| Q9BV23 | Monoacylglycerol lipase ABHD6 | ABHD6 | -0.52 | 0.95 | 0.38 | 0.44 | -1.04 | 1.25 | 0.09 | -0.73 | -1.55 | -0.90 | 1.28 | 0.80 |
| Q99798 | Aconitate hydratase, mitochondrial | ACO2 | -0.52 | 2.14 | -0.70 | 1.27 | 1.75 | -0.06 | -0.26 | -1.56 | -0.36 | 0.72 | 0.02 | -0.83 |
| Q9NRA8 | Eukaryotic translation initiation factor 4E transporter | EIF4ENIF1 | -0.52 | 1.20 | 1.12 | -0.12 | -0.47 | 0.69 | 0.12 | 1.29 | 0.97 | -1.00 | -1.43 | -1.17 |
| Q8IWU2 | Serine/threonine-protein kinase LMTK2 | LMTK2 | -0.52 | 0.61 | 1.38 | -0.93 | -1.47 | 0.46 | 1.33 | 0.12 | -1.25 | 0.58 | 0.15 | -0.38 |
| P09132 | Signal recognition particle 19 kDa protein | SRP19 | -0.52 | 0.92 | 0.41 | -0.73 | 0.95 | -0.99 | 1.44 | -0.85 | -1.68 | 0.23 | 0.43 | 0.78 |
| Q96FZ2 | Abasic site processing protein HMCES | HMCES | -0.52 | 1.49 | 2.75 | -0.27 | -0.57 | 0.02 | -0.31 | -0.15 | -0.27 | -0.89 | -0.16 | -0.16 |
| Q9P0L1 | Zinc finger protein with KRAB and SCAN domains 7 | ZKSCAN7 | -0.52 | 0.71 | 0.80 | -1.38 | 0.60 | 0.45 | 0.41 | -0.65 | -0.20 | -1.18 | 1.85 | -0.69 |
| Q8IZH2 | 5'-3' exoribonuclease 1 | XRN1 | -0.52 | 0.61 | 1.89 | -1.40 | -0.15 | 0.24 | 0.18 | 0.23 | -0.05 | -1.69 | 0.07 | 0.66 |
| Q8IXS6 | Paralemmin-2 | PALM2 | -0.52 | 0.71 | 1.22 | 1.15 | -0.30 | 0.40 | -1.60 | 0.38 | 0.75 | -1.57 | -0.03 | -0.41 |
| P54646 | 5'-AMP-activated protein kinase catalytic subunit alpha-2 | PRKAA2 | -0.52 | 1.38 | -0.13 | -0.96 | 0.27 | 1.00 | 1.30 | -1.29 | -0.92 | 0.54 | 1.22 | -1.02 |
| O96019 | Actin-like protein 6A | ACTL6A | -0.52 | 0.84 | -0.42 | 1.37 | -1.58 | 0.51 | 1.13 | 0.09 | -1.36 | -0.73 | 0.60 | 0.41 |
| Q13151 | Heterogeneous nuclear ribonucleoprotein A0 | HNRNPA0 | -0.52 | 0.62 | 1.17 | -1.46 | -1.12 | 0.80 | 1.40 | -0.07 | -1.01 | -0.34 | 0.71 | -0.07 |
| Q4G0F5 | Vacuolar protein sorting-associated protein 26B | VPS26B | -0.52 | 2.00 | 0.90 | 1.15 | -0.48 | 0.60 | -0.24 | -1.21 | 0.21 | -2.01 | 0.76 | 0.31 |
| Q15029 | 116 kDa U5 small nuclear ribonucleoprotein component | EFTUD2 | -0.52 | 0.80 | 0.55 | 0.33 | 0.31 | 1.12 | -1.36 | 0.46 | -1.29 | -1.57 | 0.77 | 0.66 |

| | | | | | | | | | | | | | | |
|---|---|---|---|---|---|---|---|---|---|---|---|---|---|---|
| Q8NCG7 | Sn1-specific diacylglycerol lipase beta | DAGLB | -0.52 | 1.67 | -0.02 | 2.77 | -0.47 | -0.18 | -0.33 | -0.28 | -0.11 | -0.84 | -0.19 | -0.35 |
| O95372 | Acyl-protein thioesterase 2 | LYPLA2 | -0.52 | 0.64 | -1.08 | 1.47 | 0.20 | 0.69 | -0.48 | -1.13 | -1.20 | -0.42 | 0.72 | 1.23 |
| Q9P2S2 | Neurexin-2 | NRXN2 | -0.52 | 1.31 | -0.43 | 1.38 | -0.90 | -0.45 | 1.82 | -0.54 | -0.63 | 1.03 | -0.63 | -0.65 |
| Q86VP1 | Tax1-binding protein 1 | TAX1BP1 | -0.52 | 1.11 | -0.33 | -0.82 | 0.59 | 1.04 | 0.77 | -1.66 | -1.06 | 0.74 | 1.23 | -0.51 |
| Q7Z5K2 | Wings apart-like protein homolog | WAPL | -0.52 | 0.45 | 0.27 | -1.48 | 1.73 | -0.13 | 0.20 | 0.53 | -0.06 | 0.73 | -0.11 | -1.69 |
| Q5R3F8 | Protein phosphatase 1 regulatory subunit 29 | ELFN2 | -0.52 | 0.83 | 1.59 | 0.20 | -0.76 | 0.49 | -0.53 | -0.62 | 1.79 | -1.04 | -0.69 | -0.43 |
| P78362 | SRSF protein kinase 2 | SRPK2 | -0.52 | 1.06 | 1.04 | 0.82 | -1.13 | -0.03 | 0.51 | -2.21 | 0.30 | 0.84 | -0.21 | 0.07 |
| Q01581 | Hydroxymethylglutaryl-CoA synthase, cytoplasmic | HMGCS1 | -0.52 | 0.59 | -1.26 | 0.43 | 0.50 | 0.97 | 0.13 | -1.85 | -1.00 | 0.47 | 0.97 | 0.66 |
| P84101 | Small EDRK-rich factor 2 | SERF2 | -0.53 | 0.84 | -0.84 | -0.11 | 1.93 | 0.47 | -0.45 | -1.15 | -1.00 | -0.54 | 0.65 | 1.05 |
| Q9NW68 | BSD domain-containing protein 1 | BSDC1 | -0.53 | 1.07 | 0.07 | 1.92 | -1.05 | 1.02 | -0.74 | -0.79 | -0.06 | -1.22 | 0.67 | 0.18 |
| Q9H2U1 | ATP-dependent DNA/RNA helicase DHX36 | DHX36 | -0.53 | 1.62 | -0.74 | 1.92 | 0.60 | 0.72 | -0.84 | 0.36 | -1.26 | -1.11 | 0.48 | -0.13 |
| O14647 | Chromodomain-helicase-DNA-binding protein 2 | CHD2 | -0.53 | 1.38 | 0.75 | 1.56 | -1.39 | 0.11 | 0.44 | -0.76 | 0.10 | -1.58 | -0.10 | 0.87 |
| P04216 | Thy-1 membrane glycoprotein | THY1 | -0.53 | 1.56 | -0.12 | 0.64 | -0.02 | 0.79 | 0.38 | -1.63 | -1.93 | 0.77 | 0.87 | 0.25 |
| O00217 | NADH dehydrogenase [ubiquinone] iron-sulfur protein 8, mitochon | NDUFS8 | -0.53 | 1.36 | 2.77 | -0.27 | -0.60 | -0.07 | -0.31 | -0.10 | -0.27 | -0.79 | -0.19 | -0.16 |
| Q08722 | Leukocyte surface antigen CD47 | CD47 | -0.53 | 0.50 | -1.04 | 0.64 | -0.21 | 0.88 | 0.38 | -2.37 | 0.39 | 0.17 | 0.75 | 0.41 |
| P29350 | Tyrosine-protein phosphatase non-receptor type 6 | PTPN6 | -0.53 | 0.37 | -1.06 | 0.27 | 0.61 | 0.61 | 0.07 | -1.59 | -1.50 | 0.84 | 0.77 | 0.98 |
| Q15036 | Sorting nexin-17 | SNX17 | -0.53 | 1.19 | 0.99 | 0.72 | -1.18 | 0.48 | 0.30 | 1.01 | -1.81 | -1.10 | 0.51 | 0.07 |
| Q5T011 | KICSTOR complex protein SZT2 | SZT2 | -0.53 | 0.58 | 0.30 | -0.85 | -1.01 | 0.97 | 1.33 | -0.81 | 1.11 | -1.21 | 0.83 | -0.66 |
| Q9UIS9 | Methyl-CpG-binding domain protein 1 | MBD1 | -0.53 | 0.62 | 0.85 | -1.04 | -1.14 | 0.35 | 1.76 | -0.72 | -1.00 | 0.36 | 0.95 | -0.37 |
| O15042 | U2 snRNP-associated SURP motif-containing protein | U2SURP | -0.53 | 0.63 | 0.59 | 1.03 | -2.31 | 0.76 | 0.74 | 0.01 | 0.19 | -0.90 | 0.32 | -0.42 |
| Q86TX2 | Acyl-coenzyme A thioesterase 1 | ACOT1 | -0.53 | 1.56 | -0.96 | -1.04 | 1.01 | 0.68 | 1.96 | -0.67 | -0.79 | -0.62 | 0.01 | 0.43 |
| O43933 | Peroxisome biogenesis factor 1 | PEX1 | -0.53 | 1.96 | 0.36 | 2.60 | -0.50 | -0.26 | -0.25 | -0.15 | -0.23 | -1.23 | -0.39 | 0.05 |
| Q9NZ45 | CDGSH iron-sulfur domain-containing protein 1 | CISD1 | -0.53 | 1.52 | 0.65 | 0.65 | 0.33 | 0.62 | -0.63 | -1.14 | -0.26 | -2.03 | 0.68 | 1.13 |
| A5YM72 | Carnosine synthase 1 | CARNS1 | -0.53 | 0.70 | 0.16 | -0.58 | 0.31 | 1.40 | -0.41 | 0.09 | -0.87 | 0.16 | 1.55 | -1.80 |
| Q9NW82 | WD repeat-containing protein 70 | WDR70 | -0.53 | 0.56 | -0.20 | 0.25 | -0.96 | 0.65 | 0.98 | -1.12 | -1.18 | -0.85 | 0.85 | 1.58 |
| Q92752 | Tenascin-R | TNR | -0.53 | 1.25 | -0.12 | -0.78 | 0.17 | 0.86 | 1.24 | -1.30 | -1.35 | 0.81 | 1.18 | -0.71 |
| Q9Y2I7 | 1-phosphatidylinositol 3-phosphate 5-kinase | PIKFYVE | -0.53 | 0.72 | 0.10 | 1.48 | 0.55 | 0.63 | -1.87 | -0.52 | -1.05 | 0.43 | 0.83 | -0.58 |
| O94818 | Nucleolar protein 4 | NOL4 | -0.53 | 0.87 | -0.95 | 0.69 | -0.72 | 1.25 | 0.77 | -1.31 | -1.11 | -0.48 | 1.06 | 0.80 |
| O75335 | Liprin-alpha-4 | PPFIA4 | -0.53 | 0.82 | 0.63 | 1.45 | -0.86 | 0.34 | -0.58 | 1.09 | -0.01 | -2.03 | -0.08 | 0.03 |
| Q16778 | Histone H2B type 2-E | HIST2H2BE | -0.54 | 0.79 | 0.63 | -0.84 | 1.81 | -0.40 | -0.26 | 0.14 | -1.99 | 0.17 | 0.13 | 0.60 |
| Q9BUH6 | Protein PAXX | PAXX | -0.54 | 1.44 | -0.06 | -0.30 | -0.63 | 2.75 | -0.17 | -0.27 | -0.07 | -0.89 | -0.17 | -0.18 |
| Q9P0M6 | Core histone macro-H2A.2 | H2AFY2 | -0.54 | 0.88 | 0.66 | -0.85 | 1.41 | -0.52 | 0.35 | 0.66 | -0.52 | -2.09 | 0.46 | 0.44 |
| P62070 | Ras-related protein R-Ras2 | RRAS2 | -0.54 | 0.89 | -0.95 | 0.33 | -0.71 | 0.86 | 1.53 | 0.56 | -0.89 | -1.65 | 0.74 | 0.21 |
| Q7L7X3 | Serine/threonine-protein kinase TAO1 | TAOK1 | -0.54 | 0.65 | -0.08 | 0.03 | -0.72 | 0.73 | 0.86 | -0.16 | 0.87 | -2.40 | 0.81 | 0.06 |
| Q9H270 | Vacuolar protein sorting-associated protein 11 homolog | VPS11 | -0.54 | 1.07 | -1.81 | 0.37 | 1.21 | 1.09 | 0.35 | -1.04 | -0.75 | -0.01 | 1.00 | -0.41 |
| Q3KR37 | Protein Aster-B | GRAMD1B | -0.54 | 0.37 | -0.97 | 0.70 | 0.09 | 0.62 | 0.07 | -1.06 | -1.87 | 1.42 | 0.67 | 0.34 |
| O00468 | Agrin | AGRN | -0.54 | 0.78 | -0.20 | 1.04 | 1.62 | -0.32 | -1.19 | 0.17 | -1.76 | 0.42 | -0.33 | 0.55 |
| Q96ST3 | Paired amphipathic helix protein Sin3a | SIN3A | -0.54 | 0.72 | 0.90 | 0.25 | 0.03 | 0.57 | -0.86 | 0.43 | 0.55 | -2.52 | 0.38 | 0.27 |
| Q9UKA9 | Polypyrimidine tract-binding protein 2 | PTBP2 | -0.54 | 1.68 | 1.08 | -0.25 | 0.26 | 1.21 | -0.59 | 0.00 | -0.38 | -2.24 | 0.85 | 0.06 |
| P13073 | Cytochrome c oxidase subunit 4 isoform 1, mitochondrial | COX4I1 | -0.54 | 1.59 | 0.43 | 0.37 | 0.03 | 0.47 | 0.42 | 0.51 | 0.44 | -0.32 | -2.75 | 0.40 |
| Q9P016 | Thymocyte nuclear protein 1 | THYN1 | -0.54 | 2.71 | 1.32 | 0.36 | -1.15 | 0.00 | 1.86 | -0.57 | -0.98 | -0.24 | 0.34 | -0.94 |
| Q86WJ1 | Chromodomain-helicase-DNA-binding protein 1-like | CHD1L | -0.54 | 0.74 | 0.46 | -1.82 | 0.54 | 1.00 | 0.72 | 0.14 | 0.15 | -0.23 | 0.76 | -1.73 |
| Q04323 | UBX domain-containing protein 1 | UBXN1 | -0.54 | 1.90 | -0.02 | 2.14 | -0.63 | 0.62 | -0.26 | -0.23 | -1.28 | -1.21 | 0.59 | 0.28 |
| Q9H4L7 | SWI/SNF-related matrix-associated actin-dependent regulator of c | SMARCAD1 | -0.54 | 0.84 | 2.55 | -0.43 | -0.54 | -0.24 | -0.33 | -0.42 | 0.89 | -0.82 | -0.29 | -0.37 |
| Q96BJ3 | Axin interactor, dorsalization-associated protein | AIDA | -0.54 | 2.18 | 0.99 | -0.41 | 0.10 | -0.17 | 1.51 | 0.13 | -2.28 | -0.38 | 0.09 | 0.41 |
| O14578 | Citron Rho-interacting kinase | CIT | -0.54 | 0.52 | 1.18 | 0.34 | -0.78 | -0.10 | 0.02 | 1.14 | 0.83 | -2.18 | -0.21 | -0.26 |
| P49802 | Regulator of G-protein signaling 7 | RGS7 | -0.54 | 1.29 | 0.52 | 1.68 | -0.90 | 0.04 | 0.05 | 0.14 | 0.87 | -2.01 | 0.12 | -0.53 |
| Q9H477 | Ribokinase | RBKS | -0.54 | 1.06 | 1.38 | -0.83 | -1.18 | 0.77 | 1.08 | -0.85 | -0.76 | 0.70 | -0.99 | 0.70 |
| P11233 | Ras-related protein Ral-A | RALA | -0.54 | 3.54 | 0.21 | 0.80 | 1.20 | 0.22 | 0.42 | -2.57 | 0.00 | -0.03 | -0.19 | -0.06 |
| A6NHX0 | Cytosolic arginine sensor for mTORC1 subunit 2 | CASTOR2 | -0.55 | 1.31 | -0.15 | 0.43 | 0.35 | 0.69 | 0.16 | -2.39 | -0.93 | 0.34 | 1.15 | 0.36 |
| P10451 | Osteopontin | SPP1 | -0.55 | 0.44 | 0.19 | 1.48 | 0.17 | -0.58 | -0.68 | -0.70 | -0.68 | 2.05 | -0.64 | -0.62 |
| Q96P16 | Regulation of nuclear pre-mRNA domain-containing protein 1A | RPRD1A | -0.55 | 0.65 | 1.56 | -0.83 | -1.02 | 0.62 | 0.49 | -0.18 | 1.49 | -1.26 | -0.48 | -0.39 |
| Q12905 | Interleukin enhancer-binding factor 2 | ILF2 | -0.55 | 0.97 | -0.99 | 1.03 | 0.34 | 0.69 | 0.06 | -1.88 | -0.82 | 1.31 | 0.57 | -0.31 |
| Q8N142 | Adenylosuccinate synthetase isozyme 1 | ADSSL1 | -0.55 | 0.50 | -1.61 | 0.44 | -0.27 | 0.90 | 1.19 | -0.31 | -1.70 | 0.43 | 0.88 | 0.05 |
| Q9H7Z6 | Histone acetyltransferase KAT8 | KAT8 | -0.55 | 0.70 | 0.86 | -0.87 | 0.58 | 0.11 | 0.20 | -0.98 | 1.83 | -1.34 | 0.49 | -0.88 |
| Q8TDI0 | Chromodomain-helicase-DNA-binding protein 5 | CHD5 | -0.55 | 1.99 | 0.33 | -0.36 | 2.62 | -0.10 | -0.52 | -0.21 | -0.29 | -1.17 | -0.34 | 0.03 |
| Q96NT1 | Nucleosome assembly protein 1-like 5 | NAP1L5 | -0.55 | 0.54 | 1.18 | 0.68 | -1.57 | 0.11 | 0.29 | -0.17 | 1.36 | -1.63 | -0.11 | -0.15 |
| Q9UJ68 | Mitochondrial peptide methionine sulfoxide reductase | MSRA | -0.55 | 1.02 | -0.83 | 0.45 | 0.39 | 0.68 | 0.50 | -0.10 | -2.51 | 0.81 | 0.49 | 0.11 |
| Q5QP82 | DDB1- and CUL4-associated factor 10 | DCAF10 | -0.55 | 0.80 | -0.75 | -0.96 | 0.64 | 1.32 | 0.70 | -0.78 | 1.61 | 0.01 | -1.01 | -0.78 |
| Q01432 | AMP deaminase 3 | AMPD3 | -0.55 | 1.00 | -0.18 | 0.47 | -0.28 | 0.94 | 0.23 | -2.02 | -1.32 | 1.19 | 0.57 | 0.40 |
| Q9UBL3 | Set1/Ash2 histone methyltransferase complex subunit ASH2 | ASH2L | -0.55 | 0.77 | 0.88 | 0.71 | -0.68 | 0.74 | -0.71 | 0.46 | -2.25 | -0.29 | 0.88 | 0.26 |
| Q86VW2 | Rho guanine nucleotide exchange factor 25 | ARHGEF25 | -0.55 | 1.20 | 2.76 | -0.34 | -0.57 | -0.12 | -0.35 | -0.13 | -0.17 | -0.86 | -0.13 | -0.08 |
| Q9NXN4 | Ganglioside-induced differentiation-associated protein 2 | GDAP2 | -0.55 | 0.58 | -0.22 | 0.98 | -1.16 | 0.74 | 0.40 | 0.45 | -1.51 | -1.34 | 0.46 | 1.20 |
| Q13356 | RING-type E3 ubiquitin-protein ligase PPIL2 | PPIL2 | -0.55 | 1.14 | -1.63 | 0.42 | 1.05 | 0.12 | 1.32 | 1.15 | -0.80 | -1.06 | -0.09 | -0.47 |
| Q9NSE4 | Isoleucine--tRNA ligase, mitochondrial | IARS2 | -0.55 | 0.71 | -1.68 | 0.43 | 1.82 | 0.38 | -0.07 | -1.43 | -0.36 | 0.08 | 0.33 | 0.50 |
| P16104 | Histone H2AX | H2AFX | -0.55 | 0.92 | 1.03 | -1.81 | 1.95 | -0.18 | 0.12 | 0.30 | -0.34 | -0.63 | -0.47 | 0.04 |
| Q9BTV5 | Fibronectin type III and SPRY domain-containing protein 1 | FSD1 | -0.55 | 1.16 | -0.38 | 1.69 | 0.29 | -0.06 | -0.24 | 0.22 | 0.48 | -2.33 | 0.42 | -0.09 |
| Q8N1A0 | Keratin-like protein KRT222 | KRT222 | -0.55 | 0.95 | 1.60 | -0.50 | -0.77 | -0.53 | 1.30 | -0.50 | -0.44 | -0.88 | -0.68 | 1.40 |
| P35568 | Insulin receptor substrate 1 | IRS1 | -0.56 | 1.71 | 0.12 | 2.70 | -0.53 | -0.10 | -0.40 | -0.26 | -0.34 | -1.02 | -0.06 | -0.10 |
| Q8NEB9 | Phosphatidylinositol 3-kinase catalytic subunit type 3 | PIK3C3 | -0.56 | 0.53 | 0.11 | 0.47 | -1.47 | 0.48 | 1.09 | 0.30 | 0.40 | -2.16 | 0.36 | 0.42 |
| Q9BZK7 | F-box-like/WD repeat-containing protein TBL1XR1 | TBL1XR1 | -0.56 | 0.79 | 1.78 | -1.25 | -0.05 | 0.91 | -0.43 | 0.72 | -0.26 | -1.25 | 0.67 | -0.83 |
| Q96P48 | Arf-GAP with Rho-GAP domain, ANK repeat and PH domain-contai | ARAP1 | -0.56 | 0.64 | 0.32 | 0.49 | -0.22 | 0.33 | -0.11 | 0.79 | 0.13 | -2.71 | 0.53 | 0.44 |
| Q8NB66 | Protein unc-13 homolog C | UNC13C | -0.56 | 1.12 | -0.04 | -0.25 | -0.73 | -0.25 | 2.56 | -0.40 | -0.35 | -0.85 | 0.77 | -0.45 |
| P12694 | 2-oxoisovalerate dehydrogenase subunit alpha, mitochondrial | BCKDHA | -0.56 | 1.39 | -0.41 | -0.60 | 1.36 | 0.35 | 0.77 | -1.97 | -1.00 | 0.10 | 0.52 | 0.87 |
| O60508 | Pre-mRNA-processing factor 17 | CDC40 | -0.56 | 0.68 | 0.94 | -0.95 | -1.04 | 0.71 | 1.18 | 0.49 | -0.46 | -1.21 | 1.24 | -0.89 |
| O43678 | NADH dehydrogenase [ubiquinone] 1 alpha subcomplex subunit 2 | NDUFA2 | -0.56 | 1.07 | 1.62 | 1.29 | -0.83 | -0.37 | -0.49 | -0.56 | -0.43 | -0.97 | -0.62 | 1.36 |
| Q9NU22 | Midasin | MDN1 | -0.56 | 0.82 | 0.88 | 0.20 | 1.39 | -0.61 | -0.87 | -0.69 | 1.36 | -1.64 | -0.23 | 0.21 |
| O14775 | Guanine nucleotide-binding protein subunit beta-5 | GNB5 | -0.56 | 2.28 | 0.03 | 1.41 | -0.26 | 0.79 | 0.14 | -0.09 | 0.15 | -2.48 | 0.32 | -0.02 |
| Q6GQQ9 | OTU domain-containing protein 7B | OTUD7B | -0.56 | 1.27 | -0.99 | 0.54 | 1.44 | 1.11 | -0.72 | -0.96 | -1.21 | -0.33 | 1.18 | -0.06 |
| P52630 | Signal transducer and activator of transcription 2 | STAT2 | -0.56 | 0.63 | 1.14 | 0.23 | -1.71 | 0.87 | 0.25 | 0.46 | -0.18 | -1.83 | 0.34 | 0.42 |
| Q9UHV5 | Rap guanine nucleotide exchange factor-like 1 | RAPGEFL1 | -0.56 | 1.70 | 2.75 | -0.07 | -0.63 | -0.02 | -0.24 | -0.12 | -0.29 | -0.86 | -0.26 | -0.27 |
| Q13017 | Rho GTPase-activating protein 5 | ARHGAP5 | -0.56 | 1.20 | -0.33 | 1.35 | -0.87 | 0.96 | 0.21 | -0.27 | -1.68 | -1.04 | 0.92 | 0.74 |
| Q96RS6 | NudC domain-containing protein 1 | NUDCD1 | -0.56 | 0.64 | 0.27 | -0.17 | -1.28 | 1.37 | 0.61 | -0.52 | 0.71 | -0.30 | 1.05 | -1.74 |

| | | | | | | | | | | | | | | |
|---|---|---|---|---|---|---|---|---|---|---|---|---|---|---|
| O00178 | GTP-binding protein 1 | GTPBP1 | -0.56 | 0.53 | -1.19 | -0.04 | 0.66 | 0.38 | 0.88 | -1.04 | -1.77 | 1.23 | 0.64 | 0.26 |
| Q9BY77 | Polymerase delta-interacting protein 3 | POLDIP3 | -0.56 | 1.01 | -0.77 | 0.30 | 0.26 | 0.79 | 0.60 | -1.01 | 0.13 | -2.13 | 0.98 | 0.85 |
| P62891 | 60S ribosomal protein L39 | RPL39 | -0.56 | 0.63 | -0.29 | -1.69 | 0.88 | 0.11 | 1.79 | -1.24 | 0.45 | 0.02 | 0.39 | -0.42 |
| Q96L91 | E1A-binding protein p400 | EP400 | -0.56 | 0.48 | -1.15 | 0.65 | 0.26 | 0.68 | 0.19 | -1.97 | -0.99 | 0.98 | 0.64 | 0.71 |
| P41219 | Peripherin | PRPH | -0.56 | 1.29 | 0.62 | 0.36 | 1.65 | -0.27 | -0.97 | -0.23 | 0.75 | -1.98 | -0.29 | 0.35 |
| Q6KC79 | Nipped-B-like protein | NIPBL | -0.56 | 0.75 | 1.01 | 0.46 | -0.53 | -0.09 | 0.09 | -2.37 | 1.36 | 0.03 | 0.11 | -0.06 |
| Q6P3W7 | SCY1-like protein 2 | SCYL2 | -0.56 | 0.85 | -0.65 | -0.28 | -0.10 | 1.07 | 0.97 | -0.58 | -1.48 | -1.11 | 0.69 | 1.47 |
| P22415 | Upstream stimulatory factor 1 | USF1 | -0.56 | 0.77 | 2.57 | -0.40 | -0.52 | -0.29 | -0.41 | 0.86 | -0.39 | -0.78 | -0.40 | -0.23 |
| Q9UHG2 | ProSAAS | PCSK1N | -0.57 | 0.67 | 0.43 | 0.31 | -1.02 | -0.60 | 1.72 | -0.61 | -1.12 | -0.01 | 1.53 | -0.63 |
| P11169 | Solute carrier family 2, facilitated glucose transporter member 3 | SLC2A3 | -0.57 | 0.79 | -0.23 | 2.05 | -0.08 | 0.08 | -0.87 | -0.74 | -0.46 | -1.29 | 1.18 | 0.37 |
| Q9Y2W3 | Proton-associated sugar transporter A | SLC45A1 | -0.57 | 0.90 | -0.30 | 2.13 | -0.84 | 0.64 | -0.57 | -0.60 | -0.54 | -1.07 | 1.08 | 0.06 |
| Q6F5E8 | Capping protein, Arp2/3 and myosin-I linker protein 2 | CARMIL2 | -0.57 | 0.86 | 1.27 | 1.45 | 0.41 | -1.74 | -0.35 | -0.54 | 0.48 | -0.54 | -0.95 | 0.51 |
| Q99250 | Sodium channel protein type 2 subunit alpha | SCN2A | -0.57 | 0.94 | 0.66 | 1.42 | -0.20 | -0.11 | -0.67 | 1.32 | 0.64 | -1.72 | -0.52 | -0.81 |
| Q9UMZ2 | Synergin gamma | SYNRG | -0.57 | 0.66 | -1.07 | 1.14 | -0.47 | 0.37 | 0.85 | -0.44 | 0.26 | -1.96 | 0.15 | 1.16 |
| O60260 | E3 ubiquitin-protein ligase parkin | PRKN | -0.57 | 0.82 | 1.80 | -0.13 | -1.54 | 0.54 | 0.31 | 0.39 | -1.17 | 0.17 | 0.64 | -1.01 |
| Q14CZ8 | Hepatocyte cell adhesion molecule | HEPACAM | -0.57 | 1.35 | -0.95 | 1.21 | 0.55 | -0.53 | 1.17 | -0.40 | -1.82 | -0.56 | 0.75 | 0.59 |
| P17706 | Tyrosine-protein phosphatase non-receptor type 2 | PTPN2 | -0.57 | 1.02 | -0.34 | 2.25 | 0.11 | -0.54 | -0.31 | -0.64 | -0.72 | -1.18 | 0.38 | 0.98 |
| Q96LR2 | Leucine rich adaptor protein 1 | LURAP1 | -0.57 | 1.16 | -1.08 | 0.20 | 0.54 | 1.64 | -0.01 | -1.16 | -1.42 | 0.88 | -0.34 | 0.75 |
| Q99961 | Endophilin-A2 | SH3GL1 | -0.57 | 0.97 | 2.13 | 0.63 | -1.19 | 0.04 | -0.48 | -0.34 | 0.72 | -1.28 | -0.39 | 0.16 |
| P27449 | V-type proton ATPase 16 kDa proteolipid subunit | ATP6V0C | -0.57 | 0.89 | 0.87 | 1.34 | -0.26 | 0.52 | -1.43 | -1.44 | 1.19 | -0.45 | 0.22 | -0.57 |
| Q8WZ42 | Titin | TTN | -0.57 | 0.51 | -2.00 | 0.75 | 0.24 | 0.70 | 0.97 | -1.32 | -0.66 | 0.11 | 0.64 | 0.58 |
| Q01844 | RNA-binding protein EWS | EWSR1 | -0.57 | 0.84 | 0.63 | -0.36 | -0.74 | -0.63 | 2.11 | -1.37 | -0.31 | 1.01 | -0.07 | -0.27 |
| Q9NZU7 | Calcium-binding protein 1 | CABP1 | -0.57 | 1.43 | 1.20 | -0.60 | -0.11 | -0.46 | 1.48 | 0.07 | -1.88 | -0.41 | 1.00 | -0.28 |
| Q9H6B1 | Zinc finger protein 385D | ZNF385D | -0.57 | 1.80 | 0.17 | -0.19 | -0.59 | -0.21 | 2.65 | -0.12 | -0.24 | -1.18 | -0.23 | -0.07 |
| O95391 | Pre-mRNA-splicing factor SLU7 | SLU7 | -0.57 | 0.80 | 0.19 | 0.07 | -0.19 | -0.35 | 1.27 | -1.01 | 2.03 | -1.22 | -0.75 | -0.02 |
| Q9H501 | ESF1 homolog | ESF1 | -0.57 | 1.05 | 1.90 | -0.54 | -0.83 | -0.33 | 1.00 | -0.53 | 1.29 | -0.88 | -0.59 | -0.49 |
| Q06413 | Myocyte-specific enhancer factor 2C | MEF2C | -0.57 | 0.65 | -0.92 | 0.63 | -1.24 | 0.96 | 1.39 | -1.07 | -1.03 | -0.20 | 0.81 | 0.68 |
| Q9Y5K8 | V-type proton ATPase subunit D | ATP6V1D | -0.57 | 1.83 | -0.23 | 0.33 | 0.42 | 0.77 | 0.58 | -1.30 | -1.55 | 1.14 | 1.00 | -1.16 |
| Q99719 | Septin-5 | SEPTIN5 | -0.57 | 1.26 | 0.62 | 0.89 | -1.26 | 0.69 | 0.44 | -0.29 | 1.21 | -1.96 | 0.10 | -0.44 |
| Q969F9 | Hermansky-Pudlak syndrome 3 protein | HPS3 | -0.57 | 1.57 | 2.75 | -0.26 | -0.63 | -0.09 | -0.09 | -0.16 | -0.09 | -0.90 | -0.31 | -0.22 |
| Q05209 | Tyrosine-protein phosphatase non-receptor type 12 | PTPN12 | -0.57 | 0.62 | -0.70 | 1.23 | -1.10 | 0.46 | 0.89 | -0.86 | 1.09 | -1.14 | 0.95 | -0.83 |
| Q96CW1 | AP-2 complex subunit mu | AP2M1 | -0.57 | 4.28 | 1.10 | 1.22 | -0.66 | 0.78 | 0.55 | -2.09 | -0.52 | -0.09 | 0.24 | -0.53 |
| Q8N608 | Inactive dipeptidyl peptidase 10 | DPP10 | -0.57 | 0.77 | 0.24 | 1.39 | 1.13 | -1.54 | -0.27 | 0.07 | -0.76 | 0.66 | 0.50 | -1.42 |
| P56962 | Syntaxin-17 | STX17 | -0.58 | 0.83 | 0.65 | 2.48 | -0.78 | -0.64 | -0.69 | -0.47 | -0.58 | 0.29 | 0.22 | -0.48 |
| P45954 | Short/branched chain specific acyl-CoA dehydrogenase, mitochonc | ACADSB | -0.58 | 0.72 | -0.93 | -0.82 | 1.78 | 0.39 | 0.47 | -1.20 | -1.11 | 1.02 | 0.43 | -0.02 |
| P53618 | Coatomer subunit beta | COPB1 | -0.58 | 0.67 | -0.89 | 0.41 | 0.61 | 0.72 | -0.01 | -1.54 | -1.65 | 1.13 | 0.66 | 0.57 |
| O94811 | Tubulin polymerization-promoting protein | TPPP | -0.58 | 2.45 | 0.22 | 1.08 | -0.09 | 0.67 | 0.39 | -2.14 | -0.03 | -0.64 | 1.28 | -0.74 |
| Q92686 | Neurogranin | NRGN | -0.58 | 1.21 | -0.38 | -0.35 | -0.15 | -0.44 | 2.69 | -0.70 | -0.54 | -0.01 | -0.55 | 0.43 |
| Q9UPP2 | IQ motif and SEC7 domain-containing protein 3 | IQSEC3 | -0.58 | 0.58 | 0.90 | -0.13 | 1.24 | 0.56 | -1.84 | 0.67 | 0.04 | -1.38 | 0.48 | -0.55 |
| Q9UNA1 | Rho GTPase-activating protein 26 | ARHGAP26 | -0.58 | 0.48 | 0.60 | 0.65 | -0.78 | 0.35 | -0.20 | 0.97 | 0.24 | -2.49 | 0.37 | 0.28 |
| O00303 | Eukaryotic translation initiation factor 3 subunit F | EIF3F | -0.58 | 0.63 | 0.83 | 0.55 | 0.06 | 0.54 | -1.18 | 0.87 | -0.61 | -2.15 | 0.53 | 0.57 |
| Q6PKG0 | La-related protein 1 | LARP1 | -0.58 | 1.36 | -0.09 | 0.77 | -0.23 | 1.29 | -0.26 | -2.04 | -0.96 | -0.08 | 1.14 | 0.47 |
| P32519 | ETS-related transcription factor Elf-1 | ELF1 | -0.58 | 1.03 | 1.65 | -0.59 | -0.95 | 0.31 | 0.76 | -0.61 | -0.64 | -0.96 | 1.54 | -0.50 |
| O43157 | Plexin-B1 | PLXNB1 | -0.58 | 0.50 | -0.90 | 0.21 | 0.70 | 0.58 | 0.07 | -2.43 | -0.18 | 0.84 | 0.74 | 0.38 |
| Q8NAB2 | Kelch repeat and BTB domain-containing protein 3 | KBTBD3 | -0.58 | 0.71 | -0.48 | 1.14 | -0.77 | 1.53 | -0.55 | -0.54 | -0.56 | -0.89 | 1.61 | -0.49 |
| P24752 | Acetyl-CoA acetyltransferase, mitochondrial | ACAT1 | -0.58 | 0.74 | -1.09 | 0.99 | 0.58 | 1.26 | -0.84 | -0.91 | -1.43 | 0.08 | 1.14 | 0.22 |
| Q07912 | Activated CDC42 kinase 1 | TNK2 | -0.58 | 0.66 | 0.56 | 0.64 | -1.58 | 0.35 | 0.84 | 1.37 | -1.43 | -0.89 | 0.43 | -0.30 |
| O75746 | Calcium-binding mitochondrial carrier protein Aralar1 | SLC25A12 | -0.58 | 1.72 | 1.37 | -1.04 | -0.39 | 0.40 | 1.38 | -0.87 | -1.64 | 0.16 | 0.03 | 0.60 |
| Q14161 | ARF GTPase-activating protein GIT2 | GIT2 | -0.58 | 1.96 | -0.51 | 1.79 | 0.63 | 0.24 | -0.27 | -0.82 | -1.91 | -0.28 | 0.55 | 0.57 |
| Q6ZMW3 | Echinoderm microtubule-associated protein-like 6 | EML6 | -0.58 | 1.72 | -0.14 | -0.14 | 0.32 | 0.30 | 1.41 | -1.97 | -1.10 | 1.28 | 0.19 | -0.15 |
| P35609 | Alpha-actinin-2 | ACTN2 | -0.58 | 1.24 | -0.46 | 1.74 | 0.23 | -0.20 | 0.05 | -0.31 | 1.09 | -2.08 | -0.19 | 0.13 |
| P78527 | DNA-dependent protein kinase catalytic subunit | PRKDC | -0.58 | 2.37 | -0.65 | 1.41 | 0.14 | 0.89 | 0.35 | -2.01 | -0.87 | -0.35 | 0.29 | 0.80 |
| Q5GLZ8 | Probable E3 ubiquitin-protein ligase HERC4 | HERC4 | -0.58 | 0.74 | 0.99 | 0.88 | -0.34 | 0.35 | -0.98 | 1.15 | -1.22 | -1.68 | 0.53 | 0.32 |
| B7ZBB8 | Protein phosphatase 1 regulatory subunit 3G | PPP1R3G | -0.58 | 1.19 | -0.34 | -0.73 | 1.91 | 1.24 | -0.74 | -0.70 | -0.66 | -1.02 | 0.27 | 0.78 |
| Q9BR61 | Acyl-CoA-binding domain-containing protein 6 | ACBD6 | -0.58 | 0.76 | -0.32 | 1.24 | -1.49 | 1.02 | 0.47 | -0.05 | 0.52 | -1.69 | 0.75 | -0.45 |
| Q7Z2K8 | G protein-regulated inducer of neurite outgrowth 1 | GPRIN1 | -0.58 | 1.67 | -0.56 | 1.53 | 0.64 | 0.71 | -0.63 | -2.18 | 0.10 | -0.23 | 0.42 | 0.21 |
| O75525 | KH domain-containing, RNA-binding, signal transduction-associate | KHDRBS3 | -0.59 | 1.34 | 0.04 | 1.85 | -1.00 | 0.60 | -0.05 | -1.59 | -0.30 | 1.12 | -0.09 | -0.58 |
| Q9UBS5 | Gamma-aminobutyric acid type B receptor subunit 1 | GABBR1 | -0.59 | 0.42 | 1.76 | 0.27 | -0.55 | 0.02 | -0.94 | 1.51 | 0.22 | -1.38 | -0.51 | -0.40 |
| Q53HC0 | Coiled-coil domain-containing protein 92 | CCDC92 | -0.59 | 2.20 | 0.37 | -0.62 | 1.22 | 1.02 | 0.06 | -1.22 | -1.37 | 0.48 | 1.11 | -1.05 |
| Q86T24 | Transcriptional regulator Kaiso | ZBTB33 | -0.59 | 2.97 | 0.44 | 0.13 | -0.20 | 1.29 | 0.85 | -0.52 | 0.21 | -0.66 | -2.26 | 0.71 |
| Q01831 | DNA repair protein complementing XP-C cells | XPC | -0.59 | 0.54 | 1.46 | 0.78 | -0.40 | 0.02 | -1.16 | 1.06 | 0.69 | -1.35 | 0.04 | -1.14 |
| Q9H993 | Damage-control phosphatase ARMT1 | ARMT1 | -0.59 | 0.64 | 0.08 | 0.46 | -0.86 | 0.82 | 0.31 | 0.37 | -0.19 | -2.45 | 0.66 | 0.80 |
| Q06787 | Synaptic functional regulator FMR1 | FMR1 | -0.59 | 1.67 | 1.43 | -0.15 | -0.38 | 1.18 | -0.40 | -0.19 | 0.24 | -2.11 | 0.75 | -0.38 |
| Q9H254 | Spectrin beta chain, non-erythrocytic 4 | SPTBN4 | -0.59 | 2.25 | -0.36 | 1.90 | -0.55 | 0.59 | 0.48 | -1.92 | -0.27 | -0.29 | 0.67 | -0.25 |
| P48426 | Phosphatidylinositol 5-phosphate 4-kinase type-2 alpha | PIP4K2A | -0.59 | 1.47 | 1.04 | 0.72 | 1.13 | -0.94 | -0.41 | 0.16 | -1.52 | 1.24 | -1.02 | -0.40 |
| Q5TDH0 | Protein DDI1 homolog 2 | DDI2 | -0.59 | 1.17 | 1.06 | 1.44 | -0.48 | -0.31 | -0.40 | 0.57 | -0.29 | -2.18 | 0.25 | 0.34 |
| Q8N684 | Cleavage and polyadenylation specificity factor subunit 7 | CPSF7 | -0.59 | 1.92 | 0.39 | -0.44 | -0.68 | 1.25 | 1.34 | -0.33 | -0.23 | -1.52 | 1.19 | -0.98 |
| Q96Q04 | Serine/threonine-protein kinase LMTK3 | LMTK3 | -0.59 | 0.89 | 0.95 | -0.24 | -1.78 | 0.11 | 2.04 | -0.69 | 0.10 | -0.24 | 0.09 | -0.35 |
| Q5T440 | Putative transferase CAF17, mitochondrial | IBA57 | -0.59 | 0.74 | -0.98 | 0.99 | 2.24 | -0.70 | -0.63 | -0.59 | -0.62 | 0.40 | -0.42 | 0.31 |
| P07357 | Complement component C8 alpha chain | C8A | -0.59 | 0.54 | -0.66 | -0.78 | 1.83 | 1.13 | -0.83 | -0.77 | -0.68 | 0.39 | 1.01 | -0.66 |
| Q15818 | Neuronal pentraxin-1 | NPTX1 | -0.59 | 0.52 | -0.44 | 1.45 | -1.35 | 0.90 | 0.11 | -1.70 | 0.09 | -0.46 | 0.74 | 0.66 |
| Q5TAQ9 | DDB1- and CUL4-associated factor 8 | DCAF8 | -0.59 | 0.98 | 0.62 | -1.25 | 0.22 | 1.75 | -0.20 | -0.46 | -0.12 | -1.64 | 0.99 | 0.10 |
| P08559 | Pyruvate dehydrogenase E1 component subunit alpha, somatic for | PDHA1 | -0.59 | 1.45 | -0.12 | -0.83 | -0.51 | 1.46 | 1.52 | -0.03 | -0.18 | -0.59 | 0.85 | -1.58 |
| P63000 | Ras-related C3 botulinum toxin substrate 1 | RAC1 | -0.59 | 3.03 | -0.38 | 1.41 | 0.42 | 0.82 | 0.25 | -1.93 | -0.54 | 0.45 | 0.65 | -1.15 |
| Q70EL4 | Ubiquitin carboxyl-terminal hydrolase 43 | USP43 | -0.59 | 1.62 | 0.00 | -0.13 | -0.56 | 2.74 | -0.32 | -0.34 | -0.32 | -0.90 | -0.17 | 0.00 |
| Q5VTL8 | Pre-mRNA-splicing factor 38B | PRPF38B | -0.59 | 0.80 | 0.50 | -1.64 | 0.54 | 0.06 | 1.50 | 0.36 | -1.64 | -0.53 | 0.29 | 0.56 |
| Q9NS86 | LanC-like protein 2 | LANCL2 | -0.60 | 1.38 | 0.90 | 0.11 | 0.57 | 0.71 | -0.80 | -1.08 | -1.76 | 1.16 | 0.82 | -0.63 |
| Q8IVD9 | NudC domain-containing protein 3 | NUDCD3 | -0.60 | 0.92 | 1.76 | -0.35 | 0.54 | 0.28 | -1.15 | 0.28 | 0.63 | -1.88 | 0.16 | -0.27 |
| Q9BX66 | Sorbin and SH3 domain-containing protein 1 | SORBS1 | -0.60 | 1.31 | -0.22 | 0.46 | 0.39 | 0.75 | 0.09 | 0.61 | -2.56 | -0.66 | 0.60 | 0.53 |
| Q9HB19 | Pleckstrin homology domain-containing family A member 2 | PLEKHA2 | -0.60 | 0.87 | 0.28 | 1.01 | -0.38 | 0.76 | -0.63 | -0.09 | -2.23 | -0.47 | 0.70 | 1.05 |
| Q92688 | Acidic leucine-rich nuclear phosphoprotein 32 family member B | ANP32B | -0.60 | 1.22 | -0.10 | -0.69 | 2.01 | 0.98 | -0.85 | -0.62 | -0.58 | 1.12 | -0.60 | -0.67 |
| P04275 | von Willebrand factor | VWF | -0.60 | 0.79 | -0.85 | -0.56 | 1.23 | 0.44 | 0.69 | -1.29 | -1.65 | 0.79 | 0.73 | 0.47 |
| P62753 | 40S ribosomal protein S6 | RPS6 | -0.60 | 0.70 | -0.14 | -1.55 | 0.17 | 0.12 | 2.27 | -0.30 | -1.10 | 0.25 | 0.15 | 0.11 |

| | | | | | | | | | | | | | | |
|---|---|---|---|---|---|---|---|---|---|---|---|---|---|---|
| Q6R327 | Rapamycin-insensitive companion of mTOR | RICTOR | -0.60 | 1.43 | 0.51 | 0.72 | -0.55 | 0.54 | 0.33 | -1.06 | -2.04 | -0.27 | 0.41 | 1.41 |
| P62195 | 26S proteasome regulatory subunit 8 | PSMC5 | -0.60 | 2.22 | 0.86 | 0.90 | -0.68 | 0.48 | 0.53 | 0.57 | -0.86 | -2.30 | 0.37 | 0.14 |
| P30085 | UMP-CMP kinase | CMPK1 | -0.60 | 1.20 | -1.65 | 1.01 | 0.47 | 0.96 | 0.53 | -1.07 | -1.15 | -0.55 | 0.81 | 0.63 |
| Q99490 | Arf-GAP with GTPase, ANK repeat and PH domain-containing prot | AGAP2 | -0.60 | 0.66 | -1.14 | 0.95 | 0.70 | 0.58 | -0.27 | -1.94 | -0.88 | 0.77 | 0.52 | 0.72 |
| O43301 | Heat shock 70 kDa protein 12A | HSPA12A | -0.60 | 1.70 | 1.16 | 0.42 | -1.30 | 0.94 | 0.47 | -0.34 | 0.21 | -1.97 | 0.72 | -0.33 |
| P20916 | Myelin-associated glycoprotein | MAG | -0.60 | 0.64 | -0.94 | 0.32 | 1.49 | 0.40 | -0.47 | -1.70 | -0.53 | 1.30 | 0.58 | -0.46 |
| O75363 | Breast carcinoma-amplified sequence 1 | BCAS1 | -0.61 | 1.02 | 0.88 | -0.76 | 2.24 | 0.12 | -1.27 | -0.09 | 0.02 | -0.30 | 0.11 | -0.95 |
| Q5TCY1 | Tau-tubulin kinase 1 | TTBK1 | -0.61 | 0.51 | 0.53 | 0.60 | -0.85 | 0.19 | 0.21 | 0.17 | 1.86 | -1.53 | 0.14 | -1.30 |
| Q9BYN0 | Sulfiredoxin-1 | SRXN1 | -0.61 | 0.66 | 0.43 | 0.79 | -1.05 | -0.21 | 0.87 | 1.44 | -0.96 | -1.22 | -0.99 | 0.89 |
| O95714 | E3 ubiquitin-protein ligase HERC2 | HERC2 | -0.61 | 0.77 | -1.21 | 0.47 | 0.94 | 0.61 | 0.13 | -1.50 | -1.50 | 0.86 | 0.80 | 0.40 |
| Q6GMV3 | Putative peptidyl-tRNA hydrolase PTRHD1 | PTRHD1 | -0.61 | 0.54 | -0.60 | 1.53 | -0.63 | -0.74 | 1.13 | -0.66 | 0.16 | -0.95 | -0.72 | 1.49 |
| O95267 | RAS guanyl-releasing protein 1 | RASGRP1 | -0.61 | 0.69 | 1.74 | -0.59 | -0.74 | 1.21 | -0.77 | 0.34 | -0.61 | -0.95 | 1.06 | -0.70 |
| Q9Y4D2 | Sn1-specific diacylglycerol lipase alpha | DAGLA | -0.61 | 0.74 | 1.50 | 0.57 | -1.88 | 0.95 | -0.22 | 0.06 | -0.37 | -0.96 | 0.82 | -0.47 |
| Q6PL24 | Protein TMED8 | TMED8 | -0.61 | 1.34 | 0.56 | 1.39 | -1.04 | 0.75 | -0.22 | 0.44 | -0.67 | -1.97 | 0.74 | 0.03 |
| P42331 | Rho GTPase-activating protein 25 | ARHGAP25 | -0.61 | 0.92 | -0.48 | 0.84 | -0.78 | 0.70 | 0.81 | -1.18 | -1.23 | -0.77 | 0.49 | 1.61 |
| O60488 | Long-chain-fatty-acid--CoA ligase 4 | ACSL4 | -0.61 | 0.91 | -0.45 | 0.06 | 0.37 | -0.63 | 1.71 | 0.26 | -1.80 | 1.28 | -0.58 | -0.24 |
| O60760 | Hematopoietic prostaglandin D synthase | HPGDS | -0.61 | 0.97 | -0.44 | -0.48 | -0.76 | 0.93 | 1.88 | -0.56 | -0.44 | -0.96 | 1.37 | -0.54 |
| P42684 | Tyrosine-protein kinase ABL2 | ABL2 | -0.61 | 0.65 | 0.65 | 0.96 | -0.92 | 0.25 | -0.12 | 0.57 | 0.89 | -2.34 | 0.28 | -0.22 |
| Q01813 | ATP-dependent 6-phosphofructokinase, platelet type | PFKP | -0.61 | 2.08 | -0.21 | 0.93 | 1.63 | 0.16 | -0.56 | -1.03 | -0.53 | -1.65 | 0.27 | 0.98 |
| O43295 | SLIT-ROBO Rho GTPase-activating protein 3 | SRGAP3 | -0.61 | 2.04 | 0.21 | 1.00 | 1.00 | 1.15 | -1.42 | -0.98 | 0.78 | -0.36 | 0.01 | -1.38 |
| P02656 | Apolipoprotein C-III | APOC3 | -0.61 | 1.07 | -0.29 | 1.15 | -0.76 | -0.47 | 1.57 | -0.45 | -0.59 | -1.09 | -0.59 | 1.50 |
| Q6PGP7 | Tetratricopeptide repeat protein 37 | TTC37 | -0.61 | 2.12 | 0.81 | -0.74 | 2.29 | -0.37 | 0.04 | -1.18 | 0.56 | -0.67 | -0.34 | -0.40 |
| P39023 | 60S ribosomal protein L3 | RPL3 | -0.61 | 0.94 | -0.68 | -1.20 | 1.03 | 0.30 | 1.65 | -0.73 | -1.45 | 0.61 | 0.44 | 0.03 |
| P25705 | ATP synthase subunit alpha, mitochondrial | ATP5F1A | -0.61 | 2.27 | -0.03 | -0.24 | 2.65 | 0.11 | -0.34 | -0.88 | -0.34 | -0.84 | 0.24 | -0.33 |
| Q05193 | Dynamin-1 | DNM1 | -0.61 | 1.44 | -0.20 | 1.66 | -0.22 | 0.64 | -0.36 | -1.10 | 0.98 | -1.75 | 0.58 | -0.23 |
| Q5TFE4 | 5'-nucleotidase domain-containing protein 1 | NT5DC1 | -0.61 | 1.02 | -0.01 | 1.70 | 0.53 | 0.18 | -1.23 | -1.96 | -0.24 | 0.39 | 0.20 | 0.45 |
| Q9P035 | Very-long-chain (3R)-3-hydroxyacyl-CoA dehydratase 3 | HACD3 | -0.62 | 0.69 | -1.66 | 0.41 | -0.02 | 1.24 | 0.88 | -0.92 | -1.14 | -0.42 | 1.02 | 0.61 |
| Q8NB46 | Serine/threonine-protein phosphatase 6 regulatory ankyrin repeat | ANKRD52 | -0.62 | 0.52 | -0.54 | -0.74 | -0.88 | 1.09 | 1.75 | -0.68 | -0.70 | -0.91 | 1.01 | 0.59 |
| Q8N7H5 | RNA polymerase II-associated factor 1 homolog | PAF1 | -0.62 | 0.76 | 0.55 | -1.01 | -1.05 | 0.81 | 1.62 | 0.17 | -0.92 | -1.31 | 0.72 | 0.41 |
| Q9BQI5 | SH3-containing GRB2-like protein 3-interacting protein 1 | SGIP1 | -0.62 | 1.40 | 0.68 | 1.38 | 0.28 | -0.14 | -0.70 | -1.99 | 1.31 | -0.01 | -0.50 | -0.30 |
| Q9NPB6 | Partitioning defective 6 homolog alpha | PARD6A | -0.62 | 1.26 | 1.52 | 0.39 | 1.47 | -0.96 | -1.02 | 0.59 | -0.84 | -0.58 | -0.93 | 0.37 |
| Q9Y639 | Neuroplastin | NPTN | -0.62 | 0.81 | 1.20 | 1.45 | -1.37 | -0.09 | -0.22 | 0.13 | -1.15 | -1.12 | 0.24 | 0.94 |
| P05026 | Sodium/potassium-transporting ATPase subunit beta-1 | ATP1B1 | -0.62 | 2.11 | 1.60 | 0.94 | 0.78 | 0.18 | -1.49 | -1.22 | 0.02 | 0.29 | -0.04 | -1.06 |
| Q9P2F8 | Signal-induced proliferation-associated 1-like protein 2 | SIPA1L2 | -0.62 | 2.76 | 1.45 | 0.49 | -0.97 | -0.22 | 1.62 | -0.44 | -0.35 | -1.48 | 0.47 | -0.57 |
| O14559 | Rho GTPase-activating protein 33 | ARHGAP33 | -0.62 | 0.64 | -0.71 | 0.96 | -1.05 | 1.03 | 0.57 | -0.88 | -0.88 | -1.15 | 1.02 | 1.08 |
| Q13200 | 26S proteasome non-ATPase regulatory subunit 2 | PSMD2 | -0.62 | 0.79 | -0.39 | 0.28 | 0.11 | 0.48 | 0.51 | -0.17 | -2.65 | 0.49 | 0.51 | 0.84 |
| P42679 | Megakaryocyte-associated tyrosine-protein kinase | MATK | -0.62 | 0.63 | 0.97 | 0.59 | -0.85 | 0.81 | -0.73 | -0.63 | -0.64 | 1.97 | -0.73 | -0.76 |
| Q5VYS8 | Terminal uridylyltransferase 7 | TUT7 | -0.62 | 0.89 | 0.84 | -0.66 | -0.89 | 0.68 | 1.08 | -0.62 | -0.67 | -0.96 | -0.62 | 1.82 |
| P60520 | Gamma-aminobutyric acid receptor-associated protein-like 2 | GABARAPL2 | -0.62 | 2.48 | 0.61 | 1.62 | -0.52 | -0.05 | 0.54 | -1.60 | 1.20 | -0.41 | -0.31 | -1.08 |
| Q9NQR7 | Coiled-coil domain-containing protein 177 | CCDC177 | -0.62 | 0.74 | 0.62 | 1.29 | -1.13 | 0.21 | -0.09 | 0.95 | 0.30 | -2.15 | -0.12 | 0.12 |
| Q9Y6V0 | Protein piccolo | PCLO | -0.62 | 1.06 | 1.19 | 1.15 | -0.05 | -0.73 | -0.34 | 0.05 | 1.21 | -1.92 | -0.66 | 0.12 |
| Q6NT76 | Homeobox-containing protein 1 | HMBOX1 | -0.62 | 1.75 | 0.00 | -0.24 | -0.49 | -0.17 | 2.71 | -0.15 | -0.17 | -1.05 | -0.39 | -0.06 |
| Q92890 | Ubiquitin recognition factor in ER-associated degradation protein | UFD1 | -0.62 | 2.10 | 0.91 | 1.43 | 0.09 | -0.14 | -0.31 | -2.15 | -0.67 | 0.97 | -0.13 | -0.01 |
| Q9BQ69 | ADP-ribose glycohydrolase MACROD1 | MACROD1 | -0.62 | 0.82 | -0.67 | -0.11 | 1.61 | 0.95 | -0.80 | -0.82 | -0.72 | -1.23 | 1.10 | 0.69 |
| P68371 | Tubulin beta-4B chain | TUBB4B | -0.62 | 2.67 | 0.83 | 1.96 | -0.79 | 0.52 | -0.23 | -0.45 | 0.17 | -1.78 | 0.08 | -0.32 |
| Q9NR46 | Endophilin-B2 | SH3GLB2 | -0.62 | 0.85 | -0.03 | 0.87 | -0.16 | -0.07 | 0.43 | -0.70 | 1.40 | -2.32 | 0.15 | 0.43 |
| Q99460 | 26S proteasome non-ATPase regulatory subunit 1 | PSMD1 | -0.62 | 1.51 | -0.05 | 1.37 | 0.96 | 0.75 | -1.46 | -0.57 | -1.54 | -0.47 | 0.66 | 0.35 |
| P50851 | Lipopolysaccharide-responsive and beige-like anchor protein | LRBA | -0.63 | 0.71 | 1.96 | -0.55 | -0.37 | 0.53 | -0.70 | -1.12 | 0.26 | -0.73 | -0.60 | 1.32 |
| Q13423 | NAD(P) transhydrogenase, mitochondrial | NNT | -0.63 | 0.63 | -1.99 | 1.08 | 0.50 | 0.71 | 0.48 | -0.02 | -1.52 | -0.26 | 0.62 | 0.39 |
| P41240 | Tyrosine-protein kinase CSK | CSK | -0.63 | 0.56 | -2.01 | 1.46 | 1.27 | 0.43 | -0.42 | -0.40 | -0.16 | -0.70 | 0.39 | 0.13 |
| Q13505 | Metaxin-1 | MTX1 | -0.63 | 1.55 | 0.12 | -0.29 | -0.54 | 2.76 | -0.37 | -0.20 | -0.35 | -0.81 | -0.21 | -0.10 |
| O94979 | Protein transport protein Sec31A | SEC31A | -0.63 | 1.67 | 1.02 | 0.26 | -0.40 | 0.61 | 0.25 | -0.34 | -1.92 | -1.27 | 0.54 | 1.26 |
| Q14693 | Phosphatidate phosphatase LPIN1 | LPIN1 | -0.63 | 0.93 | 0.86 | 0.57 | 0.12 | -1.20 | 0.74 | -0.05 | 0.44 | -1.59 | -1.21 | 1.32 |
| P01266 | Thyroglobulin | TG | -0.63 | 1.35 | 2.79 | -0.29 | -0.49 | -0.11 | -0.39 | -0.28 | -0.12 | -0.73 | -0.22 | -0.16 |
| P57737 | Coronin-7 | CORO7 | -0.63 | 1.33 | 1.34 | -0.43 | -1.08 | 1.02 | 0.58 | -0.18 | -0.98 | -1.59 | 0.86 | 0.46 |
| Q86XD5 | Protein FAM131B | FAM131B | -0.63 | 1.93 | 0.38 | 1.60 | -0.02 | 0.08 | -0.15 | -1.59 | 0.88 | 0.73 | -0.41 | -1.50 |
| P52272 | Heterogeneous nuclear ribonucleoprotein M | HNRNPM | -0.63 | 1.48 | 1.56 | -0.50 | -1.06 | 0.98 | 0.55 | -0.04 | -1.36 | -1.15 | 0.84 | 0.17 |
| P62314 | Small nuclear ribonucleoprotein Sm D1 | SNRPD1 | -0.63 | 1.63 | -0.23 | -1.29 | 1.83 | 0.54 | 0.83 | -0.92 | -1.34 | 0.53 | -0.10 | 0.15 |
| Q7Z6G3 | N-terminal EF-hand calcium-binding protein 2 | NECAB2 | -0.63 | 0.95 | 0.83 | 0.47 | -1.43 | 0.67 | 0.56 | 0.17 | 0.81 | -1.43 | 0.75 | -1.40 |
| P10586 | Receptor-type tyrosine-protein phosphatase F | PTPRF | -0.63 | 1.51 | -0.97 | 1.72 | -0.28 | 0.79 | 0.32 | 0.12 | -0.04 | -2.03 | 0.39 | 0.00 |
| Q9HCI5 | Melanoma-associated antigen E1 | MAGEE1 | -0.63 | 1.59 | 2.77 | -0.13 | -0.44 | -0.13 | -0.37 | -0.32 | -0.18 | -0.85 | -0.33 | -0.02 |
| Q01064 | Calcium/calmodulin-dependent 3',5'-cyclic nucleotide phosphodiest | PDE1B | -0.63 | 0.89 | 1.37 | 0.39 | -0.84 | 0.13 | 0.02 | 0.30 | 0.70 | -2.37 | 0.20 | 0.10 |
| Q9H7N4 | Splicing factor, arginine/serine-rich 19 | SCAF1 | -0.63 | 0.72 | -0.72 | -0.07 | -0.28 | 0.46 | 1.49 | -1.69 | -1.13 | 1.20 | 0.62 | 0.12 |
| P14598 | Neutrophil cytosol factor 1 | NCF1 | -0.63 | 0.74 | -0.78 | 1.71 | -0.06 | 1.05 | -1.02 | 0.26 | -0.05 | -1.16 | 1.00 | -0.94 |
| Q9H0E2 | Toll-interacting protein | TOLLIP | -0.64 | 5.14 | 0.55 | 1.60 | -0.02 | 0.44 | 0.74 | 0.08 | -1.30 | -1.89 | 0.14 | -0.35 |
| Q6NW34 | Nucleolus and neural progenitor protein | NEPRO | -0.64 | 2.07 | 0.22 | 2.69 | -0.49 | -0.08 | -0.29 | -0.07 | -0.40 | -1.02 | -0.19 | -0.37 |
| Q12955 | Ankyrin-3 | ANK3 | -0.64 | 1.91 | 0.88 | 0.73 | -1.42 | 1.29 | 0.36 | -0.89 | -0.12 | -1.38 | 0.99 | -0.45 |
| Q15700 | Disks large homolog 2 | DLG2 | -0.64 | 1.71 | -0.05 | 0.59 | 0.40 | -0.35 | 1.17 | -1.37 | 1.77 | -0.86 | -1.11 | -0.18 |
| P43243 | Matrin-3 | MATR3 | -0.64 | 3.60 | 0.91 | -0.90 | 0.92 | 1.02 | 0.79 | -0.32 | -2.03 | -0.77 | 0.28 | 0.10 |
| Q14155 | Rho guanine nucleotide exchange factor 7 | ARHGEF7 | -0.64 | 1.01 | 0.17 | 1.15 | -0.57 | 0.50 | -0.08 | -0.23 | 1.48 | -2.11 | 0.23 | -0.55 |
| Q00537 | Cyclin-dependent kinase 17 | CDK17 | -0.64 | 1.02 | 0.45 | 1.13 | -1.91 | 0.89 | 0.62 | 0.26 | 0.24 | -1.45 | 0.37 | -0.60 |
| Q9UK76 | Jupiter microtubule associated homolog 1 | JPT1 | -0.64 | 0.69 | 0.45 | 0.63 | -0.81 | -0.76 | 1.35 | -0.50 | -0.72 | 1.86 | -0.77 | -0.73 |
| Q92879 | CUGBP Elav-like family member 1 | CELF1 | -0.64 | 1.09 | 0.14 | -0.22 | -1.29 | 1.06 | 1.53 | -1.44 | -0.60 | -0.47 | 1.06 | 0.22 |
| Q8IUR0 | Trafficking protein particle complex subunit 5 | TRAPPC5 | -0.64 | 1.02 | -0.38 | -0.65 | -0.71 | 1.49 | 1.42 | -0.56 | -0.55 | -0.86 | -0.61 | 1.41 |
| P35498 | Sodium channel protein type 1 subunit alpha | SCN1A | -0.64 | 1.20 | -1.59 | 1.63 | 0.01 | 1.16 | 0.13 | -0.40 | 0.46 | -1.42 | 0.19 | -0.17 |
| P55316 | Forkhead box protein G1 | FOXG1 | -0.64 | 1.33 | 1.68 | 1.44 | -0.78 | -0.34 | -0.57 | -0.50 | -0.47 | -1.06 | 1.09 | -0.51 |
| Q9Y4F1 | FERM, ARHGEF and pleckstrin domain-containing protein 1 | FARP1 | -0.64 | 2.00 | 0.98 | 1.16 | -0.84 | -0.39 | 0.99 | 0.27 | -0.64 | -1.90 | 0.77 | -0.40 |
| Q92565 | Rap guanine nucleotide exchange factor 5 | RAPGEF5 | -0.64 | 0.80 | -0.40 | -0.51 | -0.70 | 1.25 | 1.32 | -0.63 | 1.70 | -0.94 | -0.57 | -0.52 |
| O14686 | Histone-lysine N-methyltransferase 2D | KMT2D | -0.64 | 2.22 | 0.96 | 1.47 | -0.57 | 0.87 | -0.69 | -0.03 | 0.35 | -1.89 | 0.29 | -0.76 |
| Q96RF0 | Sorting nexin-18 | SNX18 | -0.64 | 1.05 | 0.18 | 0.28 | 0.14 | 0.77 | -0.13 | 0.76 | -0.71 | -2.52 | 0.81 | 0.43 |
| Q53F19 | Nuclear cap-binding protein subunit 3 | NCBP3 | -0.64 | 0.93 | 1.50 | -1.05 | 0.23 | -0.03 | 0.42 | -1.02 | 1.51 | -1.44 | 0.04 | -0.18 |
| Q5XPI4 | E3 ubiquitin-protein ligase RNF123 | RNF123 | -0.64 | 0.78 | -1.57 | 1.44 | -0.04 | 0.86 | 0.25 | -1.36 | -0.13 | -0.84 | 0.95 | 0.43 |
| Q9P1Y5 | Calmodulin-regulated spectrin-associated protein 3 | CAMSAP3 | -0.64 | 0.95 | 0.98 | 1.63 | -0.46 | 0.65 | -1.70 | -0.97 | -0.35 | 0.58 | 0.24 | -0.62 |

| | | | | | | | | | | | | | | |
|---|---|---|---|---|---|---|---|---|---|---|---|---|---|---|
| Q9UQ16 | Dynamin-3 | DNM3 | -0.64 | 1.46 | 0.75 | 1.75 | -0.41 | 0.58 | -1.14 | 0.00 | -0.03 | -1.85 | 0.30 | 0.05 |
| A6NHR9 | Structural maintenance of chromosomes flexible hinge domain-cc | SMCHD1 | -0.64 | 0.62 | -0.25 | 0.62 | -1.41 | 0.94 | 0.88 | -1.26 | -1.38 | 0.21 | 0.87 | 0.78 |
| P23378 | Glycine dehydrogenase (decarboxylating), mitochondrial | GLDC | -0.65 | 1.59 | 2.77 | -0.24 | -0.53 | -0.06 | -0.22 | -0.05 | -0.28 | -0.83 | -0.31 | -0.23 |
| Q6NXS1 | Protein phosphatase inhibitor 2 family member B | PPP1R2B | -0.65 | 0.58 | 1.50 | 0.32 | -1.02 | -0.87 | 0.80 | 0.71 | -0.93 | 1.19 | -0.88 | -0.81 |
| P23368 | NAD-dependent malic enzyme, mitochondrial | ME2 | -0.65 | 1.01 | -0.61 | 0.57 | 0.95 | -0.03 | 0.30 | -1.66 | -1.67 | 0.67 | 0.40 | 1.08 |
| Q99698 | Lysosomal-trafficking regulator | LYST | -0.65 | 0.66 | 0.28 | -0.66 | -1.24 | 0.87 | 1.58 | -1.08 | -1.11 | 0.40 | 1.07 | -0.11 |
| P25490 | Transcriptional repressor protein YY1 | YY1 | -0.65 | 0.71 | 0.50 | 0.34 | -1.34 | 0.62 | 0.77 | -0.07 | -2.12 | -0.19 | 0.43 | 1.07 |
| Q7Z6K5 | Arpin | ARPIN | -0.65 | 0.71 | 0.20 | 0.47 | -0.39 | 0.69 | -0.08 | 0.37 | 0.05 | -2.64 | 0.91 | 0.41 |
| Q9BU61 | NADH dehydrogenase [ubiquinone] 1 alpha subcomplex assembly | NDUFAF3 | -0.65 | 0.65 | -0.98 | 0.82 | 1.32 | 0.73 | -1.09 | -1.14 | 0.57 | -1.28 | 0.75 | 0.29 |
| Q5W0V3 | Protein FAM160B1 | FAM160B1 | -0.65 | 1.03 | -0.50 | 1.04 | 1.34 | -1.09 | 0.39 | -1.42 | -1.30 | 0.69 | 0.44 | 0.41 |
| Q96PY6 | Serine/threonine-protein kinase Nek1 | NEK1 | -0.65 | 0.81 | 1.52 | -0.57 | -0.32 | -0.06 | 0.40 | -0.43 | -1.13 | -1.44 | 0.51 | 1.52 |
| O43818 | U3 small nucleolar RNA-interacting protein 2 | RRP9 | -0.65 | 1.20 | -1.86 | 1.92 | -0.08 | 0.97 | 0.43 | -0.61 | 0.03 | -0.49 | 0.04 | -0.36 |
| P46783 | 40S ribosomal protein S10 | RPS10 | -0.65 | 2.02 | -0.40 | -0.23 | 0.21 | 0.88 | 1.47 | -2.07 | 0.06 | 0.46 | 0.60 | -0.98 |
| Q12906 | Interleukin enhancer-binding factor 3 | ILF3 | -0.65 | 2.34 | -0.77 | 1.78 | 0.25 | 0.34 | 0.52 | -2.13 | -0.19 | -0.29 | 0.36 | 0.12 |
| P51513 | RNA-binding protein Nova-1 | NOVA1 | -0.65 | 0.87 | 1.40 | -1.56 | -0.81 | 0.82 | 1.19 | 0.15 | -0.30 | -1.05 | 0.65 | -0.50 |
| Q8N653 | Leucine-zipper-like transcriptional regulator 1 | LZTR1 | -0.65 | 1.44 | 0.31 | -0.63 | -1.05 | 1.89 | 1.02 | -0.64 | 0.53 | -1.22 | 0.44 | -0.64 |
| Q5THK1 | Protein PRR14L | PRR14L | -0.65 | 0.42 | -1.36 | 0.06 | 0.57 | 0.81 | 0.47 | -1.45 | -1.43 | 0.66 | 0.85 | 0.81 |
| Q99426 | Tubulin-folding cofactor B | TBCB | -0.65 | 0.97 | 0.04 | -0.53 | 0.74 | 0.47 | 0.44 | -0.08 | -2.55 | 0.83 | 0.74 | -0.09 |
| Q6QEF8 | Coronin-6 | CORO6 | -0.65 | 0.54 | -0.46 | -0.51 | -0.72 | 1.49 | 0.90 | -0.60 | -0.52 | -0.82 | 1.83 | -0.59 |
| Q96Q06 | Perilipin-4 | PLIN4 | -0.65 | 1.06 | -0.48 | -1.30 | 1.35 | 0.08 | 1.56 | -1.04 | -1.05 | 0.48 | -0.21 | 0.62 |
| Q7L8C5 | Synaptotagmin-13 | SYT13 | -0.65 | 0.91 | 0.89 | 0.96 | -1.01 | 0.23 | 0.01 | 0.32 | 1.36 | -1.98 | -0.55 | -0.22 |
| Q14254 | Flotillin-2 | FLOT2 | -0.66 | 0.88 | 0.78 | 0.75 | -1.81 | 0.15 | 1.18 | 0.08 | 0.97 | -1.10 | -0.01 | -0.98 |
| P30711 | Glutathione S-transferase theta-1 | GSTT1 | -0.66 | 0.82 | -0.87 | 0.33 | 0.64 | 0.19 | 0.71 | -1.03 | -1.16 | 1.78 | 0.55 | -1.14 |
| Q9H3Z4 | DnaJ homolog subfamily C member 5 | DNAJC5 | -0.66 | 1.20 | 0.86 | 1.73 | -0.49 | -0.12 | -0.66 | -0.08 | 0.87 | -0.82 | -1.72 | 0.42 |
| Q14676 | Mediator of DNA damage checkpoint protein 1 | MDC1 | -0.66 | 1.03 | 1.59 | 0.13 | -0.96 | 0.30 | 0.12 | 0.04 | 0.05 | -0.39 | 1.12 | -2.01 |
| Q6NXE6 | Armadillo repeat-containing protein 6 | ARMC6 | -0.66 | 0.78 | -0.06 | -0.11 | -1.22 | 0.91 | 1.43 | 0.17 | -0.91 | -1.62 | 0.98 | 0.44 |
| P23471 | Receptor-type tyrosine-protein phosphatase zeta | PTPRZ1 | -0.66 | 0.99 | -1.00 | 0.10 | 0.27 | 0.92 | 0.86 | -1.45 | -1.46 | 1.06 | 0.96 | -0.25 |
| Q7Z6I6 | Rho GTPase-activating protein 30 | ARHGAP30 | -0.66 | 1.64 | 0.04 | 2.75 | -0.39 | -0.25 | -0.41 | -0.07 | -0.35 | -0.92 | -0.20 | -0.20 |
| Q8IV50 | LysM and putative peptidoglycan-binding domain-containing protei | LYSMD2 | -0.66 | 1.26 | 0.33 | 0.96 | -0.17 | -0.06 | 0.37 | 0.84 | 0.20 | -2.64 | -0.08 | 0.27 |
| P08572 | Collagen alpha-2(IV) chain | COL4A2 | -0.66 | 0.85 | -0.48 | -0.63 | 1.98 | 0.02 | 0.12 | -1.45 | 0.80 | -0.97 | -0.23 | 0.83 |
| Q9P2K8 | eIF-2-alpha kinase GCN2 | EIF2AK4 | -0.66 | 0.98 | -0.57 | 0.41 | -1.09 | 1.22 | 1.15 | -0.16 | -0.44 | -1.74 | 0.09 | 1.11 |
| Q9H8T0 | AKT-interacting protein | AKTIP | -0.66 | 1.36 | 2.80 | -0.25 | -0.47 | -0.20 | -0.37 | -0.06 | -0.25 | -0.69 | -0.28 | -0.25 |
| Q16774 | Guanylate kinase | GUK1 | -0.66 | 1.17 | 0.90 | 0.79 | -0.49 | 0.43 | -0.31 | 0.71 | 0.63 | -2.47 | -0.01 | -0.17 |
| Q6NYC8 | Phostensin | PPP1R18 | -0.66 | 1.00 | 0.74 | 2.01 | -1.04 | 0.29 | -0.84 | 0.32 | -0.87 | -0.91 | -0.51 | 0.79 |
| P62917 | 60S ribosomal protein L8 | RPL8 | -0.66 | 0.74 | -0.85 | -0.78 | 0.05 | -0.11 | 2.62 | -0.66 | -0.47 | 0.35 | -0.07 | -0.07 |
| P56385 | ATP synthase subunit e, mitochondrial | ATP5ME | -0.66 | 0.47 | 0.48 | -0.83 | -0.91 | 1.55 | 0.32 | -0.79 | -0.82 | -1.02 | 1.45 | 0.56 |
| Q8TEA7 | TBC domain-containing protein kinase-like protein | TBCK | -0.66 | 1.49 | 0.08 | 2.77 | -0.49 | -0.24 | -0.49 | -0.20 | -0.25 | -0.77 | -0.29 | -0.12 |
| Q9NRY4 | Rho GTPase-activating protein 35 | ARHGAP35 | -0.67 | 1.14 | 0.13 | 1.09 | -0.70 | 0.77 | -0.01 | -0.15 | 0.89 | -2.37 | 0.49 | -0.15 |
| Q9NQC1 | E3 ubiquitin-protein ligase Jade-2 | JADE2 | -0.67 | 1.53 | 2.77 | -0.06 | -0.55 | -0.24 | -0.25 | -0.18 | -0.31 | -0.84 | -0.23 | -0.10 |
| Q9UKA4 | A-kinase anchor protein 11 | AKAP11 | -0.67 | 1.55 | 1.78 | 0.33 | -0.84 | 0.98 | -0.66 | -0.48 | -0.53 | -1.26 | -0.47 | 1.14 |
| O43310 | CBP80/20-dependent translation initiation factor | CTIF | -0.67 | 0.98 | 1.03 | 0.80 | -0.57 | 0.64 | -0.76 | 0.87 | -2.15 | -0.49 | 0.64 | -0.01 |
| Q8NE09 | Regulator of G-protein signaling 22 | RGS22 | -0.67 | 0.39 | -1.24 | -0.30 | 1.13 | 0.33 | 0.61 | -1.25 | -1.24 | 1.51 | 0.47 | -0.01 |
| Q9UJC3 | Protein Hook homolog 1 | HOOK1 | -0.67 | 0.60 | 0.84 | -0.85 | -1.13 | 0.13 | 1.76 | 0.07 | 0.69 | -1.29 | -0.83 | 0.60 |
| Q5JR59 | Microtubule-associated tumor suppressor candidate 2 | MTUS2 | -0.67 | 1.48 | 2.80 | -0.14 | -0.60 | -0.05 | -0.38 | -0.19 | -0.27 | -0.61 | -0.26 | -0.30 |
| Q9BPU6 | Dihydropyrimidinase-related protein 5 | DPYSL5 | -0.67 | 1.08 | -1.35 | 0.83 | 0.67 | 0.21 | 0.87 | -0.97 | -1.65 | 1.23 | 0.23 | -0.06 |
| Q9BUR5 | MICOS complex subunit MIC26 | APOO | -0.67 | 0.70 | 0.47 | -1.07 | -1.19 | 1.07 | 1.60 | -0.12 | -0.55 | -1.27 | 0.67 | 0.39 |
| Q6ZVM7 | TOM1-like protein 2 | TOM1L2 | -0.67 | 0.97 | -0.23 | 1.28 | 1.04 | 0.07 | -1.04 | 0.83 | 0.84 | -1.80 | -0.48 | -0.52 |
| Q7L5Y9 | E3 ubiquitin-protein transferase MAEA | MAEA | -0.67 | 1.25 | 1.04 | 0.88 | -0.64 | -0.15 | 0.25 | 0.63 | 1.04 | -2.18 | -0.59 | -0.29 |
| Q9Y6A4 | Cilia- and flagella-associated protein 20 | CFAP20 | -0.67 | 1.67 | 0.05 | -0.62 | 0.54 | 0.82 | 0.92 | -0.98 | -2.25 | 0.52 | 0.54 | 0.46 |
| Q8IV36 | Protein HID1 | HID1 | -0.67 | 1.64 | -0.02 | 2.75 | -0.60 | -0.10 | -0.29 | -0.08 | -0.32 | -0.85 | -0.39 | -0.10 |
| Q9P2R7 | Succinate--CoA ligase [ADP-forming] subunit beta, mitochondrial | SUCLA2 | -0.67 | 1.29 | -0.19 | 0.90 | 1.33 | -0.13 | -0.50 | -1.19 | 1.50 | -1.54 | -0.31 | 0.12 |
| Q7Z7K6 | Centromere protein V | CENPV | -0.67 | 0.89 | 1.10 | -1.58 | -0.71 | 0.48 | 1.78 | 0.09 | -0.97 | -0.25 | 0.48 | -0.41 |
| Q8TCS8 | Polyribonucleotide nucleotidyltransferase 1, mitochondrial | PNPT1 | -0.67 | 1.01 | -0.43 | 0.56 | 2.17 | -0.54 | -0.59 | -0.55 | 1.25 | -0.92 | -0.47 | -0.47 |
| Q05655 | Protein kinase C delta type | PRKCD | -0.67 | 1.22 | 0.14 | 1.91 | -0.53 | -0.26 | 0.07 | 0.68 | 0.64 | -1.96 | -0.33 | -0.36 |
| Q8NI08 | Nuclear receptor coactivator 7 | NCOA7 | -0.68 | 1.02 | 0.15 | 1.26 | 0.40 | -0.43 | -0.20 | -0.52 | 1.96 | -1.32 | -0.28 | -1.03 |
| Q8IUR7 | Armadillo repeat-containing protein 8 | ARMC8 | -0.68 | 0.91 | 1.56 | 1.35 | -1.03 | 0.14 | -0.95 | -0.75 | -0.13 | -1.26 | 0.49 | 0.59 |
| Q8TAM6 | Ermin | ERMN | -0.68 | 0.76 | -0.31 | -0.27 | 0.90 | 0.56 | 0.06 | -0.51 | -2.38 | 1.10 | 0.76 | 0.09 |
| Q9NRD5 | PRKCA-binding protein | PICK1 | -0.68 | 2.96 | -1.26 | 0.55 | 0.89 | 0.39 | 1.96 | -0.21 | -0.76 | -0.95 | 0.26 | -0.87 |
| P46776 | 60S ribosomal protein L27a | RPL27A | -0.68 | 0.82 | -0.44 | -1.05 | 0.58 | -0.45 | 2.36 | -0.27 | 0.45 | -1.06 | -0.34 | 0.23 |
| P24043 | Laminin subunit alpha-2 | LAMA2 | -0.68 | 5.15 | 0.88 | -0.41 | 1.12 | 1.45 | 0.22 | -0.53 | -1.93 | -0.81 | 0.02 | -0.01 |
| Q9NQ89 | Protein C12orf4 | C12orf4 | -0.68 | 0.82 | 1.02 | 0.56 | -1.06 | 0.57 | -0.10 | 0.32 | 1.02 | -2.25 | -0.07 | -0.01 |
| O00750 | Phosphatidylinositol 4-phosphate 3-kinase C2 domain-containing s | PIK3C2B | -0.68 | 1.43 | -0.06 | 2.79 | -0.59 | -0.17 | -0.38 | -0.18 | -0.29 | -0.71 | -0.18 | -0.23 |
| Q96JK2 | DDB1- and CUL4-associated factor 5 | DCAF5 | -0.68 | 2.09 | 2.62 | -0.22 | -0.60 | 0.51 | -0.25 | -0.31 | -0.22 | -1.07 | -0.33 | -0.12 |
| Q6PID6 | Tetratricopeptide repeat protein 33 | TTC33 | -0.68 | 1.53 | 2.78 | -0.09 | -0.57 | -0.17 | -0.28 | -0.14 | -0.16 | -0.79 | -0.36 | -0.21 |
| Q9Y2S7 | Polymerase delta-interacting protein 2 | POLDIP2 | -0.68 | 1.72 | -0.18 | -0.31 | 2.79 | -0.19 | -0.31 | -0.28 | -0.32 | -0.86 | -0.20 | -0.15 |
| O75077 | Disintegrin and metalloproteinase domain-containing protein 23 | ADAM23 | -0.68 | 1.38 | -1.36 | 0.02 | 0.76 | 0.15 | 1.92 | -1.12 | -1.09 | 0.65 | 0.11 | -0.04 |
| Q9H307 | Pinin | PNN | -0.68 | 1.02 | 0.54 | -1.67 | 1.30 | 0.22 | 0.78 | 0.16 | -1.83 | 0.42 | 0.09 | 0.00 |
| Q02086 | Transcription factor Sp2 | SP2 | -0.68 | 1.03 | -0.40 | -0.55 | -0.71 | 1.22 | 1.62 | -0.49 | -0.50 | -0.96 | 1.44 | -0.67 |
| Q92782 | Zinc finger protein neuro-d4 | DPF1 | -0.68 | 1.10 | 2.72 | -0.35 | -0.61 | -0.21 | -0.26 | 0.39 | -0.35 | -0.69 | -0.39 | -0.24 |
| Q9Y263 | Phospholipase A-2-activating protein | PLAA | -0.68 | 0.57 | -1.61 | 0.56 | 0.50 | 0.59 | 0.68 | -1.71 | -0.93 | 0.77 | 0.68 | 0.46 |
| Q9H902 | Receptor expression-enhancing protein 1 | REEP1 | -0.68 | 1.79 | -0.52 | 1.67 | -1.03 | 0.32 | 1.34 | -0.70 | -0.04 | -1.53 | 0.14 | 0.35 |
| Q8TEV9 | Guanine nucleotide exchange protein SMCR8 | SMCR8 | -0.68 | 0.83 | -0.39 | 1.21 | -1.00 | 0.30 | 0.87 | -2.17 | 0.84 | 0.32 | 0.15 | -0.13 |
| Q8TCT0 | Ceramide kinase | CERK | -0.68 | 2.45 | 2.38 | -0.23 | -0.62 | 0.96 | -0.22 | -0.26 | -0.36 | -1.05 | 0.19 | -0.79 |
| Q8N5C8 | TGF-beta-activated kinase 1 and MAP3K7-binding protein 3 | TAB3 | -0.68 | 0.82 | 1.15 | 1.01 | -1.39 | 0.50 | -0.30 | 1.28 | -0.46 | -1.44 | 0.26 | -0.63 |
| P09038 | Fibroblast growth factor 2 | FGF2 | -0.68 | 0.45 | -0.72 | -1.20 | 0.55 | 0.43 | 1.52 | -0.35 | -1.80 | 0.61 | 0.34 | 0.61 |
| B7ZAP0 | Rab GTPase-activating protein 1-like, isoform 10 | RABGAP1L | -0.68 | 1.43 | -0.03 | 2.80 | -0.59 | -0.28 | -0.31 | -0.21 | -0.32 | -0.62 | -0.28 | -0.17 |
| P16220 | Cyclic AMP-responsive element-binding protein 1 | CREB1 | -0.68 | 1.06 | 1.60 | -1.53 | 1.05 | 0.34 | -0.24 | -0.31 | 0.65 | 0.01 | -0.05 | -1.52 |
| Q8N3D4 | EH domain-binding protein 1-like protein 1 | EHBP1L1 | -0.68 | 1.53 | -0.06 | 2.78 | -0.45 | -0.13 | -0.47 | -0.26 | -0.17 | -0.82 | -0.21 | -0.21 |
| Q9H267 | Vacuolar protein sorting-associated protein 33B | VPS33B | -0.68 | 0.94 | 0.04 | 1.56 | -0.37 | 0.60 | -0.73 | 0.02 | 0.70 | -2.23 | 0.27 | 0.14 |
| Q9BY32 | Inosine triphosphate pyrophosphatase | ITPA | -0.69 | 0.87 | -0.70 | 1.60 | -1.05 | 0.57 | 0.62 | -0.85 | -0.86 | 1.38 | 0.16 | -0.85 |
| P02686 | Myelin basic protein | MBP | -0.69 | 0.98 | -0.34 | -0.36 | 2.22 | 0.48 | -0.86 | -0.83 | -0.84 | 0.70 | 0.61 | -0.78 |
| Q9NQW7 | Xaa-Pro aminopeptidase 1 | XPNPEP1 | -0.69 | 0.81 | -1.44 | 0.28 | 0.39 | 0.19 | 1.56 | -1.55 | -0.93 | 0.61 | 0.74 | 0.16 |
| Q2TAL8 | Glutamine-rich protein 1 | QRICH1 | -0.69 | 0.84 | 1.31 | -0.54 | -1.02 | 0.74 | 0.51 | 0.74 | -1.73 | -1.01 | 0.74 | 0.25 |

| | | | | | | | | | | | | | | |
|---|---|---|---|---|---|---|---|---|---|---|---|---|---|---|
| Q5T0D9 | Tumor protein p63-regulated gene 1-like protein | TPRG1L | -0.69 | 1.53 | 0.04 | 1.39 | -0.16 | 0.18 | 0.17 | -0.67 | 0.30 | -2.38 | 0.31 | 0.83 |
| Q9UMR2 | ATP-dependent RNA helicase DDX19B | DDX19B | -0.69 | 1.51 | 0.15 | 1.06 | -0.72 | 0.46 | 0.65 | 0.52 | 0.77 | -2.39 | -0.32 | -0.16 |
| Q12933 | TNF receptor-associated factor 2 | TRAF2 | -0.69 | 1.07 | 0.81 | 1.08 | -1.19 | 0.98 | -0.46 | 0.33 | 0.31 | -1.98 | -0.41 | 0.54 |
| Q8N1G1 | RNA exonuclease 1 homolog | REXO1 | -0.69 | 1.56 | -0.34 | -0.07 | -0.99 | 0.91 | 2.10 | -0.68 | 0.52 | -1.19 | 0.33 | -0.60 |
| O60264 | SWI/SNF-related matrix-associated actin-dependent regulator of c | SMARCA5 | -0.69 | 1.39 | -0.92 | 1.53 | 0.20 | 0.61 | 0.06 | -1.31 | -0.67 | -1.27 | 0.78 | 0.99 |
| P06576 | ATP synthase subunit beta, mitochondrial | ATP5F1B | -0.69 | 1.88 | 0.46 | 0.27 | 1.52 | -0.20 | -0.19 | -2.27 | -0.49 | 0.93 | 0.16 | -0.18 |
| Q92581 | Sodium/hydrogen exchanger 6 | SLC9A6 | -0.69 | 1.32 | 1.23 | 0.99 | -1.79 | 0.23 | 0.78 | -1.48 | -0.25 | -0.41 | 0.38 | 0.32 |
| O75334 | Liprin-alpha-2 | PPFIA2 | -0.69 | 1.57 | 1.24 | 0.98 | -1.12 | 0.47 | 0.04 | 0.14 | 0.72 | -2.00 | 0.19 | -0.66 |
| Q96AX9 | E3 ubiquitin-protein ligase MIB2 | MIB2 | -0.69 | 1.21 | 1.43 | 0.49 | -1.20 | 0.30 | 0.32 | 1.15 | -0.15 | -1.87 | 0.01 | -0.47 |
| P53675 | Clathrin heavy chain 2 | CLTCL1 | -0.69 | 1.64 | -0.07 | -0.31 | -0.58 | -0.06 | 2.79 | -0.30 | -0.31 | -0.68 | -0.13 | -0.33 |
| Q9NVE7 | Pantothenate kinase 4 | PANK4 | -0.69 | 1.84 | 1.42 | 0.17 | 0.52 | 0.49 | -0.78 | 0.49 | 0.28 | -2.32 | 0.09 | -0.35 |
| Q08J23 | tRNA (cytosine(34)-C(5))-methyltransferase | NSUN2 | -0.69 | 1.11 | 1.78 | 0.14 | -1.28 | 0.35 | 0.25 | -0.23 | 0.43 | -1.82 | -0.24 | 0.61 |
| Q12756 | Kinesin-like protein KIF1A | KIF1A | -0.69 | 1.03 | -0.90 | 2.04 | -0.37 | 0.42 | -0.01 | -1.13 | -1.30 | 0.07 | 0.49 | 0.69 |
| Q9NVQ4 | Fas apoptotic inhibitory molecule 1 | FAIM | -0.69 | 0.99 | -0.91 | 0.45 | -0.10 | 1.17 | 0.54 | -0.95 | -1.08 | -1.26 | 1.50 | 0.63 |
| Q00613 | Heat shock factor protein 1 | HSF1 | -0.69 | 1.38 | 2.78 | -0.30 | -0.56 | -0.11 | -0.27 | -0.15 | -0.25 | -0.77 | -0.33 | -0.02 |
| Q8IX12 | Cell division cycle and apoptosis regulator protein 1 | CCAR1 | -0.69 | 1.40 | 0.17 | 1.29 | 1.10 | -1.48 | 0.40 | -0.74 | -0.56 | 1.31 | -0.85 | -0.63 |
| Q6P2Q9 | Pre-mRNA-processing-splicing factor 8 | PRPF8 | -0.70 | 0.79 | 0.62 | 0.20 | 0.25 | 0.40 | -0.49 | 0.84 | -2.61 | 0.62 | 0.40 | -0.23 |
| P40938 | Replication factor C subunit 3 | RFC3 | -0.70 | 1.01 | 1.02 | 1.71 | 0.02 | 0.08 | -1.65 | 0.21 | 0.54 | -1.12 | 0.03 | -0.84 |
| Q13425 | Beta-2-syntrophin | SNTB2 | -0.70 | 0.90 | 0.73 | 0.69 | -1.08 | 0.28 | 0.45 | 0.12 | 0.12 | -2.42 | 0.48 | 0.64 |
| Q96PE2 | Rho guanine nucleotide exchange factor 17 | ARHGEF17 | -0.70 | 0.79 | 0.53 | -0.40 | -0.33 | 0.28 | 0.90 | -0.62 | 0.79 | -2.40 | 0.54 | 0.72 |
| Q8IXW5 | Putative RNA polymerase II subunit B1 CTD phosphatase RPAP2 | RPAP2 | -0.70 | 1.38 | 2.78 | -0.23 | -0.41 | -0.27 | -0.33 | -0.10 | -0.22 | -0.85 | -0.20 | -0.16 |
| Q99884 | Sodium-dependent proline transporter | SLC6A7 | -0.70 | 0.79 | 0.01 | 1.69 | -0.83 | 0.02 | 0.07 | -0.70 | 1.84 | -0.99 | -0.53 | -0.56 |
| Q9H871 | E3 ubiquitin-protein transferase RMND5A | RMND5A | -0.70 | 0.57 | -0.79 | 0.31 | 0.50 | 0.13 | 0.57 | -1.50 | -1.51 | 1.73 | 0.27 | 0.27 |
| Q8IVF5 | T-lymphoma invasion and metastasis-inducing protein 2 | TIAM2 | -0.70 | 1.69 | 1.15 | 0.41 | -1.60 | 0.82 | 0.93 | 0.03 | -1.01 | -1.45 | 0.32 | 0.41 |
| Q9Y3C6 | Peptidyl-prolyl cis-trans isomerase-like 1 | PPIL1 | -0.70 | 1.81 | 0.21 | 1.22 | -0.18 | 0.88 | -0.31 | 0.41 | -1.90 | -1.44 | 0.73 | 0.39 |
| Q15283 | Ras GTPase-activating protein 2 | RASA2 | -0.70 | 0.89 | 1.85 | -0.18 | -1.22 | 0.53 | 0.07 | -0.39 | 1.14 | -1.35 | 0.21 | -0.67 |
| P23468 | Receptor-type tyrosine-protein phosphatase delta | PTPRD | -0.70 | 1.00 | 1.99 | 0.35 | -0.52 | 0.51 | -1.17 | -0.22 | 0.53 | -0.60 | 0.56 | -1.43 |
| Q13085 | Acetyl-CoA carboxylase 1 | ACACA | -0.70 | 1.74 | 0.81 | 1.42 | -0.68 | 0.47 | -0.29 | 0.32 | 0.81 | -2.06 | -0.08 | -0.73 |
| Q96CM8 | Medium-chain acyl-CoA ligase ACSF2, mitochondrial | ACSF2 | -0.70 | 1.30 | -0.65 | 0.41 | 1.98 | -0.65 | 0.32 | -0.83 | -0.67 | -1.29 | 0.36 | 1.02 |
| P25205 | DNA replication licensing factor MCM3 | MCM3 | -0.70 | 1.43 | 2.80 | -0.28 | -0.44 | -0.28 | -0.22 | -0.12 | -0.27 | -0.76 | -0.32 | -0.10 |
| Q9H0M0 | NEDD4-like E3 ubiquitin-protein ligase WWP1 | WWP1 | -0.70 | 1.04 | 0.63 | 1.62 | -1.07 | 0.91 | -0.91 | 0.45 | -0.96 | -1.35 | 0.26 | 0.40 |
| Q00169 | Phosphatidylinositol transfer protein alpha isoform | PITPNA | -0.70 | 1.14 | -0.44 | -0.11 | 0.47 | 0.52 | 0.88 | -1.33 | -2.08 | 0.70 | 0.72 | 0.68 |
| Q6ZVC0 | Neuronal tyrosine-phosphorylated phosphoinositide-3-kinase adapt | NYAP1 | -0.70 | 0.91 | 0.09 | 1.47 | -1.60 | 0.54 | 0.57 | 0.17 | 0.52 | -1.75 | 0.37 | -0.38 |
| Q01081 | Splicing factor U2AF 35 kDa subunit | U2AF1 | -0.70 | 1.34 | 1.39 | -1.01 | 0.35 | 0.43 | 0.29 | 0.56 | -2.21 | -0.47 | 0.42 | 0.27 |
| O43318 | Mitogen-activated protein kinase kinase kinase 7 | MAP3K7 | -0.70 | 0.93 | 0.23 | 1.70 | -0.72 | 0.48 | -0.60 | -1.88 | -0.50 | -0.20 | 0.79 | 0.71 |
| P52747 | Zinc finger protein 143 | ZNF143 | -0.70 | 1.17 | 1.98 | -0.54 | -0.67 | -0.71 | 1.26 | 0.08 | -0.57 | -1.05 | 0.74 | -0.52 |
| Q9NWZ5 | Uridine-cytidine kinase-like 1 | UCKL1 | -0.70 | 0.90 | 0.95 | -0.03 | -1.40 | 1.02 | 0.51 | -0.04 | -1.21 | -1.44 | 0.85 | 0.78 |
| P05161 | Ubiquitin-like protein ISG15 | ISG15 | -0.70 | 0.70 | -1.47 | 1.53 | 0.56 | 0.19 | 0.06 | 0.05 | -1.90 | 0.08 | 0.19 | 0.71 |
| O14827 | Ras-specific guanine nucleotide-releasing factor 2 | RASGRF2 | -0.70 | 0.90 | 0.87 | 0.08 | -0.34 | 0.43 | 0.05 | 1.16 | 0.84 | -2.33 | -0.09 | -0.67 |
| O95758 | Polypyrimidine tract-binding protein 3 | PTBP3 | -0.71 | 0.65 | 1.22 | -1.19 | -0.69 | 0.70 | 0.76 | 0.56 | -1.17 | -1.43 | 0.50 | 0.75 |
| Q9NWB1 | RNA binding protein fox-1 homolog 1 | RBFOX1 | -0.71 | 0.87 | 0.49 | -1.74 | 0.12 | 0.66 | 1.50 | -1.51 | -0.51 | 0.17 | 0.66 | 0.16 |
| O95810 | Caveolae-associated protein 2 | CAVIN2 | -0.71 | 0.80 | 1.47 | 0.37 | -1.65 | 0.24 | 0.53 | 0.53 | 0.81 | -1.31 | 0.01 | -1.00 |
| P11234 | Ras-related protein Ral-B | RALB | -0.71 | 2.22 | 0.41 | 1.07 | 0.40 | 0.26 | 0.00 | 0.19 | -1.99 | -1.57 | 0.27 | 0.96 |
| Q92614 | Unconventional myosin-XVIIIa | MYO18A | -0.71 | 1.59 | -0.72 | 1.72 | -0.03 | 0.84 | -0.18 | -0.63 | 0.67 | -1.97 | 0.28 | 0.02 |
| Q96N21 | AP-4 complex accessory subunit Tepsin | TEPSIN | -0.71 | 1.84 | 2.76 | -0.05 | -0.49 | -0.12 | -0.22 | -0.28 | -0.31 | -0.91 | -0.26 | -0.12 |
| O00194 | Ras-related protein Rab-27B | RAB27B | -0.71 | 1.61 | 0.14 | 2.74 | -0.62 | -0.09 | -0.44 | -0.32 | -0.25 | -0.83 | -0.13 | -0.20 |
| Q9H444 | Charged multivesicular body protein 4b | CHMP4B | -0.71 | 2.33 | -0.27 | 0.35 | 0.33 | -0.25 | 1.95 | -1.68 | 0.50 | -1.17 | -0.31 | 0.55 |
| Q9H6F5 | Coiled-coil domain-containing protein 86 | CCDC86 | -0.71 | 1.62 | 0.30 | -1.17 | 1.71 | 0.43 | 0.38 | -1.38 | -0.17 | -0.44 | -0.85 | 1.20 |
| Q9H6W3 | Ribosomal oxygenase 1 | RIOX1 | -0.71 | 1.78 | -0.07 | -0.25 | 2.78 | -0.34 | -0.28 | -0.20 | -0.40 | -0.84 | -0.23 | -0.17 |
| P51665 | 26S proteasome non-ATPase regulatory subunit 7 | PSMD7 | -0.71 | 1.38 | -0.30 | -0.60 | -0.80 | 1.00 | 2.19 | 0.74 | -0.36 | -1.04 | -0.48 | -0.36 |
| Q15291 | Retinoblastoma-binding protein 5 | RBBP5 | -0.71 | 1.73 | -0.67 | 1.45 | -1.08 | 1.47 | 0.57 | -0.79 | -0.93 | 0.32 | 0.58 | -0.92 |
| Q86X10 | Ral GTPase-activating protein subunit beta | RALGAPB | -0.71 | 2.46 | -0.03 | 0.53 | -0.47 | 0.99 | 1.18 | -0.89 | -1.95 | -0.75 | 0.52 | 0.84 |
| Q14318 | Peptidyl-prolyl cis-trans isomerase FKBP8 | FKBP8 | -0.71 | 1.88 | 1.60 | -0.66 | -0.55 | 1.15 | 0.29 | -0.29 | -0.60 | -1.76 | 0.86 | -0.04 |
| Q9H4L4 | Sentrin-specific protease 3 | SENP3 | -0.71 | 2.95 | 0.29 | -0.68 | 0.63 | 1.16 | 1.07 | -2.19 | -0.78 | -0.02 | 0.41 | 0.12 |
| Q9NZ08 | Endoplasmic reticulum aminopeptidase 1 | ERAP1 | -0.71 | 0.87 | -0.49 | 0.97 | 1.93 | -0.71 | -0.67 | -0.73 | 0.54 | -1.00 | -0.73 | 0.88 |
| P82970 | High mobility group nucleosome-binding domain-containing protei | HMGN5 | -0.71 | 2.44 | -0.08 | 2.20 | 0.06 | -0.08 | 0.07 | -1.37 | -0.48 | -0.43 | -0.89 | 1.01 |
| Q9H6Z4 | Ran-binding protein 3 | RANBP3 | -0.71 | 0.88 | -1.27 | 0.42 | 0.17 | 1.23 | 0.49 | -1.91 | -0.75 | 0.47 | 1.00 | 0.14 |
| O43147 | Small G protein signaling modulator 2 | SGSM2 | -0.71 | 1.00 | -0.70 | 1.63 | -1.00 | 1.56 | -0.32 | -0.68 | -0.79 | 0.63 | 0.46 | -0.79 |
| Q08257 | Quinone oxidoreductase | CRYZ | -0.71 | 1.04 | -1.46 | 0.70 | 0.88 | 0.47 | 0.59 | -1.36 | -1.49 | 0.72 | 0.47 | 0.48 |
| P42685 | Tyrosine-protein kinase FRK | FRK | -0.71 | 0.52 | -1.39 | 0.72 | 0.25 | 0.73 | 0.37 | -1.43 | -1.43 | 0.42 | 0.93 | 0.83 |
| P57723 | Poly(rC)-binding protein 4 | PCBP4 | -0.72 | 1.03 | 0.19 | -0.07 | -0.49 | 1.32 | 0.24 | 0.73 | -2.02 | -0.67 | 1.26 | -0.50 |
| P04350 | Tubulin beta-4A chain | TUBB4A | -0.72 | 2.52 | 0.65 | 1.54 | -0.91 | 0.47 | 0.47 | -0.29 | 0.21 | -2.18 | 0.17 | -0.13 |
| Q9NW64 | Pre-mRNA-splicing factor RBM22 | RBM22 | -0.72 | 1.14 | 1.30 | -0.33 | -1.10 | 0.37 | 1.03 | 0.47 | -1.21 | -1.59 | 0.50 | 0.56 |
| Q9ULH4 | Leucine-rich repeat and fibronectin type-III domain-containing pro | LRFN2 | -0.72 | 1.47 | -0.01 | 2.80 | -0.56 | -0.27 | -0.33 | -0.27 | -0.25 | -0.62 | -0.22 | -0.25 |
| Q8ND24 | RING finger protein 214 | RNF214 | -0.72 | 1.01 | -0.59 | 1.24 | 1.14 | 0.61 | -1.23 | -1.23 | -1.33 | 0.52 | 0.50 | 0.38 |
| Q9Y3Z3 | Deoxynucleoside triphosphate triphosphohydrolase SAMHD1 | SAMHD1 | -0.72 | 1.22 | -0.32 | -0.21 | 0.50 | 0.98 | 0.43 | -0.08 | -2.60 | 0.18 | 0.72 | 0.40 |
| O00478 | Butyrophilin subfamily 3 member A3 | BTN3A3 | -0.72 | 1.05 | 0.10 | 1.19 | -0.36 | 0.99 | -0.72 | -0.03 | -1.41 | -1.52 | 1.11 | 0.65 |
| Q6P3X3 | Tetratricopeptide repeat protein 27 | TTC27 | -0.72 | 0.83 | 0.71 | -0.56 | -0.67 | -0.46 | 1.97 | 1.45 | -0.51 | -0.85 | -0.53 | -0.55 |
| Q05329 | Glutamate decarboxylase 2 | GAD2 | -0.72 | 2.39 | -0.53 | 1.93 | -0.30 | 0.74 | 0.29 | -0.53 | 0.05 | -1.98 | 0.12 | 0.20 |
| Q13492 | Phosphatidylinositol-binding clathrin assembly protein | PICALM | -0.72 | 1.87 | 0.38 | 0.70 | -0.06 | 0.42 | 0.48 | -1.18 | 1.76 | -1.70 | -0.04 | -0.76 |
| P37275 | Zinc finger E-box-binding homeobox 1 | ZEB1 | -0.72 | 1.74 | 2.77 | -0.13 | -0.52 | -0.14 | -0.16 | -0.12 | -0.27 | -0.87 | -0.34 | -0.23 |
| P31943 | Heterogeneous nuclear ribonucleoprotein H | HNRNPH1 | -0.72 | 1.53 | 1.47 | -1.03 | -0.93 | 0.81 | 1.27 | 0.08 | -0.68 | -1.36 | 0.53 | -0.17 |
| Q8IUE6 | Histone H2A type 2-B | HIST2H2AB | -0.72 | 0.48 | -0.20 | -1.66 | 1.36 | 0.66 | 0.47 | -0.24 | -1.67 | 0.00 | 0.65 | 0.63 |
| Q9BRK0 | Receptor expression-enhancing protein 2 | REEP2 | -0.72 | 1.37 | -1.08 | -0.16 | 0.29 | 0.16 | 2.27 | -0.10 | -1.57 | 0.10 | 0.22 | -0.12 |
| O60245 | Protocadherin-7 | PCDH7 | -0.72 | 1.50 | -0.05 | 2.80 | -0.59 | -0.22 | -0.30 | -0.26 | -0.30 | -0.62 | -0.30 | -0.16 |
| O75122 | CLIP-associating protein 2 | CLASP2 | -0.72 | 1.51 | -0.87 | 1.63 | 0.39 | -0.39 | 0.81 | -1.14 | 0.67 | -1.36 | -0.58 | 0.84 |
| Q12980 | GATOR complex protein NPRL3 | NPRL3 | -0.72 | 1.57 | 2.81 | -0.29 | -0.44 | -0.14 | -0.24 | -0.37 | -0.38 | -0.66 | -0.15 | -0.15 |
| P20042 | Eukaryotic translation initiation factor 2 subunit 2 | EIF2S2 | -0.72 | 2.66 | 0.23 | 0.83 | -0.15 | -0.09 | 1.50 | -1.20 | -1.25 | -0.94 | -0.35 | 1.41 |
| Q27J81 | Inverted formin-2 | INF2 | -0.72 | 1.79 | -0.50 | 2.17 | 0.17 | 0.58 | -0.63 | -0.44 | -1.27 | -0.98 | 0.69 | 0.23 |
| P22897 | Macrophage mannose receptor 1 | MRC1 | -0.72 | 0.97 | -0.35 | -0.55 | -0.81 | 1.89 | 0.95 | 1.35 | -0.54 | -0.86 | -0.61 | -0.48 |
| Q96GD0 | Pyridoxal phosphate phosphatase | PDXP | -0.72 | 3.30 | -0.43 | 1.20 | 0.03 | 1.11 | 0.71 | -1.95 | -0.91 | 0.04 | 0.80 | -0.61 |
| Q92843 | Bcl-2-like protein 2 | BCL2L2 | -0.73 | 0.80 | -0.26 | 0.81 | -1.49 | 1.11 | 0.80 | -0.05 | -1.38 | -1.04 | 0.87 | 0.64 |
| P12270 | Nucleoprotein TPR | TPR | -0.73 | 0.90 | 0.63 | -1.00 | 0.94 | -0.05 | 0.55 | -1.06 | 0.32 | 1.75 | -1.06 | -1.01 |

| | | | | | | | | | | | | | | |
|---|---|---|---|---|---|---|---|---|---|---|---|---|---|---|
| Q6VAB6 | Kinase suppressor of Ras 2 | KSR2 | -0.73 | 0.89 | 0.42 | 0.20 | -1.01 | 0.60 | 0.85 | 0.08 | -2.27 | 1.02 | 0.54 | -0.43 |
| O75530 | Polycomb protein EED | EED | -0.73 | 1.11 | -0.34 | 2.17 | -0.78 | -0.57 | 0.78 | -0.46 | 1.06 | -0.96 | -0.48 | -0.42 |
| Q96Q07 | BTB/POZ domain-containing protein 9 | BTBD9 | -0.73 | 0.73 | 0.06 | 0.71 | -1.39 | 1.19 | 0.32 | -1.22 | 0.76 | -1.53 | 0.66 | 0.44 |
| Q9Y6X4 | Soluble lamin-associated protein of 75 kDa | FAM169A | -0.73 | 2.88 | -0.05 | -0.48 | 1.17 | -0.41 | 2.31 | -0.29 | -0.19 | -1.18 | -0.43 | -0.45 |
| Q8NFA0 | Ubiquitin carboxyl-terminal hydrolase 32 | USP32 | -0.73 | 3.19 | 1.29 | 0.87 | 1.48 | -0.35 | -0.72 | -1.16 | -0.09 | 0.30 | -0.09 | -1.52 |
| Q6XE24 | RNA-binding motif, single-stranded-interacting protein 3 | RBMS3 | -0.73 | 1.02 | 1.21 | 0.46 | -1.06 | -0.95 | 1.53 | 0.36 | 0.52 | -1.36 | 0.16 | -0.84 |
| Q01650 | Large neutral amino acids transporter small subunit 1 | SLC7A5 | -0.73 | 1.10 | 0.70 | 1.50 | -1.80 | 0.24 | 0.62 | 0.52 | -0.90 | -1.20 | 0.07 | 0.25 |
| Q9NTU7 | Cerebellin-4 | CBLN4 | -0.73 | 0.71 | -1.33 | -0.21 | 0.34 | 0.87 | 1.19 | -0.87 | -1.73 | 0.23 | 0.80 | 0.70 |
| Q6ZMQ8 | Serine/threonine-protein kinase LMTK1 | AATK | -0.73 | 2.30 | 2.58 | 0.62 | -0.48 | -0.23 | -0.30 | -0.27 | -0.26 | -1.11 | -0.47 | -0.08 |
| Q8N3J6 | Cell adhesion molecule 2 | CADM2 | -0.73 | 2.51 | -0.44 | 2.20 | -0.66 | 1.17 | 0.02 | -0.31 | -0.87 | -1.03 | -0.38 | 0.31 |
| P28332 | Alcohol dehydrogenase 6 | ADH6 | -0.73 | 2.08 | -0.02 | -0.33 | 2.77 | -0.15 | -0.21 | -0.41 | -0.36 | -0.87 | -0.25 | -0.18 |
| Q13243 | Serine/arginine-rich splicing factor 5 | SRSF5 | -0.73 | 0.72 | 1.51 | -0.11 | -1.32 | 0.22 | 0.58 | 1.52 | -1.14 | -0.19 | -0.96 | -0.12 |
| Q00535 | Cyclin-dependent-like kinase 5 | CDK5 | -0.73 | 2.62 | 1.19 | 1.43 | -1.46 | 0.95 | 0.24 | -0.82 | -0.71 | -0.74 | 0.58 | -0.64 |
| Q9NQU5 | Serine/threonine-protein kinase PAK 6 | PAK6 | -0.73 | 1.78 | -0.05 | -0.17 | -0.47 | 2.76 | -0.22 | -0.23 | -0.24 | -0.94 | -0.22 | -0.22 |
| Q69YN2 | CWF19-like protein 1 | CWF19L1 | -0.73 | 0.77 | 1.51 | 0.20 | -1.35 | 0.51 | 0.06 | 1.21 | -0.43 | -1.10 | 0.57 | -1.19 |
| O96013 | Serine/threonine-protein kinase PAK 4 | PAK4 | -0.73 | 0.49 | -0.77 | 0.20 | 2.28 | -0.18 | -0.89 | 0.70 | 0.45 | -1.13 | -0.62 | -0.05 |
| Q2M1Z3 | Rho GTPase-activating protein 31 | ARHGAP31 | -0.73 | 1.78 | 0.06 | 2.74 | -0.50 | -0.20 | -0.25 | -0.30 | -0.11 | -0.96 | -0.33 | -0.16 |
| Q9Y4G8 | Rap guanine nucleotide exchange factor 2 | RAPGEF2 | -0.73 | 1.65 | -0.85 | 0.07 | 0.04 | -0.14 | 2.60 | -0.95 | -0.06 | -0.72 | -0.18 | 0.18 |
| Q9UL54 | Serine/threonine-protein kinase TAO2 | TAOK2 | -0.73 | 1.36 | 1.74 | -1.40 | 0.09 | 0.84 | 0.18 | 0.48 | 0.49 | -1.53 | -0.30 | -0.60 |
| Q13573 | SNW domain-containing protein 1 | SNW1 | -0.73 | 1.08 | -0.78 | 0.74 | 0.95 | 1.19 | -0.88 | -0.86 | -0.95 | -1.19 | 1.13 | 0.65 |
| Q13148 | TAR DNA-binding protein 43 | TARDBP | -0.73 | 2.10 | 0.77 | -0.61 | -0.59 | 1.24 | 1.16 | -0.51 | -1.34 | -1.33 | 0.96 | 0.25 |
| P49902 | Cytosolic purine 5'-nucleotidase | NT5C2 | -0.73 | 0.65 | -1.16 | -0.47 | 0.98 | 0.58 | 0.88 | -1.07 | -1.71 | 0.67 | 0.71 | 0.59 |
| Q8NF91 | Nesprin-1 | SYNE1 | -0.73 | 0.57 | -1.19 | 0.82 | 0.61 | 0.87 | -0.38 | -0.98 | -1.74 | 0.26 | 0.68 | 1.06 |
| O43865 | S-adenosylhomocysteine hydrolase-like protein 1 | AHCYL1 | -0.73 | 1.21 | -1.36 | -0.03 | 0.13 | 0.69 | 1.92 | -1.21 | -0.90 | -0.05 | 0.84 | -0.02 |
| P62805 | Histone H4 | HIST1H4A | -0.74 | 2.36 | 0.74 | 0.00 | 1.13 | 0.07 | 0.27 | 0.23 | -2.59 | -0.33 | 0.01 | 0.47 |
| Q9UIW2 | Plexin-A1 | PLXNA1 | -0.74 | 0.95 | -1.25 | 1.66 | 0.55 | -0.14 | 0.29 | -1.09 | 0.95 | -1.44 | 0.25 | 0.22 |
| O75955 | Flotillin-1 | FLOT1 | -0.74 | 0.92 | 0.76 | 0.90 | -1.73 | -0.10 | 1.24 | 0.53 | 0.72 | -1.23 | -0.29 | -0.82 |
| P10619 | Lysosomal protective protein | CTSA | -0.74 | 1.54 | 0.02 | -0.34 | -0.52 | -0.26 | 2.76 | -0.07 | -0.32 | -0.84 | -0.14 | -0.30 |
| O94822 | E3 ubiquitin-protein ligase listerin | LTN1 | -0.74 | 0.62 | 1.14 | 0.36 | -0.92 | 0.13 | 0.08 | 0.89 | 1.09 | -2.13 | -0.33 | -0.30 |
| P19878 | Neutrophil cytosol factor 2 | NCF2 | -0.74 | 1.58 | -0.18 | 1.72 | -0.80 | 1.59 | -0.67 | -0.55 | -0.54 | -1.10 | 0.74 | -0.20 |
| Q7Z7G0 | Target of Nesh-SH3 | ABI3BP | -0.74 | 0.98 | 0.44 | -0.16 | -0.42 | 1.18 | 0.12 | 0.84 | -1.48 | -1.84 | 0.72 | 0.61 |
| O14979 | Heterogeneous nuclear ribonucleoprotein D-like | HNRNPDL | -0.74 | 2.25 | 1.15 | -1.07 | 0.84 | 0.62 | 0.52 | -0.45 | -2.18 | -0.04 | 0.40 | 0.21 |
| Q13724 | Mannosyl-oligosaccharide glucosidase | MOGS | -0.74 | 0.92 | 1.18 | -0.50 | -0.69 | 1.69 | -0.60 | -0.53 | -0.58 | -0.84 | 1.42 | -0.55 |
| P61026 | Ras-related protein Rab-10 | RAB10 | -0.74 | 3.45 | -0.69 | 1.99 | 1.08 | 0.65 | -0.29 | -0.91 | -1.10 | -0.91 | 0.12 | 0.07 |
| Q5VZ66 | Janus kinase and microtubule-interacting protein 3 | JAKMIP3 | -0.74 | 1.96 | 0.10 | 2.72 | -0.58 | -0.04 | -0.22 | -0.22 | -0.16 | -0.96 | -0.35 | -0.28 |
| Q9NX63 | MICOS complex subunit MIC19 | CHCHD3 | -0.74 | 0.48 | -0.59 | 1.29 | -0.37 | 1.38 | -1.10 | -0.66 | -0.91 | -0.88 | 0.99 | 0.84 |
| P53597 | Succinate--CoA ligase [ADP/GDP-forming] subunit alpha, mitochon | SUCLG1 | -0.74 | 2.37 | 2.23 | 0.39 | 0.55 | -0.54 | -0.47 | 0.84 | -0.63 | -1.01 | -0.82 | -0.53 |
| Q13434 | Putative E3 ubiquitin-protein ligase makorin-4 | MKRN4P | -0.74 | 1.36 | 0.06 | 2.79 | -0.57 | -0.32 | -0.43 | -0.34 | -0.23 | -0.62 | -0.16 | -0.18 |
| P17677 | Neuromodulin | GAP43 | -0.74 | 1.26 | 0.16 | 0.95 | 0.66 | -1.30 | 0.90 | -0.48 | 0.81 | 0.62 | -1.93 | -0.40 |
| P08579 | U2 small nuclear ribonucleoprotein B'' | SNRPB2 | -0.74 | 0.95 | -0.56 | -1.04 | 0.96 | 1.04 | 0.71 | -1.36 | -1.22 | -0.35 | 0.87 | 0.94 |
| O76094 | Signal recognition particle subunit SRP72 | SRP72 | -0.74 | 0.78 | 0.65 | -0.26 | 0.82 | -0.63 | 0.38 | 0.21 | -0.43 | -1.82 | -0.71 | 1.80 |
| Q6ZSZ5 | Rho guanine nucleotide exchange factor 18 | ARHGEF18 | -0.74 | 1.67 | 0.07 | 2.75 | -0.46 | -0.15 | -0.45 | -0.25 | -0.18 | -0.91 | -0.18 | -0.24 |
| O75410 | Transforming acidic coiled-coil-containing protein 1 | TACC1 | -0.74 | 1.47 | -0.31 | 1.43 | 0.20 | 1.40 | -1.17 | -0.23 | -1.40 | -0.89 | 0.82 | 0.15 |
| P30531 | Sodium- and chloride-dependent GABA transporter 1 | SLC6A1 | -0.74 | 0.88 | -0.26 | 2.57 | -0.51 | -0.33 | -0.42 | -0.46 | -0.36 | -0.68 | -0.43 | 0.88 |
| A7KAX9 | Rho GTPase-activating protein 32 | ARHGAP32 | -0.74 | 0.54 | 0.93 | -0.16 | 0.29 | 0.26 | -0.61 | 1.24 | -0.66 | -2.25 | 0.25 | 0.72 |
| O94992 | Protein HEXIM1 | HEXIM1 | -0.75 | 1.62 | 2.78 | -0.31 | -0.48 | -0.10 | -0.17 | -0.16 | -0.30 | -0.83 | -0.34 | -0.09 |
| O95639 | Cleavage and polyadenylation specificity factor subunit 4 | CPSF4 | -0.75 | 1.52 | 2.76 | -0.21 | -0.63 | -0.09 | -0.19 | -0.11 | -0.34 | -0.82 | -0.13 | -0.25 |
| Q8WXF1 | Paraspeckle component 1 | PSPC1 | -0.75 | 2.16 | -0.42 | 0.41 | 0.87 | 0.61 | 0.62 | -1.49 | -1.97 | -0.08 | 0.78 | 0.67 |
| P61764 | Syntaxin-binding protein 1 | STXBP1 | -0.75 | 2.86 | -0.34 | 2.07 | 0.27 | 0.16 | 0.24 | -0.89 | 0.33 | -1.87 | -0.15 | 0.19 |
| Q99832 | T-complex protein 1 subunit eta | CCT7 | -0.75 | 4.34 | 1.24 | 0.36 | 1.34 | 0.55 | -0.49 | -0.80 | -1.84 | -0.41 | 0.64 | -0.61 |
| A4D161 | Protein FAM221A | FAM221A | -0.75 | 1.34 | 2.81 | -0.23 | -0.58 | -0.29 | -0.21 | -0.23 | -0.15 | -0.61 | -0.29 | -0.22 |
| Q8N3P4 | Vacuolar protein sorting-associated protein 8 homolog | VPS8 | -0.75 | 2.08 | 1.58 | -0.04 | -1.21 | 0.94 | 0.69 | -0.31 | -1.20 | -0.91 | 1.01 | -0.55 |
| O60315 | Zinc finger E-box-binding homeobox 2 | ZEB2 | -0.75 | 0.81 | 1.40 | -1.22 | -0.11 | 0.05 | 0.84 | 0.95 | -1.58 | -0.97 | 0.72 | -0.10 |
| Q9UPT6 | C-Jun-amino-terminal kinase-interacting protein 3 | MAPK8IP3 | -0.75 | 2.63 | 0.11 | 1.68 | 0.29 | 0.37 | -0.15 | -1.63 | 0.55 | -1.45 | -0.52 | 0.76 |
| Q8WUA2 | Peptidyl-prolyl cis-trans isomerase-like 4 | PPIL4 | -0.75 | 0.74 | 0.13 | 0.91 | -2.00 | 1.08 | 0.79 | -0.11 | -0.65 | -0.62 | 1.08 | -0.60 |
| Q6W3E5 | Glycerophosphodiester phosphodiesterase domain-containing prote | GDPD4 | -0.75 | 0.54 | -1.00 | 0.27 | 1.40 | 1.18 | -1.16 | -1.04 | -1.09 | 0.01 | 0.71 | 0.71 |
| P35222 | Catenin beta-1 | CTNNB1 | -0.75 | 2.69 | 0.06 | 1.11 | 0.96 | 0.75 | -0.55 | 0.05 | -0.59 | -2.26 | 0.66 | -0.19 |
| Q16653 | Myelin-oligodendrocyte glycoprotein | MOG | -0.75 | 0.80 | -0.08 | -0.30 | 1.54 | -0.01 | -0.18 | -0.29 | -2.04 | 1.47 | 0.16 | -0.26 |
| Q6UXB8 | Peptidase inhibitor 16 | PI16 | -0.75 | 2.88 | 0.06 | 0.92 | 2.40 | -0.30 | -0.55 | -0.23 | -0.47 | -1.19 | -0.53 | -0.11 |
| O94964 | Protein SOGA1 | SOGA1 | -0.75 | 1.03 | 0.67 | -0.92 | 0.78 | -0.76 | 1.41 | -0.80 | -0.73 | -1.24 | 1.32 | 0.28 |
| O60524 | Nuclear export mediator factor NEMF | NEMF | -0.75 | 1.27 | -0.48 | 1.34 | -0.50 | 1.27 | -0.25 | -1.44 | -1.44 | 0.20 | 0.74 | 0.56 |
| Q9H082 | Ras-related protein Rab-33B | RAB33B | -0.75 | 2.79 | -0.43 | 2.02 | -0.09 | 1.05 | -0.18 | -1.10 | -0.18 | 0.10 | 0.32 | -1.49 |
| Q14183 | Double C2-like domain-containing protein alpha | DOC2A | -0.75 | 1.54 | -0.15 | -0.48 | 0.29 | 0.68 | 1.28 | -1.24 | -1.40 | 1.26 | 0.73 | -0.97 |
| O95396 | Adenylyltransferase and sulfurtransferase MOCS3 | MOCS3 | -0.75 | 0.91 | 0.35 | 0.41 | -1.45 | 1.07 | 0.69 | 0.24 | -1.98 | -0.51 | 0.80 | 0.38 |
| Q9H1Z4 | WD repeat-containing protein 13 | WDR13 | -0.75 | 3.81 | 0.23 | 1.41 | -0.55 | 1.13 | 0.59 | -1.73 | 0.11 | -1.18 | 0.54 | -0.56 |
| Q9UDY4 | DnaJ homolog subfamily B member 4 | DNAJB4 | -0.75 | 0.79 | 0.15 | 0.82 | -0.03 | 0.25 | -0.21 | 0.27 | 1.27 | -2.55 | 0.03 | 0.02 |
| P62068 | Ubiquitin carboxyl-terminal hydrolase 46 | USP46 | -0.75 | 2.60 | -0.49 | 0.84 | 0.27 | 2.10 | -0.44 | -0.77 | -0.81 | -1.37 | 0.37 | 0.31 |
| Q99996 | A-kinase anchor protein 9 | AKAP9 | -0.76 | 0.48 | -1.19 | 1.42 | -0.26 | 0.50 | 0.16 | -2.00 | 0.09 | 1.07 | 0.27 | -0.05 |
| P06748 | Nucleophosmin | NPM1 | -0.76 | 1.69 | 0.38 | -1.59 | 0.38 | 1.04 | 1.49 | -0.34 | -1.58 | -0.09 | 0.47 | -0.17 |
| P00403 | Cytochrome c oxidase subunit 2 | MT-CO2 | -0.76 | 0.97 | 0.34 | 0.50 | 0.32 | 0.08 | -0.08 | 0.69 | -2.47 | -0.85 | 0.45 | 1.01 |
| Q5VZM2 | Ras-related GTP-binding protein B | RRAGB | -0.76 | 1.01 | 1.06 | 1.00 | -1.04 | 1.02 | -0.89 | -0.76 | -0.78 | -1.20 | 1.01 | 0.56 |
| Q9H0R8 | Gamma-aminobutyric acid receptor-associated protein-like 1 | GABARAPL1 | -0.76 | 2.78 | 0.42 | 2.17 | -0.19 | 0.25 | -0.30 | -1.22 | 0.85 | -0.77 | -0.16 | -1.05 |
| Q6P1L5 | Protein FAM117B | FAM117B | -0.76 | 1.55 | 0.01 | 2.79 | -0.49 | -0.21 | -0.42 | -0.32 | -0.34 | -0.67 | -0.16 | -0.18 |
| Q8TCY9 | Up-regulator of cell proliferation | URGCP | -0.76 | 1.52 | 1.18 | -0.75 | -0.86 | 1.64 | 0.36 | -0.64 | -0.60 | -1.12 | 1.14 | -0.37 |
| Q08174 | Protocadherin-1 | PCDH1 | -0.76 | 1.45 | -0.03 | 2.79 | -0.51 | -0.23 | -0.42 | -0.25 | -0.16 | -0.70 | -0.32 | -0.17 |
| Q86UA1 | Pre-mRNA-processing factor 39 | PRPF39 | -0.76 | 1.98 | 0.45 | 1.39 | -0.70 | 0.59 | 0.18 | -0.99 | -0.44 | -1.90 | 0.42 | 1.00 |
| Q9HBL0 | Tensin-1 | TNS1 | -0.76 | 1.00 | 0.87 | 0.56 | -0.61 | 0.60 | -0.24 | 0.62 | 0.57 | -2.53 | 0.01 | 0.16 |
| Q8NEZ5 | F-box only protein 22 | FBXO22 | -0.76 | 1.00 | 0.35 | -0.35 | 0.73 | 1.58 | -1.16 | 0.04 | -1.12 | 1.33 | -0.28 | -1.13 |
| O43251 | RNA binding protein fox-1 homolog 2 | RBFOX2 | -0.76 | 1.25 | 2.07 | -0.59 | -0.80 | -0.19 | 0.88 | 0.97 | -0.40 | -0.18 | -0.54 | -1.22 |
| Q9BVM4 | Gamma-glutamylaminecyclotransferase | GGACT | -0.76 | 0.89 | -0.82 | 0.46 | 1.03 | 0.08 | 0.31 | -1.09 | -1.03 | -1.33 | 0.95 | 1.44 |
| P11274 | Breakpoint cluster region protein | BCR | -0.76 | 3.29 | 0.55 | 0.82 | -0.61 | 1.53 | 0.32 | -0.08 | -0.57 | -1.98 | 0.70 | -0.68 |
| Q99726 | Zinc transporter 3 | SLC30A3 | -0.77 | 1.27 | 1.53 | 1.70 | -0.77 | -0.53 | -0.54 | -0.45 | -0.53 | -0.97 | 0.98 | -0.42 |
| Q5TBA9 | Protein furry homolog | FRY | -0.77 | 1.39 | 0.88 | 0.05 | -1.29 | 1.25 | 0.59 | -1.00 | -0.99 | 1.00 | 0.63 | -1.12 |

| | | | | | | | | | | | | | | |
|---|---|---|---|---|---|---|---|---|---|---|---|---|---|---|
| Q8N2M8 | CLK4-associating serine/arginine rich protein | CLASRP | -0.77 | 1.84 | 1.38 | 1.76 | -0.64 | -0.21 | -0.49 | -0.25 | 0.90 | -0.93 | -1.20 | -0.34 |
| Q11206 | CMP-N-acetylneuraminate-beta-galactosamide-alpha-2,3-sialyltran | ST3GAL4 | -0.77 | 1.08 | 0.15 | 0.18 | 0.31 | 0.57 | 0.06 | 0.47 | 0.75 | -2.60 | 0.75 | -0.64 |
| Q96QH2 | PML-RARA-regulated adapter molecule 1 | PRAM1 | -0.77 | 0.65 | 1.11 | 0.49 | -0.53 | 0.24 | -0.50 | 1.76 | -1.23 | -1.51 | -0.06 | 0.21 |
| P37840 | Alpha-synuclein | SNCA | -0.77 | 1.49 | 0.02 | 0.46 | -0.52 | -0.31 | 1.90 | -1.86 | 0.96 | 0.22 | -0.58 | -0.29 |
| O75177 | Calcium-responsive transactivator | SS18L1 | -0.77 | 1.64 | -0.39 | -0.38 | -0.81 | 1.44 | 1.82 | -0.57 | -0.61 | -1.04 | 0.87 | -0.33 |
| Q8TDY2 | RB1-inducible coiled-coil protein 1 | RB1CC1 | -0.77 | 2.27 | 1.68 | 1.27 | -1.29 | 0.73 | -0.27 | -0.24 | -0.30 | -1.45 | 0.08 | -0.20 |
| O14994 | Synapsin-3 | SYN3 | -0.77 | 1.67 | 1.06 | 1.50 | -0.68 | 0.31 | -0.50 | -0.22 | 1.24 | -1.61 | -0.36 | -0.74 |
| P05388 | 60S acidic ribosomal protein P0 | RPLP0 | -0.77 | 1.46 | -0.50 | 0.46 | -0.29 | 0.76 | 1.13 | -0.86 | -2.14 | 1.04 | 0.47 | -0.06 |
| Q96QD9 | UAP56-interacting factor | FYTTD1 | -0.77 | 1.26 | 0.61 | 0.50 | -1.41 | 0.83 | 0.83 | -0.37 | -2.06 | -0.06 | 0.64 | 0.48 |
| Q13459 | Unconventional myosin-IXb | MYO9B | -0.77 | 0.66 | -1.21 | 0.60 | 0.66 | 0.74 | 0.03 | -1.53 | -1.45 | 0.47 | 0.58 | 1.11 |
| P06681 | Complement C2 | C2 | -0.77 | 0.77 | -0.93 | 0.37 | 1.16 | 0.89 | -0.55 | -0.85 | -1.85 | 0.23 | 1.06 | 0.48 |
| Q9NRG4 | N-lysine methyltransferase SMYD2 | SMYD2 | -0.77 | 1.51 | 2.43 | -0.61 | -0.73 | 0.35 | 0.16 | 0.57 | -0.59 | -1.08 | -0.10 | -0.41 |
| P20336 | Ras-related protein Rab-3A | RAB3A | -0.77 | 2.72 | -0.55 | 0.95 | 0.94 | 0.63 | 0.40 | -2.47 | -0.17 | -0.25 | 0.32 | 0.20 |
| Q15773 | Myeloid leukemia factor 2 | MLF2 | -0.77 | 2.55 | 1.28 | 1.00 | -0.72 | 0.42 | 0.28 | 0.27 | 0.35 | -2.28 | -0.40 | -0.18 |
| Q96CV9 | Optineurin | OPTN | -0.77 | 2.37 | 0.66 | 1.92 | -1.23 | 0.82 | 0.00 | -0.52 | -0.74 | -1.29 | 0.45 | -0.07 |
| Q8TBB6 | Probable cationic amino acid transporter | SLC7A14 | -0.77 | 1.40 | -0.07 | 2.78 | -0.54 | -0.21 | -0.41 | -0.25 | -0.22 | -0.79 | -0.17 | -0.13 |
| Q99569 | Plakophilin-4 | PKP4 | -0.78 | 1.68 | 1.12 | 1.07 | -0.55 | 0.80 | -0.74 | 0.42 | -0.22 | -1.68 | 0.90 | -1.12 |
| P23258 | Tubulin gamma-1 chain | TUBG1 | -0.78 | 0.91 | 0.88 | 0.69 | -0.05 | 0.81 | -1.27 | -0.10 | -1.24 | -1.52 | 0.92 | 0.87 |
| Q96GX2 | Ataxin-7-like protein 3B | ATXN7L3B | -0.78 | 1.51 | 2.82 | -0.22 | -0.46 | -0.28 | -0.21 | -0.13 | -0.30 | -0.65 | -0.28 | -0.30 |
| P19532 | Transcription factor E3 | TFE3 | -0.78 | 1.65 | 0.40 | 0.83 | -1.61 | 0.93 | 1.13 | 1.06 | -0.45 | -0.29 | -0.98 | -1.02 |
| P08243 | Asparagine synthetase [glutamine-hydrolyzing] | ASNS | -0.78 | 2.63 | 0.80 | 1.12 | -0.30 | 0.98 | -0.30 | 0.01 | -1.27 | -1.81 | 1.01 | -0.23 |
| P11277 | Spectrin beta chain, erythrocytic | SPTB | -0.78 | 0.98 | 0.49 | 0.03 | 1.48 | -0.12 | -0.74 | 0.16 | 0.96 | -2.04 | -0.77 | 0.55 |
| A6NL88 | Protein shisa-7 | SHISA7 | -0.78 | 1.56 | 1.40 | 0.31 | 1.50 | -0.76 | -0.83 | 1.04 | -0.08 | -1.12 | -0.82 | -0.64 |
| Q16777 | Histone H2A type 2-C | HIST2H2AC | -0.78 | 1.06 | 0.49 | -0.38 | 1.30 | 0.66 | -0.88 | 0.21 | -2.21 | -0.04 | 0.86 | -0.03 |
| Q9H9B1 | Histone-lysine N-methyltransferase EHMT1 | EHMT1 | -0.78 | 1.43 | 0.19 | 0.37 | -1.41 | 0.68 | 1.68 | -1.16 | 0.24 | -0.08 | 0.75 | -1.26 |
| O75688 | Protein phosphatase 1B | PPM1B | -0.78 | 1.07 | -0.64 | 0.98 | 2.17 | -0.60 | -0.67 | -0.60 | -0.64 | -0.24 | -0.67 | 0.92 |
| Q8IXS8 | Protein FAM126B | FAM126B | -0.78 | 0.96 | -0.26 | 2.65 | -0.45 | -0.39 | -0.41 | -0.37 | -0.41 | -0.71 | -0.34 | 0.68 |
| O00168 | Phospholemman | FXYD1 | -0.78 | 1.60 | -0.07 | 2.80 | -0.44 | -0.16 | -0.41 | -0.28 | -0.19 | -0.69 | -0.36 | -0.21 |
| O00522 | Krev interaction trapped protein 1 | KRIT1 | -0.78 | 0.95 | 1.04 | -0.01 | -1.34 | 0.96 | 0.45 | 0.18 | -1.28 | -1.49 | 0.79 | 0.70 |
| Q9NXU5 | ADP-ribosylation factor-like protein 15 | ARL15 | -0.78 | 2.31 | -0.26 | 2.26 | 0.44 | 0.23 | -0.56 | -1.38 | 0.40 | -0.94 | 0.28 | -0.48 |
| Q9ULM3 | YEATS domain-containing protein 2 | YEATS2 | -0.78 | 0.59 | -1.05 | 0.56 | 1.56 | 0.78 | -1.09 | -1.12 | -1.06 | 0.32 | 0.91 | 0.20 |
| Q9H0W8 | Protein SMG9 | SMG9 | -0.78 | 1.40 | 0.08 | -0.94 | 0.89 | 1.14 | 0.33 | -1.00 | -1.01 | -1.37 | 1.30 | 0.58 |
| Q9P1U1 | Actin-related protein 3B | ACTR3B | -0.78 | 1.62 | -0.40 | 1.30 | -0.36 | 0.90 | 0.23 | -2.24 | 0.57 | -0.17 | 0.72 | -0.54 |
| Q8N3C7 | CAP-Gly domain-containing linker protein 4 | CLIP4 | -0.78 | 0.67 | -0.70 | -1.08 | 0.55 | 1.25 | 0.80 | -1.11 | -1.05 | -0.63 | 1.29 | 0.67 |
| Q96S44 | EKC/KEOPS complex subunit TP53RK | TP53RK | -0.78 | 1.19 | 1.04 | -0.36 | -0.71 | 1.20 | 0.16 | 0.14 | -1.00 | -1.85 | 1.14 | 0.25 |
| O14880 | Microsomal glutathione S-transferase 3 | MGST3 | -0.78 | 0.79 | -1.49 | 0.81 | -0.09 | 1.04 | 0.68 | -1.09 | -1.53 | 0.32 | 0.81 | 0.53 |
| O43307 | Rho guanine nucleotide exchange factor 9 | ARHGEF9 | -0.78 | 1.58 | -0.02 | -0.97 | 0.53 | 1.26 | 0.81 | -1.92 | -0.49 | -0.60 | 1.06 | 0.32 |
| Q96ME7 | Zinc finger protein 512 | ZNF512 | -0.78 | 0.99 | 1.49 | -1.02 | -0.92 | 0.54 | 1.04 | 0.31 | -1.67 | 0.21 | 0.55 | -0.54 |
| Q9Y6T7 | Diacylglycerol kinase beta | DGKB | -0.78 | 1.87 | 0.00 | 0.44 | -0.25 | 0.93 | 0.76 | -2.47 | -0.59 | -0.14 | 0.69 | 0.62 |
| Q9H0L4 | Cleavage stimulation factor subunit 2 tau variant | CSTF2T | -0.78 | 1.41 | -0.64 | 0.84 | -1.05 | 0.86 | 1.48 | -0.81 | -0.78 | -1.28 | 0.63 | 0.75 |
| Q52LJ0 | Protein FAM98B | FAM98B | -0.78 | 0.45 | -1.25 | 0.33 | 0.65 | 0.85 | 0.02 | -1.56 | -1.38 | 0.65 | 0.91 | 0.78 |
| Q9H009 | Nascent polypeptide-associated complex subunit alpha-2 | NACA2 | -0.78 | 3.72 | 1.05 | 1.01 | 0.67 | 0.45 | -0.32 | -1.07 | 0.01 | -1.57 | -1.23 | 1.00 |
| Q9UPR5 | Sodium/calcium exchanger 2 | SLC8A2 | -0.78 | 1.29 | -0.38 | 1.49 | 0.46 | 0.12 | -0.28 | -1.91 | -0.47 | 1.49 | 0.00 | -0.52 |
| Q8IX01 | SURP and G-patch domain-containing protein 2 | SUGP2 | -0.78 | 0.68 | 1.07 | -0.72 | -0.88 | 0.43 | 0.95 | 0.58 | 0.86 | -1.99 | 0.31 | -0.61 |
| Q7Z794 | Keratin, type II cytoskeletal 1b | KRT77 | -0.79 | 2.29 | 0.38 | -0.97 | 1.04 | -0.09 | 1.71 | -1.87 | -0.20 | -0.28 | 0.48 | -0.21 |
| Q96BY6 | Dedicator of cytokinesis protein 10 | DOCK10 | -0.79 | 0.97 | 0.87 | 0.58 | -0.64 | 0.50 | -0.17 | -1.10 | 1.64 | -1.33 | 0.68 | -1.03 |
| Q00653 | Nuclear factor NF-kappa-B p100 subunit | NFKB2 | -0.79 | 1.17 | -0.58 | 1.42 | -1.04 | 1.03 | 0.47 | -0.82 | -0.86 | -1.22 | 1.05 | 0.54 |
| Q9C0H5 | Rho GTPase-activating protein 39 | ARHGAP39 | -0.79 | 1.01 | -0.59 | 0.89 | -0.04 | 0.49 | 0.44 | -1.76 | 1.54 | -1.28 | 0.53 | -0.21 |
| Q9H0Q3 | FXYD domain-containing ion transport regulator 6 | FXYD6 | -0.79 | 1.67 | 0.00 | 2.77 | -0.46 | -0.17 | -0.37 | -0.12 | -0.25 | -0.83 | -0.35 | -0.21 |
| Q9BYJ9 | YTH domain-containing family protein 1 | YTHDF1 | -0.79 | 1.40 | 1.36 | -1.17 | -0.69 | 0.79 | 1.21 | 0.38 | 0.07 | -1.61 | 0.32 | -0.65 |
| Q92905 | COP9 signalosome complex subunit 5 | COPS5 | -0.79 | 2.92 | 0.71 | -0.46 | 0.63 | 1.60 | -0.05 | -0.61 | 0.44 | -0.82 | 0.52 | -1.96 |
| Q9H0B6 | Kinesin light chain 2 | KLC2 | -0.79 | 1.45 | 0.77 | 1.14 | -1.52 | 0.00 | 1.12 | 0.18 | -1.58 | 0.62 | 0.03 | -0.78 |
| Q9UKE5 | TRAF2 and NCK-interacting protein kinase | TNIK | -0.79 | 2.33 | 0.69 | 0.92 | -0.65 | 1.03 | 0.15 | 0.01 | -0.01 | -2.39 | 0.59 | -0.34 |
| O60518 | Ran-binding protein 6 | RANBP6 | -0.79 | 0.86 | -1.35 | 0.89 | -0.35 | 0.88 | 0.96 | 0.46 | -1.46 | -1.21 | 0.70 | 0.50 |
| P10915 | Hyaluronan and proteoglycan link protein 1 | HAPLN1 | -0.79 | 0.96 | -0.02 | -0.79 | 0.29 | -0.12 | 1.75 | -0.90 | -1.48 | 1.48 | -0.09 | -0.13 |
| O43566 | Regulator of G-protein signaling 14 | RGS14 | -0.79 | 1.25 | -0.04 | 1.64 | -1.69 | 0.95 | 0.53 | 0.13 | -1.04 | -0.79 | 0.67 | -0.36 |
| Q9H2U2 | Inorganic pyrophosphatase 2, mitochondrial | PPA2 | -0.79 | 0.71 | -1.45 | 0.47 | 0.24 | 0.64 | 0.98 | -0.99 | -1.69 | 1.00 | 0.61 | 0.20 |
| P27448 | MAP/microtubule affinity-regulating kinase 3 | MARK3 | -0.79 | 2.02 | -0.49 | 2.54 | -0.21 | 0.44 | -0.30 | -1.10 | 0.11 | -0.80 | 0.15 | -0.33 |
| O95425 | Supervillin | SVIL | -0.79 | 1.07 | -0.65 | 1.39 | 0.30 | 1.11 | -0.94 | 1.02 | -0.93 | -1.13 | -0.88 | 0.70 |
| P31942 | Heterogeneous nuclear ribonucleoprotein H3 | HNRNPH3 | -0.79 | 1.65 | 1.55 | -0.24 | -1.20 | 0.59 | 0.97 | 0.24 | -1.13 | -1.49 | 0.27 | 0.43 |
| P19525 | Interferon-induced, double-stranded RNA-activated protein kinase | EIF2AK2 | -0.80 | 1.38 | 1.23 | -0.63 | -0.92 | 0.61 | 1.18 | 0.38 | -0.52 | -1.96 | 0.40 | 0.24 |
| O14924 | Regulator of G-protein signaling 12 | RGS12 | -0.80 | 1.02 | -0.10 | 0.49 | -0.85 | 0.40 | 1.24 | -1.61 | -0.52 | -1.11 | 1.28 | 0.78 |
| O43920 | NADH dehydrogenase [ubiquinone] iron-sulfur protein 5 | NDUFS5 | -0.80 | 1.05 | -0.08 | -0.91 | 0.88 | -0.36 | 1.66 | -1.41 | -1.01 | 0.30 | -0.23 | 1.16 |
| Q15427 | Splicing factor 3B subunit 4 | SF3B4 | -0.80 | 1.29 | -0.36 | -0.66 | 0.86 | -0.61 | 2.18 | -0.60 | -0.64 | 1.04 | -0.64 | -0.58 |
| P57087 | Junctional adhesion molecule B | JAM2 | -0.80 | 1.09 | -0.59 | 0.56 | 0.92 | -0.26 | 0.62 | 0.53 | -1.87 | 0.24 | -1.34 | 1.19 |
| Q9P260 | RAB11-binding protein RELCH | RELCH | -0.80 | 0.73 | -1.17 | 1.22 | -0.16 | 0.89 | 0.11 | -1.25 | 1.03 | -1.49 | 0.66 | 0.16 |
| O43148 | mRNA cap guanine-N7 methyltransferase | RNMT | -0.80 | 0.92 | -0.73 | -1.13 | 0.93 | 1.21 | 0.79 | 0.79 | -1.17 | -1.39 | 0.24 | 0.46 |
| Q13542 | Eukaryotic translation initiation factor 4E-binding protein 2 | EIF4EBP2 | -0.80 | 0.77 | -0.75 | -0.87 | 0.75 | 0.95 | 0.85 | -0.89 | -0.97 | -1.20 | 0.91 | 1.22 |
| P04114 | Apolipoprotein B-100 | APOB | -0.80 | 0.40 | -1.59 | 0.61 | 0.50 | 0.71 | 0.31 | -0.83 | -1.75 | 0.48 | 0.75 | 0.83 |
| P49411 | Elongation factor Tu, mitochondrial | TUFM | -0.80 | 2.35 | 0.11 | -0.10 | 2.44 | -0.36 | 0.06 | -0.95 | 0.56 | -1.20 | -0.08 | -0.47 |
| Q9UQB8 | Brain-specific angiogenesis inhibitor 1-associated protein 2 | BAIAP2 | -0.80 | 1.78 | 1.47 | 0.45 | -0.29 | -0.31 | 0.47 | 0.62 | 0.78 | -2.17 | -0.41 | -0.60 |
| Q13136 | Liprin-alpha-1 | PPFIA1 | -0.80 | 0.89 | -0.71 | 1.63 | -0.92 | 0.49 | 0.55 | -0.54 | -1.36 | -0.81 | 1.21 | 0.45 |
| O00401 | Neural Wiskott-Aldrich syndrome protein | WASL | -0.80 | 1.27 | -0.76 | 1.29 | -0.52 | 1.13 | 0.24 | -0.24 | 0.56 | -1.92 | 0.84 | -0.63 |
| P08294 | Extracellular superoxide dismutase [Cu-Zn] | SOD3 | -0.80 | 1.22 | 0.86 | 0.25 | 0.97 | -1.04 | 0.31 | -0.96 | 0.22 | 1.24 | -1.89 | 0.03 |
| O15085 | Rho guanine nucleotide exchange factor 11 | ARHGEF11 | -0.80 | 1.88 | -0.20 | 1.35 | -0.11 | 0.57 | 0.26 | -0.86 | 0.45 | -2.30 | 0.14 | 0.70 |
| Q96JJ3 | Engulfment and cell motility protein 2 | ELMO2 | -0.80 | 1.14 | -1.54 | 1.62 | 0.33 | 0.67 | 0.20 | -1.06 | -1.22 | -0.22 | 0.54 | 0.67 |
| Q15843 | NEDD8 | NEDD8 | -0.80 | 1.30 | -0.91 | 1.44 | 0.22 | -0.22 | 0.88 | -1.69 | -0.07 | 1.33 | -0.26 | -0.73 |
| P20337 | Ras-related protein Rab-3B | RAB3B | -0.80 | 0.79 | 0.63 | 1.56 | -0.90 | 0.45 | -0.79 | 0.00 | 0.94 | -1.81 | 0.41 | -0.48 |
| P07910 | Heterogeneous nuclear ribonucleoproteins C1/C2 | HNRNPC | -0.80 | 2.00 | -0.58 | 1.37 | 0.34 | 0.60 | 0.21 | -0.36 | -2.37 | -0.18 | 0.60 | 0.37 |
| Q8WVY7 | Ubiquitin-like domain-containing CTD phosphatase 1 | UBLCP1 | -0.80 | 0.99 | 1.43 | 0.65 | -1.27 | 0.14 | 0.19 | 0.83 | -0.18 | -1.46 | 0.83 | -1.15 |
| P62760 | Visinin-like protein 1 | VSNL1 | -0.81 | 0.87 | -1.34 | -0.35 | 0.46 | 0.03 | 2.25 | -0.95 | -0.76 | 0.53 | 0.09 | 0.05 |
| P07199 | Major centromere autoantigen B | CENPB | -0.81 | 1.69 | -0.14 | -0.25 | -0.37 | 2.79 | -0.24 | -0.14 | -0.26 | -0.85 | -0.32 | -0.22 |
| Q7Z6J2 | General receptor for phosphoinositides 1-associated scaffold prot | GRASP | -0.81 | 1.57 | 2.81 | -0.19 | -0.48 | -0.21 | -0.23 | -0.25 | -0.21 | -0.67 | -0.29 | -0.29 |

| | | | | | | | | | | | | | | |
|---|---|---|---|---|---|---|---|---|---|---|---|---|---|---|
| P19387 | DNA-directed RNA polymerase II subunit RPB3 | POLR2C | -0.81 | 1.95 | 0.29 | 0.84 | -0.85 | 0.27 | 1.33 | 0.15 | -1.89 | -1.19 | 0.72 | 0.33 |
| P08574 | Cytochrome c1, heme protein, mitochondrial | CYC1 | -0.81 | 1.68 | -0.45 | 0.55 | -0.97 | 1.53 | 1.03 | -0.58 | -0.64 | -1.11 | 1.30 | -0.66 |
| P0DJI9 | Serum amyloid A-2 protein | SAA2 | -0.81 | 1.15 | -0.28 | 2.59 | 0.16 | -0.55 | -0.59 | -0.50 | 0.34 | -0.90 | -0.54 | 0.28 |
| Q9BRP8 | Partner of Y14 and mago | PYM1 | -0.81 | 1.25 | 2.05 | 0.53 | 0.66 | -0.82 | -1.04 | -0.32 | 0.52 | -0.78 | -1.12 | 0.31 |
| P25054 | Adenomatous polyposis coli protein | APC | -0.81 | 0.72 | 1.15 | 1.52 | -0.39 | -0.12 | -1.29 | 0.23 | 0.83 | -1.66 | -0.15 | -0.11 |
| O00429 | Dynamin-1-like protein | DNM1L | -0.81 | 1.01 | 0.41 | 0.61 | 0.24 | -0.13 | 0.06 | 0.67 | 1.01 | -2.62 | -0.04 | -0.21 |
| Q9BY44 | Eukaryotic translation initiation factor 2A | EIF2A | -0.81 | 1.76 | 0.73 | 0.07 | -0.76 | 1.06 | 0.68 | -0.91 | -1.10 | -1.60 | 1.04 | 0.80 |
| Q9Y618 | Nuclear receptor corepressor 2 | NCOR2 | -0.81 | 0.70 | 0.46 | 0.56 | 0.33 | 0.05 | -0.53 | -0.91 | -1.04 | -1.12 | -0.02 | 2.21 |
| P42226 | Signal transducer and activator of transcription 6 | STAT6 | -0.81 | 1.04 | 1.44 | -0.06 | -1.01 | 0.61 | 0.21 | 1.17 | -1.57 | -1.25 | 0.30 | 0.16 |
| Q7L099 | Protein RUFY3 | RUFY3 | -0.82 | 3.59 | 0.46 | 2.14 | -0.28 | 0.79 | -0.35 | -0.73 | -0.56 | -1.54 | 0.33 | -0.27 |
| O95208 | Epsin-2 | EPN2 | -0.82 | 1.13 | -0.74 | 1.02 | -0.92 | 0.55 | 1.36 | -1.03 | -1.03 | -0.90 | 1.02 | 0.67 |
| Q6PFW1 | Inositol hexakisphosphate and diphosphoinositol-pentakisphosphat | PPIP5K1 | -0.82 | 1.47 | 0.78 | 0.83 | -0.14 | 0.96 | -0.87 | -0.15 | -0.49 | -2.19 | 0.88 | 0.40 |
| Q9BZ72 | Membrane-associated phosphatidylinositol transfer protein 2 | PITPNM2 | -0.82 | 0.76 | 1.18 | 0.09 | -0.91 | 0.46 | 0.11 | 1.08 | 0.67 | -2.13 | 0.07 | -0.63 |
| O95294 | RasGAP-activating-like protein 1 | RASAL1 | -0.82 | 2.09 | 1.38 | -0.32 | -0.12 | 1.21 | -0.17 | -0.88 | 0.48 | -1.51 | 1.02 | -1.08 |
| Q6PH85 | DCN1-like protein 2 | DCUN1D2 | -0.82 | 1.16 | -0.34 | 1.16 | -0.70 | 1.80 | -0.62 | -0.57 | -0.57 | -0.93 | 1.28 | -0.50 |
| O14617 | AP-3 complex subunit delta-1 | AP3D1 | -0.82 | 3.62 | -0.33 | 1.65 | 1.35 | 0.11 | -0.03 | -1.49 | -0.51 | 0.60 | -0.11 | -1.23 |
| P45880 | Voltage-dependent anion-selective channel protein 2 | VDAC2 | -0.82 | 2.47 | 1.23 | 1.09 | -0.97 | 0.44 | 0.40 | 0.49 | -0.09 | -2.07 | -0.68 | 0.16 |
| Q8TF44 | C2 calcium-dependent domain-containing protein 4C | C2CD4C | -0.82 | 0.73 | 0.58 | 1.00 | -2.05 | 0.85 | 0.53 | -1.08 | 0.47 | -0.02 | 0.59 | -0.87 |
| Q13613 | Myotubularin-related protein 1 | MTMR1 | -0.82 | 1.04 | 1.06 | 1.21 | -0.53 | -0.09 | -0.46 | 0.95 | 0.62 | -2.10 | -0.42 | -0.25 |
| P29466 | Caspase-1 | CASP1 | -0.82 | 1.05 | -1.50 | 0.07 | 1.45 | 0.28 | 0.89 | -0.91 | -1.52 | 0.67 | 0.28 | 0.29 |
| Q8WZA9 | Immunity-related GTPase family Q protein | IRGQ | -0.82 | 1.90 | -0.78 | 1.04 | 1.06 | -0.44 | 0.95 | -2.11 | 0.09 | 0.68 | -0.38 | -0.13 |
| P49815 | Tuberin | TSC2 | -0.82 | 1.14 | 0.74 | 1.34 | -1.07 | 0.50 | -0.24 | 0.72 | 0.56 | -1.76 | 0.29 | -1.09 |
| Q9BV19 | Uncharacterized protein C1orf50 | C1orf50 | -0.82 | 0.72 | 0.67 | -0.84 | 0.64 | -0.88 | 1.29 | 0.90 | 1.13 | -1.16 | -0.92 | -0.84 |
| P30837 | Aldehyde dehydrogenase X, mitochondrial | ALDH1B1 | -0.82 | 1.76 | -0.02 | -0.20 | 2.78 | -0.31 | -0.40 | -0.28 | -0.19 | -0.85 | -0.33 | -0.18 |
| Q9Y2Y9 | Krueppel-like factor 13 | KLF13 | -0.83 | 1.86 | -0.07 | -0.18 | -0.45 | -0.16 | 2.76 | -0.34 | -0.21 | -0.90 | -0.37 | -0.09 |
| Q13263 | Transcription intermediary factor 1-beta | TRIM28 | -0.83 | 1.75 | 1.72 | -0.24 | -0.78 | 0.75 | 0.29 | 0.18 | -1.93 | -0.50 | 0.80 | -0.29 |
| Q8N7J2 | APC membrane recruitment protein 2 | AMER2 | -0.83 | 2.00 | 1.20 | 0.07 | 1.67 | 0.02 | -1.05 | -1.02 | -1.54 | 0.21 | 0.02 | 0.42 |
| P08247 | Synaptophysin | SYP | -0.83 | 0.56 | 0.40 | 1.50 | -2.06 | 0.54 | 0.34 | -0.45 | 0.64 | -1.10 | 0.30 | -0.10 |
| Q9Y5A7 | NEDD8 ultimate buster 1 | NUB1 | -0.83 | 2.19 | -0.50 | 0.29 | 0.12 | 1.30 | 0.84 | -1.31 | -1.71 | -0.57 | 1.02 | 0.50 |
| P17600 | Synapsin-1 | SYN1 | -0.83 | 2.42 | 0.50 | 1.55 | -0.27 | 0.29 | 0.13 | -1.60 | 1.20 | -1.37 | 0.10 | -0.53 |
| P16949 | Stathmin | STMN1 | -0.83 | 0.79 | -0.26 | 0.45 | -0.11 | -0.67 | 1.55 | -1.03 | -0.29 | 1.88 | -0.60 | -0.91 |
| O15079 | Syntaphilin | SNPH | -0.83 | 1.58 | 0.49 | 0.02 | 0.88 | 0.69 | -0.40 | 0.63 | -0.34 | -2.54 | -0.02 | 0.61 |
| Q16850 | Lanosterol 14-alpha demethylase | CYP51A1 | -0.83 | 1.13 | 0.85 | 0.59 | -1.68 | 1.13 | 0.37 | -0.29 | 0.20 | -1.80 | 0.05 | 0.58 |
| Q9H1V8 | Sodium-dependent neutral amino acid transporter SLC6A17 | SLC6A17 | -0.83 | 1.92 | -0.18 | 2.69 | -0.65 | -0.36 | 0.46 | -0.38 | -0.52 | -0.04 | -0.50 | -0.53 |
| O60256 | Phosphoribosyl pyrophosphate synthase-associated protein 2 | PRPSAP2 | -0.83 | 1.96 | -1.26 | 1.73 | 0.17 | 1.07 | 0.19 | -0.90 | -1.20 | -0.52 | 0.83 | -0.10 |
| P09012 | U1 small nuclear ribonucleoprotein A | SNRPA | -0.83 | 2.99 | -0.41 | 0.02 | 1.27 | 0.51 | 1.09 | -1.52 | -1.74 | 0.38 | 0.54 | -0.14 |
| Q86WR0 | Coiled-coil domain-containing protein 25 | CCDC25 | -0.83 | 0.55 | -1.39 | 0.23 | 0.86 | 0.50 | 0.50 | -1.31 | -1.55 | 0.97 | 0.60 | 0.60 |
| Q8N5H7 | SH2 domain-containing protein 3C | SH2D3C | -0.83 | 0.95 | 0.62 | 0.87 | -1.41 | 0.61 | 0.42 | 0.49 | 0.28 | -1.58 | 0.96 | -1.26 |
| Q15109 | Advanced glycosylation end product-specific receptor | AGER | -0.83 | 0.44 | 0.39 | 0.78 | 1.50 | -1.05 | -1.05 | 0.34 | 0.44 | -1.23 | -1.05 | 0.92 |
| Q96KR7 | Phosphatase and actin regulator 3 | PHACTR3 | -0.84 | 2.33 | 0.66 | 0.01 | -0.60 | -0.39 | 2.55 | -0.29 | -0.47 | -1.03 | -0.45 | 0.01 |
| P35612 | Beta-adducin | ADD2 | -0.84 | 4.00 | 0.65 | 2.32 | 0.24 | -0.09 | -0.16 | -1.06 | -1.25 | -0.51 | 0.22 | -0.35 |
| Q9Y2H0 | Disks large-associated protein 4 | DLGAP4 | -0.84 | 0.88 | 0.91 | 0.50 | -1.48 | 1.00 | 0.12 | -1.31 | -1.32 | 0.13 | 1.03 | 0.44 |
| P43378 | Tyrosine-protein phosphatase non-receptor type 9 | PTPN9 | -0.84 | 1.09 | -0.23 | 0.98 | -0.36 | 0.47 | 0.39 | -2.08 | -0.18 | -0.84 | 0.33 | 1.51 |
| Q9H1E3 | Nuclear ubiquitous casein and cyclin-dependent kinase substrate | NUCKS1 | -0.84 | 0.92 | 0.69 | 1.04 | 1.45 | -1.30 | -0.80 | -0.59 | 0.23 | 0.00 | -1.44 | 0.72 |
| O76070 | Gamma-synuclein | SNCG | -0.84 | 1.05 | 0.91 | 0.39 | 0.51 | -1.71 | 1.10 | 0.36 | 0.74 | 0.12 | -1.27 | -1.15 |
| P11137 | Microtubule-associated protein 2 | MAP2 | -0.84 | 1.24 | 0.96 | -0.18 | -0.76 | 0.84 | 0.50 | -0.69 | 1.42 | -1.77 | 0.48 | -0.80 |
| P84085 | ADP-ribosylation factor 5 | ARF5 | -0.84 | 2.36 | -0.73 | 1.39 | 0.35 | 0.56 | 0.57 | -2.04 | -1.02 | -0.13 | 0.52 | 0.53 |
| Q9UFC0 | Leucine-rich repeat and WD repeat-containing protein 1 | LRWD1 | -0.84 | 1.39 | 1.74 | -0.66 | -0.92 | -0.26 | 1.61 | -0.35 | -0.59 | -1.02 | -0.22 | 0.68 |
| Q5VVQ6 | Ubiquitin thioesterase OTU1 | YOD1 | -0.84 | 1.74 | 0.28 | 0.85 | 0.93 | 0.57 | -0.87 | -1.09 | 0.79 | -2.00 | 0.66 | -0.11 |
| Q16799 | Reticulon-1 | RTN1 | -0.84 | 4.19 | 0.24 | 0.07 | 1.65 | 0.80 | 0.24 | -2.13 | 0.27 | -0.35 | 0.10 | -0.88 |
| Q8N5T2 | TBC1 domain family member 19 | TBC1D19 | -0.84 | 1.36 | 1.84 | -0.48 | -0.77 | -0.50 | 1.37 | 1.00 | -0.53 | -0.94 | -0.55 | -0.44 |
| Q6P1X5 | Transcription initiation factor TFIID subunit 2 | TAF2 | -0.85 | 1.80 | -0.10 | -0.17 | -0.44 | -0.19 | 2.77 | -0.37 | -0.17 | -0.88 | -0.13 | -0.32 |
| Q9NYS0 | NF-kappa-B inhibitor-interacting Ras-like protein 1 | NKIRAS1 | -0.85 | 0.81 | 0.79 | 0.32 | -1.69 | 1.11 | 0.44 | -0.11 | 0.14 | -1.83 | 0.78 | 0.04 |
| O75912 | Diacylglycerol kinase iota | DGKI | -0.85 | 3.99 | 0.84 | 1.75 | 0.74 | 0.84 | -1.15 | -0.65 | -0.26 | -1.29 | -0.13 | -0.70 |
| Q9Y2K5 | R3H domain-containing protein 2 | R3HDM2 | -0.85 | 0.96 | 0.56 | 0.00 | -1.80 | 0.45 | 1.92 | -0.66 | -0.92 | 0.29 | 0.39 | -0.23 |
| Q9HCE3 | Zinc finger protein 532 | ZNF532 | -0.85 | 1.08 | -0.59 | -0.74 | 1.05 | 0.10 | 1.40 | -0.60 | -1.98 | 0.53 | 0.18 | 0.65 |
| Q96LT7 | Guanine nucleotide exchange C9orf72 | C9orf72 | -0.85 | 1.00 | -0.82 | 1.39 | -0.99 | 1.19 | 0.39 | -0.95 | -0.91 | -0.78 | 1.19 | 0.30 |
| Q96E39 | RNA binding motif protein, X-linked-like-1 | RBMXL1 | -0.85 | 1.20 | 0.99 | 0.64 | -1.51 | 0.60 | 0.61 | -1.25 | -0.28 | -1.34 | 0.79 | 0.75 |
| P25713 | Metallothionein-3 | MT3 | -0.85 | 0.71 | -0.86 | 0.48 | -0.28 | 0.70 | 0.83 | -1.54 | -1.45 | 1.42 | 0.44 | 0.25 |
| Q7OIA6 | MOB kinase activator 2 | MOB2 | -0.85 | 3.58 | -0.01 | 1.58 | -0.44 | 1.02 | 0.57 | -1.23 | -1.79 | -0.18 | 0.42 | 0.05 |
| Q8NHG7 | Small VCP/p97-interacting protein | SVIP | -0.85 | 0.54 | -1.75 | 0.53 | 1.01 | 0.37 | 0.54 | -1.80 | -0.37 | 0.39 | 0.38 | 0.71 |
| Q9NQW6 | Anillin | ANLN | -0.85 | 1.15 | 0.69 | -1.83 | 0.32 | 0.05 | 2.09 | -0.33 | -0.26 | -0.15 | 0.11 | -0.70 |
| Q9Y6J0 | Calcineurin-binding protein cabin-1 | CABIN1 | -0.85 | 1.25 | 0.97 | 1.66 | -0.96 | 0.47 | -0.78 | 0.96 | -0.72 | -1.17 | 0.35 | -0.79 |
| Q9UHP3 | Ubiquitin carboxyl-terminal hydrolase 25 | USP25 | -0.85 | 1.62 | 0.52 | 1.74 | -0.53 | 0.12 | -0.21 | -1.52 | 0.08 | 0.79 | 0.47 | -1.48 |
| O14772 | Fucose-1-phosphate guanylyltransferase | FPGT | -0.85 | 1.05 | -1.25 | 0.42 | 0.07 | 0.94 | 1.01 | -1.45 | -1.43 | 0.17 | 0.87 | 0.64 |
| P00367 | Glutamate dehydrogenase 1, mitochondrial | GLUD1 | -0.85 | 2.79 | -0.69 | -0.27 | 1.81 | 0.35 | 1.16 | -1.85 | 0.05 | -0.10 | 0.05 | -0.51 |
| Q16623 | Syntaxin-1A | STX1A | -0.85 | 2.42 | -0.26 | 0.80 | -0.29 | -0.12 | 2.04 | -1.76 | 0.67 | -0.44 | -0.08 | -0.54 |
| Q8IZD0 | Sterile alpha motif domain-containing protein 14 | SAMD14 | -0.85 | 0.99 | -0.15 | -0.17 | -0.52 | 0.76 | 1.22 | 0.05 | -2.39 | 0.78 | 0.51 | -0.09 |
| P36915 | Guanine nucleotide-binding protein-like 1 | GNL1 | -0.85 | 2.61 | 0.98 | 1.38 | 0.06 | 0.46 | -0.61 | 0.02 | 0.55 | -2.08 | 0.17 | -0.94 |
| Q96J87 | CUGBP Elav-like family member 6 | CELF6 | -0.85 | 0.75 | -0.24 | -0.67 | -0.68 | 1.14 | 1.36 | -1.02 | 1.12 | -1.27 | 0.81 | -0.56 |
| Q8WW38 | Zinc finger protein ZFPM2 | ZFPM2 | -0.85 | 0.81 | -0.48 | 1.33 | -0.67 | 1.40 | -0.61 | -0.56 | -0.57 | -0.85 | 1.59 | -0.58 |
| Q9BXI3 | Cytosolic 5'-nucleotidase 1A | NT5C1A | -0.85 | 1.15 | 1.08 | 0.76 | -1.43 | 1.03 | -0.16 | 0.02 | -1.11 | -1.44 | 0.68 | 0.58 |
| Q9BV44 | THUMP domain-containing protein 3 | THUMPD3 | -0.85 | 1.31 | -0.01 | 1.75 | -0.64 | 1.00 | -0.68 | -0.43 | -0.84 | -1.31 | 1.20 | -0.04 |
| O15047 | Histone-lysine N-methyltransferase SETD1A | SETD1A | -0.85 | 1.23 | 1.76 | -0.89 | -1.01 | 0.59 | 0.91 | -0.33 | 0.42 | -1.19 | 0.65 | -0.90 |
| Q5T8I3 | Protein FAM102B | FAM102B | -0.85 | 1.12 | 1.33 | 0.89 | -1.10 | 0.19 | -0.05 | 1.48 | -0.94 | -1.15 | 0.20 | -0.86 |
| Q15287 | RNA-binding protein with serine-rich domain 1 | RNPS1 | -0.85 | 2.07 | -0.07 | 0.23 | 1.08 | 0.99 | -0.24 | -1.95 | -0.99 | -0.62 | 0.38 | 1.19 |
| Q9BXS5 | AP-1 complex subunit mu-1 | AP1M1 | -0.86 | 1.83 | -0.10 | 1.75 | -0.04 | 0.88 | -0.70 | -0.72 | -0.24 | -1.82 | 0.87 | 0.12 |
| Q8WZA2 | Rap guanine nucleotide exchange factor 4 | RAPGEF4 | -0.86 | 1.64 | 0.70 | 0.90 | -1.79 | 0.78 | 1.09 | -0.81 | -1.40 | 0.09 | 0.17 | 0.26 |
| P21281 | V-type proton ATPase subunit B, brain isoform | ATP6V1B2 | -0.86 | 3.44 | 0.31 | 0.83 | 1.37 | 0.59 | -0.39 | -1.74 | -0.56 | 0.17 | 0.79 | -1.36 |
| Q6IQ19 | Centriole, cilia and spindle-associated protein | CCSAP | -0.86 | 2.11 | 2.48 | 1.02 | -0.67 | -0.24 | -0.49 | -0.35 | -0.37 | -0.73 | -0.43 | -0.23 |
| Q92871 | Phosphomannomutase 1 | PMM1 | -0.86 | 0.90 | 0.19 | -0.32 | 0.40 | 1.41 | -0.62 | 0.18 | 0.65 | 1.00 | -0.84 | -2.04 |
| P21695 | Glycerol-3-phosphate dehydrogenase [NAD(+)], cytoplasmic | GPD1 | -0.86 | 1.47 | -1.07 | 0.99 | 1.04 | -0.07 | 0.66 | -1.27 | -0.47 | 1.35 | 0.14 | -1.29 |
| Q9UKT7 | F-box/LRR-repeat protein 3 | FBXL3 | -0.86 | 1.61 | -0.12 | -0.28 | -0.54 | -0.13 | 2.81 | -0.21 | -0.29 | -0.62 | -0.29 | -0.33 |

| | | | | | | | | | | | | | | |
|---|---|---|---|---|---|---|---|---|---|---|---|---|---|---|
| O75147 | Obscurin-like protein 1 | OBSL1 | -0.86 | 1.27 | -0.83 | -0.17 | 0.02 | 0.57 | 1.79 | -2.07 | -0.11 | -0.08 | 0.60 | 0.27 |
| P08311 | Cathepsin G | CTSG | -0.86 | 0.78 | 1.18 | 0.00 | -0.62 | 0.45 | -0.05 | 1.34 | -1.53 | 0.33 | -1.59 | 0.49 |
| Q9GZY8 | Mitochondrial fission factor | MFF | -0.86 | 2.65 | 0.29 | -0.12 | -0.48 | 0.94 | 1.65 | -0.28 | -0.41 | -2.16 | 0.38 | 0.17 |
| O95817 | BAG family molecular chaperone regulator 3 | BAG3 | -0.86 | 1.62 | -0.34 | 1.96 | -0.79 | 1.47 | -0.61 | -0.60 | -0.48 | 0.67 | -0.65 | -0.64 |
| Q9BQE5 | Apolipoprotein L2 | APOL2 | -0.87 | 1.52 | -0.46 | 1.70 | -0.43 | 0.66 | 0.10 | -0.24 | 0.00 | -2.19 | 0.28 | 0.57 |
| Q9H981 | Actin-related protein 8 | ACTR8 | -0.87 | 0.61 | -1.38 | 0.32 | 0.72 | 0.71 | 0.41 | -1.43 | -1.43 | 0.97 | 0.84 | 0.28 |
| Q8IZ69 | tRNA (uracil-5-)-methyltransferase homolog A | TRMT2A | -0.87 | 0.99 | 0.43 | -0.65 | -0.78 | 0.57 | 1.56 | -0.65 | 1.70 | -0.97 | -0.61 | -0.61 |
| Q7L5D6 | Golgi to ER traffic protein 4 homolog | GET4 | -0.87 | 2.24 | 1.19 | -0.22 | -0.76 | 0.46 | 1.38 | 0.07 | -0.10 | -2.16 | 0.32 | -0.19 |
| Q99081 | Transcription factor 12 | TCF12 | -0.87 | 1.75 | -0.10 | -0.27 | -0.46 | -0.15 | 2.82 | -0.29 | -0.29 | -0.59 | -0.34 | -0.33 |
| O14594 | Neurocan core protein | NCAN | -0.87 | 1.12 | 0.08 | -0.86 | 0.61 | -0.28 | 1.70 | -1.09 | -1.10 | 1.50 | -0.07 | -0.50 |
| Q53GL7 | Protein mono-ADP-ribosyltransferase PARP10 | PARP10 | -0.87 | 2.50 | -0.22 | 1.52 | 0.33 | 1.08 | -0.51 | -2.23 | 0.04 | -0.25 | -0.01 | 0.24 |
| Q15311 | RalA-binding protein 1 | RALBP1 | -0.87 | 1.28 | 1.74 | 1.05 | -0.47 | -0.25 | -0.68 | 0.76 | 0.65 | -1.47 | -0.43 | -0.90 |
| P20339 | Ras-related protein Rab-5A | RAB5A | -0.87 | 1.05 | 2.33 | -0.01 | -1.38 | -0.29 | 0.58 | 0.03 | -0.96 | 0.30 | -0.45 | -0.15 |
| O75382 | Tripartite motif-containing protein 3 | TRIM3 | -0.87 | 1.99 | 0.84 | 1.45 | -0.96 | 0.69 | -0.12 | -0.13 | -0.02 | -2.01 | 0.72 | -0.46 |
| P20591 | Interferon-induced GTP-binding protein Mx1 | MX1 | -0.87 | 1.27 | 0.55 | 1.65 | -0.81 | 0.29 | -0.30 | 0.55 | -1.65 | -1.27 | 0.42 | 0.58 |
| Q9Y5L0 | Transportin-3 | TNPO3 | -0.87 | 1.79 | -0.94 | 0.43 | -0.19 | 1.03 | 1.44 | -1.07 | -1.63 | 0.41 | 0.83 | -0.31 |
| Q9NP81 | Serine--tRNA ligase, mitochondrial | SARS2 | -0.87 | 1.57 | -0.90 | 0.20 | 1.97 | -0.11 | 0.45 | -1.67 | 0.58 | -0.89 | 0.36 | 0.01 |
| Q5VWQ0 | Lysine-specific demethylase 9 | RSBN1 | -0.87 | 1.47 | -0.09 | -0.21 | -0.52 | -0.35 | 2.79 | -0.22 | -0.18 | -0.78 | -0.28 | -0.14 |
| Q52LD8 | Raftlin-2 | RFTN2 | -0.87 | 1.48 | -0.33 | 1.75 | -0.86 | 1.53 | -0.54 | -0.50 | -0.46 | -0.98 | 0.88 | -0.51 |
| O75781 | Paralemmin-1 | PALM | -0.87 | 2.36 | -0.03 | 2.09 | -0.57 | 0.39 | 0.24 | -1.19 | -1.56 | -0.06 | 0.40 | 0.29 |
| Q9H2M9 | Rab3 GTPase-activating protein non-catalytic subunit | RAB3GAP2 | -0.87 | 1.32 | -0.11 | 0.69 | -0.03 | 0.23 | 0.69 | -0.86 | -2.12 | -0.32 | 0.23 | 1.60 |
| Q14289 | Protein-tyrosine kinase 2-beta | PTK2B | -0.88 | 5.70 | 1.06 | -0.30 | 0.49 | 0.34 | 1.85 | -1.22 | -0.88 | 0.44 | -0.78 | -0.98 |
| Q9P244 | Leucine-rich repeat and fibronectin type III domain-containing pro | LRFN1 | -0.88 | 1.47 | -0.09 | 2.79 | -0.52 | -0.28 | -0.29 | -0.13 | -0.33 | -0.74 | -0.22 | -0.19 |
| P62333 | 26S proteasome regulatory subunit 10B | PSMC6 | -0.88 | 2.36 | -0.18 | 1.54 | 0.06 | 0.36 | 0.39 | 0.29 | -1.66 | -1.68 | 0.81 | 0.07 |
| Q7Z5L9 | Interferon regulatory factor 2-binding protein 2 | IRF2BP2 | -0.88 | 1.03 | -0.39 | -0.54 | -0.78 | 1.59 | 1.31 | -0.57 | -0.51 | -0.89 | 1.40 | -0.60 |
| Q86V48 | Leucine zipper protein 1 | LUZP1 | -0.88 | 0.84 | 0.92 | 0.14 | -1.49 | 0.68 | 0.74 | 0.69 | -2.05 | -0.18 | 0.42 | 0.12 |
| P55795 | Heterogeneous nuclear ribonucleoprotein H2 | HNRNPH2 | -0.88 | 1.60 | 1.43 | -0.90 | -1.00 | 1.03 | 1.10 | -0.46 | -0.95 | -1.02 | 0.84 | -0.06 |
| Q6ZWB6 | BTB/POZ domain-containing protein KCTD8 | KCTD8 | -0.88 | 0.47 | -1.04 | 0.86 | 1.11 | 0.87 | -1.19 | -1.14 | 0.17 | 0.50 | 0.97 | -1.11 |
| Q9UHC7 | E3 ubiquitin-protein ligase makorin-1 | MKRN1 | -0.88 | 1.62 | -0.50 | 1.31 | -0.29 | 0.60 | 0.55 | -2.37 | -0.42 | 0.58 | 0.25 | 0.30 |
| P21980 | Protein-glutamine gamma-glutamyltransferase 2 | TGM2 | -0.88 | 1.12 | -0.99 | 2.44 | -0.43 | -0.38 | 0.64 | -1.08 | -0.20 | 0.25 | -0.01 | -0.25 |
| Q8IXQ6 | Protein mono-ADP-ribosyltransferase PARP9 | PARP9 | -0.88 | 0.48 | -1.25 | -0.01 | 0.50 | 0.86 | 0.52 | -1.83 | -1.03 | 0.84 | 0.92 | 0.47 |
| P05455 | Lupus La protein | SSB | -0.88 | 1.29 | -0.40 | 0.60 | 0.32 | -0.01 | 0.92 | 0.07 | -2.61 | 0.81 | 0.06 | 0.23 |
| Q17RY0 | Cytoplasmic polyadenylation element-binding protein 4 | CPEB4 | -0.89 | 1.60 | -0.13 | 0.20 | -1.19 | 0.82 | 1.95 | -1.49 | 0.27 | 0.30 | 0.08 | -0.80 |
| Q8IWQ3 | Serine/threonine-protein kinase BRSK2 | BRSK2 | -0.89 | 0.86 | -1.78 | 0.97 | 0.34 | 0.35 | 1.15 | -1.69 | -0.17 | 0.61 | 0.24 | -0.02 |
| Q9Y5M8 | Signal recognition particle receptor subunit beta | SRPRB | -0.89 | 0.87 | 0.87 | 0.33 | -1.70 | 0.25 | 1.28 | 0.62 | 0.00 | -1.76 | -0.09 | 0.20 |
| O43172 | U4/U6 small nuclear ribonucleoprotein Prp4 | PRPF4 | -0.89 | 0.78 | -0.97 | 1.44 | 0.10 | 0.38 | -0.01 | -1.08 | -1.82 | 0.38 | 0.81 | 0.77 |
| Q92947 | Glutaryl-CoA dehydrogenase, mitochondrial | GCDH | -0.89 | 0.72 | -1.44 | 0.65 | 0.73 | 0.40 | 0.55 | -1.43 | -1.45 | 0.82 | 0.61 | 0.57 |
| P25440 | Bromodomain-containing protein 2 | BRD2 | -0.89 | 1.87 | 0.42 | 2.71 | -0.56 | -0.16 | -0.48 | -0.27 | -0.24 | -0.73 | -0.40 | -0.29 |
| Q9NQX3 | Gephyrin | GPHN | -0.89 | 1.74 | 0.08 | 0.74 | -0.09 | 0.30 | 0.77 | -0.56 | -2.57 | 0.70 | 0.57 | 0.04 |
| Q9BUE6 | Iron-sulfur cluster assembly 1 homolog, mitochondrial | ISCA1 | -0.89 | 1.35 | 0.82 | 0.64 | 0.11 | 1.19 | -1.32 | 1.07 | 0.40 | -1.60 | -0.62 | -0.69 |
| Q9BY43 | Charged multivesicular body protein 4a | CHMP4A | -0.89 | 0.88 | -0.20 | 0.57 | 1.22 | -1.35 | 0.80 | -1.28 | -1.38 | 0.12 | 0.83 | 0.67 |
| Q9Y5F6 | Protocadherin gamma-C5 | PCDHGC5 | -0.89 | 1.40 | -0.54 | 1.45 | -0.80 | 0.92 | 0.46 | -0.56 | -0.67 | -1.09 | -0.73 | 1.56 |
| Q9BX68 | Histidine triad nucleotide-binding protein 2, mitochondrial | HINT2 | -0.89 | 1.75 | 1.45 | 0.42 | 1.02 | 0.42 | -1.56 | -1.45 | 0.67 | 0.01 | -0.36 | -0.62 |
| O94953 | Lysine-specific demethylase 4B | KDM4B | -0.89 | 1.26 | -1.60 | 1.92 | 0.00 | 0.53 | 0.54 | -0.03 | -1.14 | -0.83 | 0.40 | 0.21 |
| Q8WVK2 | U4/U6.U5 small nuclear ribonucleoprotein 27 kDa protein | SNRNP27 | -0.89 | 0.85 | 1.76 | -1.32 | 0.65 | 0.03 | -0.10 | 1.06 | -1.47 | -0.60 | 0.18 | -0.18 |
| Q14165 | Malectin | MLEC | -0.89 | 1.08 | -0.67 | -0.42 | 0.26 | 0.89 | 1.17 | -0.10 | 0.26 | -2.33 | 0.79 | 0.15 |
| Q53GS9 | U4/U6.U5 tri-snRNP-associated protein 2 | USP39 | -0.89 | 1.61 | 0.59 | 0.56 | 1.22 | 0.68 | -1.41 | -1.27 | -1.22 | -0.11 | 1.12 | -0.17 |
| P16157 | Ankyrin-1 | ANK1 | -0.89 | 2.15 | -0.25 | 1.66 | -0.53 | 0.74 | 0.38 | -0.98 | -0.13 | -1.90 | 0.74 | 0.27 |
| Q643R3 | Lysophospholipid acyltransferase LPCAT4 | LPCAT4 | -0.89 | 1.20 | 1.04 | 0.26 | -0.61 | -0.12 | 0.77 | 0.15 | 0.79 | -2.48 | -0.03 | 0.23 |
| Q99471 | Prefoldin subunit 5 | PFDN5 | -0.90 | 1.88 | -0.08 | -0.15 | -0.48 | -0.15 | 2.79 | -0.22 | -0.24 | -0.73 | -0.40 | -0.34 |
| Q96E17 | Ras-related protein Rab-3C | RAB3C | -0.90 | 1.44 | 0.26 | 1.44 | 0.42 | -0.41 | -0.18 | -0.61 | 1.37 | -2.03 | 0.06 | -0.32 |
| Q9UHW9 | Solute carrier family 12 member 6 | SLC12A6 | -0.90 | 1.77 | 0.61 | 2.38 | -0.88 | 0.42 | -0.71 | 0.27 | -0.46 | -0.93 | -0.11 | -0.59 |
| P00519 | Tyrosine-protein kinase ABL1 | ABL1 | -0.90 | 1.61 | -0.01 | 2.80 | -0.44 | -0.19 | -0.43 | -0.18 | -0.30 | -0.68 | -0.23 | -0.34 |
| O95782 | AP-2 complex subunit alpha-1 | AP2A1 | -0.90 | 0.86 | -1.24 | 0.34 | 1.65 | 0.60 | -0.32 | -1.28 | -1.16 | 0.83 | 0.69 | -0.10 |
| Q96K21 | Abscission/NoCut checkpoint regulator | ZFYVE19 | -0.90 | 1.26 | 1.32 | 1.39 | -1.14 | 0.07 | -0.27 | 0.77 | -0.84 | -1.59 | 0.31 | -0.03 |
| Q5T5U3 | Rho GTPase-activating protein 21 | ARHGAP21 | -0.90 | 1.17 | 1.71 | 0.67 | -0.38 | 0.39 | -1.09 | 0.75 | 0.53 | -1.58 | -0.10 | -0.89 |
| Q9NQ86 | E3 ubiquitin-protein ligase TRIM36 | TRIM36 | -0.90 | 2.66 | 0.08 | -0.33 | 2.23 | 0.22 | 0.10 | -1.24 | -1.37 | 0.18 | 0.52 | -0.37 |
| Q6ZS17 | Rho family-interacting cell polarization regulator 1 | RIPOR1 | -0.90 | 0.70 | -0.75 | 1.13 | -0.97 | 1.17 | 0.30 | -0.85 | -0.90 | -1.12 | 1.00 | 1.00 |
| Q6IQ22 | Ras-related protein Rab-12 | RAB12 | -0.90 | 3.77 | -0.07 | 1.19 | 0.59 | -0.77 | 1.94 | -1.31 | 0.11 | -0.33 | -0.92 | -0.44 |
| P53667 | LIM domain kinase 1 | LIMK1 | -0.90 | 0.93 | -0.89 | -0.26 | 0.65 | 0.47 | 1.13 | -1.58 | -1.49 | 1.02 | 0.59 | 0.36 |
| O15269 | Serine palmitoyltransferase 1 | SPTLC1 | -0.90 | 1.17 | 0.87 | -0.84 | 0.89 | 1.33 | -0.95 | -0.86 | -0.85 | -1.11 | 1.08 | 0.44 |
| O60678 | Protein arginine N-methyltransferase 3 | PRMT3 | -0.91 | 0.90 | 0.52 | 0.63 | -0.66 | 0.61 | -0.02 | 0.76 | -0.95 | -2.26 | 0.66 | 0.71 |
| P62837 | Ubiquitin-conjugating enzyme E2 D2 | UBE2D2 | -0.91 | 1.01 | -0.61 | 1.79 | 1.44 | -0.70 | -0.76 | -0.66 | -0.60 | -0.93 | 0.76 | 0.25 |
| P10606 | Cytochrome c oxidase subunit 5B, mitochondrial | COX5B | -0.91 | 1.28 | -0.37 | 0.50 | 1.07 | 0.28 | -0.06 | -0.23 | 0.33 | -2.52 | 0.08 | 0.92 |
| Q9NUQ6 | SPATS2-like protein | SPATS2L | -0.91 | 1.56 | 1.13 | -0.11 | -0.80 | 0.92 | 0.47 | 0.99 | 0.46 | -1.72 | 0.01 | -1.36 |
| Q9UBW8 | COP9 signalosome complex subunit 7a | COPS7A | -0.91 | 1.35 | -1.15 | 0.24 | 0.15 | 0.82 | 1.38 | -1.28 | -1.40 | 0.31 | 1.14 | -0.22 |
| P35527 | Keratin, type I cytoskeletal 9 | KRT9 | -0.91 | 3.27 | 0.79 | -1.07 | 1.48 | -0.01 | 1.46 | -0.03 | -1.33 | -0.87 | 0.16 | -0.58 |
| Q5UIP0 | Telomere-associated protein RIF1 | RIF1 | -0.91 | 2.68 | -0.51 | 0.48 | 0.46 | 1.31 | 0.61 | -2.37 | 0.26 | -0.60 | 0.39 | -0.01 |
| Q9UQ26 | Regulating synaptic membrane exocytosis protein 2 | RIMS2 | -0.91 | 1.60 | 2.18 | 0.48 | -1.10 | 0.32 | -0.23 | -0.80 | 0.65 | 0.25 | -0.80 | -0.94 |
| Q93050 | V-type proton ATPase 116 kDa subunit a isoform 1 | ATP6V0A1 | -0.91 | 1.85 | -0.70 | 0.74 | 0.68 | 0.79 | 0.34 | -1.64 | -1.52 | -0.42 | 1.07 | 0.67 |
| Q15048 | Leucine-rich repeat-containing protein 14 | LRRC14 | -0.91 | 1.34 | 1.51 | 0.43 | 0.01 | -0.74 | 0.24 | 1.53 | -0.92 | -1.62 | -0.13 | -0.30 |
| Q9UBL0 | cAMP-regulated phosphoprotein 21 | ARPP21 | -0.91 | 1.77 | 1.72 | 0.79 | -0.51 | 0.03 | -0.26 | -0.80 | -0.86 | -1.30 | -0.21 | 1.41 |
| Q14012 | Calcium/calmodulin-dependent protein kinase type 1 | CAMK1 | -0.91 | 2.36 | -0.10 | -0.44 | 0.61 | -0.04 | 2.09 | -0.43 | -1.96 | 0.07 | 0.20 | 0.00 |
| Q96DM3 | Regulator of MON1-CCZ1 complex | RMC1 | -0.91 | 1.29 | 1.32 | 0.74 | -1.25 | 0.65 | -0.06 | -1.08 | -0.47 | -1.42 | 0.90 | 0.68 |
| Q8IXT5 | RNA-binding protein 12B | RBM12B | -0.91 | 0.75 | 1.54 | -0.95 | -0.85 | 0.50 | 0.66 | 1.37 | -1.04 | -1.04 | 0.26 | -0.47 |
| Q5VZ89 | DENN domain-containing protein 4C | DENND4C | -0.92 | 1.75 | -0.57 | 1.13 | 1.26 | 0.89 | -0.96 | -0.82 | -0.87 | -1.31 | 0.35 | 0.91 |
| O15066 | Kinesin-like protein KIF3B | KIF3B | -0.92 | 1.35 | 0.89 | 0.17 | -0.13 | 0.59 | -0.02 | 0.61 | -0.15 | -2.68 | 0.31 | 0.41 |
| Q9UL51 | Potassium/sodium hyperpolarization-activated cyclic nucleotide-ga | HCN2 | -0.92 | 1.44 | -0.65 | 0.61 | 0.69 | -0.69 | 1.54 | -0.52 | 1.54 | -1.06 | -0.69 | -0.78 |
| Q86V81 | THO complex subunit 4 | ALYREF | -0.92 | 1.90 | 1.15 | 0.69 | 0.57 | 0.75 | -1.32 | -0.70 | 0.53 | -0.46 | -1.80 | 0.59 |
| P35749 | Myosin-11 | MYH11 | -0.92 | 1.48 | -1.39 | 1.09 | 1.94 | 0.04 | -0.11 | -0.82 | -0.38 | -1.02 | 0.17 | 0.47 |
| Q9Y2L9 | Leucine-rich repeat and calponin homology domain-containing prot | LRCH1 | -0.92 | 1.24 | -0.95 | 0.91 | -0.08 | 0.88 | 0.60 | -1.00 | -1.10 | -1.32 | 1.16 | 0.90 |
| Q641Q2 | WASH complex subunit 2A | WASHC2A | -0.92 | 1.94 | 1.10 | 1.07 | -0.21 | 0.02 | -0.08 | 0.46 | 0.20 | -0.26 | -2.49 | 0.19 |
| Q12874 | Splicing factor 3A subunit 3 | SF3A3 | -0.92 | 0.96 | 1.15 | 0.34 | -1.71 | 0.69 | 0.64 | 0.23 | -0.49 | -1.74 | 0.57 | 0.33 |

| | | | | | | | | | | | | | | |
|---|---|---|---|---|---|---|---|---|---|---|---|---|---|---|
| Q7Z6L0 | Proline-rich transmembrane protein 2 | PRRT2 | -0.92 | 1.38 | -0.59 | 1.86 | -0.51 | 0.57 | 0.13 | -1.85 | -0.58 | 0.62 | 0.59 | -0.24 |
| Q9Y2D8 | Afadin- and alpha-actinin-binding protein | SSX2IP | -0.92 | 3.35 | -0.43 | 1.65 | -0.06 | 0.58 | 0.88 | 0.30 | -2.08 | -0.70 | 0.10 | -0.25 |
| Q5SYC1 | Clavesin-2 | CLVS2 | -0.92 | 2.48 | -0.13 | 0.19 | 1.58 | 0.80 | -0.22 | -2.29 | -0.42 | 0.32 | 0.43 | -0.26 |
| Q9Y6N8 | Cadherin-10 | CDH10 | -0.93 | 1.32 | -0.14 | 2.81 | -0.51 | -0.33 | -0.35 | -0.18 | -0.24 | -0.65 | -0.24 | -0.17 |
| Q8NF99 | Zinc finger protein 397 | ZNF397 | -0.93 | 1.49 | -1.42 | 1.46 | -0.32 | 1.13 | 0.71 | -0.30 | -1.54 | -0.47 | 0.48 | 0.27 |
| P49918 | Cyclin-dependent kinase inhibitor 1C | CDKN1C | -0.93 | 2.03 | 2.76 | -0.39 | 0.18 | -0.15 | -0.36 | -0.31 | -0.19 | -0.81 | -0.42 | -0.31 |
| P01111 | GTPase NRas | NRAS | -0.93 | 1.37 | 0.51 | 0.83 | -0.22 | 0.18 | 0.22 | -1.76 | -1.81 | 0.40 | 0.72 | 0.93 |
| P22681 | E3 ubiquitin-protein ligase CBL | CBL | -0.93 | 1.60 | -0.13 | 2.81 | -0.41 | -0.31 | -0.24 | -0.18 | -0.19 | -0.71 | -0.34 | -0.31 |
| Q5SY16 | Polynucleotide 5'-hydroxyl-kinase NOL9 | NOL9 | -0.93 | 1.06 | 1.13 | -0.74 | -0.97 | 1.25 | 0.53 | 0.91 | -0.53 | -1.76 | 0.37 | -0.19 |
| Q66GS9 | Centrosomal protein of 135 kDa | CEP135 | -0.93 | 0.59 | -0.99 | 0.21 | 0.49 | 0.29 | 0.75 | -1.57 | -1.54 | 1.28 | 0.41 | 0.66 |
| Q10713 | Mitochondrial-processing peptidase subunit alpha | PMPCA | -0.93 | 1.76 | 0.63 | -0.69 | 2.05 | 0.55 | -0.78 | -0.64 | -0.80 | -1.24 | 0.39 | 0.53 |
| Q4V328 | GRIP1-associated protein 1 | GRIPAP1 | -0.93 | 1.17 | 0.65 | 0.83 | -0.66 | 0.47 | 0.04 | 0.67 | 0.85 | -2.38 | 0.12 | -0.59 |
| Q9Y2J2 | Band 4.1-like protein 3 | EPB41L3 | -0.93 | 2.82 | 0.57 | 1.26 | -1.16 | 1.15 | 0.56 | -0.21 | -0.22 | -1.68 | 0.63 | -0.89 |
| P21796 | Voltage-dependent anion-selective channel protein 1 | VDAC1 | -0.93 | 3.62 | 0.67 | 0.97 | -0.27 | 0.80 | 0.68 | -1.87 | -1.02 | -0.61 | 1.13 | -0.48 |
| Q16798 | NADP-dependent malic enzyme, mitochondrial | ME3 | -0.93 | 1.21 | 0.79 | -0.31 | 1.20 | -0.52 | 0.19 | 0.92 | -2.04 | 0.77 | -0.04 | -0.96 |
| Q9NR50 | Translation initiation factor eIF-2B subunit gamma | EIF2B3 | -0.93 | 1.12 | 1.00 | 0.20 | 0.44 | 0.46 | -0.82 | 0.85 | 0.58 | -2.34 | 0.20 | -0.57 |
| Q9BWE0 | Replication initiator 1 | REPIN1 | -0.93 | 0.75 | 0.74 | -0.94 | 0.35 | -0.53 | 1.30 | 1.11 | 1.03 | -1.20 | -0.92 | -0.94 |
| Q6IA86 | Elongator complex protein 2 | ELP2 | -0.93 | 1.54 | -0.07 | -0.23 | -0.53 | -0.29 | 2.81 | -0.29 | -0.18 | -0.63 | -0.28 | -0.30 |
| O14974 | Protein phosphatase 1 regulatory subunit 12A | PPP1R12A | -0.94 | 2.30 | -0.74 | 0.55 | 0.45 | -0.14 | 1.99 | -0.99 | -1.10 | -0.83 | -0.18 | 1.03 |
| Q5VSL9 | Striatin-interacting protein 1 | STRIP1 | -0.94 | 0.98 | 0.67 | 0.68 | 0.57 | 0.23 | -1.00 | 0.94 | -0.85 | -2.16 | 0.37 | 0.55 |
| Q9Y4D8 | Probable E3 ubiquitin-protein ligase HECTD4 | HECTD4 | -0.94 | 0.92 | -0.61 | -0.34 | 1.30 | 0.48 | 0.26 | -0.35 | -2.33 | 0.29 | 0.72 | 0.59 |
| Q8IVL0 | Neuron navigator 3 | NAV3 | -0.94 | 1.05 | 1.82 | 0.19 | -0.98 | 0.16 | 0.00 | 1.53 | -0.84 | -1.17 | -0.34 | -0.37 |
| Q9ULX5 | RING finger protein 112 | RNF112 | -0.94 | 1.59 | -0.76 | 1.22 | 0.27 | 1.32 | -0.42 | -0.99 | -1.01 | -1.27 | 0.85 | 0.79 |
| Q13387 | C-Jun-amino-terminal kinase-interacting protein 2 | MAPK8IP2 | -0.94 | 1.89 | -0.51 | 0.75 | 0.88 | -0.33 | 1.07 | -1.43 | -0.63 | 0.69 | -1.49 | 1.01 |
| P0DN79 | Cystathionine beta-synthase-like protein | CBSL | -0.94 | 1.67 | -0.51 | 0.99 | -0.11 | 0.89 | 0.44 | -0.36 | -2.44 | 0.21 | 0.77 | 0.10 |
| Q96Q05 | Trafficking protein particle complex subunit 9 | TRAPPC9 | -0.94 | 1.60 | 0.92 | 0.99 | -0.96 | 0.41 | 0.29 | 0.16 | 0.35 | -2.39 | 0.36 | -0.12 |
| P12429 | Annexin A3 | ANXA3 | -0.94 | 1.79 | -1.26 | 0.82 | 1.07 | 1.00 | 0.14 | 0.18 | -1.43 | 0.83 | -0.01 | -1.34 |
| P62877 | E3 ubiquitin-protein ligase RBX1 | RBX1 | -0.94 | 0.87 | -1.40 | 0.73 | 0.33 | 0.89 | 0.48 | -1.50 | -1.41 | 0.63 | 0.69 | 0.55 |
| Q969K3 | E3 ubiquitin-protein ligase RNF34 | RNF34 | -0.94 | 1.02 | 0.28 | 0.31 | -1.68 | 0.80 | 1.46 | 0.18 | -1.65 | 0.17 | 0.53 | -0.40 |
| Q02040 | A-kinase anchor protein 17A | AKAP17A | -0.94 | 1.10 | 0.65 | -0.50 | -0.67 | -0.53 | 2.31 | -0.45 | 0.97 | -0.84 | -0.46 | -0.47 |
| Q16186 | Proteasomal ubiquitin receptor ADRM1 | ADRM1 | -0.94 | 2.46 | 1.23 | 0.76 | -1.46 | 0.60 | 1.08 | -1.74 | -0.04 | -0.30 | 0.22 | -0.34 |
| Q9Y2W2 | WW domain-binding protein 11 | WBP11 | -0.94 | 2.51 | -0.09 | 0.16 | 1.32 | 0.56 | 0.33 | -1.40 | -1.38 | -1.20 | 0.93 | 0.78 |
| P17980 | 26S proteasome regulatory subunit 6A | PSMC3 | -0.95 | 1.21 | 0.93 | 1.06 | -0.21 | -0.05 | -0.38 | 0.60 | 0.43 | -2.42 | -0.45 | 0.48 |
| Q9BXM0 | Periaxin | PRX | -0.95 | 2.55 | 0.10 | 0.79 | 1.15 | 0.60 | -0.36 | -1.78 | -1.62 | -0.05 | 0.75 | 0.42 |
| A0AVT1 | Ubiquitin-like modifier-activating enzyme 6 | UBA6 | -0.95 | 1.35 | -1.57 | 1.10 | 0.89 | 0.60 | 0.43 | -1.35 | -1.29 | 0.34 | 0.51 | 0.35 |
| O94983 | Calmodulin-binding transcription activator 2 | CAMTA2 | -0.95 | 2.72 | -0.21 | -0.35 | -0.64 | 1.91 | 1.74 | -0.25 | -0.48 | -1.15 | -0.30 | -0.26 |
| Q9Y520 | Protein PRRC2C | PRRC2C | -0.95 | 1.81 | 2.30 | -0.11 | 0.55 | -0.05 | -0.89 | 0.07 | -0.71 | 0.38 | -1.38 | -0.18 |
| O94967 | WD repeat-containing protein 47 | WDR47 | -0.95 | 2.41 | -0.01 | 0.74 | -0.27 | 1.24 | 0.51 | -1.31 | -2.04 | 0.16 | 0.79 | 0.19 |
| P47869 | Gamma-aminobutyric acid receptor subunit alpha-2 | GABRA2 | -0.95 | 1.84 | 0.53 | 0.79 | 0.33 | -0.21 | 0.45 | 0.61 | 0.33 | -2.69 | 0.00 | -0.13 |
| Q99758 | ATP-binding cassette sub-family A member 3 | ABCA3 | -0.95 | 0.57 | -0.98 | 0.24 | 0.03 | 0.80 | 0.65 | -1.61 | -1.53 | 0.68 | 0.83 | 0.91 |
| Q86XP1 | Diacylglycerol kinase eta | DGKH | -0.95 | 2.03 | 1.18 | -0.49 | -0.91 | 1.18 | 0.96 | -0.44 | -1.97 | 0.41 | 0.04 | 0.04 |
| Q7L804 | Rab11 family-interacting protein 2 | RAB11FIP2 | -0.95 | 1.34 | 1.27 | 0.60 | -0.87 | 0.85 | -0.41 | 0.69 | 0.36 | -2.04 | 0.28 | -0.72 |
| Q8IYT4 | Katanin p60 ATPase-containing subunit A-like 2 | KATNAL2 | -0.95 | 1.06 | 0.14 | 0.27 | 0.22 | 0.28 | 0.35 | -0.38 | -2.65 | 1.11 | 0.42 | 0.24 |
| Q92615 | La-related protein 4B | LARP4B | -0.95 | 0.78 | -0.35 | -0.18 | -0.15 | 0.02 | 1.61 | -1.69 | -0.22 | -0.60 | -0.15 | 1.72 |
| Q13643 | Four and a half LIM domains protein 3 | FHL3 | -0.95 | 2.05 | 0.55 | 0.05 | 0.27 | 0.73 | 0.46 | 0.47 | 0.18 | -2.67 | 0.49 | -0.53 |
| Q16143 | Beta-synuclein | SNCB | -0.96 | 1.20 | -0.85 | 0.53 | -0.48 | 0.19 | 1.93 | -1.52 | -0.96 | 0.86 | 0.03 | 0.25 |
| Q7Z6J0 | E3 ubiquitin-protein ligase SH3RF1 | SH3RF1 | -0.96 | 1.48 | 0.56 | 1.78 | -0.98 | 1.07 | -0.87 | -0.70 | 0.56 | -1.07 | 0.38 | -0.74 |
| Q8NFG4 | Folliculin | FLCN | -0.96 | 0.99 | 1.18 | -0.52 | -0.76 | 1.87 | -0.62 | -0.56 | 1.20 | -0.75 | -0.49 | -0.55 |
| Q9NUL3 | Double-stranded RNA-binding protein Staufen homolog 2 | STAU2 | -0.96 | 0.75 | 0.88 | 1.81 | -1.06 | -0.49 | -0.22 | 0.73 | 0.73 | -0.19 | -0.91 | -1.27 |
| Q8N1I0 | Dedicator of cytokinesis protein 4 | DOCK4 | -0.96 | 1.39 | 0.38 | 1.56 | -0.68 | 0.17 | 0.05 | -2.07 | 0.84 | -0.74 | 0.60 | -0.12 |
| Q9H3K6 | BolA-like protein 2 | BOLA2 | -0.96 | 2.49 | -0.67 | 1.41 | 0.35 | 0.40 | 0.73 | -1.43 | -1.48 | -0.70 | 0.53 | 0.86 |
| Q86YS7 | C2 domain-containing protein 5 | C2CD5 | -0.96 | 1.54 | 0.81 | 0.08 | 0.09 | 0.46 | 0.23 | 0.49 | 0.51 | -2.74 | 0.25 | -0.17 |
| Q9P107 | GEM-interacting protein | GMIP | -0.96 | 0.70 | 0.49 | 0.34 | -0.97 | -0.79 | 1.79 | -0.36 | -0.41 | 1.51 | -0.85 | -0.76 |
| Q9BW30 | Tubulin polymerization-promoting protein family member 3 | TPPP3 | -0.96 | 0.87 | -1.22 | -0.18 | 0.55 | 0.45 | 1.42 | -1.33 | -1.28 | 1.19 | 0.33 | 0.06 |
| P22105 | Tenascin-X | TNXB | -0.96 | 1.29 | 0.92 | -0.08 | 1.83 | -0.19 | -1.07 | 0.53 | -1.02 | -1.37 | -0.16 | 0.63 |
| Q9NUQ8 | ATP-binding cassette sub-family F member 3 | ABCF3 | -0.96 | 1.42 | 0.51 | -0.29 | 1.03 | 0.30 | -0.02 | -0.34 | -2.56 | 0.85 | 0.29 | 0.21 |
| Q99755 | Phosphatidylinositol 4-phosphate 5-kinase type-1 alpha | PIP5K1A | -0.96 | 2.16 | 0.42 | 0.96 | -1.06 | 1.17 | 0.52 | -1.53 | -0.25 | -1.29 | 1.05 | 0.01 |
| P14625 | Endoplasmin | HSP90B1 | -0.96 | 1.55 | -0.79 | -0.37 | 0.57 | -0.27 | 2.50 | 0.15 | -1.20 | -0.14 | -0.30 | -0.16 |
| P34947 | G protein-coupled receptor kinase 5 | GRK5 | -0.96 | 1.53 | 0.60 | 1.49 | -1.48 | 1.22 | -0.23 | -1.59 | -0.18 | -0.13 | 0.29 | 0.01 |
| Q9BQ04 | RNA-binding protein 4B | RBM4B | -0.96 | 1.31 | 1.05 | 0.12 | -1.60 | 1.02 | 0.84 | -0.02 | 0.18 | -1.75 | 0.57 | -0.40 |
| Q6XUX3 | Dual serine/threonine and tyrosine protein kinase | DSTYK | -0.97 | 0.63 | -1.24 | 0.56 | 0.47 | 0.13 | 0.89 | -1.17 | -1.64 | 1.37 | 0.29 | 0.35 |
| P60880 | Synaptosomal-associated protein 25 | SNAP25 | -0.97 | 3.06 | 0.13 | 2.13 | 0.14 | 0.50 | -0.41 | -1.07 | 0.38 | -1.55 | 0.25 | -0.51 |
| Q96T37 | RNA-binding protein 15 | RBM15 | -0.97 | 1.12 | 1.84 | -0.87 | -1.08 | 0.92 | 0.46 | 0.21 | -0.82 | -1.23 | 0.61 | -0.04 |
| Q16762 | Thiosulfate sulfurtransferase | TST | -0.97 | 1.12 | -0.76 | 0.54 | 1.35 | 0.05 | 0.09 | -1.51 | -1.56 | 1.13 | 0.55 | 0.11 |
| Q8NEY1 | Neuron navigator 1 | NAV1 | -0.97 | 1.17 | -1.29 | 0.77 | 0.44 | 0.83 | 0.56 | -0.61 | -2.01 | -0.16 | 0.79 | 0.69 |
| Q9Y6X8 | Zinc fingers and homeoboxes protein 2 | ZHX2 | -0.97 | 1.51 | 1.30 | 0.30 | -0.87 | -0.79 | 1.63 | -0.73 | -0.76 | -1.12 | 0.89 | 0.15 |
| Q8N336 | ELMO domain-containing protein 1 | ELMOD1 | -0.97 | 1.63 | -0.47 | 0.88 | -0.34 | 0.66 | 0.95 | -1.18 | -2.06 | 0.36 | 0.75 | 0.45 |
| Q15111 | Inactive phospholipase C-like protein 1 | PLCL1 | -0.97 | 2.16 | -0.78 | 1.91 | 0.77 | 0.75 | -0.63 | -1.28 | -0.34 | -0.91 | 0.75 | -0.23 |
| Q2UY09 | Collagen alpha-1(XXVIII) chain | COL28A1 | -0.98 | 0.92 | -0.28 | -0.40 | 2.57 | -0.36 | -0.44 | 0.89 | -0.43 | -0.72 | -0.44 | -0.38 |
| Q92547 | DNA topoisomerase 2-binding protein 1 | TOPBP1 | -0.98 | 1.45 | -0.36 | -0.63 | -0.70 | 1.13 | 2.10 | -0.43 | -0.54 | -0.91 | 0.85 | -0.51 |
| Q6DN90 | IQ motif and SEC7 domain-containing protein 1 | IQSEC1 | -0.98 | 2.49 | 0.93 | 1.28 | -0.92 | 0.74 | 0.17 | -1.04 | 0.67 | -1.83 | 0.34 | -0.34 |
| Q6P995 | Protein FAM171B | FAM171B | -0.98 | 2.06 | -0.68 | 1.29 | -0.46 | 1.02 | 0.78 | -1.35 | -0.91 | -1.07 | 1.05 | 0.34 |
| Q15555 | Microtubule-associated protein RP/EB family member 2 | MAPRE2 | -0.98 | 3.78 | 0.62 | 1.08 | -0.66 | 1.05 | 0.74 | -1.11 | -0.01 | -1.91 | 0.59 | -0.38 |
| P56693 | Transcription factor SOX-10 | SOX10 | -0.98 | 1.25 | 0.76 | -0.20 | -1.55 | 0.73 | 1.63 | 0.13 | -1.34 | -0.52 | 0.75 | -0.40 |
| P50993 | Sodium/potassium-transporting ATPase subunit alpha-2 | ATP1A2 | -0.98 | 1.21 | 0.44 | 0.89 | 0.41 | 0.00 | -0.37 | 0.41 | -2.69 | 0.27 | 0.16 | 0.47 |
| Q5T2T1 | MAGUK p55 subfamily member 7 | MPP7 | -0.98 | 1.52 | -0.77 | 1.13 | -1.09 | 1.61 | 0.71 | -0.91 | 0.29 | -1.33 | -0.07 | 0.42 |
| Q92558 | Wiskott-Aldrich syndrome protein family member 1 | WASF1 | -0.98 | 1.52 | -0.70 | 1.00 | 0.35 | 0.27 | 0.68 | -2.55 | 0.50 | 0.32 | 0.12 | 0.00 |
| Q9P2R3 | Rabankyrin-5 | ANKFY1 | -0.98 | 1.57 | -0.32 | -0.32 | -0.56 | 0.54 | 2.28 | 0.28 | -0.41 | -1.64 | -0.03 | 0.18 |
| Q9C0D0 | Phosphatase and actin regulator 1 | PHACTR1 | -0.98 | 2.18 | 0.56 | 1.67 | -1.26 | 0.97 | 0.10 | 0.08 | -0.31 | -0.74 | 0.51 | -1.56 |
| Q8N8S7 | Protein enabled homolog | ENAH | -0.99 | 1.33 | -1.30 | -0.08 | 1.59 | 0.55 | 0.67 | -0.97 | -1.48 | 0.86 | 0.33 | -0.17 |
| Q9P0K1 | Disintegrin and metalloproteinase domain-containing protein 22 | ADAM22 | -0.99 | 2.59 | -0.32 | 2.02 | -0.88 | 0.86 | 0.61 | -0.33 | -0.78 | -1.10 | 0.67 | -0.75 |
| O43681 | ATPase ASNA1 | ASNA1 | -0.99 | 1.36 | -0.36 | 0.36 | -0.72 | -0.32 | 2.53 | -1.13 | -0.22 | -0.37 | -0.24 | 0.47 |
| P46952 | 3-hydroxyanthranilate 3,4-dioxygenase | HAAO | -0.99 | 2.19 | -0.40 | 1.03 | 0.43 | 0.78 | 0.24 | -0.32 | -2.16 | -1.05 | 0.84 | 0.60 |

| | | | | | | | | | | | | | | |
|---|---|---|---|---|---|---|---|---|---|---|---|---|---|---|
| Q16531 | DNA damage-binding protein 1 | DDB1 | -0.99 | 0.76 | -0.63 | -0.07 | 0.69 | 0.49 | 0.45 | -1.09 | -2.14 | 1.09 | 0.71 | 0.48 |
| Q6P1M0 | Long-chain fatty acid transport protein 4 | SLC27A4 | -0.99 | 1.74 | 0.54 | 1.35 | -0.29 | 0.18 | -0.01 | 0.64 | -2.35 | -0.69 | 0.02 | 0.60 |
| Q92561 | Phytanoyl-CoA hydroxylase-interacting protein | PHYHIP | -0.99 | 3.35 | 1.10 | 0.99 | 0.07 | 0.63 | -0.07 | -0.82 | 0.61 | -2.31 | 0.01 | -0.21 |
| Q14137 | Ribosome biogenesis protein BOP1 | BOP1 | -0.99 | 1.65 | 0.80 | -1.04 | 0.57 | -0.03 | 1.37 | 1.19 | -1.04 | -1.51 | 0.19 | -0.52 |
| Q6UX04 | Spliceosome-associated protein CWC27 homolog | CWC27 | -0.99 | 2.25 | 2.21 | -1.03 | 0.14 | 0.38 | 0.39 | -1.08 | -0.22 | -1.19 | 0.14 | 0.26 |
| Q9BZK3 | Putative nascent polypeptide-associated complex subunit alpha-li | NACA4P | -0.99 | 0.55 | -1.40 | 0.74 | 0.36 | 0.56 | 0.44 | -1.44 | -1.44 | 0.50 | 0.72 | 0.96 |
| O15144 | Actin-related protein 2/3 complex subunit 2 | ARPC2 | -1.00 | 0.69 | -1.46 | 0.24 | 0.40 | 0.51 | 1.15 | -1.46 | -1.29 | 0.83 | 0.63 | 0.44 |
| P02538 | Keratin, type II cytoskeletal 6A | KRT6A | -1.00 | 1.73 | 1.36 | -1.04 | 0.18 | 0.40 | 0.83 | -0.79 | -1.62 | 1.09 | 0.37 | -0.79 |
| Q9BWQ8 | Protein lifeguard 2 | FAIM2 | -1.00 | 1.11 | 1.10 | 1.56 | -0.96 | 0.40 | -0.86 | 0.80 | -0.78 | -1.16 | 0.63 | -0.75 |
| P19086 | Guanine nucleotide-binding protein G(z) subunit alpha | GNAZ | -1.00 | 2.70 | 0.40 | 1.60 | -0.12 | 0.46 | 0.00 | -0.85 | 0.36 | -2.15 | 0.67 | -0.37 |
| Q9ULV0 | Unconventional myosin-Vb | MYO5B | -1.00 | 0.92 | -0.30 | 0.22 | -0.12 | 0.50 | 0.81 | -0.63 | 1.38 | -2.32 | 0.53 | -0.07 |
| Q9Y6K9 | NF-kappa-B essential modulator | IKBKG | -1.00 | 0.88 | -0.92 | 0.22 | 1.66 | 0.47 | -0.39 | -1.72 | -0.88 | 0.91 | 0.67 | -0.03 |
| Q06546 | GA-binding protein alpha chain | GABPA | -1.00 | 1.38 | -0.08 | 0.84 | 0.69 | 0.10 | -0.03 | -0.41 | -2.59 | 0.22 | 0.41 | 0.85 |
| Q684P5 | Rap1 GTPase-activating protein 2 | RAP1GAP2 | -1.00 | 2.45 | -0.14 | 1.13 | -0.53 | 0.98 | 0.77 | -1.28 | -0.24 | -1.83 | 0.89 | 0.25 |
| Q8NBN7 | Retinol dehydrogenase 13 | RDH13 | -1.00 | 0.79 | -1.33 | 0.59 | 0.25 | 0.82 | 0.62 | -1.45 | -1.48 | 0.28 | 0.85 | 0.83 |
| Q15735 | Phosphatidylinositol 4,5-bisphosphate 5-phosphatase A | INPP5J | -1.00 | 1.21 | -0.39 | 1.29 | -0.75 | -0.59 | 1.78 | -0.48 | 1.18 | -0.92 | -0.58 | -0.53 |
| A5YKK6 | CCR4-NOT transcription complex subunit 1 | CNOT1 | -1.00 | 1.41 | 0.88 | 0.58 | 0.04 | 0.28 | -0.23 | -1.03 | -2.31 | 0.27 | 0.48 | 1.04 |
| O76039 | Cyclin-dependent kinase-like 5 | CDKL5 | -1.00 | 1.12 | -0.03 | 0.21 | 0.19 | 0.82 | 0.11 | -0.58 | -2.59 | 0.70 | 0.53 | 0.64 |
| Q96HU8 | GTP-binding protein Di-Ras2 | DIRAS2 | -1.01 | 3.21 | 0.99 | 1.16 | -0.82 | 0.60 | 0.63 | -0.65 | 0.19 | -2.07 | 0.46 | -0.50 |
| Q9UF33 | Ephrin type-A receptor 6 | EPHA6 | -1.01 | 1.05 | -0.45 | 1.52 | -0.72 | 1.43 | -0.58 | -0.61 | -0.53 | -0.89 | 1.36 | -0.52 |
| Q9H6K5 | Proline-rich protein 36 | PRR36 | -1.01 | 0.93 | -0.28 | 1.02 | 0.39 | 0.73 | -0.77 | -2.28 | -0.35 | 0.51 | 0.04 | 1.00 |
| Q9NQX4 | Unconventional myosin-Vc | MYO5C | -1.01 | 1.03 | -1.21 | 0.41 | -0.03 | 1.54 | 0.47 | -0.09 | -1.33 | 0.46 | 1.07 | -1.28 |
| Q9C0E2 | Exportin-4 | XPO4 | -1.01 | 0.82 | -0.54 | -0.69 | 2.11 | 0.82 | -0.70 | 0.62 | -0.70 | -0.88 | -0.70 | 0.67 |
| Q7L5N1 | COP9 signalosome complex subunit 6 | COPS6 | -1.01 | 0.59 | -1.71 | 0.86 | 0.37 | 0.63 | 0.59 | -0.49 | -1.80 | 0.17 | 0.75 | 0.63 |
| Q05823 | 2-5A-dependent ribonuclease | RNASEL | -1.01 | 2.61 | -0.20 | 1.58 | -0.58 | 2.09 | -0.49 | -0.16 | -0.45 | -0.99 | -0.45 | -0.36 |
| P62995 | Transformer-2 protein homolog beta | TRA2B | -1.01 | 1.43 | -1.09 | 0.72 | 0.42 | 0.93 | 0.53 | -1.66 | -1.45 | 0.58 | 0.87 | 0.14 |
| O15400 | Syntaxin-7 | STX7 | -1.01 | 2.46 | -0.30 | 1.83 | 0.40 | -0.64 | 0.90 | -0.79 | -1.24 | -0.01 | 0.94 | -1.08 |
| Q8WVR3 | Microtubule-associated protein 11 | MAP11 | -1.01 | 3.90 | 1.00 | 1.31 | -1.12 | 1.27 | 0.44 | 0.43 | -0.79 | -1.19 | -0.87 | -0.48 |
| P63010 | AP-2 complex subunit beta | AP2B1 | -1.01 | 1.88 | -1.19 | 1.30 | 0.48 | 0.58 | 0.66 | -1.92 | -0.85 | -0.02 | 0.59 | 0.37 |
| Q9NZ09 | Ubiquitin-associated protein 1 | UBAP1 | -1.01 | 1.57 | -0.03 | 2.81 | -0.47 | -0.27 | -0.35 | -0.26 | -0.19 | -0.58 | -0.36 | -0.31 |
| Q5K651 | Sterile alpha motif domain-containing protein 9 | SAMD9 | -1.01 | 1.68 | -0.57 | 0.82 | -0.89 | 0.87 | 1.46 | -0.64 | -0.69 | -1.03 | 1.37 | -0.70 |
| Q99501 | GAS2-like protein 1 | GAS2L1 | -1.02 | 2.27 | 0.23 | 0.06 | 0.62 | 0.41 | 0.87 | -0.42 | 0.54 | -0.91 | 0.94 | -2.34 |
| Q69YU3 | Ankyrin repeat domain-containing protein 34A | ANKRD34A | -1.02 | 1.44 | 0.22 | 0.43 | 0.40 | 1.03 | -0.53 | -1.60 | -1.45 | 0.96 | 1.15 | -0.60 |
| O60229 | Kalirin | KALRN | -1.02 | 1.40 | 0.61 | 1.06 | -0.88 | 0.55 | 0.16 | -0.05 | 0.68 | -2.38 | 0.49 | -0.24 |
| Q96T60 | Bifunctional polynucleotide phosphatase/kinase | PNKP | -1.02 | 1.18 | -0.11 | 0.54 | 1.16 | -0.28 | 0.02 | -0.99 | -2.14 | 1.07 | 0.76 | -0.04 |
| Q6P996 | Pyridoxal-dependent decarboxylase domain-containing protein 1 | PDXDC1 | -1.02 | 2.29 | -0.67 | 0.71 | 2.48 | 0.01 | -0.36 | -0.55 | -0.51 | -1.10 | -0.01 | 0.00 |
| Q7Z7M0 | Multiple epidermal growth factor-like domains protein 8 | MEGF8 | -1.02 | 2.04 | 1.28 | 1.45 | -0.45 | 0.44 | -0.80 | 0.40 | -1.50 | 0.41 | 0.05 | -1.28 |
| Q9Y5B0 | RNA polymerase II subunit A C-terminal domain phosphatase | CTDP1 | -1.02 | 1.18 | 0.89 | 0.36 | -1.29 | 0.77 | 0.58 | 1.23 | -1.24 | -1.41 | 0.52 | -0.41 |
| Q86YZ3 | Hornerin | HRNR | -1.02 | 3.45 | 0.42 | 1.94 | 0.02 | 0.47 | -0.19 | 0.19 | 0.29 | -1.97 | -0.36 | -0.81 |
| O94812 | BAI1-associated protein 3 | BAIAP3 | -1.02 | 1.27 | 0.58 | 0.73 | -0.08 | 1.33 | -1.19 | 0.39 | -1.14 | -1.42 | 1.15 | -0.37 |
| Q92835 | Phosphatidylinositol 3,4,5-trisphosphate 5-phosphatase 1 | INPP5D | -1.02 | 2.12 | -0.81 | 1.92 | 0.16 | 0.83 | -0.12 | -1.21 | -0.21 | -1.42 | 0.56 | 0.29 |
| Q9UJY4 | ADP-ribosylation factor-binding protein GGA2 | GGA2 | -1.02 | 1.50 | 1.56 | -0.83 | -0.96 | 0.81 | 0.98 | -0.77 | -0.81 | -1.07 | 0.96 | 0.13 |
| Q96P50 | Arf-GAP with coiled-coil, ANK repeat and PH domain-containing pı | ACAP3 | -1.02 | 3.01 | 0.53 | 0.25 | -1.19 | 1.73 | 1.19 | -0.69 | -0.86 | -1.23 | 0.30 | -0.04 |
| Q8NE35 | Cytoplasmic polyadenylation element-binding protein 3 | CPEB3 | -1.02 | 1.72 | 0.64 | -0.50 | -0.76 | 1.02 | 1.31 | -1.21 | -0.62 | -1.37 | 0.47 | 1.01 |
| Q86TG7 | Retrotransposon-derived protein PEG10 | PEG10 | -1.02 | 1.63 | 1.85 | -0.69 | -0.95 | 0.37 | 1.07 | 0.96 | -0.27 | -0.95 | -0.76 | -0.65 |
| Q86SX6 | Glutaredoxin-related protein 5, mitochondrial | GLRX5 | -1.02 | 1.80 | -0.04 | -0.32 | 2.81 | -0.24 | -0.33 | -0.34 | -0.31 | -0.63 | -0.42 | -0.18 |
| Q9Y5Q8 | General transcription factor 3C polypeptide 5 | GTF3C5 | -1.02 | 1.79 | 1.20 | 0.36 | 1.43 | -0.15 | -1.08 | -0.16 | -1.00 | -1.32 | 1.13 | -0.42 |
| O76081 | Regulator of G-protein signaling 20 | RGS20 | -1.03 | 1.11 | -0.81 | 0.92 | -1.13 | 1.42 | 0.85 | 0.15 | -0.21 | -1.27 | 0.99 | -0.92 |
| P15056 | Serine/threonine-protein kinase B-raf | BRAF | -1.03 | 2.21 | 1.32 | 0.40 | -0.80 | 0.82 | 0.31 | -0.32 | -0.81 | -2.06 | 0.46 | 0.68 |
| P10316 | HLA class I histocompatibility antigen, A-69 alpha chain | HLA-A | -1.03 | 2.70 | -0.88 | 1.73 | 0.44 | 0.80 | 0.22 | -1.02 | -1.43 | -0.87 | 0.62 | 0.38 |
| Q9UKJ3 | G patch domain-containing protein 8 | GPATCH8 | -1.03 | 1.23 | -1.03 | 0.58 | 0.64 | 1.00 | 0.16 | -1.55 | -1.58 | 0.29 | 0.92 | 0.57 |
| Q9Y2Q0 | Phospholipid-transporting ATPase IA | ATP8A1 | -1.03 | 1.64 | -0.10 | 1.42 | 0.09 | 0.28 | 0.01 | -1.19 | 1.47 | -1.82 | -0.06 | -0.10 |
| Q13177 | Serine/threonine-protein kinase PAK 2 | PAK2 | -1.03 | 1.30 | 0.59 | 0.02 | 1.18 | -0.08 | -0.27 | 1.12 | -2.40 | 0.23 | -0.34 | -0.05 |
| P05129 | Protein kinase C gamma type | PRKCG | -1.03 | 1.58 | -0.41 | -0.28 | 2.12 | 0.19 | 0.00 | -1.71 | -0.62 | 0.87 | 0.17 | -0.33 |
| Q96EK4 | THAP domain-containing protein 11 | THAP11 | -1.03 | 2.38 | -0.09 | -0.23 | -0.61 | 0.60 | 2.56 | -0.10 | -0.16 | -0.79 | -0.21 | -0.97 |
| Q96JE9 | Microtubule-associated protein 6 | MAP6 | -1.03 | 3.12 | 1.39 | 1.49 | 0.54 | -0.24 | -0.66 | -1.27 | 0.76 | -1.22 | -0.58 | -0.21 |
| O94966 | Ubiquitin carboxyl-terminal hydrolase 19 | USP19 | -1.03 | 2.88 | -0.18 | 1.76 | -0.05 | -0.24 | 1.10 | -0.21 | 0.32 | -2.04 | 0.07 | -0.53 |
| Q9Y4F5 | Centrosomal protein of 170 kDa protein B | CEP170B | -1.03 | 2.98 | 0.63 | 0.94 | 1.07 | 0.43 | -0.56 | -1.34 | -0.38 | 0.96 | 0.05 | -1.79 |
| Q9Y217 | Myotubularin-related protein 6 | MTMR6 | -1.04 | 1.22 | 0.83 | 0.59 | -0.78 | 0.73 | -0.01 | 0.19 | 0.29 | -2.54 | 0.46 | 0.23 |
| Q15056 | Eukaryotic translation initiation factor 4H | EIF4H | -1.04 | 2.31 | 0.30 | 2.02 | -0.27 | 0.66 | -0.63 | -0.76 | -0.08 | -1.77 | 0.41 | 0.11 |
| P20264 | POU domain, class 3, transcription factor 3 | POU3F3 | -1.04 | 1.40 | 0.03 | -0.62 | -0.74 | 1.29 | 1.53 | -0.66 | -0.56 | -1.00 | 1.38 | -0.64 |
| P13611 | Versican core protein | VCAN | -1.04 | 1.23 | -0.39 | -0.76 | 1.63 | 0.13 | 0.73 | -0.66 | -1.71 | 1.16 | 0.35 | -0.48 |
| Q8WUH6 | Transmembrane protein 263 | TMEM263 | -1.04 | 1.16 | -0.70 | -0.61 | -0.36 | 0.37 | 2.65 | -0.68 | -0.39 | -0.36 | 0.27 | -0.19 |
| Q9NP77 | RNA polymerase II subunit A C-terminal domain phosphatase SSl | SSU72 | -1.04 | 2.31 | 0.70 | 0.09 | -0.88 | 1.33 | 0.85 | 0.03 | 0.46 | -2.13 | -0.65 | 0.19 |
| Q9H000 | Probable E3 ubiquitin-protein ligase makorin-2 | MKRN2 | -1.04 | 1.54 | 0.77 | -0.62 | -1.05 | 0.97 | 1.52 | -0.28 | -0.97 | -1.35 | 0.91 | 0.09 |
| Q9Y6M7 | Sodium bicarbonate cotransporter 3 | SLC4A7 | -1.04 | 2.35 | -0.92 | 1.03 | 0.15 | -0.29 | 2.22 | -0.99 | -1.01 | 0.06 | -0.22 | -0.03 |
| Q96RT7 | Gamma-tubulin complex component 6 | TUBGCP6 | -1.04 | 0.75 | 1.02 | -0.96 | 1.28 | -0.93 | 0.50 | 1.10 | -0.91 | 0.73 | -0.96 | -0.88 |
| Q6FI81 | Anamorsin | CIAPIN1 | -1.05 | 2.51 | -0.70 | -0.18 | 0.85 | 0.29 | 1.94 | -0.92 | -1.65 | 0.29 | -0.30 | 0.37 |
| P31321 | cAMP-dependent protein kinase type I-beta regulatory subunit | PRKAR1B | -1.05 | 1.59 | 0.38 | 1.77 | -1.03 | 0.53 | -0.03 | 0.05 | 0.06 | -2.08 | 0.20 | 0.14 |
| Q99259 | Glutamate decarboxylase 1 | GAD1 | -1.05 | 1.38 | 0.54 | 0.61 | 0.46 | -0.26 | 0.19 | -0.87 | 0.58 | -2.44 | 0.24 | 0.97 |
| Q9BZC1 | CUGBP Elav-like family member 4 | CELF4 | -1.05 | 0.66 | 1.02 | -1.14 | -1.22 | 0.99 | 1.18 | -0.52 | 0.20 | -1.35 | 0.81 | 0.04 |
| Q8IYB3 | Serine/arginine repetitive matrix protein 1 | SRRM1 | -1.05 | 2.13 | -1.18 | 0.72 | 0.45 | 0.62 | 1.37 | -1.87 | -0.95 | 0.33 | 0.28 | 0.23 |
| P11678 | Eosinophil peroxidase | EPX | -1.05 | 3.06 | 1.45 | 1.27 | -0.83 | 1.35 | -0.66 | -0.63 | -0.57 | -1.16 | 0.22 | -0.45 |
| Q969S2 | Endonuclease 8-like 2 | NEIL2 | -1.05 | 2.33 | 2.16 | -0.53 | -0.70 | -0.18 | 1.49 | -0.47 | -0.33 | -0.85 | -0.06 | -0.52 |
| O60307 | Microtubule-associated serine/threonine-protein kinase 3 | MAST3 | -1.05 | 1.59 | -0.41 | 0.24 | 0.62 | 0.89 | 0.34 | -2.08 | -1.32 | 0.81 | 0.86 | 0.06 |
| Q6UUV7 | CREB-regulated transcription coactivator 3 | CRTC3 | -1.05 | 2.89 | 1.84 | -0.40 | 1.88 | -0.23 | -0.51 | -0.30 | -0.28 | -0.96 | -0.53 | -0.51 |
| P36542 | ATP synthase subunit gamma, mitochondrial | ATP5F1C | -1.05 | 2.04 | -0.19 | 0.42 | 1.64 | -0.18 | 0.27 | -0.32 | -2.13 | -0.76 | 0.58 | 0.68 |
| P10515 | Dihydrolipoyllysine-residue acetyltransferase component of pyruv | DLAT | -1.05 | 2.58 | 1.22 | 0.21 | 1.09 | 0.28 | -0.53 | -0.44 | 0.88 | -2.17 | -0.07 | -0.48 |
| P11498 | Pyruvate carboxylase, mitochondrial | PC | -1.05 | 1.79 | -0.58 | -0.49 | 2.33 | 0.68 | -0.15 | -0.86 | 0.02 | -1.21 | 0.50 | -0.24 |
| A6NHQ2 | rRNA/tRNA 2'-O-methyltransferase fibrillarin-like protein 1 | FBLL1 | -1.06 | 1.35 | -0.51 | -0.69 | -0.58 | 1.17 | 2.07 | -0.63 | -0.70 | -0.42 | 0.91 | -0.63 |
| Q2NKQ1 | Small G protein signaling modulator 1 | SGSM1 | -1.06 | 1.18 | 0.54 | 1.27 | -1.14 | 0.57 | 0.07 | -0.53 | 1.14 | -1.95 | 0.22 | -0.19 |
| Q6VVB1 | E3 ubiquitin-protein ligase NHLRC1 | NHLRC1 | -1.06 | 1.63 | 1.10 | -0.77 | -1.02 | 0.67 | 1.69 | 0.58 | -0.58 | -1.22 | 0.31 | -0.77 |
| Q8TEU7 | Rap guanine nucleotide exchange factor 6 | RAPGEF6 | -1.06 | 1.68 | -0.09 | -0.34 | -0.42 | -0.18 | 2.81 | -0.29 | -0.18 | -0.69 | -0.29 | -0.33 |

| | | | | | | | | | | | | | | |
|---|---|---|---|---|---|---|---|---|---|---|---|---|---|---|
| Q86UE4 | Protein LYRIC | MTDH | -1.06 | 1.02 | -1.43 | 0.60 | 0.27 | 1.18 | 0.55 | -1.61 | -1.01 | -0.12 | 0.87 | 0.69 |
| O95198 | Kelch-like protein 2 | KLHL2 | -1.06 | 2.63 | -0.08 | 2.01 | -0.66 | 1.68 | -0.51 | -0.49 | -0.36 | -0.91 | -0.40 | -0.28 |
| Q96DA2 | Ras-related protein Rab-39B | RAB39B | -1.06 | 3.12 | 0.67 | 1.27 | -0.97 | 0.52 | 1.02 | -0.86 | -0.34 | -1.88 | 0.55 | 0.02 |
| Q96JG8 | Melanoma-associated antigen D4 | MAGED4 | -1.06 | 1.15 | 0.41 | 0.08 | -0.59 | 0.32 | 1.10 | -1.65 | -1.73 | 0.79 | 0.51 | 0.77 |
| P55196 | Afadin | AFDN | -1.06 | 2.17 | -0.05 | 0.44 | 1.92 | -0.88 | 0.58 | -0.83 | -1.11 | 0.82 | 0.25 | -1.15 |
| Q9UQM7 | Calcium/calmodulin-dependent protein kinase type II subunit alph | CAMK2A | -1.06 | 3.25 | 0.99 | -0.20 | -0.61 | 0.91 | 1.48 | -1.06 | 0.23 | -1.72 | 0.46 | -0.48 |
| Q96JN2 | Coiled-coil domain-containing protein 136 | CCDC136 | -1.06 | 1.50 | 0.19 | 0.66 | 0.47 | 0.34 | -0.02 | 0.31 | -2.77 | 0.54 | 0.36 | -0.07 |
| Q96G97 | Seipin | BSCL2 | -1.06 | 5.39 | 1.05 | 0.08 | 1.14 | 0.79 | 0.41 | 0.43 | -0.48 | -1.32 | -0.21 | -1.89 |
| Q9NQX5 | Neural proliferation differentiation and control protein 1 | NPDC1 | -1.06 | 1.16 | -0.75 | 1.55 | 1.10 | 0.32 | -0.91 | -0.80 | 0.89 | -1.12 | 0.64 | -0.90 |
| O95299 | NADH dehydrogenase [ubiquinone] 1 alpha subcomplex subunit 10 | NDUFA10 | -1.07 | 2.05 | 1.22 | -0.48 | 1.87 | -0.57 | -0.09 | -0.85 | 0.50 | -1.36 | -0.66 | 0.43 |
| Q8IXK0 | Polyhomeotic-like protein 2 | PHC2 | -1.07 | 1.49 | 0.33 | -0.15 | 0.64 | -0.11 | 0.91 | -1.02 | 0.93 | -2.08 | 1.08 | -0.51 |
| P42658 | Dipeptidyl aminopeptidase-like protein 6 | DPP6 | -1.07 | 1.33 | 0.44 | 0.53 | -0.52 | 0.23 | 0.79 | -1.95 | 1.59 | 0.14 | -0.16 | -1.09 |
| Q96EP0 | E3 ubiquitin-protein ligase RNF31 | RNF31 | -1.07 | 1.18 | 0.81 | 0.52 | -1.53 | 0.85 | 0.66 | 0.23 | -1.07 | -1.62 | 0.75 | 0.41 |
| Q8IYM9 | E3 ubiquitin-protein ligase TRIM22 | TRIM22 | -1.07 | 2.02 | -0.42 | 0.51 | -0.91 | 1.33 | 1.41 | -0.61 | -0.61 | -1.12 | 1.18 | -0.76 |
| P08240 | Signal recognition particle receptor subunit alpha | SRPRA | -1.07 | 1.29 | -1.12 | 0.94 | 0.09 | 0.89 | 0.60 | 0.03 | 0.34 | -2.32 | 0.44 | 0.10 |
| Q9UBP0 | Spastin | SPAST | -1.07 | 0.58 | -1.29 | 0.01 | 0.51 | 0.13 | 1.38 | -1.28 | -1.29 | 1.30 | 0.35 | 0.19 |
| P22087 | rRNA 2'-O-methyltransferase fibrillarin | FBL | -1.07 | 1.47 | 0.42 | 0.30 | -1.15 | 0.90 | 1.07 | -0.53 | -2.09 | 0.40 | 0.80 | -0.13 |
| O75347 | Tubulin-specific chaperone A | TBCA | -1.07 | 0.97 | -0.51 | 0.98 | -0.71 | -0.58 | 1.94 | -0.60 | -0.65 | 1.30 | -0.58 | -0.59 |
| Q8NFH8 | RalBP1-associated Eps domain-containing protein 2 | REPS2 | -1.08 | 2.52 | 1.58 | 1.34 | -0.27 | 0.04 | -0.47 | 0.64 | -0.11 | -1.87 | -0.20 | -0.68 |
| Q8TDC3 | Serine/threonine-protein kinase BRSK1 | BRSK1 | -1.08 | 2.28 | 0.62 | 0.91 | -1.06 | 0.86 | 0.75 | 0.17 | 0.11 | -2.02 | 0.60 | -0.93 |
| Q86XP3 | ATP-dependent RNA helicase DDX42 | DDX42 | -1.08 | 1.56 | 0.93 | 0.44 | -0.19 | -0.05 | 0.52 | 0.15 | -0.05 | -2.67 | 0.37 | 0.54 |
| P30876 | DNA-directed RNA polymerase II subunit RPB2 | POLR2B | -1.08 | 2.13 | 0.89 | 0.00 | -1.21 | 1.22 | 1.09 | 1.02 | -1.36 | -0.70 | -0.14 | -0.81 |
| Q9Y5Q9 | General transcription factor 3C polypeptide 3 | GTF3C3 | -1.08 | 1.03 | -1.44 | 0.70 | 0.67 | 0.73 | 0.52 | -1.36 | -1.51 | 0.34 | 0.76 | 0.60 |
| P42704 | Leucine-rich PPR motif-containing protein, mitochondrial | LRPPRC | -1.08 | 1.22 | -0.73 | 0.80 | 0.60 | 0.59 | 0.10 | -2.53 | 0.19 | -0.17 | 0.63 | 0.51 |
| Q96DZ5 | CAP-Gly domain-containing linker protein 3 | CLIP3 | -1.08 | 1.57 | 0.91 | 0.65 | -1.00 | 1.11 | -0.05 | -1.51 | -1.52 | 0.05 | 0.60 | 0.76 |
| Q12979 | Active breakpoint cluster region-related protein | ABR | -1.08 | 3.12 | 0.90 | 0.96 | -1.09 | 1.03 | 0.71 | -0.28 | -0.43 | -1.85 | 0.67 | -0.62 |
| P56545 | C-terminal-binding protein 2 | CTBP2 | -1.08 | 0.65 | -1.00 | 0.04 | 0.52 | 0.41 | 0.85 | -1.54 | -1.58 | 1.14 | 0.59 | 0.58 |
| Q3YEC7 | Rab-like protein 6 | RABL6 | -1.08 | 1.43 | -1.23 | 0.47 | 0.96 | 0.77 | 0.55 | -1.32 | -1.64 | 0.62 | 0.80 | 0.03 |
| O43432 | Eukaryotic translation initiation factor 4 gamma 3 | EIF4G3 | -1.09 | 1.29 | 1.11 | 0.60 | -0.13 | -0.04 | -0.11 | 0.72 | 0.78 | -0.09 | -0.38 | -2.47 |
| Q9Y3A3 | MOB-like protein phocein | MOB4 | -1.09 | 2.02 | 2.09 | 0.15 | -0.02 | 0.03 | -0.33 | -0.40 | 0.30 | -1.92 | -0.47 | 0.58 |
| P09496 | Clathrin light chain A | CLTA | -1.09 | 1.55 | -0.93 | 0.87 | -0.22 | -0.15 | 2.05 | -0.94 | -0.89 | 0.82 | 0.24 | -0.83 |
| Q9H3S7 | Tyrosine-protein phosphatase non-receptor type 23 | PTPN23 | -1.09 | 3.57 | -0.28 | 1.28 | 1.03 | 0.72 | 0.01 | -2.20 | -0.83 | -0.06 | 0.41 | -0.08 |
| P49916 | DNA ligase 3 | LIG3 | -1.09 | 2.10 | 1.29 | 1.71 | -1.44 | 0.51 | -0.04 | 0.02 | -1.25 | -0.70 | 0.08 | -0.17 |
| O60493 | Sorting nexin-3 | SNX3 | -1.09 | 3.02 | 1.46 | 0.58 | -0.68 | 0.70 | 0.42 | -0.18 | -1.22 | -1.88 | 0.55 | 0.24 |
| Q6ZTN6 | Ankyrin repeat domain-containing protein 13D | ANKRD13D | -1.09 | 1.20 | 0.84 | 0.61 | -1.65 | 0.66 | 0.87 | 0.90 | -1.52 | -0.75 | 0.43 | -0.38 |
| Q16854 | Deoxyguanosine kinase, mitochondrial | DGUOK | -1.09 | 1.28 | -0.51 | -0.56 | 1.32 | -0.59 | 1.75 | -0.57 | -0.52 | -0.93 | -0.58 | 1.21 |
| P05060 | Secretogranin-1 | CHGB | -1.09 | 3.21 | 0.94 | 0.69 | 0.98 | -0.38 | 0.40 | 0.34 | -2.04 | -0.41 | -1.21 | 0.67 |
| P19827 | Inter-alpha-trypsin inhibitor heavy chain H1 | ITIH1 | -1.10 | 2.28 | -0.71 | -0.14 | 1.05 | 0.87 | 1.02 | -1.51 | -1.57 | 0.33 | 0.83 | -0.17 |
| Q9H2G2 | STE20-like serine/threonine-protein kinase | SLK | -1.10 | 1.14 | 0.84 | 0.74 | 0.42 | 0.02 | -0.72 | 0.66 | 0.53 | -2.49 | -0.28 | 0.27 |
| Q14BN4 | Sarcolemmal membrane-associated protein | SLMAP | -1.10 | 2.05 | -0.02 | 1.44 | -1.43 | 0.48 | 1.50 | -0.82 | -0.15 | -1.02 | 0.58 | -0.58 |
| Q9UQL6 | Histone deacetylase 5 | HDAC5 | -1.10 | 1.61 | -0.68 | 0.40 | 0.01 | 1.29 | 0.64 | -1.77 | -1.33 | -0.12 | 1.08 | 0.47 |
| O96000 | NADH dehydrogenase [ubiquinone] 1 beta subcomplex subunit 10 | NDUFB10 | -1.10 | 1.79 | 0.29 | 1.45 | -1.31 | 0.55 | 0.80 | -0.23 | 0.05 | -1.42 | -1.10 | 0.94 |
| Q9H582 | Zinc finger protein 644 | ZNF644 | -1.10 | 1.61 | -0.89 | 0.49 | 0.99 | 0.28 | 0.79 | -2.23 | -0.62 | 0.72 | 0.62 | -0.14 |
| Q5VTU8 | ATP synthase subunit epsilon-like protein, mitochondrial | ATP5F1EP2 | -1.10 | 1.59 | -0.94 | 0.64 | 0.99 | 0.96 | -0.01 | -1.07 | -1.09 | -1.30 | 1.19 | 0.62 |
| Q8N9B5 | Junction-mediating and -regulatory protein | JMY | -1.10 | 1.40 | -0.71 | 1.21 | -0.65 | 0.90 | 0.74 | -0.81 | 0.87 | -1.80 | 0.67 | -0.42 |
| Q9UHB6 | LIM domain and actin-binding protein 1 | LIMA1 | -1.10 | 1.77 | -1.22 | 1.13 | 0.54 | 0.74 | 0.56 | -1.46 | -1.30 | -0.45 | 0.70 | 0.76 |
| P43155 | Carnitine O-acetyltransferase | CRAT | -1.10 | 1.28 | 0.22 | 0.25 | 1.95 | 0.41 | -1.43 | -0.64 | -1.19 | 0.80 | 0.21 | -0.57 |
| Q8NHH9 | Atlastin-2 | ATL2 | -1.10 | 1.53 | 0.38 | 1.35 | -0.28 | 0.46 | -0.30 | -0.26 | 0.41 | -2.48 | 0.35 | 0.37 |
| Q9Y496 | Kinesin-like protein KIF3A | KIF3A | -1.11 | 1.92 | -0.84 | 1.10 | 0.38 | 0.59 | 0.64 | -1.81 | -1.45 | 0.36 | 0.23 | 0.78 |
| Q8WUH1 | Protein Churchill | CHURC1 | -1.11 | 1.20 | 0.45 | 0.18 | 1.17 | -1.25 | 0.78 | 0.59 | 0.17 | -1.51 | -1.38 | 0.82 |
| P51572 | B-cell receptor-associated protein 31 | BCAP31 | -1.11 | 1.71 | -0.04 | 1.01 | 0.56 | -1.23 | 1.41 | 0.16 | 0.71 | -1.29 | 0.10 | -1.39 |
| Q9NNW5 | WD repeat-containing protein 6 | WDR6 | -1.11 | 2.45 | 0.06 | -0.54 | -0.73 | 1.84 | 1.62 | -0.58 | -0.64 | -0.90 | 0.49 | -0.62 |
| Q14839 | Chromodomain-helicase-DNA-binding protein 4 | CHD4 | -1.11 | 1.00 | 0.38 | -1.11 | 0.69 | 0.25 | 0.95 | -0.36 | -2.19 | 1.03 | 0.45 | -0.10 |
| Q9Y383 | Putative RNA-binding protein Luc7-like 2 | LUC7L2 | -1.11 | 3.61 | 1.14 | -0.91 | 0.30 | 0.63 | 1.57 | -0.07 | -1.37 | -1.39 | 0.23 | -0.14 |
| Q93015 | N-alpha-acetyltransferase 80 | NAA80 | -1.11 | 1.40 | -0.17 | 2.82 | -0.48 | -0.27 | -0.35 | -0.27 | -0.22 | -0.59 | -0.26 | -0.23 |
| Q9UJ04 | Testis-specific Y-encoded-like protein 4 | TSPYL4 | -1.11 | 1.98 | 0.18 | 0.59 | -1.32 | 0.86 | 1.58 | 0.90 | -0.74 | -0.95 | 0.07 | -1.18 |
| Q8N6D5 | Ankyrin repeat domain-containing protein 29 | ANKRD29 | -1.11 | 2.37 | -0.25 | 0.49 | 0.98 | 0.89 | 0.09 | -0.07 | -2.56 | -0.19 | 0.56 | 0.05 |
| Q14244 | Ensconsin | MAP7 | -1.11 | 3.21 | 1.57 | 0.38 | 0.30 | 0.38 | -0.01 | -0.31 | -0.66 | -2.31 | 0.18 | 0.49 |
| Q9UPQ9 | Trinucleotide repeat-containing gene 6B protein | TNRC6B | -1.11 | 3.03 | 2.28 | 0.79 | -0.70 | -0.58 | 0.87 | -0.40 | -0.47 | -0.87 | -0.49 | -0.44 |
| Q15084 | Protein disulfide-isomerase A6 | PDIA6 | -1.12 | 1.95 | -0.14 | -0.25 | 2.80 | -0.14 | -0.29 | -0.33 | -0.31 | -0.76 | -0.35 | -0.23 |
| Q9NP64 | Nucleolar protein of 40 kDa | ZCCHC17 | -1.12 | 1.20 | -0.84 | 0.42 | 0.94 | 0.51 | 0.32 | -0.80 | -2.25 | 0.89 | 0.27 | 0.56 |
| P43246 | DNA mismatch repair protein Msh2 | MSH2 | -1.12 | 1.98 | 0.36 | 1.12 | -0.95 | 0.78 | 0.60 | -0.38 | 0.26 | -2.25 | 0.66 | -0.20 |
| Q9NXH9 | tRNA (guanine(26)-N(2))-dimethyltransferase | TRMT1 | -1.12 | 1.64 | 1.34 | -0.47 | -0.70 | -0.48 | 2.00 | -0.49 | 0.80 | -0.83 | -0.60 | -0.58 |
| P67870 | Casein kinase II subunit beta | CSNK2B | -1.12 | 2.56 | 1.33 | 0.77 | -0.38 | -0.25 | 0.79 | -2.14 | 0.72 | -0.54 | -0.60 | 0.30 |
| P18505 | Gamma-aminobutyric acid receptor subunit beta-1 | GABRB1 | -1.12 | 3.23 | 1.38 | -1.08 | 0.43 | 0.81 | 1.03 | 0.58 | -0.77 | -1.02 | 0.08 | -1.44 |
| Q99574 | Neuroserpin | SERPINI1 | -1.12 | 2.53 | -0.13 | 0.84 | 2.58 | -0.38 | -0.52 | -0.45 | -0.43 | -0.73 | -0.35 | -0.42 |
| Q96R05 | Retinoid-binding protein 7 | RBP7 | -1.12 | 1.24 | -0.83 | 0.82 | -0.53 | 1.10 | 0.79 | -0.96 | -0.99 | -1.25 | 1.35 | 0.50 |
| Q7Z4S6 | Kinesin-like protein KIF21A | KIF21A | -1.12 | 2.18 | 1.30 | 0.79 | -0.91 | 0.79 | 0.05 | 0.68 | -0.30 | -1.92 | 0.42 | -0.91 |
| Q9UHD9 | Ubiquilin-2 | UBQLN2 | -1.12 | 1.24 | 0.77 | 0.84 | -0.16 | 0.36 | -0.42 | 0.23 | 0.65 | -2.60 | 0.33 | 0.00 |
| P45985 | Dual specificity mitogen-activated protein kinase kinase 4 | MAP2K4 | -1.12 | 1.80 | -0.03 | 1.66 | -0.37 | 0.66 | -0.13 | -0.13 | -0.01 | -2.30 | 0.60 | 0.05 |
| O60831 | PRA1 family protein 2 | PRAF2 | -1.12 | 3.01 | -0.18 | -0.02 | 0.34 | 0.34 | 1.98 | -1.32 | 0.05 | -1.71 | 0.32 | 0.19 |
| P23435 | Cerebellin-1 | CBLN1 | -1.12 | 1.09 | 1.69 | -0.81 | -0.94 | 0.39 | 0.91 | 1.16 | -0.72 | -1.04 | -0.81 | 0.17 |
| P98077 | SHC-transforming protein 2 | SHC2 | -1.13 | 2.20 | 0.97 | 2.54 | -0.50 | -0.43 | -0.41 | -0.34 | -0.34 | -0.69 | -0.37 | -0.43 |
| Q9BXB5 | Oxysterol-binding protein-related protein 10 | OSBPL10 | -1.13 | 1.38 | 0.62 | 0.56 | -0.80 | 0.37 | 0.74 | -0.31 | 1.37 | -2.21 | -0.02 | -0.32 |
| Q9NXR7 | BRISC and BRCA1-A complex member 2 | BABAM2 | -1.13 | 1.64 | 0.61 | -0.52 | 0.87 | 0.33 | 0.42 | -1.45 | -0.51 | -1.73 | 1.04 | 0.94 |
| P67775 | Serine/threonine-protein phosphatase 2A catalytic subunit alpha is | PPP2CA | -1.13 | 3.32 | 0.93 | 1.20 | -0.46 | 0.88 | 0.10 | -1.74 | 0.48 | -1.37 | 0.54 | -0.56 |
| Q5XUX1 | F-box/WD repeat-containing protein 9 | FBXW9 | -1.13 | 1.94 | -0.59 | 1.68 | 0.70 | -0.68 | 0.75 | -0.63 | -0.73 | -1.13 | 1.29 | -0.68 |
| Q96IZ7 | Serine/Arginine-related protein 53 | RSRC1 | -1.13 | 2.26 | 0.58 | -1.01 | 0.14 | 0.37 | 2.00 | -0.01 | -1.02 | 0.82 | -0.99 | -0.88 |
| Q14964 | Ras-related protein Rab-39A | RAB39A | -1.13 | 1.43 | 1.01 | 1.53 | -1.04 | 0.80 | -0.79 | -0.77 | -0.88 | -1.08 | 0.68 | 0.54 |
| Q86VP3 | Phosphofurin acidic cluster sorting protein 2 | PACS2 | -1.13 | 2.21 | -1.09 | 0.68 | 0.31 | 1.29 | 0.84 | -1.17 | -1.52 | -0.42 | 1.02 | 0.04 |
| P23677 | Inositol-trisphosphate 3-kinase A | ITPKA | -1.13 | 2.61 | -0.09 | 1.23 | 0.23 | 0.85 | 0.11 | -1.62 | 0.64 | -1.85 | 0.48 | 0.02 |
| Q9NQC3 | Reticulon-4 | RTN4 | -1.13 | 3.85 | 0.09 | 1.65 | 0.27 | 1.14 | -0.33 | -0.54 | -1.47 | -1.36 | 0.69 | -0.15 |
| Q7L3S4 | Zinc finger protein 771 | ZNF771 | -1.13 | 1.37 | -1.47 | 1.24 | -0.02 | 0.70 | 1.02 | -0.31 | -1.61 | -0.68 | 0.52 | 0.60 |

| | | | | | | | | | | | | | | |
|---|---|---|---|---|---|---|---|---|---|---|---|---|---|---|
| O00151 | PDZ and LIM domain protein 1 | PDLIM1 | -1.13 | 1.56 | -0.13 | 2.82 | -0.36 | -0.32 | -0.33 | -0.22 | -0.26 | -0.63 | -0.30 | -0.29 |
| P42858 | Huntingtin | HTT | -1.13 | 1.83 | 0.40 | 1.00 | 0.28 | 0.24 | -0.05 | -0.27 | 0.01 | -2.64 | 0.28 | 0.75 |
| P41217 | OX-2 membrane glycoprotein | CD200 | -1.14 | 2.05 | 0.85 | 2.57 | -0.53 | -0.31 | -0.53 | -0.32 | -0.37 | -0.76 | -0.32 | -0.30 |
| Q16864 | V-type proton ATPase subunit F | ATP6V1F | -1.14 | 1.15 | 0.80 | 0.88 | -1.29 | -0.61 | 1.50 | -1.09 | -1.12 | 0.88 | -0.14 | 0.19 |
| O60711 | Leupaxin | LPXN | -1.14 | 2.34 | 0.00 | 0.92 | -1.04 | 0.90 | 1.33 | -1.12 | -0.81 | -1.17 | -0.11 | 1.10 |
| Q13526 | Peptidyl-prolyl cis-trans isomerase NIMA-interacting 1 | PIN1 | -1.14 | 3.20 | 0.88 | 1.66 | -0.43 | 0.59 | -0.14 | -0.37 | -0.16 | -2.14 | 0.35 | -0.23 |
| Q7Z3B1 | Neuronal growth regulator 1 | NEGR1 | -1.14 | 1.81 | 0.27 | 1.76 | -0.78 | 0.27 | 0.26 | -0.31 | -1.94 | 0.88 | 0.21 | -0.62 |
| Q9HCJ6 | Synaptic vesicle membrane protein VAT-1 homolog-like | VAT1L | -1.14 | 2.07 | -0.71 | -0.46 | 1.76 | 0.07 | 1.30 | -1.64 | -0.68 | 0.49 | 0.16 | -0.29 |
| Q9NYV4 | Cyclin-dependent kinase 12 | CDK12 | -1.15 | 1.26 | 0.69 | -0.69 | -0.80 | 0.19 | 1.98 | -0.67 | 1.20 | -1.02 | -0.23 | -0.66 |
| P49840 | Glycogen synthase kinase-3 alpha | GSK3A | -1.15 | 1.35 | -1.11 | 0.36 | 0.26 | 1.06 | 0.88 | -1.68 | -1.16 | -0.16 | 1.14 | 0.41 |
| Q14C86 | GTPase-activating protein and VPS9 domain-containing protein 1 | GAPVD1 | -1.15 | 1.28 | -0.66 | 0.70 | 0.62 | 0.57 | 0.19 | -1.19 | -2.04 | 0.07 | 0.56 | 1.18 |
| Q14566 | DNA replication licensing factor MCM6 | MCM6 | -1.15 | 2.00 | 0.36 | -0.18 | -1.15 | 1.94 | 0.95 | 0.65 | -0.23 | -1.17 | -0.97 | -0.21 |
| Q96BH1 | E3 ubiquitin-protein ligase RNF25 | RNF25 | -1.15 | 1.70 | 1.47 | 2.00 | -0.69 | -0.49 | -0.54 | 0.60 | -0.58 | -0.84 | -0.46 | -0.48 |
| Q8N163 | Cell cycle and apoptosis regulator protein 2 | CCAR2 | -1.15 | 1.32 | -0.59 | -0.12 | 1.03 | 0.32 | 0.81 | -1.04 | -2.10 | 1.04 | 0.38 | 0.28 |
| Q12873 | Chromodomain-helicase-DNA-binding protein 3 | CHD3 | -1.16 | 1.44 | 0.96 | 0.18 | 0.56 | -0.55 | 0.41 | 0.99 | 0.78 | -1.78 | -1.58 | 0.04 |
| O95822 | Malonyl-CoA decarboxylase, mitochondrial | MLYCD | -1.16 | 2.04 | 0.68 | 1.03 | 1.15 | 0.07 | -1.00 | -1.04 | -0.96 | -1.42 | 0.65 | 0.84 |
| Q14149 | MORC family CW-type zinc finger protein 3 | MORC3 | -1.16 | 0.98 | 1.64 | -1.09 | -0.53 | 0.38 | 0.75 | 1.48 | -0.63 | -0.65 | -0.31 | -1.02 |
| Q13330 | Metastasis-associated protein MTA1 | MTA1 | -1.16 | 1.50 | 0.83 | 1.32 | -1.28 | 0.88 | -0.19 | 0.74 | -1.14 | -1.23 | -0.58 | 0.65 |
| Q8IVN3 | Musculoskeletal embryonic nuclear protein 1 | MUSTN1 | -1.16 | 1.58 | -0.18 | -0.23 | -0.42 | -0.28 | 2.83 | -0.23 | -0.32 | -0.56 | -0.31 | -0.29 |
| Q96NX5 | Calcium/calmodulin-dependent protein kinase type 1G | CAMK1G | -1.16 | 2.08 | 2.05 | 0.61 | -0.89 | 0.85 | -0.63 | -0.63 | -0.63 | -0.89 | 0.75 | -0.59 |
| Q9UMF0 | Intercellular adhesion molecule 5 | ICAM5 | -1.16 | 1.84 | 0.03 | 1.47 | 0.05 | 0.88 | -0.62 | -1.47 | -0.76 | 1.47 | -0.85 | -0.19 |
| Q9H936 | Mitochondrial glutamate carrier 1 | SLC25A22 | -1.16 | 1.03 | -1.55 | 0.70 | 0.42 | 0.83 | 0.79 | -0.72 | -1.82 | 0.08 | 0.62 | 0.65 |
| Q96T17 | MAP7 domain-containing protein 2 | MAP7D2 | -1.16 | 1.02 | -1.00 | 0.70 | 0.56 | 0.60 | 0.33 | -1.99 | -1.18 | 0.48 | 0.68 | 0.82 |
| P46459 | Vesicle-fusing ATPase | NSF | -1.17 | 1.89 | 0.18 | 1.43 | -0.26 | 0.57 | -0.05 | 1.06 | -0.39 | -2.19 | 0.28 | -0.62 |
| Q6YP21 | Kynurenine--oxoglutarate transaminase 3 | KYAT3 | -1.17 | 2.54 | 0.39 | 1.37 | 0.61 | -0.38 | 0.30 | -1.98 | 0.74 | 0.17 | 0.15 | -1.35 |
| Q13332 | Receptor-type tyrosine-protein phosphatase S | PTPRS | -1.17 | 2.19 | -0.11 | 1.79 | 0.69 | 0.81 | -1.14 | -0.97 | -0.47 | -1.35 | 0.61 | 0.14 |
| O95218 | Zinc finger Ran-binding domain-containing protein 2 | ZRANB2 | -1.17 | 1.81 | -0.63 | 0.84 | 0.12 | 0.52 | 0.96 | -2.20 | -1.03 | 0.27 | 0.42 | 0.72 |
| Q14671 | Pumilio homolog 1 | PUM1 | -1.17 | 1.97 | 0.00 | -0.78 | 2.07 | 0.18 | 0.42 | -1.54 | -1.07 | 0.53 | 0.27 | -0.08 |
| P32780 | General transcription factor IIH subunit 1 | GTF2H1 | -1.17 | 1.73 | -0.73 | 0.93 | 1.60 | 0.85 | -0.92 | -0.95 | -0.87 | 0.83 | 0.27 | -1.02 |
| Q53H96 | Pyrroline-5-carboxylate reductase 3 | PYCR3 | -1.17 | 1.62 | -0.56 | -0.69 | 0.06 | 1.14 | 1.69 | -1.11 | -0.64 | 0.12 | 1.08 | -1.11 |
| O43739 | Cytohesin-3 | CYTH3 | -1.17 | 2.65 | -0.24 | 2.13 | -0.61 | -0.38 | 1.53 | -0.37 | -0.37 | -1.00 | -0.38 | -0.32 |
| Q6ZVD8 | PH domain leucine-rich repeat-containing protein phosphatase 2 | PHLPP2 | -1.17 | 1.61 | 0.96 | 0.40 | -1.20 | 0.68 | 0.81 | -1.04 | 1.14 | -1.29 | 0.55 | -0.99 |
| Q9NZC9 | SWI/SNF-related matrix-associated actin-dependent regulator of ( | SMARCAL1 | -1.17 | 1.73 | 2.14 | -0.71 | -0.86 | 0.41 | 0.78 | 0.69 | -0.63 | -1.07 | -0.12 | -0.61 |
| Q8IWS0 | PHD finger protein 6 | PHF6 | -1.18 | 2.41 | 1.61 | -0.50 | -0.66 | 0.33 | 1.38 | 0.30 | -1.80 | -0.60 | 0.09 | -0.15 |
| Q96P47 | Arf-GAP with GTPase, ANK repeat and PH domain-containing prot | AGAP3 | -1.18 | 3.40 | 1.24 | 0.67 | -0.40 | 0.66 | 0.54 | -0.16 | 0.56 | -2.00 | 0.21 | -1.33 |
| O95319 | CUGBP Elav-like family member 2 | CELF2 | -1.18 | 2.35 | 0.99 | -0.78 | -0.59 | 0.88 | 1.62 | -1.46 | -0.36 | -0.94 | 0.71 | -0.07 |
| Q9BWF3 | RNA-binding protein 4 | RBM4 | -1.18 | 1.20 | 1.01 | -0.23 | -1.01 | 0.65 | 0.91 | 0.40 | 0.09 | -2.32 | 0.31 | 0.20 |
| Q9H7Z3 | Protein NRDE2 homolog | NRDE2 | -1.18 | 0.93 | -1.38 | 0.17 | 0.34 | 1.08 | 0.86 | -1.37 | -1.40 | 0.18 | 0.91 | 0.60 |
| Q9P265 | Disco-interacting protein 2 homolog B | DIP2B | -1.18 | 5.26 | -0.37 | 0.92 | 1.05 | 0.84 | 0.91 | -1.84 | -0.82 | -0.79 | 0.64 | -0.54 |
| Q49A26 | Putative oxidoreductase GLYR1 | GLYR1 | -1.18 | 1.91 | 0.18 | 0.35 | 0.69 | 0.20 | 0.55 | -0.40 | -2.66 | 0.72 | 0.52 | -0.14 |
| Q96J01 | THO complex subunit 3 | THOC3 | -1.18 | 2.01 | 0.83 | 0.30 | -0.74 | 0.68 | 0.87 | -1.22 | -0.87 | -1.53 | 1.32 | 0.34 |
| Q6P2E9 | Enhancer of mRNA-decapping protein 4 | EDC4 | -1.19 | 1.14 | 0.28 | 1.16 | 0.73 | -0.40 | -0.47 | 0.28 | 0.93 | -2.17 | 0.53 | -0.86 |
| Q9NWB6 | Arginine and glutamate-rich protein 1 | ARGLU1 | -1.19 | 1.06 | 0.00 | 0.02 | 0.40 | 0.33 | 0.50 | -2.69 | -0.24 | 0.95 | 0.39 | 0.34 |
| Q2TAK8 | PWWP domain-containing DNA repair factor 3A | PWWP3A | -1.19 | 1.45 | 0.62 | 0.94 | -0.22 | 0.56 | -0.34 | -1.53 | 0.13 | -1.80 | 0.43 | 1.21 |
| Q96DH6 | RNA-binding protein Musashi homolog 2 | MSI2 | -1.19 | 1.88 | 0.46 | 0.29 | -0.56 | 0.76 | 0.93 | -0.41 | -2.26 | -0.72 | 0.71 | 0.81 |
| Q9HCK8 | Chromodomain-helicase-DNA-binding protein 8 | CHD8 | -1.19 | 1.56 | 1.46 | -0.34 | -1.00 | 0.57 | 0.92 | 1.14 | -0.93 | -1.29 | 0.30 | -0.83 |
| O00192 | Armadillo repeat protein deleted in velo-cardio-facial syndrome | ARVCF | -1.19 | 1.50 | 0.90 | 1.28 | -1.36 | 0.60 | 0.13 | -0.34 | -1.16 | -1.39 | 0.53 | 0.80 |
| P42166 | Lamina-associated polypeptide 2, isoform alpha | TMPO | -1.19 | 1.91 | -0.90 | 1.02 | 1.02 | 0.50 | 0.22 | -1.35 | -1.80 | 0.09 | 0.67 | 0.53 |
| Q9Y5A9 | YTH domain-containing family protein 2 | YTHDF2 | -1.19 | 1.90 | 1.04 | -0.21 | -0.68 | 0.47 | 1.23 | -0.05 | 0.48 | -2.26 | 0.40 | -0.42 |
| P33402 | Guanylate cyclase soluble subunit alpha-2 | GUCY1A2 | -1.19 | 2.60 | 0.83 | 0.31 | -0.09 | 0.80 | 0.52 | -0.34 | -0.95 | -2.23 | 0.01 | 1.14 |
| Q15139 | Serine/threonine-protein kinase D1 | PRKD1 | -1.19 | 1.79 | -0.57 | 1.29 | 0.08 | 0.66 | 0.33 | 0.12 | -2.32 | -0.65 | 0.35 | 0.71 |
| Q9UII2 | ATPase inhibitor, mitochondrial | ATP5IF1 | -1.20 | 2.00 | 0.57 | -1.30 | 0.12 | 0.54 | 2.01 | -1.11 | -0.45 | 0.42 | 0.20 | -1.01 |
| Q13574 | Diacylglycerol kinase zeta | DGKZ | -1.20 | 1.23 | 1.61 | -0.01 | -0.47 | 0.44 | -0.21 | 1.12 | 0.13 | -0.69 | 0.14 | -2.05 |
| Q9BX40 | Protein LSM14 homolog B | LSM14B | -1.20 | 0.81 | 1.25 | -0.29 | -0.90 | 0.23 | 0.68 | -0.79 | 1.90 | -1.04 | -0.26 | -0.80 |
| Q9UPT8 | Zinc finger CCCH domain-containing protein 4 | ZC3H4 | -1.20 | 2.35 | -0.11 | 0.75 | -0.53 | -0.45 | 2.60 | -0.34 | -0.42 | -0.76 | -0.29 | -0.46 |
| P67809 | Nuclease-sensitive element-binding protein 1 | YBX1 | -1.20 | 1.99 | -0.52 | 0.66 | 1.73 | -0.46 | 0.48 | -0.14 | -1.28 | -1.58 | 0.71 | 0.40 |
| Q9BVC5 | Ashwin | C2orf49 | -1.20 | 2.98 | 0.83 | 0.64 | -1.08 | 0.60 | 1.46 | -0.34 | -0.95 | -1.28 | 0.94 | -0.82 |
| P43007 | Neutral amino acid transporter A | SLC1A4 | -1.20 | 2.17 | -0.45 | 1.68 | -0.56 | -0.57 | 2.03 | -0.51 | -0.62 | 0.05 | -0.63 | -0.42 |
| Q8WY36 | HMG box transcription factor BBX | BBX | -1.20 | 2.01 | -0.64 | 1.09 | -0.98 | 0.78 | 1.67 | -0.03 | -0.78 | -1.18 | 0.74 | -0.68 |
| Q9UMS4 | Pre-mRNA-processing factor 19 | PRPF19 | -1.20 | 2.46 | 0.00 | 0.14 | 2.00 | 0.37 | -0.33 | -1.05 | -1.72 | -0.41 | 0.46 | 0.54 |
| Q9BXM9 | FSD1-like protein | FSD1L | -1.20 | 1.97 | 1.57 | 0.47 | -0.42 | 0.35 | -0.06 | 0.69 | -0.38 | -2.32 | 0.06 | 0.05 |
| Q00536 | Cyclin-dependent kinase 16 | CDK16 | -1.21 | 2.89 | 0.26 | -0.17 | 1.66 | 0.94 | -0.28 | -1.20 | -1.14 | -0.52 | 1.24 | -0.79 |
| O43390 | Heterogeneous nuclear ribonucleoprotein R | HNRNPR | -1.21 | 1.88 | -0.43 | -0.10 | 1.44 | 0.65 | 0.29 | -1.25 | -1.87 | 0.59 | 0.89 | -0.22 |
| Q96J02 | E3 ubiquitin-protein ligase Itchy homolog | ITCH | -1.21 | 1.76 | 0.20 | 1.30 | -0.19 | 0.31 | 0.17 | -0.14 | 0.09 | -2.58 | 0.56 | 0.28 |
| Q8WXA9 | Splicing regulatory glutamine/lysine-rich protein 1 | SREK1 | -1.21 | 1.73 | -1.08 | 1.06 | -0.18 | 0.81 | 1.12 | -1.73 | -0.94 | -0.31 | 0.69 | 0.56 |
| O95819 | Mitogen-activated protein kinase kinase kinase kinase 4 | MAP4K4 | -1.21 | 1.67 | -0.03 | 0.79 | -0.29 | 0.92 | 0.35 | -0.40 | -2.46 | -0.24 | 0.89 | 0.49 |
| Q8N3C0 | Activating signal cointegrator 1 complex subunit 3 | ASCC3 | -1.21 | 1.54 | 0.01 | 1.51 | -1.33 | 0.78 | 0.63 | -0.24 | -1.08 | -1.43 | 0.62 | 0.53 |
| Q6NV74 | Uncharacterized protein KIAA1211-like | KIAA1211L | -1.21 | 2.17 | 0.83 | 1.35 | -0.77 | 0.23 | 0.40 | -0.45 | 1.18 | -1.88 | -0.16 | -0.72 |
| P63027 | Vesicle-associated membrane protein 2 | VAMP2 | -1.21 | 2.55 | 0.65 | 1.86 | -0.12 | -0.10 | -0.06 | -1.14 | 0.97 | -1.66 | -0.10 | -0.30 |
| Q00975 | Voltage-dependent N-type calcium channel subunit alpha-1B | CACNA1B | -1.21 | 1.54 | -0.42 | 1.99 | -0.74 | -0.58 | 1.38 | -0.56 | -0.58 | -0.77 | 0.77 | -0.48 |
| Q9NUB1 | Acetyl-coenzyme A synthetase 2-like, mitochondrial | ACSS1 | -1.21 | 1.67 | 0.57 | -0.68 | 0.61 | 0.30 | 0.92 | 1.37 | 0.61 | -1.46 | -1.08 | -1.17 |
| Q9Y4H2 | Insulin receptor substrate 2 | IRS2 | -1.22 | 2.35 | -0.51 | -0.41 | 0.48 | 1.09 | 1.47 | -1.94 | -0.50 | -0.40 | 0.89 | -0.17 |
| Q9NYB0 | Telomeric repeat-binding factor 2-interacting protein 1 | TERF2IP | -1.22 | 3.64 | 1.27 | -0.75 | 0.69 | 1.18 | 0.36 | 0.56 | -0.47 | -1.95 | -0.22 | -0.67 |
| O75689 | Arf-GAP with dual PH domain-containing protein 1 | ADAP1 | -1.22 | 3.66 | 0.27 | 0.83 | 0.61 | 1.20 | -0.04 | -0.61 | -0.12 | -2.07 | 0.90 | -0.97 |
| Q13596 | Sorting nexin-1 | SNX1 | -1.22 | 0.94 | -1.05 | 0.15 | 1.51 | 0.51 | -0.02 | -1.72 | -1.15 | 0.65 | 0.64 | 0.48 |
| Q6P6B7 | Ankyrin repeat domain-containing protein 16 | ANKRD16 | -1.22 | 1.57 | 1.26 | -0.96 | -1.02 | 1.12 | 1.23 | 0.37 | 0.26 | -1.24 | -0.97 | -0.04 |
| P29558 | RNA-binding motif, single-stranded-interacting protein 1 | RBMS1 | -1.22 | 1.10 | 2.04 | -0.53 | -0.71 | -0.57 | 1.02 | -0.50 | -0.56 | -0.75 | -0.56 | 1.12 |
| Q7Z417 | Nuclear fragile X mental retardation-interacting protein 2 | NUFIP2 | -1.22 | 1.14 | -1.42 | 0.59 | 0.69 | 0.64 | 0.78 | -1.23 | -1.63 | 0.22 | 0.69 | 0.67 |
| Q9Y5X0 | Sorting nexin-10 | SNX10 | -1.22 | 2.29 | 2.46 | -0.45 | -0.55 | 1.07 | -0.32 | -0.31 | -0.30 | -0.84 | -0.43 | -0.34 |
| P53365 | Arfaptin-2 | ARFIP2 | -1.23 | 1.92 | 0.53 | 0.19 | -1.08 | 1.23 | 0.98 | -0.54 | 0.46 | -1.90 | 0.74 | -0.62 |
| Q9BZ95 | Histone-lysine N-methyltransferase NSD3 | NSD3 | -1.23 | 1.82 | 1.22 | 1.01 | -1.51 | 0.09 | 0.99 | 0.01 | -1.44 | -0.97 | 0.43 | 0.17 |
| Q08378 | Golgin subfamily A member 3 | GOLGA3 | -1.23 | 4.23 | 1.07 | 0.21 | 1.42 | 0.47 | -0.16 | -0.60 | -1.15 | 0.44 | 0.21 | -1.91 |
| O14734 | Acyl-coenzyme A thioesterase 8 | ACOT8 | -1.23 | 2.06 | 0.61 | 0.31 | 0.91 | -0.45 | 0.63 | -0.40 | 0.83 | -2.47 | 0.29 | -0.26 |

| | | | | | | | | | | | | | | |
|---|---|---|---|---|---|---|---|---|---|---|---|---|---|---|
| P0DPH8 | Tubulin alpha-3D chain | TUBA3D | -1.23 | 1.48 | 1.09 | -0.01 | 0.14 | 0.71 | -0.35 | 0.45 | -2.56 | -0.20 | 0.58 | 0.15 |
| Q15276 | Rab GTPase-binding effector protein 1 | RABEP1 | -1.23 | 2.00 | -0.84 | 1.53 | -0.19 | 1.10 | 0.31 | -0.96 | -1.46 | -0.82 | 0.74 | 0.59 |
| Q9UPN9 | E3 ubiquitin-protein ligase TRIM33 | TRIM33 | -1.23 | 1.84 | 0.63 | -0.92 | 0.26 | 0.54 | 1.30 | -1.16 | -1.89 | 0.05 | 0.77 | 0.42 |
| P04049 | RAF proto-oncogene serine/threonine-protein kinase | RAF1 | -1.23 | 2.92 | 0.26 | 0.85 | -0.34 | 1.00 | 0.72 | -2.26 | -0.09 | -0.97 | 0.70 | 0.13 |
| P28472 | Gamma-aminobutyric acid receptor subunit beta-3 | GABRB3 | -1.23 | 2.46 | 0.34 | 1.04 | -0.82 | 1.02 | 0.62 | -2.12 | -0.03 | -0.17 | 0.83 | -0.70 |
| Q8NI36 | WD repeat-containing protein 36 | WDR36 | -1.23 | 2.67 | 1.79 | -0.32 | -0.57 | 1.96 | -0.39 | -0.42 | -0.33 | -0.83 | -0.45 | -0.44 |
| Q14192 | Four and a half LIM domains protein 2 | FHL2 | -1.24 | 1.66 | -1.41 | 0.99 | 0.67 | 0.70 | 0.72 | -1.24 | -1.39 | -0.42 | 0.59 | 0.78 |
| Q8NFX7 | Syntaxin-binding protein 6 | STXBP6 | -1.24 | 3.23 | 0.65 | 0.32 | 0.53 | 0.84 | 0.45 | -0.17 | -0.97 | -2.32 | 0.93 | -0.26 |
| Q96I24 | Far upstream element-binding protein 3 | FUBP3 | -1.24 | 2.21 | -0.64 | 0.70 | 0.62 | 0.68 | 0.72 | -1.08 | -2.09 | -0.27 | 1.00 | 0.34 |
| O60885 | Bromodomain-containing protein 4 | BRD4 | -1.24 | 2.21 | 0.85 | -0.56 | -0.68 | 2.43 | 0.09 | -0.55 | -0.49 | -0.91 | -0.41 | 0.23 |
| Q12800 | Alpha-globin transcription factor CP2 | TFCP2 | -1.24 | 2.42 | -0.34 | 0.68 | -0.07 | 1.27 | 0.66 | -0.29 | -1.13 | -2.03 | 0.84 | 0.42 |
| Q9Y314 | Nitric oxide synthase-interacting protein | NOSIP | -1.24 | 2.08 | 0.63 | 0.43 | -0.56 | 0.66 | 0.86 | 0.09 | -2.55 | -0.28 | 0.52 | 0.22 |
| Q8TEQ6 | Gem-associated protein 5 | GEMIN5 | -1.24 | 1.30 | -0.90 | -0.15 | 1.89 | 0.40 | 0.17 | -1.65 | -0.99 | 0.59 | 0.59 | 0.06 |
| Q06587 | E3 ubiquitin-protein ligase RING1 | RING1 | -1.24 | 2.72 | 1.19 | -0.69 | 0.87 | 0.68 | 0.30 | -0.09 | -2.26 | -0.50 | 0.65 | -0.14 |
| Q8NE62 | Choline dehydrogenase, mitochondrial | CHDH | -1.25 | 2.82 | 0.60 | -0.88 | 0.28 | 0.45 | 1.92 | -0.80 | -0.66 | -1.18 | 0.97 | -0.70 |
| Q7RTP6 | [F-actin]-monooxygenase MICAL3 | MICAL3 | -1.25 | 1.35 | -0.57 | 0.19 | 0.52 | 0.56 | 0.79 | -2.06 | -1.22 | 1.20 | 0.49 | 0.11 |
| Q9Y2I8 | WD repeat-containing protein 37 | WDR37 | -1.25 | 5.54 | -0.45 | 1.51 | 0.34 | 0.68 | 1.29 | -1.11 | -0.46 | -1.18 | 0.48 | -1.10 |
| Q96PE3 | Inositol polyphosphate-4-phosphatase type I A | INPP4A | -1.25 | 1.41 | 1.03 | 0.52 | 0.17 | 0.05 | -0.23 | 0.65 | 0.59 | -2.60 | 0.10 | -0.28 |
| Q9UJU3 | Zinc finger protein 112 | ZNF112 | -1.25 | 1.47 | 1.32 | -0.94 | -1.07 | 1.15 | 1.09 | 0.68 | -0.92 | 0.31 | -0.83 | -0.79 |
| P49591 | Serine--tRNA ligase, cytoplasmic | SARS | -1.25 | 1.57 | 0.04 | 0.59 | 0.72 | 0.43 | -0.10 | -0.38 | -2.63 | 0.38 | 0.83 | 0.11 |
| Q68DU8 | BTB/POZ domain-containing protein KCTD16 | KCTD16 | -1.25 | 2.63 | 0.19 | 1.73 | 0.01 | 0.63 | -0.26 | -0.06 | -2.16 | -0.76 | 0.46 | 0.23 |
| Q9Y426 | C2 domain-containing protein 2 | C2CD2 | -1.25 | 1.97 | 0.70 | 1.44 | -0.61 | 0.79 | -0.43 | 0.52 | -1.30 | -1.70 | -0.06 | 0.65 |
| Q6ZUM4 | Rho GTPase-activating protein 27 | ARHGAP27 | -1.25 | 1.80 | -0.39 | 0.31 | 2.38 | -0.60 | 0.10 | -0.50 | -0.64 | -0.97 | -0.57 | 0.88 |
| Q76I76 | Protein phosphatase Slingshot homolog 2 | SSH2 | -1.25 | 3.51 | 1.06 | 1.40 | -0.85 | 0.81 | 0.27 | -1.31 | -0.66 | -0.02 | 0.67 | -1.37 |
| O00483 | Cytochrome c oxidase subunit NDUFA4 | NDUFA4 | -1.25 | 0.71 | -1.38 | 0.68 | 0.31 | 0.71 | 0.55 | -1.45 | -1.47 | 0.59 | 0.79 | 0.67 |
| O94772 | Lymphocyte antigen 6H | LY6H | -1.25 | 2.21 | 0.03 | 1.57 | -0.22 | 0.69 | -0.02 | -2.44 | 0.07 | 0.16 | 0.14 | 0.02 |
| Q5H9R7 | Serine/threonine-protein phosphatase 6 regulatory subunit 3 | PPP6R3 | -1.25 | 1.59 | 0.57 | 0.81 | -1.44 | 0.91 | 0.79 | 0.03 | -1.24 | -1.51 | 0.76 | 0.34 |
| Q9NPF5 | DNA methyltransferase 1-associated protein 1 | DMAP1 | -1.25 | 1.77 | 1.19 | -0.10 | -0.93 | 0.54 | 1.04 | 0.89 | -1.70 | -0.88 | 0.62 | -0.69 |
| Q9NWV8 | BRISC and BRCA1-A complex member 1 | BABAM1 | -1.25 | 1.94 | 1.28 | 1.46 | -0.33 | 0.47 | -1.01 | -1.23 | 0.83 | -0.30 | 0.09 | -1.26 |
| Q96PX6 | Coiled-coil domain-containing protein 85A | CCDC85A | -1.25 | 3.04 | 0.14 | 1.90 | -0.95 | 0.38 | 1.04 | -0.70 | -0.78 | -1.10 | 0.72 | -0.65 |
| Q6P158 | Putative ATP-dependent RNA helicase DHX57 | DHX57 | -1.25 | 2.03 | 1.34 | -0.22 | 0.03 | 0.53 | 0.28 | -0.85 | 1.00 | -2.19 | 0.38 | -0.31 |
| Q8WUQ7 | Cactin | CACTIN | -1.25 | 1.18 | -0.35 | 0.59 | -1.09 | 0.71 | 1.44 | -0.95 | 1.20 | -1.31 | 0.46 | -0.70 |
| Q71RC2 | La-related protein 4 | LARP4 | -1.25 | 3.22 | 0.50 | 0.44 | -0.96 | 0.52 | 2.12 | -0.74 | -0.65 | -1.13 | -0.60 | 0.50 |
| P53804 | E3 ubiquitin-protein ligase TTC3 | TTC3 | -1.26 | 2.29 | -0.66 | 1.56 | 1.29 | 0.70 | -0.79 | -0.65 | -0.67 | -1.07 | 0.95 | -0.66 |
| P69891 | Hemoglobin subunit gamma-1 | HBG1 | -1.26 | 1.35 | -0.12 | 0.43 | 0.56 | 0.43 | 0.22 | -0.89 | -2.34 | 1.21 | 0.70 | -0.19 |
| Q9NQC7 | Ubiquitin carboxyl-terminal hydrolase CYLD | CYLD | -1.26 | 3.65 | -0.04 | 0.91 | 0.47 | 1.02 | 0.54 | -1.19 | 0.27 | -2.16 | 0.56 | -0.37 |
| P09497 | Clathrin light chain B | CLTB | -1.26 | 1.32 | -1.07 | 1.46 | -0.75 | -0.13 | 1.94 | -0.78 | 0.36 | 0.08 | -0.47 | -0.64 |
| Q9UHV9 | Prefoldin subunit 2 | PFDN2 | -1.26 | 1.94 | 0.46 | 0.51 | -0.12 | 0.14 | 0.95 | -1.43 | -2.13 | 0.69 | 0.19 | 0.73 |
| P05452 | Tetranectin | CLEC3B | -1.26 | 3.00 | -0.13 | -0.33 | 2.41 | 1.11 | -0.42 | -0.49 | -0.33 | -0.96 | -0.38 | -0.47 |
| O15294 | UDP-N-acetylglucosamine--peptide N-acetylglucosaminyltransfera | OGT | -1.26 | 5.52 | 0.66 | 1.32 | -0.06 | 1.02 | 0.51 | -1.02 | -0.70 | -1.73 | 0.70 | -0.70 |
| Q14376 | UDP-glucose 4-epimerase | GALE | -1.26 | 2.55 | 2.32 | -0.54 | 0.47 | -0.51 | 0.55 | -0.67 | -0.60 | -1.01 | -0.60 | 0.59 |
| P31040 | Succinate dehydrogenase [ubiquinone] flavoprotein subunit, mitocl | SDHA | -1.26 | 1.34 | -0.81 | 0.21 | 1.58 | 1.34 | -0.88 | -0.31 | -0.93 | -1.09 | 1.03 | -0.14 |
| B0I1T2 | Unconventional myosin-Ig | MYO1G | -1.26 | 2.52 | 0.88 | 2.30 | 0.65 | -0.69 | -0.78 | -0.71 | -0.58 | -0.21 | -0.71 | -0.15 |
| Q9NRL3 | Striatin-4 | STRN4 | -1.26 | 2.72 | 0.64 | 0.42 | 1.14 | 0.26 | -0.03 | 0.16 | 0.15 | -2.62 | -0.33 | 0.21 |
| P33992 | DNA replication licensing factor MCM5 | MCM5 | -1.26 | 2.49 | 1.81 | -0.59 | -0.80 | 0.92 | 0.88 | -0.59 | -0.72 | -1.05 | 0.82 | -0.68 |
| Q9BPX5 | Actin-related protein 2/3 complex subunit 5-like protein | ARPC5L | -1.26 | 1.97 | 0.29 | 1.43 | -0.59 | 0.22 | 0.56 | -2.31 | 0.56 | -0.65 | 0.23 | 0.26 |
| P09914 | Interferon-induced protein with tetratricopeptide repeats 1 | IFIT1 | -1.27 | 2.41 | 0.50 | 1.21 | -0.33 | 0.29 | 0.54 | 0.61 | -2.36 | -0.80 | 0.43 | -0.09 |
| Q6ULP2 | Aftiphilin | AFTPH | -1.27 | 1.15 | -0.52 | 0.31 | 0.62 | 0.68 | 0.23 | -2.30 | -0.97 | 0.77 | 0.89 | 0.30 |
| Q9Y484 | WD repeat domain phosphoinositide-interacting protein 4 | WDR45 | -1.27 | 2.19 | -0.24 | 0.47 | 1.60 | 0.36 | -0.15 | -1.58 | -1.61 | 0.48 | -0.16 | 0.82 |
| Q9NS69 | Mitochondrial import receptor subunit TOM22 homolog | TOMM22 | -1.27 | 1.34 | 2.11 | -0.52 | -0.64 | -0.57 | 1.08 | -0.51 | -0.52 | -0.78 | -0.59 | 0.94 |
| Q9Y2H1 | Serine/threonine-protein kinase 38-like | STK38L | -1.28 | 2.11 | -0.58 | -0.60 | 0.46 | 0.30 | 2.47 | -0.92 | -0.88 | -0.06 | 0.25 | -0.45 |
| Q9C0C7 | Activating molecule in BECN1-regulated autophagy protein 1 | AMBRA1 | -1.28 | 1.78 | 0.87 | -1.39 | 0.51 | 0.43 | 1.33 | 0.41 | 0.07 | -1.68 | -0.96 | 0.39 |
| P62166 | Neuronal calcium sensor 1 | NCS1 | -1.28 | 1.38 | 1.85 | -0.13 | -0.82 | -0.70 | 1.28 | 1.01 | -0.72 | -0.91 | -0.29 | -0.57 |
| Q9UQR1 | Zinc finger protein 148 | ZNF148 | -1.28 | 1.12 | -0.41 | -0.93 | 1.09 | -0.59 | 2.12 | 0.11 | -0.09 | 0.50 | -0.71 | -1.09 |
| P07477 | Trypsin-1 | PRSS1 | -1.28 | 1.72 | -0.75 | -0.63 | 0.60 | 0.08 | 2.49 | -0.76 | -0.79 | 0.22 | -0.01 | -0.44 |
| Q96SB3 | Neurabin-2 | PPP1R9B | -1.28 | 2.52 | 0.59 | 1.26 | -0.23 | 0.44 | 0.24 | 0.30 | 0.68 | -2.10 | 0.16 | -1.33 |
| Q969R2 | Oxysterol-binding protein 2 | OSBP2 | -1.28 | 4.68 | -0.37 | 1.76 | 0.14 | 0.20 | 1.43 | -1.39 | -1.28 | -0.02 | -0.27 | -0.21 |
| Q9HBZ2 | Aryl hydrocarbon receptor nuclear translocator 2 | ARNT2 | -1.29 | 1.84 | -0.48 | -0.16 | -0.70 | 1.38 | 1.78 | -0.66 | -0.67 | -0.95 | 1.04 | -0.57 |
| Q9BRG1 | Vacuolar protein-sorting-associated protein 25 | VPS25 | -1.29 | 2.58 | -0.21 | 2.08 | -0.66 | -0.43 | 1.62 | -0.33 | -0.31 | -0.86 | -0.46 | -0.44 |
| Q96RQ3 | Methylcrotonoyl-CoA carboxylase subunit alpha, mitochondrial | MCCC1 | -1.29 | 2.16 | -0.65 | 0.16 | 0.64 | 0.92 | 0.98 | -1.99 | -1.13 | -0.27 | 0.90 | 0.46 |
| O75044 | SLIT-ROBO Rho GTPase-activating protein 2 | SRGAP2 | -1.29 | 2.83 | -0.18 | 1.59 | -0.57 | 0.74 | 0.80 | -0.14 | -0.52 | -2.05 | 0.61 | -0.29 |
| Q2KHT3 | Protein CLEC16A | CLEC16A | -1.29 | 0.59 | -1.32 | -0.10 | 0.57 | 0.61 | 0.98 | -1.41 | -1.39 | 1.01 | 0.73 | 0.32 |
| O75064 | DENN domain-containing protein 4B | DENND4B | -1.29 | 2.13 | 0.44 | 2.05 | -0.96 | 0.66 | -0.19 | 0.26 | -0.82 | -1.23 | 0.57 | -0.78 |
| Q13469 | Nuclear factor of activated T-cells, cytoplasmic 2 | NFATC2 | -1.29 | 1.67 | -1.21 | 0.95 | 1.10 | 0.31 | 0.54 | -1.37 | -1.61 | 0.45 | 0.31 | 0.54 |
| Q96K17 | Transcription factor BTF3 homolog 4 | BTF3L4 | -1.30 | 2.38 | 0.02 | 0.25 | 1.82 | -0.20 | 0.26 | -0.60 | 0.33 | -1.81 | -0.97 | 0.90 |
| Q5THJ4 | Vacuolar protein sorting-associated protein 13D | VPS13D | -1.30 | 2.33 | 0.45 | 0.73 | 0.57 | 0.35 | 0.14 | 0.64 | -2.46 | -1.07 | 0.37 | 0.27 |
| Q9UPT9 | Ubiquitin carboxyl-terminal hydrolase 22 | USP22 | -1.30 | 2.39 | -0.21 | 0.31 | -0.82 | 0.95 | 1.92 | -1.08 | -0.58 | -0.95 | 0.97 | -0.51 |
| Q92529 | SHC-transforming protein 3 | SHC3 | -1.30 | 1.70 | 0.61 | 0.17 | 1.10 | -0.09 | -0.03 | 1.80 | -0.86 | -1.33 | -0.11 | -1.26 |
| Q70YC5 | Protein ZNF365 | ZNF365 | -1.30 | 2.94 | 0.69 | 0.43 | -0.33 | 0.62 | 1.10 | 0.87 | -0.07 | -2.29 | -0.71 | -0.32 |
| O15197 | Ephrin type-B receptor 6 | EPHB6 | -1.30 | 1.79 | 0.22 | 0.61 | -0.16 | 0.38 | 0.80 | -1.12 | -2.32 | 0.33 | 0.28 | 0.98 |
| P20290 | Transcription factor BTF3 | BTF3 | -1.30 | 1.64 | -0.15 | 0.17 | 1.73 | -0.49 | 0.40 | -1.12 | -0.37 | 0.22 | -1.61 | 1.22 |
| O95057 | GTP-binding protein Di-Ras1 | DIRAS1 | -1.30 | 2.90 | 0.51 | 0.73 | -0.56 | 1.31 | 0.46 | -0.29 | -0.64 | -2.19 | 0.82 | -0.15 |
| P08631 | Tyrosine-protein kinase HCK | HCK | -1.30 | 2.31 | -0.17 | 1.03 | -0.49 | 0.47 | 1.29 | -0.57 | -0.33 | -2.20 | 0.58 | 0.41 |
| Q6IQ55 | Tau-tubulin kinase 2 | TTBK2 | -1.31 | 2.15 | 1.59 | 1.82 | -0.90 | -0.79 | 0.37 | 0.04 | -0.86 | 0.02 | -0.61 | -0.69 |
| Q7Z6L1 | Tectonin beta-propeller repeat-containing protein 1 | TECPR1 | -1.31 | 1.51 | 1.48 | 1.29 | -0.41 | -0.41 | -0.39 | 0.92 | 0.38 | -1.47 | -0.19 | -1.20 |
| Q02539 | Histone H1.1 | HIST1H1A | -1.31 | 1.54 | -0.45 | 0.70 | 2.15 | -0.12 | -0.67 | -0.61 | -0.64 | -0.89 | 1.13 | -0.59 |
| P30414 | NK-tumor recognition protein | NKTR | -1.31 | 1.34 | -1.33 | 1.12 | 0.33 | 0.54 | 0.78 | -1.47 | -1.40 | 0.54 | 0.74 | 0.16 |
| O15327 | Inositol polyphosphate 4-phosphatase type II | INPP4B | -1.31 | 2.93 | -0.38 | 2.03 | 0.75 | 0.78 | -0.73 | -0.55 | -0.69 | -1.12 | -0.75 | 0.65 |
| Q8IVT5 | Kinase suppressor of Ras 1 | KSR1 | -1.31 | 1.67 | 1.26 | 1.12 | -1.25 | 0.73 | -0.17 | 0.67 | -1.11 | -1.33 | 0.59 | -0.49 |
| O95721 | Synaptosomal-associated protein 29 | SNAP29 | -1.31 | 2.21 | -0.33 | 1.63 | 0.64 | 0.08 | 0.02 | -0.38 | 0.69 | -2.25 | -0.28 | 0.16 |
| P25686 | DnaJ homolog subfamily B member 2 | DNAJB2 | -1.31 | 4.22 | 0.18 | 1.54 | 0.68 | 0.50 | 0.13 | -0.82 | 0.15 | -2.25 | 0.17 | -0.30 |
| O75143 | Autophagy-related protein 13 | ATG13 | -1.31 | 2.32 | 0.32 | 1.68 | -0.94 | 1.20 | -0.15 | -0.67 | -0.62 | -1.11 | 1.04 | -0.74 |
| O60573 | Eukaryotic translation initiation factor 4E type 2 | EIF4E2 | -1.31 | 3.38 | 1.14 | -0.25 | 0.07 | 1.12 | 0.61 | -1.06 | -1.34 | -1.50 | 0.85 | 0.36 |

| | | | | | | | | | | | | | | |
|---|---|---|---|---|---|---|---|---|---|---|---|---|---|---|
| P18583 | Protein SON | SON | -1.32 | 1.46 | 0.50 | 0.55 | -1.57 | 0.86 | 1.20 | -0.40 | -1.41 | -0.92 | 0.85 | 0.34 |
| Q8TD47 | 40S ribosomal protein S4, Y isoform 2 | RPS4Y2 | -1.32 | 2.92 | -0.17 | -0.42 | 2.31 | 1.32 | -0.43 | -0.44 | -0.42 | -0.85 | -0.44 | -0.46 |
| A1X283 | SH3 and PX domain-containing protein 2B | SH3PXD2B | -1.32 | 1.55 | 0.40 | 0.98 | 1.12 | 0.35 | -1.24 | -1.07 | 1.20 | -1.46 | 0.21 | -0.48 |
| Q8N4X5 | Actin filament-associated protein 1-like 2 | AFAP1L2 | -1.32 | 3.74 | 0.89 | 0.35 | -0.37 | 1.06 | 0.92 | 0.25 | 0.30 | -1.93 | -0.02 | -1.45 |
| Q9NRU3 | Metal transporter CNNM1 | CNNM1 | -1.32 | 2.67 | 0.43 | 0.76 | -0.30 | 0.88 | 0.62 | 0.55 | -0.40 | -1.58 | 0.86 | -1.81 |
| Q8IV38 | Ankyrin repeat and MYND domain-containing protein 2 | ANKMY2 | -1.32 | 2.09 | -0.52 | 0.30 | 0.31 | 0.40 | 1.51 | -1.54 | -1.76 | 0.48 | 0.61 | 0.21 |
| Q5T6F2 | Ubiquitin-associated protein 2 | UBAP2 | -1.32 | 2.75 | 0.72 | 1.66 | 0.04 | 0.79 | -0.88 | 0.95 | -0.14 | -1.33 | -0.94 | -0.88 |
| O95405 | Zinc finger FYVE domain-containing protein 9 | ZFYVE9 | -1.32 | 1.21 | 1.26 | 0.10 | -1.04 | 0.42 | 0.59 | 1.43 | -0.98 | -1.20 | 0.47 | -1.06 |
| Q15418 | Ribosomal protein S6 kinase alpha-1 | RPS6KA1 | -1.32 | 1.42 | 1.23 | 0.01 | -0.24 | 0.52 | 0.01 | 1.01 | -1.53 | -1.82 | 0.11 | 0.70 |
| O60684 | Importin subunit alpha-7 | KPNA6 | -1.33 | 2.30 | -0.81 | 0.02 | 0.04 | 0.42 | 2.51 | -0.93 | -0.72 | -0.39 | 0.33 | -0.46 |
| Q9BW91 | ADP-ribose pyrophosphatase, mitochondrial | NUDT9 | -1.33 | 2.40 | -0.05 | 1.07 | -0.17 | 0.06 | 1.27 | 1.07 | -0.04 | -1.64 | -1.54 | -0.04 |
| P01871 | Immunoglobulin heavy constant mu | IGHM | -1.33 | 1.22 | -0.12 | 1.06 | 0.33 | 0.64 | -0.55 | -0.61 | -2.36 | 0.19 | 0.57 | 0.85 |
| O43759 | Synaptogyrin-1 | SYNGR1 | -1.33 | 1.76 | 0.94 | 1.83 | -1.08 | 0.05 | 0.01 | -1.45 | 0.10 | -1.10 | 0.46 | 0.24 |
| Q9Y4E6 | WD repeat-containing protein 7 | WDR7 | -1.33 | 3.03 | 0.51 | 1.71 | -0.30 | 0.36 | 0.22 | -0.43 | 0.30 | -2.21 | 0.36 | -0.51 |
| Q8N6T3 | ADP-ribosylation factor GTPase-activating protein 1 | ARFGAP1 | -1.33 | 2.90 | 0.67 | 1.76 | -0.50 | 0.43 | 0.06 | -0.98 | 0.61 | -1.89 | 0.14 | -0.30 |
| Q05086 | Ubiquitin-protein ligase E3A | UBE3A | -1.33 | 3.49 | 0.34 | 1.62 | -0.31 | 1.22 | -0.18 | -0.67 | -1.55 | -1.21 | 0.52 | 0.22 |
| Q15942 | Zyxin | ZYX | -1.33 | 2.18 | 1.12 | 1.01 | -0.49 | -0.36 | 0.75 | 0.63 | -0.67 | -2.20 | 0.31 | -0.10 |
| Q9NY12 | H/ACA ribonucleoprotein complex subunit 1 | GAR1 | -1.33 | 1.69 | 1.08 | 1.13 | 0.71 | -1.32 | 0.10 | -0.07 | -1.33 | 0.31 | -1.37 | 0.75 |
| Q9UHR5 | SAP30-binding protein | SAP30BP | -1.34 | 2.29 | 2.12 | -0.43 | -0.67 | -0.41 | 1.62 | -0.41 | -0.37 | -0.73 | -0.31 | -0.41 |
| P35658 | Nuclear pore complex protein Nup214 | NUP214 | -1.34 | 2.61 | -0.18 | -0.44 | -0.66 | 2.09 | 1.60 | -0.36 | -0.34 | -0.90 | -0.49 | -0.33 |
| P53680 | AP-2 complex subunit sigma | AP2S1 | -1.34 | 1.83 | -1.01 | 1.33 | 0.26 | 0.59 | 0.64 | -1.93 | -1.00 | 0.03 | 0.44 | 0.67 |
| Q96IV0 | Peptide-N(4)-(N-acetyl-beta-glucosaminyl)asparagine amidase | NGLY1 | -1.34 | 3.27 | 0.22 | 0.36 | -0.72 | 0.82 | 1.90 | -0.70 | -1.04 | -1.27 | 0.82 | -0.39 |
| P22102 | Trifunctional purine biosynthetic protein adenosine-3 | GART | -1.34 | 1.72 | -0.65 | 0.74 | 0.16 | 0.80 | 0.71 | -0.32 | -2.49 | 0.52 | 0.02 | 0.51 |
| Q8N806 | Putative E3 ubiquitin-protein ligase UBR7 | UBR7 | -1.34 | 2.22 | 0.54 | 0.29 | 1.09 | -0.11 | 0.33 | -0.25 | -1.23 | -1.98 | -0.02 | 1.35 |
| Q9Y2X9 | Zinc finger protein 281 | ZNF281 | -1.34 | 3.40 | -0.41 | 1.02 | -0.88 | 1.31 | 1.74 | -0.71 | -0.69 | -1.02 | 0.13 | -0.49 |
| O60503 | Adenylate cyclase type 9 | ADCY9 | -1.35 | 3.72 | 1.79 | 1.62 | -0.65 | -0.23 | 0.37 | -0.85 | -1.01 | -0.79 | -0.43 | 0.17 |
| A8MVW0 | Protein FAM171A2 | FAM171A2 | -1.35 | 1.67 | 0.39 | 1.66 | -1.38 | 0.19 | 0.82 | -1.13 | 0.60 | -0.78 | 0.57 | -0.95 |
| Q7Z589 | BRCA2-interacting transcriptional repressor EMSY | EMSY | -1.35 | 2.88 | -0.49 | -0.71 | 1.40 | 0.93 | 1.28 | -0.80 | -0.82 | -1.03 | -0.71 | 0.95 |
| P14406 | Cytochrome c oxidase subunit 7A2, mitochondrial | COX7A2 | -1.35 | 1.24 | -0.75 | 1.32 | -1.08 | 0.90 | 0.96 | -0.84 | -0.89 | -1.08 | 0.88 | 0.58 |
| Q08AE8 | Protein spire homolog 1 | SPIRE1 | -1.35 | 1.87 | 0.56 | 0.78 | -0.39 | 0.60 | 0.34 | -0.18 | 0.47 | -2.64 | 0.50 | -0.05 |
| Q8WUH2 | Transforming growth factor-beta receptor-associated protein 1 | TGFBRAP1 | -1.35 | 3.49 | 1.01 | 1.07 | -0.91 | 0.65 | 0.85 | -0.35 | -2.11 | -0.32 | 0.35 | -0.26 |
| Q9Y4W2 | Ribosomal biogenesis protein LAS1L | LAS1L | -1.35 | 1.72 | 1.42 | -0.79 | -0.88 | 1.46 | 0.51 | -0.72 | -0.75 | -1.02 | 1.00 | -0.23 |
| Q6UB98 | Ankyrin repeat domain-containing protein 12 | ANKRD12 | -1.35 | 1.02 | -0.07 | -0.71 | -0.77 | 0.91 | 1.80 | -0.87 | -0.88 | -0.92 | 0.35 | 1.15 |
| Q9H4B7 | Tubulin beta-1 chain | TUBB1 | -1.36 | 2.04 | -0.76 | 1.52 | 1.27 | 0.79 | -0.87 | -0.95 | -0.97 | 0.24 | 0.63 | -0.91 |
| Q9BY89 | Uncharacterized protein KIAA1671 | KIAA1671 | -1.36 | 2.05 | -1.06 | 1.49 | -0.45 | 0.81 | 1.16 | -1.26 | -1.13 | -0.44 | 0.49 | 0.39 |
| Q92526 | T-complex protein 1 subunit zeta-2 | CCT6B | -1.36 | 0.91 | -1.37 | 0.67 | 0.72 | 0.64 | 0.41 | -1.44 | -1.52 | 0.61 | 0.70 | 0.58 |
| Q96G61 | Diphosphoinositol polyphosphate phosphohydrolase 3-beta | NUDT11 | -1.36 | 1.92 | -0.34 | 0.86 | 0.05 | 0.86 | 0.49 | -0.26 | -0.67 | -2.36 | 0.94 | 0.44 |
| Q8WXS3 | Brain and acute leukemia cytoplasmic protein | BAALC | -1.36 | 2.15 | 1.20 | 1.83 | -0.87 | 0.64 | -0.77 | -0.64 | -0.58 | -0.90 | 0.72 | -0.62 |
| Q9HB21 | Pleckstrin homology domain-containing family A member 1 | PLEKHA1 | -1.36 | 2.95 | 1.10 | 0.67 | -1.06 | 0.99 | 0.73 | 0.33 | -0.61 | -1.73 | 0.54 | -0.96 |
| Q03252 | Lamin-B2 | LMNB2 | -1.37 | 2.69 | -0.68 | 0.58 | 0.40 | 0.68 | 1.34 | -0.77 | -0.87 | -1.26 | -0.87 | 1.44 |
| Q92785 | Zinc finger protein ubi-d4 | DPF2 | -1.37 | 3.33 | 1.26 | 1.52 | -1.06 | 0.46 | 0.45 | -0.37 | -0.06 | -1.75 | 0.15 | -0.59 |
| P04179 | Superoxide dismutase [Mn], mitochondrial | SOD2 | -1.37 | 1.84 | -0.11 | 0.46 | 0.40 | 0.56 | 0.59 | -0.94 | -2.36 | 1.20 | 0.22 | -0.02 |
| P16112 | Aggrecan core protein | ACAN | -1.37 | 1.61 | -0.38 | -0.22 | 1.54 | 0.16 | 0.57 | -0.60 | -1.96 | 1.32 | -0.04 | -0.38 |
| Q02156 | Protein kinase C epsilon type | PRKCE | -1.37 | 2.41 | 1.22 | 1.21 | -0.74 | 0.58 | -0.11 | -0.37 | 0.13 | -2.16 | 0.52 | -0.27 |
| P08779 | Keratin, type I cytoskeletal 16 | KRT16 | -1.37 | 1.17 | -0.57 | 0.07 | 0.77 | 0.58 | 0.49 | -2.40 | -0.70 | 0.91 | 0.64 | 0.23 |
| Q9HD26 | Golgi-associated PDZ and coiled-coil motif-containing protein | GOPC | -1.37 | 2.56 | 0.57 | 0.73 | 0.25 | 0.80 | 0.02 | 0.61 | -1.28 | -2.29 | 0.37 | 0.21 |
| Q9Y6K8 | Adenylate kinase isoenzyme 5 | AK5 | -1.37 | 3.26 | 1.16 | 0.89 | -0.23 | 1.02 | -0.24 | -1.35 | 0.12 | -1.83 | 0.68 | -0.23 |
| Q9BZE9 | Tether containing UBX domain for GLUT4 | ASPSCR1 | -1.37 | 2.42 | 0.57 | 1.08 | 0.19 | 0.46 | -0.03 | -0.11 | -1.24 | -1.64 | 1.54 | -0.81 |
| Q86UU1 | Pleckstrin homology-like domain family B member 1 | PHLDB1 | -1.38 | 1.96 | -0.61 | -0.73 | 0.77 | 0.53 | 1.92 | -0.78 | -0.80 | -1.18 | 0.93 | -0.06 |
| Q9P0P8 | Mitochondrial transcription rescue factor 1 | MTRES1 | -1.38 | 2.42 | 0.69 | 0.47 | 1.88 | 0.15 | -1.02 | -0.92 | -0.81 | -1.33 | 0.43 | 0.47 |
| O75348 | V-type proton ATPase subunit G 1 | ATP6V1G1 | -1.38 | 2.07 | 0.07 | 0.64 | 1.33 | 0.19 | -0.24 | -0.88 | 0.03 | -2.29 | 0.39 | 0.75 |
| Q9BZF1 | Oxysterol-binding protein-related protein 8 | OSBPL8 | -1.38 | 1.74 | 0.53 | 0.25 | 0.98 | 0.66 | -0.64 | 0.48 | -0.01 | -2.51 | 0.51 | -0.25 |
| P56211 | cAMP-regulated phosphoprotein 19 | ARPP19 | -1.38 | 3.71 | 0.24 | 1.68 | 0.21 | -0.11 | 0.78 | -2.24 | -0.50 | -0.34 | 0.02 | 0.27 |
| Q15637 | Splicing factor 1 | SF1 | -1.39 | 4.87 | 0.38 | 1.06 | 0.61 | 0.90 | 0.48 | -2.17 | -0.07 | -1.25 | 0.17 | -0.12 |
| Q5JQF8 | Polyadenylate-binding protein 1-like 2 | PABPC1L2A | -1.39 | 2.61 | 0.84 | 0.30 | -0.66 | 1.05 | 0.78 | 0.13 | -1.31 | -1.98 | 0.59 | 0.28 |
| Q9NPF8 | Arf-GAP with dual PH domain-containing protein 2 | ADAP2 | -1.39 | 1.62 | -0.10 | 2.83 | -0.43 | -0.22 | -0.33 | -0.29 | -0.30 | -0.50 | -0.36 | -0.29 |
| A0A1B0GTU1 | Zinc finger CCCH domain-containing protein 11B | ZC3H11B | -1.39 | 1.93 | 1.51 | 0.89 | -1.42 | 0.31 | 0.57 | 0.56 | -1.16 | -0.45 | 0.39 | -1.21 |
| O60331 | Phosphatidylinositol 4-phosphate 5-kinase type-1 gamma | PIP5K1C | -1.39 | 4.48 | -0.01 | 0.82 | 1.78 | 0.55 | -0.08 | -1.38 | -0.07 | -0.71 | 0.56 | -1.46 |
| P35626 | Beta-adrenergic receptor kinase 2 | GRK3 | -1.39 | 3.03 | -0.32 | -0.33 | 1.48 | -0.36 | 2.20 | -0.49 | -0.31 | -0.90 | -0.51 | -0.45 |
| Q9NVN3 | Synembryn-B | RIC8B | -1.39 | 1.03 | 0.26 | 1.75 | -0.87 | 0.89 | -0.85 | -0.74 | 0.04 | -0.94 | -0.79 | 1.24 |
| P29074 | Tyrosine-protein phosphatase non-receptor type 4 | PTPN4 | -1.40 | 3.60 | 1.55 | -0.44 | -0.87 | 1.52 | 1.21 | -0.55 | -0.53 | -0.93 | -0.49 | -0.47 |
| Q8N490 | Probable hydrolase PNKD | PNKD | -1.40 | 2.24 | -0.12 | 2.55 | -0.64 | 0.90 | -0.48 | -0.41 | -0.45 | -0.66 | -0.36 | -0.30 |
| P46939 | Utrophin | UTRN | -1.40 | 3.19 | -0.74 | 2.20 | 0.05 | 0.60 | 0.49 | -0.33 | -1.35 | -1.02 | 0.11 | -0.02 |
| P61421 | V-type proton ATPase subunit d 1 | ATP6V0D1 | -1.40 | 6.48 | 0.95 | 1.21 | 1.09 | 0.42 | -0.01 | -0.82 | 0.31 | -1.87 | -0.35 | -0.94 |
| Q9Y3A4 | Ribosomal RNA-processing protein 7 homolog A | RRP7A | -1.40 | 2.13 | -0.71 | 1.12 | 0.14 | -0.48 | 1.93 | -0.82 | -0.78 | 0.99 | -0.59 | -0.80 |
| Q8TE49 | OTU domain-containing protein 7A | OTUD7A | -1.40 | 1.05 | 0.85 | 0.79 | 1.17 | -0.90 | -0.72 | -0.98 | 0.86 | -1.15 | 1.03 | -0.95 |
| Q6ZV89 | SH2 domain-containing protein 5 | SH2D5 | -1.41 | 2.03 | -0.21 | 0.98 | -0.09 | 0.96 | 0.34 | -2.24 | 0.40 | -1.06 | 0.80 | 0.12 |
| Q05516 | Zinc finger and BTB domain-containing protein 16 | ZBTB16 | -1.41 | 2.56 | 0.34 | 0.89 | -1.38 | 1.02 | 1.38 | -0.59 | 0.11 | -1.30 | 0.49 | -0.97 |
| Q8IWT3 | Cullin-9 | CUL9 | -1.41 | 2.07 | 0.27 | -0.04 | -0.98 | 1.05 | 1.64 | -0.84 | -0.98 | -1.16 | 1.07 | -0.05 |
| Q9H2P0 | Activity-dependent neuroprotector homeobox protein | ADNP | -1.42 | 2.13 | -0.38 | 0.48 | 0.65 | 0.62 | 0.70 | -0.60 | -2.53 | 0.47 | 0.58 | 0.01 |
| P16144 | Integrin beta-4 | ITGB4 | -1.42 | 0.66 | -1.01 | 0.43 | 0.40 | 0.84 | 0.17 | -1.40 | -1.76 | 0.68 | 0.87 | 0.78 |
| Q13075 | Baculoviral IAP repeat-containing protein 1 | NAIP | -1.42 | 2.75 | 0.43 | 1.07 | -0.77 | -0.53 | 2.32 | -0.39 | -0.40 | -0.80 | -0.53 | -0.40 |
| Q96DZ9 | CKLF-like MARVEL transmembrane domain-containing protein 5 | CMTM5 | -1.42 | 2.82 | -0.35 | 1.84 | 1.92 | -0.39 | -0.46 | -0.40 | -0.47 | -0.83 | -0.41 | -0.44 |
| Q9BV73 | Centrosome-associated protein CEP250 | CEP250 | -1.42 | 1.92 | 2.48 | -0.44 | 0.85 | -0.47 | -0.49 | -0.50 | -0.46 | -0.84 | -0.44 | 0.31 |
| P47224 | Guanine nucleotide exchange factor MSS4 | RABIF | -1.42 | 2.32 | 1.63 | 2.11 | -0.62 | -0.44 | -0.43 | -0.39 | -0.30 | -0.74 | -0.45 | -0.38 |
| P35613 | Basigin | BSG | -1.42 | 1.64 | -0.15 | -0.29 | 2.83 | -0.31 | -0.32 | -0.34 | -0.23 | -0.54 | -0.37 | -0.28 |
| Q6ZMT1 | SH3 and cysteine-rich domain-containing protein 2 | STAC2 | -1.42 | 3.29 | 1.29 | 1.72 | -0.80 | 1.06 | -0.56 | 0.22 | -0.67 | -1.01 | -0.65 | -0.60 |
| O15240 | Neurosecretory protein VGF | VGF | -1.42 | 1.55 | 1.17 | 1.16 | -1.18 | -0.47 | 0.91 | 0.96 | 0.00 | -1.33 | -1.06 | -0.18 |
| Q17R89 | Rho GTPase-activating protein 44 | ARHGAP44 | -1.42 | 4.42 | 0.82 | 1.62 | 0.06 | 0.71 | -0.17 | -0.25 | -1.60 | -1.48 | 0.52 | -0.24 |
| P56377 | AP-1 complex subunit sigma-2 | AP1S2 | -1.42 | 1.78 | 1.53 | 2.01 | -0.66 | -0.45 | -0.61 | 0.48 | -0.55 | -0.76 | -0.50 | -0.49 |
| Q9UQ13 | Leucine-rich repeat protein SHOC-2 | SHOC2 | -1.43 | 2.02 | 0.43 | 0.10 | 1.21 | -1.07 | 1.24 | -1.16 | -1.05 | 0.89 | 0.49 | -1.09 |
| P78352 | Disks large homolog 4 | DLG4 | -1.43 | 2.35 | -0.16 | 0.84 | 1.50 | -0.23 | 0.18 | -1.34 | 1.18 | -1.63 | -0.26 | -0.09 |
| Q9UPW8 | Protein unc-13 homolog A | UNC13A | -1.43 | 1.00 | -0.27 | 0.36 | 1.14 | -0.67 | 0.61 | 0.28 | -1.85 | 1.34 | -1.15 | 0.21 |

| | | | | | | | | | | | | | | |
|---|---|---|---|---|---|---|---|---|---|---|---|---|---|---|
| Q96B45 | BLOC-1-related complex subunit 7 | BORCS7 | -1.43 | 3.35 | -0.42 | 0.52 | 0.73 | -0.44 | 2.46 | -0.53 | -0.58 | -0.89 | -0.48 | -0.37 |
| P43250 | G protein-coupled receptor kinase 6 | GRK6 | -1.43 | 2.14 | -1.26 | 1.69 | 0.03 | 0.62 | 0.93 | -0.09 | 0.48 | -1.22 | 0.07 | -1.25 |
| Q8TAB3 | Protocadherin-19 | PCDH19 | -1.43 | 3.39 | 1.33 | 1.69 | 0.75 | -0.16 | -0.91 | -0.81 | 0.20 | -1.24 | -0.02 | -0.83 |
| P50454 | Serpin H1 | SERPINH1 | -1.43 | 1.60 | -0.23 | -0.28 | 2.83 | -0.30 | -0.29 | -0.23 | -0.32 | -0.55 | -0.33 | -0.30 |
| Q13972 | Ras-specific guanine nucleotide-releasing factor 1 | RASGRF1 | -1.43 | 2.11 | 1.45 | -0.16 | -0.99 | 0.99 | 0.69 | -0.32 | -0.08 | -2.00 | 0.55 | -0.12 |
| Q9BZI7 | Regulator of nonsense transcripts 3B | UPF3B | -1.44 | 2.05 | 0.73 | 2.28 | -0.48 | -0.77 | 0.22 | 0.02 | -0.18 | -0.51 | -1.44 | 0.14 |
| Q9NRD1 | F-box only protein 6 | FBXO6 | -1.44 | 3.86 | 0.85 | 0.15 | 1.23 | 0.85 | -0.17 | -1.69 | -1.11 | -1.16 | 0.44 | 0.61 |
| Q12923 | Tyrosine-protein phosphatase non-receptor type 13 | PTPN13 | -1.44 | 1.56 | -0.86 | 0.89 | 1.26 | 0.56 | -0.25 | -0.06 | -2.06 | -0.71 | 0.63 | 0.59 |
| P35232 | Prohibitin | PHB | -1.44 | 2.50 | 0.50 | 0.88 | 0.23 | 0.94 | -0.25 | -0.52 | -2.36 | -0.60 | 0.80 | 0.38 |
| Q9UQC2 | GRB2-associated-binding protein 2 | GAB2 | -1.44 | 1.92 | 0.82 | 1.25 | -1.30 | 0.60 | 0.49 | 0.47 | -0.70 | -1.48 | 0.82 | -0.95 |
| O75151 | Lysine-specific demethylase PHF2 | PHF2 | -1.45 | 4.87 | 0.49 | 0.66 | 1.37 | 0.40 | 0.42 | 0.07 | -0.68 | -2.28 | 0.16 | -0.62 |
| Q53HC5 | Kelch-like protein 26 | KLHL26 | -1.45 | 2.12 | -0.58 | -0.06 | 2.22 | 1.19 | -0.73 | -0.57 | -0.68 | -0.97 | 0.20 | -0.04 |
| Q9UPY6 | Wiskott-Aldrich syndrome protein family member 3 | WASF3 | -1.45 | 3.10 | 1.99 | -0.01 | 0.59 | 0.23 | -0.29 | 0.64 | 0.19 | -1.06 | -1.58 | -0.69 |
| Q12805 | EGF-containing fibulin-like extracellular matrix protein 1 | EFEMP1 | -1.45 | 1.58 | -0.73 | 0.65 | 0.65 | -0.03 | 1.10 | -0.36 | -2.34 | 0.85 | 0.24 | -0.03 |
| Q86TP1 | Exopolyphosphatase PRUNE1 | PRUNE1 | -1.46 | 1.97 | -0.05 | 0.00 | 2.78 | -0.12 | -0.59 | -0.26 | -0.62 | -0.46 | -0.36 | -0.32 |
| Q969G6 | Riboflavin kinase | RFK | -1.46 | 2.12 | -0.18 | 0.62 | 0.35 | 0.17 | 1.11 | -0.68 | -2.43 | 0.88 | 0.23 | -0.05 |
| Q9Y6R1 | Electrogenic sodium bicarbonate cotransporter 1 | SLC4A4 | -1.46 | 2.12 | -1.08 | 0.55 | 1.79 | 0.01 | 0.71 | -1.16 | -1.10 | -0.43 | -0.29 | 1.00 |
| O75970 | Multiple PDZ domain protein | MPDZ | -1.46 | 3.59 | 1.16 | 0.63 | -0.33 | 1.13 | 0.18 | -0.26 | 0.36 | -2.23 | 0.16 | -0.80 |
| Q14151 | Scaffold attachment factor B2 | SAFB2 | -1.46 | 1.84 | 0.78 | 0.78 | -0.96 | 0.70 | 0.53 | -1.75 | -1.41 | 0.97 | 0.03 | 0.35 |
| Q13873 | Bone morphogenetic protein receptor type-2 | BMPR2 | -1.47 | 2.78 | -0.34 | 2.12 | 1.41 | -0.96 | 0.31 | -0.45 | -0.50 | -0.75 | -0.51 | -0.34 |
| O14640 | Segment polarity protein dishevelled homolog DVL-1 | DVL1 | -1.47 | 2.38 | 0.05 | 0.88 | -1.14 | 1.06 | 1.29 | -0.98 | -0.80 | -1.27 | 0.95 | -0.04 |
| O15230 | Laminin subunit alpha-5 | LAMA5 | -1.47 | 1.92 | -0.95 | -0.28 | 1.47 | 0.35 | 1.26 | -1.09 | -1.09 | 0.88 | 0.37 | -0.93 |
| Q9BV68 | E3 ubiquitin-protein ligase RNF126 | RNF126 | -1.47 | 2.75 | 1.14 | 1.29 | -1.07 | -0.09 | 1.08 | -0.87 | -0.81 | 0.98 | -0.78 | -0.86 |
| Q9NRX5 | Serine incorporator 1 | SERINC1 | -1.47 | 2.50 | 2.20 | 1.53 | -0.55 | -0.34 | -0.46 | -0.36 | -0.41 | -0.71 | -0.47 | -0.42 |
| Q5JU85 | IQ motif and SEC7 domain-containing protein 2 | IQSEC2 | -1.47 | 4.15 | 0.46 | 1.34 | -0.34 | 0.85 | 0.68 | -1.78 | -0.62 | -1.35 | 0.47 | 0.29 |
| Q9Y2G0 | Protein EFR3 homolog B | EFR3B | -1.47 | 2.40 | 0.68 | 1.29 | -0.28 | 0.34 | 0.17 | -0.76 | 0.60 | -2.37 | 0.30 | 0.03 |
| P80723 | Brain acid soluble protein 1 | BASP1 | -1.47 | 2.90 | 0.12 | 1.85 | -0.29 | -0.42 | 1.16 | -1.83 | 0.48 | -0.29 | -0.42 | -0.35 |
| A6XGL0 | YjeF N-terminal domain-containing protein 3 | YJEFN3 | -1.48 | 1.94 | -0.20 | 0.95 | 0.57 | 0.78 | -0.19 | -1.70 | -1.66 | -0.23 | 0.87 | 0.79 |
| Q9UBM7 | 7-dehydrocholesterol reductase | DHCR7 | -1.48 | 2.06 | -0.52 | 0.98 | -0.81 | 0.35 | 1.96 | -0.67 | -0.61 | -0.99 | -0.65 | 0.96 |
| Q6ZS81 | WD repeat- and FYVE domain-containing protein 4 | WDFY4 | -1.48 | 1.87 | 1.15 | -0.19 | 0.43 | 0.12 | 0.38 | 0.81 | -2.55 | 0.24 | -0.13 | -0.26 |
| O95793 | Double-stranded RNA-binding protein Staufen homolog 1 | STAU1 | -1.48 | 1.76 | 1.31 | 0.40 | 0.74 | -1.17 | 0.48 | 0.61 | -0.38 | -1.47 | -1.29 | 0.78 |
| O43166 | Signal-induced proliferation-associated 1-like protein 1 | SIPA1L1 | -1.49 | 2.68 | -0.93 | 1.75 | 0.51 | 0.81 | 0.15 | -0.85 | -1.14 | -1.24 | 0.73 | 0.20 |
| O75152 | Zinc finger CCCH domain-containing protein 11A | ZC3H11A | -1.49 | 2.24 | -0.60 | -0.14 | 0.38 | 1.14 | 1.29 | -1.92 | -0.57 | -0.82 | 0.79 | 0.47 |
| Q15772 | Striated muscle preferentially expressed protein kinase | SPEG | -1.49 | 2.80 | 2.04 | -0.37 | 1.72 | -0.33 | -0.51 | -0.39 | -0.51 | -0.74 | -0.51 | -0.41 |
| P63215 | Guanine nucleotide-binding protein G(I)/G(S)/G(O) subunit gamma | GNG3 | -1.49 | 1.89 | 1.22 | 1.38 | -0.32 | -1.13 | 0.69 | 0.71 | -0.34 | -1.37 | -1.09 | 0.25 |
| Q86UW8 | Hyaluronan and proteoglycan link protein 4 | HAPLN4 | -1.49 | 2.35 | 0.54 | 0.00 | 0.96 | -0.58 | 1.22 | -0.20 | -2.34 | 0.61 | -0.26 | 0.06 |
| Q9UJF2 | Ras GTPase-activating protein nGAP | RASAL2 | -1.50 | 3.46 | 1.01 | 0.98 | -1.09 | 0.88 | 0.90 | -0.33 | -0.54 | -1.77 | 0.59 | -0.62 |
| Q8NDI1 | EH domain-binding protein 1 | EHBP1 | -1.50 | 2.83 | 0.83 | 0.88 | 0.21 | 0.80 | -0.25 | 0.18 | -0.43 | -2.55 | 0.20 | 0.13 |
| Q7Z7E8 | Ubiquitin-conjugating enzyme E2 Q1 | UBE2Q1 | -1.50 | 1.60 | -0.84 | 1.05 | -0.55 | 1.17 | 0.80 | -1.04 | -0.94 | -1.23 | 1.06 | 0.52 |
| O00408 | cGMP-dependent 3',5'-cyclic phosphodiesterase | PDE2A | -1.50 | 3.28 | 0.38 | 1.89 | 0.31 | 0.18 | -0.16 | -1.40 | 0.49 | -1.71 | 0.12 | -0.10 |
| Q9NRF2 | SH2B adapter protein 1 | SH2B1 | -1.50 | 2.78 | -0.02 | 0.79 | 1.51 | 0.50 | -0.41 | -0.55 | -1.18 | -1.77 | 0.28 | 0.86 |
| Q2M389 | WASH complex subunit 4 | WASHC4 | -1.51 | 2.80 | -1.00 | 1.54 | 0.27 | 0.76 | 0.78 | -1.42 | -1.28 | -0.47 | 0.68 | 0.13 |
| O94885 | SAM and SH3 domain-containing protein 1 | SASH1 | -1.51 | 1.64 | 0.79 | 0.52 | -0.78 | 0.68 | 0.48 | -0.20 | 0.68 | -2.46 | 0.49 | -0.20 |
| P29475 | Nitric oxide synthase, brain | NOS1 | -1.51 | 1.38 | -0.98 | -0.42 | 0.61 | 0.24 | 2.02 | -1.60 | -0.70 | 0.46 | 0.28 | 0.09 |
| O94910 | Adhesion G protein-coupled receptor L1 | ADGRL1 | -1.51 | 2.32 | 1.01 | 1.49 | -0.70 | 0.56 | -0.25 | 0.65 | -0.83 | -1.74 | 0.52 | -0.70 |
| O75155 | Cullin-associated NEDD8-dissociated protein 2 | CAND2 | -1.51 | 1.04 | 0.42 | 2.21 | -1.32 | 0.07 | -0.18 | 0.05 | 0.27 | -1.39 | 0.22 | -0.36 |
| P0DJI8 | Serum amyloid A-1 protein | SAA1 | -1.51 | 1.35 | -0.46 | 1.96 | 1.23 | -0.60 | -0.67 | -0.53 | -0.56 | -0.82 | -0.57 | 1.02 |
| P52824 | Diacylglycerol kinase theta | DGKQ | -1.52 | 1.63 | -0.65 | 0.50 | 0.19 | 0.38 | 1.26 | -1.26 | -2.03 | 0.66 | 0.46 | 0.48 |
| Q9ULI1 | NACHT and WD repeat domain-containing protein 2 | NWD2 | -1.52 | 1.20 | -0.01 | 1.41 | -1.40 | 0.66 | 0.66 | -1.27 | -1.35 | 0.14 | 0.66 | 0.50 |
| Q7Z2W4 | Zinc finger CCCH-type antiviral protein 1 | ZC3HAV1 | -1.52 | 5.29 | 0.39 | 1.23 | -0.19 | 0.21 | 1.65 | -0.23 | -1.60 | -1.36 | 0.09 | -0.19 |
| Q9UPX8 | SH3 and multiple ankyrin repeat domains protein 2 | SHANK2 | -1.53 | 3.73 | 0.71 | 0.87 | 0.83 | 0.35 | 0.23 | -0.71 | -0.11 | -2.41 | 0.64 | -0.40 |
| Q86US8 | Telomerase-binding protein EST1A | SMG6 | -1.53 | 0.98 | -1.38 | 0.51 | 0.59 | 0.85 | 0.57 | -1.42 | -1.47 | 0.19 | 0.74 | 0.83 |
| O75351 | Vacuolar protein sorting-associated protein 4B | VPS4B | -1.53 | 2.36 | 0.27 | 0.56 | 1.38 | 0.49 | -0.54 | 0.19 | 0.00 | -2.38 | 0.51 | -0.47 |
| P58005 | Sestrin-3 | SESN3 | -1.53 | 2.54 | 0.88 | -0.32 | -0.47 | -0.27 | 2.57 | -0.43 | -0.38 | -0.73 | -0.45 | -0.42 |
| O00533 | Neural cell adhesion molecule L1-like protein | CHL1 | -1.53 | 3.39 | -0.89 | 0.69 | 1.85 | 0.05 | 0.97 | -1.24 | -0.91 | 0.61 | -0.57 | -0.57 |
| Q6ZUT9 | DENN domain-containing protein 5B | DENND5B | -1.54 | 5.34 | 0.75 | 2.06 | -0.36 | 0.92 | 0.08 | -0.60 | -1.30 | -0.98 | -0.16 | -0.41 |
| Q9Y4F4 | TOG array regulator of axonemal microtubules protein 1 | TOGARAM1 | -1.54 | 1.47 | -0.20 | -0.33 | -0.42 | -0.27 | 2.84 | -0.28 | -0.33 | -0.47 | -0.30 | -0.24 |
| Q96PV0 | Ras/Rap GTPase-activating protein SynGAP | SYNGAP1 | -1.54 | 1.86 | 0.01 | 0.03 | 0.84 | 0.63 | 0.39 | -1.07 | 1.46 | -2.05 | 0.24 | -0.48 |
| Q9P2G1 | Ankyrin repeat and IBR domain-containing protein 1 | ANKIB1 | -1.54 | 2.78 | 0.60 | 1.33 | -1.17 | 1.08 | 0.51 | -0.98 | -0.90 | -1.32 | 0.73 | 0.12 |
| Q69YW2 | Protein stum homolog | STUM | -1.55 | 1.55 | 0.43 | 1.10 | -1.00 | 0.93 | 0.15 | 0.50 | 0.45 | 0.58 | -1.58 | -1.56 |
| O94905 | Erlin-2 | ERLIN2 | -1.55 | 2.33 | 1.00 | 0.70 | 0.28 | 0.01 | 0.22 | 0.25 | -2.67 | -0.28 | 0.21 | 0.28 |
| P40123 | Adenylyl cyclase-associated protein 2 | CAP2 | -1.55 | 3.98 | 0.25 | 1.81 | -0.16 | 0.67 | 0.32 | -0.75 | -0.52 | -2.00 | 0.48 | -0.10 |
| P29323 | Ephrin type-B receptor 2 | EPHB2 | -1.55 | 3.53 | 0.37 | 0.25 | 0.91 | 0.88 | 0.48 | -1.26 | -1.21 | -1.69 | 0.97 | 0.30 |
| Q9BZ71 | Membrane-associated phosphatidylinositol transfer protein 3 | PITPNM3 | -1.55 | 1.64 | 0.95 | 0.07 | 0.18 | 0.39 | 0.15 | 0.71 | 0.51 | -2.57 | 0.26 | -0.65 |
| Q9Y2U9 | Kelch domain-containing protein 2 | KLHDC2 | -1.55 | 2.59 | 2.65 | -0.35 | -0.53 | 0.20 | 0.45 | -0.35 | -0.44 | -0.81 | -0.41 | -0.40 |
| Q9UD71 | Protein phosphatase 1 regulatory subunit 1B | PPP1R1B | -1.55 | 2.64 | 0.49 | 0.94 | 0.05 | -0.95 | 1.75 | -0.99 | -1.10 | 0.86 | -1.00 | -0.05 |
| Q96DG6 | Carboxymethylenebutenolidase homolog | CMBL | -1.55 | 1.87 | -1.14 | 0.52 | 0.38 | 0.83 | 1.23 | -1.23 | -1.61 | -0.30 | 0.66 | 0.66 |
| Q6ZVL6 | UPF0606 protein KIAA1549L | KIAA1549L | -1.55 | 3.35 | 0.22 | 1.17 | -0.01 | 0.40 | 0.94 | -2.15 | 0.47 | -0.04 | 0.27 | -1.27 |
| Q562R1 | Beta-actin-like protein 2 | ACTBL2 | -1.56 | 1.16 | 0.17 | -0.84 | 0.19 | 1.17 | 0.62 | -2.09 | -0.37 | -0.53 | 1.24 | 0.45 |
| Q14669 | E3 ubiquitin-protein ligase TRIP12 | TRIP12 | -1.56 | 1.66 | -0.25 | 0.45 | -0.05 | 0.31 | 1.26 | -1.73 | -1.72 | 0.79 | 0.28 | 0.65 |
| Q9NZQ8 | Transient receptor potential cation channel subfamily M member | TRPM5 | -1.56 | 2.18 | -1.22 | 0.90 | 1.06 | 0.94 | 0.34 | -1.36 | -1.42 | 0.83 | 0.08 | -0.14 |
| Q96N16 | Janus kinase and microtubule-interacting protein 1 | JAKMIP1 | -1.56 | 1.93 | -0.14 | 1.38 | -1.13 | 1.09 | 0.67 | -0.92 | -0.92 | -1.27 | 0.83 | 0.41 |
| Q8N6N2 | Tetratricopeptide repeat protein 9B | TTC9B | -1.56 | 3.33 | 0.79 | 0.85 | -1.10 | 0.80 | 1.28 | -0.89 | 0.01 | -0.57 | 0.52 | -1.67 |
| Q9NZR2 | Low-density lipoprotein receptor-related protein 1B | LRP1B | -1.57 | 3.16 | -0.12 | 2.04 | -0.17 | 1.18 | -0.37 | -0.25 | 0.10 | -1.69 | -0.19 | -0.54 |
| Q86VY4 | Testis-specific Y-encoded-like protein 5 | TSPYL5 | -1.57 | 2.76 | 1.76 | -0.70 | -0.83 | 0.53 | 1.76 | -0.57 | -0.65 | -0.47 | -0.57 | -0.27 |
| O60716 | Catenin delta-1 | CTNND1 | -1.57 | 1.40 | -0.82 | 0.94 | 0.11 | 0.17 | 1.10 | -1.44 | 0.65 | -1.70 | 0.11 | 0.88 |
| Q9BZ29 | Dedicator of cytokinesis protein 9 | DOCK9 | -1.57 | 2.19 | -0.83 | 1.18 | 0.24 | 0.78 | 0.66 | -1.98 | -0.91 | -0.44 | 0.79 | 0.50 |
| Q9NR82 | Potassium voltage-gated channel subfamily KQT member 5 | KCNQ5 | -1.58 | 3.90 | 0.20 | 1.64 | -0.99 | 0.75 | 1.32 | -0.68 | -0.62 | -1.17 | 0.36 | -0.81 |
| O15164 | Transcription intermediary factor 1-alpha | TRIM24 | -1.58 | 3.27 | 2.11 | -0.45 | -0.83 | 0.99 | 0.99 | -0.48 | -0.41 | -0.88 | -0.58 | -0.45 |
| Q12816 | Trophinin | TRO | -1.58 | 2.79 | 2.11 | -0.49 | -0.58 | -0.10 | 1.59 | -0.44 | -0.50 | -0.82 | -0.39 | -0.39 |
| Q6ZNB6 | NF-X1-type zinc finger protein NFXL1 | NFXL1 | -1.58 | 3.52 | 0.37 | 0.87 | -1.14 | 1.42 | 1.21 | -0.87 | -0.84 | -1.33 | -0.04 | 0.35 |
| Q13435 | Splicing factor 3B subunit 2 | SF3B2 | -1.58 | 2.12 | 0.51 | -0.12 | 0.79 | -0.15 | 1.02 | -0.64 | -2.26 | 0.47 | -0.58 | 0.97 |
| Q9P2Y5 | UV radiation resistance-associated gene protein | UVRAG | -1.59 | 2.83 | 0.76 | 0.83 | -0.62 | 0.93 | 0.52 | -0.48 | 0.00 | -2.34 | 0.66 | -0.27 |

| | | | | | | | | | | | | | | |
|---|---|---|---|---|---|---|---|---|---|---|---|---|---|---|
| Q7Z6G8 | Ankyrin repeat and sterile alpha motif domain-containing protein | ANKS1B | -1.59 | 3.32 | 0.73 | 0.22 | 0.30 | -0.43 | 1.79 | -1.95 | 0.35 | -0.66 | 0.34 | -0.69 |
| O43761 | Synaptogyrin-3 | SYNGR3 | -1.59 | 1.88 | 0.91 | 1.71 | -1.25 | 0.50 | -0.04 | -0.58 | 0.65 | -1.53 | 0.18 | -0.55 |
| A6NKN8 | Purkinje cell protein 4-like protein 1 | PCP4L1 | -1.59 | 4.43 | -0.34 | 0.75 | 1.76 | -0.48 | 1.58 | -0.68 | -0.23 | -1.02 | -0.69 | -0.67 |
| P17029 | Zinc finger protein with KRAB and SCAN domains 1 | ZKSCAN1 | -1.59 | 1.26 | -0.75 | 0.37 | -0.47 | 1.04 | 1.19 | -1.84 | -0.41 | -0.76 | 1.07 | 0.57 |
| Q8WVD5 | RING finger protein 141 | RNF141 | -1.59 | 2.74 | -0.28 | 1.56 | -0.37 | 0.92 | 0.51 | -1.49 | -1.47 | -0.49 | 0.64 | 0.48 |
| O94805 | Actin-like protein 6B | ACTL6B | -1.59 | 1.33 | 1.01 | -1.49 | 0.26 | 0.98 | 0.67 | 0.81 | -0.28 | -1.36 | 0.56 | -1.16 |
| Q9Y6D6 | Brefeldin A-inhibited guanine nucleotide-exchange protein 1 | ARFGEF1 | -1.60 | 2.92 | 0.53 | 1.31 | -0.25 | -0.07 | 0.95 | 0.09 | 0.35 | -2.40 | -0.31 | -0.19 |
| O15357 | Phosphatidylinositol 3,4,5-trisphosphate 5-phosphatase 2 | INPPL1 | -1.60 | 2.03 | -0.83 | 0.33 | 1.85 | -0.16 | 0.75 | -0.10 | -0.66 | -1.82 | -0.01 | 0.67 |
| Q9HCH5 | Synaptotagmin-like protein 2 | SYTL2 | -1.60 | 3.61 | -0.67 | 0.83 | 0.66 | 0.79 | 1.14 | -1.93 | -1.05 | 0.07 | 0.64 | -0.48 |
| Q13671 | Ras and Rab interactor 1 | RIN1 | -1.60 | 2.32 | 0.60 | 1.07 | -1.15 | 1.03 | 0.55 | -0.81 | -0.28 | -1.86 | 0.79 | 0.06 |
| Q9Y6W5 | Wiskott-Aldrich syndrome protein family member 2 | WASF2 | -1.60 | 4.11 | -0.33 | 1.48 | 1.58 | 1.19 | -0.74 | -0.52 | -0.58 | -1.03 | -0.56 | -0.49 |
| Q01804 | OTU domain-containing protein 4 | OTUD4 | -1.61 | 7.31 | 1.12 | 0.73 | 1.22 | 0.68 | 0.11 | -1.65 | -0.66 | -1.31 | 0.28 | -0.51 |
| Q8N111 | Cell cycle exit and neuronal differentiation protein 1 | CEND1 | -1.61 | 4.16 | -0.23 | 0.06 | 0.98 | -0.02 | 2.34 | -0.38 | -0.50 | -1.02 | -0.23 | -1.00 |
| Q16629 | Serine/arginine-rich splicing factor 7 | SRSF7 | -1.61 | 1.68 | -0.09 | 0.49 | 0.28 | 0.08 | 1.01 | -2.65 | -0.24 | 0.63 | 0.06 | 0.43 |
| Q9NTX7 | E3 ubiquitin-protein ligase RNF146 | RNF146 | -1.61 | 2.37 | 0.94 | 1.13 | -1.26 | 0.27 | 1.06 | 0.06 | -0.22 | -1.46 | -1.19 | 0.67 |
| Q05519 | Serine/arginine-rich splicing factor 11 | SRSF11 | -1.61 | 2.93 | -0.15 | -0.35 | 0.33 | 0.43 | 2.17 | -1.58 | 0.14 | -1.08 | 0.33 | -0.24 |
| Q9NYI0 | PH and SEC7 domain-containing protein 3 | PSD3 | -1.61 | 3.49 | 0.69 | 1.21 | -0.34 | 0.83 | 0.36 | -0.68 | 0.50 | -2.22 | 0.28 | -0.63 |
| Q14088 | Ras-related protein Rab-33A | RAB33A | -1.61 | 2.90 | 0.44 | 0.95 | 0.08 | 0.55 | 0.55 | -0.34 | 0.11 | -2.64 | 0.44 | -0.15 |
| O95359 | Transforming acidic coiled-coil-containing protein 2 | TACC2 | -1.62 | 1.16 | 1.20 | -0.90 | 0.68 | -0.92 | 1.23 | 0.16 | -0.92 | 1.22 | -0.89 | -0.87 |
| Q12926 | ELAV-like protein 2 | ELAVL2 | -1.62 | 2.65 | 0.88 | 0.41 | -0.45 | 0.34 | 1.16 | -1.70 | -0.91 | 1.21 | 0.16 | -1.09 |
| Q9UI09 | NADH dehydrogenase [ubiquinone] 1 alpha subcomplex subunit 12 | NDUFA12 | -1.62 | 3.73 | -0.49 | 1.41 | 1.33 | 0.65 | -0.12 | -1.45 | -1.36 | -0.47 | -0.13 | 0.63 |
| Q14444 | Caprin-1 | CAPRIN1 | -1.63 | 3.27 | 1.30 | -0.10 | -0.87 | 1.05 | 1.21 | -0.81 | -0.43 | -1.50 | 0.74 | -0.59 |
| P25098 | Beta-adrenergic receptor kinase 1 | GRK2 | -1.63 | 2.17 | 0.56 | 0.50 | 0.02 | 0.18 | 0.86 | 0.91 | -0.31 | -2.55 | 0.25 | -0.43 |
| Q58WW2 | DDB1- and CUL4-associated factor 6 | DCAF6 | -1.63 | 3.97 | -0.78 | 0.53 | 0.78 | 1.19 | 1.16 | -0.96 | -0.99 | 1.00 | -0.91 | -1.02 |
| Q9ULJ8 | Neurabin-1 | PPP1R9A | -1.64 | 3.14 | 0.56 | 0.69 | -0.30 | 0.46 | 1.20 | -1.82 | 1.20 | -1.17 | -0.49 | -0.33 |
| O75400 | Pre-mRNA-processing factor 40 homolog A | PRPF40A | -1.64 | 2.76 | -0.04 | -0.30 | 1.30 | 0.76 | 0.67 | -1.94 | -1.43 | 0.14 | 0.44 | 0.40 |
| P49619 | Diacylglycerol kinase gamma | DGKG | -1.65 | 2.36 | 1.23 | 0.11 | -0.59 | 0.55 | 0.86 | 0.02 | -0.78 | -2.26 | 0.43 | 0.44 |
| O15117 | FYN-binding protein 1 | FYB1 | -1.65 | 1.13 | -1.60 | 0.88 | 0.84 | 0.51 | 0.63 | -1.67 | -0.93 | 0.37 | 0.35 | 0.62 |
| Q15031 | Probable leucine--tRNA ligase, mitochondrial | LARS2 | -1.65 | 2.45 | -0.53 | 0.84 | 1.32 | 0.27 | 0.31 | -1.16 | -1.16 | -1.46 | 0.65 | 0.93 |
| P14867 | Gamma-aminobutyric acid receptor subunit alpha-1 | GABRA1 | -1.65 | 1.65 | -0.16 | 0.53 | 0.52 | 0.28 | 0.58 | -2.77 | 0.08 | 0.39 | 0.25 | 0.31 |
| Q9BZL4 | Protein phosphatase 1 regulatory subunit 12C | PPP1R12C | -1.66 | 2.62 | 1.35 | 0.78 | 0.32 | 0.10 | -0.22 | 0.12 | 0.77 | -2.12 | 0.06 | -1.15 |
| Q8NFI3 | Cytosolic endo-beta-N-acetylglucosaminidase | ENGASE | -1.66 | 2.94 | 0.03 | 0.36 | 0.11 | 0.27 | 1.70 | -1.20 | -1.68 | 0.88 | 0.39 | -0.86 |
| O75962 | Triple functional domain protein | TRIO | -1.67 | 2.18 | -0.29 | -0.30 | 1.62 | 0.51 | 0.49 | -1.56 | 0.54 | -1.31 | 0.92 | -0.64 |
| Q13439 | Golgin subfamily A member 4 | GOLGA4 | -1.67 | 1.31 | -1.31 | 1.26 | 0.42 | 0.62 | 0.42 | -1.94 | -0.75 | 0.23 | 0.61 | 0.44 |
| O75420 | GRB10-interacting GYF protein 1 | GIGYF1 | -1.67 | 3.79 | 0.98 | 1.13 | 0.48 | 0.45 | -0.11 | 0.33 | 0.63 | -1.40 | -0.61 | -1.87 |
| Q9UBW7 | Zinc finger MYM-type protein 2 | ZMYM2 | -1.68 | 1.68 | -0.55 | 0.95 | 0.58 | 0.85 | -0.11 | -2.24 | -0.81 | -0.10 | 0.77 | 0.66 |
| Q9BY12 | S phase cyclin A-associated protein in the endoplasmic reticulum | SCAPER | -1.68 | 1.33 | -0.44 | 0.65 | 0.30 | 0.63 | 0.35 | -1.76 | -1.64 | -0.05 | 0.77 | 1.19 |
| Q9BZF2 | Oxysterol-binding protein-related protein 7 | OSBPL7 | -1.68 | 1.77 | 0.89 | 0.30 | -1.31 | 1.08 | 0.79 | 0.27 | -1.28 | -1.48 | 0.84 | -0.10 |
| Q8IUS5 | Epoxide hydrolase 4 | EPHX4 | -1.68 | 2.53 | 0.42 | 1.11 | -1.09 | 1.41 | 0.37 | -1.04 | -0.98 | -1.26 | 0.36 | 0.71 |
| P40424 | Pre-B-cell leukemia transcription factor 1 | PBX1 | -1.68 | 3.71 | 1.15 | 1.19 | 0.64 | -0.74 | 0.54 | 0.13 | 0.19 | -2.05 | -0.20 | -0.85 |
| P0DP25 | Calmodulin-3 | CALM3 | -1.68 | 1.19 | -0.36 | 0.41 | 0.34 | 0.69 | 0.28 | -1.65 | -1.65 | 1.54 | 0.47 | -0.08 |
| P56945 | Breast cancer anti-estrogen resistance protein 1 | BCAR1 | -1.68 | 3.41 | -0.88 | 1.56 | -0.15 | 0.88 | 1.26 | -0.98 | 0.45 | -1.40 | -0.44 | -0.30 |
| P98179 | RNA-binding protein 3 | RBM3 | -1.70 | 2.28 | 1.25 | 1.39 | -1.10 | 0.65 | -0.11 | -0.80 | 0.14 | -1.17 | 0.82 | -1.07 |
| Q8TAG9 | Exocyst complex component 6 | EXOC6 | -1.70 | 4.44 | 0.77 | 1.17 | -0.32 | 0.48 | 1.01 | -1.35 | -0.72 | -1.75 | 0.43 | 0.28 |
| Q9H6S0 | 3'-5' RNA helicase YTHDC2 | YTHDC2 | -1.71 | 2.23 | 0.84 | 0.02 | -0.39 | 0.94 | 0.69 | -1.30 | 0.95 | -1.58 | 0.83 | -1.00 |
| Q99578 | GTP-binding protein Rit2 | RIT2 | -1.71 | 2.76 | 1.48 | 0.34 | -0.95 | 0.99 | 0.48 | -0.91 | -0.63 | -1.12 | 1.17 | -0.85 |
| O60231 | Pre-mRNA-splicing factor ATP-dependent RNA helicase DHX16 | DHX16 | -1.71 | 5.08 | 1.32 | 1.17 | -0.45 | 0.69 | 0.53 | -0.42 | -1.26 | -1.74 | 0.34 | -0.17 |
| O14523 | Phospholipid transfer protein C2CD2L | C2CD2L | -1.71 | 1.64 | 1.09 | 0.05 | 1.13 | -0.16 | -0.43 | 0.74 | 0.62 | -2.25 | -0.25 | -0.54 |
| O95263 | High affinity cAMP-specific and IBMX-insensitive 3',5'-cyclic phos | PDE8B | -1.71 | 1.79 | 1.59 | -0.81 | 1.42 | 0.47 | -0.89 | 0.23 | -0.76 | -1.04 | 0.63 | -0.83 |
| Q9BYC9 | 39S ribosomal protein L20, mitochondrial | MRPL20 | -1.71 | 2.99 | -0.25 | -0.43 | 0.67 | 1.05 | 1.42 | -1.66 | -0.67 | -0.01 | 0.93 | -1.06 |
| P12755 | Ski oncogene | SKI | -1.72 | 4.61 | 1.78 | 0.99 | -0.34 | 0.41 | 0.26 | -1.18 | -1.13 | 0.17 | 0.35 | -1.30 |
| Q5SZQ8 | CUGBP Elav-like family member 3 | CELF3 | -1.72 | 2.71 | 2.03 | -0.49 | -0.76 | 0.11 | 1.57 | -0.50 | -0.58 | -0.82 | 0.00 | -0.57 |
| Q8NCB2 | CaM kinase-like vesicle-associated protein | CAMKV | -1.72 | 3.33 | 0.54 | 0.39 | 0.04 | 1.09 | 0.71 | -0.73 | -0.18 | -2.35 | 0.82 | -0.33 |
| Q96MW1 | Coiled-coil domain-containing protein 43 | CCDC43 | -1.72 | 1.42 | 1.08 | 0.48 | 0.52 | -0.54 | -0.02 | 1.01 | 0.86 | -1.73 | -1.51 | -0.16 |
| O14490 | Disks large-associated protein 1 | DLGAP1 | -1.72 | 2.05 | 0.35 | 0.71 | 0.15 | 0.35 | 0.51 | 0.24 | 0.59 | -2.53 | 0.57 | -0.93 |
| Q16875 | 6-phosphofructo-2-kinase/fructose-2,6-bisphosphatase 3 | PFKFB3 | -1.72 | 1.38 | -1.09 | 0.71 | 0.25 | 0.87 | 0.74 | -1.10 | -1.94 | 0.60 | 0.78 | 0.19 |
| Q8N5V2 | Ephexin-1 | NGEF | -1.73 | 2.18 | 0.69 | 0.75 | -0.43 | 0.69 | 0.39 | -0.57 | 0.90 | -2.35 | 0.44 | -0.51 |
| Q9Y3C5 | RING finger protein 11 | RNF11 | -1.73 | 3.58 | -0.83 | 2.04 | 0.86 | 0.21 | 0.49 | -0.96 | -1.02 | -0.13 | 0.35 | -1.01 |
| Q14206 | Calcipressin-2 | RCAN2 | -1.74 | 1.71 | -0.74 | 1.37 | -0.99 | 1.23 | 0.85 | -0.81 | -0.88 | -1.06 | 0.89 | 0.14 |
| O94921 | Cyclin-dependent kinase 14 | CDK14 | -1.74 | 2.80 | 0.66 | 0.77 | -0.27 | 0.79 | 0.52 | 0.05 | -0.41 | -2.48 | 0.74 | -0.38 |
| Q96G28 | Cilia- and flagella-associated protein 36 | CFAP36 | -1.75 | 3.09 | 1.22 | 1.24 | -1.14 | 0.41 | 0.78 | -1.02 | -0.94 | -1.28 | 0.56 | 0.16 |
| Q9BSH4 | Translational activator of cytochrome c oxidase 1 | TACO1 | -1.75 | 3.97 | 0.27 | 0.27 | 1.59 | 0.52 | 0.31 | -2.24 | -0.27 | -0.75 | 0.52 | -0.21 |
| P13804 | Electron transfer flavoprotein subunit alpha, mitochondrial | ETFA | -1.75 | 2.48 | -0.02 | 0.61 | 0.30 | 0.19 | 1.20 | -0.44 | -2.42 | 0.81 | 0.28 | -0.50 |
| Q9UN37 | Vacuolar protein sorting-associated protein 4A | VPS4A | -1.75 | 2.26 | 0.38 | 0.89 | -0.18 | 0.95 | 0.10 | -0.32 | -0.01 | -2.57 | 0.65 | 0.10 |
| Q8NDH6 | Islet cell autoantigen 1-like protein | ICA1L | -1.75 | 2.50 | -0.56 | 1.69 | -0.84 | 0.90 | 1.02 | -0.80 | -0.69 | -0.96 | 0.89 | -0.66 |
| Q9Y5I4 | Protocadherin alpha-C2 | PCDHAC2 | -1.75 | 2.47 | 1.28 | 0.92 | -0.79 | 0.32 | 0.47 | -0.17 | 1.18 | -1.55 | -0.41 | -1.26 |
| O14737 | Programmed cell death protein 5 | PDCD5 | -1.76 | 1.97 | -0.81 | 1.36 | 0.54 | -0.85 | 1.68 | -0.81 | -0.91 | 0.17 | -0.92 | 0.55 |
| O14522 | Receptor-type tyrosine-protein phosphatase T | PTPRT | -1.76 | 2.77 | -0.16 | 0.95 | 0.11 | 0.40 | 1.12 | -1.50 | -1.63 | 0.82 | 0.67 | -0.77 |
| P49674 | Casein kinase I isoform epsilon | CSNK1E | -1.76 | 4.89 | 1.33 | 0.58 | 0.50 | 0.58 | 0.39 | -0.11 | -0.54 | -2.19 | 0.40 | -0.94 |
| Q16288 | NT-3 growth factor receptor | NTRK3 | -1.76 | 3.49 | 0.40 | 0.87 | 0.58 | 0.52 | 0.55 | -0.02 | -2.57 | -0.69 | 0.28 | 0.07 |
| Q9H1Y0 | Autophagy protein 5 | ATG5 | -1.77 | 3.44 | 0.25 | 0.94 | 0.55 | 0.28 | 0.83 | 0.13 | 1.13 | -1.58 | -1.23 | -1.30 |
| Q9C0H9 | SRC kinase signaling inhibitor 1 | SRCIN1 | -1.77 | 6.54 | 0.82 | 0.86 | 0.45 | 1.47 | 0.07 | -1.19 | -0.43 | -1.46 | 0.51 | -1.10 |
| Q08043 | Alpha-actinin-3 | ACTN3 | -1.77 | 1.40 | -1.35 | 0.37 | 0.69 | 0.69 | 1.07 | -1.46 | -1.43 | 0.25 | 0.75 | 0.39 |
| O60551 | Glycylpeptide N-tetradecanoyltransferase 2 | NMT2 | -1.77 | 4.41 | 0.80 | 0.20 | 1.44 | -0.09 | 0.75 | -0.35 | 0.02 | -2.28 | 0.11 | -0.59 |
| Q86W42 | THO complex subunit 6 homolog | THOC6 | -1.77 | 4.72 | 0.01 | 1.51 | 1.60 | 0.06 | -0.05 | -1.21 | -0.71 | -1.47 | 0.14 | 0.11 |
| Q969V3 | Nicalin | NCLN | -1.78 | 4.52 | 1.30 | 0.38 | 2.06 | 0.27 | -0.66 | -0.65 | -0.51 | -1.03 | -0.67 | -0.49 |
| Q9UPU7 | TBC1 domain family member 2B | TBC1D2B | -1.78 | 2.78 | -0.35 | 1.32 | -0.07 | 0.92 | 0.56 | -0.39 | -1.46 | -1.72 | 0.72 | 0.46 |
| Q96NW7 | Leucine-rich repeat-containing protein 7 | LRRC7 | -1.78 | 3.48 | 0.65 | 2.09 | 1.32 | -0.54 | -0.60 | -0.50 | -0.50 | -0.85 | -0.53 | -0.53 |
| Q9H4G0 | Band 4.1-like protein 1 | EPB41L1 | -1.78 | 4.58 | 0.62 | 1.38 | -0.50 | 0.89 | 0.71 | -0.73 | 0.27 | -1.66 | 0.32 | -1.30 |
| Q96C86 | m7GpppX diphosphatase | DCPS | -1.78 | 1.98 | -0.79 | 1.22 | 0.82 | 0.21 | 0.45 | -1.25 | -1.87 | 0.22 | 0.09 | 0.91 |
| O75038 | 1-phosphatidylinositol 4,5-bisphosphate phosphodiesterase eta-2 | PLCH2 | -1.79 | 5.21 | -0.04 | 1.88 | 0.56 | 0.49 | 0.40 | -1.20 | -1.17 | -0.11 | 0.50 | -1.31 |
| Q12851 | Mitogen-activated protein kinase kinase kinase kinase 2 | MAP4K2 | -1.79 | 2.51 | -0.05 | 0.80 | -0.19 | -0.24 | 1.89 | -0.83 | -1.83 | -0.44 | 0.27 | 0.62 |
| Q96JH7 | Deubiquitinating protein VCIP135 | VCPIP1 | -1.80 | 1.37 | 0.23 | -0.75 | 1.14 | -0.20 | 1.05 | 0.51 | -0.20 | -2.02 | 1.00 | -0.76 |

| | | | | | | | | | | | | | | |
|---|---|---|---|---|---|---|---|---|---|---|---|---|---|---|
| Q8NDX5 | Polyhomeotic-like protein 3 | PHC3 | -1.80 | 2.77 | 0.56 | 0.76 | 0.70 | 0.54 | -0.07 | -1.25 | -2.19 | -0.33 | 0.51 | 0.78 |
| O00507 | Probable ubiquitin carboxyl-terminal hydrolase FAF-Y | USP9Y | -1.80 | 3.53 | 1.09 | 1.05 | -1.23 | 0.85 | 0.96 | -1.04 | -1.13 | 0.20 | 0.29 | -1.03 |
| O43524 | Forkhead box protein O3 | FOXO3 | -1.80 | 6.27 | 1.59 | -0.23 | 0.09 | 0.76 | 1.37 | -0.04 | -0.38 | -1.53 | -0.54 | -1.09 |
| Q9BXB4 | Oxysterol-binding protein-related protein 11 | OSBPL11 | -1.80 | 2.01 | -0.32 | 1.34 | 0.29 | 0.66 | -0.02 | 0.27 | -1.56 | -1.83 | 0.59 | 0.59 |
| Q9Y2J0 | Rabphilin-3A | RPH3A | -1.80 | 3.93 | 0.20 | 1.70 | -0.54 | 0.97 | 0.51 | -1.12 | 0.31 | -1.58 | 0.32 | -0.79 |
| O00555 | Voltage-dependent P/Q-type calcium channel subunit alpha-1A | CACNA1A | -1.80 | 2.57 | 1.13 | 0.44 | -0.76 | -0.59 | 2.10 | 0.34 | -0.67 | -1.01 | -0.74 | -0.24 |
| P34903 | Gamma-aminobutyric acid receptor subunit alpha-3 | GABRA3 | -1.81 | 4.96 | -0.61 | 0.91 | 0.45 | 1.46 | 0.99 | -0.72 | -0.91 | -1.34 | 0.69 | -0.93 |
| Q9P2U7 | Vesicular glutamate transporter 1 | SLC17A7 | -1.81 | 3.64 | 0.52 | 1.65 | -0.28 | 0.71 | 0.17 | -0.60 | 0.52 | -1.38 | 0.35 | -1.66 |
| P41743 | Protein kinase C iota type | PRKCI | -1.81 | 2.71 | 0.10 | 2.09 | -0.33 | 0.33 | 0.13 | 0.05 | -0.44 | -2.03 | 0.23 | -0.11 |
| P54259 | Atrophin-1 | ATN1 | -1.81 | 3.64 | 1.94 | -0.55 | -0.66 | 1.05 | 1.22 | -0.54 | -0.57 | -0.87 | -0.45 | -0.56 |
| Q15393 | Splicing factor 3B subunit 3 | SF3B3 | -1.82 | 1.06 | -1.27 | 0.97 | 0.28 | 0.76 | 0.47 | -1.22 | -1.68 | 0.12 | 0.79 | 0.79 |
| Q9BUQ8 | Probable ATP-dependent RNA helicase DDX23 | DDX23 | -1.82 | 1.37 | -0.52 | 1.06 | 0.41 | 0.46 | 0.09 | -0.95 | -2.27 | 0.30 | 0.55 | 0.88 |
| Q96D05 | Protein FAM241B | FAM241B | -1.82 | 1.60 | -0.28 | 1.15 | -0.10 | 0.70 | 0.20 | -1.74 | -1.66 | 0.16 | 0.83 | 0.73 |
| Q8IZ83 | Aldehyde dehydrogenase family 16 member A1 | ALDH16A1 | -1.82 | 3.00 | 1.00 | 1.10 | -0.03 | 0.74 | -0.29 | -2.16 | -0.96 | -0.23 | 0.69 | 0.15 |
| Q06828 | Fibromodulin | FMOD | -1.83 | 4.14 | 1.19 | 0.32 | -0.26 | 0.62 | 1.12 | -0.90 | 0.15 | -2.22 | -0.04 | 0.01 |
| C9JLW8 | Mapk-regulated corepressor-interacting protein 1 | MCRIP1 | -1.84 | 2.90 | 0.90 | 1.30 | -0.81 | -0.65 | 1.77 | -0.61 | -0.64 | -1.01 | 0.36 | -0.60 |
| Q969Q5 | Ras-related protein Rab-24 | RAB24 | -1.84 | 2.95 | 0.34 | 1.40 | 0.05 | 0.79 | -0.08 | -0.46 | -0.27 | -2.40 | 0.49 | 0.13 |
| P54762 | Ephrin type-B receptor 1 | EPHB1 | -1.84 | 2.71 | -0.30 | -0.15 | 0.63 | 0.51 | 1.64 | -1.65 | -1.59 | 0.41 | 0.14 | 0.35 |
| O95786 | Probable ATP-dependent RNA helicase DDX58 | DDX58 | -1.85 | 2.41 | -1.29 | 0.89 | 1.17 | 0.55 | 0.83 | -0.63 | -1.04 | -1.48 | 0.66 | 0.34 |
| Q9Y2I6 | Ninein-like protein | NINL | -1.85 | 2.01 | 0.45 | 0.70 | 0.31 | 0.36 | 0.21 | -0.42 | -2.72 | 0.22 | 0.36 | 0.53 |
| Q9UJU6 | Drebrin-like protein | DBNL | -1.85 | 1.73 | -1.22 | 0.12 | 0.58 | 0.36 | 1.89 | -1.37 | -1.11 | 0.45 | 0.38 | -0.08 |
| Q9Y2X7 | ARF GTPase-activating protein GIT1 | GIT1 | -1.85 | 2.79 | 0.13 | 1.41 | 0.02 | 0.86 | 0.00 | -0.97 | 0.33 | -2.23 | 0.50 | -0.04 |
| Q99767 | Amyloid-beta A4 precursor protein-binding family A member 2 | APBA2 | -1.85 | 5.40 | 0.34 | 0.78 | 1.19 | 1.11 | 0.02 | -1.08 | -1.75 | -0.71 | 0.72 | -0.61 |
| Q9Y296 | Trafficking protein particle complex subunit 4 | TRAPPC4 | -1.86 | 1.82 | 1.07 | -0.73 | 1.52 | -0.73 | 0.65 | 1.29 | -0.69 | -0.97 | -0.71 | -0.70 |
| P29120 | Neuroendocrine convertase 1 | PCSK1 | -1.86 | 3.29 | 1.05 | 1.36 | -1.28 | 0.62 | 0.87 | 0.37 | -0.28 | -1.32 | -0.29 | -1.10 |
| O95166 | Gamma-aminobutyric acid receptor-associated protein | GABARAP | -1.86 | 2.86 | -0.98 | 0.49 | 0.97 | 0.55 | 1.36 | -1.11 | -1.12 | 0.89 | 0.14 | -1.19 |
| Q9BQE3 | Tubulin alpha-1C chain | TUBA1C | -1.86 | 1.71 | 0.08 | 1.11 | 0.16 | -1.20 | 1.56 | 0.12 | 0.25 | -1.45 | -1.18 | 0.55 |
| Q9ULX3 | RNA-binding protein NOB1 | NOB1 | -1.86 | 2.57 | -0.33 | 1.28 | -0.52 | -0.38 | 2.36 | -0.39 | -0.43 | -0.78 | -0.39 | -0.44 |
| P56524 | Histone deacetylase 4 | HDAC4 | -1.87 | 3.84 | 0.46 | 0.99 | -0.63 | 0.96 | 1.07 | -0.92 | -0.28 | -1.82 | 0.83 | -0.67 |
| A6NEL2 | Ankyrin repeat domain-containing protein SOWAHB | SOWAHB | -1.87 | 5.15 | -0.73 | 0.41 | 1.26 | 1.31 | 1.03 | 0.51 | -0.92 | -1.16 | -0.83 | -0.88 |
| Q6UUV9 | CREB-regulated transcription coactivator 1 | CRTC1 | -1.87 | 3.88 | 1.20 | 1.00 | -0.20 | 0.43 | 0.49 | -1.22 | 0.79 | -1.77 | 0.16 | -0.89 |
| Q02410 | Amyloid-beta A4 precursor protein-binding family A member 1 | APBA1 | -1.88 | 3.88 | 1.42 | 1.63 | -0.29 | -0.10 | 0.18 | 0.24 | 0.02 | -1.27 | -0.26 | -1.57 |
| Q9HAD4 | WD repeat-containing protein 41 | WDR41 | -1.88 | 2.89 | 0.40 | 1.29 | -1.09 | 1.10 | 0.71 | -0.65 | -0.85 | -1.14 | 1.06 | -0.82 |
| Q9HAU0 | Pleckstrin homology domain-containing family A member 5 | PLEKHA5 | -1.88 | 1.32 | -1.32 | 0.62 | 0.43 | 0.76 | 0.93 | -1.37 | -1.57 | 0.22 | 0.74 | 0.55 |
| O00257 | E3 SUMO-protein ligase CBX4 | CBX4 | -1.88 | 2.32 | -0.63 | 1.16 | -0.84 | 1.29 | 1.13 | -0.73 | -0.74 | -0.97 | 1.04 | -0.71 |
| Q96EV2 | RNA-binding protein 33 | RBM33 | -1.89 | 2.91 | 1.61 | -0.41 | -1.09 | 1.11 | 1.30 | -0.42 | -0.02 | -1.15 | -0.16 | -0.78 |
| Q9P0J7 | E3 ubiquitin-protein ligase KCMF1 | KCMF1 | -1.90 | 7.00 | -0.52 | 1.22 | 1.02 | 0.91 | 1.41 | -0.70 | -0.77 | -1.13 | -0.68 | -0.76 |
| Q6ZSY5 | Protein phosphatase 1 regulatory subunit 3F | PPP1R3F | -1.91 | 3.04 | -0.19 | 1.29 | 0.45 | 0.88 | 0.11 | 0.44 | -1.78 | -1.65 | 0.47 | -0.03 |
| O43572 | A-kinase anchor protein 10, mitochondrial | AKAP10 | -1.91 | 2.44 | -0.41 | 1.26 | 0.46 | 0.56 | 0.36 | -1.09 | -2.23 | 0.37 | 0.41 | 0.33 |
| O95670 | V-type proton ATPase subunit G 2 | ATP6V1G2 | -1.91 | 4.80 | 0.64 | 1.29 | 1.53 | -0.03 | -0.28 | -1.82 | -0.15 | -1.08 | -0.05 | -0.05 |
| A6NFN3 | RNA binding protein fox-1 homolog 3 | RBFOX3 | -1.91 | 2.84 | 0.67 | -0.27 | -0.23 | 1.08 | 1.17 | -0.83 | -2.07 | -0.11 | 0.91 | -0.33 |
| Q15771 | Ras-related protein Rab-30 | RAB30 | -1.92 | 0.99 | -0.98 | 0.39 | 0.68 | 0.70 | 0.36 | -1.59 | -1.61 | 0.95 | 0.81 | 0.30 |
| Q8N1F7 | Nuclear pore complex protein Nup93 | NUP93 | -1.92 | 2.59 | -0.92 | 1.13 | 0.48 | 0.85 | 0.72 | -1.08 | -1.82 | -0.51 | 0.63 | 0.52 |
| O75531 | Barrier-to-autointegration factor | BANF1 | -1.93 | 2.86 | 1.66 | 1.11 | 1.10 | -0.65 | -0.77 | -0.67 | -0.62 | -1.02 | -0.74 | 0.61 |
| Q14202 | Zinc finger MYM-type protein 3 | ZMYM3 | -1.95 | 2.15 | -0.32 | 0.42 | 0.96 | 0.67 | 0.35 | -1.68 | -1.90 | 0.37 | 0.63 | 0.50 |
| Q9ULX6 | A-kinase anchor protein 8-like | AKAP8L | -1.95 | 2.37 | 0.76 | 0.93 | -0.51 | 0.66 | 0.34 | 0.28 | -2.09 | -1.34 | 0.63 | 0.33 |
| Q9NZT2 | Opioid growth factor receptor | OGFR | -1.95 | 3.52 | 0.89 | 1.42 | -0.96 | 1.11 | 0.24 | -0.90 | -0.84 | -1.16 | 0.84 | -0.63 |
| Q92572 | AP-3 complex subunit sigma-1 | AP3S1 | -1.95 | 2.55 | 1.06 | 1.24 | -1.42 | 0.71 | 0.66 | -1.18 | -1.26 | 0.26 | 0.39 | -0.45 |
| Q9Y5L4 | Mitochondrial import inner membrane translocase subunit Tim13 | TIMM13 | -1.95 | 2.49 | -0.66 | 0.62 | 1.80 | -0.66 | 1.12 | -0.71 | -0.68 | -0.97 | -0.75 | 0.89 |
| Q86XI8 | Uncharacterized protein ZSWIM9 | ZSWIM9 | -1.95 | 1.50 | -0.95 | 0.14 | 0.60 | 0.49 | 1.28 | -1.54 | -1.59 | 0.25 | 0.61 | 0.71 |
| Q8IVH8 | Mitogen-activated protein kinase kinase kinase kinase 3 | MAP4K3 | -1.96 | 2.46 | 0.40 | 0.77 | 0.25 | 0.72 | 0.18 | -1.76 | -1.83 | 0.91 | 0.55 | -0.18 |
| Q9Y3I1 | F-box only protein 7 | FBXO7 | -1.96 | 5.26 | 1.09 | 0.58 | 0.05 | 0.94 | 0.80 | -1.51 | -1.40 | -0.98 | 0.78 | -0.35 |
| Q8IZ57 | Neurensin-1 | NRSN1 | -1.97 | 3.26 | 0.65 | 1.45 | -1.18 | 0.32 | 1.39 | 0.33 | -0.48 | -1.26 | -0.19 | -1.03 |
| Q96QZ7 | Membrane-associated guanylate kinase, WW and PDZ domain-con | MAGI1 | -1.97 | 1.78 | -0.74 | 1.52 | 0.04 | 0.83 | 0.11 | -1.59 | -1.36 | -0.22 | 0.79 | 0.61 |
| Q9H2D6 | TRIO and F-actin-binding protein | TRIOBP | -1.97 | 1.23 | -1.16 | 0.22 | 0.27 | 0.86 | 1.17 | -2.04 | -0.70 | 0.23 | 0.75 | 0.41 |
| Q86UX6 | Serine/threonine-protein kinase 32C | STK32C | -1.97 | 1.94 | -0.68 | 0.58 | 0.71 | 0.69 | 0.62 | -1.90 | -1.55 | 0.46 | 0.64 | 0.44 |
| Q8TBG4 | Ethanolamine-phosphate phospho-lyase | ETNPPL | -1.97 | 2.64 | 0.18 | 0.46 | 0.37 | 0.67 | 0.76 | -0.91 | -2.48 | 0.04 | 0.75 | 0.17 |
| Q4G0J3 | La-related protein 7 | LARP7 | -1.97 | 3.87 | 0.33 | -0.92 | 1.10 | 1.49 | 0.85 | -0.56 | -0.85 | -1.25 | 0.71 | -0.89 |
| O95696 | Bromodomain-containing protein 1 | BRD1 | -1.98 | 2.38 | -0.67 | 1.44 | -0.85 | 1.16 | 1.08 | -0.71 | -0.71 | -0.90 | 0.93 | -0.76 |
| Q5U5X0 | Complex III assembly factor LYRM7 | LYRM7 | -1.99 | 3.84 | -0.91 | 1.01 | 1.27 | 0.89 | 0.57 | -1.01 | -1.10 | -1.39 | 0.45 | 0.23 |
| O43324 | Eukaryotic translation elongation factor 1 epsilon-1 | EEF1E1 | -1.99 | 2.77 | 0.64 | -0.24 | 1.55 | 0.07 | 0.36 | -1.22 | -1.12 | -1.40 | 1.12 | 0.24 |
| Q12888 | TP53-binding protein 1 | TP53BP1 | -2.01 | 3.29 | 0.29 | 0.20 | 1.36 | 0.50 | 0.35 | -1.63 | -1.86 | 0.44 | 0.52 | -0.18 |
| Q8N6R0 | eEF1A lysine and N-terminal methyltransferase | EEF1AKNMT | -2.01 | 3.98 | 1.34 | -0.05 | 1.13 | 0.52 | -0.02 | 0.54 | -1.65 | -1.31 | 0.38 | -0.89 |
| Q15256 | Receptor-type tyrosine-protein phosphatase R | PTPRR | -2.02 | 3.66 | 0.51 | 2.06 | -0.88 | 0.29 | 0.85 | -0.73 | 0.43 | -1.06 | -0.73 | -0.74 |
| P31644 | Gamma-aminobutyric acid receptor subunit alpha-5 | GABRA5 | -2.02 | 3.87 | -0.44 | 1.43 | -0.75 | 1.26 | 1.59 | -0.51 | -0.62 | -0.97 | -0.54 | -0.45 |
| Q86SE5 | RNA-binding Raly-like protein | RALYL | -2.02 | 4.66 | 1.07 | -0.86 | 0.51 | 1.06 | 1.35 | 0.58 | -0.76 | -1.25 | -0.79 | -0.90 |
| Q9UPV7 | PHD finger protein 24 | PHF24 | -2.02 | 4.77 | 0.53 | 1.73 | -0.35 | 0.60 | 0.65 | -1.21 | 0.26 | -1.64 | 0.20 | -0.75 |
| P49454 | Centromere protein F | CENPF | -2.02 | 1.42 | -1.31 | 0.33 | 0.38 | 1.01 | 1.08 | -1.38 | -1.38 | -0.03 | 0.98 | 0.31 |
| Q5VT52 | Regulation of nuclear pre-mRNA domain-containing protein 2 | RPRD2 | -2.03 | 4.02 | 0.03 | 1.17 | 0.36 | 1.02 | 0.44 | -0.66 | -2.14 | -0.98 | 0.51 | 0.24 |
| Q6IPU0 | Centromere protein P | CENPP | -2.04 | 1.21 | -1.37 | 0.69 | 1.14 | 0.50 | 0.37 | -1.44 | -1.40 | 0.18 | 0.58 | 0.76 |
| Q96PE5 | Opalin | OPALIN | -2.04 | 4.15 | 0.15 | -0.27 | 0.99 | 0.86 | 1.25 | -1.42 | 0.34 | -1.78 | 0.32 | -0.44 |
| P62851 | 40S ribosomal protein S25 | RPS25 | -2.04 | 1.34 | 0.19 | -0.02 | 0.53 | 0.45 | 0.37 | 0.13 | -2.79 | 0.48 | 0.54 | 0.13 |
| Q5SQI0 | Alpha-tubulin N-acetyltransferase 1 | ATAT1 | -2.05 | 3.03 | 0.82 | 0.79 | -0.30 | 1.66 | -0.49 | -0.25 | 0.23 | -2.02 | 0.20 | -0.62 |
| Q8N9V3 | WD repeat, SAM and U-box domain-containing protein 1 | WDSUB1 | -2.05 | 2.85 | -0.91 | 1.13 | 0.26 | 0.52 | 1.39 | -1.05 | 0.38 | -1.33 | -1.07 | 0.68 |
| Q9Y2D9 | Zinc finger protein 652 | ZNF652 | -2.05 | 9.44 | 1.52 | 0.75 | 0.45 | 0.77 | 0.65 | -1.06 | -0.90 | -1.44 | 0.18 | -0.92 |
| P83111 | Serine beta-lactamase-like protein LACTB, mitochondrial | LACTB | -2.06 | 2.32 | -1.08 | 1.39 | 0.51 | 0.75 | 0.54 | -0.82 | -1.14 | -1.41 | 0.77 | 0.50 |
| Q92796 | Disks large homolog 3 | DLG3 | -2.06 | 2.14 | 0.96 | 0.30 | -0.04 | -1.11 | 1.91 | 0.26 | 0.56 | -0.77 | -1.14 | -0.93 |
| Q92574 | Hamartin | TSC1 | -2.07 | 4.43 | 0.54 | 1.24 | 0.61 | 0.72 | 0.10 | -0.64 | -0.46 | -2.23 | 0.65 | -0.53 |
| P02533 | Keratin, type I cytoskeletal 14 | KRT14 | -2.07 | 6.04 | 1.65 | -0.38 | 0.83 | 0.30 | 1.11 | -1.35 | -0.68 | -0.16 | 0.03 | -1.35 |
| P41732 | Tetraspanin-7 | TSPAN7 | -2.08 | 3.20 | 1.22 | 1.68 | -0.65 | -0.26 | 0.57 | -1.22 | 0.41 | -1.28 | -0.72 | 0.25 |
| Q12824 | SWI/SNF-related matrix-associated actin-dependent regulator of c | SMARCB1 | -2.09 | 2.74 | -0.28 | 1.09 | 0.44 | 1.04 | 0.10 | -1.75 | -1.60 | -0.16 | 0.86 | 0.26 |
| Q92567 | Protein FAM168A | FAM168A | -2.10 | 3.29 | 1.12 | -0.07 | -0.61 | 0.73 | 1.42 | -1.02 | -1.74 | -0.43 | 0.73 | -0.14 |
| Q15904 | V-type proton ATPase subunit S1 | ATP6AP1 | -2.10 | 3.73 | 1.02 | 0.79 | -1.06 | 1.06 | 0.97 | -0.85 | -0.64 | -1.19 | 0.84 | -0.93 |

| | | | | | | | | | | | | | | |
|---|---|---|---|---|---|---|---|---|---|---|---|---|---|---|
| P12236 | ADP/ATP translocase 3 | SLC25A6 | -2.11 | 4.23 | -0.38 | -0.60 | 1.09 | 1.31 | 1.83 | -0.60 | -0.60 | -0.99 | -0.61 | -0.44 |
| Q9Y3Y2 | Chromatin target of PRMT1 protein | CHTOP | -2.11 | 4.26 | 0.84 | 0.25 | 0.94 | 0.08 | 1.02 | -2.38 | -0.72 | -0.30 | 0.10 | 0.16 |
| Q8IY37 | Probable ATP-dependent RNA helicase DHX37 | DHX37 | -2.12 | 2.60 | -0.81 | 0.22 | 0.62 | 1.21 | 1.03 | -0.94 | -1.50 | -1.11 | 0.97 | 0.31 |
| Q7Z478 | ATP-dependent RNA helicase DHX29 | DHX29 | -2.14 | 5.81 | 0.72 | -0.22 | 0.42 | 1.37 | 1.17 | 0.00 | -0.93 | -1.43 | -1.42 | 0.31 |
| O15523 | ATP-dependent RNA helicase DDX3Y | DDX3Y | -2.14 | 2.43 | 1.33 | -0.85 | -0.29 | 0.94 | 1.03 | -0.78 | -0.78 | -1.02 | 1.24 | -0.82 |
| Q8TF61 | F-box only protein 41 | FBXO41 | -2.14 | 3.79 | 0.68 | 0.98 | -0.55 | 1.06 | 0.69 | -1.54 | -1.57 | -0.06 | 0.78 | -0.46 |
| O75896 | Tumor suppressor candidate 2 | TUSC2 | -2.15 | 5.69 | 0.83 | 1.78 | 0.20 | 0.63 | -0.02 | -1.11 | -0.09 | -1.47 | 0.35 | -1.09 |
| Q16849 | Receptor-type tyrosine-protein phosphatase-like N | PTPRN | -2.15 | 3.11 | 0.57 | 1.52 | -0.07 | 0.47 | 0.09 | -1.93 | 0.79 | -1.32 | 0.10 | -0.21 |
| Q9BTT0 | Acidic leucine-rich nuclear phosphoprotein 32 family member E | ANP32E | -2.16 | 2.28 | 0.13 | 1.15 | -0.07 | 0.62 | 0.34 | -0.71 | -2.45 | -0.14 | 0.61 | 0.53 |
| Q9BTE6 | Alanyl-tRNA editing protein Aarsd1 | AARSD1 | -2.16 | 2.69 | 0.18 | 0.32 | 0.08 | 1.02 | 0.83 | -0.81 | 0.10 | -2.47 | 0.56 | 0.19 |
| Q9BYX4 | Interferon-induced helicase C domain-containing protein 1 | IFIH1 | -2.17 | 4.79 | -0.73 | 1.44 | 1.40 | 0.20 | 0.89 | -0.99 | -1.00 | -1.27 | -0.08 | 0.14 |
| Q9H426 | Regulating synaptic membrane exocytosis protein 4 | RIMS4 | -2.17 | 3.68 | 0.53 | 0.88 | 0.40 | 0.76 | 0.42 | -1.74 | -1.68 | 0.57 | 0.57 | -0.72 |
| Q9Y5W8 | Sorting nexin-13 | SNX13 | -2.17 | 4.31 | 1.27 | 0.11 | 0.03 | 0.98 | 0.69 | -0.35 | -0.91 | -2.19 | 0.42 | -0.05 |
| Q8N3F8 | MICAL-like protein 1 | MICALL1 | -2.19 | 4.42 | -0.56 | 0.72 | 0.69 | 0.14 | 2.25 | -0.06 | -0.74 | -1.04 | -0.72 | -0.67 |
| Q14694 | Ubiquitin carboxyl-terminal hydrolase 10 | USP10 | -2.19 | 3.81 | 0.11 | 1.11 | 0.30 | 0.96 | 0.48 | -0.81 | -0.88 | -2.14 | 0.70 | 0.16 |
| Q9UPQ3 | Arf-GAP with GTPase, ANK repeat and PH domain-containing prot | AGAP1 | -2.20 | 3.01 | 0.60 | 0.83 | 0.54 | 0.37 | 0.31 | -0.10 | 0.26 | -2.69 | 0.16 | -0.29 |
| Q9UIG0 | Tyrosine-protein kinase BAZ1B | BAZ1B | -2.22 | 1.82 | -0.57 | 0.84 | 0.55 | 0.46 | 0.56 | -1.38 | -2.08 | 0.50 | 0.45 | 0.67 |
| Q9H6U6 | Breast carcinoma-amplified sequence 3 | BCAS3 | -2.24 | 3.74 | -0.27 | 1.08 | 0.37 | 1.00 | 0.69 | -0.87 | -0.21 | -2.25 | 0.54 | -0.09 |
| Q9H0U9 | Testis-specific Y-encoded-like protein 1 | TSPYL1 | -2.24 | 1.38 | -0.96 | 0.78 | 0.61 | 0.64 | 0.41 | -1.62 | -1.66 | 0.80 | 0.58 | 0.40 |
| Q9UK39 | Nocturnin | NOCT | -2.25 | 2.38 | -0.34 | 2.51 | -0.51 | -0.40 | 1.03 | -0.42 | -0.36 | -0.69 | -0.47 | -0.36 |
| O43639 | Cytoplasmic protein NCK2 | NCK2 | -2.25 | 2.17 | 0.19 | 0.65 | 1.33 | 0.08 | -0.20 | -0.17 | -0.60 | -2.31 | 0.03 | 0.99 |
| O95886 | Disks large-associated protein 3 | DLGAP3 | -2.26 | 4.83 | -0.80 | 1.03 | 1.71 | 0.38 | 0.92 | -0.94 | -0.86 | -1.28 | 0.16 | -0.32 |
| Q15788 | Nuclear receptor coactivator 1 | NCOA1 | -2.26 | 3.35 | 0.44 | -0.54 | -0.61 | 1.58 | 1.98 | -0.52 | -0.50 | -0.83 | -0.56 | -0.44 |
| Q9UPP1 | Histone lysine demethylase PHF8 | PHF8 | -2.28 | 2.03 | 0.13 | 0.03 | 0.08 | 1.02 | 0.74 | -0.03 | -1.64 | -1.87 | 0.87 | 0.67 |
| Q5QJE6 | Deoxynucleotidyltransferase terminal-interacting protein 2 | DNTTIP2 | -2.29 | 5.86 | 0.63 | 1.04 | -0.08 | 0.68 | 1.27 | -1.18 | -1.17 | -1.58 | 0.43 | -0.04 |
| Q9UI30 | Multifunctional methyltransferase subunit TRM112-like protein | TRMT112 | -2.29 | 4.06 | 0.61 | 0.73 | -0.17 | 0.25 | 1.54 | -1.27 | -1.19 | -1.46 | 0.32 | 0.64 |
| P48634 | Protein PRRC2A | PRRC2A | -2.30 | 3.52 | 0.74 | 0.42 | -0.45 | 0.18 | 1.80 | -1.25 | 0.78 | -1.54 | -0.23 | -0.45 |
| Q86YM7 | Homer protein homolog 1 | HOMER1 | -2.32 | 3.31 | 0.83 | 0.85 | 0.69 | -0.20 | 0.57 | -1.29 | 0.70 | -2.14 | 0.08 | -0.08 |
| O43795 | Unconventional myosin-Ib | MYO1B | -2.33 | 3.29 | 1.07 | 0.55 | 0.55 | 0.74 | -0.19 | -2.16 | -1.15 | 0.58 | 0.42 | -0.41 |
| Q8WXD9 | Caskin-1 | CASKIN1 | -2.33 | 3.38 | 0.96 | 0.97 | -0.61 | 1.10 | 0.24 | -1.01 | 0.08 | -1.87 | 0.77 | -0.63 |
| Q9UJX5 | Anaphase-promoting complex subunit 4 | ANAPC4 | -2.34 | 1.66 | -1.36 | 1.05 | 0.41 | 0.74 | 0.84 | -1.42 | -1.41 | 0.02 | 0.65 | 0.47 |
| Q9Y450 | HBS1-like protein | HBS1L | -2.34 | 1.48 | 0.07 | -0.19 | 0.77 | 0.36 | 0.61 | -0.20 | -2.63 | 0.82 | 0.48 | -0.07 |
| P59190 | Ras-related protein Rab-15 | RAB15 | -2.34 | 3.29 | 0.44 | 1.28 | -0.34 | 0.20 | 1.06 | -1.41 | 0.90 | -1.70 | 0.00 | -0.42 |
| Q14011 | Cold-inducible RNA-binding protein | CIRBP | -2.34 | 6.24 | 1.18 | 0.84 | -0.34 | 1.03 | 0.85 | -1.87 | -0.48 | -0.53 | 0.27 | -0.96 |
| Q9Y2T4 | Serine/threonine-protein phosphatase 2A 55 kDa regulatory subuni | PPP2R2C | -2.34 | 2.95 | 0.37 | 0.62 | 0.27 | 1.22 | 0.08 | -0.15 | -1.61 | -1.84 | 0.94 | 0.11 |
| P23786 | Carnitine O-palmitoyltransferase 2, mitochondrial | CPT2 | -2.35 | 1.46 | -1.39 | 0.65 | 0.85 | 0.61 | 0.81 | -1.47 | -1.44 | 0.49 | 0.49 | 0.40 |
| Q8N9I9 | Probable E3 ubiquitin-protein ligase DTX3 | DTX3 | -2.36 | 3.29 | 1.09 | 0.80 | -0.86 | 1.04 | 0.52 | -0.93 | -1.22 | -1.44 | 0.76 | 0.23 |
| Q13523 | Serine/threonine-protein kinase PRP4 homolog | PRPF4B | -2.37 | 1.36 | -0.76 | 0.49 | 0.33 | 0.46 | 0.96 | -1.53 | -1.84 | 0.62 | 0.76 | 0.51 |
| A8MU93 | Uncharacterized protein C17orf100 | C17orf100 | -2.37 | 3.95 | -0.31 | 1.10 | 0.07 | 1.04 | 1.01 | -1.38 | -1.46 | -1.01 | 0.69 | 0.25 |
| Q5T2E6 | Armadillo-like helical domain-containing protein 3 | ARMH3 | -2.40 | 4.01 | 2.01 | -0.78 | 0.24 | 0.54 | 0.95 | -0.83 | -0.79 | -1.07 | 0.42 | -0.68 |
| Q9UN86 | Ras GTPase-activating protein-binding protein 2 | G3BP2 | -2.41 | 2.90 | 0.69 | 0.16 | -0.24 | 0.78 | 1.10 | -0.53 | 0.06 | -2.46 | 0.51 | -0.07 |
| Q9BZE4 | Nucleolar GTP-binding protein 1 | GTPBP4 | -2.43 | 2.91 | -0.78 | 0.55 | 0.51 | 0.97 | 1.18 | -0.09 | -1.39 | -1.65 | 0.90 | -0.20 |
| Q99627 | COP9 signalosome complex subunit 8 | COPS8 | -2.44 | 3.84 | -0.77 | 0.87 | 0.58 | 1.18 | 0.97 | -0.90 | -0.90 | -1.15 | 1.07 | -0.94 |
| O60925 | Prefoldin subunit 1 | PFDN1 | -2.45 | 2.54 | -0.99 | 0.43 | 0.43 | 1.03 | 1.33 | -1.03 | -1.08 | -1.30 | 0.89 | 0.30 |
| Q99700 | Ataxin-2 | ATXN2 | -2.45 | 2.99 | -0.04 | 0.98 | 0.30 | 0.67 | 0.67 | -1.82 | -1.80 | 0.09 | 0.59 | 0.35 |
| Q5VUA4 | Zinc finger protein 318 | ZNF318 | -2.46 | 4.03 | 0.46 | 0.24 | 0.96 | 0.52 | 0.92 | -1.28 | -1.33 | -1.63 | 0.54 | 0.59 |
| Q6UWE0 | E3 ubiquitin-protein ligase LRSAM1 | LRSAM1 | -2.51 | 2.30 | -0.35 | 1.42 | -0.37 | 1.41 | -0.02 | -0.90 | -0.81 | -0.37 | -1.26 | 1.25 |
| Q9NUA8 | Zinc finger and BTB domain-containing protein 40 | ZBTB40 | -2.54 | 4.18 | -0.58 | 1.03 | 1.06 | 0.62 | 0.85 | -1.15 | -1.18 | -1.50 | 0.59 | 0.26 |
| Q08499 | cAMP-specific 3',5'-cyclic phosphodiesterase 4D | PDE4D | -2.56 | 4.88 | 0.46 | 0.87 | 1.22 | 0.47 | 0.37 | -2.33 | -0.48 | -0.70 | 0.19 | -0.07 |
| Q9H6Y2 | WD repeat-containing protein 55 | WDR55 | -2.59 | 3.93 | 1.15 | 0.32 | 2.18 | -0.65 | 0.04 | -0.62 | -0.03 | -1.05 | -0.71 | -0.64 |
| O95365 | Zinc finger and BTB domain-containing protein 7A | ZBTB7A | -2.60 | 2.77 | -0.67 | 1.18 | 0.60 | 0.99 | 0.27 | -1.50 | -0.15 | -1.71 | 0.66 | 0.33 |
| O60237 | Protein phosphatase 1 regulatory subunit 12B | PPP1R12B | -2.62 | 6.06 | 1.71 | 1.01 | -0.59 | 0.93 | 0.54 | -1.03 | -0.36 | -1.35 | -0.69 | -0.17 |
| P17081 | Rho-related GTP-binding protein RhoQ | RHOQ | -2.63 | 4.94 | 0.16 | 0.83 | 0.09 | 0.82 | 1.39 | 0.34 | -1.20 | -1.57 | -1.24 | 0.37 |
| Q5T5P2 | Sickle tail protein homolog | KIAA1217 | -2.65 | 4.98 | 0.14 | 0.95 | 0.77 | 0.84 | 0.75 | -1.05 | -0.49 | -2.11 | 0.54 | -0.33 |
| Q96A00 | Protein phosphatase 1 regulatory subunit 14A | PPP1R14A | -2.69 | 3.31 | 0.65 | 0.10 | 1.54 | 0.73 | -0.40 | -1.65 | -1.44 | 0.00 | 0.78 | -0.32 |
| Q07343 | cAMP-specific 3',5'-cyclic phosphodiesterase 4B | PDE4B | -2.75 | 5.02 | -0.37 | 1.37 | 0.08 | 1.23 | 0.91 | -0.95 | -0.97 | -1.18 | 0.77 | -0.90 |
| P25189 | Myelin protein P0 | MPZ | -2.83 | 2.30 | 0.13 | -0.43 | 2.74 | -0.02 | -0.20 | -0.16 | -0.22 | -0.53 | -0.72 | -0.59 |
| Q96S38 | Ribosomal protein S6 kinase delta-1 | RPS6KC1 | -2.87 | 7.44 | 0.55 | 1.38 | 1.01 | 0.68 | 0.28 | -1.15 | -0.60 | -1.47 | 0.43 | -1.10 |
| Q9Y6R4 | Mitogen-activated protein kinase kinase kinase 4 | MAP3K4 | -2.90 | 3.17 | -1.29 | 1.07 | 1.47 | 0.87 | 0.46 | -0.59 | -0.20 | -1.60 | 0.13 | -0.34 |
| Q5BKZ1 | DBIRD complex subunit ZNF326 | ZNF326 | -2.90 | 5.75 | 1.08 | 0.20 | 0.64 | 0.57 | 1.12 | -0.61 | -1.91 | -1.12 | 0.53 | -0.51 |
| Q9H792 | Inactive tyrosine-protein kinase PEAK1 | PEAK1 | -2.93 | 3.70 | -0.84 | 0.40 | 0.59 | 1.24 | 1.38 | -0.89 | -0.90 | -1.07 | 0.94 | -0.84 |
| O94762 | ATP-dependent DNA helicase Q5 | RECQL5 | -2.97 | 6.82 | 0.41 | 0.35 | 1.67 | 0.56 | 0.72 | -1.12 | 0.21 | -1.49 | -1.25 | -0.06 |
| Q6ZT07 | TBC1 domain family member 9 | TBC1D9 | -3.02 | 0.89 | -0.90 | 0.99 | 0.94 | -0.96 | 0.98 | -0.95 | -0.97 | 0.83 | 0.99 | -0.95 |
| Q13464 | Rho-associated protein kinase 1 | ROCK1 | -3.02 | 2.95 | -0.22 | 0.43 | 1.79 | 0.51 | -0.07 | -0.01 | -1.18 | 1.08 | -1.15 | -1.18 |
| Q9NVP4 | Double zinc ribbon and ankyrin repeat-containing protein 1 | DZANK1 | -3.02 | 2.60 | 2.11 | 0.25 | -1.00 | 0.61 | 0.32 | -0.92 | -0.87 | -1.10 | 0.46 | 0.14 |
| Q9ULH7 | Myocardin-related transcription factor B | MRTFB | -3.11 | 5.02 | 0.88 | 1.29 | -0.07 | 0.49 | 0.74 | -1.53 | -0.02 | -1.75 | 0.41 | -0.44 |
| P35030 | Trypsin-3 | PRSS3 | -3.24 | 3.55 | 0.41 | 0.37 | -0.49 | 0.70 | 1.71 | -1.23 | 0.29 | 0.67 | -1.26 | -1.18 |
| Q13427 | Peptidyl-prolyl cis-trans isomerase G | PPIG | -3.27 | 5.28 | 0.69 | -0.19 | 0.26 | 1.09 | 1.46 | -1.32 | -1.25 | -0.76 | 0.78 | -0.77 |
| E9PRG8 | Uncharacterized protein C11orf98 | C11orf98 | -3.32 | 1.61 | -1.15 | 0.57 | 0.53 | 0.76 | 0.93 | -1.56 | -1.54 | 0.31 | 0.67 | 0.48 |
| Q6YHK3 | CD109 antigen | CD109 | -3.43 | 14.94 | 0.28 | 0.92 | 1.05 | 1.12 | 1.12 | -0.59 | -1.03 | -1.35 | -0.60 | -0.93 |
| Q09019 | Dystrophia myotonica WD repeat-containing protein | DMWD | -3.57 | 3.45 | -0.09 | 0.45 | 2.20 | 0.20 | -0.09 | -0.50 | -1.87 | -0.33 | 0.16 | -0.14 |
| O60733 | 85/88 kDa calcium-independent phospholipase A2 | PLA2G6 | -3.93 | 5.45 | 0.68 | 1.22 | 1.01 | 0.24 | 0.37 | 0.29 | -1.51 | -1.74 | 0.11 | -0.66 |
| P0C7X4 | Putative ferritin heavy polypeptide-like 19 | FTH1P19 | -3.98 | 1.45 | -1.35 | 0.67 | 0.69 | 0.73 | 0.78 | -1.36 | -1.37 | -0.38 | 0.81 | 0.77 |
