# Supplementary figures and images for: TMEM106B Puncta Is Increased in Multiple Sclerosis Plaques, and Reduced Protein in Mice Results in Delayed Lipid Clearance Following CNS Injury
Source: Cells. 2023 Jun 27;12(13):1734. doi: 10.3390/cells12131734 (PMC10340176; doi:10.3390/cells12131734)

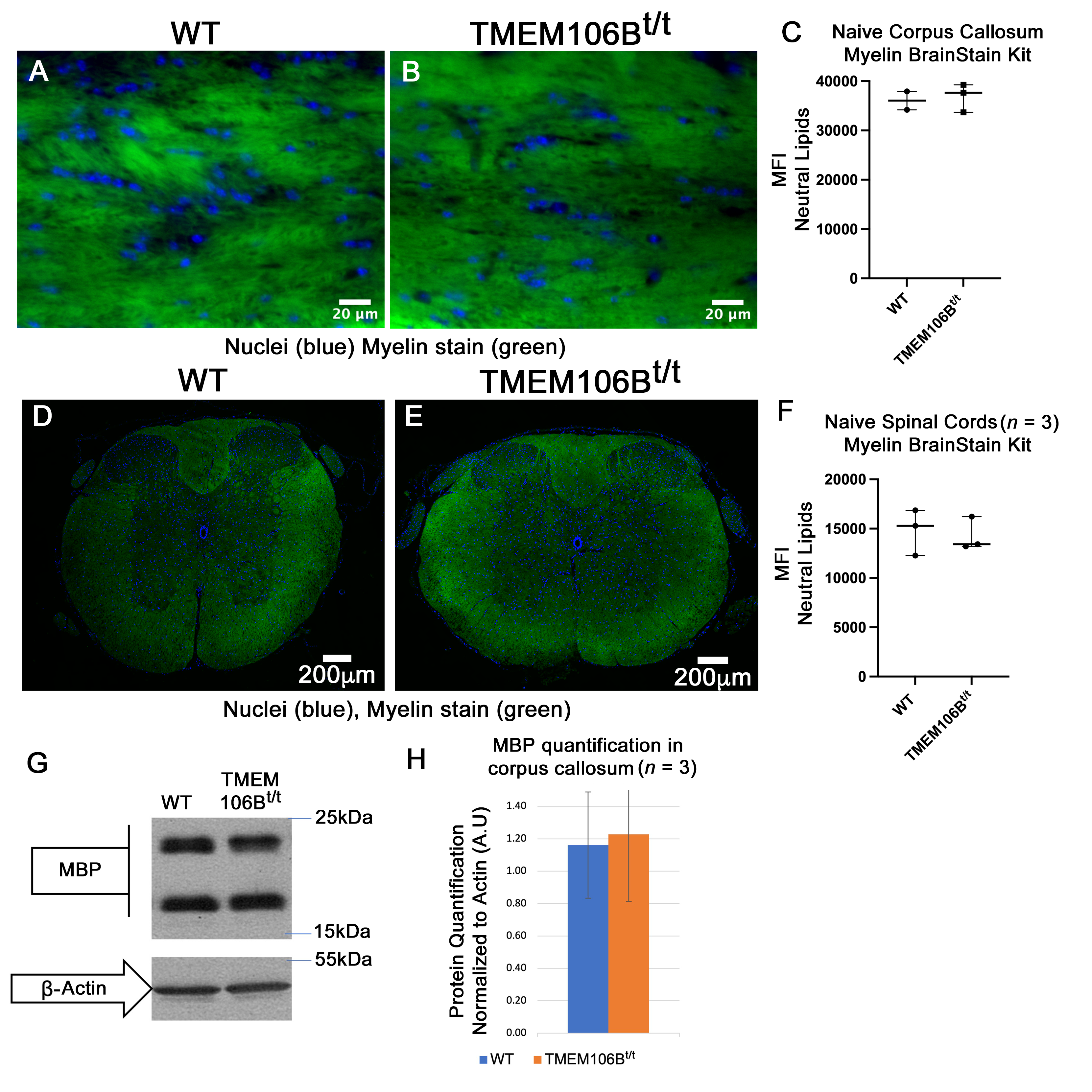

Supplement: Supplementary file 1 [file cells-12-01734-s001.zip › Supplemental Figure S1.tif]

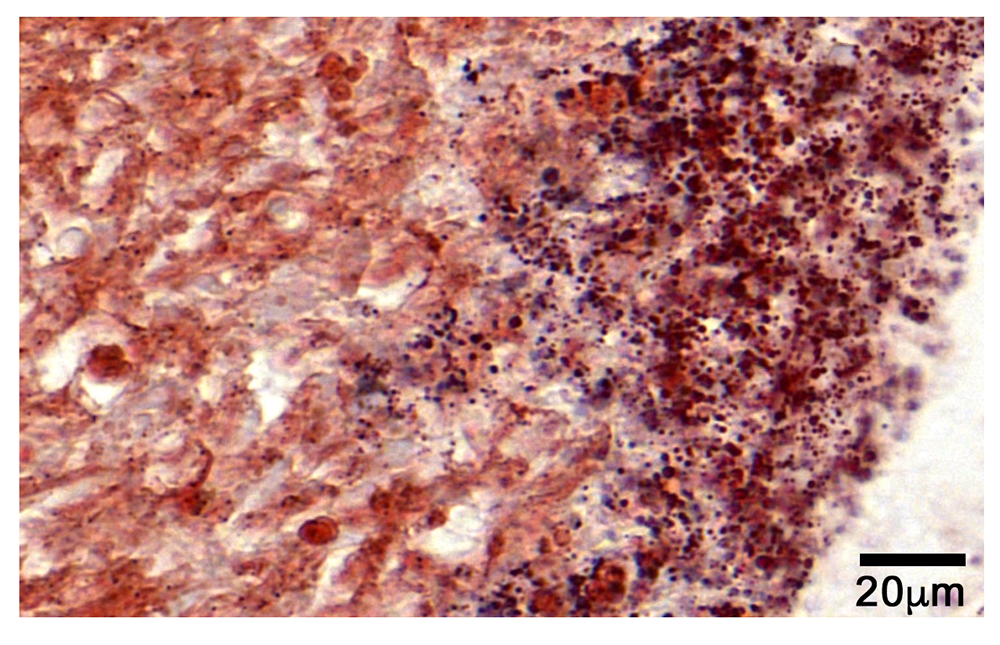

Supplement: Supplementary file 1 [file cells-12-01734-s001.zip › Supplemental Figure S2.tif]

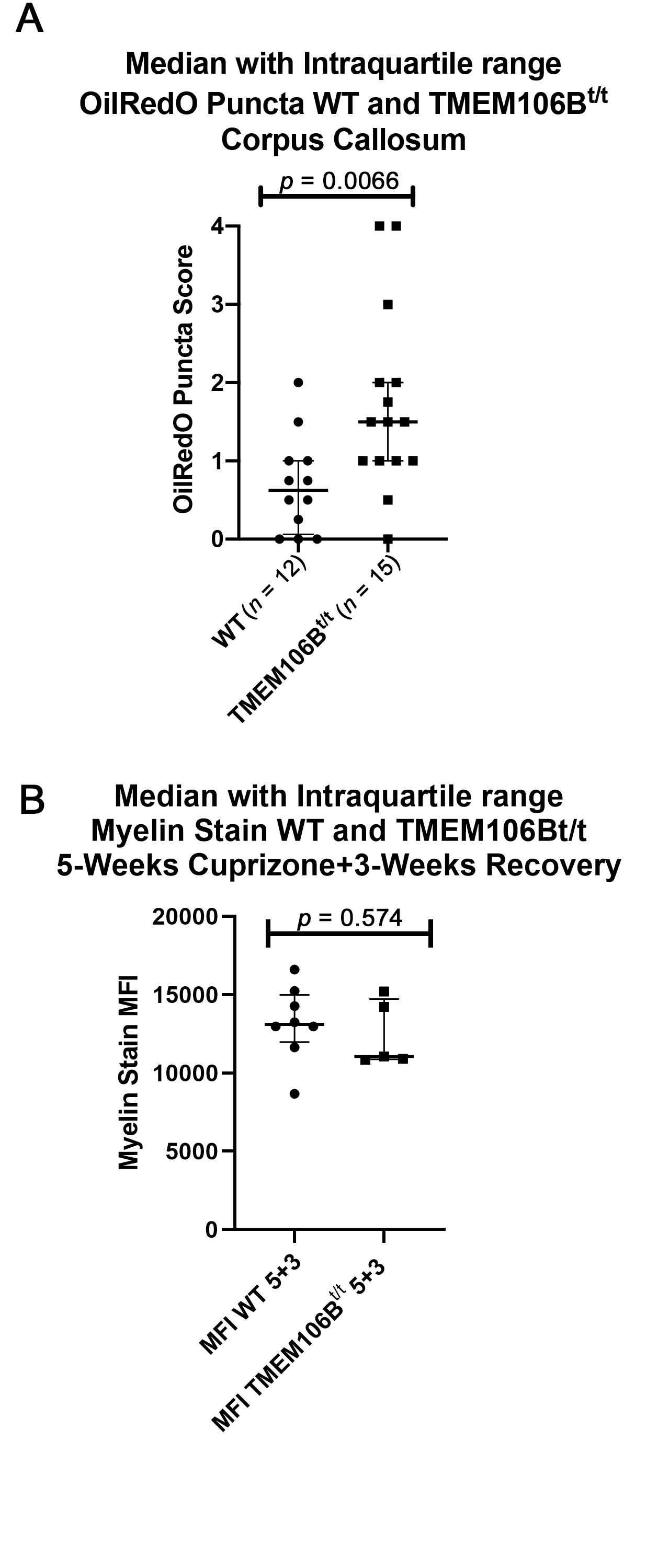

Supplement: Supplementary file 1 [file cells-12-01734-s001.zip › Supplemental Figure S3.tif]

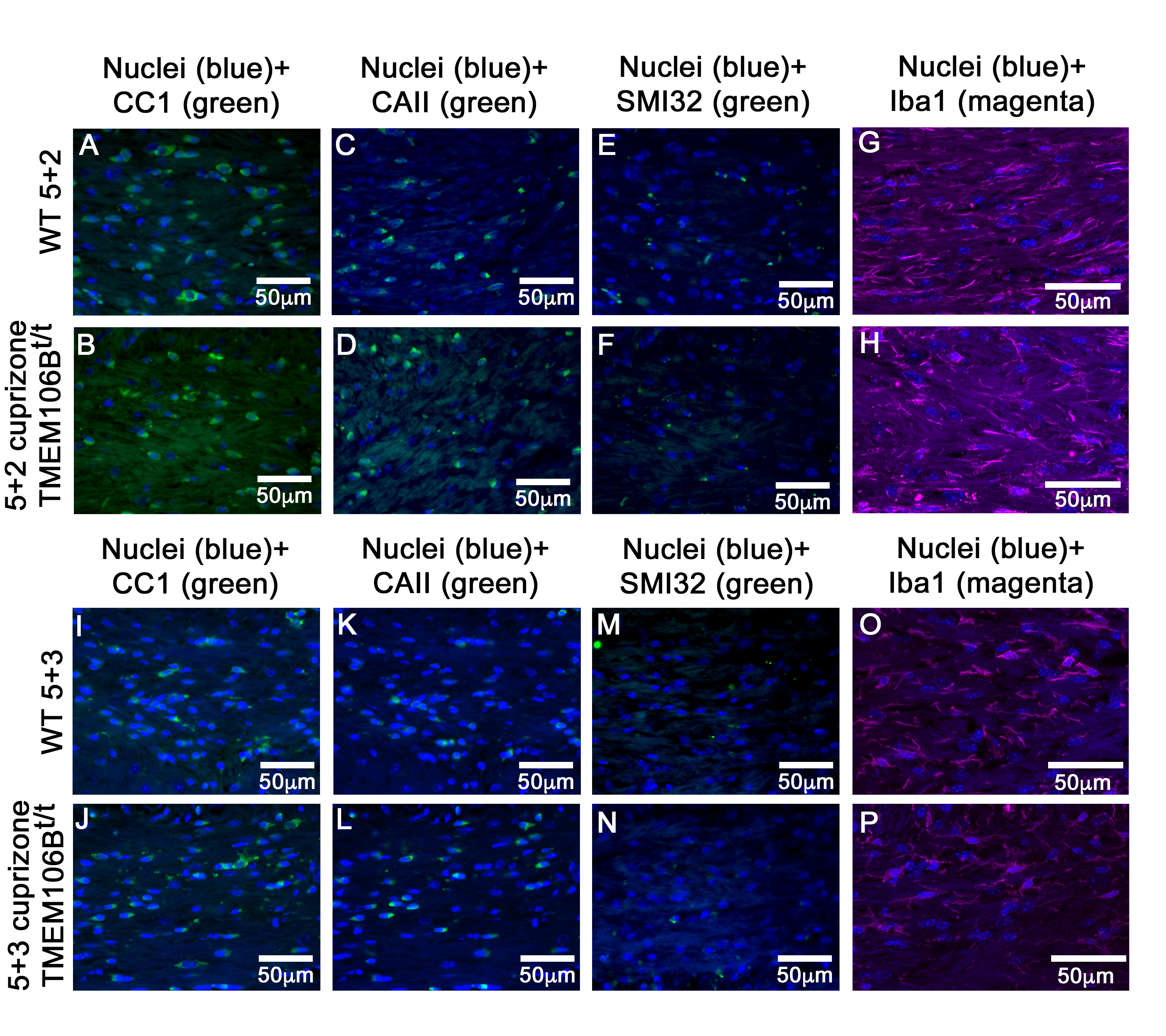

Supplement: Supplementary file 1 [file cells-12-01734-s001.zip › Supplemental Figure S4.tif]
